# Anionic N-Heterocyclic Carbenes from Mesoionic Imidazolium-4-pyrrolides: The Influence of Substituents, Solvents, and Charge on their ^77^Se NMR Chemical Shifts

**DOI:** 10.1021/acs.joc.4c01732

**Published:** 2024-10-03

**Authors:** Lucas Pruschinski, Jan C. Namyslo, Andreas Schmidt

**Affiliations:** Institute of Organic Chemistry, Clausthal University of Technology, Leibnizstraße 6, D-38678 Clausthal-Zellerfeld, Germany

## Abstract

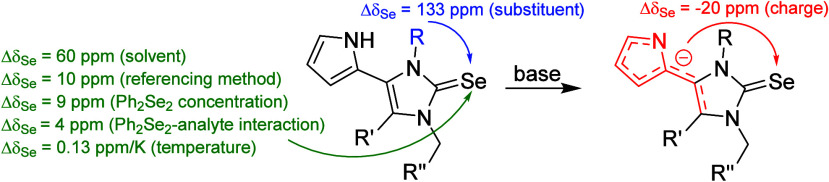

Mesoionic compounds are the starting material for the synthesis of unique anionic N-heterocyclic carbenes. Herein, mesoionic imidazolium pyrrolides synthesized from pyrrole-2-carbaldehyde via various *N*-alkyl-4-pyrroyl-imidazoles are described. These were converted into nine new 4-(pyrrol-2-yl)-substituted imidazolium salts and transformed into the mesoionic title compounds using an anion exchange resin. The DFT-calculated (B3LYP/6-311++G**) CREF values indicate a great potential for the formation of anionic N-heterocyclic carbenes by deprotonation, which were generated and reacted with selenium to obtain selenoureas. The ^77^Se NMR shifts investigated under systematic variation of conditions are dependent on the substitution pattern (Δδ_Se_ = 133 ppm) and the steric demand of the substituents. Solvent dependencies of the ^77^Se NMR shifts were investigated applying toluene-*d*_8_, THF-*d*_8_, CDCl_3_, CD_2_Cl_2_, pyridine-*d*_5_, acetone-*d*_6_, DMSO-*d*_6_, CD_3_CN, AcOD, and MeOD. The influences of the referencing method on the ^77^Se shifts using external or internal Me_2_Se or Ph_2_Se_2_ and solvent can add up to Δδ_Se_ = ca. 80 ppm. In addition, we observed a temperature dependence of both the selenoureas and the reference reagent Ph_2_Se_2_ as well as a ^77^Se shift difference of the analyte caused by interaction with internally added Ph_2_Se_2_. The negative charge of deprotonated selenoureas shifts the values by an additional −20 ppm.

## Introduction

**Figure 1:**
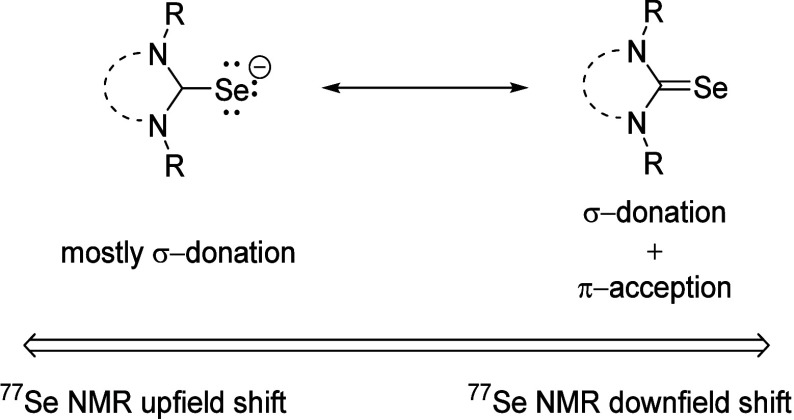
Influence of donor and acceptor properties on the ^77^Se NMR chemical shifts.

Certainly triggered by the isolation of the first stable representative of this substance class by Arduengo in 1991,^1^ intensive research has been carried out in the field of N-heterocyclic carbenes (NHC) for more than three decades.^2^ Today, it is impossible to imagine modern synthetic chemistry, which applies transition metal-catalyzed cross-couplings^3^ including asymmetric reactions,^4^ organocatalytic steps,^5^ or polymer chemistry^6^ without them, to name just a few selected subdisciplines of chemistry. In addition, numerous biological activities of NHC adducts have been found.^7^ In the course of this research, interesting structural variations with respect to the underlying heterocyclic ring system, different substitution patterns and types of carbenes such as normal, abnormal, remote NHCs,^8^ mesoionic carbenes (MIC)^9^ and cyclic alkyl amino carbenes (CAAC)^10^ have been studied and applied. A number of approaches have also been developed to quantify the properties of N-heterocyclic carbenes and their precursor molecules and thus predict them for customized applications. Thus, in order to quantify the ability of heterocyclic precursors to form carbenes, the CREF values (carbene relative energy of formation) of heterocycles, heteroaromatics and mesomeric betaines, which can in principle form N-heterocyclic carbenes by deprotonation, were determined.^11^ As consequence, various methods are available to characterize the electronic properties of N-heterocyclic carbenes themselves. These include the Tolman parameter (TEP),^12^ Huynh parameter,^13^ proton affinities, molecular electrostatic potentials (MESP),^14^ computationally derived electronic ligand parameters (CEP),^15^^1^*J*_C–H_ and ^1^*J*_C–Se_ coupling constants, and selenium NMR shifts,^18^ which are used with respect to the π-acceptor and σ-donor properties of the resulting NHCs. The basic idea of using ^77^Se NMR resonance frequencies in this context is that carbenes with high σ-donicity lead to selenones in which the ^77^Se atom is more shielded and therefore an upfield shift of the signals occurs, whereas in π-acceptor carbenes the resulting selenones are more deshielded and therefore a downfield shift is observed (Figure 1).^16−19^

**Figure 2:**
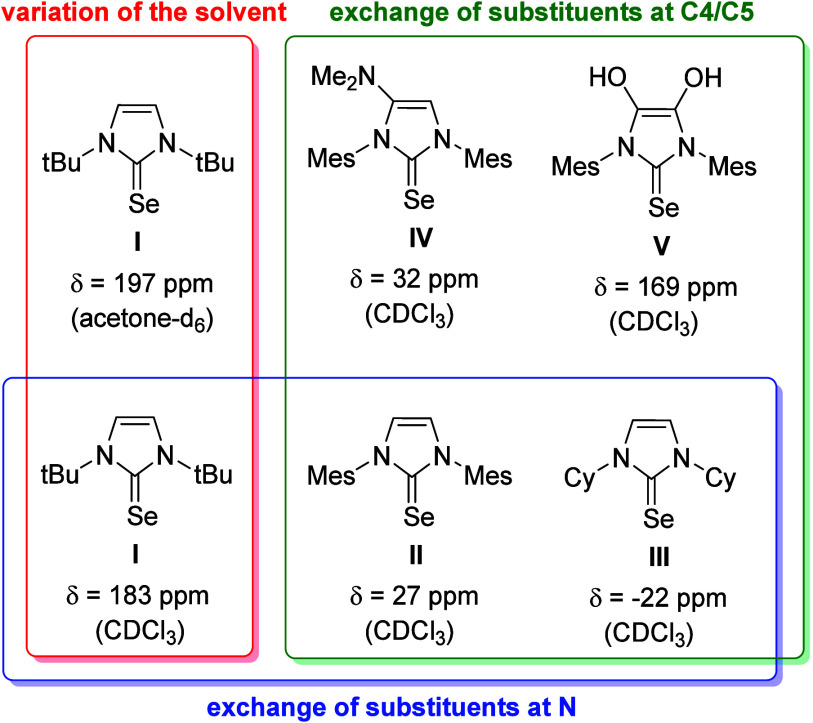
Solvent and substituent influences affect the ^77^Se NMR shifts.

The literature occasionally mentions that, in addition to the structure, the temperature,^17^ the pH value, the analyte concentration and the solvents also influence the position of the ^77^Se NMR resonance frequencies.^18,19^ As can be seen from Figure 2, the ^77^Se NMR shifts do indeed change with the change of solvent in the few examples studied so far. For example, the ^77^Se NMR signal of 1,3-di-*tert*-butyl-imidazole-2-selenone **I** appears at δ = 197 ppm in acetone-*d*_6_ and at δ = 183 ppm in deuterated chloroform. In the literature, selenium shifts of selenoureas derived from N-heterocyclic carbenes are found in a variety of solvents ranging from CDCl_3_,^20^ acetone-*d*_6_,^21^ CD_3_CN,^22^ THF-*d*_8_^23^ to DMSO-*d*_6_.^24^ The exchange of substituents on the N atoms of the imidazole for substituents with different steric requirements and electronic properties results in considerable changes in the ^77^Se NMR shifts. For example, the shift differences when exchanging the *tert*-butyl substituents of **I** to cyclohexyl (**III**), whose *E*_s_ values (Cy = −2.03; *t*Bu = −2.78)^25^ and A values (Cy: 2.15; *t*Bu: > 4)^25^ as a measure of steric hindrance differ, are around Δδ = 205 ppm into the high field. If Hammett’s σ parameter is used as a measure of the electronic properties, the values of the two substituents differ only slightly (Cy: σ_m_ = −0.05; σ_p_ = −0.15; *t*Bu: σ_m_: −0.1, σ_p_: −0.2),^26,27^ but it is undoubtedly too simple to reduce substituent effects on the ^77^Se NMR shifts to the parameters captured by these numbers. The aromatic mesityl residue on both N atoms causes a resonance frequency at δ = 27 ppm in deuterated chloroform. The substituents on the imidazole ring system, which are located at positions C4 and C5, have a further influence. Examples **IV** and **V** from Figure 2 carry one and two donor substituents, respectively, with Hammett values of σ_m_ = +0.12/σ_p_ = −0.37 (OH, respectively) and σ_m_ = −0.16/σ_p_ = −0.83 (NMe_2_, respectively), which are known to combine inductive, mesomeric and field effects in one value, and show different shifts in CDCl_3_ around Δδ = 137 ppm.

**Figure 3:**
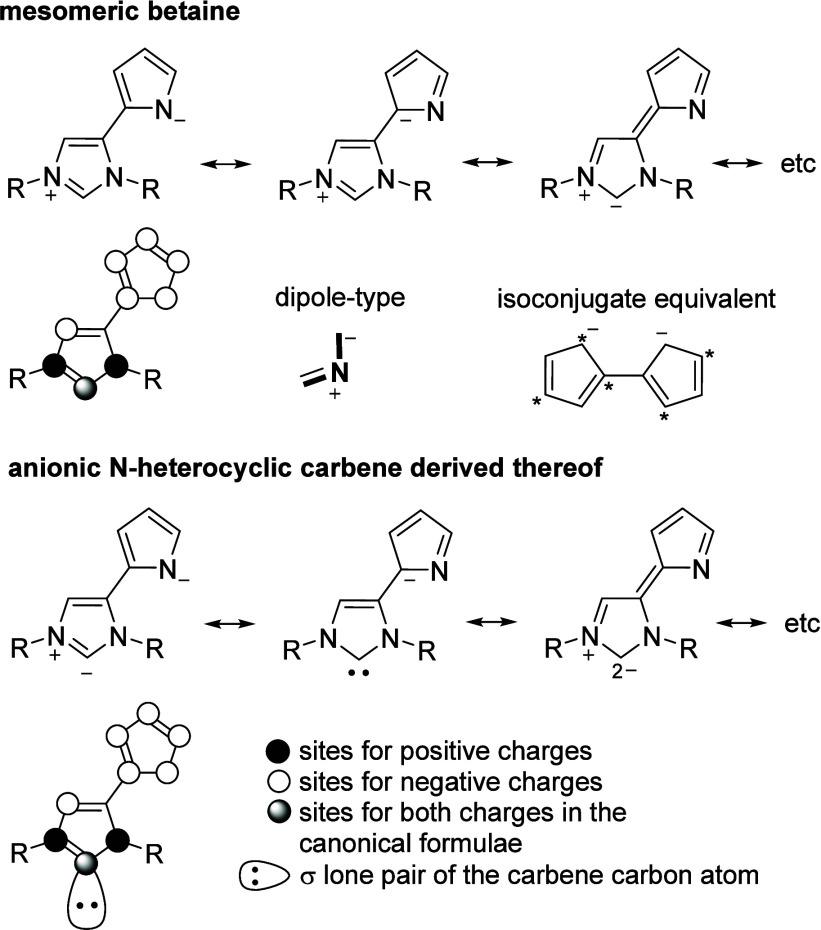
Classification of the target betaines and of the anionic NHCs derived thereof.

Overall, the comparison with systems known from the literature shows that further systematic examples and data obtained from them are still lacking,^12b^ especially those involving charges and conjugation types. Therefore, mesomeric betaines are valuable model compounds to study potential influences. The aim of our work was therefore to develop new mesomeric betaines of imidazole whose anionic structural increment is conjugated to the hetarenium ring and thus directly influences the potential carbene center. For reasons of stability, a heteroaromatic should serve as the anionic structural element, which can additionally stabilize the negative charge by delocalization. The title compounds, imidazolium-4-pyrrolides, appeared to be well suited for this purpose. As illustrated in Figure 3, according to the rules of resonance, the negative charge delocalizes not only within the anionic pyrrole ring, but can also be formulated on C2 and C5 of the imidazole ring. This fact, in combination with the characteristic dipole increment and the isoconjugate equivalent, defines the molecule as a conjugated mesomeric betaine,^28^ strictly speaking as a mesoionic compound denoting the 5-membered rings within this class.^29^ Five different classes of mesomeric betaines are now distinguished.^30^ Of these, the conjugated mesomeric betaines are likely to have a particularly significant influence on the carbene center via both inductive and mesomeric effects.^31^

Continuing our interest in the chemistry of mesomeric betaines^32^ and related structures, in particular their ability to form N-heterocyclic carbenes and their anionic analogs,^33^ we report here on the syntheses of the above-mentioned new target betaines, on their tendency to carbene formation, and on the ^77^Se NMR resonance frequencies of the selenium adducts of these carbenes.

## Results and Discussion

**Scheme 1:**
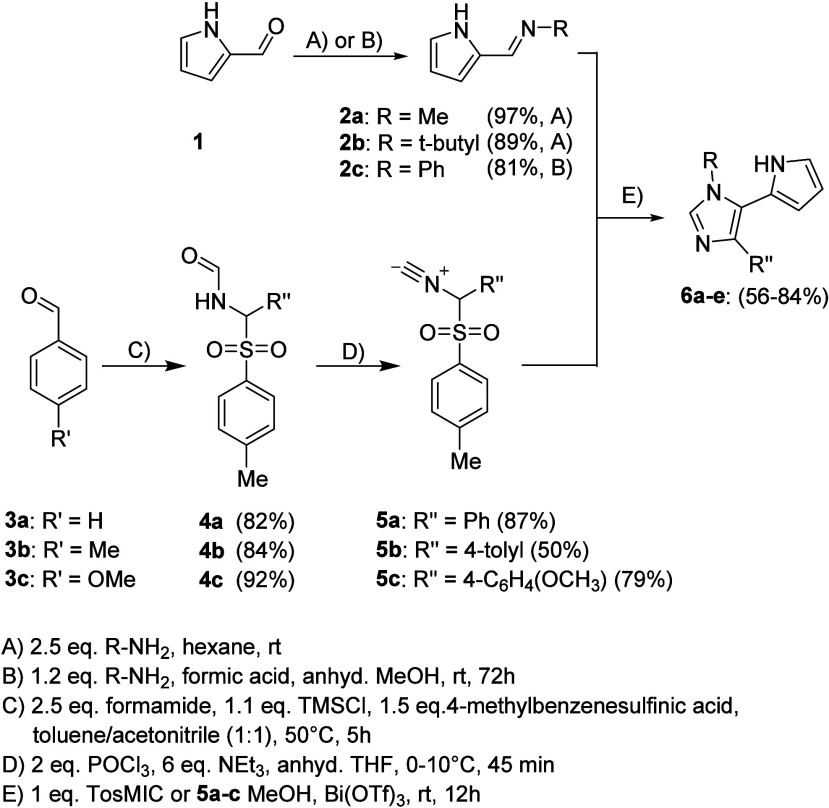
Synthesis of Imidazoles **6a**–**e**

The retrosynthetic analysis indicated that the target betaines could best be synthesized from the corresponding imidazolium salts, where the imidazolium salts should be prepared by quaternization of the corresponding 1,5-disubstituted and 1,4,5-trisubstituted imidazoles, which can be synthesized via a bismuth(III) trifluoromethanesulfonate-catalyzed van Leusen reaction as shown in Scheme 1.^34,37^ Thus, starting from pyrrole-2-carbaldehyde **1**, *N*-alkyl or *N*-aryl imines **2a**–**c** were prepared by reacting the aldehyde with primary amines. In the case of the *N*-alkylimines **2a,b**, these were easily prepared by adding the corresponding amine to a solution of the aldehyde in hexane at room temperature (procedure A). Synthesis of the *N*-aryl imine **2c** required the reaction to be carried out in methanol under reflux conditions and the addition of catalytic amounts of formic acid (procedure B). The formamides **4a**–**c** resulted from the reaction of formamide with *p*-toluenesulfonic acid and the corresponding benzaldehydes in the presence of TMSCl^34^ (method C) and served as starting materials for the synthesis of the TosMIC derivatives **5a**–**c**. These were prepared easily and with moderate to good yields by dehydration using POCl_3_ and trimethylamine (procedure D).^35^ The pyrrole imines **2a**–**c** were then reacted either with TosMIC to give 1,5-disubstituted imidazoles or with the modified TosMIC derivatives **5a**–**c** to give 1,4,5-trisubstituted imidazoles **6a**–**e**. All ring closure reactions were carried out in methanol at room temperature using *tert*-butyl amine as a base, with a reaction time of 12 h (procedure E, van Leusen reaction). Substitution patterns and yields are summarized in Table 1.

**Table 1:** Yields of Imidazoles **6a**–**f**

compd	imine	TosMIC derivative	R	R”	yield (%)
**6a**	**2a**	**TosMIC**	Me	H	75
**6b**	**2b**	**TosMIC**	*tert*-butyl	H	60
**6c**	**2a**	**5a**	Me	Ph	75
**6d**	**2a**	**5b**	Me	4-tolyl	56
**6e**	**2a**	**5c**	Me	4-C_6_H_4_(OCH_3_)	84
**6f**^a^	**2c**	**TosMIC**	Ph	H	0

a Reaction was performed with both *tert*-butylamine and K_2_CO_3_.

**Scheme 2:**
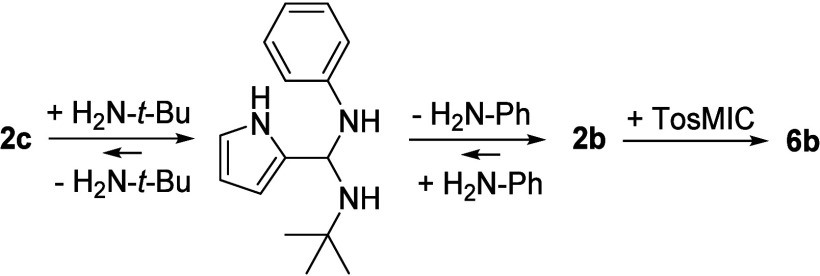
Transimination of **2c** via Aminal Formation during Ring Closure Reaction in the Presence of *tert*-Butylamine

While the syntheses of the *N*-alkyl imidazoles **5a**–**e** were successful, the *N*-aryl imidazole **5f** for the synthesis of **6f** could not be obtained by this reaction route. In fact, the reaction of imine **2c** according to the method shown above yielded the same imidazole as in the case of imine **2b** by an undesired transamination reaction (Scheme 2). Such reactions of imines in the presence of other amines are known from the literature.^36^ In order to suppress this, attempts were made, albeit unsuccessfully, to carry out the ring closure reaction of derivative **2c** with potassium carbonate.^36^

**Scheme 3:**
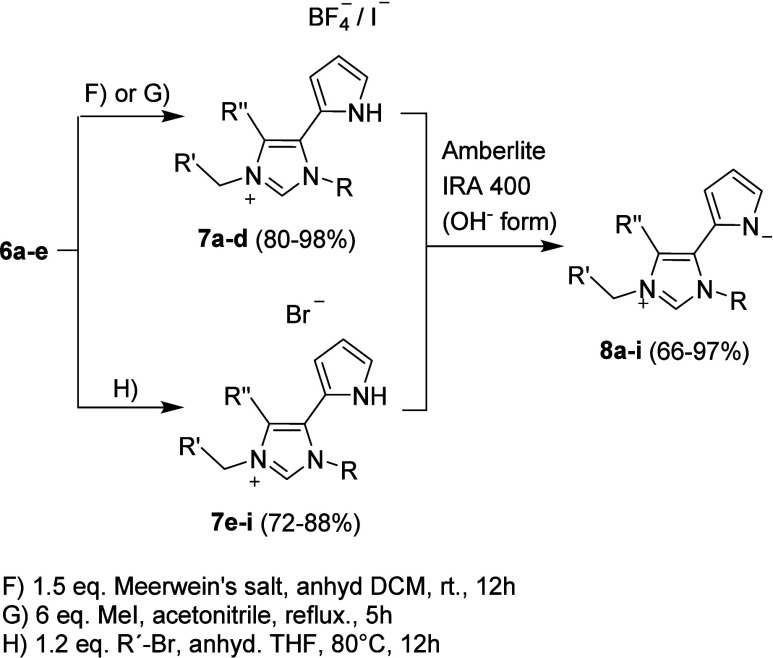
Syntheses of Imidazolium Salts **7a**–**i** and Mesomeric Betaines **8a**–**i**

To synthesize the imidazolium salts, the resulting compounds **6a**–**e** were either *N*-methylated or *N*-benzylated (Scheme 3). Methylation was carried out in case of **7a** with Meerwein’s reagent in anhydrous DCM at room temperature for 12 h, during which time the salt precipitated (procedure F). For the imidazolium salts **7b**–**d**, a reaction with Meerwein’s reagent is excluded, as they cannot be dissolved in solvents suitable for the reaction. The synthesis was therefore carried out in good to almost quantitative yields by reacting the imidazoles with excess methyl iodide in dry acetonitrile under reflux conditions. The quaternization by benzylation was carried out by reaction with the corresponding benzyl bromides in anhydrous THF under reflux, and similarly to the methylation, the corresponding salts **7e**–**i** were obtained in good yields. The imidazolium salts **7a**–**k** were then converted to the corresponding mesomeric betaines using the anion exchange resin Amberlite IRA 400 in its hydroxide forms.^38^ This was successful for all derivatives in good to very good yields (c.f. Table 2).

**Table 2:** Yields of Imidazolium Salts **7a**–**i** and Betaines **8a**–**i**

compd.	R	R’	R”	yield (%)
**7a/8a**	Me	H	H	98/97
**7b/8b**	Me	H	Ph	80/77
**7c/8c**	Me	H	4-tolyl	81/74
**7d/8d**	Me	H	4-C_6_H_4_(OCH_3_)	86/82
**7e/8e**	Me	Ph	H	76/91
**7f/8f**	Me	4-tolyl	H	78/94
**7g/8g**	Me	4-C_6_H_4_(OCH_3_)	H	79/92
**7h/8h**	*tert*-butyl	Ph	H	72/93
**7i/8i**	*tert*-butyl	4-tolyl	H	88/94

**Scheme 4:**
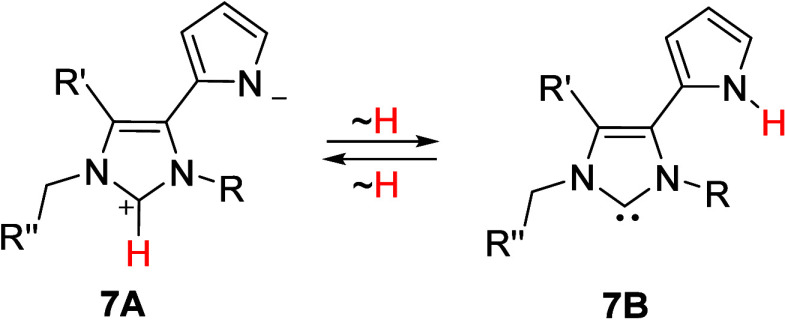
Mesomeric und Tautomeric Structures of Betaines **7a**–**i**

NMR-spectroscopic investigations, which were only possible in DMSO-*d*_6_ or CD_3_OD due to the high polarity of the betaines investigated, provided no evidence for a betaine-carbene tautomerism **7A** ↔ **7B** (Scheme 4). A similar NMR spectroscopic behavior was observed for other imidazole-based mesomeric betaines such as quinazolin-4-one imidazolium betaines^39^ and purine imidazolium betaines.^40^

**Figure 4:**
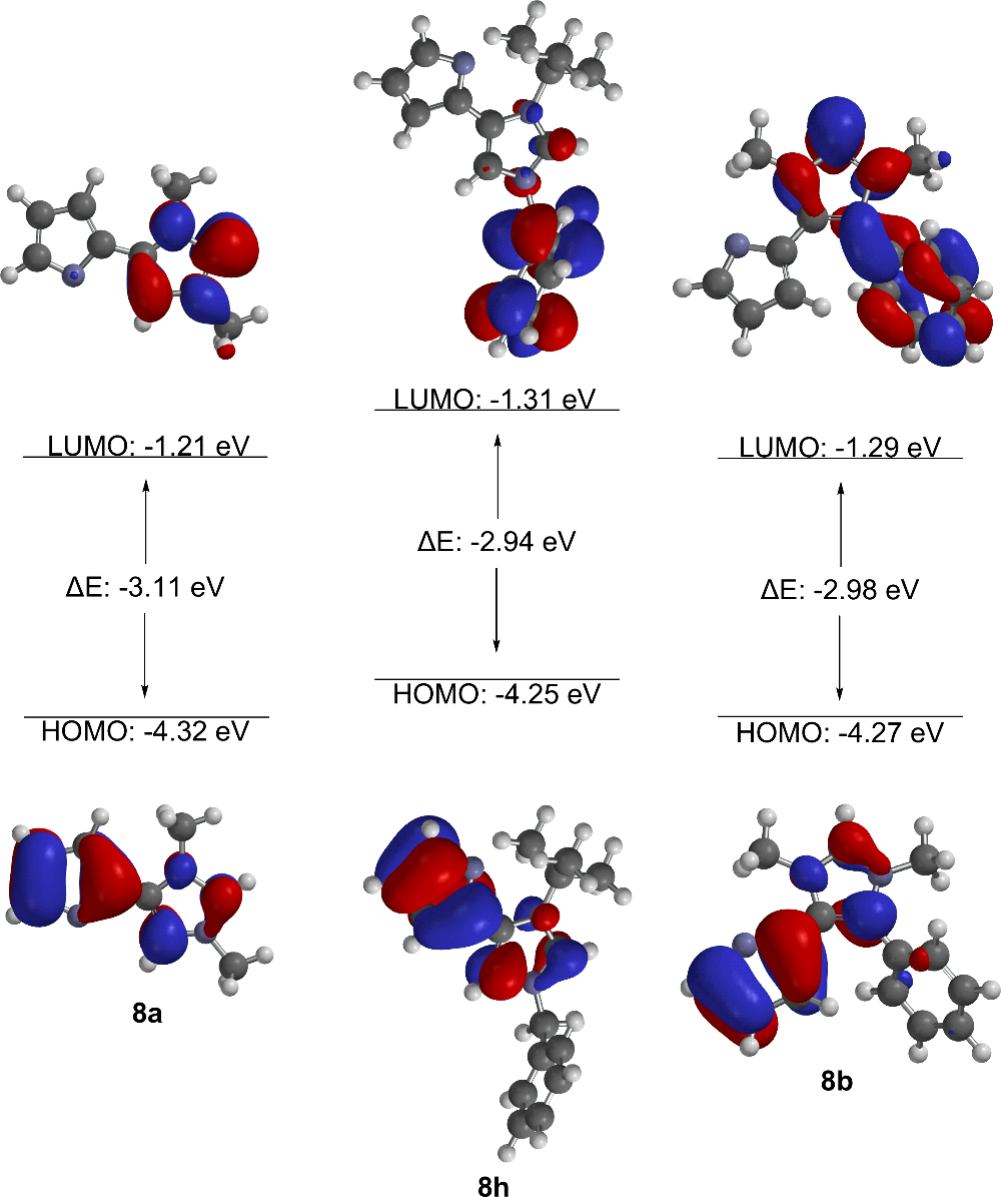
Selected HOMO/LUMO profiles of the imidazolium-4-pyrrolides.

The calculated frontier orbital profiles (B3LYP/6-311++G**) show a characteristic architecture of conjugated mesomeric betaines. Typically, the frontier orbitals are essentially located in the same parts of the molecule and this is undoubtedly valid at least for the calculated HOMOs of the betaines (Figure 4). As expected, the anionic pyrrolide ring has the larger atomic orbital coefficients in comparison to the imidazole ring. It is noteworthy that, according to the calculation, the phenyl ring in **7b** has hardly any atomic orbital coefficients in the HOMO, but strong ones in the LUMO despite the torsion angle of τ = 55.7°. On the other hand, this matches the LUMO profile of the *N*-benzylated derivative **7h**, whose LUMO is mainly localized in the benzene ring of the benzyl substituent. The HOMOs have large atomic orbital coefficients at the C2 positions of the imidazolium ring of the betaines, which form the carbene carbon atoms during subsequent deprotonation.

In order to estimate the ability of the mesoionic compounds to form anionic N-heterocyclic carbenes, the CREF (carbene relative energy of formation) of the compounds were then determined.^11^ All CREF values of the imidazolium betaines were calculated at the B3LYP/6-311++G** level to ensure comparison with values from the literature.^11^ Surprisingly, the CREF values of the derivatives considered in this work (Table 3) differ only slightly, since the values obtained are apparently neither significantly influenced by the substituents at the 4-position of the imidazole **7b**-**7d** nor by the different substituents at the two nitrogen positions of the imidazole. Apparently, they are determined exclusively by the π-character of the betainic system. Compared to other anionic imidazole-based NHCs which are derived from mesomeric betaines, such as 1,3-dimethylimidazolium-4-aminide (CREF = 0.557)^11a^ or 1,3-dimethylimidazolium-4-olate (CREF = 0.576),^11a^ the derivatives of this work show lower CREF values, which makes them promising carbene precursors among the anionic imidazolium-based NHCs.

**Table 3:** CREF-Values of the Betaines **7a**–**i**

compd.	CREF	compd.	CREF
**8a**	0.546	**8d**	0.547
**8b**	0.545	**8e**	0.545
**8c**	0.546	**8f**	0.547

The mesomeric betaine **7a** was selected as a reference compound for base screening to find the optimal conditions for the formation of selenourea derivatives of the NHCs (Table 4). The best results were obtained by reactions in anhydrous MeCN with 2 eq. of cesium carbonate as base under reflux at a reaction time of 12 h. All other betaines **8b**–**8i** were also converted to selenones **9b**–**i** under these conditions. Although the formation of the abnormal carbene at the C-5 position of the imidazole is conceivable for R″ = H,^41^ no products other than those described were obtained under the selected conditions.

**Table 4:**
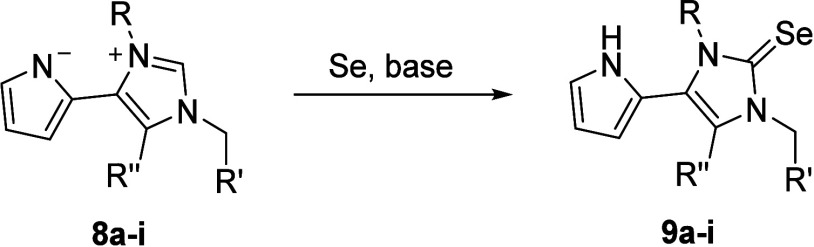
Base Screening for Selenium Trapping Reactions

educt	selenone	solvent	base	temperature	time	yield (%)
**8a**	**9a**	THF	LHMDS	–10 °C→rt	1 h −10 °C/5 h rt	38
**8a**	**9a**	THF	LHMDS	–10 °C→rt	1 h −10 °C/12 h rt	32
**8a**	**9a**	THF	KHMDS	–10 °C→rt	1 h −10 °C/5 h rt	35
**8a**	**9a**	MeCN	KO*t*-Bu	85 °C	5 h	25
**8a**	**9a**	MeCN	Cs_2_CO_3_	85 °C	5 h	40
**8a**	**9a**	MeCN	Cs_2_CO_3_	85 °C	12 h	68
**8b**	**9b**					28
**8c**	**9c**					52
**8d**	**9d**					49
**8e**	**9e**					42
**8f**	**9f**					59
**8g**	**9g**					49
**8h**	**9h**					51
**8i**	**9i**					49

**Figure 5:**
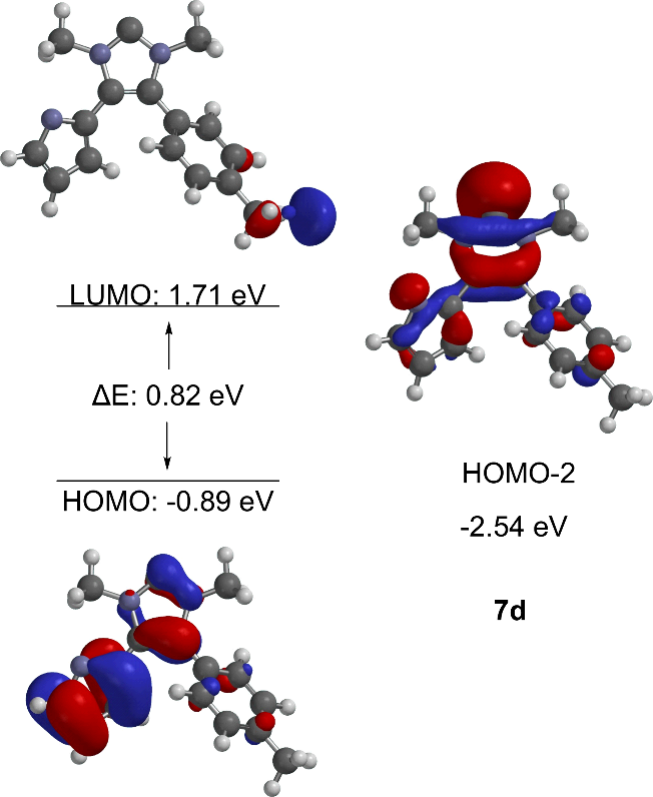
Selected HOMO/LUMO profiles of the anionic N-heterocyclic carbene.

In the pdf of the text, it appears as if the syntheses of 8b-8i have no conditions (Table 4). Is it possible to visualize this graphically so that it is clear that the same conditions apply to 8a to 8i? Perhaps by drawing a line above 8a and placing the conditions in the middle between 8a and 8i? As far as the HOMO profiles of the carbenes are concerned, they behave similarly to the betaines from which they are formed. First, it should be noted that the HOMO of the anionic N-heterocyclic carbenes is a π-orbital. Finally, HOMO–2 shows the orbital characteristic of carbenes, which lies in the plane of the heterocycle. The π-orbital of the HOMO is localized in both parts of the molecule. As in the mesomeric betaine precursor, a large atomic orbital coefficient is located on the carbene carbon atom (Figure 5).

**Figure 6:**
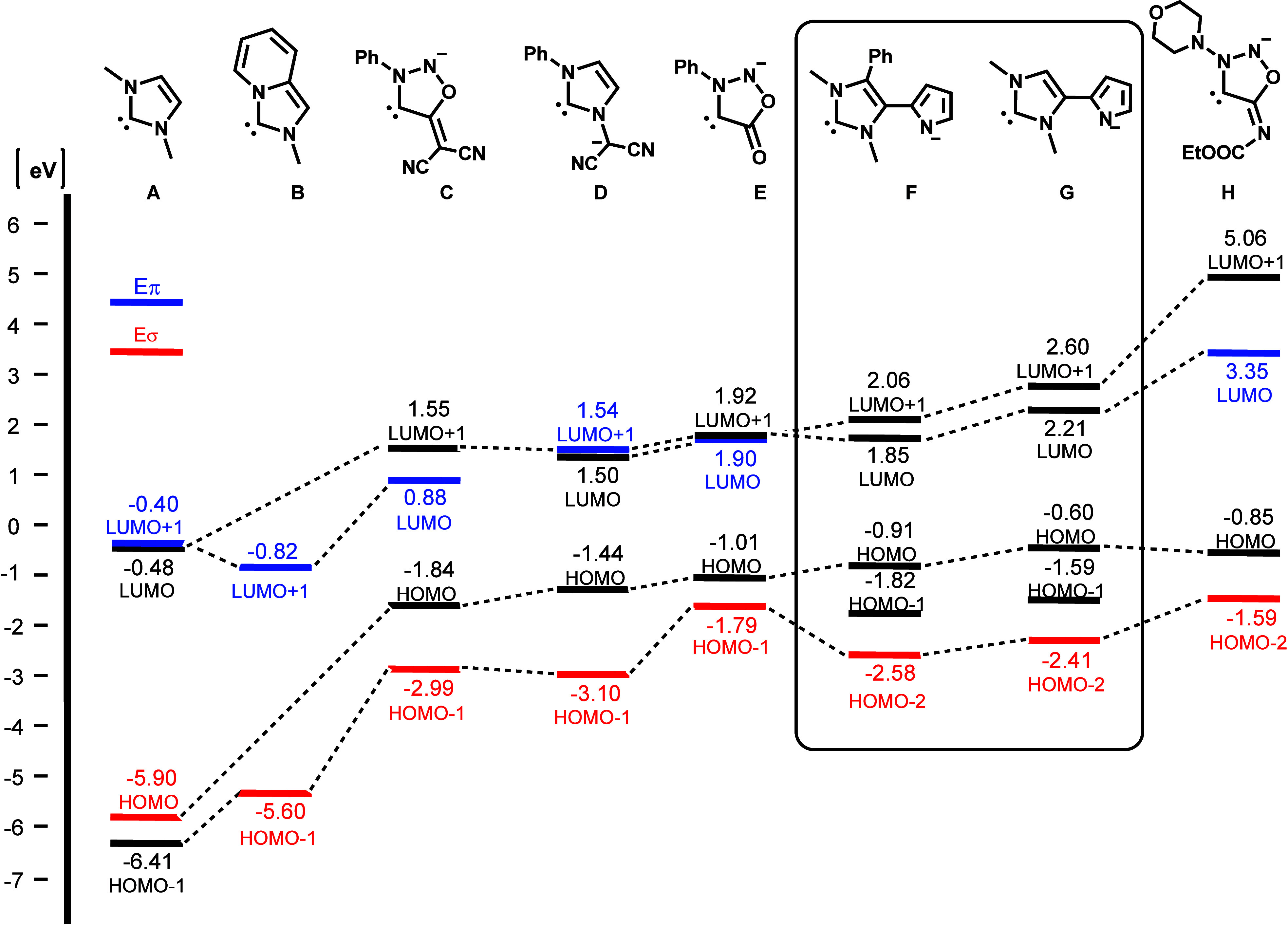
Comparison of selected molecular orbital energies calculated at B3LYP/6-311++G** level in vacuo of imidazol-2-ylidene, imidazol[1,5-*a*]pyridin-3-ylidene and carbenes of the sydnone family with selected carbenes of imidazolium-4-pyrrolides.

In comparison to classical neutral imidazole-based carbenes, such as **A**(33c) and **B**,^16^ it is evident that the carbenes of the imidazolium-4-pyrrolides described here (**F**, **G**) exhibit significantly higher HOMO energies. In this regard, these compounds inherit a similar behavior to other π-electron-rich anionic NHCs, including the sydnones (**E**),^33c^ sydnone methides (**C**),^33c^ imidazole *N*-ylides (**D**)^24a^ and sydnonimines (**H**).^32b^ (Figure 6). A feature of imidazolium-4-pyrrolides in comparison to the aforementioned NHC systems is that, as previously mentioned, the HOMO–2 exhibits the conventional carbene geometry. It should be noted that the calculations did not show any coefficients of the LUMO, LUMO+1, and LUMO+2 of compounds **F** and **G** that would allow identification as π-acceptor (Eπ).

N-Heterocyclic carbenes are known to be good electron donors, which is the reason for their broad application in chemistry mentioned at the beginning.^2−6^ The bonding between NHCs and transition metals occurs through σ- and π- interactions, which are attempted to be quantified using the methods listed in the introduction.^11−18^ However, the evaluation of the ^77^Se NMR resonance frequencies appears to be complicated.^12b^ Considering the importance of the ^77^Se resonance frequencies of selenoureas for the characterization of N-heterocyclic carbenes, we first systematically investigated the influence of the solvent in order to compare them with the results for various other selenium compounds.^42−44^ For this purpose, the ^77^Se NMR shifts of the selenones **9a**, **9b**, **9e** and **9h** were measured in a selection of common NMR solvents of different properties. All samples were measured at the same temperature. We chose a uniform concentration of 0.17 M selenium compound, because results known from the literature^42^ show that below a concentration of 2.0 M there is no concentration dependence of the ^77^Se NMR resonance frequencies, at least for compounds such as diselenides.^43^ We also used the measurements of **9a** to investigate whether different referencing methods have an influence on the ^77^Se NMR shift. Three recommendations have been made by IUPAC regarding the calibration of NMR shifts, and these are internal and external referencing and the substitution method.^45^ Especially in ^77^Se NMR spectroscopy, external referencing and the substitution method are widely used.^17,42,46^

**Figure 7:**
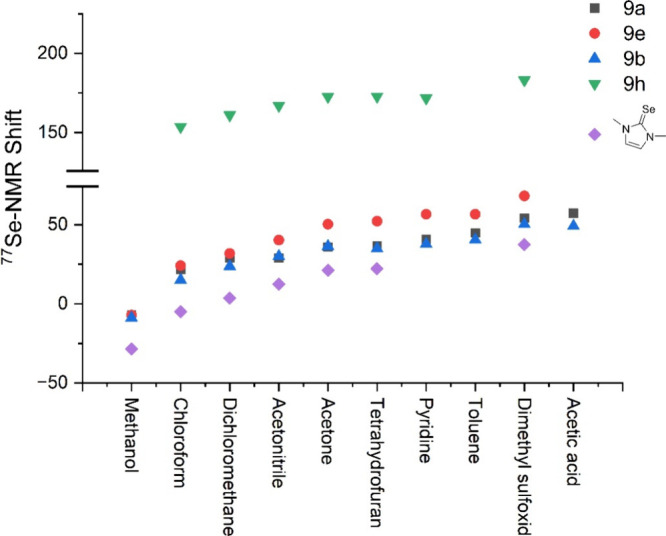
^77^Se NMR chemical shift changes in different solvents. Solvents were sorted in the order of ascending ^77^Se shift values.

The spectra of the compounds mentioned above were referenced externally against PhSe-SePh^49^ and internally by adding SeMe_2_. Since our investigations have shown that the ^77^Se NMR shift of the reference substance PhSe-SePh depends on the solvent used, we have decided to always carry out the referencing in the same solvent in which the analyte under consideration is measured. The signal of the external reference is fixed at 461.0 ppm relative to Me-Se-Me @ 0.0 ppm. For more detailed information on the behavior of the ^77^Se NMR shifts of PhSe-SePh in the various solvents, please refer to the Supporting Information (Table S1, p. S4). The latter method with the toxic SeMe_2_ is very unpleasant due to the strong odor. Findings on how this reagent can be avoided are therefore certainly of particular importance. The values summarized in Table 5 are shown graphically in Figure 7.

**Table 5:** Selenium NMR-Shifts of **9a**, **9b**, **9e,** and **9h** in Different NMR Solvents^a^

NMR solvent	^77^Se NMR shift of **9a** referenced to SeMe_2_^b^	^77^Se NMR of **9a** shift referenced to PhSe-SePh^c^	^77^Se NMR of **9b** shift referenced to PhSe-SePh^c^	^77^Se NMR of **9e** shift referenced to PhSe-SePh^c^	^77^Se NMR of **9h** shift referenced to PhSe-SePh^c^	*E*_T_^N^
Tol-*d*_8_	44.9	44.0	56.6	40.5		0.099
THF-*d*_8_	35.8	36.6	52.3	34.9	172.8	0.207
CDCl_3_	21.7	12.5	24.2	15.1	153.6	0.259
CD_2_Cl_2_	20.9	19.0	31.9	23.7	161.0	0.259
Pyridine-*d*_5_	39.8	40.8	56.6	37.8	171.7	0.302
acetone-*d*_6_	35.9	35.9	50.3	36.4	172.7	0.309
DMSO-*d*_6_	54.2	52.1	68.2	50.3	183.1	0.355
CD_3_CN	29.0	27.6	40.3	30.0	167.0	0.444
AcOD	52.4	57.4		49.2		0.648
MeOD	–7.0	–13.8	–7.1	–8.8		0.762

a *E*_T_^N^ values of solvent polarities as information.

b Internal reference @ 0.0 ppm.

c External reference @ 461.0 ppm rel. to Se(CH_3_)_2_ @ 0.0 ppm.^49^ In the pdf, you can see that d5 in Pyridine-d5 is on a separate line. This is only cosmetic, but if it were on the same line, it would look better because it would eliminate a blank line.

First of all, it is noticeable that the reference reagent can have an undeniable influence on the determination of the chemical shift in ^77^Se NMR spectroscopy, but this depends strongly on the solvent. The ^77^Se NMR signals determined in CDCl_3_ proved to be particularly sensitive to the referencing reagents, which led to widely differing values. In contrast, the signals of **9a** are unaffected if the measurements are carried out in acetone-*d*_6_, for example. It is known that the ^77^Se NMR resonance frequency of SeMe_2_ depends on the concentration (Δδ = 9 ppm from neat to very dilute), on the solvent [δ(C_6_H_12_) - δ(DMSO) > 20 ppm] and on the temperature (Δδ = 2.5 ppm above 100 °C), so that a 60% solution of dimethylselenide in CDCl_3_ was proposed as a suitable reference reagent.^47^

**Figure 8:**
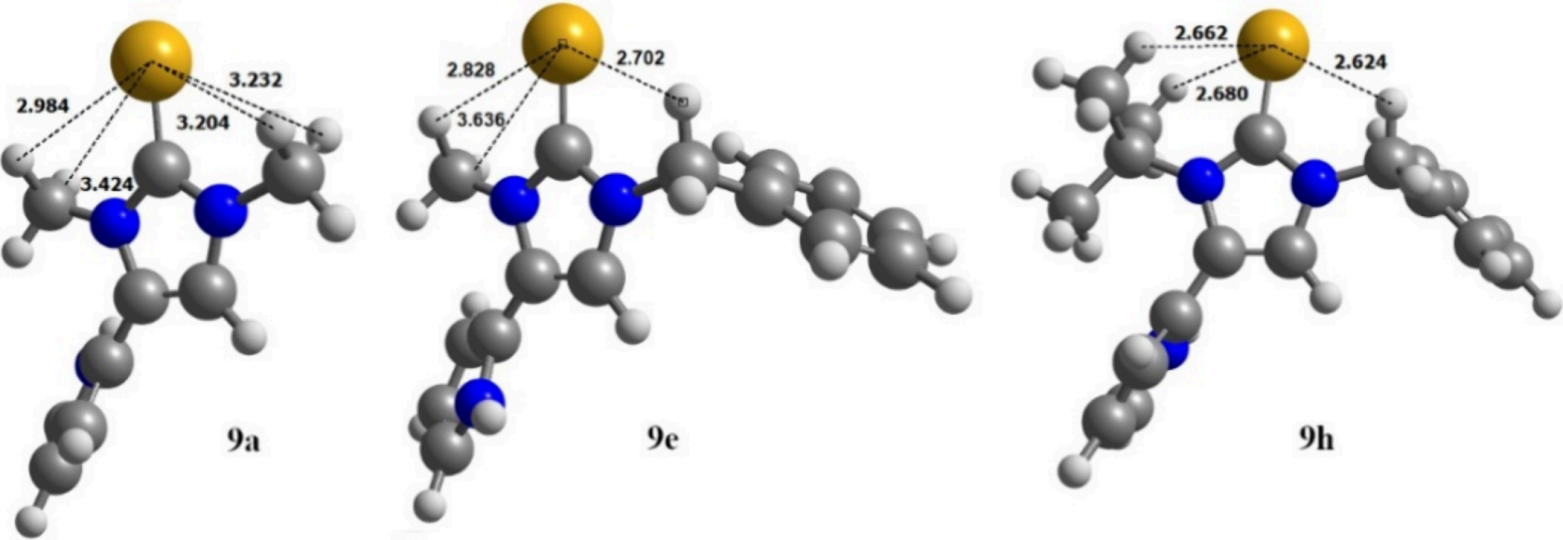
Calculated structure of selenones **9a**, **9e,** and **9h**.

In line with previous results the choice of solvent has a considerable influence on the NMR shifts obtained. The ^77^Se NMR shifts change roughly in parallel and follow a similar trend on changing the solvent (Figure 7). This also applies to 1,3-dimethylimidazole-2-selenone, which we also examined for comparison. The measured ^77^Se NMR resonance frequencies do not correlate with the known empirical parameters for the determination of solvent polarity such as the *Z*-values^50,51^ or *E*_T_^N^ values,^50,52^ which are listed in Table 5 for information and refer to the nondeuterated solvents. The solvent influence on the ^77^Se NMR shifts cannot be reduced to the parameters that are based on the linear free enthalpy relationships to determine these solvent polarity scales, and this is consistent with results on diselenides, for example.^43^ Numerous other classifications of solvents exist. Thus, our selection included protic (MeOD) and nonprotic, hydrogen bond donating (MeOD) and accepting (DMSO, acetone) and π-donating solvents (toluene). Acetonitrile, tetrahydrofuran, acetone and DMSO, for example, are also more basic than chloroform with corresponding Δδ_∞_ values of 0.56, 0.79, 0.92, and 1.32 respectively.^53^ The donor numbers (donicities), which describe the nucleophilic properties of electron-pairing solvents, also differ, as can be seen from the normalized *D*_N_^N^ values (MeCN: 14.1 kcal/mol, DMSO: 29.8 kcal/mol).^54^ A simple correlation between all these classification numbers and the observed chemical shifts of ^77^Se is not recognizable, which is obviously the sum of various influences of different weightings. The influence of the substitution pattern on the ^77^Se NMR resonance frequencies also does not follow a simple pattern in literature examples^43^ and the substituent effects are not additive in this respect.^48^ Based on the results described above, we decided to carry out the ^77^Se NMR measurements of the selenones from this work consistently in DMSO-*d*_6_, in which all compounds are soluble. Table 6 shows the ^77^Se NMR shifts obtained. With the exception of the derivatives **9h** and **9i**, all ^77^Se NMR shifts are in a range between 50.3 and 68.2 ppm, with those selenones substituted in the 4-position showing slightly higher shifts. The *N*-benzylated products, on the other hand, behave similarly to the methylated selenone **9a**. The rather high shifts of the derivatives **9h** and **9i** are surprising; this phenomenon has already been observed in other publications in connection with *tert*-butyl or adamantyl substituents on the imidazole nitrogen.^21b,55^ The reasons for this phenomenon do not yet seem to be clearly understood. Anisotropy effects, paramagnetic effects and C(sp^3^)–H-Se-interactions arising from a negative hyperconjugation between the lone pair of selenium and the σ*_C–H_ orbital are being discussed in this context. The fact is that DFT calculations (B3LYP/6-311++G**) of the energetically most favorable structures of **9a,e,h** result in planar imidazole rings in all three structures, in which the selenium also lies in the same plane, despite different steric requirements (Figure 8). The C–Se distance is about 1.84 Å in all structures and this distance corresponds to that of other selenones. The angle between the C-2 position and the nitrogen substituents is also similar in all molecules and lies between 123° and 127°. The pyrrole substituent is twisted toward the ring plane of the imidazole in all derivatives, as is the benzyl substituent in **9e**. The sum of the van der Waals radii of hydrogen (1.2 Å)^56^ and selenium (1.9 Å)^57^ (Σ = 3.1) is larger than the smallest distance between these atoms in **9a** (2.98–3.42 Å). In the derivative **9e**, the nearest proton of the methylene group of the benzyl substituent is at a distance of 2.70 Å, while the distance to the methyl protons is between 2.82 and 3.63 Å. In the case of the *N*-*tert-*butyl derivative **9h**, two protons of the methyl groups have a distance of 2.68 Å and the distance to the methylene protons of the benzyl group is 2.62 Å, which are the smallest within this series of investigated derivatives.

**Table 6:**
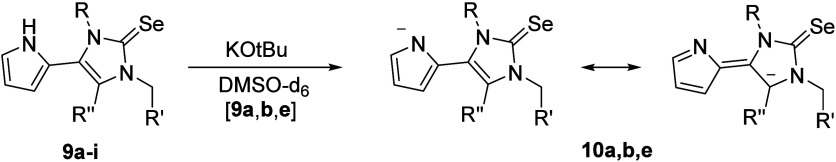
Selenium NMR Shifts of the Selenium Adducts **9a-I** and of the Anions **10a,b,e** (Vide Infra) Measured in DMSO-*d*_6_ (Externally Referenced to PhSe-SePh @ 461.0 ppm rel. to SeMe_2_ @ 0.0 ppm^49^)

compd.	^77^Se NMR shift	compd.	^77^Se NMR shift	compd.	^77^Se shift (ppm)	compd.	^77^Se shift (ppm)
**9a**	52.1	**9d**	66.0	**9g**	50.3	**10a**	31.8
**9b**	68.2	**9e**	50.3	**9h**	183.1	**10b**	48.9
**9c**	67.2	**9f**	50.4	**9i**	183.3	**10e**	32.7

**Figure 9:**
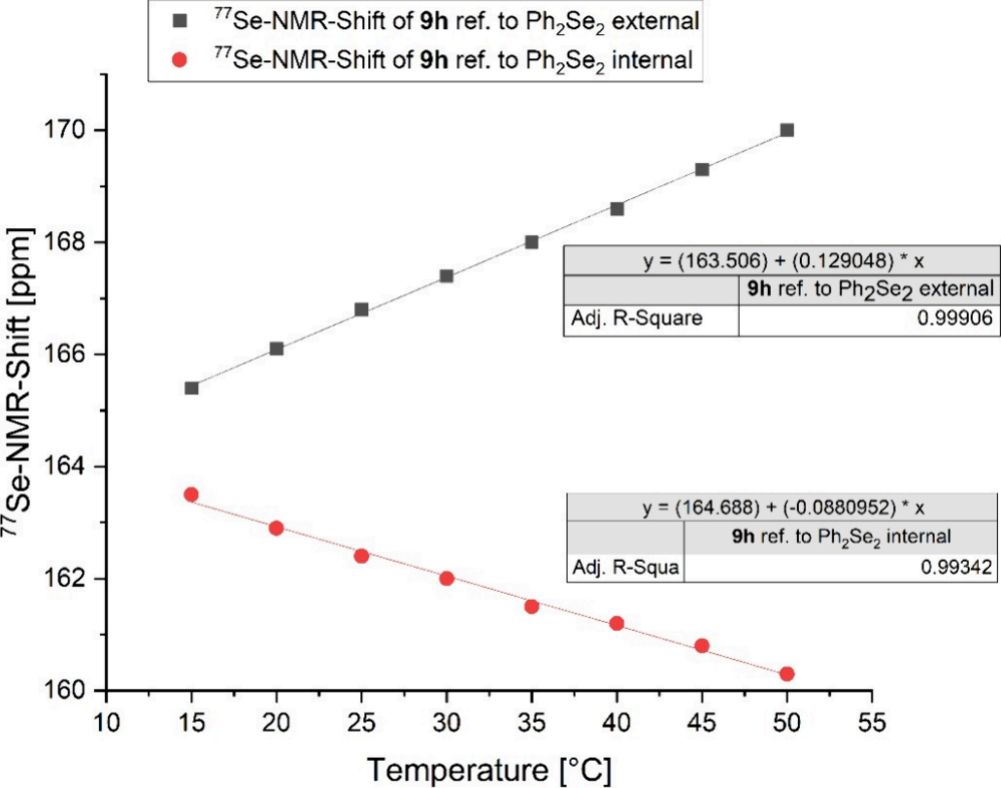
Temperature-dependent ^77^Se-NMR measurement of selenone **9h** in MeCN-*d*_3_ (reference PhSe-SePh as external reference (black) and internal reference (red).

Next, we investigated the often-cited temperature dependence of the ^77^Se NMR resonance frequency.^17,43,47,56,58,59^ For this purpose, two NMR spectra of **9h** in CD_3_CN were recorded in the range from 15 to 50 °C (Figure 9). Ph_2_Se_2_ was used as both internal and external reference; Me_2_Se was not used as internal reference due to the low vapor pressure of the substance. The samples externally referenced to Ph_2_Se_2_ at 461.0 ppm in CD_3_CN at 25 °C relative to Me_2_Se at 0.0 ppm show a linear increase in the ^77^Se NMR shift with increasing temperature, with a rate of change of +0.13 ppm/K with an *R*^2^ = 0.999.

When using Ph_2_Se_2_ as an internal standard, where the signal of the standard was set to 461 ppm independent of temperature, the ^77^Se shift of **9h** also shows a linear dependence, but it is negative at −0.09 ppm/K (*R*^2^ = 0.993), which can be explained by the temperature dependence of the standard. Ph_2_Se_2_ shows a linear positive ^77^Se NMR shift of +0.28 ppm/K (Supporting Information, Figure S201, p. S206) when it is externally referenced to Ph_2_Se_2_. In our analysis of the NMR spectra, we found that the ^77^Se NMR shift of Ph_2_Se_2_ as well as that of **9h** in the mixture is shifted downfield by 4 ppm compared to the signal of the pure standards at the same temperature. This indicates an interaction of the internal standard with the analyte. To confirm this assumption, we performed another NMR experiment in a coaxial NMR tube using a mixture of Ph_2_Se_2_ and **9h** in a 1:1 ratio in the outer chamber and only the standard in the inner tube. Two ^77^Se NMR resonance frequencies were detected for the Ph_2_Se_2_, which were offset from each other by 4 ppm, as previously observed (Supporting Information, Figure S202, p. S207). Based on these results, it can be assumed that an interaction with the analyte occurs when at least Ph_2_Se_2_ is used as the internal standard. Internally and externally referenced samples can therefore not be readily compared. Since we have already observed a considerable dependence of the ^77^Se resonance frequency on the solvent used, we were also interested in whether standard analyt interactions are also dependent on the solvent. We therefore measured a sample of selenoureas **9h** and **9e** in chloroform and referenced it once internally and once externally against Ph_2_Se_2_, as this is probably the most frequently used solvent for ^77^Se NMR measurements. The ^77^Se resonance frequencies of these measurements show no deviation, from which it can be concluded that the previously observed interactions are also solvent dependent.

**Figure 10:**
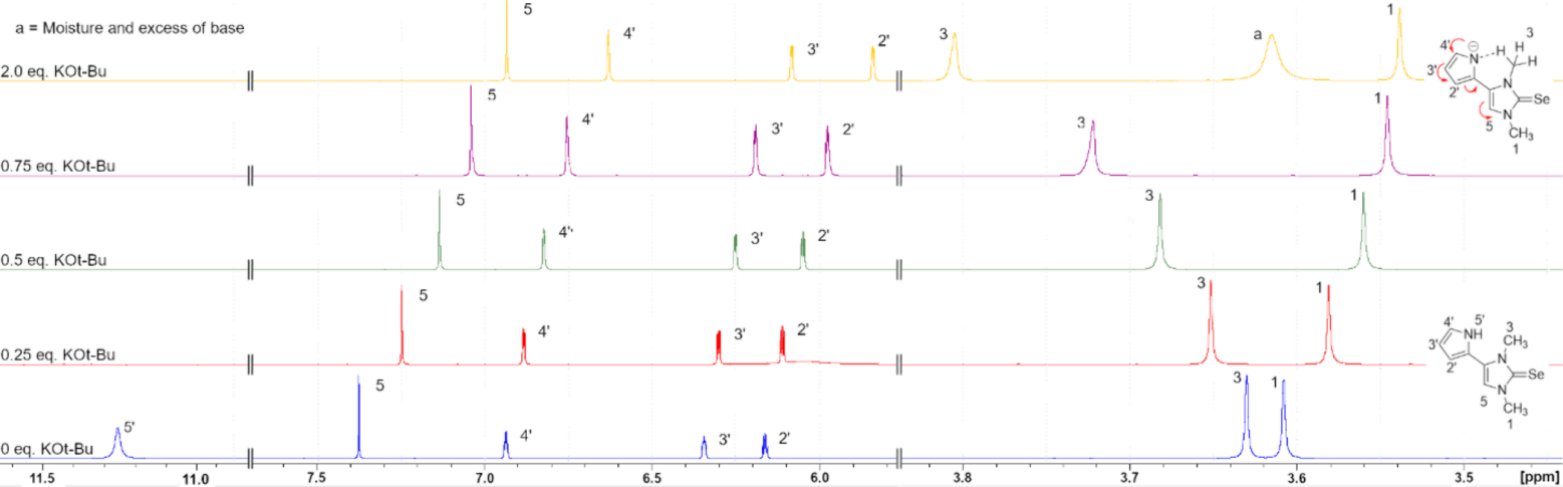
Selected ^1^H NMR shifts of the deprotonated selenone **9a** in DMSO-*d*_6_ (referenced to the solvent signal).

Finally, to study the influence of a negatively charged pyrrolide substituent on the NMR resonance frequencies, selenone **9a** was deprotonated in situ in an NMR tube to yield the anion **10a**. Thus, 0.25 to 2 equiv of potassium *tert*-butoxide were added in portions to a sample of **9a** in DMSO-*d*_6_ and the ^1^H NMR, ^13^C NMR and ^77^Se NMR shifts of the sample were measured. During deprotonation, it was observed that the ^1^H NMR signals of the pyrrole and imidazole ring undergo highfield shifts as expected (Figure 10) and a similar, albeit less pronounced, effect can also be seen in the ^13^C NMR spectrum. Position 4 of the imidazole displayed the strongest change by 0.45 ppm in the ^1^H NMR spectrum which is in accord with the delocalization of the negative charge within the conjugated anion **10a**. In analogy to the base screening experiments, no evidence of deprotonation of the C5 position was obtained when base was added to compound **9a**, indicating that no abnormal carbene is formed under the chosen conditions. Therefore, the signal of the methyl group at N1 also shifts to a higher field. As the methyl group at N3 forms a hydrogen bond to the nitrogen atom of the pyrrole ring, its signal shifts to lower field, as it is now fixed in the plane of the pyrrole ring and anisotropy effects occur. The ^77^Se NMR signal shifts by 20.3 ppm from 52.1 pmm to 31.8 ppm during this deprotonation. Results of the NMR titration are shown in Figure S181 (Supporting Information). For the same reason, protons of the phenyl ring of **10b** undergo a strong high-field shift. Similar ^77^Se NMR shifts are observed with **9e** and **9b**, whose values are shifted by 19 and 17 ppm into the high field. To the best of our knowledge, these are the first values that quantify the influence of negative charges of substituents conjugated to imidazole by ^77^Se NMR spectroscopy. A graphical and comparative compilation of the NMR shift differences during deprotonation can be found in the Supporting Material (Figures S180, S181, S188, S189, S196, and S197). After the addition of 2 eq. KO*t*-Bu, we were able to detect an additional signal in the ^1^H NMR spectrum with a resonance frequency of 3.6 ppm. It can be assumed that this is probably water that has entered the sample during the addition of the base, which was not dried beforehand, or from the environment. The measured spectra of compound **9a** show that this signal is not related to the analyte.

Comparing the ^77^Se NMR shifts of the selenium adducts described here with those of other N-heterocyclic imidazole compounds such as 1,3-diisopropyl-4,5-dimethylimidazole-2-selenone (δ^77^Se = −18 ppm),^21b^ 4,5-bis(1,3-diisopropylphenyl)imidazole-2-selenone (δ^77^Se = 87 ppm),^21b^ 1,3-diadamantylimidazole-2-selenone (δ^77^Se = 197 ppm)^21b^ and 1,3-bis(1,3,5-trimethylphenyl)-2-selenoxoimidazolidine-4,5-dione (δ^77^Se = 856 ppm),^18^ one can speak of moderate π-acceptor properties of our molecules on the basis of the previous opinion on the significance of the ^77^Se shifts, which is not surprising considering their π-electron density. This is consistent with the shift differences when the selenoureas are converted from the neutral to the anionic form. Based on our observations of the electronic structure of betaine and deprotonated selenourea, an application of the systems shown here in transition metal catalysis is conceivable. The betaines represent π-electron-rich systems which, depending on the choice of conditions, could also be used as anionic ligands.^60a,60b^ In addition, due to the synthesis used, the betaines could be easily modified and thus adapted to the requirements of catalysis. A further modification of the N-position of the pyrrole also seems possible.^60^

## Conclusions

In summary, using new mesoionic compounds with an imidazolium and a pyrrolide building block, a systematic investigation of factors for ^77^Se NMR shifts used in the literature to estimate the electronic properties of N-heterocyclic carbenes such as σ-donor and π-acceptor properties is presented here. These mesoionic compounds can be effectively prepared via a four-step synthesis and show promising CREF values of about 0.546 for estimating the formation tendency of anionic N-heterocyclic carbenes by deprotonation. These values obviously do not include N1-, N3- and C4-substituent influences because the values of the different substitution patterns of the model compounds are very similar. Rather, the π-character of the mesoionic basic system is dominant. The HOMOs of the anionic N-heterocyclic carbenes are π-orbitals originating in mesoionic compounds. As expected, systematic studies of the ^77^Se NMR resonance frequencies of the selenourea derivatives of the corresponding NHCs show dependencies on the substitution pattern, with the *tert.*-butyl derivative causing sudden downfield shifts to ca. 183 ppm, consistent with literature observations. We were able to systematically investigate the solvent dependence often cited in the literature on our model systems and found a parallelism of the solvent-induced shift differences and the respective substitution patterns, whereby the order of the solvents to visualize this parallelism does not follow any simple property relationships such as the solvent polarity, the donicity or the basicity of the solvents. However, the temperature dependence can be quantified very well. We were also able to show that the referencing method (Me_2_Se vs. PhSe-SePh) can, but does not necessarily have to, have a profound influence on the ^77^Se NMR shifts determined, depending on the NMR solvent. For example, measurements in CDCl_3_ react particularly sensitively to the type of referencing, whereas no influence could be detected for measurements in acetone-*d*_6_ for the molecules described here. The effects of the charges on the ^77^Se NMR shifts are described here for the first time. Negative charges delocalized in the π-system of heteroaromatics lead to highfield shifts of the ^77^Se NMR resonance frequencies between −20.2 ppm and −17.6 ppm, depending on the substitution pattern of the underlying ring system. These results will be useful for anyone using ^77^Se NMR values of selenium adducts to estimate the electronic properties of N-heterocyclic carbenes

## Experimental Section

### General Remarks

All commercially available chemicals were used without further purification unless otherwise mentioned. Anhydrous solvents were dried according to standard procedures (acetonitrile) or purchased from a MP5 Solvent Purification System of the company Inert Technology (diethyl ether, tetrahydrofuran). The reactions were traced by thin layer chromatography (TLC) performed on 60 F254 sillica-coated aluminum plates from Merck and visualized using UV light (254 nm). The preparative column chromatography was conducted through silica gel 60 (230–400 mesh) of the company Merck. For the ion-exchange column chromatography the exchange resin Amberlite IRA-400(Cl) from Alfa Aesar was used. The ^1^H and ^13^C NMR spectra were recorded with a Bruker Avance III 600 MHz spectrometer (at 600.35 MHz for ^1^H NMR, 150.96 MHz for ^13^C NMR), and a Bruker Avance Neo 400 MHz spectrometer (at 400.23 MHz for ^1^H NMR, 100.64 MHz for ^13^C NMR). ^77^Se spectra were measured with a Bruker Avance III 600 MHz spectrometer (at 114.50 MHz for ^77^Se) and referenced externally to dimethyl selenide or internally by addition of Me_2_Se, as indicated. NMR solvent peaks were used as the internal reference, all solvents were obtained from Deutero GmbH. Multiplicities are described by using the following abbreviations: s = singlet, d = doublet, t = triplet, q = quartet, and m = multiplet. Signal orientations in DEPT experiments were described as follows: o = no signal; + = up (CH, CH_3_); - = down (CH_2_). ^1^H NMR coupling constants are mentioned in Hz. The ATR-IR spectra were measured with a Bruker Alpha in the range of 400 to 4000 cm^–1^. The electrospray ionization mass spectra were measured with a Bruker Impact II mass spectrometer, spraying the samples from methanol. Melting points were determined in an apparatus according to Dr. Tottoli (Büchi) and are uncorrected. The compounds **2a**,^61^**2b**,^62^**2c**,^62^**4a**,^35a^**4b**,^63^**4c**,^63^ and **5a**–**c**^35a^ were prepared according to literature procedures.

#### General Procedure for the Synthesis of Imidazoles (Procedure E)

In a round-bottom flask, equipped with a magnetic stirrer, 1 eq. of imine (**2a** or **2b**) was dissolved in methanol. One eq of either Tos-MIC or a Tos-MIC-derivative (**5a**–**c**), 5 eq. of 2-methylpropane-2-amine and 10 mol % of Bi(OTf)_3_ were added. Then the solution was stirred for a duration of 12 h at room temperature. After the reaction, the resulting crude product was purified either by filtration or by column chromatography.

#### 1-Methyl-4-(1H-pyrrol-2-yl)-1H-imidazole (**6a**)

According to Procedure E, 2.00 g (18 mmol) of imine **2a** were dissolved in 20 mL of methanol. 3.61 g (18 mmol) of TosMIC, 10 mL (6.73 g, 92 mmol) of 2-methylpropan-2-amine, and 0.012 g (0.018 mmol) Bi(OTf)_3_ were added and the solution was stirred for 12 h. The solvent was then removed in vacuo and the resulting residues were purified by column chromatography, in which trichloromethane was used first to remove the imine, before the eluent was switched to ethyl acetate/methanol (9:1). 1-Methyl-5-(1H-pyrrol-2-yl)-1H-imidazole **6a** was isolated as a brown solid, 2.03 g, 75%. ^1^H NMR (400 MHz, CDCl_3_): 9.79 (bs, 1H), 7.44 (s, 1H), 7.06 (s, 1H), 6.89–6.87 (m, 1H), 6.30–6.28 (m, 2H), 3.68 (s, 3H). Spectroscopic data are in agreement with those reported in literature.^7^ Unfortunately, this is a mistake. Please cite ref. 34 instead of 7 here.

#### 1-(*tert*-Butyl)-4-(1H-pyrrol-2-yl)-1H-imidazole (**6b**)

According to Procedure E, 2.75 g (18 mmol) of imine **2b** were dissolved in 20 mL of methanol. 3.57 g (18 mmol) of TosMIC, 9.62 mL (6.69 g, 92 mmol) of 2-methylpropan-2-amine, and 0.012 g (0.018 mmol) Bi(OTf)_3_ were added and the solution was stirred for 12 h. The solvent was then removed in vacuo, the resulting residues were purified by column chromatography (trichloromethane/methanol = 9:1) to give 1-(*tert*-butyl)-5-(1H-pyrrol-2-yl)-1H-imidazole **6b**: 2.09 g, 60%, as a light brown solid. ^1^H NMR (400 MHz, CDCl_3_): 8.82 (bs, 1H), 7.62 (d, *J* = 0.8 Hz 1H), 6.97 (s, 1H), 6.88–6.86 (m, 1H), 6.30–6.28 (m, 1H),6.27–6.25 (m, 1H), 1.45 (s, 9H). Spectroscopic data are in agreement with those reported in literature.^7^ Unfortunately, this is a mistake. Please cite ref. 34 instead of 7 here.

#### 3-Methyl-5-phenyl-4-(1H-pyrrol-2-yl)-1H-imidazole (**6c**)

According to Procedure E, 1.12 g (10 mmol) of imine **2a** were dissolved in 20 mL of methanol. 2.81 g (10 mmol) of TosMIC-derivative **5a**, 5.00 mL (3.79 g, 52 mmol) of 2-methylpropan-2-amine, and 0.072 g (0.10 mmol) Bi(OTf)_3_ were added and the solution was stirred for 12 h during which the product started to participate. Then the mixture was cooled to 0 °C, the imidazole was collected by filtration, washed with cold methanol and dried in vacuo. The reaction yielded 3-methyl-5-phenyl-4-(1H-pyrrol-2-yl)-1H-imidazole **6c**, 1.75 g, 75%, as a colorless solid, mp 233–235 °C. ^1^H NMR (600 MHz, DMSO-*d*_6_): 11.10 (bs, 1H, H-5′), 7.77 (s, 1H, H-2), 7.46–7.45 (m, 2H, H-2”), 7.22–7.20 (m, 2H, H-3”), 7.13–7.10 (m, 1H, H-4”), 6.94–6.93 (m, 1H, H-4’), 6.22–6.21 (m, 1H, H-3′), 6.20–6.19 (m, 1H, H-2’), 3.44 (s, 3H, H-1”’) ppm. ^13^C{^1^H} NMR (150 MHz, DMSO-*d*_6_): 138.3 (o, C-1”), 137.9 (+, C-2), 135.3 (o, C-4 or C-5), 128.0 (+, C-3”), 125.9 (+, C-4”), 125.4 (+, C-2”), 121.8 (o, C-4 or C-5), 119.6 (+, C-4’), 118.7 (o, C-1’), 110.4 (+, C-2’), 108.7 (+, C-3′), 31.5 (+, C-1”’) ppm. IR (ATR): 3169, 3108, 2944, 1655, 1596, 1532, 1499, 1439, 1413, 1376, 1324, 1255, 1205, 1121, 1067, 1025, 967, 909, 881, 825, 773, 722, 646, 607, 542, 507, 451 cm^–1^. HRMS (ESI): *m*/*z* calcd. for [M + Na]^+^ 246.1001; found 246.1002.

#### 3-Methyl-4-(1H-pyrrol-2-yl)-5-(p-tolyl)-1H-imidazole (**6d**)

According to Procedure E, 0.65 g (6.0 mmol) of imine **2a** were dissolved in 20 mL of methanol. 1.72 g (6.0 mmol) of TosMIC-derivative **5b**, 3.17 mL (2.20 g, 30 mmol) of 2-methylpropan-2-amine, and 0.039 mg (0.060 mmol) Bi(OTf)_3_ were added and the solution was stirred for 12 h during which the product started to participate. Then the mixture was cooled to 0 °C and the imidazole was collected by filtration and washed with cold methanol. After drying under vacuum, 3-methyl-4-(1H-pyrrol-2-yl)-5-(p-tolyl)-1H-imidazole **6d** was obtained, 0.81 g, 56%, as a colorless solid, mp 215.5–217 °C. ^1^H NMR (600 MHz, DMSO-*d*_6_): 11.05 (bs, 1H, H-5′), 7.74 (s, 1H, H-2), 7.35 (d, *J* = 8.0 Hz, 2H, H-2”), 7.02 (d, *J* = 8.0 Hz, 2H, H-3”), 6.93–6.92 (m, 1H, H-4’), 6.21–6.20 (m, 1H, H-3′), 6.18–6.17 (m, 1H, H-2’), 3.43 (s, 3H, H-1”’), 2.23 (s, 3H, H-5”) ppm. ^13^C{^1^H} NMR (150 MHz, DMSO-*d*_6_): 138.9 (o, C-1”), 138.2 (+, C-2), 135.4 (o, C-4”), 132.8 (o, C-4 or C-5), 129.1 (+, C-3”), 125.9 (+, C-2”), 121.9 (o, C-4 or C-5), 120.0 (+, C-4’), 119.4 (o, C-1’), 110.8 (+, C-2’), 109.1 (+, C-3′), 32.0 (+, C-1”’), 21.2 (+, C-5”) ppm. IR (ATR): 3121, 2950, 2844, 1703, 1640, 1605, 1533, 1500, 1411, 1371, 1315, 1256, 1207, 1177, 1125, 1110, 1057, 1025, 968, 906, 877, 857, 827, 776, 730, 640, 604, 544, 514, 446 cm^–1^. HRMS (ESI): *m*/*z* calcd. for [M + Na]^+^ 260.1158; found 260.1158.

#### 5-(4-Methoxyphenyl)-3-methyl-4-(1H-pyrrol-2-yl)-1H-imidazole (**6e**)

According to Procedure E, 0.60 g (5.5 mmol) of imine **2a** were dissolved in 20 mL of methanol. 1.67 g (5.5 mmol) of TosMIC-derivative **5c**, 3.00 mL (2.03 g, 28 mmol) of 2-methylpropan-2-amine, and 0.036 mg (0.055 mmol) Bi(OTf)_3_ were added and the solution was stirred for 12 h during which the product started to participate. Then the mixture was cooled to 0 °C and the imidazole was collected by filtration, washed with cold methanol and dried in vacuo. The reaction yielded 5-(4-methoxyphenyl)-3-methyl-4-(1H-pyrrol-2-yl)-1H-imidazole **6e**, 1.19 g, 84%, as a colorless solid, mp 231.3–232.7 °C. ^1^H NMR (600 MHz, DMSO-*d*_6_): 11.05 (bs, 1H, H-5′), 7.72 (s, 1H, H-2), 7.38 (d, *J* = 9.0 Hz, 2H, H-3”), 6.93–6.92 (m, 1H, H-4’), 6.80 (d, *J* = 9.0 Hz, 2H, H-2”), 6.21–6.19 (m, 1H, H-3′), 6.18–6.17 (m, 1H, H-2’), 3.70 (s, 3H, H-5”), 3.43 (s, 3H, H-1””) ppm. ^13^C{^1^H} NMR (150 MHz, DMSO-*d*_6_): 158.2 (o, C-4”), 138.9 (o, C-1”), 138.1 (+, C-2), 128.2 (o, C-5), 127.1 (+, C-3”), 121.9 (o, C-4), 120.0 (+, C-4’), 119.4 (o, C-1’), 114.0 (+, C-2”), 110.8 (+, C-2’), 109.1 (+, C-3′), 55.5 (+, C-5”), 32.0 (+, C-1”’) ppm. IR (ATR): 3119, 2926, 2836, 1534, 1501, 1449, 1383, 1294, 1245, 1170, 1126, 1101, 1064, 1023, 967, 905, 874, 837, 722, 638, 601, 545, 523, 442 cm^–1^. HRMS (ESI): *m*/*z* calcd. for [M + Na]^+^ 276.1107; found 276.1107.

#### 1,3-Dimethyl-4-(1H-pyrrol-2-yl)-1H-imidazolium Tetrafluoroborate (**7a**) (Procedure F)

In an oven-dried round-bottom flask, equipped with a magnetic stirrer, 1.00 (6.8 mmol) of imidazole **6a** was dissolved in dry dichloromethane (DCM) under inert atmosphere. 1.5 g (10.0 mmol) of trimethyloxonium tetrafluoroborate were added to the solution and the mixture was stirred for the duration of 12 h. The product was collected by filtration and washed with DCM. The reaction yielded 1,3-dimethyl-4-(1H-pyrrol-2-yl)-1H-imidazolium tetrafluoroborate **7a**, 1.69 g, 98%, as a beige solid, mp 158–160.5 °C. ^1^H NMR (600 MHz, CD_3_CN): 10.81 (bs, 1H, H-5′), 8.73 (s, 1H, H-2), 7.58 (s, 1H, H-5), 7.02–7.00 (m, 1H, H-4’), 6.54–6.52 (m, 1H, H-3′ or H-2’), 6.30–6.28 (m, 1H, H-2’ or H-3′), 3.88 (s, 3H, H-1”), 3.86 (s, 3H, H-2”) ppm. ^13^C{^1^H} NMR (150 MHz, CD_3_CN): 136.9 (+, C-2), 129.6 (o, C-4), 121.8 (+, C-4’), 119.7 (+, C-5), 116.1 (o, C-1’), 111.2 (+, C-2’ or C-3′), 109.9 (+, C-2’ or C-3′) 36.5 (+, C-1”), 35.4 (+, C-2”) ppm. IR (ATR): 3406, 3135, 1622, 1586, 1532, 1451, 1351, 1171, 1018, 917, 876, 846, 782, 737, 655, 610, 573, 521 cm^–1^. HRMS (ESI): *m*/*z* calcd. for [M]^+^ 162.1026; found 162.1026.

#### General Procedure for the Synthesis of Methylated 4,5-Substituted Imidazolium Salts (Procedure G)

In a round-bottom flask, equipped with a magnetic stirrer and a reflux condenser 1 eq. of imidazole (**5c** - **5e**) was dissolved in 5 mL of acetonitrile. Then, 6 eq. of iodomethane were added to the solution dropwise, before the mixture was heated in an oil bath at reflux temperature for 5 h. The solvent was then removed in vacuo and the residues were purified by column chromatography (dichloromethane/methanol).

#### 1,3-Dimethyl-5-phenyl-4-(1H-pyrrol-2-yl)-1H-imidazolium Iodide (**7b**)

According to Procedure G, 0.4 g (1.79 mmol) of imidazole **6c** were dissolved in acetonitrile, then 0.67 mL (1.53 g, 10.70 mmol) of iodomethane were added. After the reaction, the product was purified by column chromatography (dichloromethane/methanol= 5:1), to yield 1,3-dimethyl-5-phenyl-4-(1H-pyrrol-2-yl)-1H-imidazolium iodide **7b**, 0.53 g, 80%, as an orange solid, mp 154.5–156 °C. ^1^H NMR (600 MHz, DMSO-*d*_6_): 11.32 (s, 1H, H-5′), 9.32 (s, 1H, H-2), 7.51–7.48 (m, 3H, H-3″, H-4”), 7.46–7.44 (m, 2H, H-2”), 6.98–6.97 (m, 1H, H-4’), 6.27–6.26 (m, 1H, H-3′), 6.14–6.13 (m, 1H, H-2’), 3.75 (s, 3H, H-1”’), 3.74 (s, 3H, H-2”’) ppm. ^13^C{^1^H} NMR (150 MHz, DMSO-*d*_6_): 137.3 (+, C-2), 132.4 (o, C-1”), 130.9 (+, C2” or C-3”), 130.5 (+, C-4”), 129.3 (+, C-2” or C-3”), 125.9 (o, C-5), 125.7 (o, C-4), 122.0 (+, C-4’), 114.3 (o, C-1’), 113.5 (+, C-3′), 109.4 (o, C-2’), 35.2 (+, C-2”’), 34.8 (C-1”’) ppm. IR (ATR): 3414, 3168, 3078, 2760, 1620, 1517, 1440, 1408, 1371, 1337, 1192, 1105, 1016, 920, 814, 750, 706, 628, 605, 572 cm^–1^. HRMS (ESI): *m*/*z* calcd. for [M]^+^ 238.1334; found 238.1341.

#### 1,3-Dimethyl-4-(1H-pyrrol-2-yl)-5-(p-tolyl)-1H-imidazolium Iodide (**7c**)

According to Procedure G, 0.75 g (3.16 mmol) of imidazole **6d** were dissolved in acetonitrile, then 1.18 mL (2.69 g, 19.00 mmol) of iodomethane were added. After the reaction the product was purified by column chromatography (dichloromethane/methanol= 5:1) to yield 1,3-dimethyl-4-(1H-pyrrol-2-yl)-5-(p-tolyl)-1H-imidazolium iodide **7c**, 0.65 g, 81%, as a yellow solid, mp 152.8–154 °C. ^1^H NMR (600 MHz, DMSO-*d*_6_): 11.26 (s, 1H, H-5′), 9.33 (s, 1H, H-2), 7.35 (d, *J* = 8.2 Hz, 2H, H-2”), 7.31 (d, *J* = 8.2 Hz, 2H, H-3”), 6.99–6.98 (m, 1H, H-4’), 6.27 (bs, 1H, H-3′), 6.15–6.14 (m, 1H, H-2’), 3.76 (s, 3H, H-1”’), 3.75 (s, 3H, H-2”’), 2.36 (s, 3H, H-5”) ppm. ^13^C{^1^H} NMR (150 MHz, DMSO-*d*_6_): 140.3 (o, C-4’), 137.2 (+, C-2), 132.5 (o, C-5), 130.8 (+, C-3”), 129.9 (+, C-2”), 125.8 (o, C-4), 122.8 (o, C-1”), 122.0 (+, C-4’), 114.4 (o, C-1’), 113.4 (+, C-3′), 109.3 (o, C-2’), 35.1 (+, C-2”’), 34.8 (C-1”’), 21.4 (+, C-5”) ppm. IR (ATR): 3419, 3168, 3083, 3021, 1622, 1568, 1513, 1480, 1450, 1404, 1337, 1194, 1115, 916, 843, 815, 755, 720, 502 cm^–1^. HRMS (ESI): *m*/*z* calcd. for [M]^+^ 252.1495; found 252.1495.

#### 5-(4-Methoxyphenyl)-1,3-dimethyl-4-(1H-pyrrol-2-yl)-1H-imidazolium Iodide (**7d**)

According to procedure G, 0.75 g (2.96 mmol) of imidazole **6e** were dissolved in acetonitrile, then 1.11 mL (2.52 g, 17.77 mmol) of iodomethane were added. After the reaction, the product was purified by column chromatography (dichloromethane/methanol= 5:1) to yield 5-(4-methoxyphenyl)-1,3-dimethyl-4-(1H-pyrrol-2-yl)-1H-imidazolium iodide **7d**, 0.69 g, 86%, as a yellow solid, mp 198.2–200 °C. ^1^H NMR (600 MHz, DMSO-*d*_6_): 11.17 (bs, 1H, H-5′), 9.26 (s, 1H, H-2), 7.35 (d, *J* = 8.7 Hz, 2H, H-2”), 7.01 (d, *J* = 8.7 Hz, 2H, H-3”), 6.95–6.94 (m, 1H, H-4’), 6.24–6.23 (m, 1H, H-3′), 6.11–6.10 (m, 1H, H-2’), 3.76 (s, 3H, H-5”), 3.71 (s, 3H, H-1”’), 3.69 (s, 3H, H-2”’) ppm. ^13^C{^1^H} NMR (150 MHz, DMSO-*d*_6_): 160.9 (o, C-4’), 137.0 (+, C-2), 132.4 (+, C-2”), 125.6 (o, C-5 or C-4), 122.0 (+, C-4’), 117.6 (o, C-4 or C-5), 114.8 (+, C-3”), 114.5 (o, C-1”), 114.4 (o, C-1’), 113.4 (+, C-3′), 109.3 (o, C-2’), 55.8 (+, C-5”), 35.0 (+, C-2”’), 34.8 (C-1”’) ppm. IR (ATR): 3427, 3153, 3049, 2961, 2836, 1627, 1606, 1568, 1511, 1442, 1414, 1291, 1247, 1179, 1112, 1017, 919, 847, 820, 749, 617, 575, 521 cm^–1^. HRMS (ESI): *m*/*z* calcd. for [M]^+^ 268.1444; found 268.1444.

#### General Procedure for the Synthesis of *N*-Benzylated Imidazolium Salts (Procedure H)

In an oven-dried round-bottom flask, equipped with a magnetic stirrer and a reflux condenser, 1 eq. of imidazole (**5a** or **5b**) was dissolved in dry THF under inter atmosphere. 1.2 eq. of the benzyl bromide derivative were added to the solution. The mixture was then heated 85 °C in an oil bath for 12 h. The product was collected by filtration, washed with cold THF, and dried in vacuo.

#### 3-Benzyl-1-methyl-4-(1H-pyrrol-2-yl)-1H-imidazolium Bromide (**7e**)

According to procedure H, 0.25 g (1.7 mmol) of imidazole **6a** were dissolved in dry THF, and 0.24 mL (0.35 g 2.0 mmol) of (bromomethyl)benzene were added. The mixture was heated under the conditions described above. After purification, the reaction yielded 1-benzyl-3-methyl-4-(1H-pyrrol-2-yl)-1H-imidazolium bromide **7e**, 0.41 g, 76%, as a gray solid, mp 207.4–209 °C. ^1^H NMR (600 MHz, DMSO-*d*_6_): 11.59 (bs, 1H, H-5′), 9.37–9.33 (m, 1H, H-2), 7.85–7.82 (m, 1H, H-5), 7.49–7–48 (m, 2H, H-3” or H-4”), 7.46–7.44 (m, 2H, H-3” or H-4”), 7.42–7.40 (m, 1H, H-5”), 7.04–7.03 (m, 1H, H-4’), 6.57–6.55 (m, 1H, H-3′), 6.24–6.23 (m, 1H, H-2’), 5.48 (s, 2H, H-1’), 3.90 (s, 3H, H-1”’) ppm. ^13^C{^1^H} NMR (150 MHz, DMSO-*d*_6_): 137.0 (+, C-2), 135.1 (o, C-2”), 129.5 (+, C-3” or C-4”), 129.4 (o, C-4), 129.3 (+, C-5”), 129.0 (+, C-3” or C-4”), 121.9 (+, C-4’), 117.8 (+, C-5), 116.2 (o, C-1’), 110.8 (+, C-3′), 109.8 (o, C-2’), 52.5 (-, C-1”), 35.6 (+, C-1”’) ppm. IR (ATR): 3410, 3108, 3051, 2970, 2862, 2569, 1728, 1663, 1623, 1575, 1533, 1452, 1401, 1344, 1259, 1204, 1164, 1123, 1079, 1039, 914, 871, 796, 731, 697, 669, 601, 524, 468 cm^–1^. HRMS (ESI): *m*/*z* calcd. for [M]^+^ 238.1339; found 238.1339.

#### 1-Methyl-3-(4-methylbenzyl)-4-(1H-pyrrol-2-yl)-1H-imidazolium Bromide (**7d**)

According to procedure H, 0.50 g (3.4 mmol) of imidazole **6a** were dissolved in dry THF, and 0.69 g (3.7 mmol) of 1-(bromomethyl)-4-methylbenzene were added. The mixture was heated under the conditions described above. After purification the reaction yielded 3-methyl-1-(4-methylbenzyl)-4-(1H-pyrrol-2-yl)-1H-imidazolium bromide **7d**, 0.88 g, 78%, as a beige solid, mp 260 °C (decomp.). ^1^H NMR (600 MHz, DMSO-*d*_6_): 11.58 (bs, 1H, H-5′), 9.31 (d, *J* = 1.8 Hz, 1H, H-2), 7.81 (d, *J* = 1.8 Hz, 1H, H-5), 7.38 (d, *J* = 8.1 Hz, 2H, H-3”), 7.26 (d, *J* = 8.1 Hz, 2H, H-4”), 7.04–7.03 (m, 1H, H-4’), 6.57–6.55 (m, 1H, H-3′), 6.24–6.23 (m, 1H, H-2’), 5.41 (s, 2H, H-1’), 3.89 (s, 3H, H-1”’), 2.32 (s, 3H, H-6”) ppm. ^13^C{^1^H} NMR (150 MHz, DMSO-*d*_6_): 138.8 (o, C-5”), 136.9 (+, C-2), 132.1 (o, C-2”), 130.0 (o, C-4), 129.3 (+, C-4”), 129.0 (+, C-3”), 121.8 (+, C-4’), 117.7 (+, C-5), 116.2 (o, C-1’), 110.8 (+, C-3′), 109.8 (+, C-2’), 52.3 (-, C-1”), 35.7 (+, C-1”’), 21.2 (+, C-6”) ppm. IR (ATR): 3366, 3138, 3053, 2969, 2875, 2359, 2303, 1619, 1572, 1543, 1515, 1450, 1408, 1337, 1249, 1206, 1153, 1126, 1045, 814, 755, 728, 661, 597, 565, 509, 465 cm^–1^. HRMS (ESI): *m*/*z* calcd. for [M]^+^ 252.1495; found 252.1504.

#### 1-(4-Methoxybenzyl)-3-methyl-4-(1H-pyrrol-2-yl)-1H-imidazolium Bromide (**7g**)

According to procedure H, 0.40 g (2.7 mmol) of imidazole **6a** were dissolved in dry THF, and 0.47 mL (0.66 g, 3.2 mmol) of 1-(bromomethyl)-4-methoxybenzene were added. The mixture was heated under the conditions described above. After purification the reaction yielded 1-(4-methoxybenzyl)-3-methyl-4-(1H-pyrrol-2-yl)-1H-imidazolium bromide **7g**, 0.75 g, 79%, as a colorless solid, mp 181.4–183 °C. ^1^H NMR (600 MHz, DMSO-*d*_6_): 11.61 (bs, 1H, H-5′), 9.34 (d, *J* = 1.8 Hz, 1H, H-2), 7.83 (d, *J* = 1.8 Hz, 1H, H-5), 7.46 (d, *J* = 8.7 Hz, 2H, H-3”), 7.03–7.02 (m, 1H, H-4’), 6.70 (d, *J* = 8.7 Hz, 3-H, H-4”), 6.55–6.54 (m, 1H, H-3′), 6.23–6.21 (m, 1H, H-2’), 5.39 (s, 2H, H-1’), 3.89 (s, 3H, H-1”’), 3.76 (s, 3H, H-6”) ppm. ^13^C{^1^H} NMR (150 MHz, DMSO-*d*_6_): 160.1 (o, C-5”), 136.7 (+, C-2), 130.8 (+, C-3”), 129.3 (o, C-4), 127.1 (o, C-2”), 121.6 (+, C-3”), 117.4 (+, C-5), 116.3 (o, C-1’), 114.8 (+, C-4”), 110.6 (+, C-3′), 109.6 (+, C-2’), 55.5 (+, C-6”), 51.8 (-, C-1”), 35.4 (+, C-1”’) ppm. IR (ATR): 3109, 3053, 3001, 2970, 2865, 2828, 1614, 1574, 1538, 1510, 1456, 1426, 1344, 1295, 1234, 1166, 1124, 1032, 874, 804, 765, 728, 670, 602, 552, 503 cm^–1^. HRMS (ESI): *m*/*z* calcd. for [M]^+^ HRMS (ESI): *m*/*z* calcd. for [M]^+^ 268.1444; found 268.1447.

#### 1-Benzyl-3-(*tert*-butyl)-4-(1H-pyrrol-2-yl)-1H-imidazolium Bromide (**7h**)

According to procedure H, 0.25 g (1.3 mmol) of imidazole **6b** were dissolved in dry THF, and 0.18 mL (0.27 g 1.6 mmol) of (bromomethyl)benzene were added. The mixture was heated under the conditions described above. After purification, the reaction yielded 1-benzyl-3-(*tert*-butyl)-4-(1H-pyrrol-2-yl)-1H-imidazolium bromide **7h**, 0.34 g, 72%, as a brown solid, mp 181.5–183 °C. ^1^H NMR (600 MHz, DMSO-*d*_6_): 11.42 (bs, 1H, H-5′), 9.67–9.57 (m, 1H, H-2), 7.99–7.97 (m, 1H, H-5), 7.57–7–52 (m, 2H, H-4”), 7.46–7.43 (m, 2H, H-3”), 7.41–7.39 (m, 1H, H-5”), 6.98 (s, 1H, H-4’), 6.35 (s, 1H, H-3′), 6.18 (s, 1H, H-2’), 5.46–5.42 (m, 2H, H-1’), 1.47 (s, 9H, H-2”’) ppm. ^13^C{^1^H} NMR (150 MHz, DMSO-*d*_6_): 136.2 (+, C-2), 135.3 (o, C-2”), 129.5 (+, C-3”), 129.3 (+, C-4”), 129.0 (+, C-5”), 127.5 (o, C-4), 125.1 (+, C-5), 120.9 (+, C-4’), 115.0 (o, C-1’), 114.3 (+, C-3′), 109.0 (o, C-2’), 62.4 (o, C-1”’), 52.4 (-, C-1”), 29.9 (+, C-2”’) ppm. IR (ATR): 3169, 3070, 2976, 2870, 1729, 1625, 1531, 1501, 1448, 1409, 1372, 1343, 1314, 1257, 1201, 1132, 1032, 918, 883, 847, 817, 738, 701, 652, 603, 529, 474 cm^–1^. HRMS (ESI): *m*/*z* calcd. for [M]^+^ 280.1808; found 280.1810.

#### 3-(*tert*-Butyl)-1-(4-methylbenzyl)-4-(1H-pyrrol-2-yl)-1H-imidazolium Bromide (**7i**)

According to procedure H, 0.30 g (1.6 mmol) of imidazole **6b** were dissolved in dry THF, and 0.32 g (1.9 mmol) of 1-(bromomethyl)-4-methylbenzene were added. The mixture was heated under the conditions described above. After purification the reaction yielded 3-(*tert*-butyl)-1-(4-methylbenzyl)-4-(1H-pyrrol-2-yl)-1H-imidazolium bromide **7i**, 0.59 g, 88%, as a beige solid, mp 193.4–195.5 °C. ^1^H NMR (600 MHz, DMSO-*d*_6_): 11.42 (bs, 1H, H-5′), 9.70 (d, *J* = 1.7 Hz, 1H, H-2), 7.96 (d, *J* = 1.7 Hz, 1H, H-5), 7.47 (d, *J* = 8.1 Hz, 2H, H-3”), 7.23 (d, *J* = 8.1 Hz, 2H, H-4”), 6.98–6.97 (m, 1H, H-4’), 6.34–6.33 (m, 1H, H-3′), 6.18–6.17 (m, 1H, H-2’), 5.41 (s, 2H, H-1’), 2.30 (s, 3H, H-6”), 1.46 (s, 3H, H-2”’) ppm. ^13^C{^1^H} NMR (150 MHz, DMSO-*d*_6_): 138.8 (o, C-5”), 136.1 (+, C-2), 132.3 (o, C-2”), 130.0 (+, C-3”), 129.2 (+, C-4”), 127.4 (o, C-4), 125.4 (+, C-5), 120.8 (+, C-4’), 115.1 (o, C-1’), 114.3 (+, C-3′), 108.9 (+, C-2’), 62.4 (o, C-1”’), 52.2 (-, C-1”), 29.9 (+, C-2”’), 21.2 (+, C-6”) ppm. IR (ATR): 3077, 3025, 2933, 2826, 1556, 1525, 1444, 1361, 1314, 1196, 1132, 1096, 1021, 861, 829, 801, 763, 724, 637, 610, 476 cm^–1^. HRMS (ESI): *m*/*z* calcd. for [M]^+^ 294.1965; found 294.1978.

#### General Procedure for the Synthesis of the Mesoionic Compounds

The Imidazolium salts **7a**–**i** were dissolved in a mixture of distilled water and methanol (1:1) and added dropwise to an ion-exchange-column packed with Amberlite IR 400 in its hydroxide form. The product was collected and unless otherwise noted, the solvent mixture was distilled off in vacuo. Finally, the product was dried at a temperature of 40 °C.

#### 2-(1,3-Dimethyl-1H-imidazol-3-ium-4-yl)pyrrol-1-ide (**8a**)

0.500 g (2.01 mmol) of salt **8a** were used, which yielded 2-(1,3-dimethyl-1H-imidazolium-4-yl)pyrrol-1-ide, 0.314 g, 97%, as a red, highly viscous oil. ^1^H NMR (600 MHz, DMSO-*d*_6_): 9.19 (s, 1H, H-2), 7.91 (s, 1H, H-5), 7.02–7.01 (m, 1H, H-4’), 6.53–6.52 (m, 1H, H-3′), 6.22–6.21 (m, 1H, H-2’), 3.89 (s, 3H, H-2”), 3.86 (s, 3H, H-1”) ppm. ^13^C{^1^H} NMR (150 MHz, DMSO-*d*_6_): 137.4 (+, C-2), 129.1 (o, C-4), 121.9 (+, C-4’), 119.3 (+, C-5), 116.4 (o, C-1’), 110.5 (+, C-3′), 109.6 (+, C-2’), 36.3 (+, C-1”), 35.4 (+, C-2”) ppm. IR (ATR): 3132, 3062, 2961, 1576, 1450, 1347, 1163, 1044, 829, 738, 684, 608 cm^–1^. HRMS (ESI): *m*/*z* calcd. for [M + H]^+^ 162.1026 found 268.1037, HRMS (ESI): *m*/*z* calcd. for [M-H]^+^ 160.0880 found 160.0871.

#### 1,3-Dimethyl-5-phenyl-4-(1H-pyrrol-2-yl)-1H-imidazolium (**8b**)

0.250 g (1.05 mmol) of salt **7b** were used, after the solvent mixture was evaporated the residue was washed with cold chloroform which gave 1,3-dimethyl-5-phenyl-4-(1H-pyrrol-2-yl)-1H-imidazolium **8b**, 0.193 g, 77%, as a beige solid, mp 126.5–128.3 °C. ^1^H NMR (600 MHz, CD_3_OD): 7.51–7.47 (m, 3H, H-2” or H-3″, H-4”), 7.42–7.41 (m, 2H, H-2” or H-3”), 6.91 (dd, *J* = 2.7, 1.4 Hz, 1H, H-4’), 6.32 (dd, *J* = 3.5, 1.4 Hz, 1H, H-3′), 6.20 (dd, *J* = 3.5, 2.7 Hz, 1H, H-2’), 3.82 (s, 3H, H-2”’), 3.80 (s, 3H, H-1”’) ppm. ^2^H NMR (92 MHz, CD_3_OD): 8.58 (s, 1H, H-2) ppm. ^13^C{^1^H} NMR (150 MHz, CD_3_OD): 137.7 (C-2), 134.2 (o, C-5), 131.6 (+, C-2” or C-3”), 131.6 (+, C-4”), 130.1 (+, C-2” or C-3”), 127.8 (o, C-4 or C-1”), 126.7 (C-4 or C-1”), 122.6 (+, C-4’), 115.0 (o, C-1’), 114.4 (+, C-3′), 110.2 (+, C-2’), 34.8 (+, C-2”’), 35.2 (+, C-1”’) ppm. ATR (IR): 3366, 3151, 3022, 2949, 2786, 1576, 1449, 1371, 1268, 1212, 1136, 1109, 870, 846, 814, 778, 724, 614, 499 cm^–1^. HRMS (ESI): *m*/*z* calcd. for [M + H]^+^ 238.1339; found 238.1337.

#### 2-(1,3-Dimethyl-5-(p-tolyl)-1H-imidazolium-4-yl)pyrrol-1-ide (**8c**)

0.250 g (0.99 mmol) of salt **7c** were used, after the solvent mixture was evaporated the residue was washed with cold chloroform which gave 2-(1,3-Dimethyl-5-(p-tolyl)-1H-imidazol-3-ium-4-yl)pyrrol-1-ide **8c**, 0.185 g, 74%, as a beige solid, mp 103.8–105.2 °C. ^1^H NMR (600 MHz, CD_3_OD): 7.29 (m, 4H, H-2” or H-3”), 6.90 (dd, *J* = 2.8, 1.5 Hz, 1H, H-4’), 6.31 (dd, *J* = 3.7, 1.5 Hz, 1H, H-3′), 6.19 (dd, *J* = 3.7, 2.8 Hz, 1H, H-2’), 3.81 (s, 3H, H-2”’), 3.78 (s, 3H, H-1”’), 2.38 (s, 3H, H-5”) ppm. ^2^H NMR (92 MHz, CD_3_OD): 8.56 (s, 1H, H-2) ppm. ^13^C{^1^H} NMR (150 MHz, CD_3_OD): 141.9 (o, C-4”), 137.5 (C-2), 134.4 (o, C-1”), 131.5 (+, C-2”), 130.7 (+, C-3”), 127.6 (o, C-4), 123.6 (C-5), 122.5 (+, C-4’), 115.1 (o, C-1’), 114.3 (+, C-3′), 110.2 (+, C-2’), 35.1 (+, C-1”’), 34.8 (+, C-2”’), 21.4 (+, C-5”) ppm. ATR (IR): 3356, 3018, 2949, 2786, 1576, 1515, 1449, 1371, 1268, 1211, 1136, 1109, 1026, 907, 870, 846, 814, 780, 724, 613, 499 cm^–1^. HRMS (ESI): *m*/*z* calcd. for [M + H]^+^ 251.9034; found 252.1495.

#### 2-(5-(4-Methoxyphenyl)-1,3-dimethyl-1H-imidazolium-4-yl)pyrrol-1-ide (**8d**)

0.250 g (0.93 mmol) of salt **7d** were used, which yielded 2-(5-(4-methoxyphenyl)-1,3-dimethyl-1H-imidazolium-4-yl)pyrrol-1-ide **8d**, 0.204 g, 82%, as a pink solid, m.p.130.4–132.2 °C. ^1^H NMR (600 MHz, CD_3_OD): 7.34 (d, *J* = 8.8 Hz, 2H, H-2’), 7.00 (dd, *J* = 8.8 Hz, 2H, H-3”), 6.91–6.90 (m, 1H, H-4’), 6.31 (dd, *J* = 1.2, 3.4 Hz, 1H, H-3′), 6.19–6.18 (m, 1H, H-2’), 3.81 (s, 3H, H-5”), 3.80 (3H, H- 2”’), 3.77 (3H, H-1”’) ppm. ^2^H NMR (92 MHz, CD_3_OD): 9.05 (s, 1H, H-2) ppm. ^13^C{^1^H} NMR (150 MHz, CD_3_OD):162.5 (o, C-4”), 133.1 (+, C-2”), 134.2 (o, C-5), 127.3 (o, C-4), 122.4 (+, C-4’), 118.4 (o, C-1”), 115.5 (+, C-3”), 115.2 (o, C-1’), 114.2 (+, C-3′), 110.1 (+, C-2’), 55.9 (+, C-5”), 35.0 (C-1”’), 34.8 (C-2”’) ppm). ATR (IR): 3403, 3148, 3033, 2840, 1643, 1608, 1569, 1512, 1440, 1411, 1381, 1339, 1291, 1250, 1183, 1112, 1012, 836, 746, 612, 571, 517, 446 cm^–1^. HRMS (ESI): *m*/*z* calcd. for [M-H]^+^ 268.1445 found 268.1447.

#### 2-(1-Benzyl-3-methyl-1H-imidazolium-4-yl)pyrrol-1-ide (**8e**)

0.350 g (0.94 mmol) of salt **7e** were used, which yielded 2-(1-benzyl-3-methyl-1H-imidazolium-4-yl)pyrrol-1-ide **8e**, 0.238 g, 91%, as an orange solid, mp 97.5–99 °C. ^1^H NMR (600 MHz, CD_3_OD): 7.61 (s, 1H, H-5), 7.50–7.44 (m, 5H, H-3″, H-4″, H-5”), 7.00 (dd, *J* = 1.3, 2.8 Hz, 1H, H-4’), 6.57 (dd, *J* = 1.3, 3.8 Hz, 1H, H-3′), 6.30 (dd, *J* = 2.8, 3.8 Hz, 1H, H-2’), 5.44 (s, 2H, H-1”), 3.94 (s, 3H, H-1”’) ppm. H-2 not observed. ^13^C{^1^H} NMR (150 MHz, CD_3_OD): 137.3 (C-2), 135.2 (o, C-2”), 131.5 (o, C-4), 130.5 (+, C-3” or C-4”), 130.4 (+, C-5”), 129.8 (+, C-3” or C-4”), 122.7 (+, C-4’), 118.8 (+, C-5), 116.5 (o, C-1’), 112.2 (+, C-3′), 110.7 (+, C-2’), 54.2 (-, C-1”), 35.5 (+, C-1”’) ppm. IR (ATR):3103, 3035, 2960, 1618, 1576, 1450, 1379, 1344, 1205, 1133, 873, 842, 813, 780, 722, 644, 605, 500, 469 cm^–1^. HRMS (ESI): *m*/*z* calcd. for [M + H]^+^ 238.1339 found 238.1353, HRMS (ESI): *m*/*z* calcd. for [M-H]^+^ 236.1193; found 236.1183.

#### 2-(3-Methyl-1-(4-methylbenzyl)-1H-imidazolium-4-yl)pyrrol-1-ide (**8f**)

0.250 g (0.94 mmol) of salt **7f** were used, which yielded 2-(3-methyl-1-(4-methylbenzyl)-1H-imidazolium-4-yl)pyrrol-1-ide **8f**, 0.178 g, 94%, as a yellow solid, mp 108.2–110.6 °C. ^1^H NMR (600 MHz, DMSO-*d*_6_): 8.92 (bs, 1H, H-2), 7.41 (s, 1H, H-5), 7.32 (d, *J* = 7.8 Hz, 2H, H-3”), 7.21 (d, *J* = 7.8 Hz, 2H, H-4”), 6.79–6.78 (m, 1H, H-4’), 6.35 (dd, *J* = 1.2, 3.3 Hz, 1H, H-3′), 6.00 (dd, *J* = 1.2, 3.3, 1H, H-2’), 5.25 (s, 2H, H-1”), 3.95 (s, 3H, H-1”’), 2.30 (s, 3H, H-6”) ppm. ^13^C{^1^H} NMR (150 MHz, DMSO-*d*_6_): 138.1 (o, C-5”), 134.7 (+, C-2), 134.0 (o C-1’), 132.1 (o, C-2”), 129.5 (+, C-4”), 128.4 (+, C-3”), 127.9 (o, C-4), 121.9 (+, C-4’), 113.2 (+, C-5), 108.1 (+, C-3′), 107.9 (+, C-2’), 51.5 (-, C-1”), 35.4 (+, C-1”’), 20.7 (+, C-6”) ppm. IR (ATR): 3128, 3044, 2963, 1621, 1569, 1448, 1344, 1144, 1041, 813, 726, 657, 605, 507, 470 cm^–1^. HRMS (ESI): *m*/*z* calcd. for [M + H]^+^ 252.1495; found 252.1500, HRMS (ESI): *m*/*z* calcd. for [M-H]^+^ 250.1349 found 250.1347.

#### 2-(1-(4-Methoxybenzyl)-3-methyl-1H-imidazolium-4-yl)pyrrol-1-ide (**8g**)

0.350 g (1.00 mmol) of salt **7g** were used, which yielded 2-(1-(4-methoxybenzyl)-3-methyl-1H-imidazolium-4-yl)pyrrol-1-ide **8g**, 0.248 g, 92%, as a brown solid, mp 126.7–128.3 °C. ^1^H NMR (600 MHz, CD_3_OD): 7.43 (d, *J* = 8.8 Hz, 2H, H-3”), 6.98–6.97 (m, 3H, H-4′, H-4”), 6.51 (dd, *J* = 1.4, 3.6 Hz, 1H, H-3′), 6.25 (dd, *J* = 2.7, 3.6, 1H, H-2’), 5.32 (s, 2H, H-1’), 3.88 (s, 3H, H-1”’), 3.78 (s, 3H, H-6”) ppm. ^2^H NMR (92 MHz, CD_3_OD): 7.61 (s, 1H, H-5) ppm. H-2 not observed. ^13^C{^1^H} NMR (150 MHz, CD_3_OD): 161.8 (o, C-5”), 136.9 (C-2), 131.5 (+, C-3”),131.3 (o, C-4), 127.0 (o, C-1’), 122.8 (+, C-4’), 118.4 (+, C-5), 116.8 (o, C-2”), 115.7 (+, C-4”), 111.8 (+, C-3′), 110.5 (+, C-2’), 55.9 (+, C-6”), 53.7 (-. C-1”), 35.6 (+, C-1”’) ppm. IR (ATR): 3131, 3031, 2957, 2691, 1612, 1562, 1534, 1513, 1450, 1384, 1245, 1181, 1146, 1120, 1024, 831, 738, 694, 641, 610, 553, 516 cm^–1^. HRMS (ESI): *m*/*z* calcd. for [M + H]^+^ 268.1444; found 268.1453.

#### 2-(1-Benzyl-3-(*tert*-butyl)-1H-imidazolium-4-yl)pyrrol-1-ide (**8h**)

0.400 g (1.11 mmol) of salt **7h** were used, which yielded 2-(1-benzyl-3-(*tert*-butyl)-1H-imidazolium-4-yl)pyrrol-1-ide **8h**, 0.289 g, 93%, as an orange solid, mp 135–136.6 °C. ^1^H NMR (600 MHz, DMSO-*d*_6_): 9.53 (bs, 1H, H-2), 7.62 (s, 1H, H-5), 7.52–7.50 (m, 2H, H-4”), 7.43–7.41 (m, 2H, H-3”),7.39–7.38 (m, 1H, H-5”), 6.77 (bs, 1H, H-4’), 6.19–6.18 (m, 1H, H-3′), 5.99–5.98 (m, 1H, H-2’), 5.39 (s, 2H, H-1”), 1.57 (s, 9H, H-2”’) ppm. ^13^C{^1^H} NMR (150 MHz, DMSO-*d*_6_): 135.2 (o, C-2”), 134.2 (+, C-2), 132.3 (o, C-4), 129.0 (+, C-3”), 128.6 (+, C-5”), 128.4 (+, C-4”), 128.0 (+, C-4’), 124.9 (+, C-5), 119.0 (o, C-1’), 111.5 (+, C-3′), 107.2 (+, C-2’), 61.4 (o, C-1”’), 51.7 (+, C-1”), 29.3 (+, C-2”’) ppm. IR (ATR): 3099, 3034, 2946, 2678, 1616, 1577, 1450, 1376, 1197, 1133, 1105, 873, 834, 719, 640, 611, 499 cm^–1^. HRMS (ESI): *m*/*z* calcd. for [M + H]^+^ 280.1808; found 280.1818.

#### 2-(3-(*tert*-Butyl)-1-(4-methylbenzyl)-1H-imidazolium-4-yl)pyrrol-1-ide (**8i**)

0.350 g (0.94 mmol) of salt **7i** were used, which yielded 2-(3-(*tert*-butyl)-1-(4-methylbenzyl)-1H-imidazolium-4-yl)pyrrol-1-ide **8i**, 0.258 g, 94%, as a yellow solid, mp 125.3–127.4 °C. ^1^H NMR (600 MHz, CD_3_OD): 7.58 (s, 1H, H-5), 7.35 (d, *J* = 7.9 Hz, 2H, H-3”), 7.26 (d, *J* = 7.9 Hz, 2H, H-4”), 6.94 (dd, *J* = 1.4, 2.8 Hz, 1H, H-4’), 6.93 (dd, *J* = 1.4, 3.5, 1H, H-3′), 6.24–6.23 (m, 1H, H-2’), 5.36 (s, 2H, H-1”), 2.34 (s, 3H, H-6”), 1.56 (s, 9H, H-2”’) ppm. ^2^H NMR (92 MHz, CD_3_OD): 9.23 (bs, 1H, H-2) ppm. ^13^C{^1^H} NMR (150 MHz, CD_3_OD): 140.6 (o, C-5”), 132.4 (o, C-2”), 131.0 (+, C-4”), 129.8 (o, C-4), 129.6 (+, C-3”), 125.8 (+, C-5), 121.5 (+, C-4’), 115.6 (+, C-3′), 115.5 (o, C-1’), 109.9 (+, C-2’), 63.7 (o, C-1”’), 53.9 (-, C-1”), 30,1 (+, C-2”’), 21.2 (+, C-6”) ppm. C-2 not observed. IR (ATR): 3090, 3025, 2931, 2695, 2596, 1614, 1532, 1449, 1381, 1322, 1226, 1191, 1148, 1123, 1000, 973, 909, 876, 835, 806, 738, 695, 643, 466 cm^–1^. HRMS (ESI): *m*/*z* calcd. for [M + H]^+^ 294.1965; found 294.1971.

#### General Procedure for the Synthesis of the Selenones

In an oven-dried round-bottom flask, equipped with a magnetic stirrer and a reflux condenser, 1 eq. of betaine (**8a**–**i**) was dissolved in dry acetonitrile under inert atmosphere. Then 2 eq. of cesium carbonate and 2 eq. of selenium were added to the solution. The mixture was then heated to 85 °C in an oil bath for 12 h. After the reaction, the solution was filtered through Celite, and purified by column chromatography.

#### 1,3-Dimethyl-4-(1H-pyrrol-2-yl)-1,3-dihydro-2H-imidazole-2-selenone (**9a**)

0.100 g (0.62 mmol) of betaine **8a**, 0.404 g (1.24 mmol) of cesium carbonate, and 0.098 g (1.24 mmol) selenium were used. The residue was purified by column chromatography (ethyl acetate) to afford 1,3-dimethyl-4-(1H-pyrrol-2-yl)-1,3-dihydro-2H-imidazole-2-selenone **9a**, 0.101 g, 68%, as a brown solid, mp 151.8–153.4 °C. ^1^H NMR (600 MHz, DMSO-*d*_6_): 11.26 (s, 1H, H-5′), 7.38 (s, 1H, H-5), 6.94–6.92 (m, 1H, H-4’), 6.35–6.34 (m, 1H, H-3′), 6.17–6.16 (m, 1H, H-2’), 3.63 (s, 3H, H-1”), 3.61 (s, 1H, H-2”) ppm. ^13^C{^1^H} NMR (150 MHz, DMSO-*d*_6_): 155.6 (o, C-2), 125.8 (o, C-4 or C-1’), 119.9 (+, C-4’), 118.1 (o, C-4 or C-1’), 117.1 (+, C-5), 109.3 (+, C-3′), 108.7 (+, C-2’), 36.4 (+, C-1” or C-2”), 35.1 (+, C-1” or C-2”) ppm. ^77^Se NMR (114 MHz, DMSO-*d*_6_): 52.1 ppm. IR (ATR): 3139, 3081, 3055, 2938, 1711, 1634, 1535, 1416, 1375, 1263, 1192, 1131, 1101, 1023, 916, 809, 782, 724, 632, 598, 540 cm^–1^. HRMS (ESI): *m*/*z* calcd. for [M + Na]^+^ 264.0010; found 264.0008.

#### 1,3-Dimethyl-5-phenyl-4-(1H-pyrrol-2-yl)-1,3-dihydro-2H-imidazole-2-selenone (**9b**)

0.130 g (0.55 mmol) of betaine **8b**, 0.357 g (1.10 mmol) cesium carbonate, and 0.087 g (1.10 mmol) selenium were used. The residue was purified by column chromatography (ethyl acetate/petrolether 1:3) to afford 1,3-dimethyl-4-phenyl-5-(1H-pyrrol-2-yl)-1,3-dihydro-2H-imidazole-2-selenone **9b** 0.049 g, 28%, as a dark blue solid, mp 151–153.6 °C. ^1^H NMR (600 MHz, DMSO-*d*_6_): 11.10 (s, 1H, H-5′), 7.43–7.37 (m, 5H, H-2″, H-3″, H-4”), 6.88–6.87 (m, 1H, H-4’), 6.14–6.13 (m, 1H, H-3′), 6.07–6.05 (m, 1H, H-2’), 3.54 (s, 3H, H-2”’), 3.49 (s, 3H, H-1”’) ppm. ^13^C{^1^H} NMR (150 MHz, DMSO-*d*_6_): 155.7 (o, C-2), 130.4 (o, C-1”), 130.2 (+, C-2” or C-3”), 128.9 (+, C-4”), 128.5 (+, C-2” or C-3”), 127.9 (o, C-5), 122.9 (o, C-4), 120.2 (+, C-4’), 116.5 (o, C-1’), 112.0 (+, C-3′), 108.3 (+, C-2’), 35.2 (+, C-2”’), 34.8 (+, C-1”’) ppm. ^77^Se NMR (114 MHz, DMSO-*d*_6_): 68.2 ppm. IR (ATR): 3262, 3051, 2927, 2226, 2055, 1884, 1813, 1731, 1634, 1593, 1442, 1371, 1251, 1123, 1094, 1026, 929, 874, 814, 760, 727, 686, 621, 584, 503, 435 cm^–1^. HRMS (ESI): *m*/*z* calcd. for [M + Na]^+^ 340.0323 found 340.0325.

#### 1,3-Dimethyl-4-(1H-pyrrol-2-yl)-5-(p-tolyl)-1,3-dihydro-2H-imidazole-2-selenone (**9c**)

0.100 g (0.40 mmol) of betaine **8c**, 0.259 g (0.80 mmol) of cesium carbonate, and 0.063 g (0.80 mmol) of selenium were used. The residue was purified by column chromatography (ethyl acetate/petrolether 1:3) to afford 1,3-dimethyl-4-(1H-pyrrol-2-yl)-5-(p-tolyl)-1,3-dihydro-2H-imidazole-2-selenone **9c**, 0.068 g, 52%, as an orange solid, mp 198–199.5 °C. ^1^H NMR (600 MHz, DMSO-*d*_6_): 11.08 (s, 1H, H-5′), 7.27 (d, *J* = 8.1 Hz, 2H, H-2”), 7.21 (d, *J* = 8.1 Hz, 2H, H-3”), 6.87–6.86 (m, 1H, H-4’), 6.13–6.12 (m, 1H, H-3′), 6.06–6.05 (m, 1H, H-2’), 3.53 (s, 3H, H-2”’), 3.48 (s, 3H, H-1”’), 2.31 (s, 3H, H-5”) ppm. ^13^C{^1^H} NMR (150 MHz, DMSO-*d*_6_): 155.5 (o, C-2), 138.5 (o, C-4”), 130.5 (o, C-5), 130.1 (+, C-2”), 129.1 (+, C-3”), 124.9 (o, C-1”), 122.8 (o, C-4), 120.2 (+, C-4’), 116.6 (o, C-1’), 111.9 (+, C-3′), 108.3 (+, C-2’), 35.1 (+, C-2”’), 34.8 (+, C-1”’), 20.8 (+, C-5”) ppm. ^77^Se NMR (114 MHz, DMSO-*d*_6_): 67.2 ppm. IR (ATR): 3242, 3140, 2937, 1512, 1444, 1373, 1261, 1191, 1128, 1099, 1025, 809, 782, 717, 662, 632, 593, 508 cm^–1^. HRMS (ESI): *m*/*z* calcd. for [M + Na]^+^ 354.0480; found 354.0482.

#### 5-(4-Methoxyphenyl)-1,3-dimethyl-4-(1H-pyrrol-2-yl)-1,3-dihydro-2H-imidazole-2-selenone (**9d**)

0.100 g (0.37 mmol) of betaine **8d**, 0.243 g (0.74 mmol) of cesium carbonate, and 0.059 g (0.74 mmol) of selenium were used. The residue was purified by column chromatography (ethyl acetate/petrolether 1:3) to afford 4-(4-methoxyphenyl)-1,3-dimethyl-5-(1H-pyrrol-2-yl)-1,3-dihydro-2H-imidazole-2-selenone **9d**, 0.063 g, 49%, as a dark green solid, mp 133.6–135.4 °C. ^1^H NMR (600 MHz, DMSO-*d*_6_): 11.07 (s, 1H, H-5′), 7.31 (d, *J* = 8.8 Hz, 2H, H-2”), 6.96 (d, *J* = 8.8 Hz, 2H, H-3”), 6.87–6.86 (m, 1H, H-4’), 6.13–6.12 (m, 1H, H-3′), 6.06–6.05 (m, 1H, H-2’), 3.76 (s, 3H, H-5”), 3.51 (s, 3H, H-2”’) 3,47 (s, 3H, H-1”’) ppm. ^13^C{^1^H} NMR (150 MHz, DMSO-*d*_6_): 159.7 (o, C-4’), 155.2 (o, C-2), 131.7 (+, C-2”), 130.3 (o, C-5), 122.7 (o, C-1”) 120.2 (+, C-4’), 119.9 (o, C-4), 116.7 (o, C-1’), 111.9 (+, C-3′), 108.3 (+, C-2’), 55.2 (+, C-5”), 35.1 (+, C-2”’), 34.8 (+, C-1”’) ppm. ^77^Se NMR (114 MHz, DMSO-*d*_6_): 66.0 ppm. IR (ATR): 3245, 2962, 2838, 1650, 1606, 1562, 1508, 1443, 1371, 1291, 1248, 1176, 1096, 1021, 997, 932, 838, 825, 762, 718, 656, 587, 524, 468 cm^–1^. HRMS (ESI): *m*/*z* calcd. for [M + Na]^+^ 370.0429; found 370.0428.

#### 1-Benzyl-3-methyl-4-(1H-pyrrol-2-yl)-1,3-dihydro-2H-imidazole-2-selenone (**9e**)

0.105 g (0.44 mmol) of betaine **8e**, 0.288 g (0.88 mmol) of cesium carbonate, and 0.070 g (0.88 mmol) of selenium were used. The residue was purified by column chromatography (ethyl acetate/petrolether 3:1) to afford 1-benzyl-3-methyl-4-(1H-pyrrol-2-yl)-1,3-dihydro-2H-imidazole-2-selenone **9e**, 0.059 g, 42%, as a green/blue solid, mp 178.8–180.5 °C. ^1^H NMR (600 MHz, DMSO-*d*_6_): 11.28 (bs, 1H, H-5′), 7.44–7.42 (m, 3H, H-5, H-3”), 7.39–7.37 (m, 2H, H-4”), 7.34–7.31 (m, 1H, H-5”), 6.93–6.92 (m, 1H, H-4’), 6.39–6.38 (m, 1H, H-3′), 6.18–6.16 (m, 1H, H-2’), 5.40 (s, 2H, H-1”), 3.71 (s, 3H, H-1”’) ppm. ^13^C{^1^H} NMR (150 MHz, DMSO-*d*_6_): 156.0 (o, C-2), 136.6 (o, C-2”), 128.6 (+, C-4”),128.0 (+, C-3”), 127.8 (o, C-5”), 126.5 (o, C-4), 120.0 (+, C-4’), 118.1 (o, C-1’), 115.9 (+, C-5), 109.3 (+, C-3′), 108.8 (+, C-2’), 51.6 (-, C-1”), 35.3 (+, C-1”’) ppm. ^77^Se NMR (114 MHz, DMSO-*d*_6_): 50.3 ppm. IR (ATR): 3107, 2967, 2924, 1663, 1592, 1513, 1337, 1226, 1105, 1036, 852, 817, 727, 695, 602, 509 cm^–1^. HRMS (ESI): *m*/*z* calcd. for [M + H]^+^ 283.1190; found 283.1186.

#### 3-Methyl-1-(4-methylbenzyl)-4-(1H-pyrrol-2-yl)-1,3-dihydro-2H-imidazole-2-selenone (**9f**)

0.200 g (0.79 mmol) of betaine **8f**, 0.519 g (1.59 mmol) of cesium carbonate, and 0.126 g (1.59 mmol) of selenium were used. The residue was purified by column chromatography (ethyl acetate/petrolether 1:3) to afford 3-methyl-1-(4-methylbenzyl)-4-(1H-pyrrol-2-yl)-1,3-dihydro-2H-imidazole-2-selenone **9f**, 0.155 g, 59%, as an orange solid, mp 156.3–158.2 °C. ^1^H NMR (600 MHz, DMSO-*d*_6_): 11.25 (s, 1H, H-5′), 7.36 (s, 1H, H-5), 7.32 (d, *J* = 8.0 Hz, 2H, H-3”), 7.17 (d, *J* = 8.0 Hz, 2H, H-4”), 6.91–6.90 (m, 1H, H-4’), 6.37–6.35 (m, 1H, H-3′), 6.16–6.14 (m, 1H, H-2’), 5.33 (s, 2H, H-1”), 3.69 (s, 3H, H-1”’), 2.28 (s, 3H, H-6”) ppm. ^13^C{^1^H} NMR (150 MHz, DMSO-*d*_6_): 155.9 (o, C-2), 137.1 (o, C-5”), 133.6 (o, C-2”), 129.1 (+, C-4”), 128.1 (+, C-3”), 126.4 (o, C-4 or C-1’), 120.0 (+, C-4’), 115.7 (+, C-5), 109.4 (+, C-3′), 108.7 (+, C-2’),108.1 (o, C-5 or C-1’), 51.0 (-, C-1”), 35.4 (+, C-1”’), 20.7 (+, C-6”) ppm. ^77^Se NMR (114 MHz, DMSO-*d*_6_): 50.4 ppm. IR (ATR): 3247, 3140, 2938, 1711, 1635, 1533, 1459, 1377, 1261, 1199, 1102, 1029, 918, 780, 744, 718, 664, 631, 579, 544, 516, 477 cm^–1^. HRMS (ESI): *m*/*z* calcd. for [M + Na]^+^ 354.0480; found 354.0489.

#### 1-(4-Methoxybenzyl)-3-methyl-4-(1H-pyrrol-2-yl)-1,3-dihydro-2H-imidazole-2-selenone (**9g**)

0.108 g (0.40 mmol) of betaine **8g**, 0.263 g (0.81 mmol) of cesium carbonate, and 0.64 g (0.81 mmol) of selenium were used. The residue was purified by column chromatography (ethyl acetate/petrolether 1:3) to afford 1-(4-methoxybenzyl)-3-methyl-4-(1H-pyrrol-2-yl)-1,3-dihydro-2H-imidazole-2-selenone **9g**, 0.068 g, 49%, as an orange solid, mp 151.2–153 °C. ^1^H NMR (600 MHz, DMSO-*d*_6_): 11.24 (s, 1H, H-5′), 7.40 (d, *J* = 8.8 Hz, 2H, H-3”), 7.36 (s, 1H, H-5), 6.92 (d, *J* = 8.8 Hz, 2H, H-4”), 6.91–6.90 (m, 1H, H-4’), 6.36–6.35 (m, 1H, H-3′), 6.15–6.14 (m, 1H, H-2’), 5.29 (s, 2H, H-1”), 3.73 (s, 3H, H-6”), 3.67 (s, 3H, H-1”’) ppm. ^13^C{^1^H} NMR (150 MHz, DMSO-*d*_6_): 158.9 (o, C-5”), 155.6 (o, C-2), 129.7 (+, C-3”), 133.6 (o, C-2”), 129.1 (+, C-4”), 128.1 (+, C-3”), 126.4 (o, C-4 or C-1’), 120.0 (+, C-4’), 115.7 (+, C-5), 128.6 (o, C-1’ or C-4), 126.4 (o, C-2”), 120.0 (+, C-4’), 118.1 (o, C-1’ or C-5), 115.7 (+, C-4), 113.9 (+, C-4”), 109.3 (+, C-3′), 108.7 (+, C-2’), 55.1 (+, C-6”), 51.1 (-, C-1”), 35.2 (+, C-1”’) ppm. ^77^Se NMR (114 MHz, DMSO-*d*_6_): 50.3 ppm. IR (ATR): 3192, 3125, 2926, 1601, 1510, 1454, 1391, 1337, 1303, 1244, 1171, 1103, 1025, 849, 814, 779, 724, 637, 596, 510 cm^–1^. HRMS (ESI): *m*/*z* calcd. for [M + Na]^+^ 370.0429; found 370.0429.

#### 1-Benzyl-3-(*tert*-butyl)-4-(1H-pyrrol-2-yl)-1,3-dihydro-2H-imidazole-2-selenone (**9h**)

0.095 g (0.34 mmol) of betaine **8h**, 0.220 g (0.69 mmol) of cesium carbonate, and 0.054 g (0.68 mmol) of selenium were used. The residue was purified by column chromatography (ethyl acetate/petrolether 1:3) to afford 1-benzyl-3-(*tert*-butyl)-4-(1H-pyrrol-2-yl)-1,3-dihydro-2H-imidazole-2-selenone **9h**, 0.063 g, 51%, as a colorless solid, mp 170.5–173 °C. ^1^H NMR (600 MHz, DMSO-*d*_6_): 11.17 (s, 1H, H-2), 7.46–7.45 (m, 2H, H-3”), 7.38 (s, 1H, H-5), 7.37–7.34 (m, 2H, H-4”),7.31–7.28 (m, 1H, H-5”), 6.84–6.83 (m, 1H, H-4’), 6.12–6.11 (m, 1H, H-3′), 6.05–6.04 (m, 1H, H-2’), 5.45 (s, 2H, H-1”), 1.69 (s, 9H, H-2”’) ppm. ^13^C{^1^H} NMR (150 MHz, DMSO-*d*_6_): 154.0 (o, C-2), 136.7 (o, C-2”), 128.5 (+, C-4”), 128.3 (+, C-3”), 127.7 (+, C-5”), 124.8 (o, C-1’), 122.1 (+, C-5), 119.5 (o, C-4), 118.8 (+, C-4’), 111.9 (+, C-3′), 107.8 (+, C-2’), 62.5 (o, C-1”’), 50.9 (-, C-1”), 28.9 (+, C-2”’) ppm. ^77^Se NMR (114 MHz, DMSO-*d*_6_): 183.1 ppm. IR (ATR): 3204, 3055, 2935, 1623, 1529, 1491, 1452, 1402, 1334, 1256, 1207, 1172, 1104, 1038, 953, 913, 790, 738, 715, 593, 587, 550, 481 cm^–1^. HRMS (ESI): *m*/*z* calcd. for [M + Na]^+^ 382.0793; found 382.0796.

#### 3-(*tert*-Butyl)-1-(4-methylbenzyl)-4-(1H-pyrrol-2-yl)-1,3-dihydro-2H-imidazole-2-selenone (**9i**)

0.137 g (0.47 mmol) of betaine **8i**, 0.304 g (0.94 mmol) of cesium carbonate, and 0.074 g (0.94 mmol) of selenium were used. The residue was purified by column chromatography (ethyl acetate/petrolether 1:3) to afford 3-(*tert*-butyl)-1-(4-methylbenzyl)-4-(1H-pyrrol-2-yl)-1,3-dihydro-2H-imidazole-2-selenone **9i**, 0.086 g, 49%, as an orange solid, mp 163.7–165.1 °C. ^1^H NMR (600 MHz, DMSO-*d*_6_): 11.15 (s, 1H, H-2), 7.36 (d, *J* = 7.8 Hz, 2H, H-3”), 7.33 (s, 1H, H-5), 7.15 (d, *J* = 7.8, 2H, H-4”),6.83–6.82 (m, 1H, H-4’), 6.10–6.09 (m, 1H, H-3′), 6.04–6.03 (m, 1H, H-2’), 5.39 (s, 2H, H-1”), 2.28 (s, 3H, H-6”), 1.68 (s, 9H, H-2”’) ppm. ^13^C{^1^H} NMR (150 MHz, DMSO-*d*_6_): 153.7 (o, C-2), 137.0 (o, C-5”), 133.7 (o, C-2”), 129.0 (+, C-4”), 128.4 (+, C-3”), 124.7 (o, C-1’), 122.0 (+, C-5), 119.6 (o, C-4), 118.8 (+, C-4’), 111.9 (+, C-3′), 107.8 (+, C-2’), 62.5 (o, C-1”’), 50.7 (-, C-1”), 28.9 (+, C-2”’),20.7 (+, C-6”) ppm. ^77^Se NMR (114 MHz, DMSO-*d*_6_): 183.3 ppm. IR (ATR): 3219, 3105, 2966, 2923, 1593, 1512, 1391, 1337, 1218, 1104, 1023, 853, 814, 723, 694, 639, 601, 480 cm^–1^. HRMS (ESI): *m*/*z* calcd. for [M + Na]^+^ 396.0949; found 396.0945.

#### General Procedure for the In Situ Deprotonation of Selenones

In an NMR tube, 20 mg of the selenone to be measured was dissolved in DMSO-*d*_6_ and mixed portionwise with 0.25 eq. of KO-*t*-Bu. The solutions were allowed to rest for 15 min after each addition until measurement. Measurements were carried out with 0.25, 0.75, 0.5, and 2 eq. of base. The chemical shifts indicated refer to the measurement after the addition of 2 eq. of base.

#### 2-(1,3-Dimethyl-2-selenoxo-2,3-dihydro-1H-imidazol-4-yl)pyrrol-1-ide (**10a**)

0.020 g of selenone **9a** were measured. ^1^H NMR (600 MHz, DMSO-*d*_6_ + 2eq KO*t*-Bu): 6.93 (s, 1H, H-5), 6.63–6.62 (m, 1H, H-4’), 6.08 (dd. J = 1.3, 3.0 Hz, 1H, H-3′), 5.84 (dd, *J* = 1.6, 3.0 Hz 1H, H-2’), 3.80 (s, 3H, H-1”), 3.53 (s, 1H, H-2”) ppm. ^13^C{^1^H} NMR (150 MHz, DMSO-*d*_6_): 151.7 (o, C-2), 134.1 (o, C-4), 128.3 (+, C-4’), 125.6 (o, C-1’), 112.0 (+, C-5), 106.2 (+, C-2’), 105.9 (+, C-3′), 31.1 (+, C-1” or C-2”), 35.7 (+, C-1” or C-2”) ppm. ^77^Se NMR (114 MHz, DMSO-*d*_6_): 31.8 ppm.

#### 2-(1,3-Dimethyl-5-phenyl-2-selenoxo-2,3-dihydro-1H-imidazol-4-yl)pyrrol-1-ide (**10b**)

0.020 g of selenone **9b** were measured. ^1^H NMR (600 MHz, DMSO-*d*_6_): 7.41–7.32 (m, 5H, H-2″, H-3″, H-4”), 6.55–6.54 (m, 1H, H-4’), 5.62 (dd, *J* = 1.5, 2.9 Hz, 1H, H-3′), 5.57 (dd, *J* = 1.2, 2.9 Hz, 1H, H-2’), 3.84 (s, 3H, H-2”’), 3.43 (s, 3H, H-1”’) ppm. ^13^C{^1^H} NMR (150 MHz, DMSO-*d*_6_): 152.2 (o, C-2), 131. (o, C-1”), 130.9 (+, C-2” or C-3”), 128.9 (+, C-4’), 128.6 (o, C-4), 128.2 (+, C-4”), 127.8 (+, C-2” or C-3”), 125.1 (o, C-1’), 123.8 (o, C-5), 108.0 (+, C-2’), 105.5 (+, C-3′), 35.9 (+, C-2”’), 34.9 (+, C-1”’) ppm. ^77^Se NMR (114 MHz, DMSO-*d*_6_): 48.9 ppm.

#### 2-(1-Benzyl-3-methyl-2-selenoxo-2,3-dihydro-1H-imidazol-4-yl)pyrrol-1-ide (**10e**)

0.020 g of selenone **9e** were measured. ^1^H NMR (600 MHz, DMSO-*d*_6_): 7.38–7.37 (m, 2H, H-3”), 7.35–7.33 (m, 2H, H-4”), 7.29–7.24 (m, 1H, H-5”), 6.94 (s, 1H, H-5), 6.65–6.64 (m, 1H, H-4’), 6.12–6.11 (m, 1H, H-3′), 5.87–5.86 (m, 1H, H-2’), 5.32 (s, 2H, H-1”), 3.86 (s, 3H, H-1”’) ppm. ^13^C{^1^H} NMR (150 MHz, DMSO-*d*_6_): 152.7 (o, C-2), 137.2 (o, C-2”), 133.6 (o, C-1’ or C-4) 128.4 (+, C-3” or C-4”),127.9 (+, C-3” or C-4”), 127.5 (+, C-5”), 127.3 (o, C-4’), 124.6 (+, C-4 or C-1’), 118.1 (o, C-1’), 1151.0 (+, C-5), 106.5 (+, C-2’), 106.3 (+, C-3′), 51.3 (-, C-1”), 35.9 (+, C-1”’) ppm. ^77^Se NMR (114 MHz, DMSO-*d*_6_): 32.7 ppm.

## Data Availability

The data underlying this study are available in the published article and its Supporting Information.

## Supporting Information Available

^1^H NMR data for all synthesized components; ^13^C{^1^H}-NMR data and 2D-NMR data for all unknown compounds; ^77^Se NMR data for all compounds containing selenium and for the temperature experiments data from the DFT calculations; HOMO/LUMO profiles; and calculations for geometry optimization of **9a**,**9e**, and **9h** (PDF)
