# Supplementary material for: Anionic N-Heterocyclic Carbenes from Mesoionic Imidazolium-4-pyrrolides: The Influence of Substituents, Solvents, and Charge on their 77Se NMR Chemical Shifts
Source: J Org Chem. 2024 Oct 3;89(20):15003–19. doi: 10.1021/acs.joc.4c01732 (PMC11494649; doi:10.1021/acs.joc.4c01732)
Supplement: Supplementary file 1 — jo4c01732_si_001.pdf [file jo4c01732_si_001.pdf]

Supporting Information

Anionic *N*-Heterocyclic Carbenes from Mesoionic Imidazolium-4-pyrrolides. The influence of Substituents, Solvents, and Charge on their ^{77}Se NMR Chemical Shifts

Lucas Pruschinski, Jan C. Namyslo, Andreas Schmidt*

E-mail: schmidt@ioc.tu-clausthal.de

Clausthal University of Technology, Institute of Organic Chemistry, Leibnitzstraße 6,

D-38678 Clausthal-Zellerfeld, Germany

Table of contents

1. Synthesis of aliphatic and aryl Imines **(2a-c)** p. S1
2. Synthesis of TosMIC-precursors **(4a-c)** p. S2
3. Synthesis of substituted TosMIC-derivatives **5(a-c)** p. S3
4. Solvents effects on Diphenyl diselenide p. S4
5. NMR data of aliphatic and aryl Imines **(2a-c)** p. S5
6. NMR data of TosMIC-precursors **(4a-c)** p. S8
7. NMR data of substituted TosMIC-derivatives **5(a-c)** p. S11
8. NMR Data of Imidazoles **(6a-e)** p. S14
9. NMR data of Imidazolium Salts **(7a-i)** p. S31
10. NMR data of Betaines **(8a-i)** p. S76
11. NMR data of the Selenones **(9a-i)** p. S125
12. Deprotonation experiments selenones **(10a-c)** p. S179
13. Temperature measurements ^{77}Se p. S 203
14. Internal and external Referencing experiments p. S 207
15. DFT calculations p. S209
16. Geometry optimised structures of 9a 9h and 9e p. S244
17. References p. S249

General procedure for the synthesis of aliphatic Imines (Procedure A):

In a round-bottom flask, equipped with a magnetic stirrer, 1 eq. of pyrrole-2-carbaldehyde was dissolved in hexane. 2.5 eq. of the corresponding amine were then added to the solution while stirring frequently. The mixture was then stirred at room temperature until full conversion was observed by TLC. Subsequently, 25 ml of distilled water were added, and the resulting mixture was further stirred for 30 minutes. The organic layer was separated, and the aqueous phase was washed twice with hexane. The collected organic layers were dried with magnesium sulfate and the organic phase was evaporated to yield the desired product, which was used without further purification.

General procedure for the synthesis of aryl Imines (Procedure B):

In an oven dried round-bottom flask, equipped with a magnetic stirrer, 1 eq of pyrrole-2-carbaldehyde and1.2 eq of aniline were dissolved in dry methanol under inert atmosphere. A catalytic amount of formic acid was added and the resulting solution was stirred for 72 hours at room temperature. The solvent was removed in vacuo. The obtained residues were purified by column chromatography.

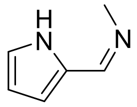

***N*-Methyl-1-(1H-pyrrole-2-yl)methanimine (2a)**: According to Procedure A, a solution of 2.00 g (21 mmol) pyrrole-2-carbaldehyde and 6.57 mL (52 mmol) of methanamine (8 mol/L in methanol) in 25 mL of hexane was stirred for 1 hour. After extraction, the organic solvent was evaporated, the reaction yielded *N*-Methyl-1-(1H-pyrrole-2-yl-methanimine) (2a), 2.21 g, 97 %, as an orange solid. ^{1}H-NMR (400 MHz, $CDCl_3$): 8.07-8.06 (m, 1H), 6.89-6.88 (m, 1H), 6.48-6.47 (m, 1H), 6.23 (dd, J=2.5, 3.6 Hz, 1H), 3.42 (d J=1.5 Hz, 3H). Spectroscopic data are in agreement with those reported in literature[1].

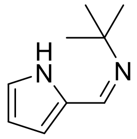

***N-tert*-Butyl-1-(1H-pyrrol-2-yl)methanimine (2b):** According to Procedure A, a solution of 1.50 g (16 mmol) pyrrole-2-carbaldehyde and 4.4 mL (42 mmol) of 2-methylpropan-2-amine in 25 mL of hexane was stirred for 1 hour. After extraction, the organic solvent was evaporated, the reaction yielded *N-tert*-Butyl-1-(1H-pyrrol-2-yl)methanimine (2b), 2.10 g, 89 %, as a brown solid. ^{1}H-NMR (400 MHz, $CDCl_3$): 8.07 (s, 1H), 7.62 (bs, 1H), 6.86-6.85 (m, 1H), 6.45 (dd, J=1.4, 3.6 Hz, 1H), 6.23 (d J=2.6, 3.6 Hz, 1H), 1.25 (s, 9H). Spectroscopic data are in agreement with those reported in literature[2].

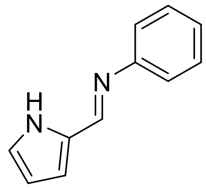

***N*-Phenyl-1-(1H-pyrrol-2-yl)methanimine (2c):**According to Procedure B, 1.50 g (15 mmol) of pyrrole-2-carbaldehyde and 1.61 g (17 mmol) of aniline were dissolved in dry methanol under inert atmosphere and a catalytic amount of formic acid was added after the reaction time described. The obtained residues were purified by column chromatography (petroleum ether/ ethyl acetat= 9:1). The reaction yielded N-Phenyl-1-(1H-pyrrol-2-yl)methanimine (2c), 2.17 g, 81 %, as a colourless solid. ^{1}H-NMR (400 MHz, $CDCl_3$): 10.05 (bs, 1H), 8.26 (d, J=0.7 Hz, 1H), 7.40-7.36 (m, 2H), 7.23-7.20 (m, 3H), 6.93-6.92 (m, 1H), 6.71 (dd, J=1.4, 3.6, 1H), 6.31 (dd, J=2.6, 3.6 Hz, 1H). Spectroscopic data are in agreement with those reported in literature[2].

General procedure for the synthesis of TosMIC-precursors (Procedure C): In a round bottom flask, equipped with a magnetic stirrer and a reflux condenser, 1 eq. of the corresponding benzaldehyde was dissolved in a mixture of toluene and acetonitrile (1:1). 2.5 eq. formamide, 1.1 eq., freshly distilled, TMSCl and 1.5 eq. 4-methylbenzenesulfinic acid, freshly prepared from the sodium salt according to literature[4], were then added to the solution. The mixture was stirred at 50° C in an oil bath for 5 hours, during which the product started to participate. The mixture was cooled to 0° C, 50 mL of diethyl ether and 100 ml of distilled water were added. The product, being insoluble in the mixture, was filtered of washed with diethyl ether/distilled water (1:2). The product was dried under vacuum for a duration of 4 hours.

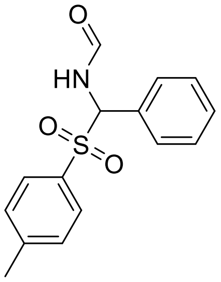

***N*-(Phenyl(tosyl)methyl)formamide (4a):** According to Procedure C, 4.00 g (38 mmol) of benzaldehyde were dissolved in 30 mL of the toluene acetonitrile mixture. Then, 3.76 mL (4.24 g, 94 mmol) of formamide, 5.27 mL (4.50 g, 41 mmol) of TMSCl and 8.80 g (57 mmol) of 4-methylbenzenesulfinic acid, were added and the reaction mixture was heated for the given duration. After the mixture was purified as described, the reaction yielded *N*-(Phenyl(tosyl)methyl)formamide (3a), 8.94 g, 82%, as colourless solid. ^{1}H-NMR (400 MHz, DMSO-d$_6$): 9.79 (d, J=10.6 Hz, 1H), 7.97 (d, J=1.1 Hz, 1H), 7.72 (d, J=8.2 Hz, 2H), 7.57-7.54 (m, 2H), 7.44-7.41 (m, 5H), 6.39 (d, J=10.6 Hz, 1H), 2.41 (s, 3H). Spectroscopic data are in agreement with those reported in literature[3].

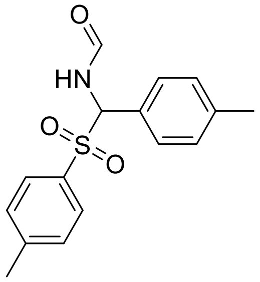

***N*-(*p*-Tolyl(tosyl)methyl)formamide (4b):** According to Procedure C, 5.00 g (37 mmol) of 4-methoxybenzaldehyde were dissolved in 30 mL of the toluene acetonitrile mixture. Then, 3.66 mL (4.13 g, 92 mmol) of formamide, 5.14 mL (4.39 g, 40 mmol) of TMSCl and 8.60 g (55 mmol) of 4-methylbenzenesulfinic acid, were added and the reaction mixture was heated for the given duration. After the mixture was purified as described, the reaction yielded *N*-(p-Tolyl(tosyl)methyl)formamide (3b), 6.52 g, 84%, as beige solid. ^{1}H-NMR (400 MHz, DMSO-d$_6$): 9.73 (d, J=10.6 Hz, 1H), 7.94 (d, J=0.9 Hz, 1H), 7.71 (d, J=8.1 Hz, 2H), 7.43 (d, J=8.1 Hz, 4H), 7.23 (d, J=8.1 Hz, 2H), 6.32 (d, J=10.6 Hz, 1H), 2.40 (s, 3H), 2.33 (s, 3H). Spectroscopic data are in agreement with those reported in literature[4].

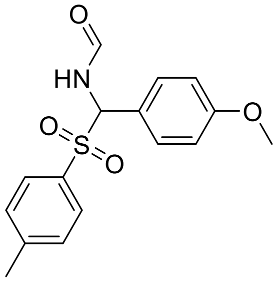

***N*-((4-Methoxyphenyl)(tosyl)methyl)formamide (4c):** According to Procedure C, 5.00 g (37 mmol) of 4-methoxybenzaldehyde were dissolved in 30 mL of the toluene acetonitrile mixture. Then, 3.66 mL (4.13 g, 92 mmol) of formamide, 5.14 mL (4.39 g, 40 mmol) of TMSCl and 8.60 g (55 mmol) of 4-methylbenzenesulfinic acid, were added and the reaction mixture was heated for the given duration. After the mixture was purified as described, the reaction yielded *N*-((4-Methoxyphenyl)(tosyl)methyl)formamide (3c), 10.79 g, 92%, as beige solid. ^{1}H-NMR (400 MHz, DMSO-d_6): 9.71 (d, J=10.6 Hz, 1H), 7.94 (d, J=1.1 Hz, 1H), 7.71 (d, J=8.1 Hz, 2H), 7.48 (d, J=8.7 Hz, 2H), 7.43 (d, J=8.1 Hz, 2H), 6.98 (d, J=8.7 Hz, 2H), 6.32 (d, J=10.6 Hz, 1H), 3.78 (s, 3H), 2.41 (s, 3H). Spectroscopic data are in agreement with those reported in literature[4].

General procedure for the synthesis of substituted TosMIC-derivatives (Procedure D): In an oven dried round-bottom flask, equipped with a magnetic stirrer, a dropping funnel and an internal temperature control, 1eq. formamide **(4a-4c)** was dissolved in dry THF under inert atmosphere, 2 eq. of POCl3 were added to the solution. The Mixture was stirred at room temperature for a duration of 15 min, before it was cooled to 0° C. 6 eq. NEt3 were gradually added to the mixture while keeping the internal temperature between 0° C 10° C. After the addition of the base was complete, the resulting mixture was stirred for 45 min while maintaining the internal temperature between 5° C-10° C. Then 50 mL of distilled water and 50 mL of ethyl acetate were added and the solution was transferred into a separation funnel. The organic layer was separated and rinsed thrice with distilled water, saturated NaHCO3-solution and brine. The volume of the organic phase was reduced under vacuum until 10% of the original volume was left. 100 mL of 1 propanol were added and the volume was reduced again by 50%, the resulting participate was collected via filtration and washed with cooled 1-propanol. The product was dried under vacuum.

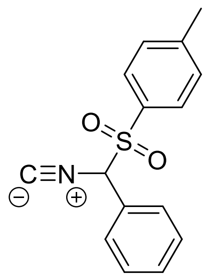

1-((Isocyano(phenyl)methyl)sulfonyl)-4-methylbenzene (5a): According to Procedure D, 4.30 g (15 mmol) of formamide (3a) and 2.78 mL (4.56 g, 30 mmol) of $POCl_3$ were dissolved in 100 mL THF. 12 mL (9.02 g, 89 mmol) of NEt_3 were added dropwise. After workup the reaction yielded 1-((Isocyano(phenyl)methyl)sulfonyl)-4-methylbenzene (4a), 3.52 g, 87%, as colourless solid. ^{1}H-NMR (400 MHz, DMSO-d_6): 7.68-7.66 (m, 2H), 7.54-7.45 (m, 5H), 7.37-7.35 (m, 2H), 6.97 (s, 1H), 2.45 (s, 3H). Spectroscopic data are in agreement with those reported in literature[3].

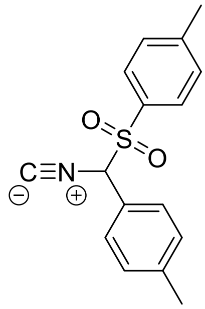

1-((Isocyano(p-tolyl)methyl)sulfonyl)-4-methylbenzene (5b): According to Procedure D, 4.20 g (14 mmol) of formamide (3b) and 2.59 mL (4.24 g, 28 mmol) of $POCl_3$ were dissolved in 100 mL THF. 12 mL (8.40 g, 83 mmol) of NEt_3 were added dropwise. After workup the reaction yielded 1-((Isocyano(p-tolyl)methyl)sulfonyl)-4-methylbenzene (4b), 1.96 g, 50%, as colourless solid. ^{1}H-NMR (400 MHz, DMSO-d_6): 7.62 (d, J=8.5 Hz, 2H), 7.33 (d, J=8.1 Hz, 2H), 7.23-7.18 (m, 4H), 5.57 (s, 1H), 2.47 (s, 3H), 2.39 (s, 3H). Spectroscopic data are in agreement with those reported in literature[3].

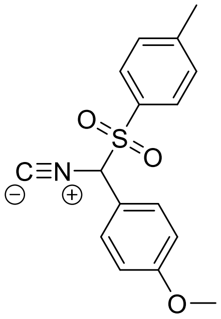

1-((Isocyano(4-methoxyphenyl)methyl)sulfonyl)-4-methylbenzene (5c): According to Procedure D, 9.36 g (29 mmol) of formamide (3c) and 5.48 mL (8.99 g, 58 mmol) of $POCl_3$ were dissolved in 100 mL THF. 24 mL (17.80 g, 175 mmol) of NEt_3 were added dropwise. After workup the reaction yielded 1-((isocyano(4-methoxyphenyl)methyl)sulfonyl)-4-methylbenzene (4c), 7.02 g, 79%, as colourless solid. ^{1}H-NMR (400 MHz, DMSO-d_6): 7.67 (d, J=8.2 Hz, 2H), 7.50 (d, J=8.2 Hz, 2H), 7.20 (d, J=8.5 Hz, 2H), 7.02 (d, J=8.5 Hz, 2H), 6.83 (s, 1H), 3.79 (s, 3H), 2.44 (s, 3H). Spectroscopic data are in agreement with those reported in literature[3].

Table S1. Solvents effects on Diphenyl diselenide

NMR solvent	^{77}Se NMR of PhSe-SePh shift referenced to Ph-Se-Se-Ph in chloroform*	Δδ [ppm] Ph-Se-Se-Ph shift relative to Ph-Se-Se-Ph in chloroform
**Tol-d_8**	458.5	-2.5
**THF-d_8**	453.3	-7.7
**$CDCl_3$**	461.0	0
**CD_2Cl_2**	459.6	-1.4
**acetone-d_6**	449.7	-11.3
**DMSO-d_6**	446.1	-14.9
**CD_3CN**	449.6	-11.4
**MeOD**	456.9	-4.1

* external reference @ 461.0 ppm rel. to $Se(CH_3)_2$ @ 0.0 ppm.[5]

^{1}H-NMR: *N*-Methyl-1-(1H-pyrrole-2-yl)methanimine (2a)

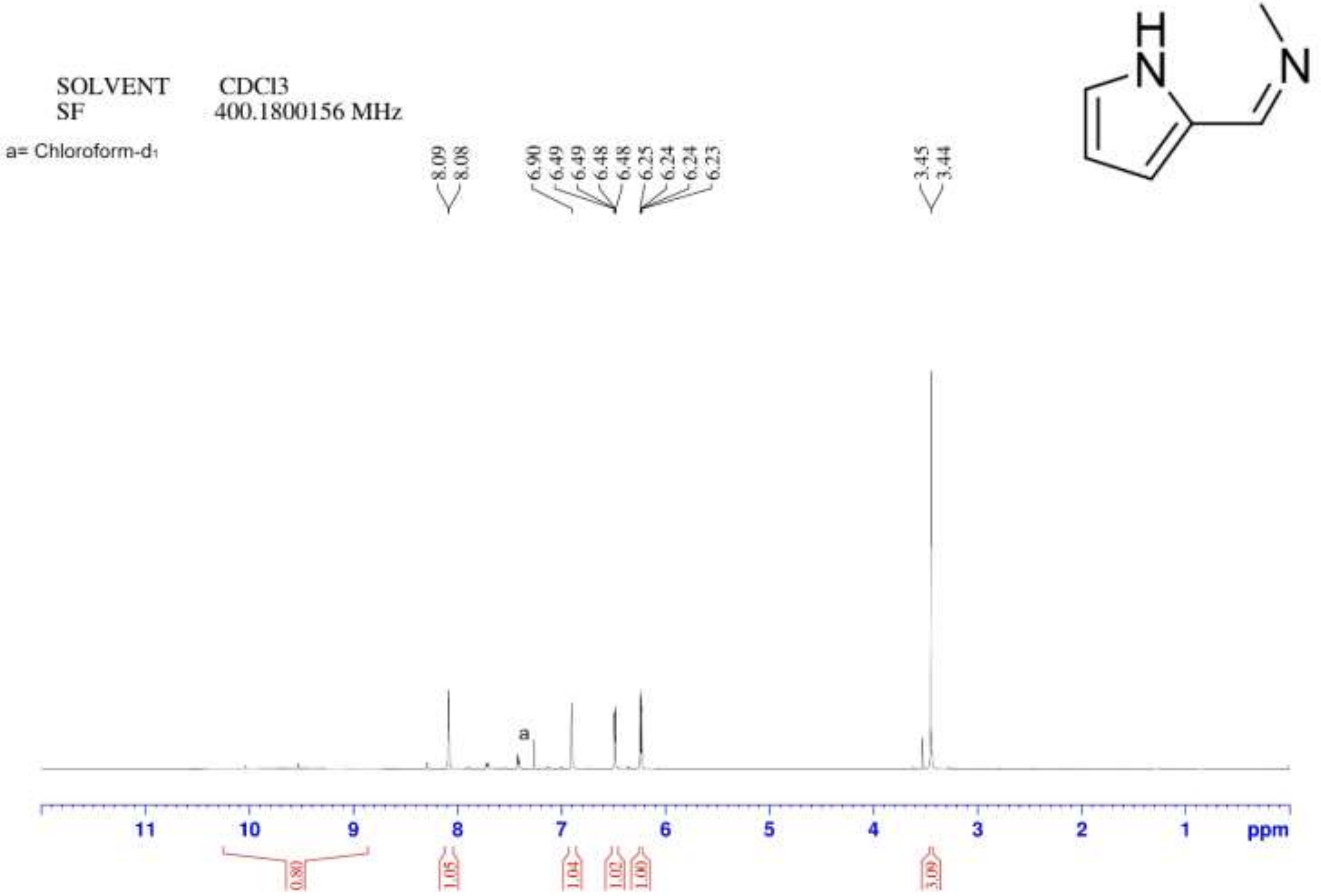

Figure S1. ^{1}H-NMR of compound 2a

^{1}H-NMR: *N-tert*-Butyl-1-(1H-pyrrol-2-yl)methanimine (2b):

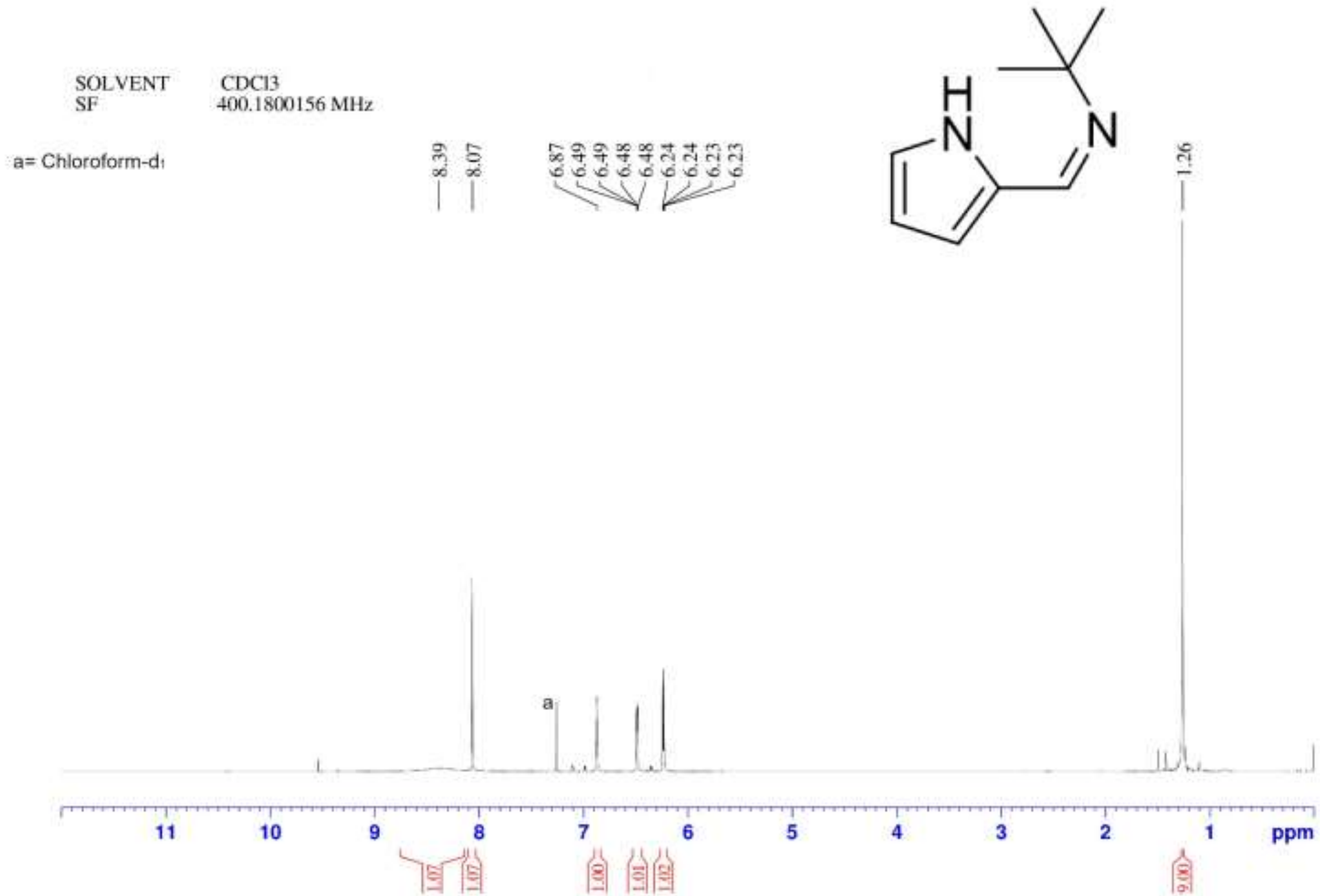

Figure S2. ^{1}H-NMR of compound 2

^{1}H-NMR: *N*-Phenyl-1-(1H-pyrrol-2-yl)methanimine (2c):

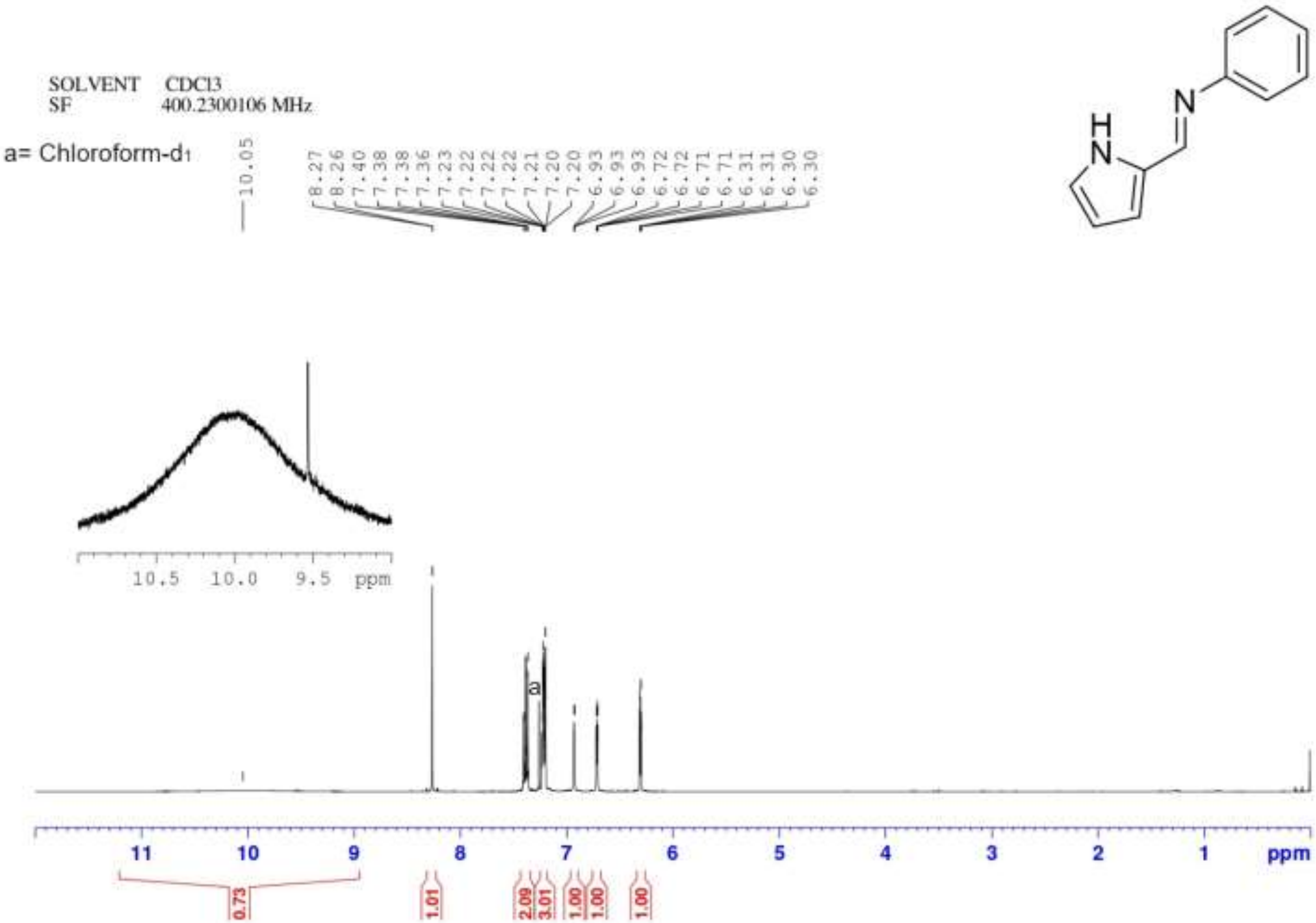

Figure S3. ^{1}H-NMR of compound 2

^{1}H-NMR: *N*-(Phenyl(tosyl)methyl)formamide (4a):

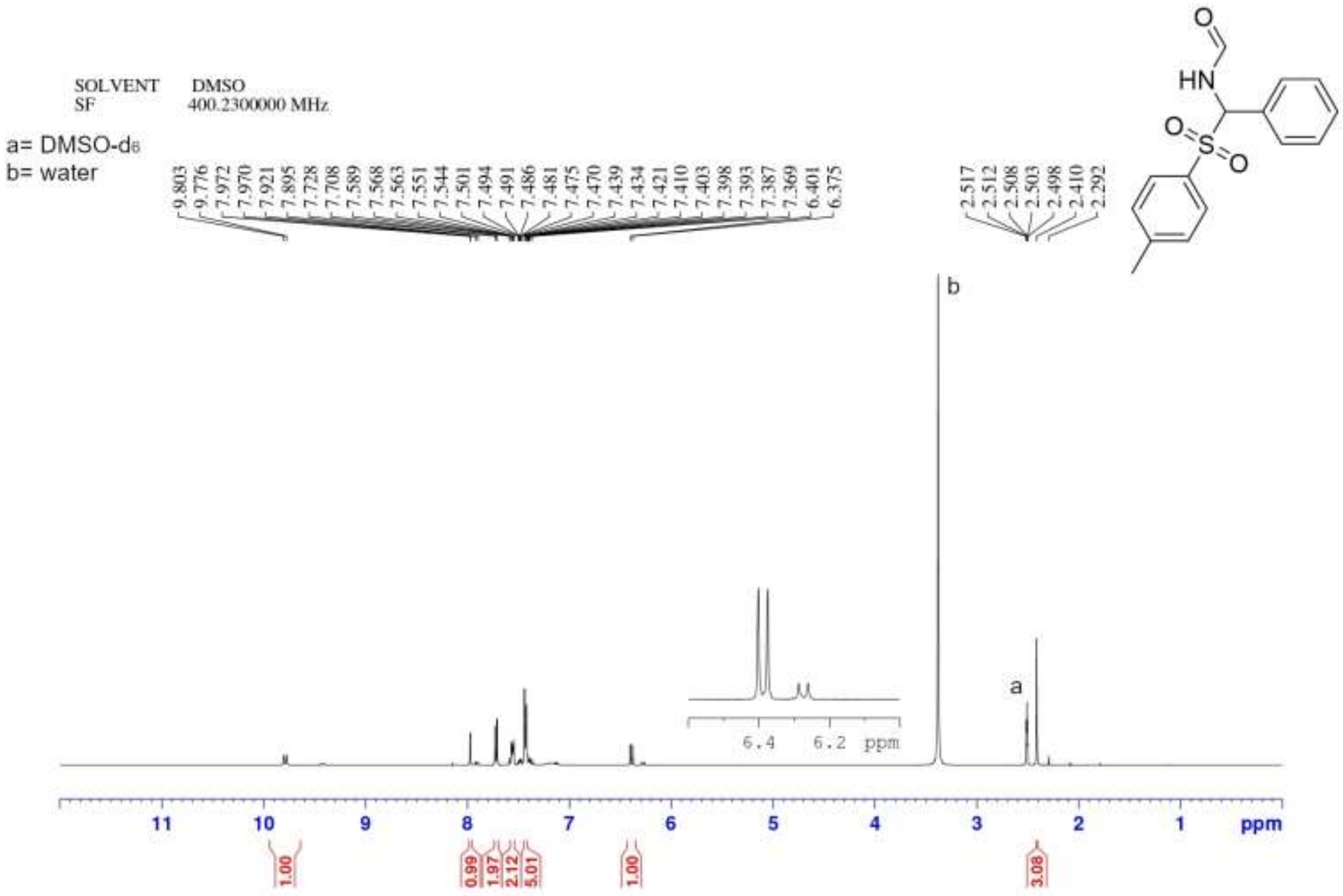

Figure S4. [1]**H-NMR of compound 4a, Compound 4a exists as a mixture of amide rotamers.**

^{1}H-NMR *N*-(*p*-Tolyl(tosyl)methyl)formamide (4b):

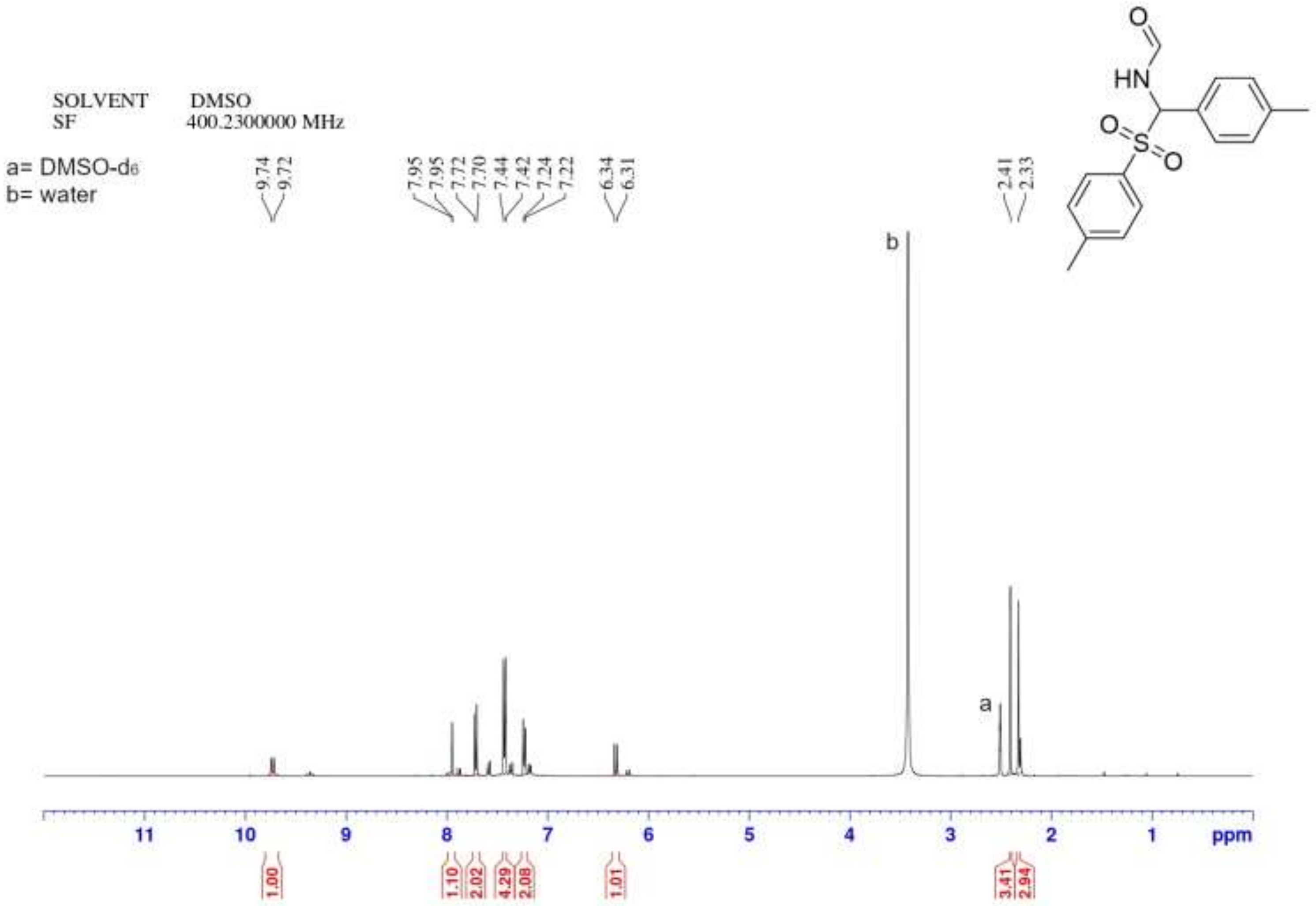

Figure S5. ^{1}H-NMR of compound 4b, Compound 4b exists as a mixture of amide rotamers.

^{1}H-NMR *N*-((4-Methoxyphenyl)(tosyl)methyl)formamide (4c):

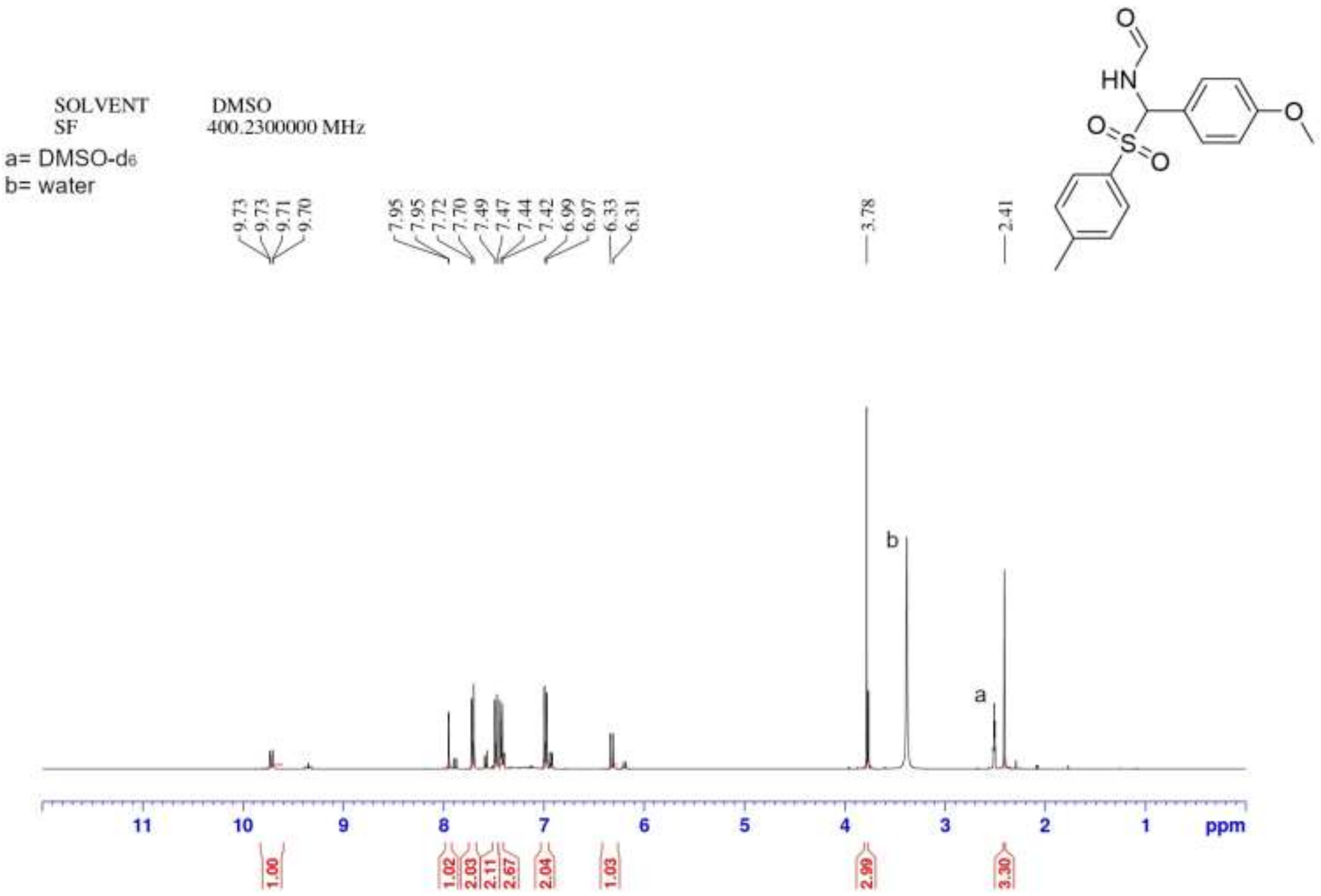

Figure S6. ^{1}H-NMR of compound 4c, Compound 4c exists as a mixture of amide rotamers.

^{1}H-NMR 1-((Isocyano(phenyl)methyl)sulfonyl)-4-methylbenzene (5a):

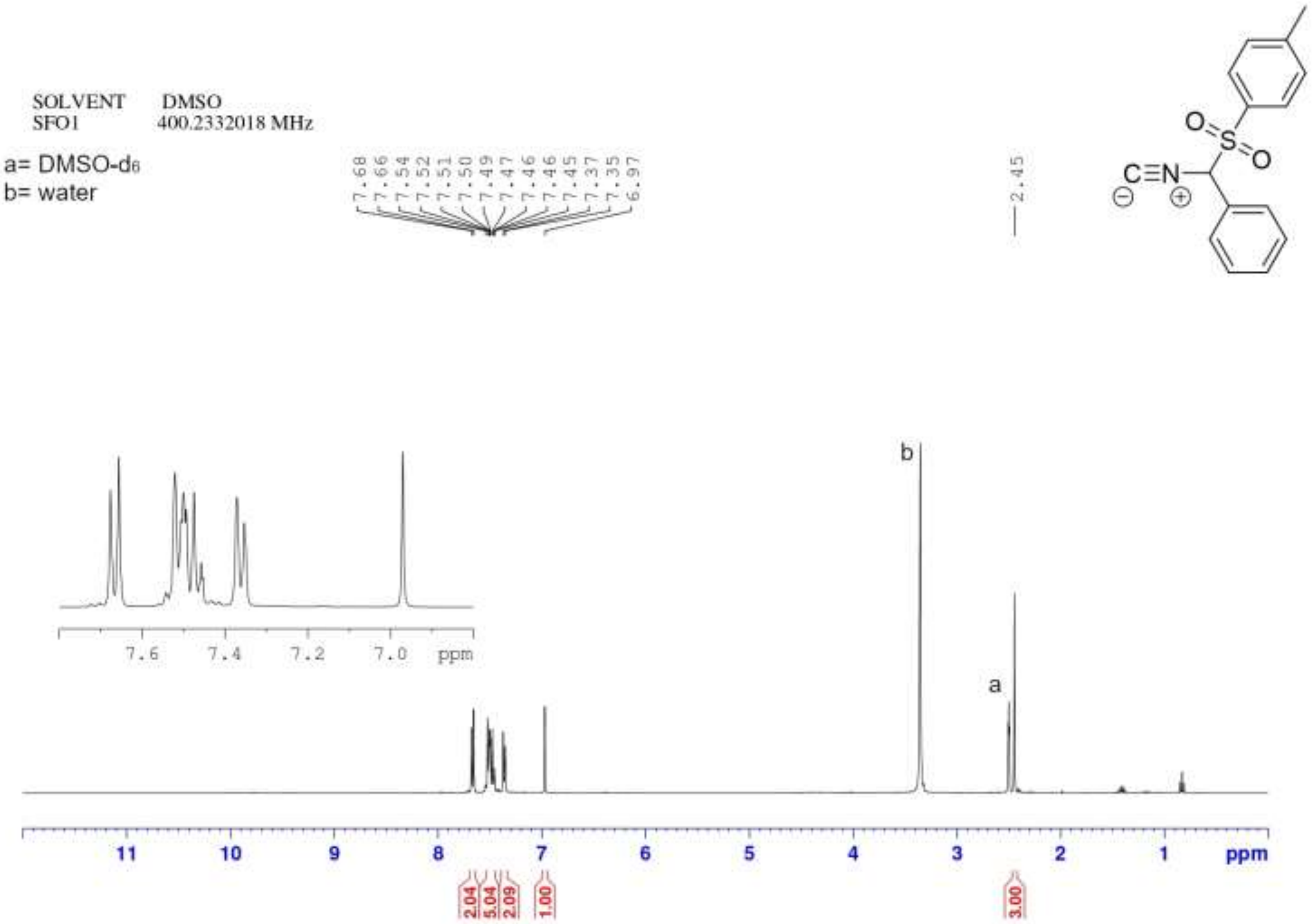

Figure S7. ^{1}H-NMR of compound 5a

^{1}H-NMR 1-((Isocyano(p-tolyl)methyl)sulfonyl)-4-methylbenzene (5b):

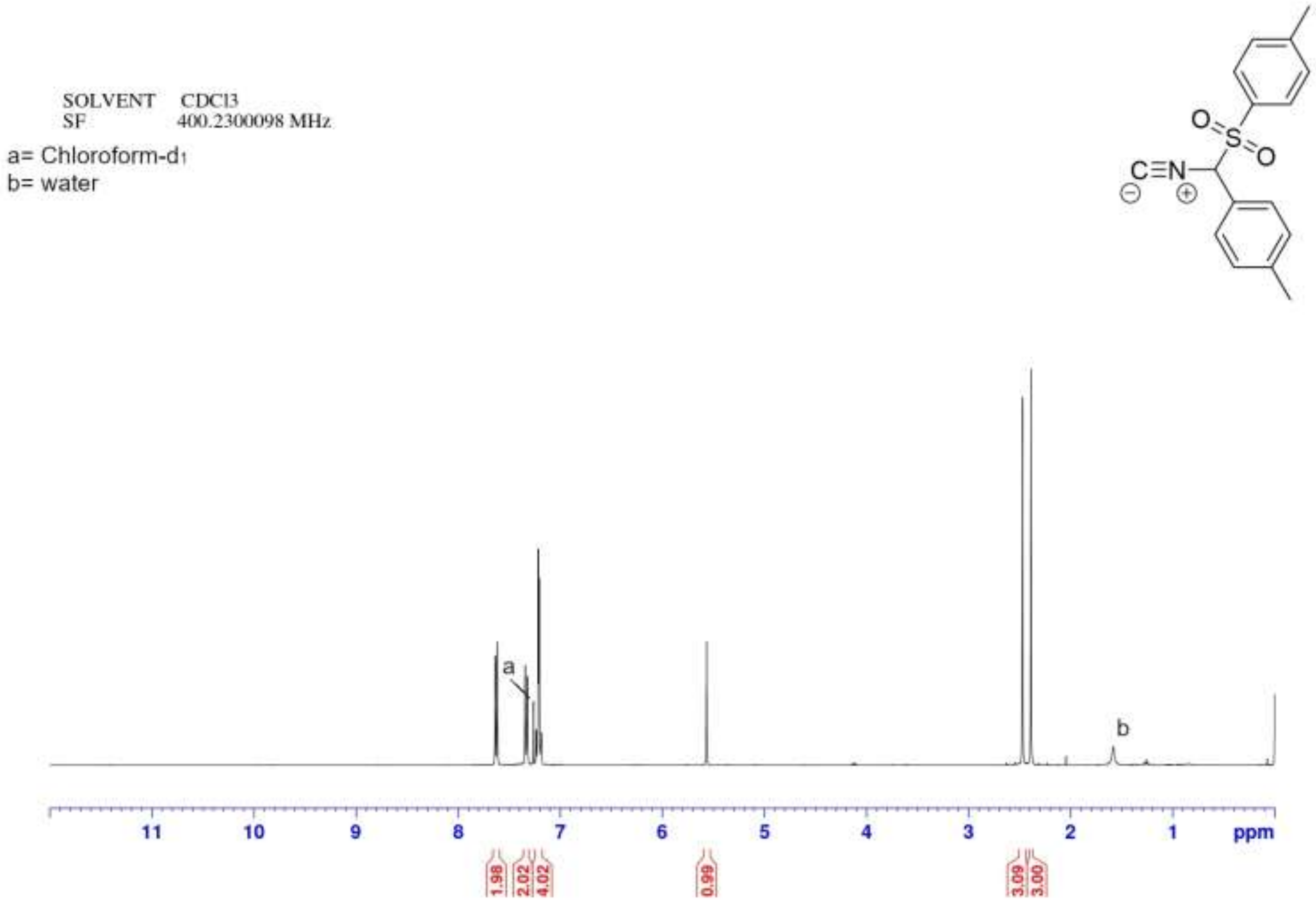

Figure S8. ^{1}H-NMR of compound 5b

^{1}H-NMR 1-((Isocyano(4-methoxyphenyl)methyl)sulfonyl)-4-methylbenzene (5c):

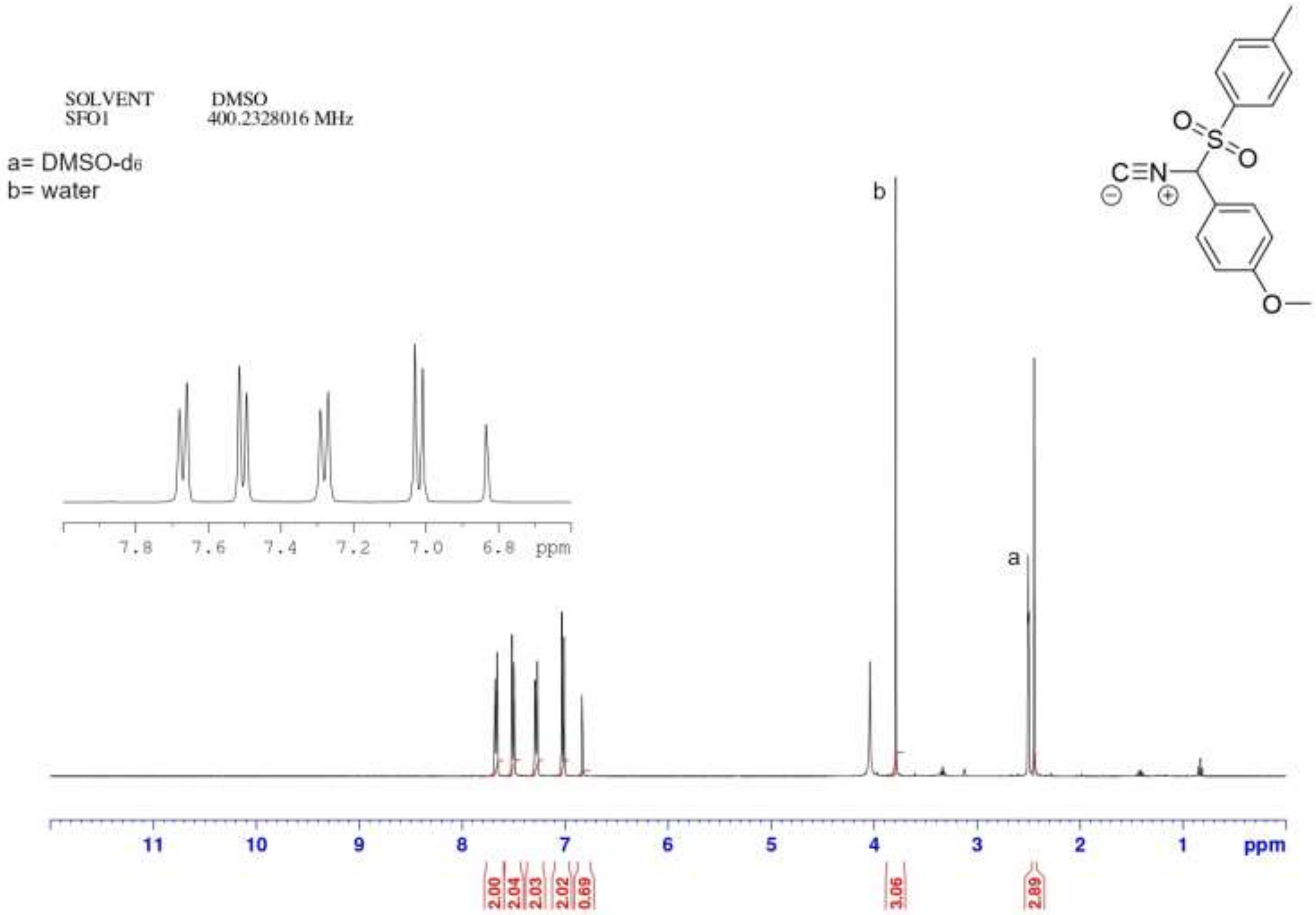

Figure S9. ^{1}H-NMR of compound 5c

^{1}H-NMR 3-Methyl-4-(1H-pyrrol-2-yl)-1H-imidazole (6a):

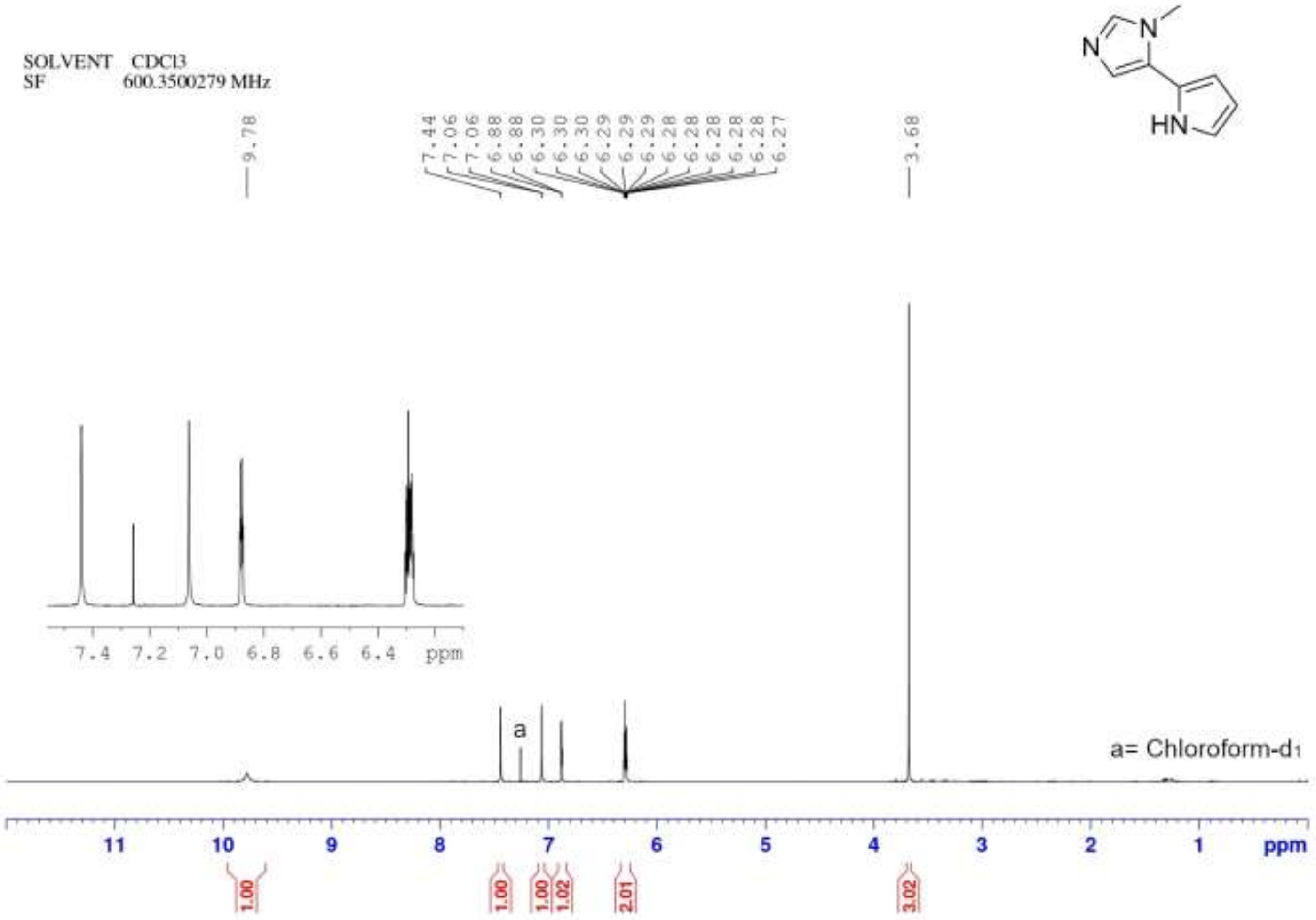

Figure S10. ^{1}H-NMR of compound 6a

^{1}H-NMR 3-(*tert*-Butyl)-4-(1H-pyrrol-2-yl)-1H-imidazole (6b):

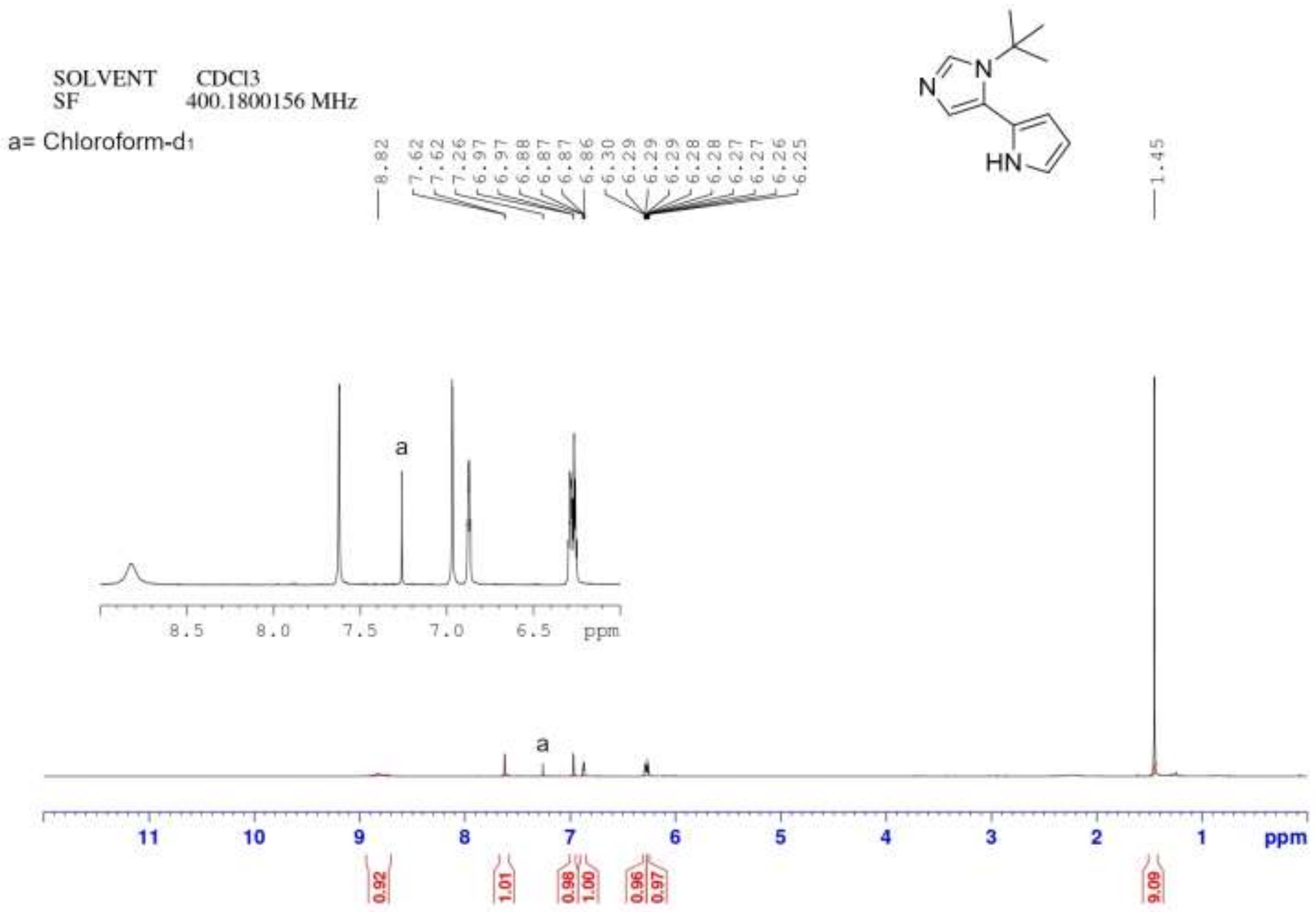

Figure S11. ^{1}H-NMR of compound 6b

^{1}H-NMR 3-Methyl-5-phenyl-4-(1H-pyrrol-2-yl)-1H-imidazole (6c):

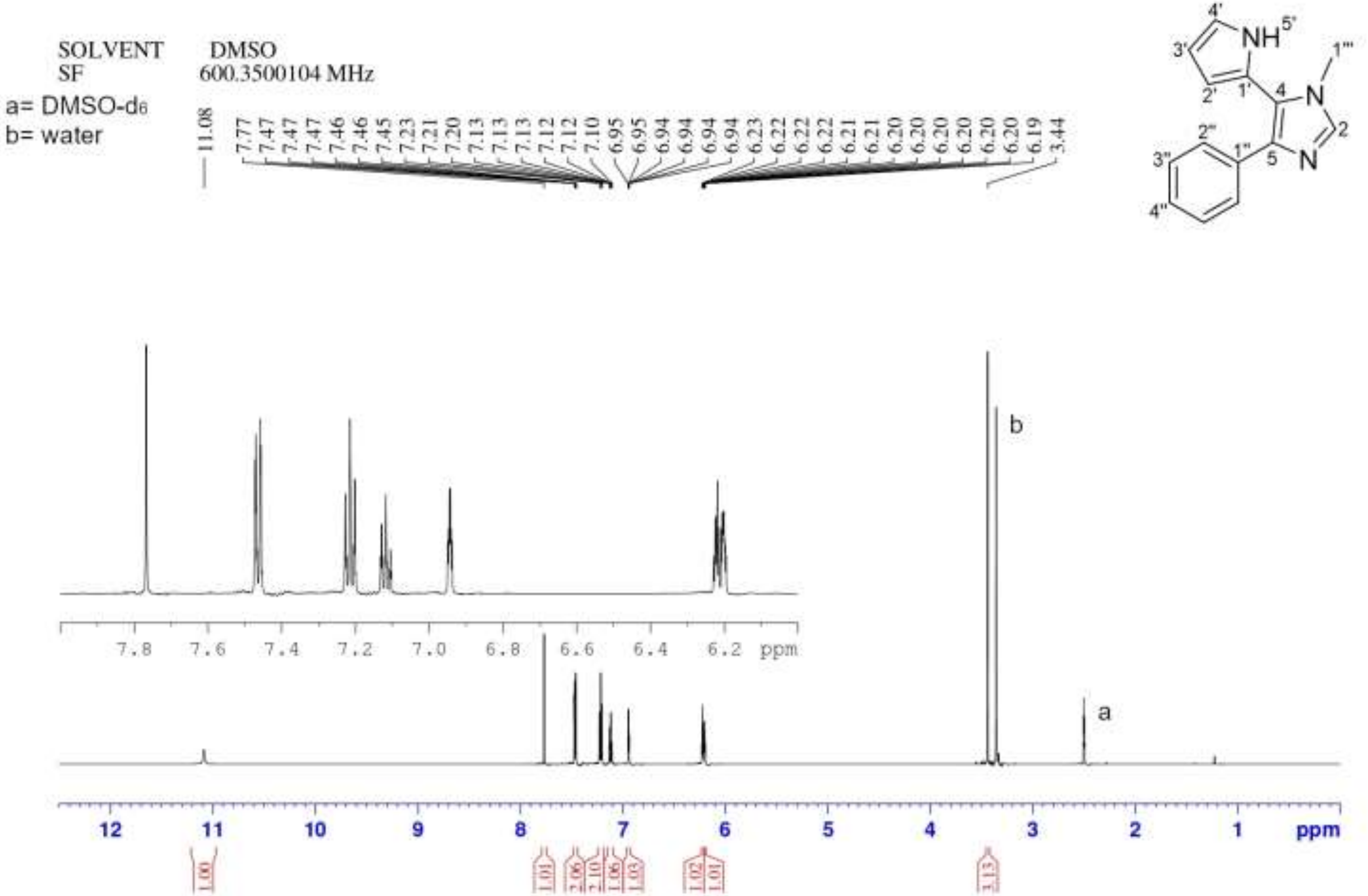

Figure S12. ^{1}H-NMR of compound 6c

$^{13}C\{^1H\}$-NMR 3-Methyl-5-phenyl-4-(1H-pyrrol-2-yl)-1H-imidazole (6c):

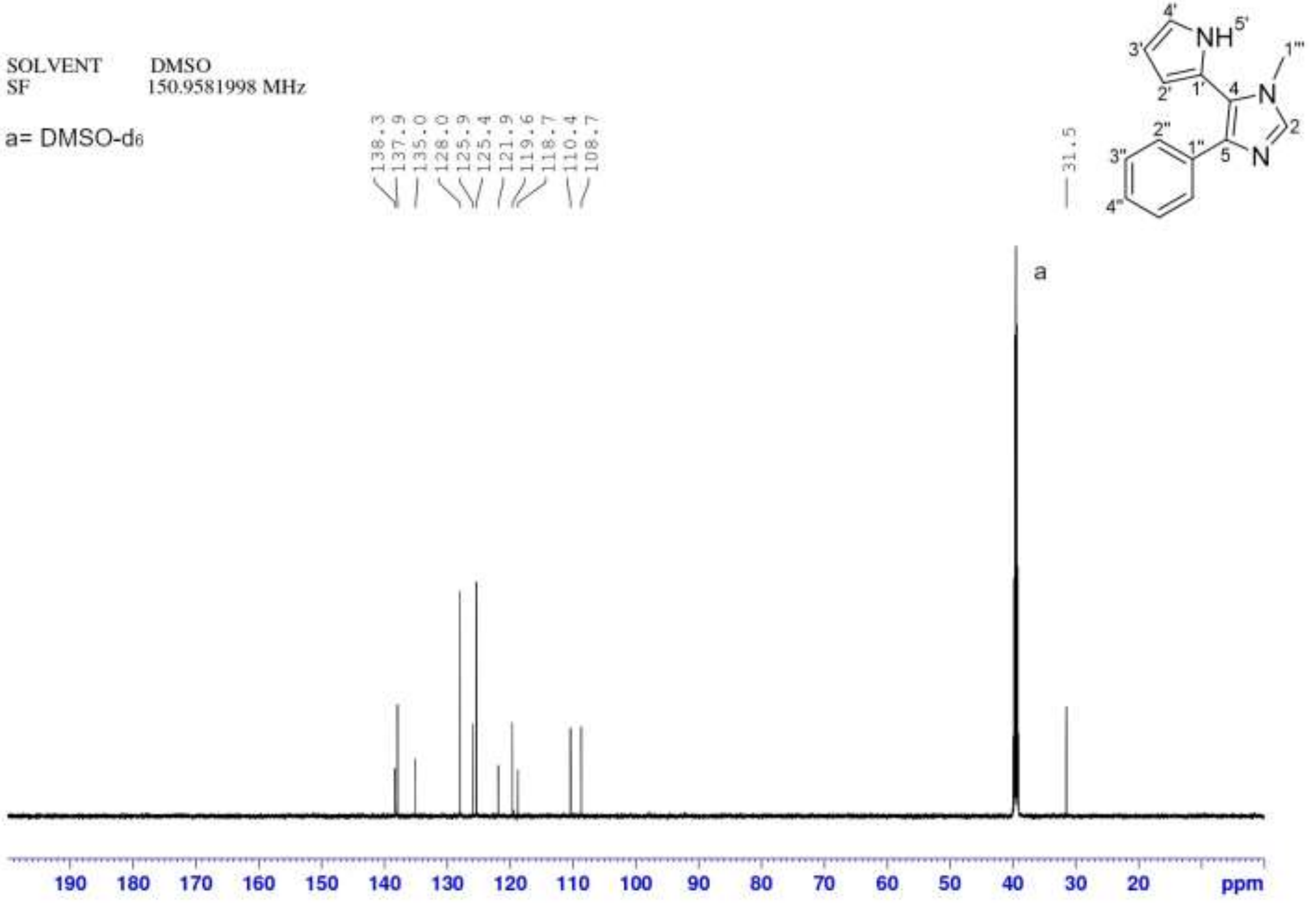

Figure S13. **$^{13}C\{^1H\}$-NMR of compound 6c**

$^{13}C\{^{1}H\}$-DEPT-NMR 3-Methyl-5-phenyl-4-(1H-pyrrol-2-yl)-1H-imidazole (6c):

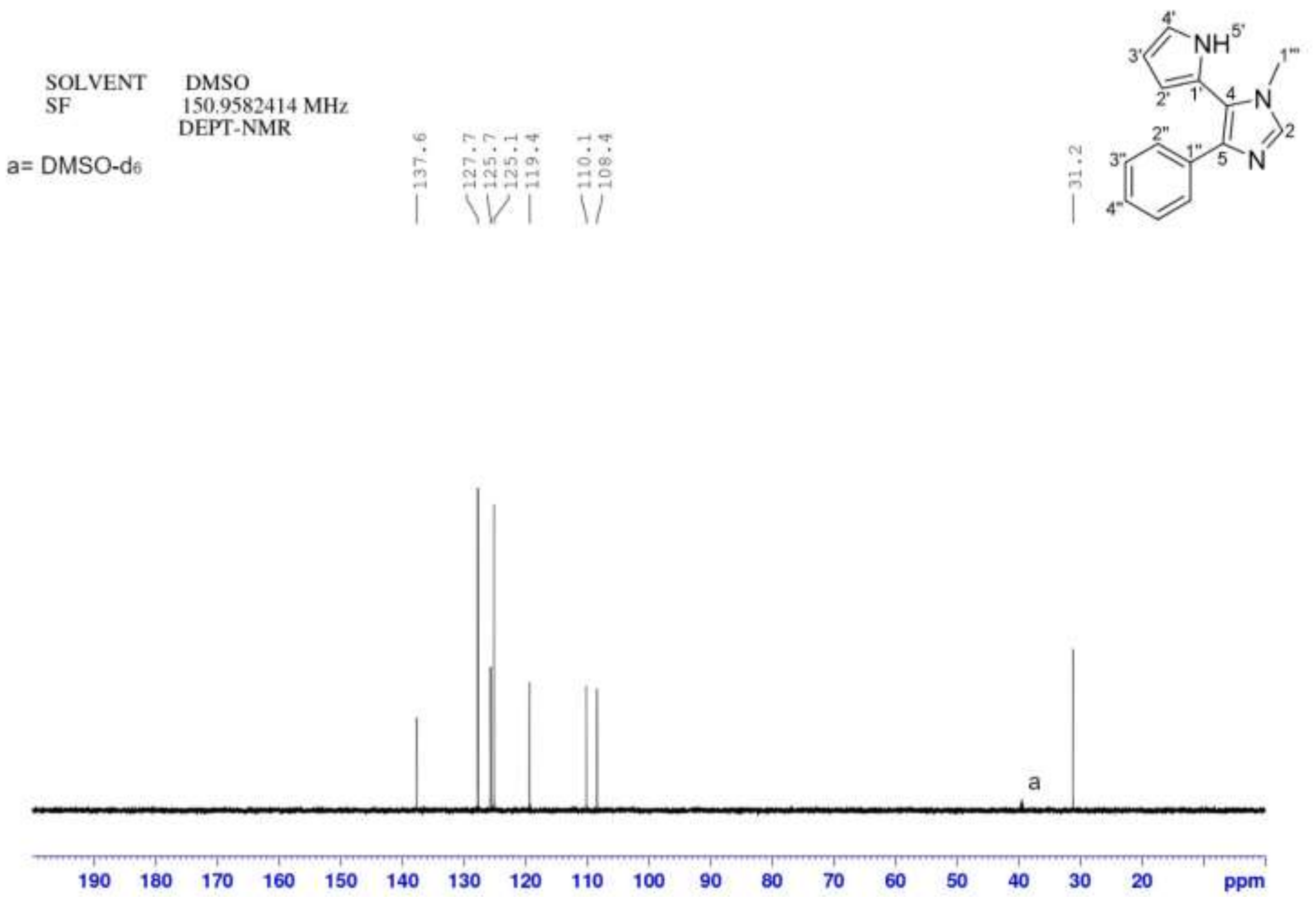

Figure S14. **$^{13}C\{^{1}H\}$-DEPT-NMR of compound 6c**

HSQC-NMR 3-Methyl-5-phenyl-4-(1H-pyrrol-2-yl)-1H-imidazole (6c):

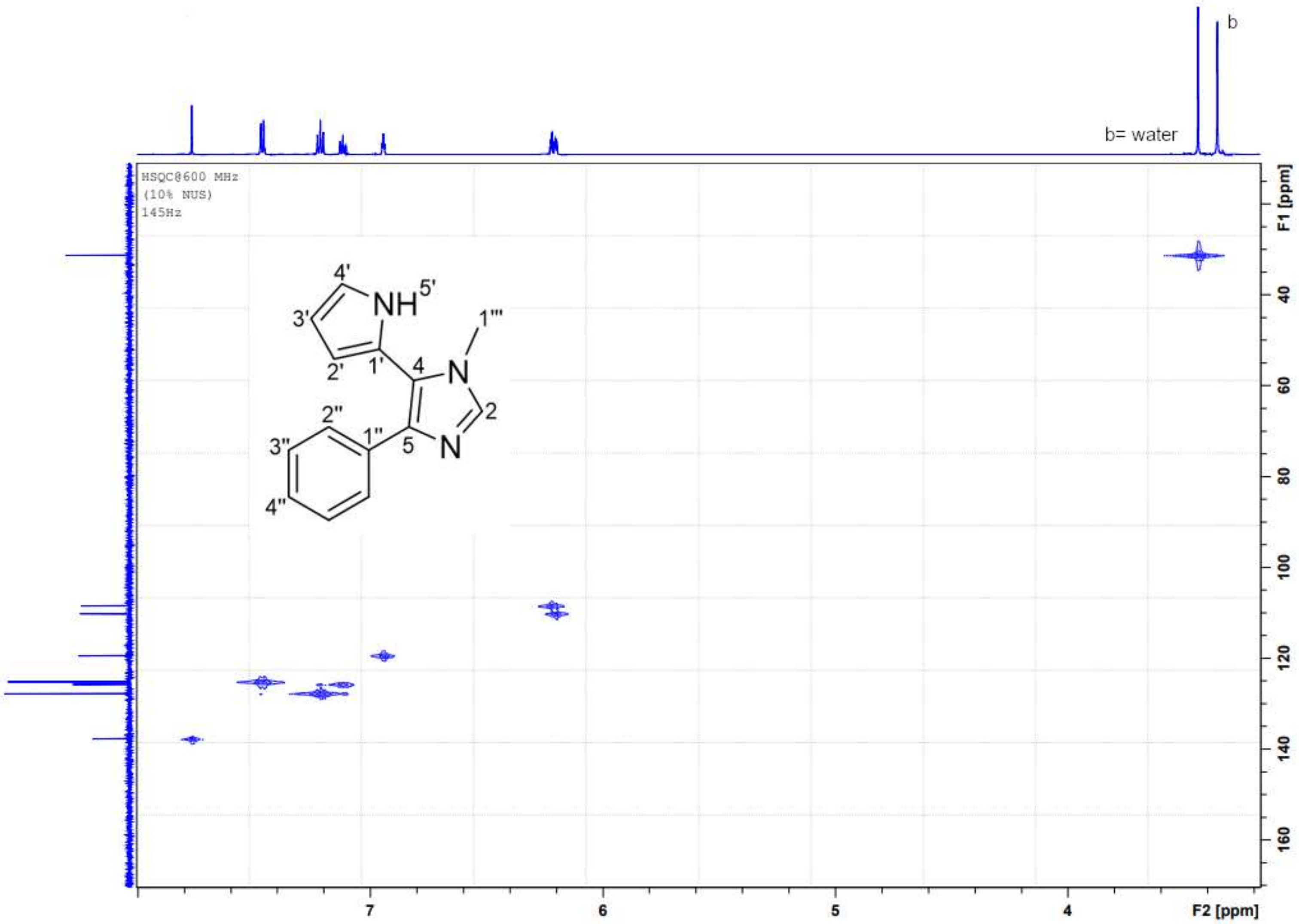

Figure S15. HSQC-NMR of compound 6c

HMBC-DEPT-NMR 3-Methyl-5-phenyl-4-(1H-pyrrol-2-yl)-1H-imidazole (6c):

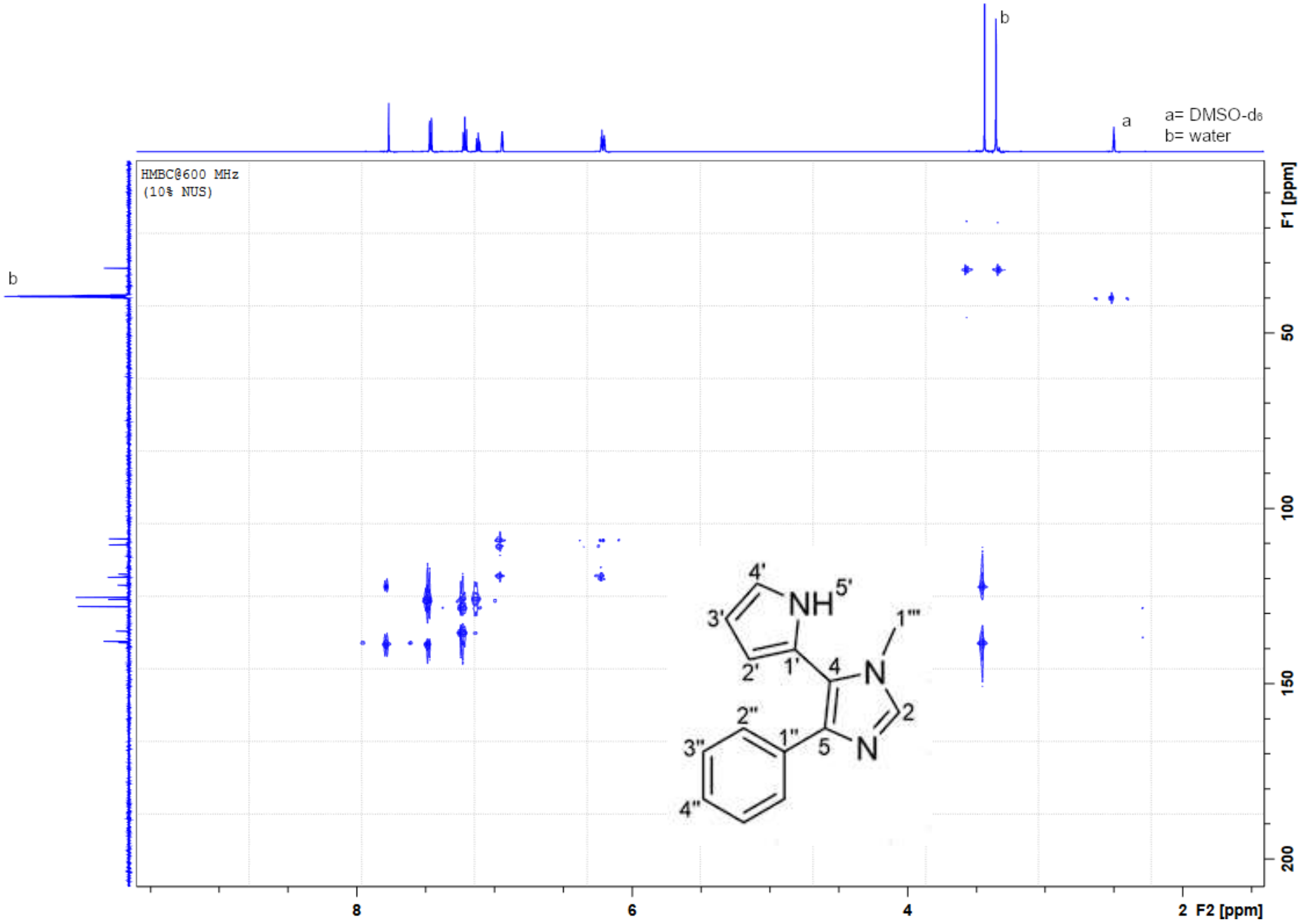

Figure S16. HMBC-NMR of compound 6c

^{1}H-NMR 3-Methyl-4-(1H-pyrrol-2-yl)-5-(p-tolyl)-1H-imidazol (6d):

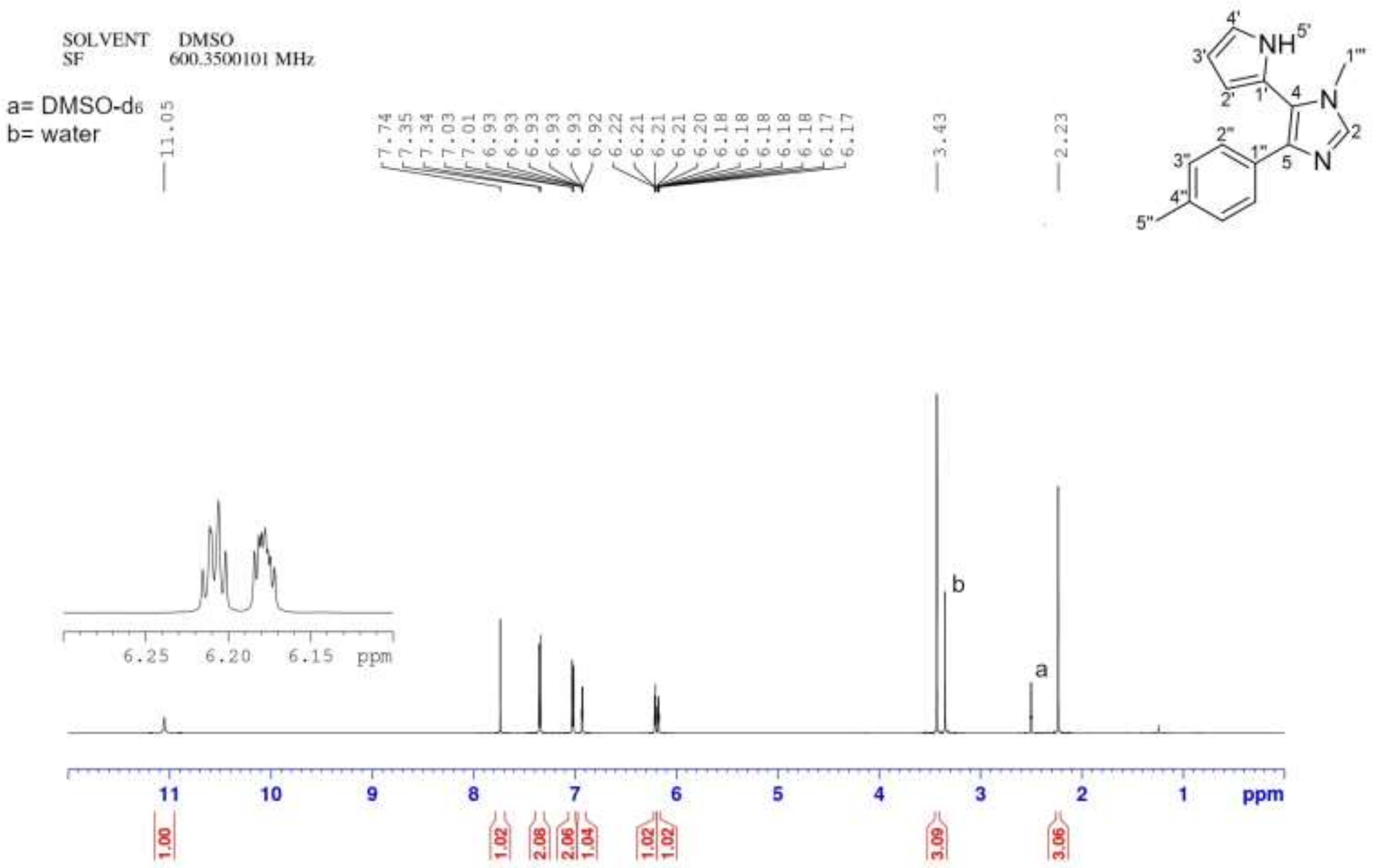

Figure S17. ^{1}H-NMR of compound 6d

$^{13}C\{^1H\}$-NMR 3-Methyl-4-(1H-pyrrol-2-yl)-5-(p-tolyl)-1H-imidazol (6d):

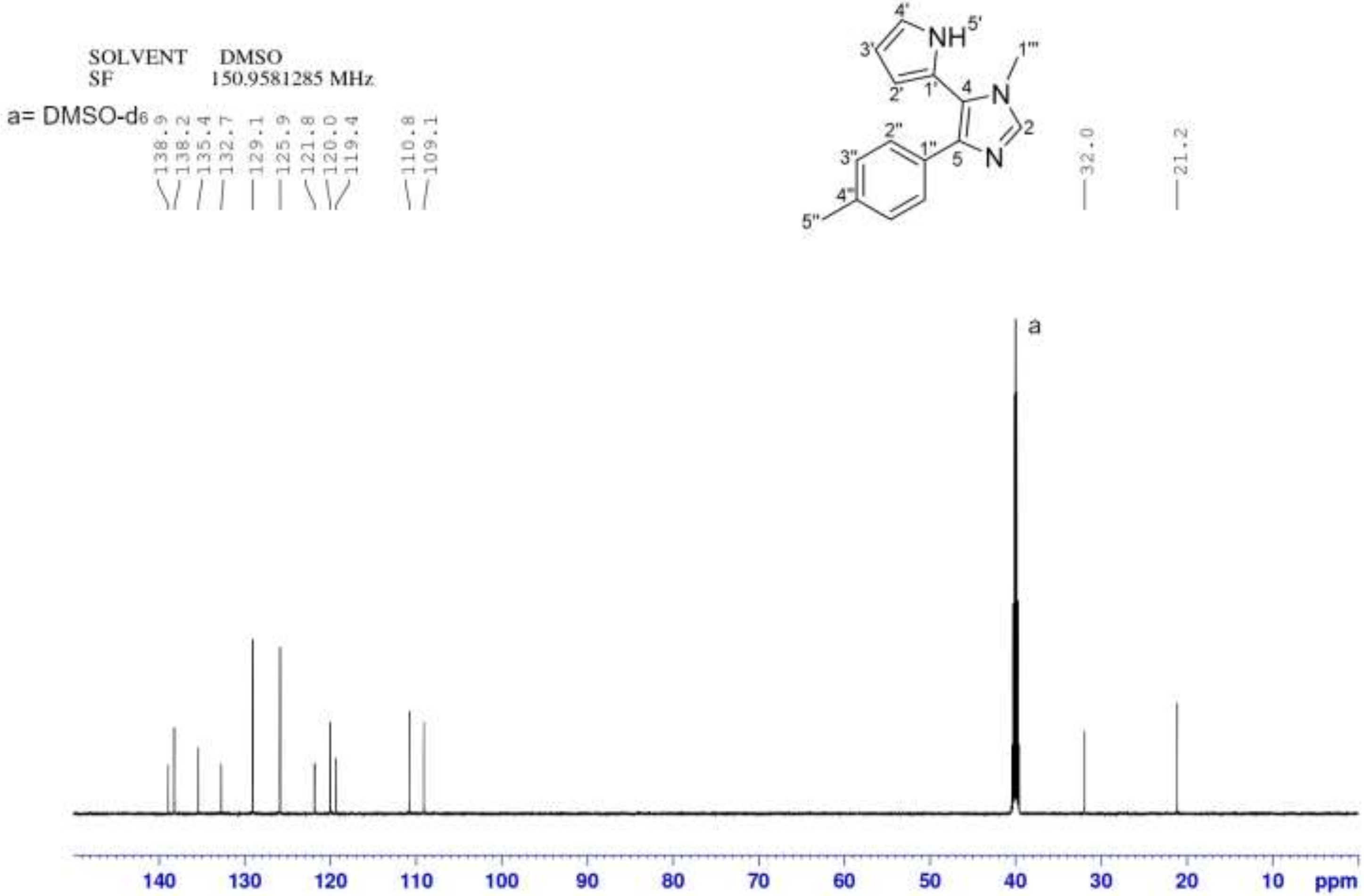

Figure S18. **$^{13}C\{^1H\}$-NMR of compound 6d**

$^{13}C\{^1H\}$-DEPT-NMR 3-Methyl-4-(1H-pyrrol-2-yl)-5-(p-tolyl)-1H-imidazol (6d):

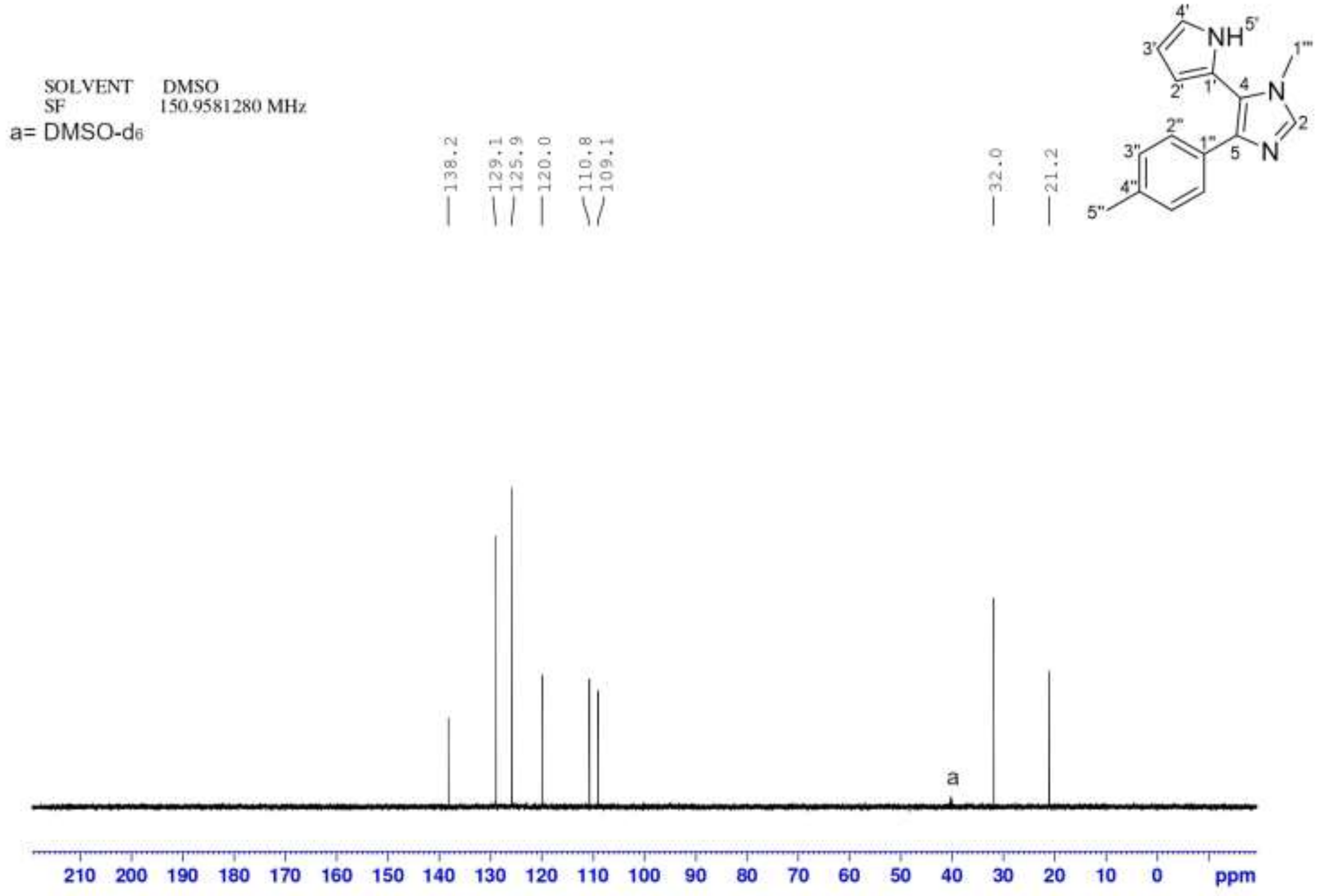

Figure S19. $^{13}C\{^1H\}$-DEPT-NMR of compound 6d

HSQC-NMR 3-Methyl-4-(1H-pyrrol-2-yl)-5-(p-tolyl)-1H-imidazol (6d):

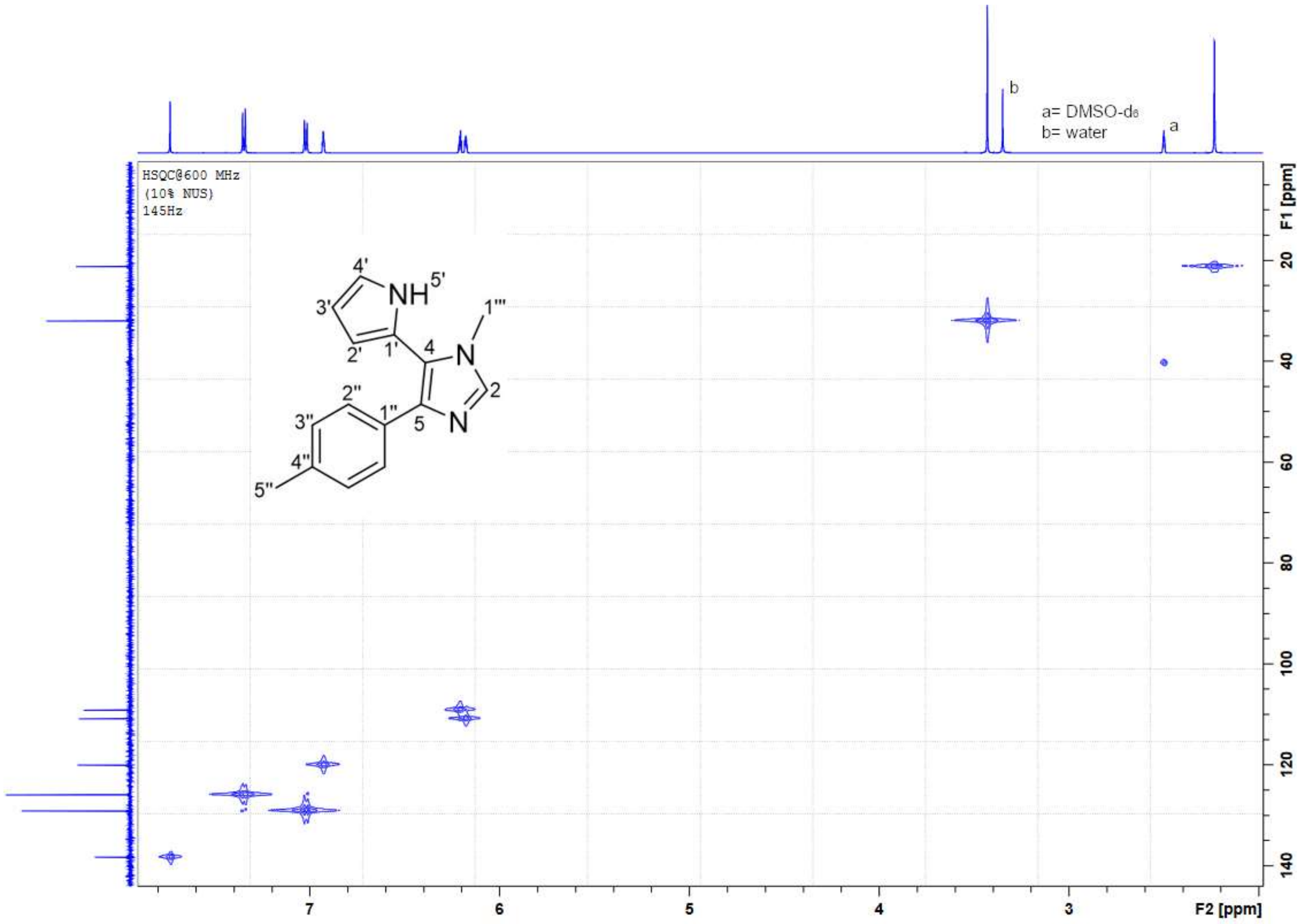

Figure S20. HSQC-NMR of compound 6d

HMBC-NMR 3-Methyl-4-(1H-pyrrol-2-yl)-5-(p-tolyl)-1H-imidazol (6d):

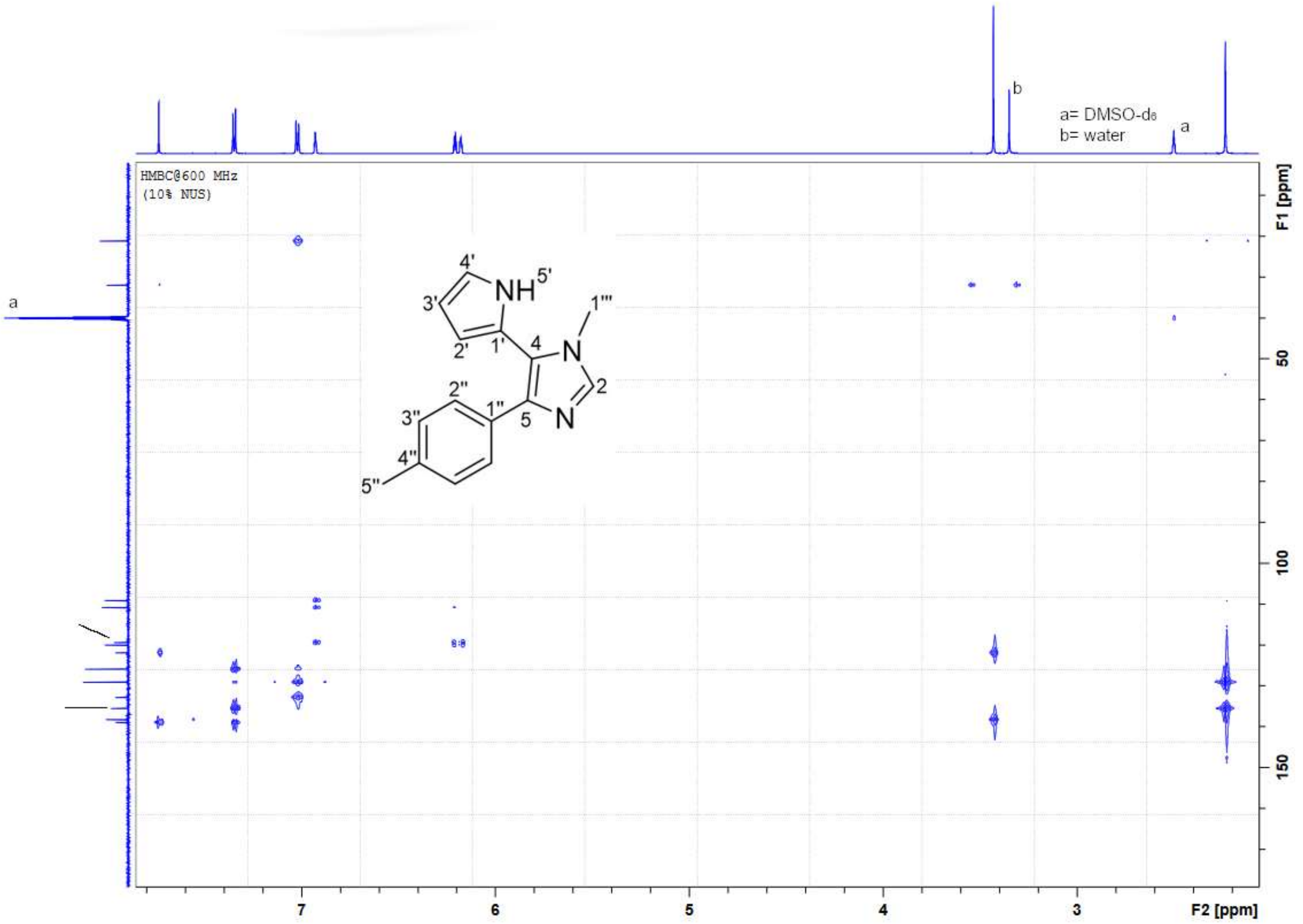

Figure S21. HMBC-NMR of compound 6d

^{1}H-NMR 5-(4-Methoxyphenyl)-3-methyl-4-(1H-pyrrol-2-yl)-1H-imidazole (6e):

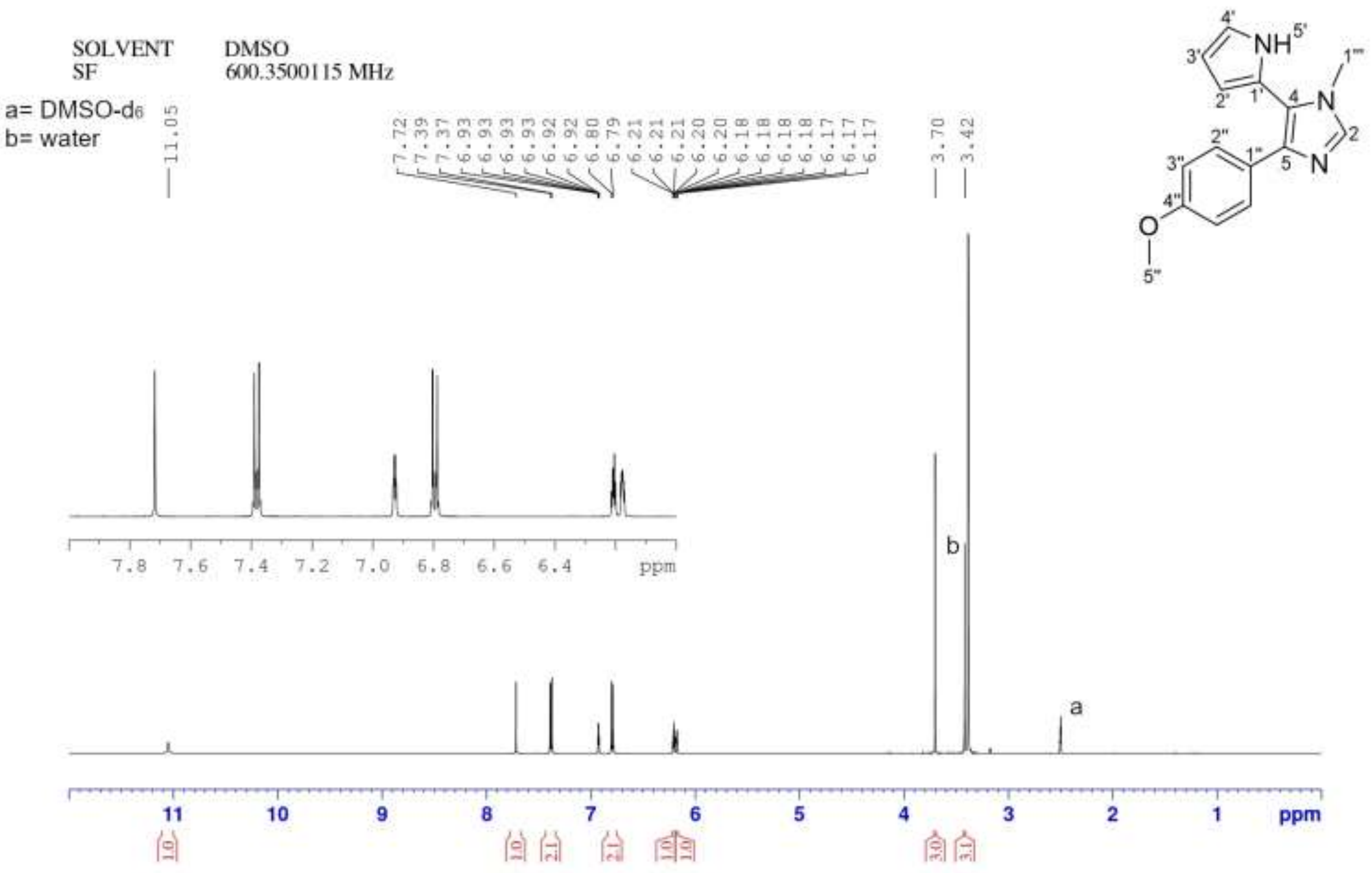

Figure S22. **^{1}H-NMR of compound 6e**

$^{13}C\{^{1}H\}$-NMR 5-(4-Methoxyphenyl)-3-methyl-4-(1H-pyrrol-2-yl)-1H-imidazole (6e):

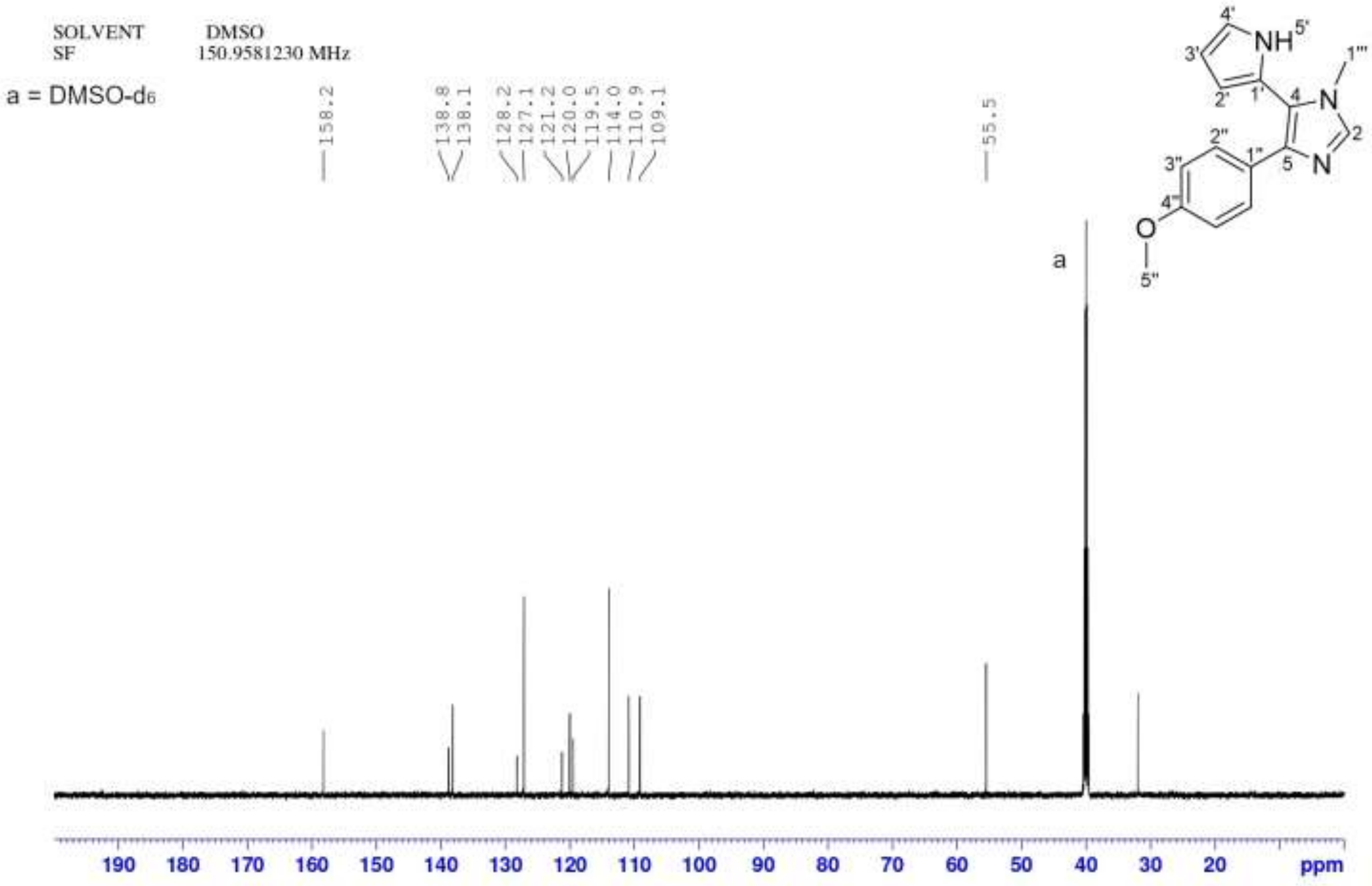

Figure S23. **$^{13}C\{^{1}H\}$-NMR of compound 6e**

$^{13}C\{^{1}H\}$-DEPT-NMR 5-(4-Methoxyphenyl)-3-methyl-4-(1H-pyrrol-2-yl)-1H-imidazole (6e):

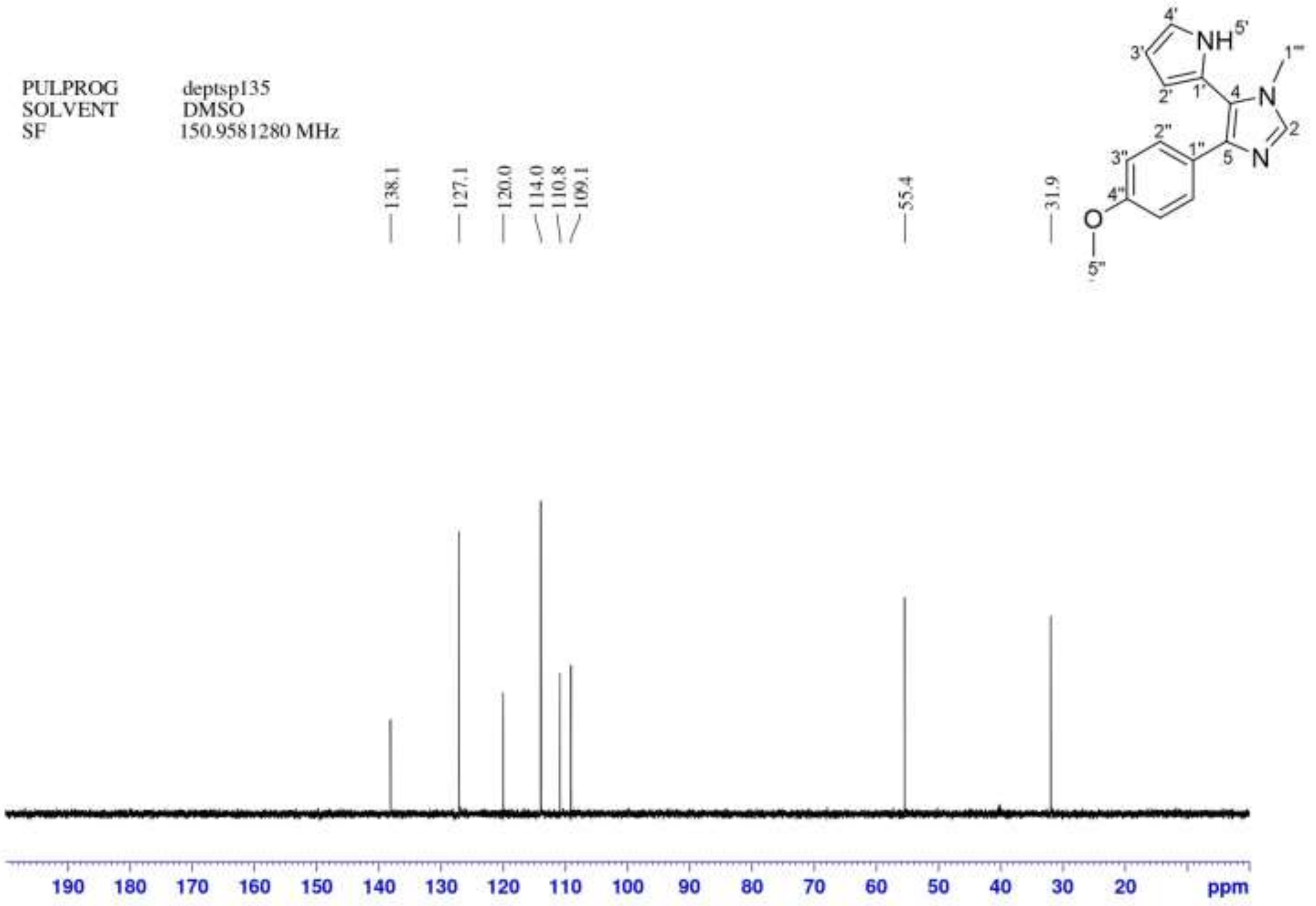

Figure S24. $^{13}C\{^{1}H\}$**-DEPT-NMR of compound 6e**

HSQC-NMR 5-(4-Methoxyphenyl)-3-methyl-4-(1H-pyrrol-2-yl)-1H-imidazole (6e):

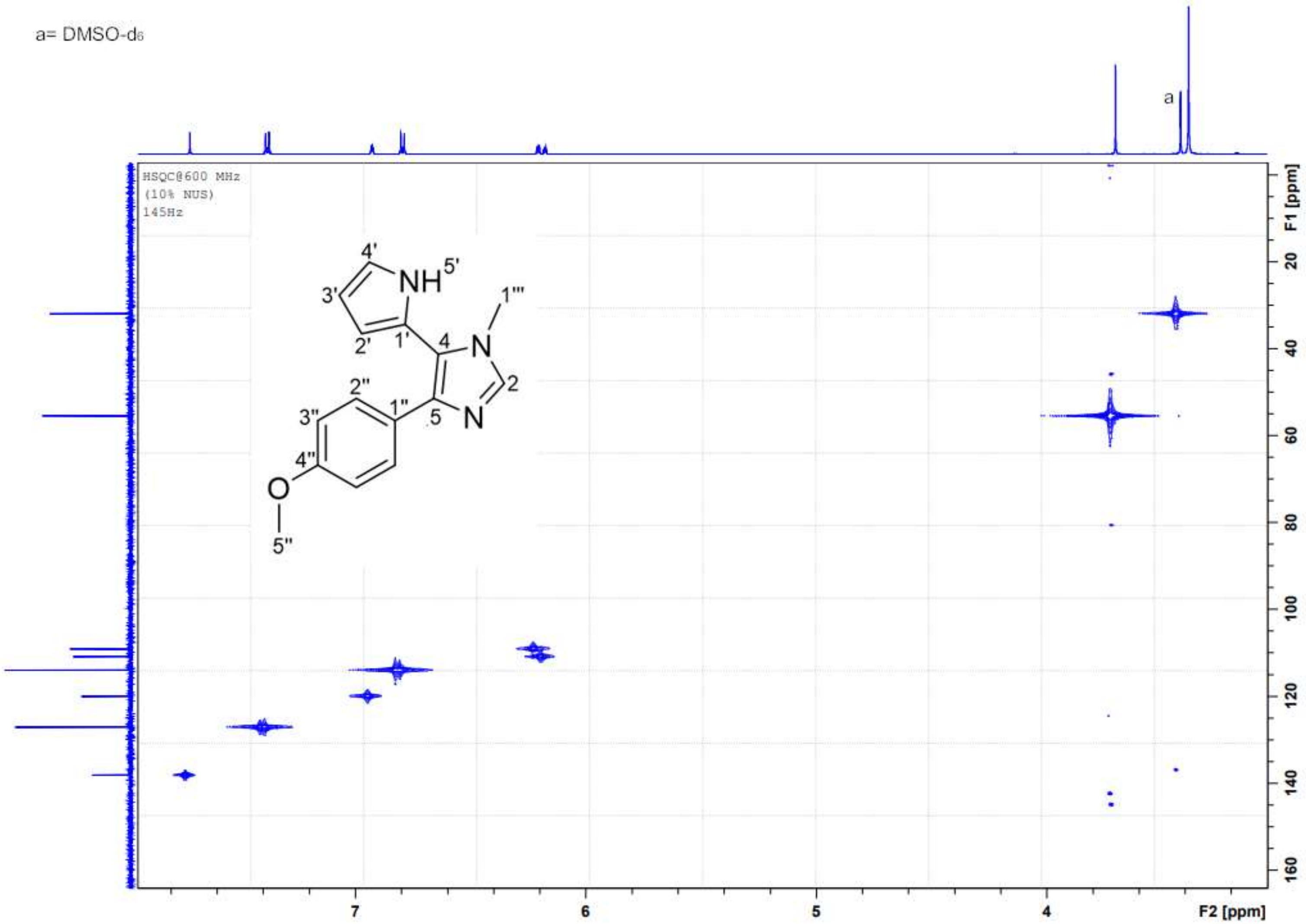

Figure S25. HSQC-NMR of compound 6e

HMBC-NMR 5-(4-Methoxyphenyl)-3-methyl-4-(1H-pyrrol-2-yl)-1H-imidazole (6e):

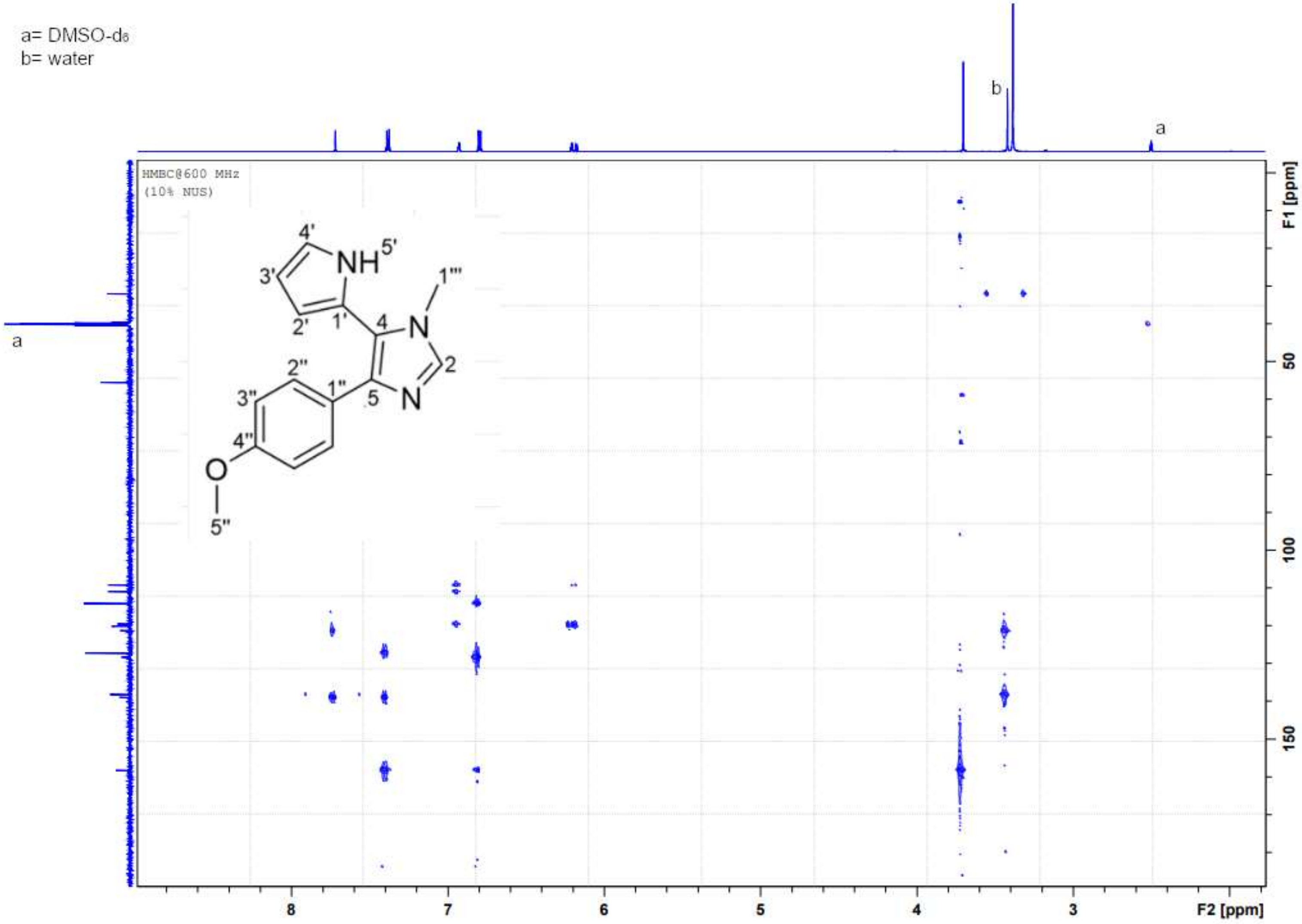

Figure S26. HMBC-NMR of compound 6e

^{1}H-NMR 1,3-Dimethyl-4-(1H-pyrrol-2-yl)-1H-imidazol-3-ium tetrafluoroborate (7a):

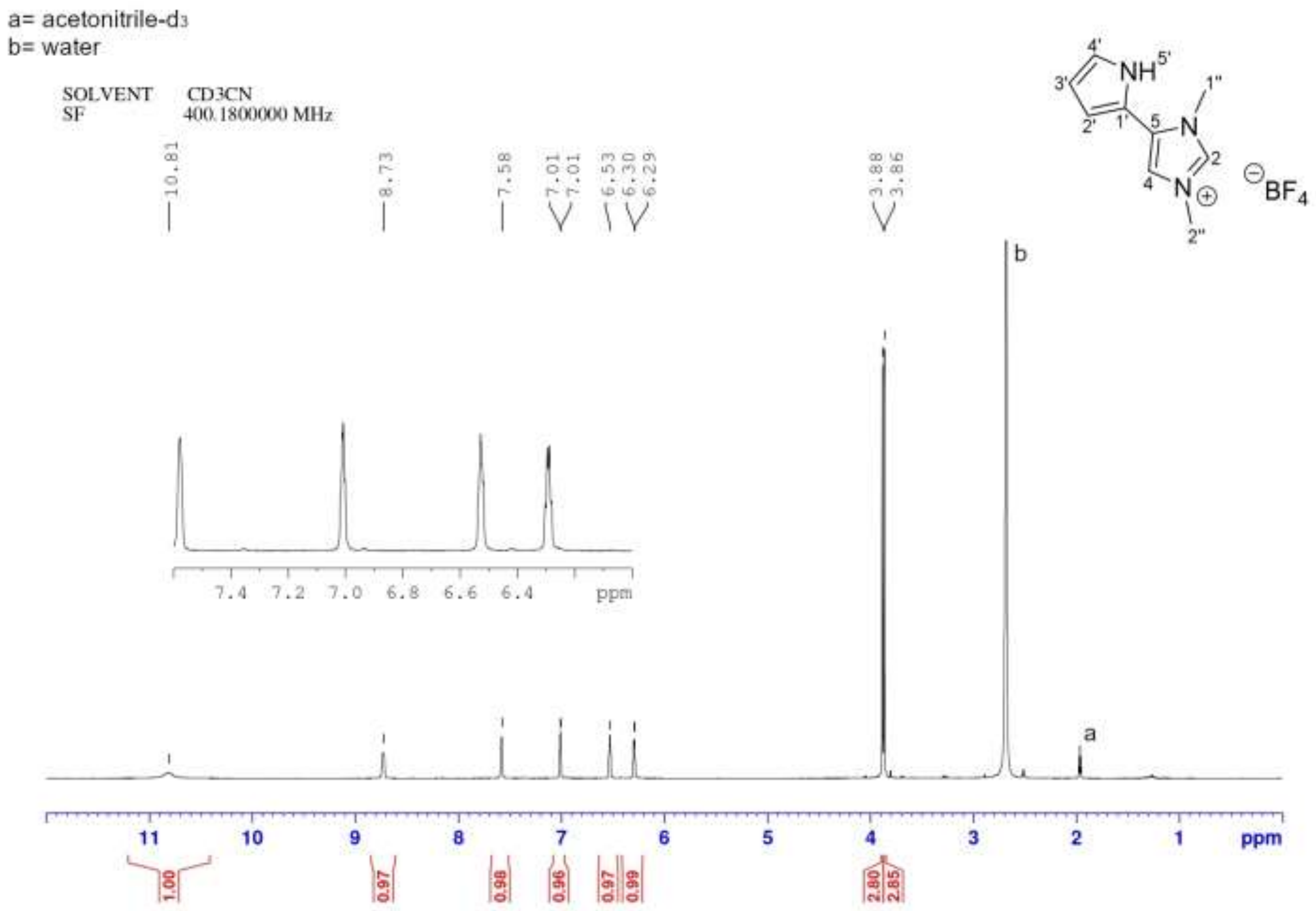

Figure S27. ^{1}H-NMR of compound 7a

$^{13}C\{^{1}H\}$-NMR 1,3-Dimethyl-4-(1H-pyrrol-2-yl)-1H-imidazol-3-ium tetrafluoroborate (7a):

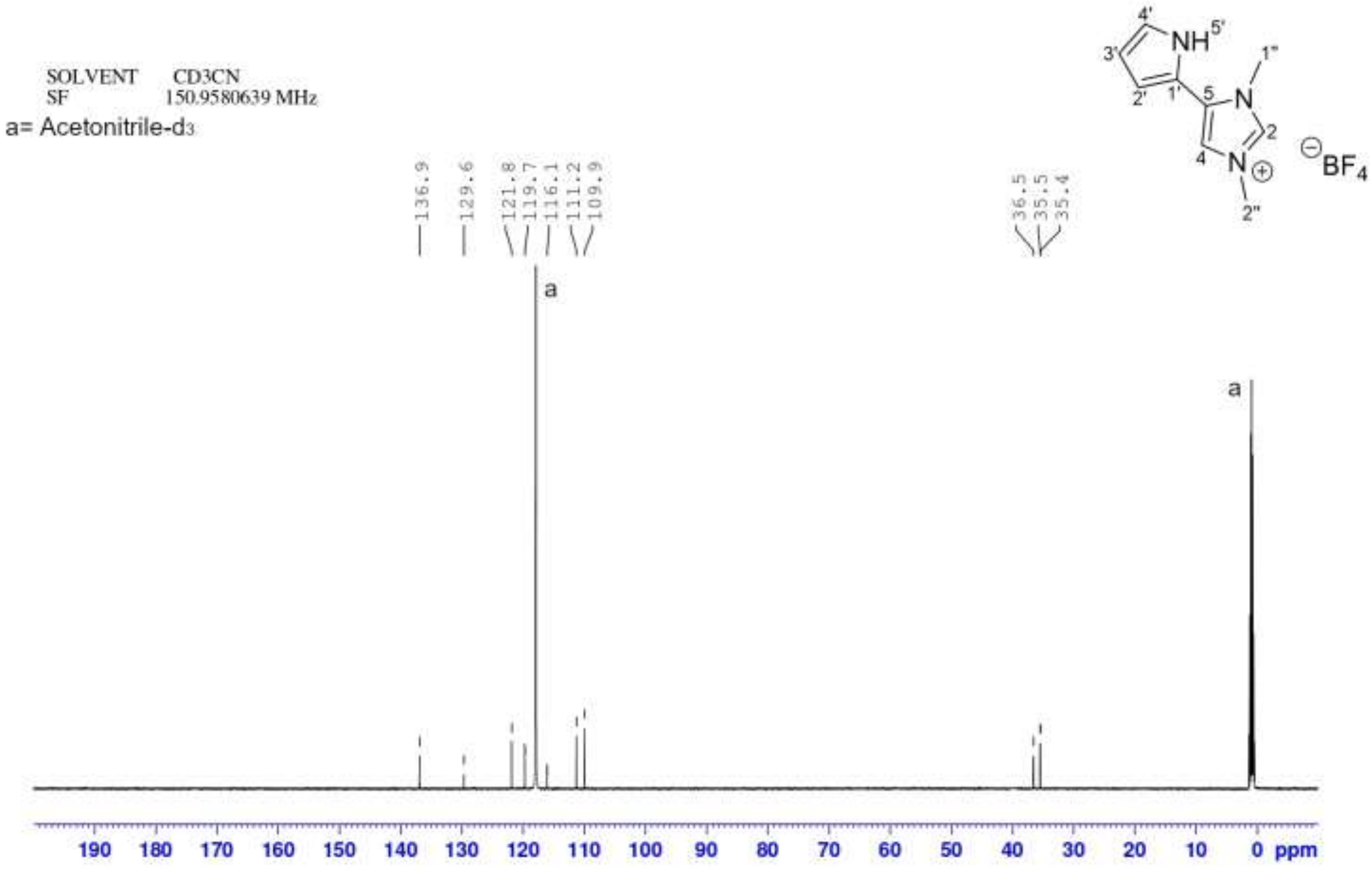

Figure S28. **$^{13}C\{^{1}H\}$-NMR of compound 7a**

$^{13}C\{^1H\}$-DEPT-NMR 1,3-Dimethyl-4-(1H-pyrrol-2-yl)-1H-imidazol-3-ium tetrafluoroborate (7a):

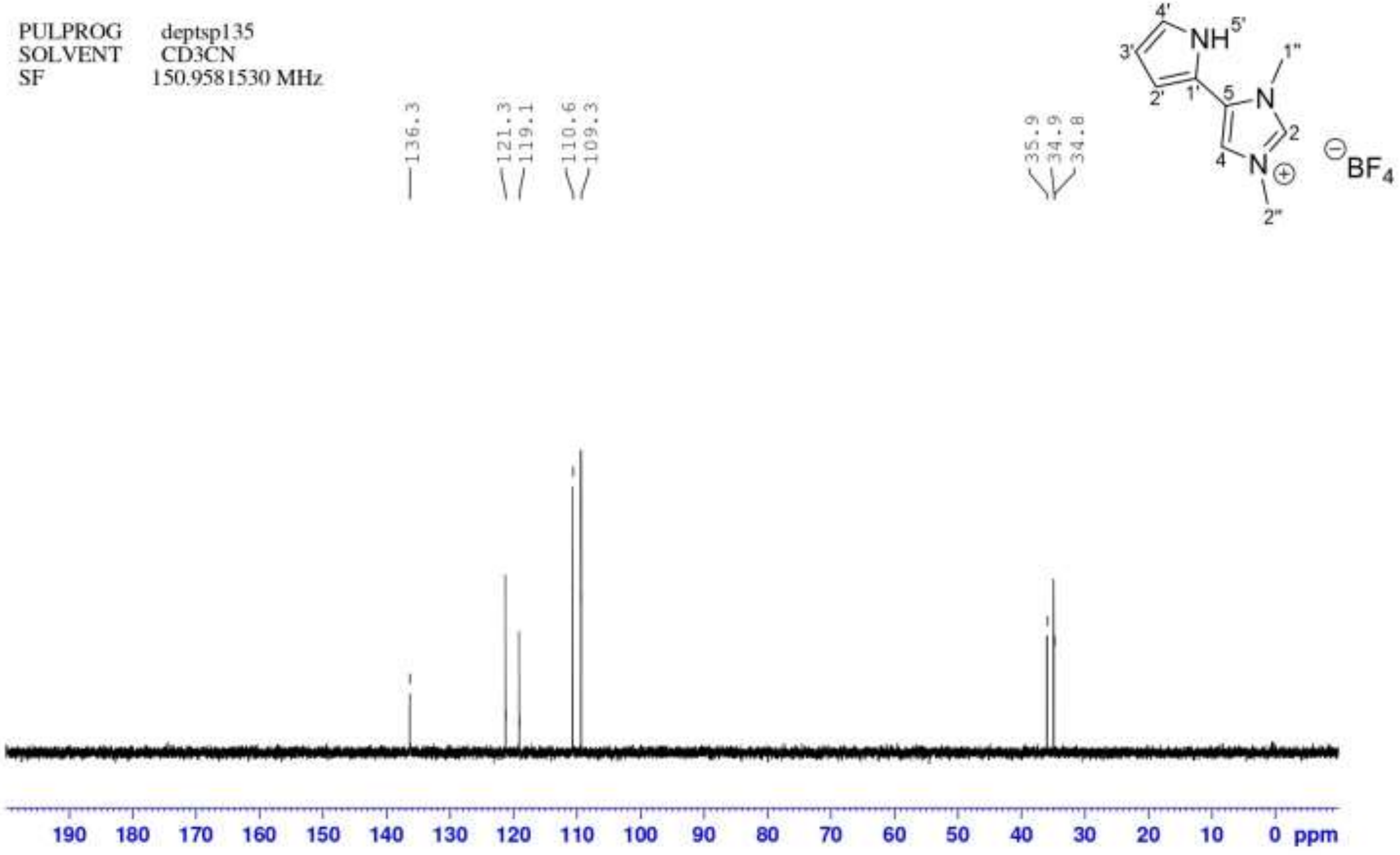

Figure S29. $^{13}C\{^1H\}$**-DEPT-NMR of compound 7a**

HSQC-NMR 1,3-Dimethyl-4-(1H-pyrrol-2-yl)-1H-imidazol-3-ium tetrafluoroborate (7a):

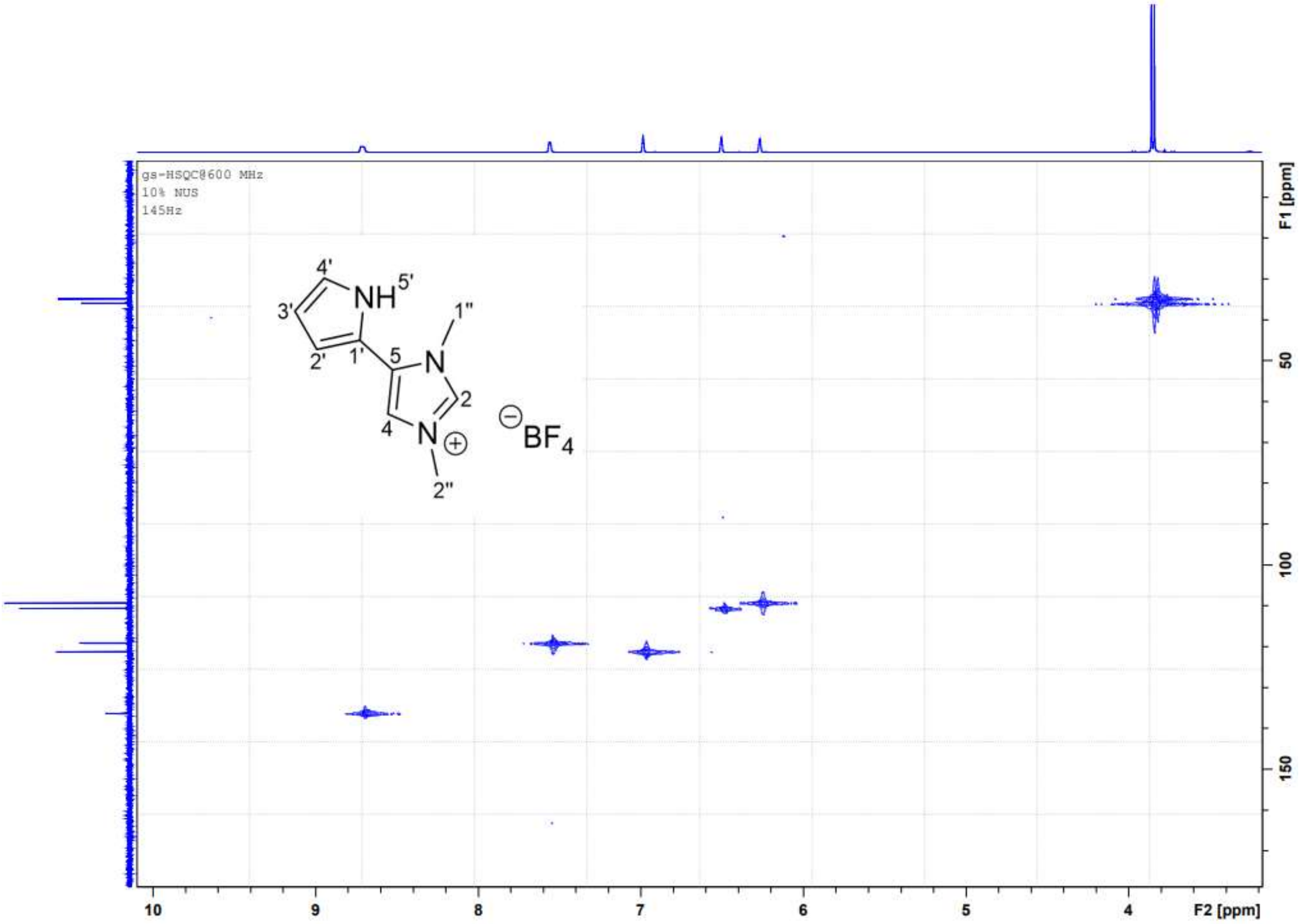

Figure S30. HSQC-NMR of compound 7a

HMBC-NMR 1,3-Dimethyl-4-(1H-pyrrol-2-yl)-1H-imidazol-3-ium tetrafluoroborate (7a):

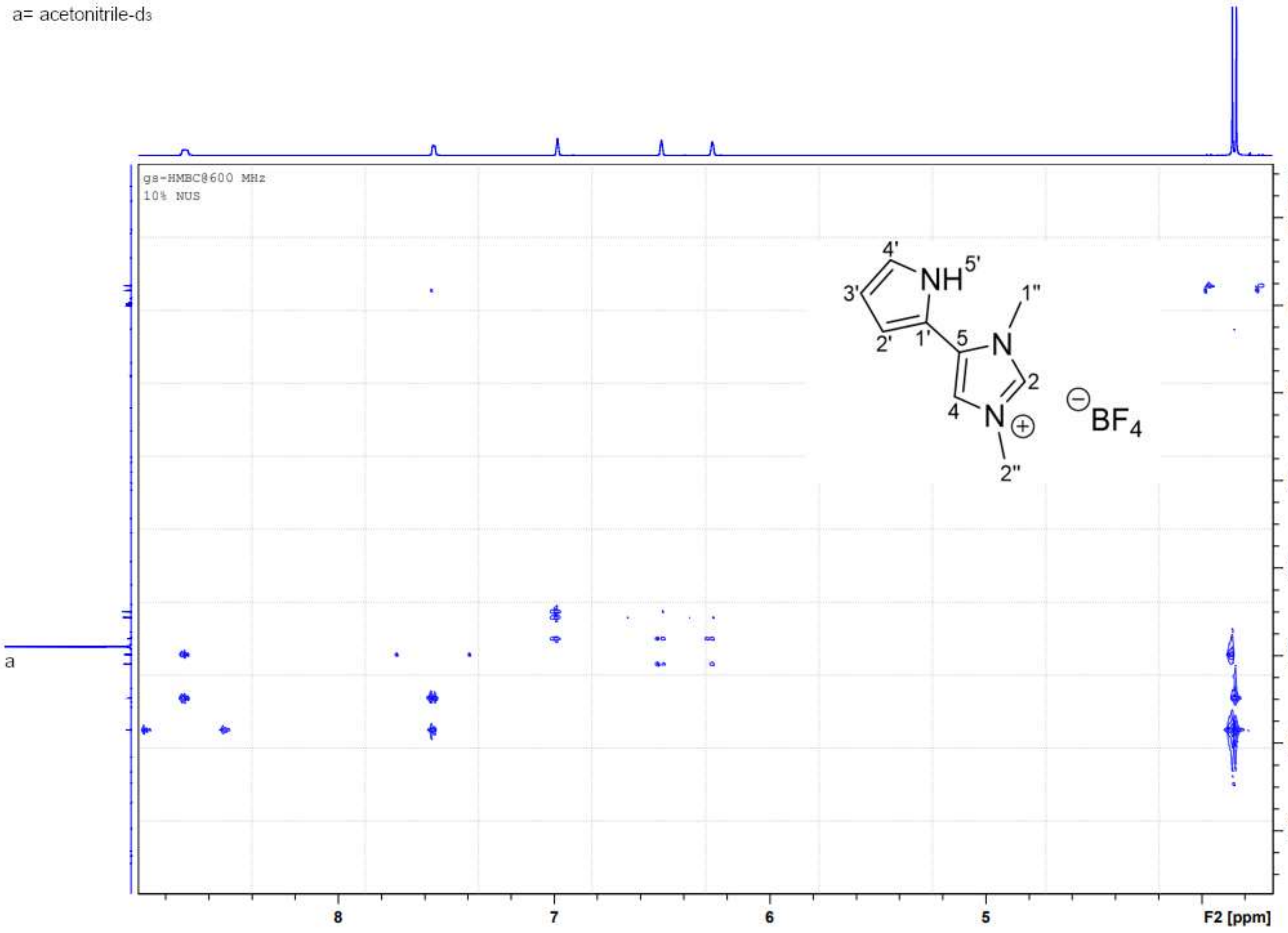

Figure S31. HMBC-NMR of compound 7a

^{1}H-NMR 1,3-Dimethyl-5-phenyl-4-(1H-pyrrol-2-yl)-1H-imidazol-3-ium iodide (7b):

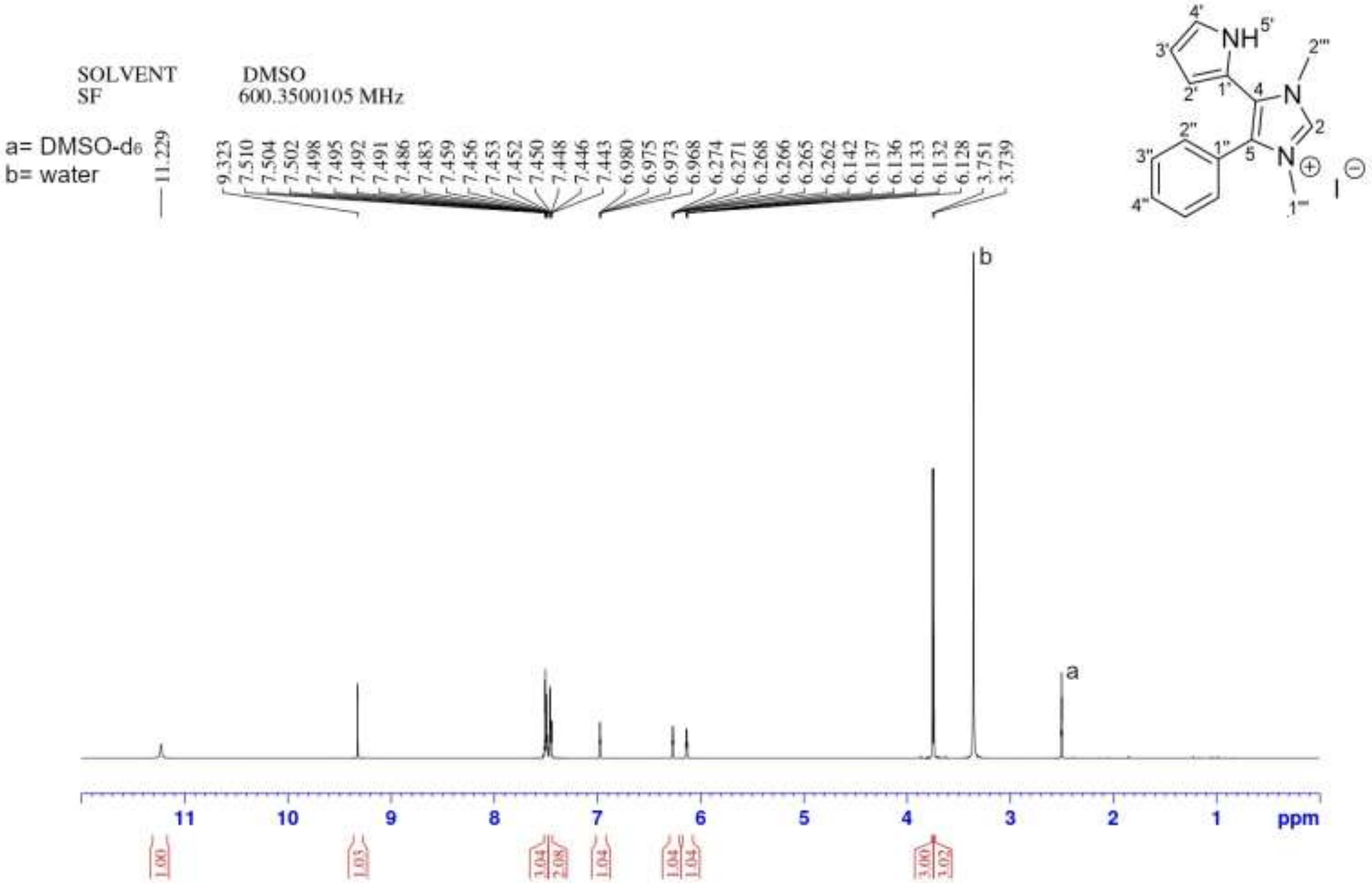

Figure S32. ^{1}H-NMR of compound 7b

$^{13}C\{^1H\}$-NMR 1,3-Dimethyl-5-phenyl-4-(1H-pyrrol-2-yl)-1H-imidazol-3-ium iodide (7b):

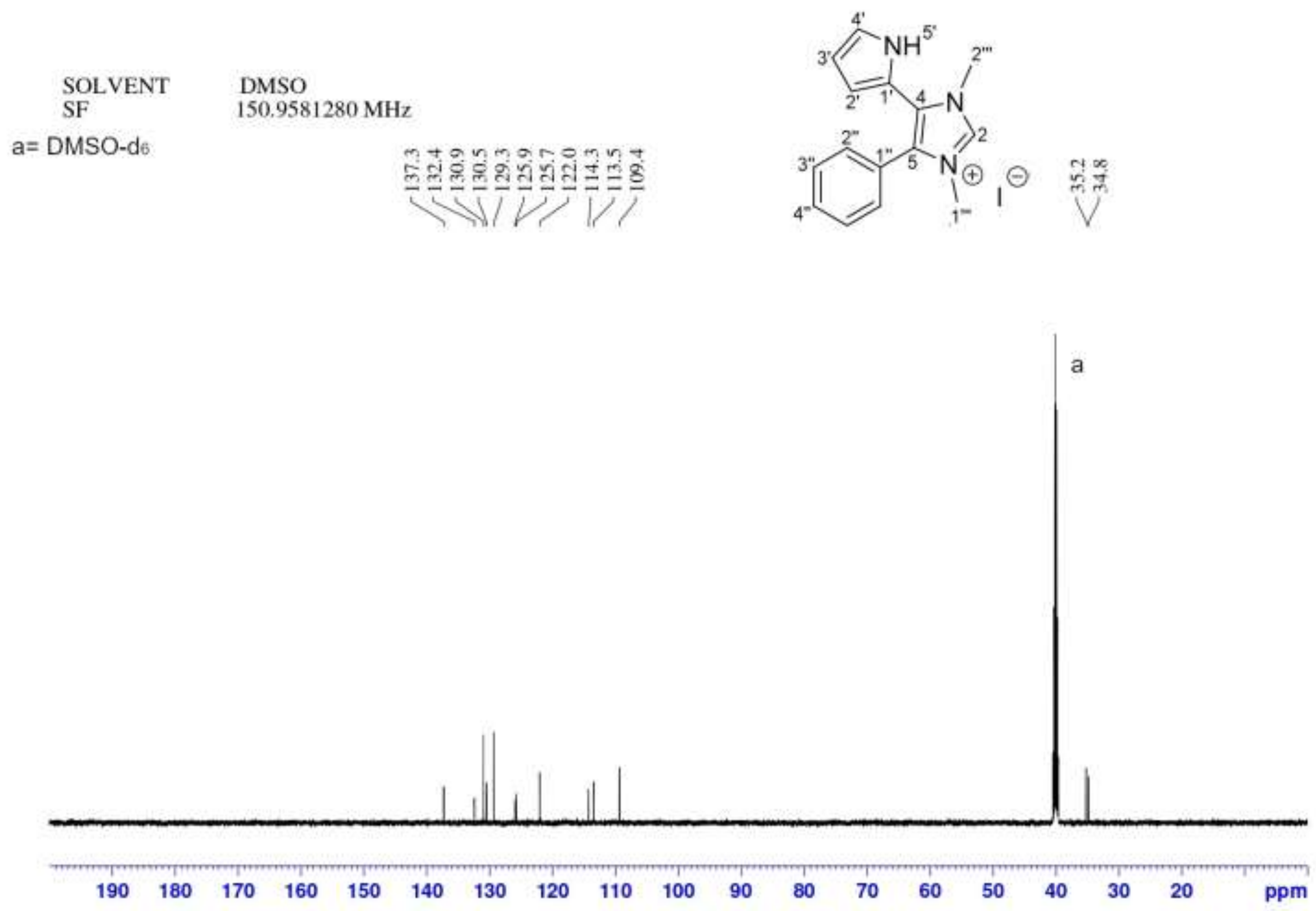

Figure S33. $^{13}C\{^1H\}$**-NMR of compound 7b**

$^{13}C\{^{1}H\}$-DEPT-NMR 1,3-Dimethyl-5-phenyl-4-(1H-pyrrol-2-yl)-1H-imidazol-3-ium iodide (7b):

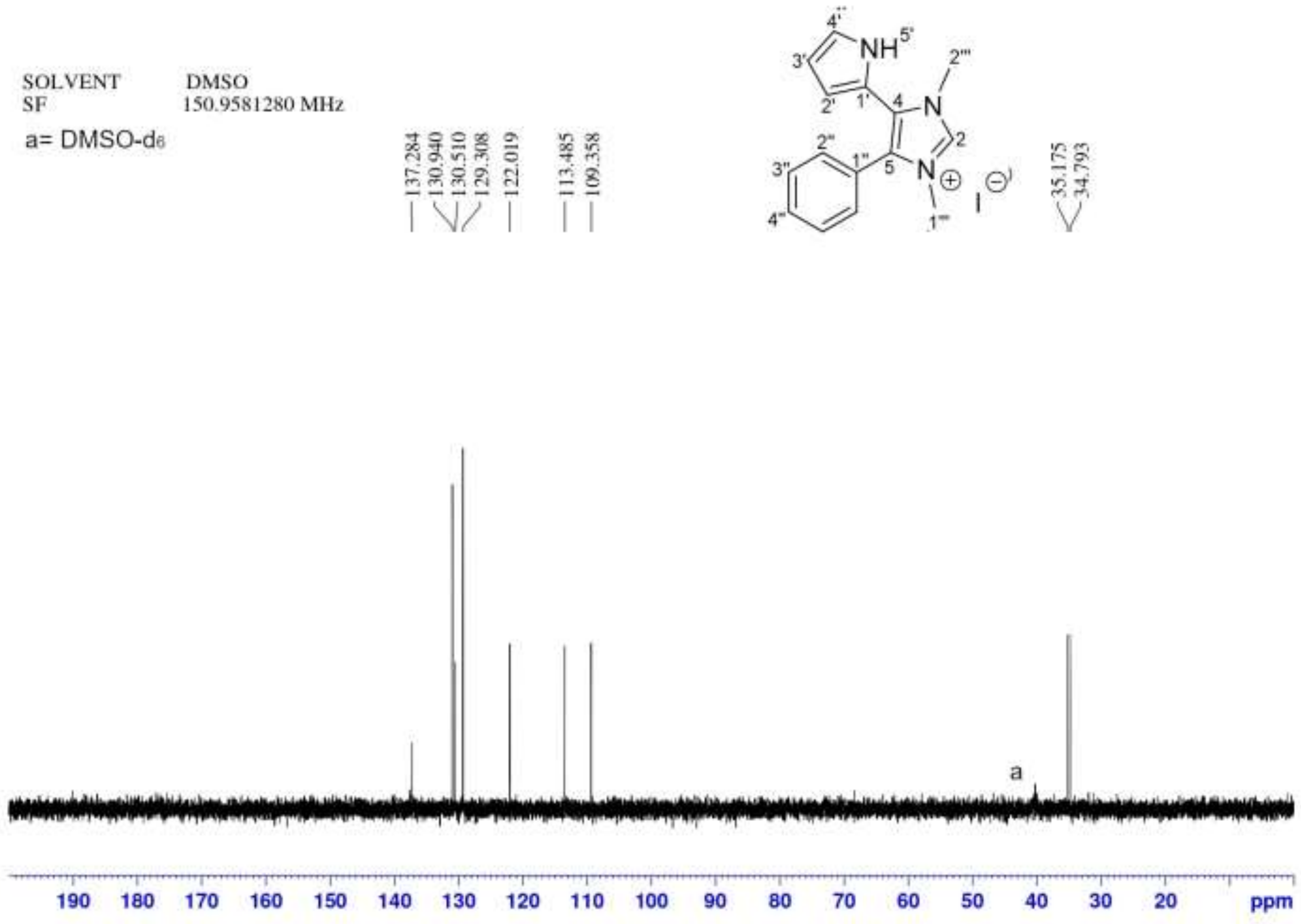

Figure S34. $^{13}C\{^{1}H\}$-DEPT-NMR of compound 7b

HSQC-NMR 1,3-Dimethyl-5-phenyl-4-(1H-pyrrol-2-yl)-1H-imidazol-3-ium iodide (7b):

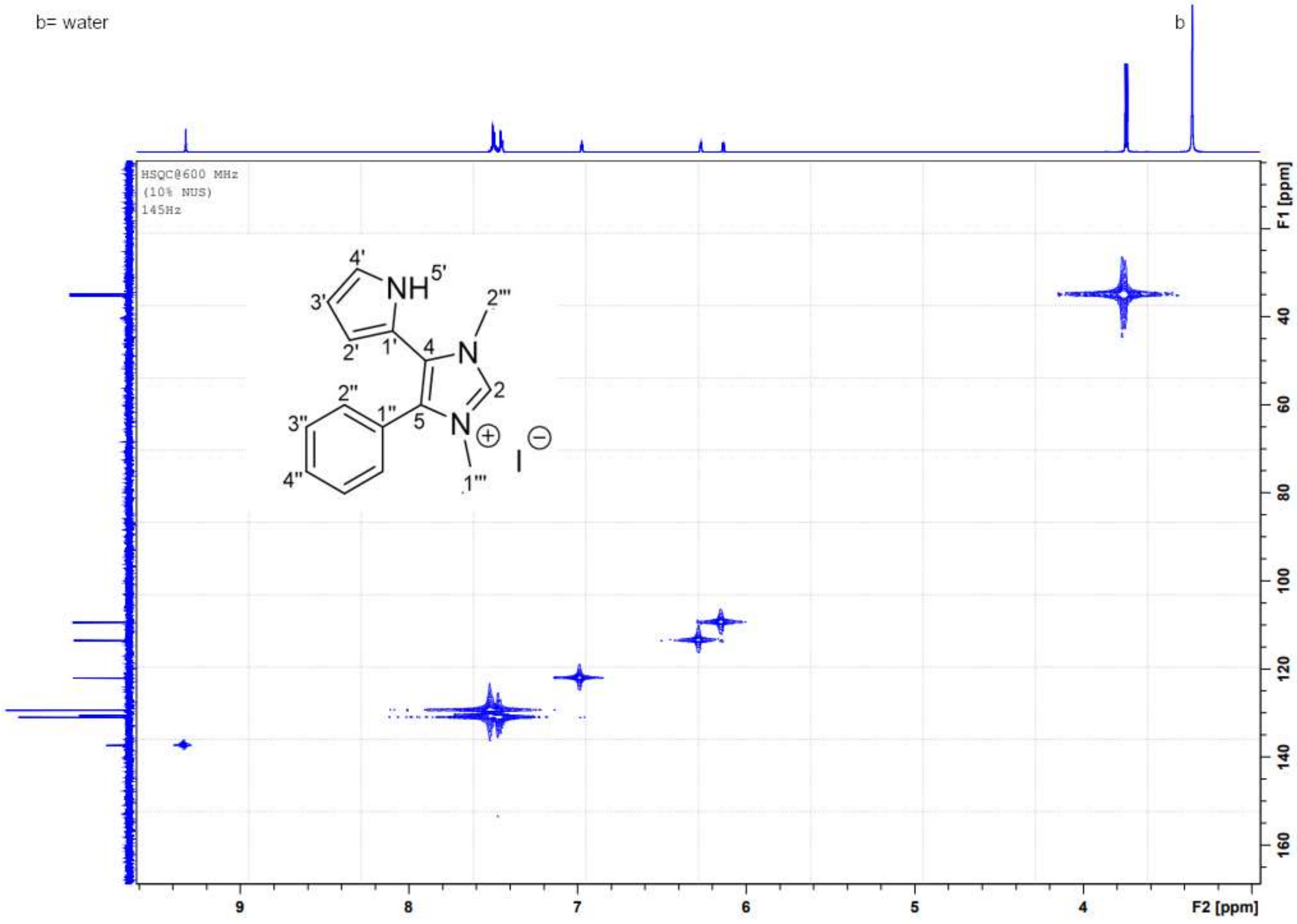

Figure S35. HSQC-NMR of compound 7b

HMBC-NMR 1,3-Dimethyl-5-phenyl-4-(1H-pyrrol-2-yl)-1H-imidazol-3-ium iodide (7b):

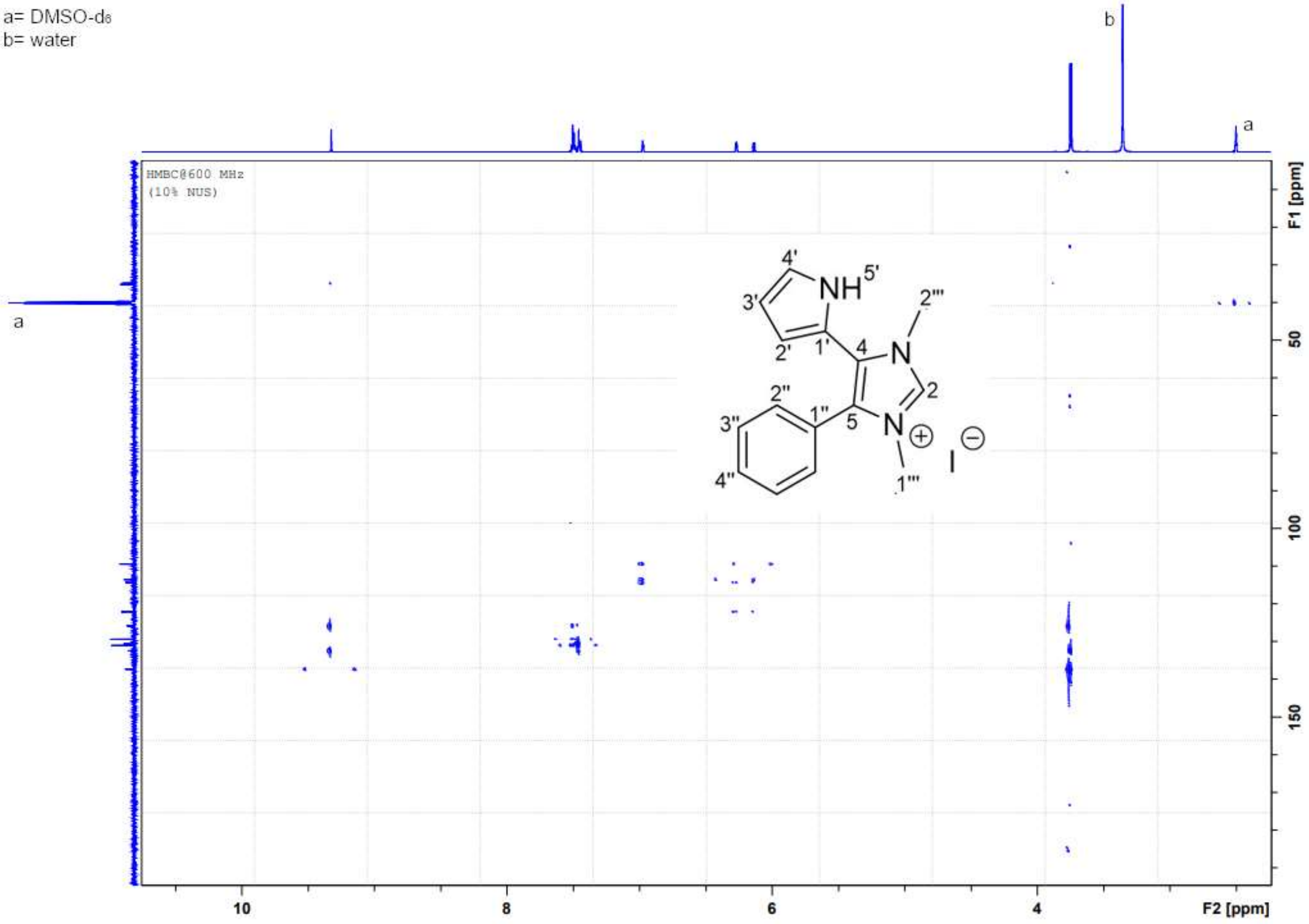

Figure S36. HMBC-NMR of compound 7b

^{1}H-NMR 1,3-Dimethyl-4-(1H-pyrrol-2-yl)-5-(p-tolyl)-1H-imidazol-3-ium iodide (7c):

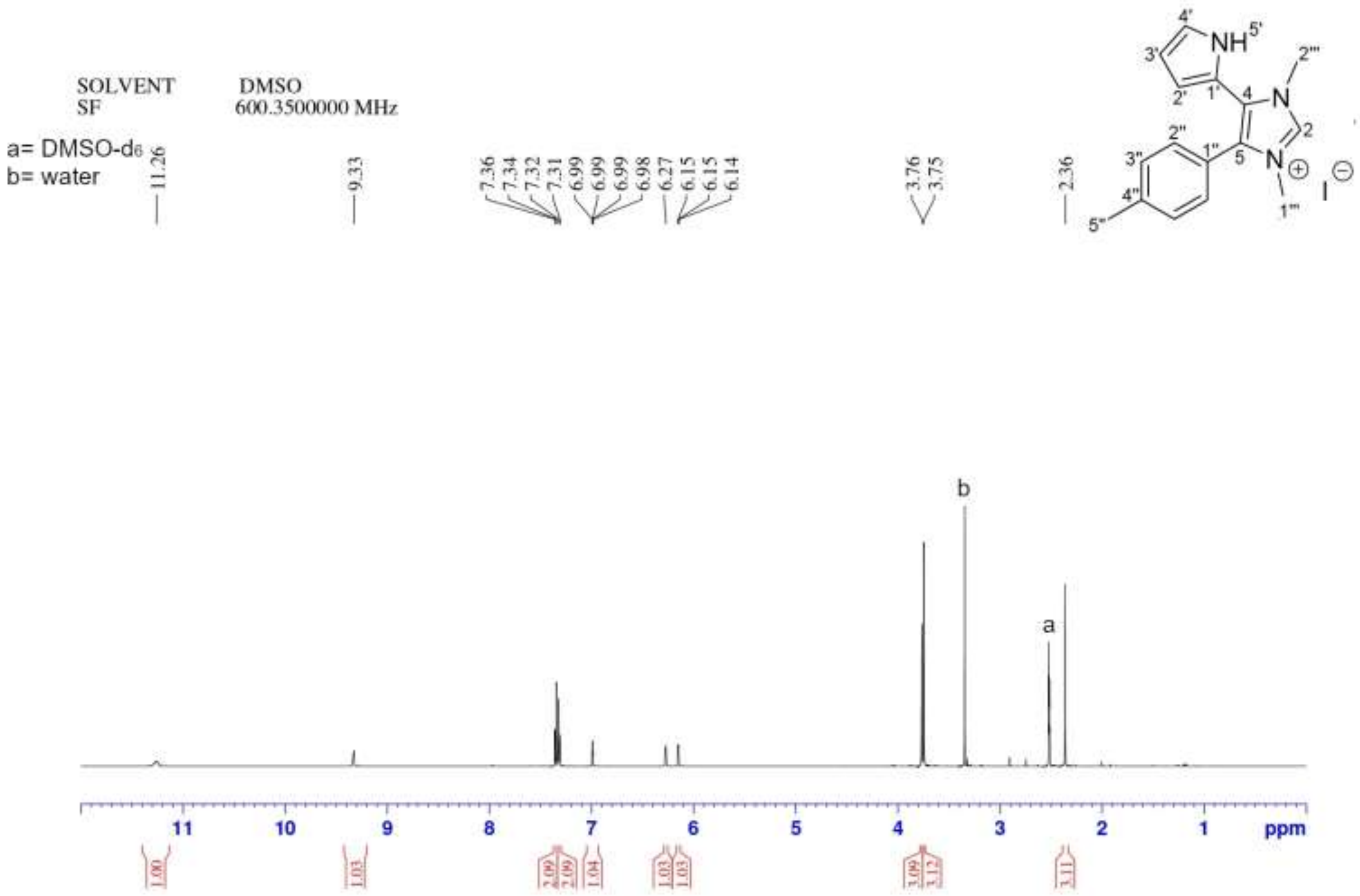

Figure S37. **^{1}H-NMR of compound 7c**

$^{13}C\{^1H\}$-NMR 1,3-Dimethyl-4-(1H-pyrrol-2-yl)-5-(p-tolyl)-1H-imidazol-3-ium iodide (7c):

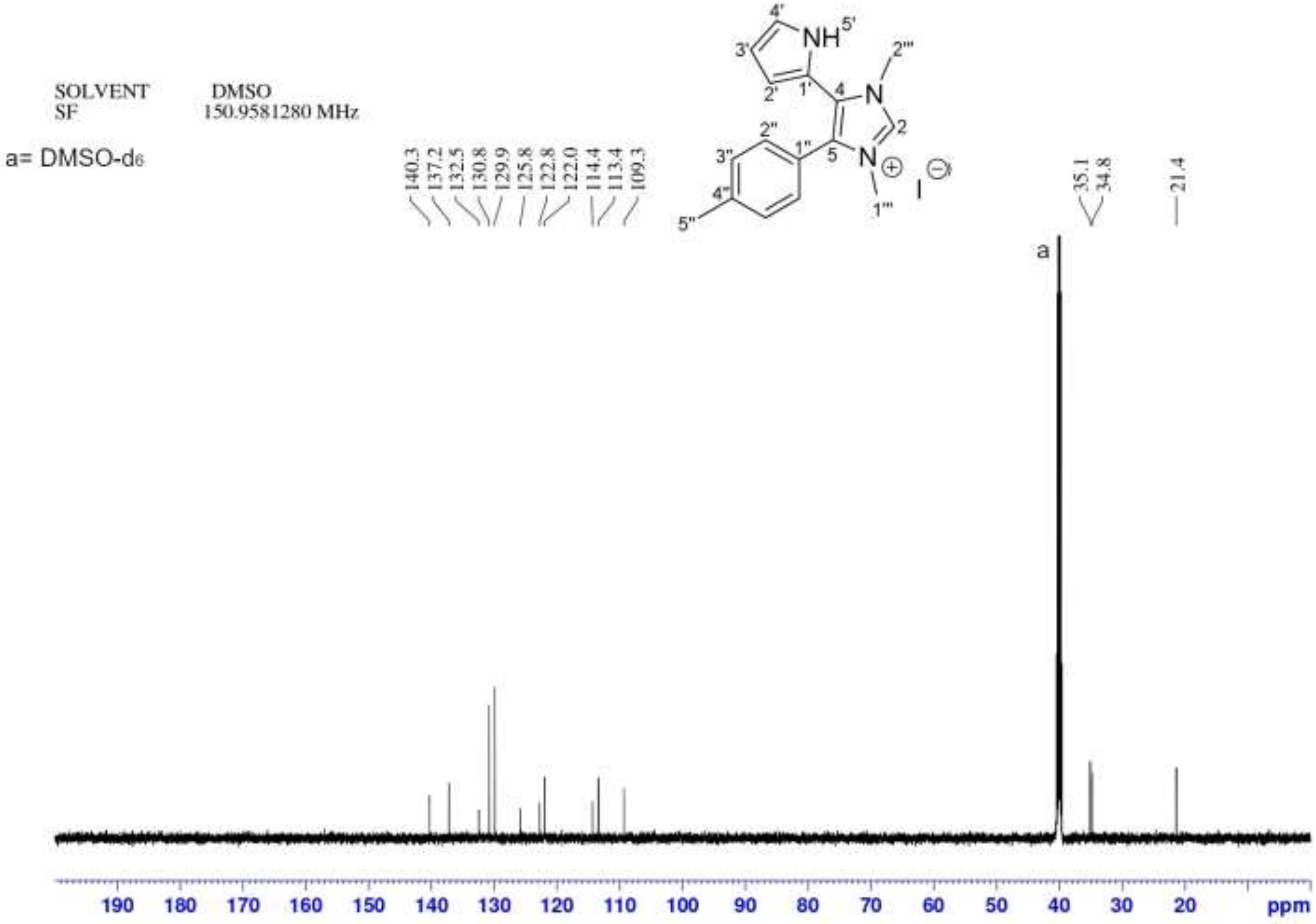

Figure S38. **$^{13}C\{^1H\}$-NMR of compound 7c**

$^{13}C\{^{1}H\}$-DEPT-NMR 1,3-Dimethyl-4-(1H-pyrrol-2-yl)-5-(p-tolyl)-1H-imidazol-3-ium iodide (7c):

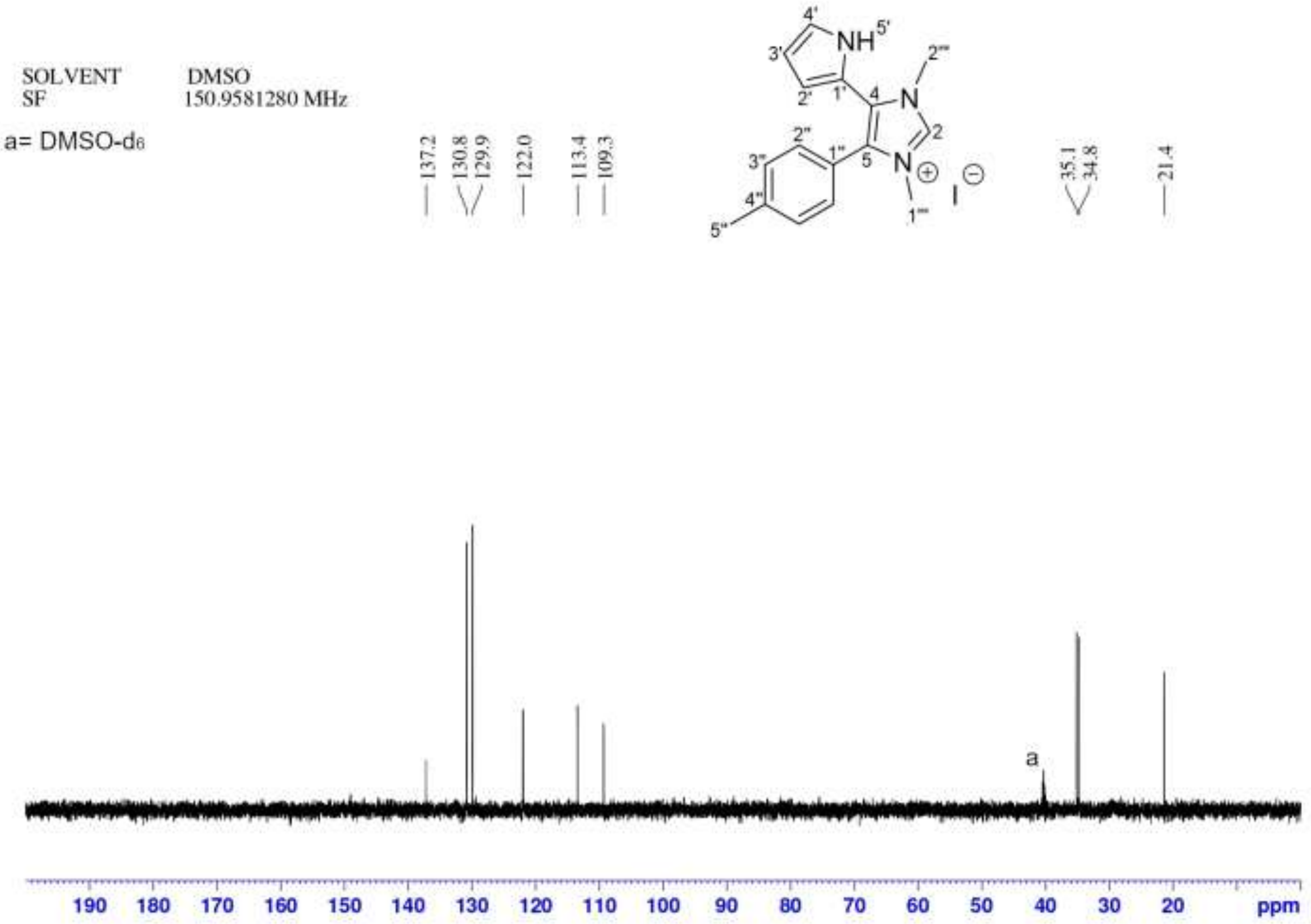

Figure S39. **$^{13}C\{^{1}H\}$-DEPT-NMR of compound 7c**

HSQC-NMR 1,3-Dimethyl-4-(1H-pyrrol-2-yl)-5-(p-tolyl)-1H-imidazol-3-ium iodide (7c):

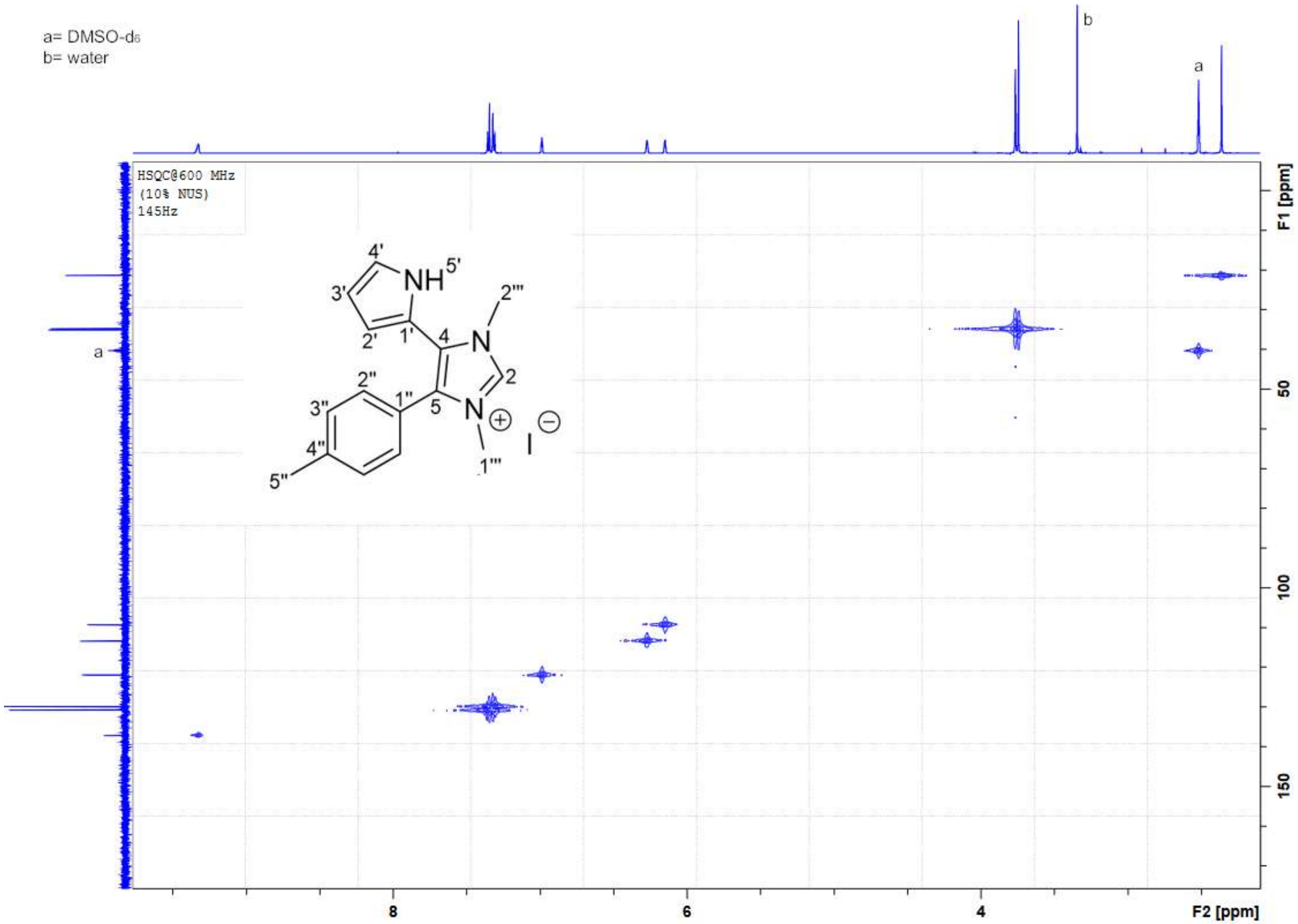

Figure S40. HSQC-NMR of compound 7c

HMBC-NMR 1,3-Dimethyl-4-(1H-pyrrol-2-yl)-5-(p-tolyl)-1H-imidazol-3-ium iodide (7c):

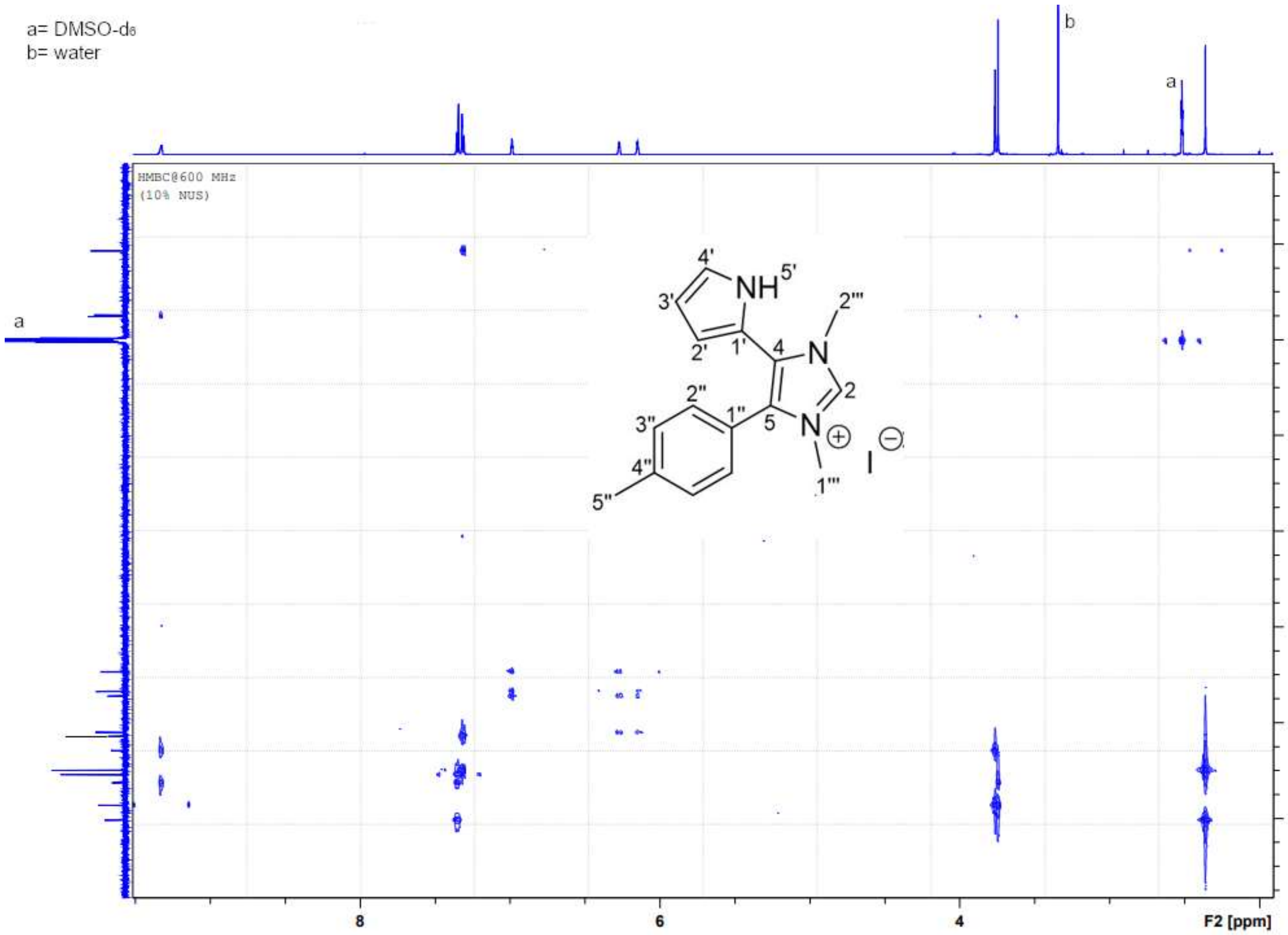

Figure S41. HMBC-NMR of compound 7c

^{1}H-NMR 5-(4-Methoxyphenyl)-1,3-dimethyl-4-(1H-pyrrol-2-yl)-1H-imidazol-3-ium, iodide (7d):

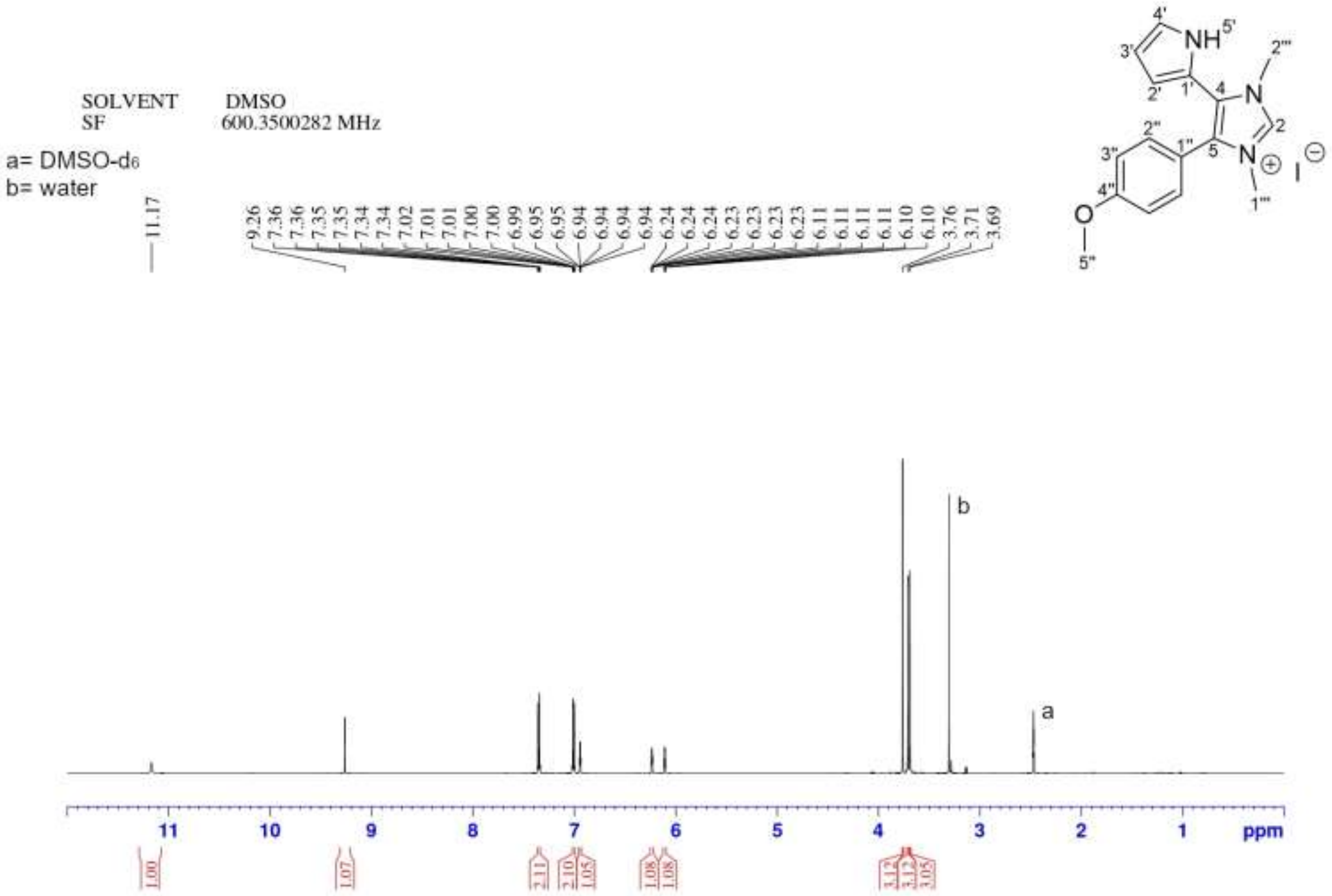

Figure S42. ^{1}H-NMR of compound 7d

$^{13}C\{^1H\}$-NMR 5-(4-Methoxyphenyl)-1,3-dimethyl-4-(1H-pyrrol-2-yl)-1H-imidazol-3-ium, iodide (7d):

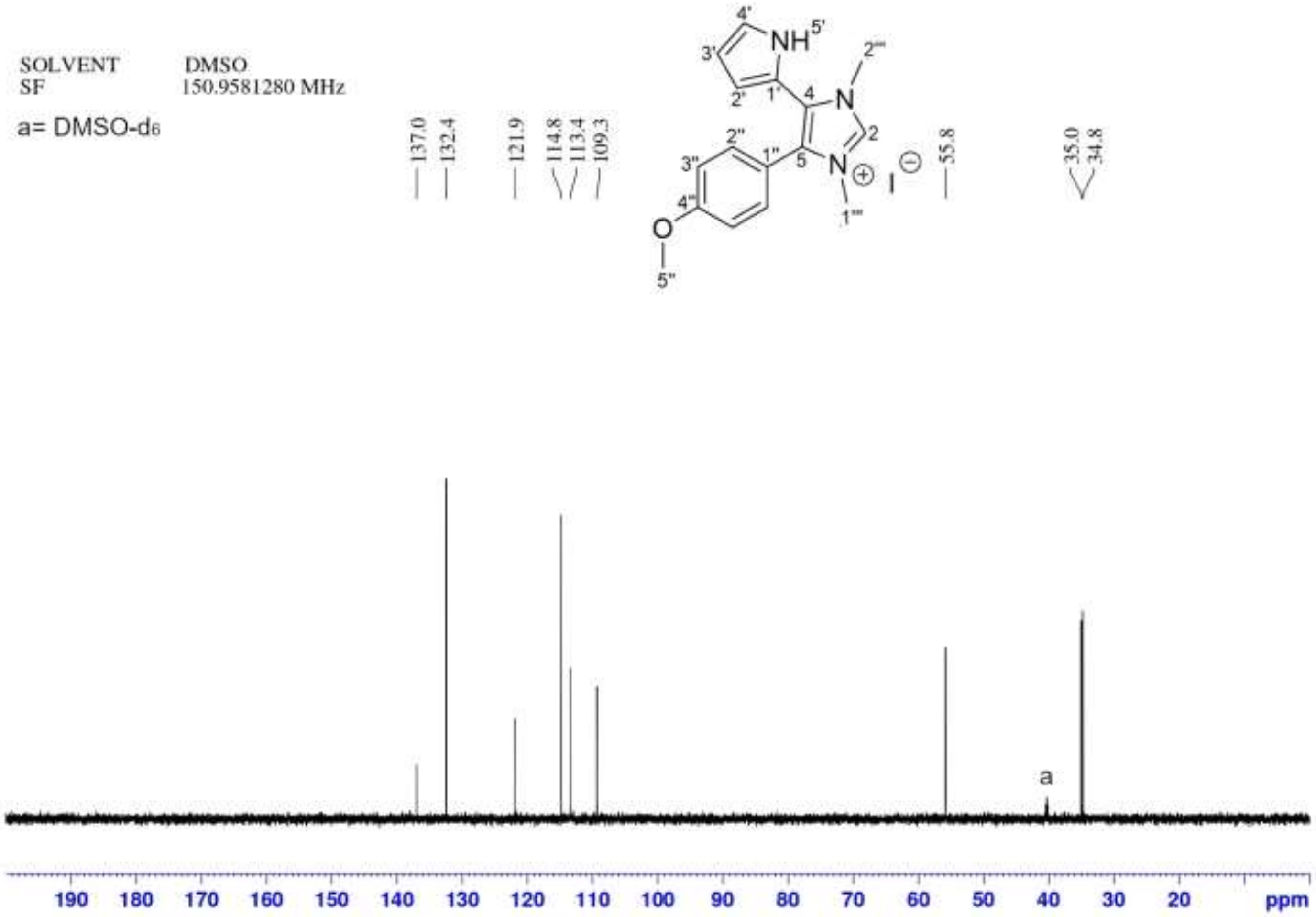

Figure S43. **$^{13}C\{^1H\}$-NMR of compound 7d**

$^{13}C\{^{1}H\}$-DEPT-NMR 5-(4-Methoxyphenyl)-1,3-dimethyl-4-(1H-pyrrol-2-yl)-1H-imidazol-3-ium, iodide (7d):

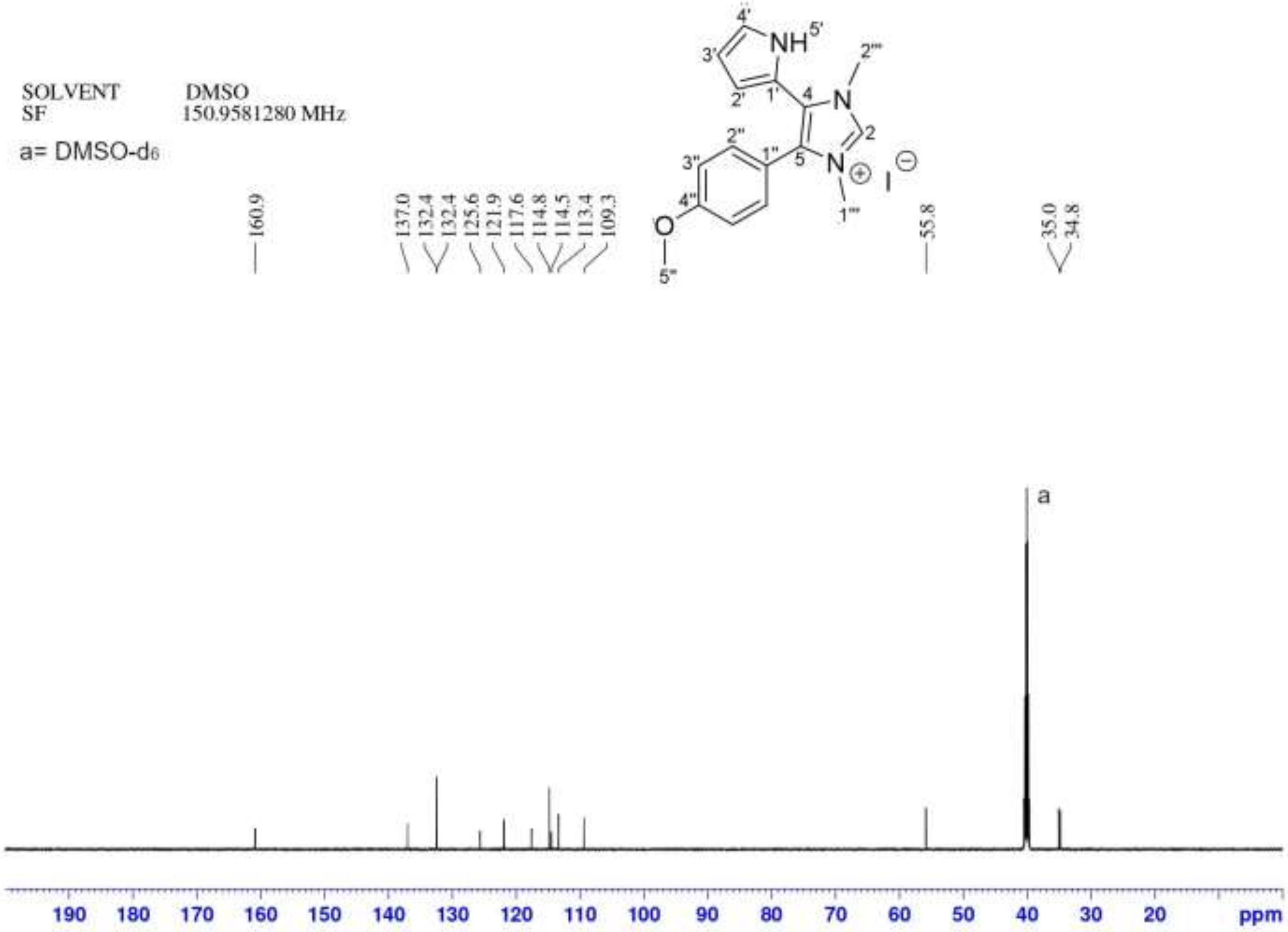

Figure S44. **$^{13}C\{^{1}H\}$-DEPT-NMR of compound 7d**

HSQC-NMR 5-(4-Methoxyphenyl)-1,3-dimethyl-4-(1H-pyrrol-2-yl)-1H-imidazol-3-ium, iodide (7d):

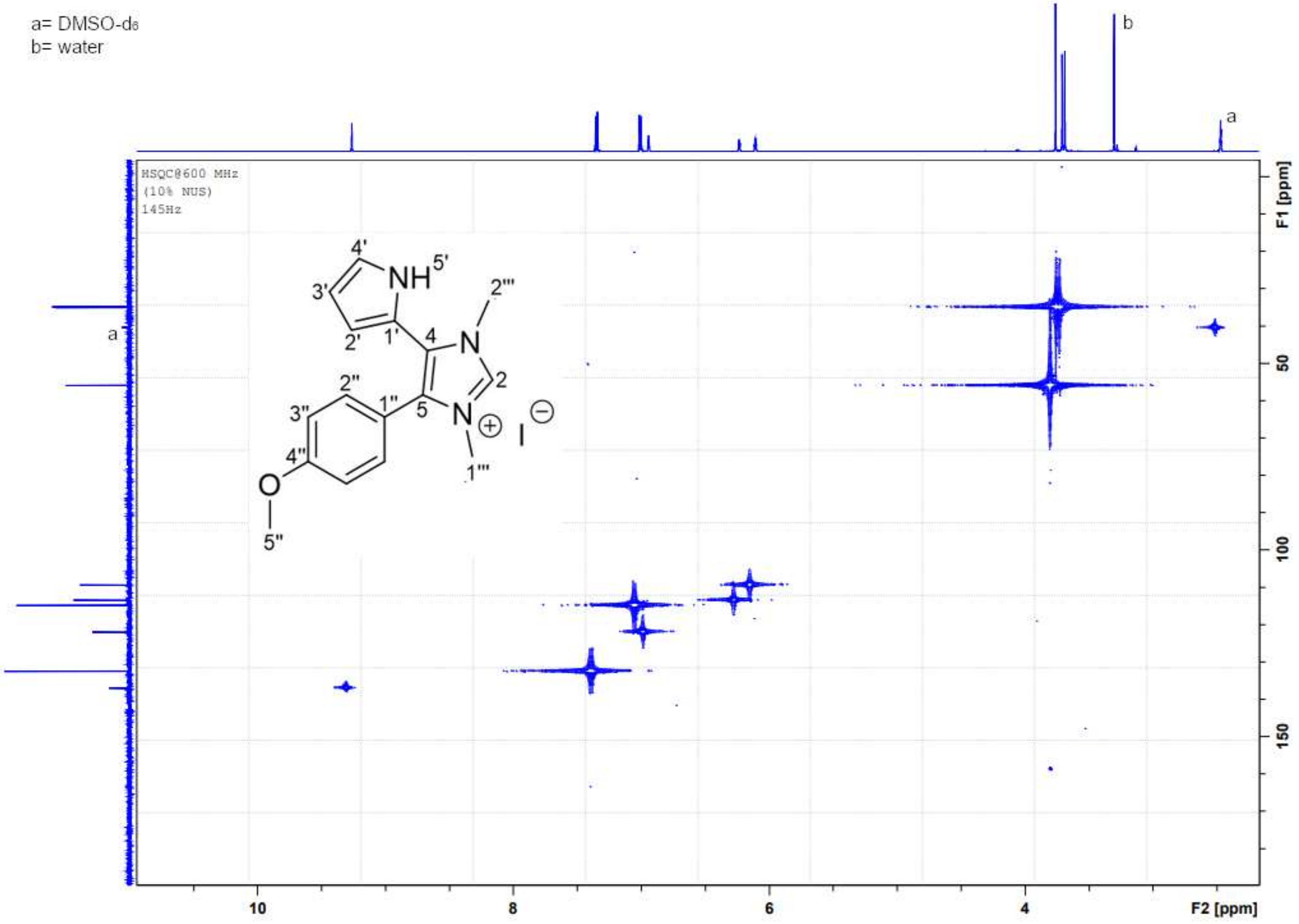

Figure S45. HSQC-NMR of compound 7d

HMBC-NMR 5-(4-Methoxyphenyl)-1,3-dimethyl-4-(1H-pyrrol-2-yl)-1H-imidazol-3-ium, iodide (7d):

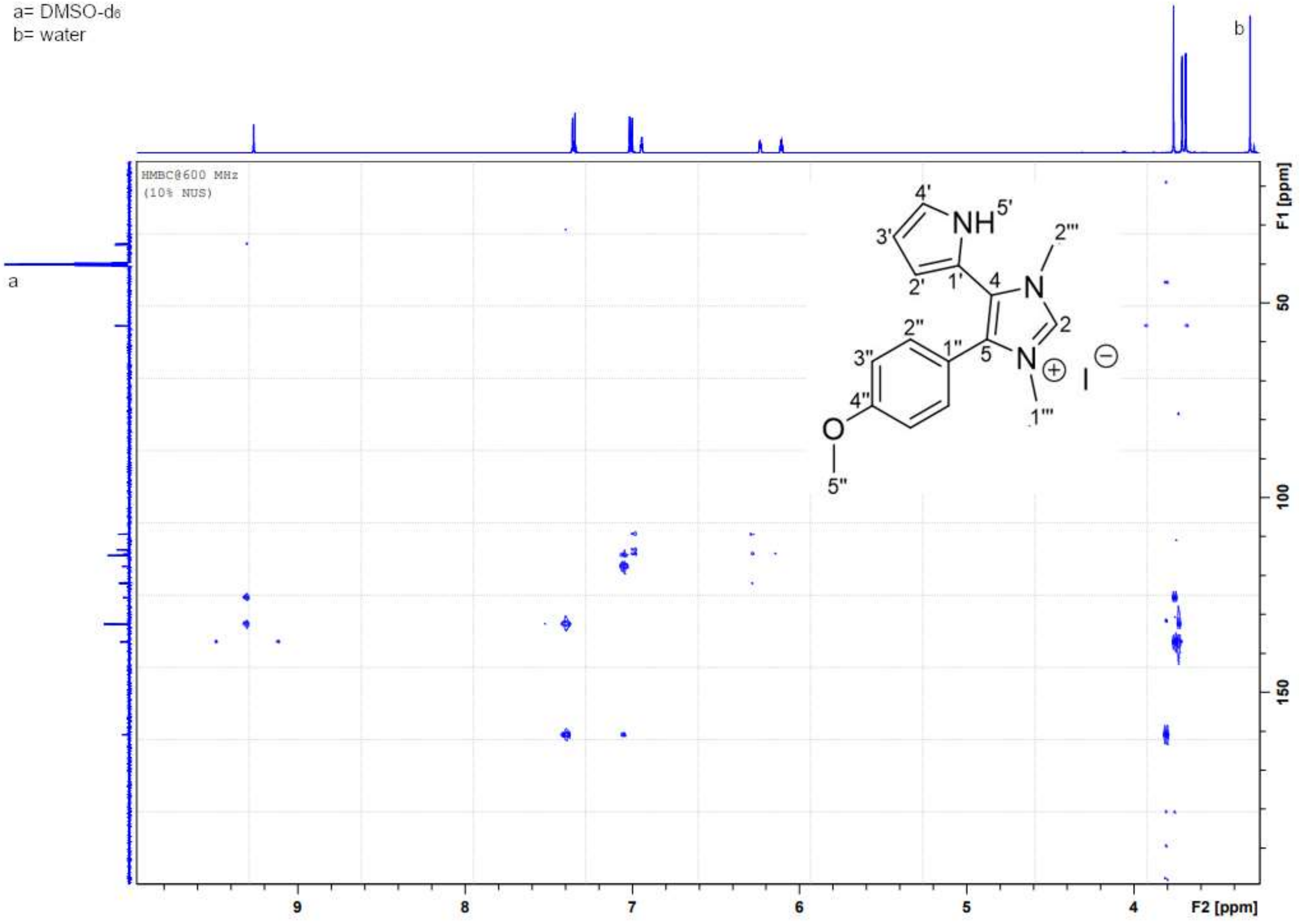

Figure S46. HMBC-NMR of compound 7d

^{1}H-NMR 3-Benzyl-1-methyl-4-(1H-pyrrol-2-yl)-1H-imidazol-3-ium bromide (7e):

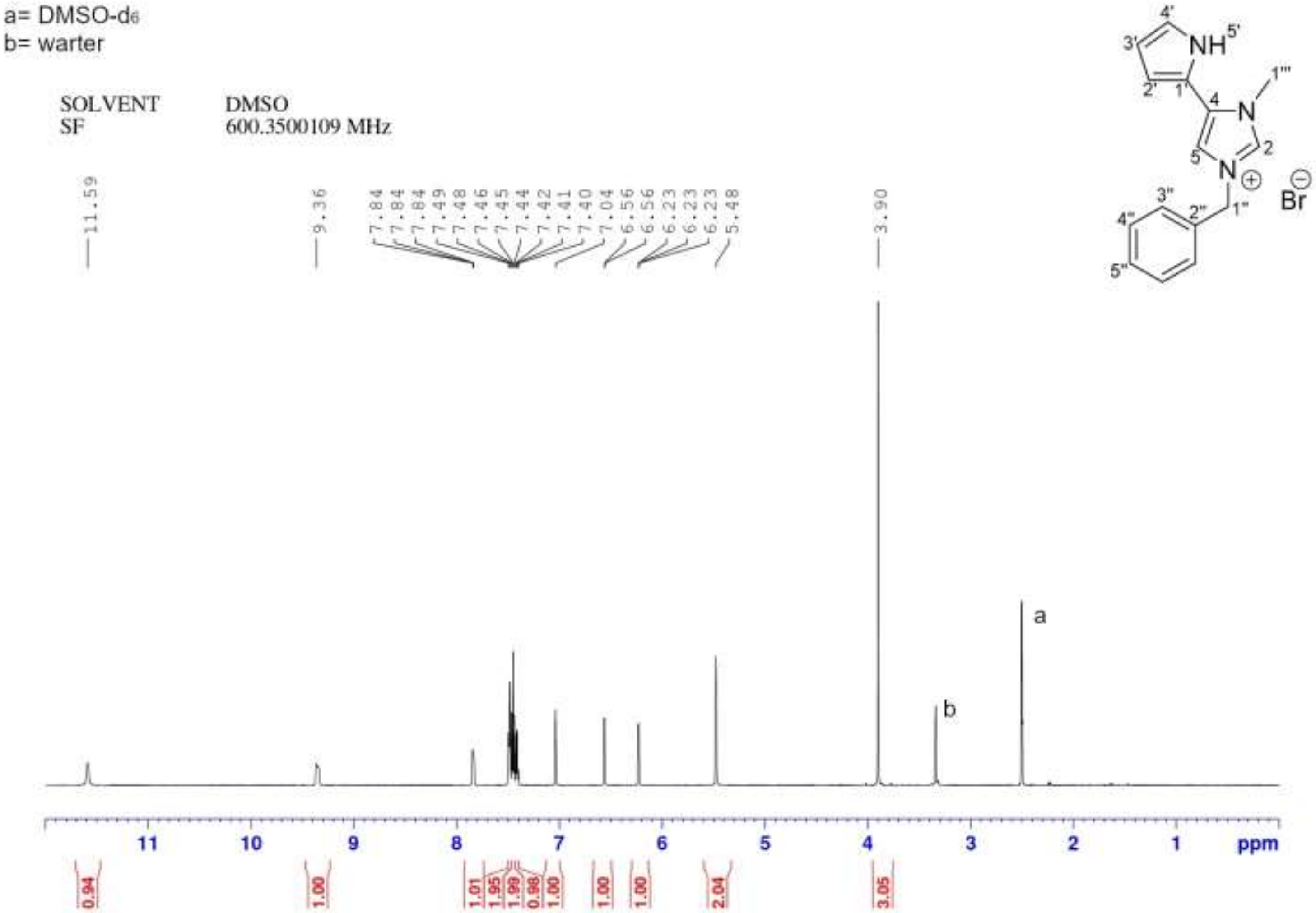

Figure S47. ^{1}H-NMR of compound 7e

$^{13}C\{^1H\}$-NMR 1-Benzyl-3-methyl-4-(1H-pyrrol-2-yl)-1H-imidazol-3-ium bromide (7e):

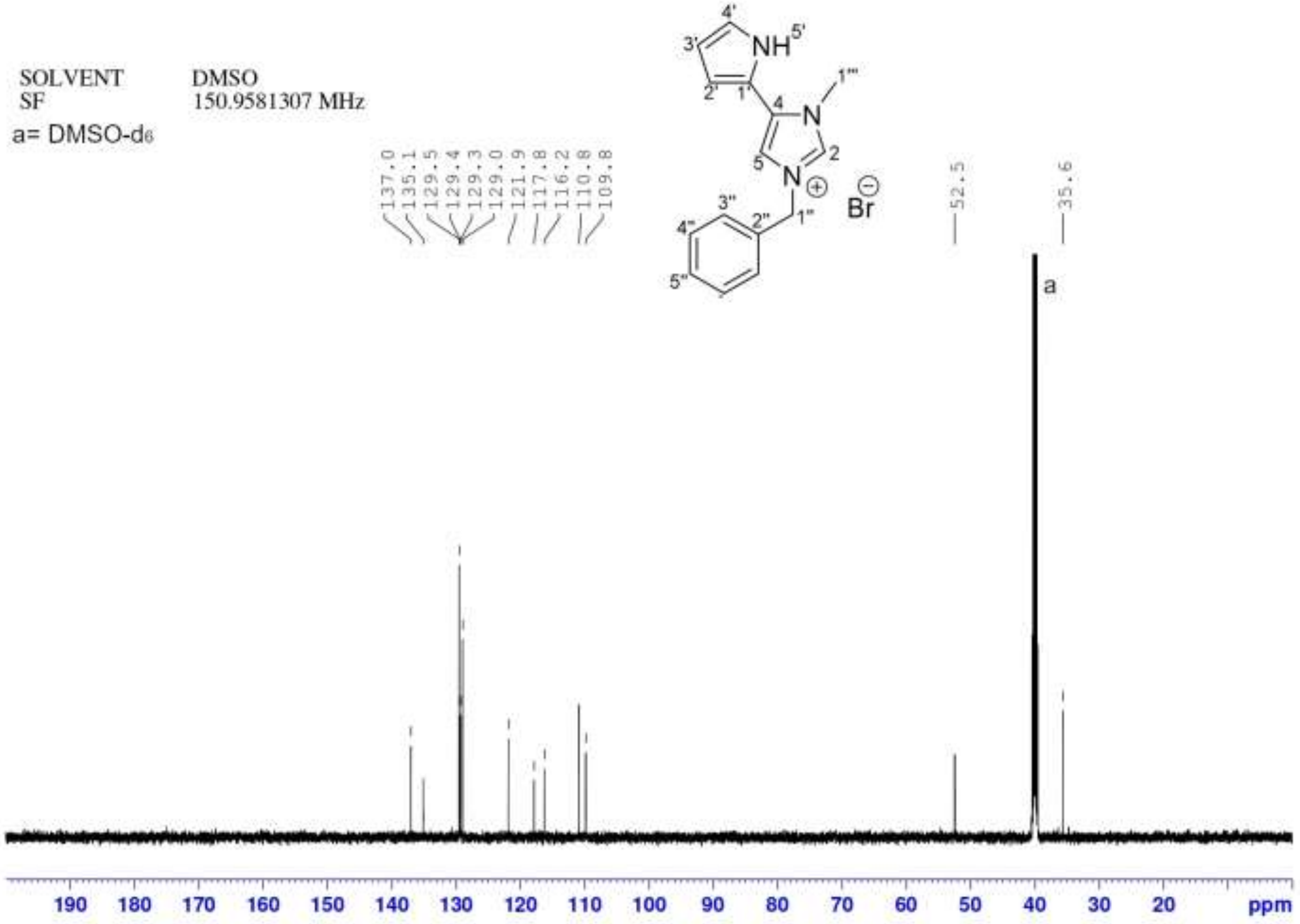

Figure S48. $^{13}C\{^1H\}$**-NMR of compound 7e**

$^{13}C\{^1H\}$-DEPT-NMR 1-Benzyl-3-methyl-4-(1H-pyrrol-2-yl)-1H-imidazol-3-ium bromide (7e):

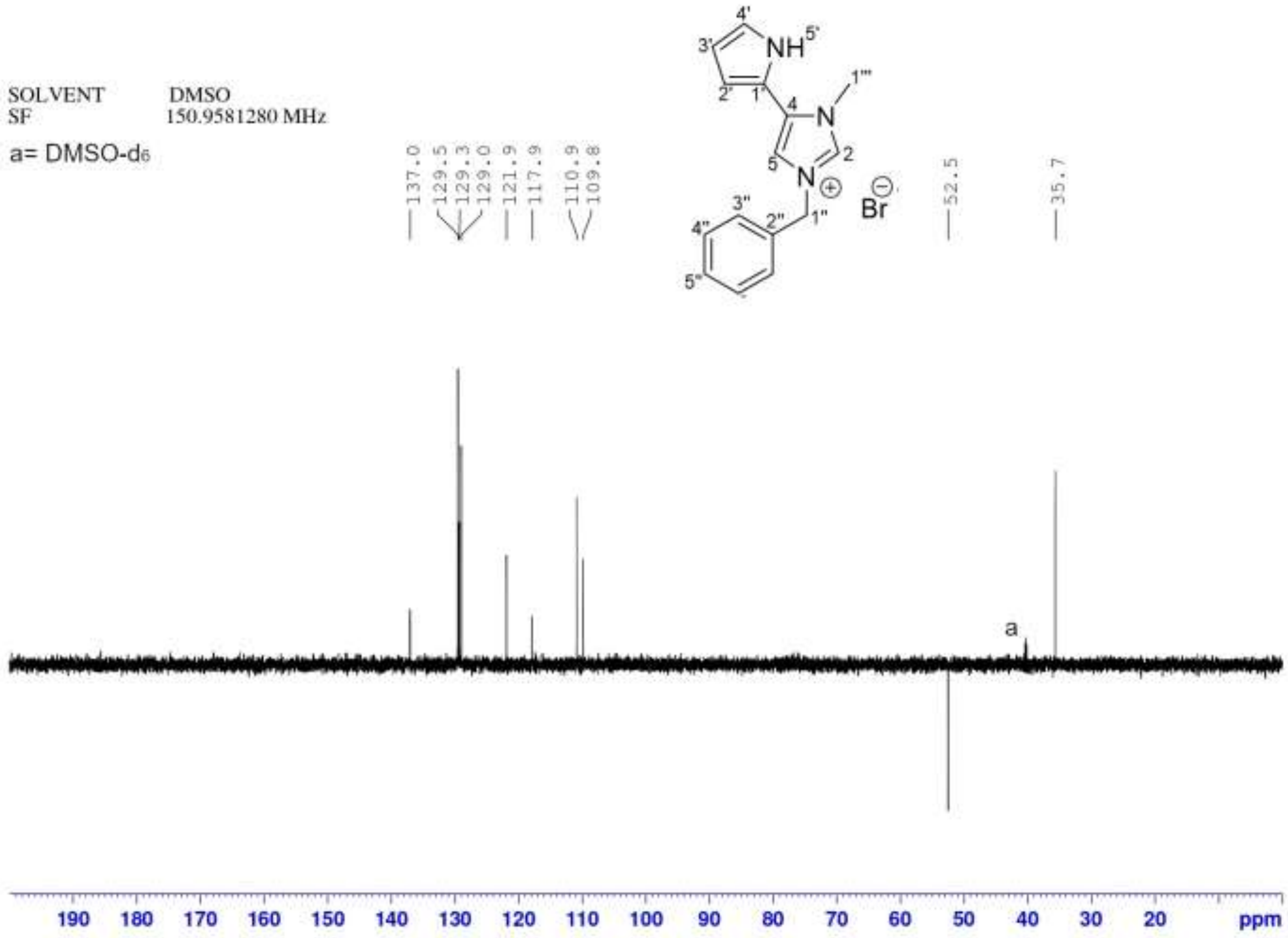

Figure S49. **$^{13}C\{^1H\}$-DEPT-NMR of compound 7e**

HSQC-NMR 3-Benzyl-1-methyl-4-(1H-pyrrol-2-yl)-1H-imidazol-3-ium bromide (7e):

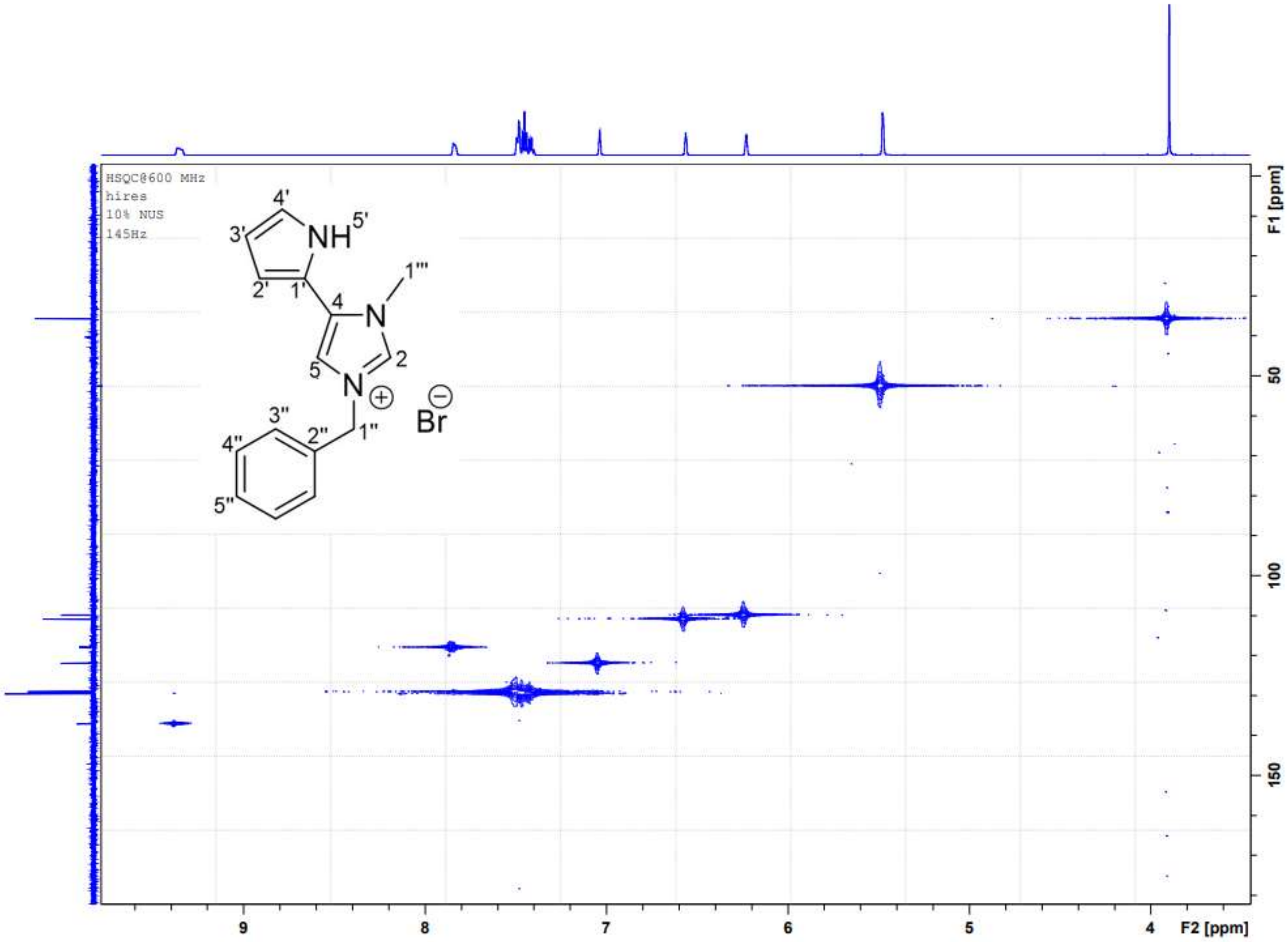

Figure S50. HSQC-NMR of compound 7e

HMBC-NMR 3-Benzyl-1-methyl-4-(1H-pyrrol-2-yl)-1H-imidazol-3-ium bromide (7e):

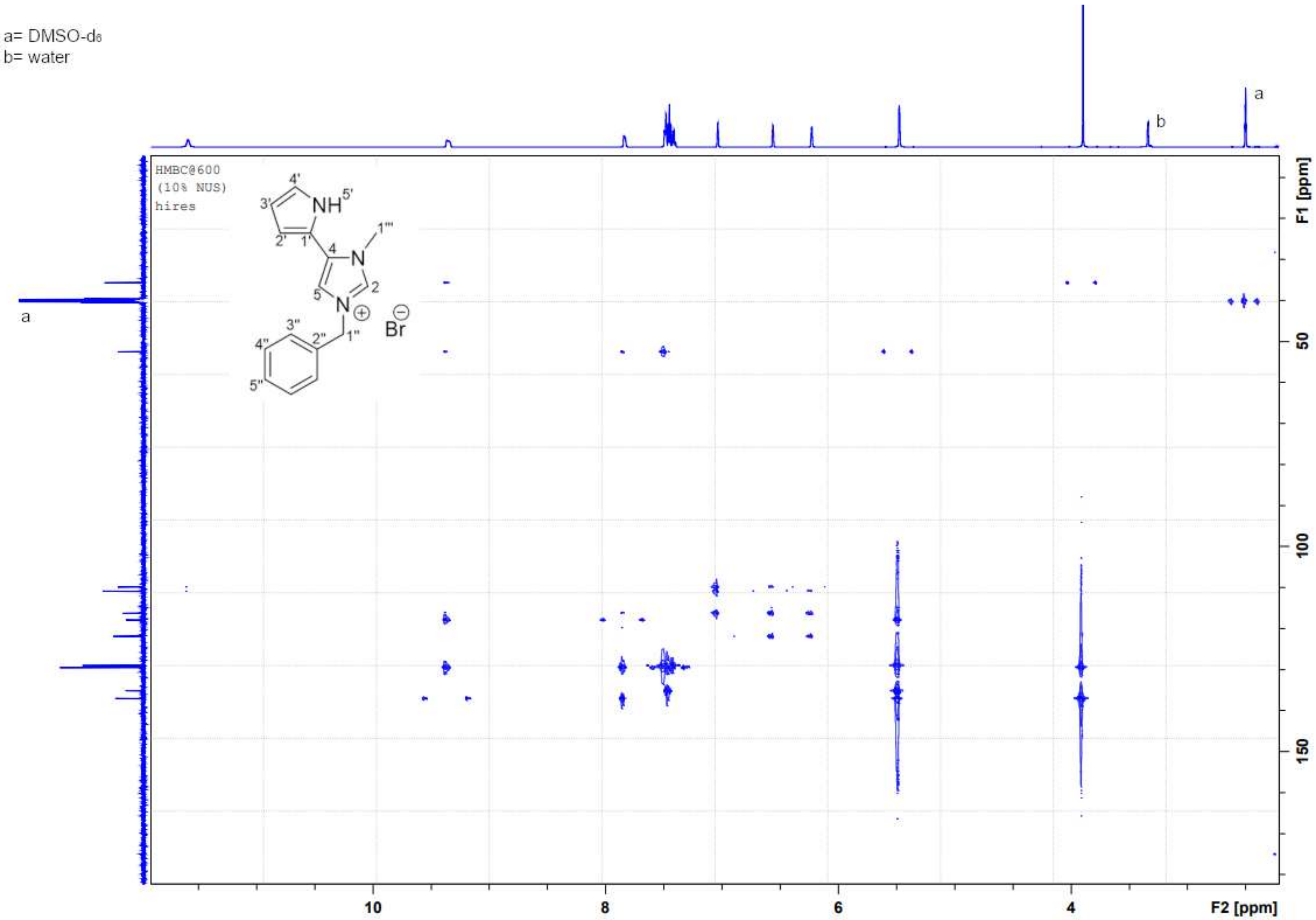

Figure S51. HMBC-NMR of compound 7e

^{1}H-NMR 3-Methyl-1-(4-methylbenzyl)-4-(1H-pyrrol-2-yl)-1H-imidazol-3-ium bromide (7f):

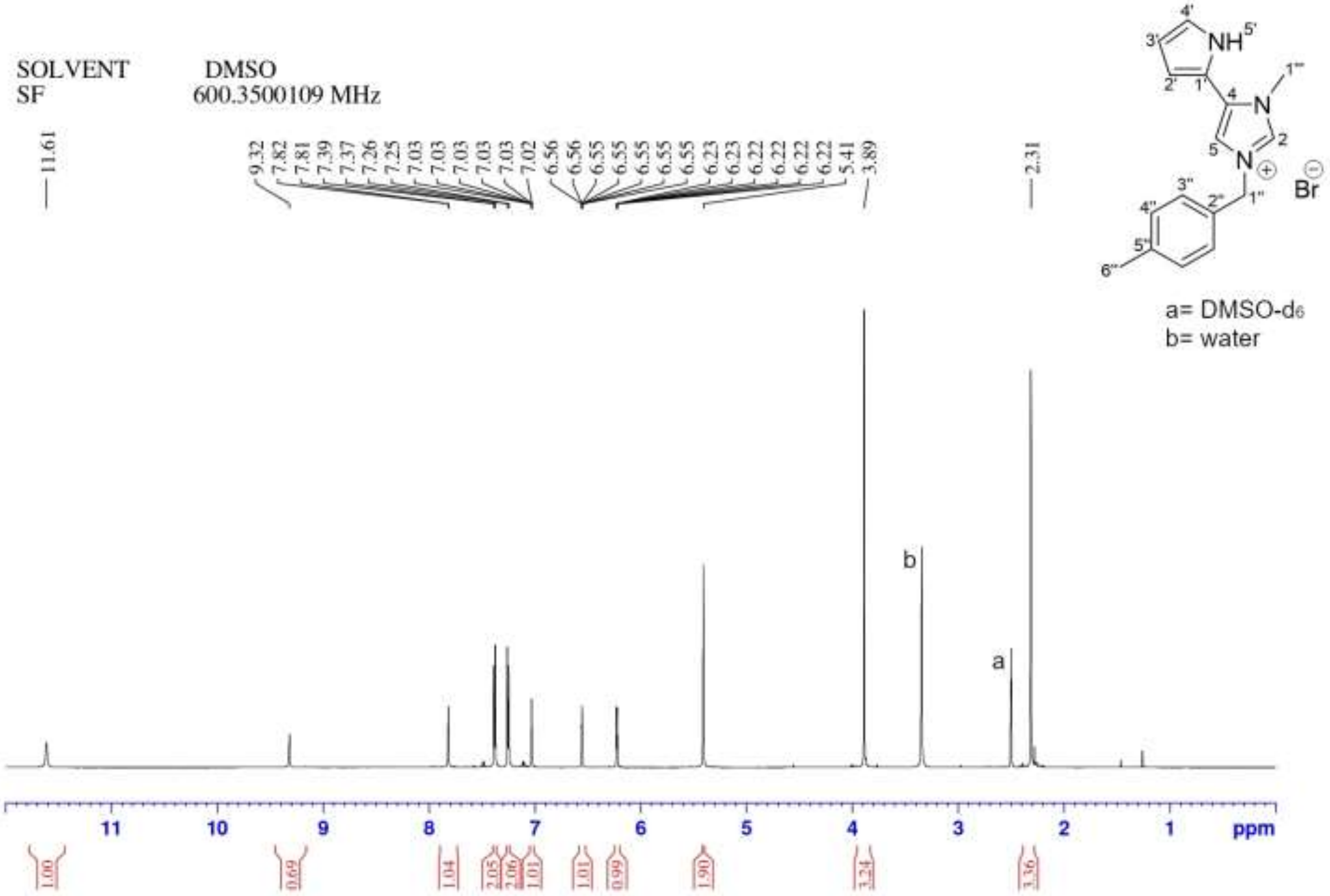

Figure S52. **^{1}H-NMR of compound 7f**

$^{13}C\{^1H\}$-NMR 3-Methyl-1-(4-methylbenzyl)-4-(1H-pyrrol-2-yl)-1H-imidazol-3-ium bromide (7f):

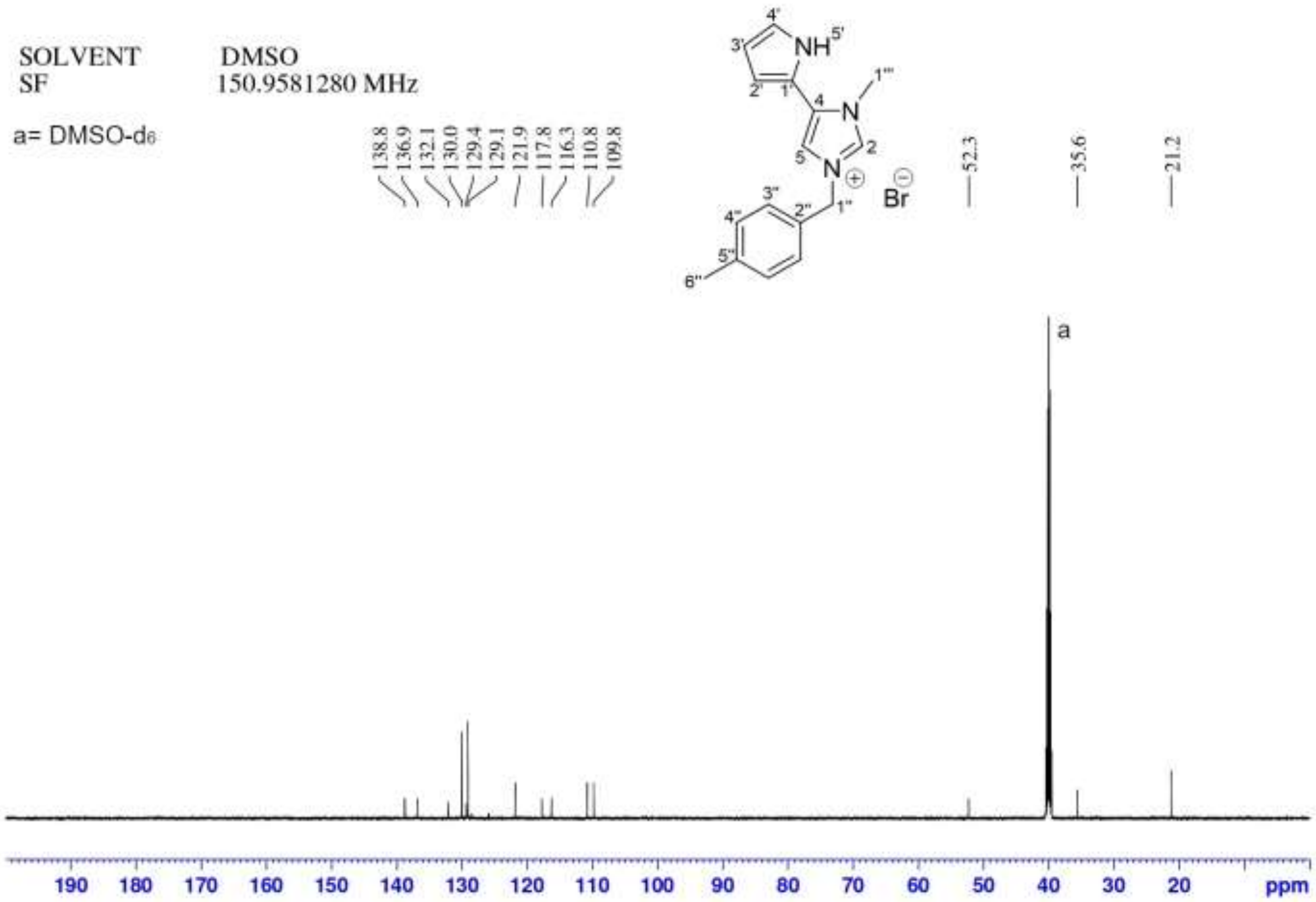

Figure S53. **$^{13}C\{^1H\}$-NMR of compound 7f**

$^{13}C\{^1H\}$-DEPT-NMR 3-Methyl-1-(4-methylbenzyl)-4-(1H-pyrrol-2-yl)-1H-imidazol-3-ium bromide (7f):

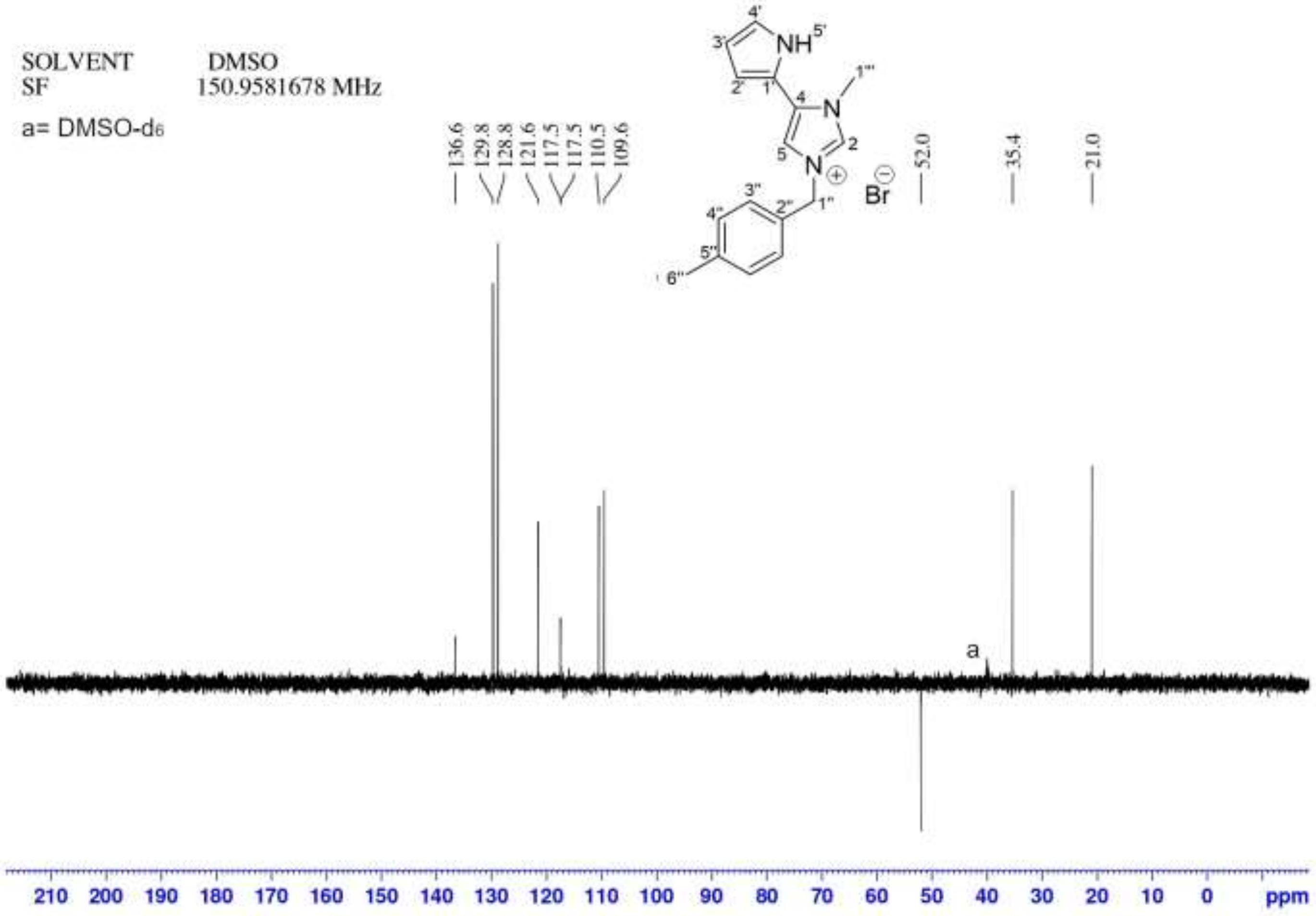

Figure S54. **$^{13}C\{^1H\}$-DEPT-NMR of compound 7f**

HSQC-NMR 3-Methyl-1-(4-methylbenzyl)-4-(1H-pyrrol-2-yl)-1H-imidazol-3-ium bromide (7f):

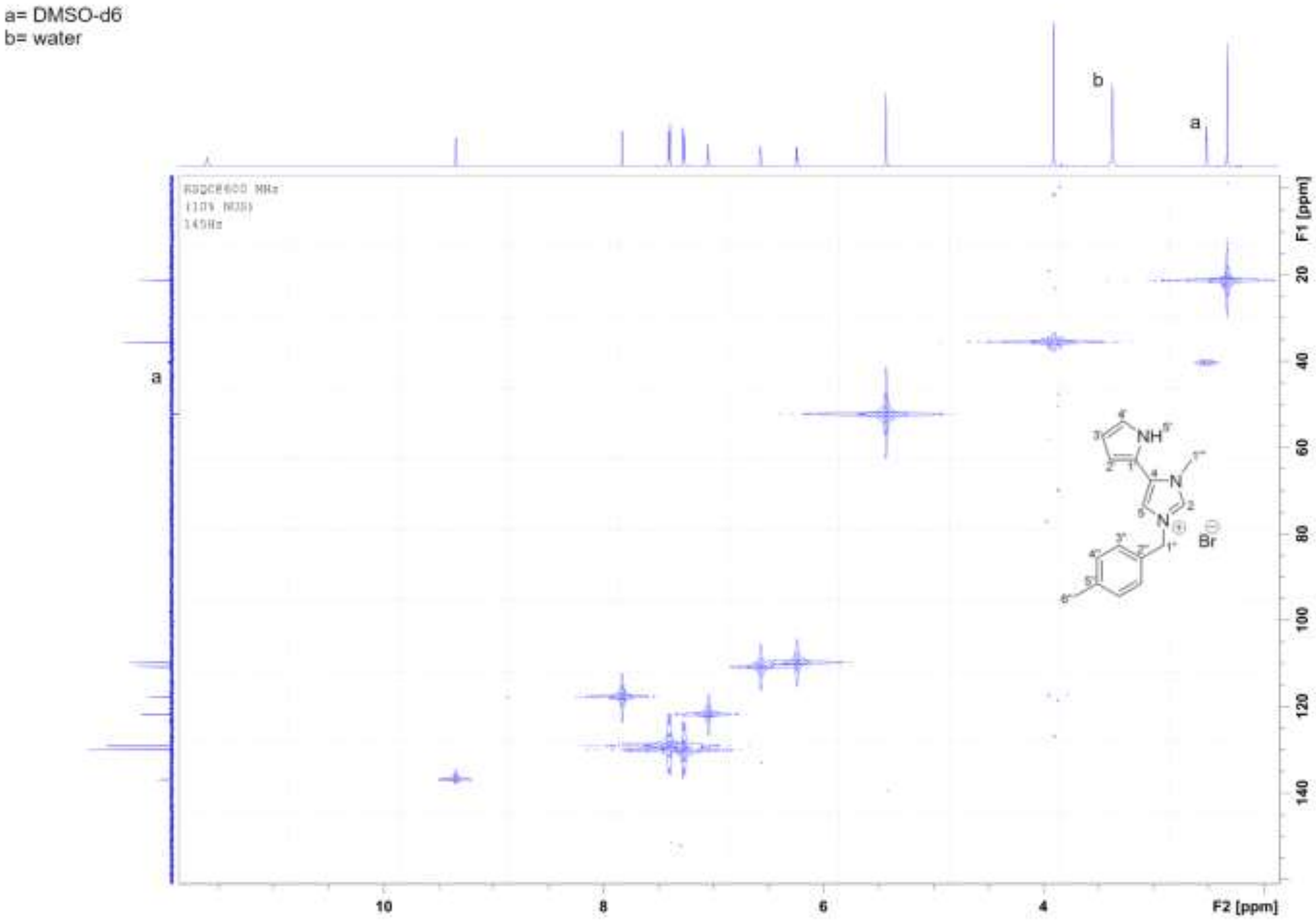

Figure S55. HSQC-NMR of compound 7f

HMBC-NMR 3-Methyl-1-(4-methylbenzyl)-4-(1H-pyrrol-2-yl)-1H-imidazol-3-ium bromide (7f):

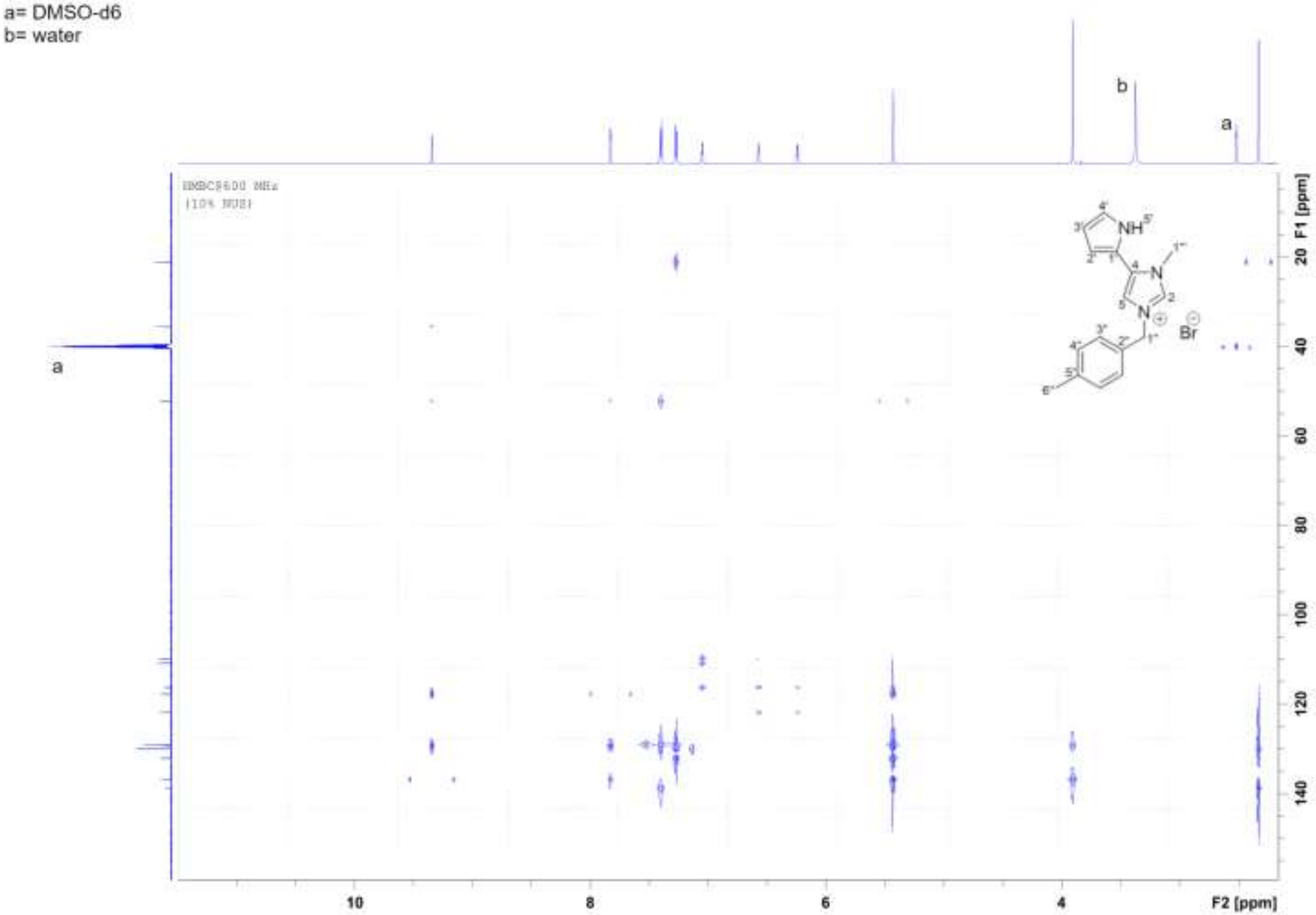

Figure S56. HMBC-NMR of compound 7f

^{1}H-NMR 3-(4-Methoxybenzyl)-1-methyl-4-(1H-pyrrol-2-yl)-1H-imidazol-3-ium bromide (7g):

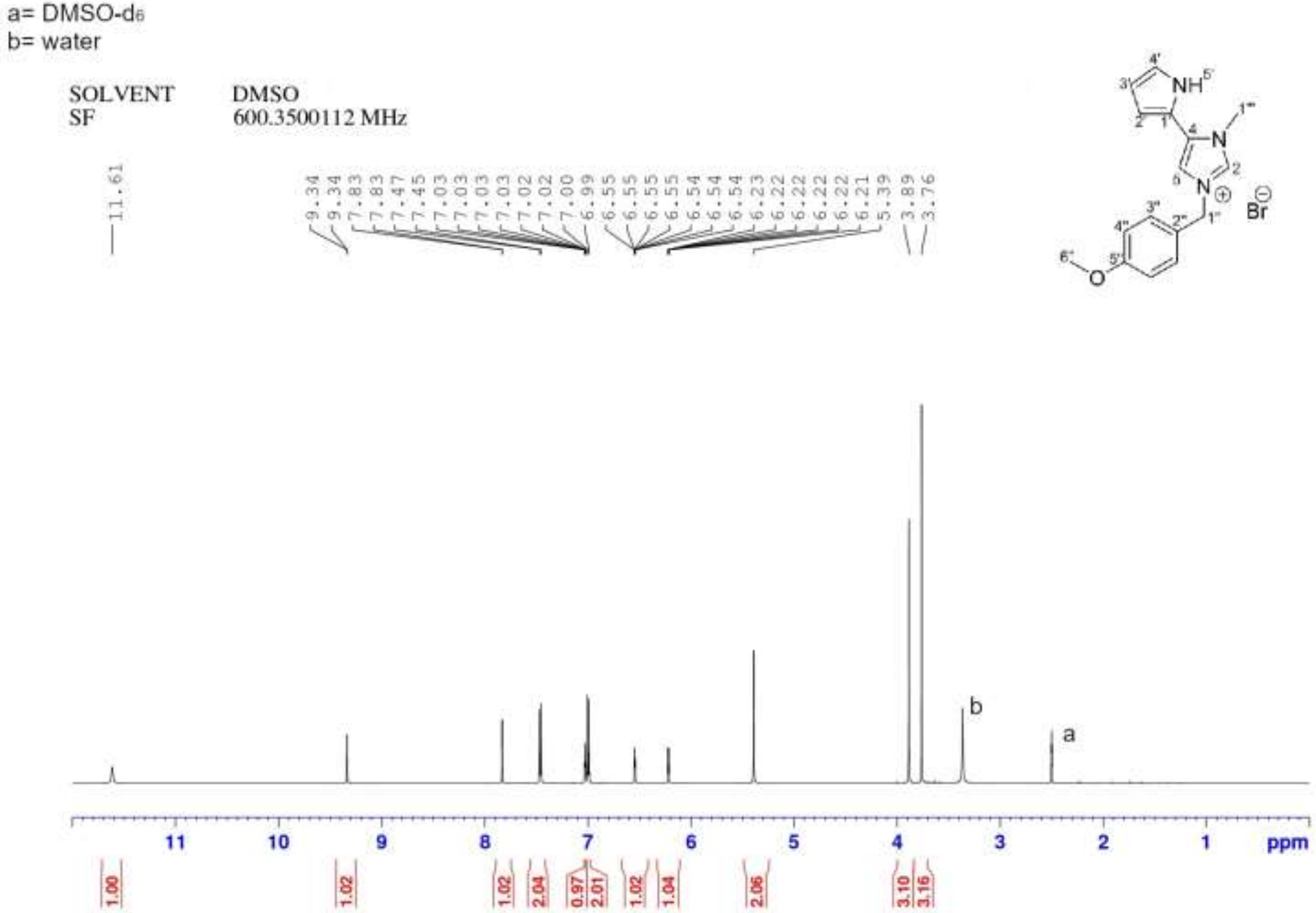

Figure S57. **^{1}H-NMR of compound 7g**

$^{13}C\{^{1}H\}$-NMR 1-(4-Methoxybenzyl)-3-methyl-4-(1H-pyrrol-2-yl)-1H-imidazol-3-ium bromide (7g):

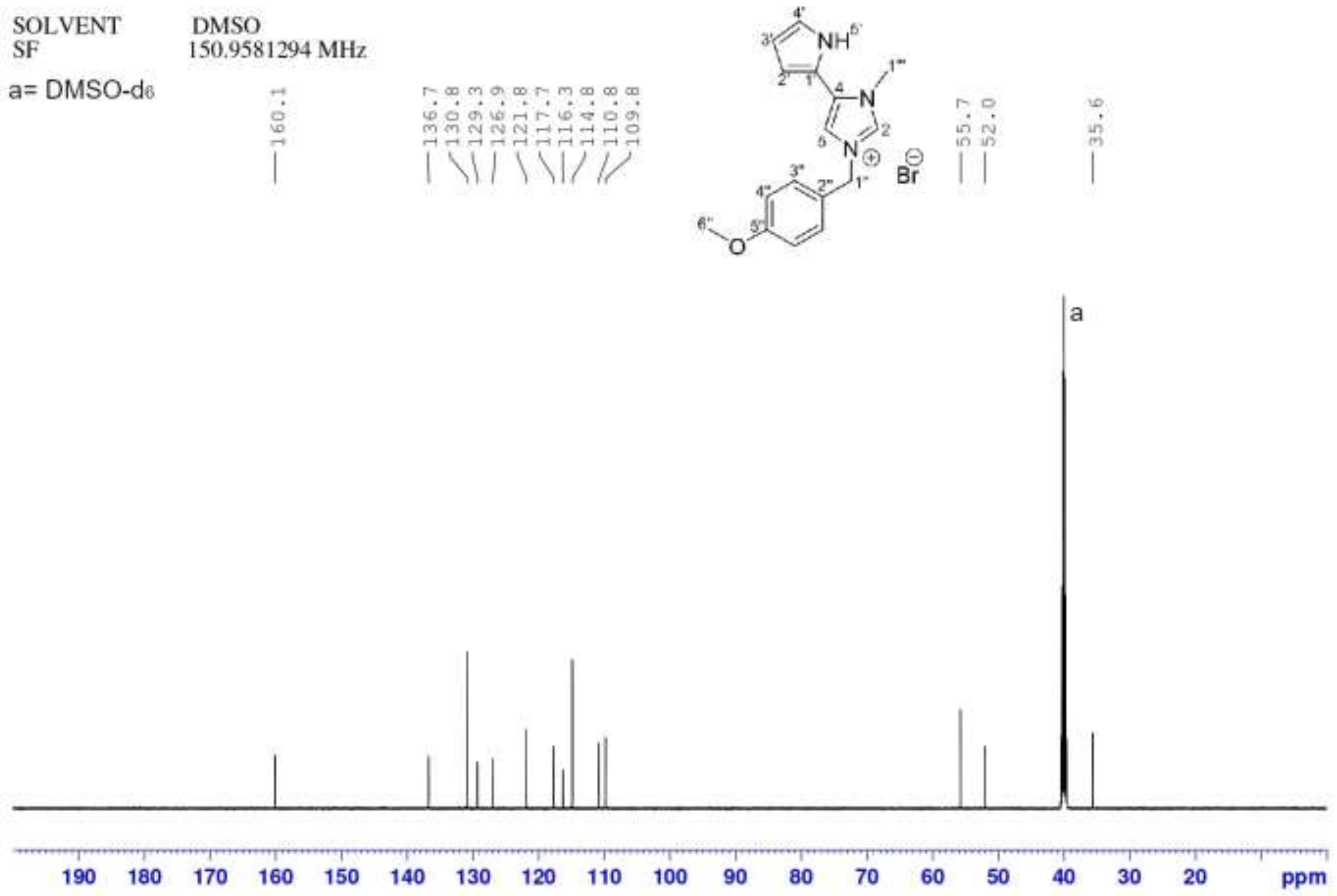

Figure S58. **$^{13}C\{^{1}H\}$-NMR of compound 7g**

$^{13}C\{^{1}H\}$-DEPT-NMR 1-(4-Methoxybenzyl)-3-methyl-4-(1H-pyrrol-2-yl)-1H-imidazol-3-ium bromide (7g):

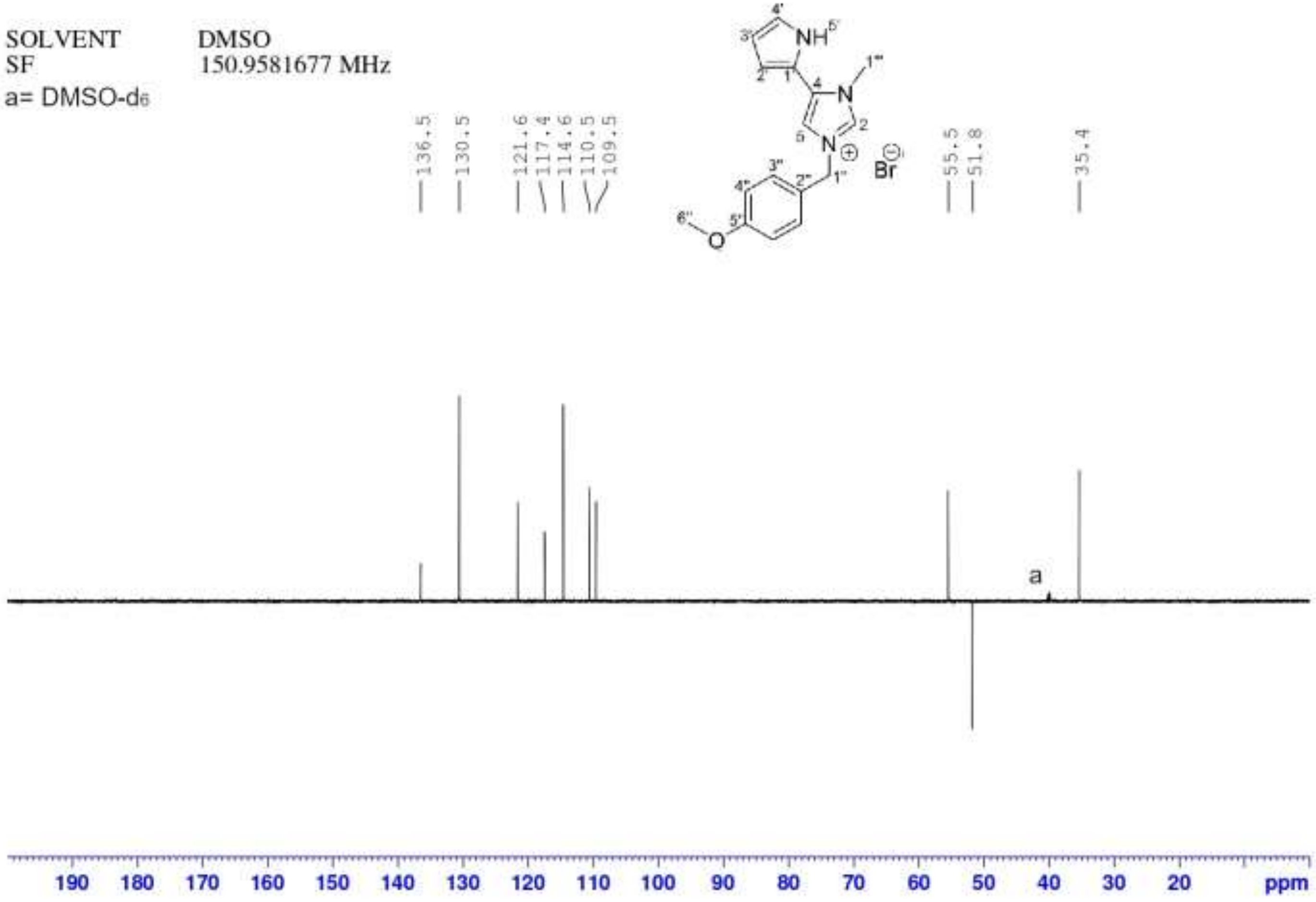

Figure S59. $^{13}C\{^{1}H\}$**-DEPT-NMR of compound 7g**

HSQC-NMR 3-(4-Methoxybenzyl)-1-methyl-4-(1H-pyrrol-2-yl)-1H-imidazol-3-ium bromide (7g):

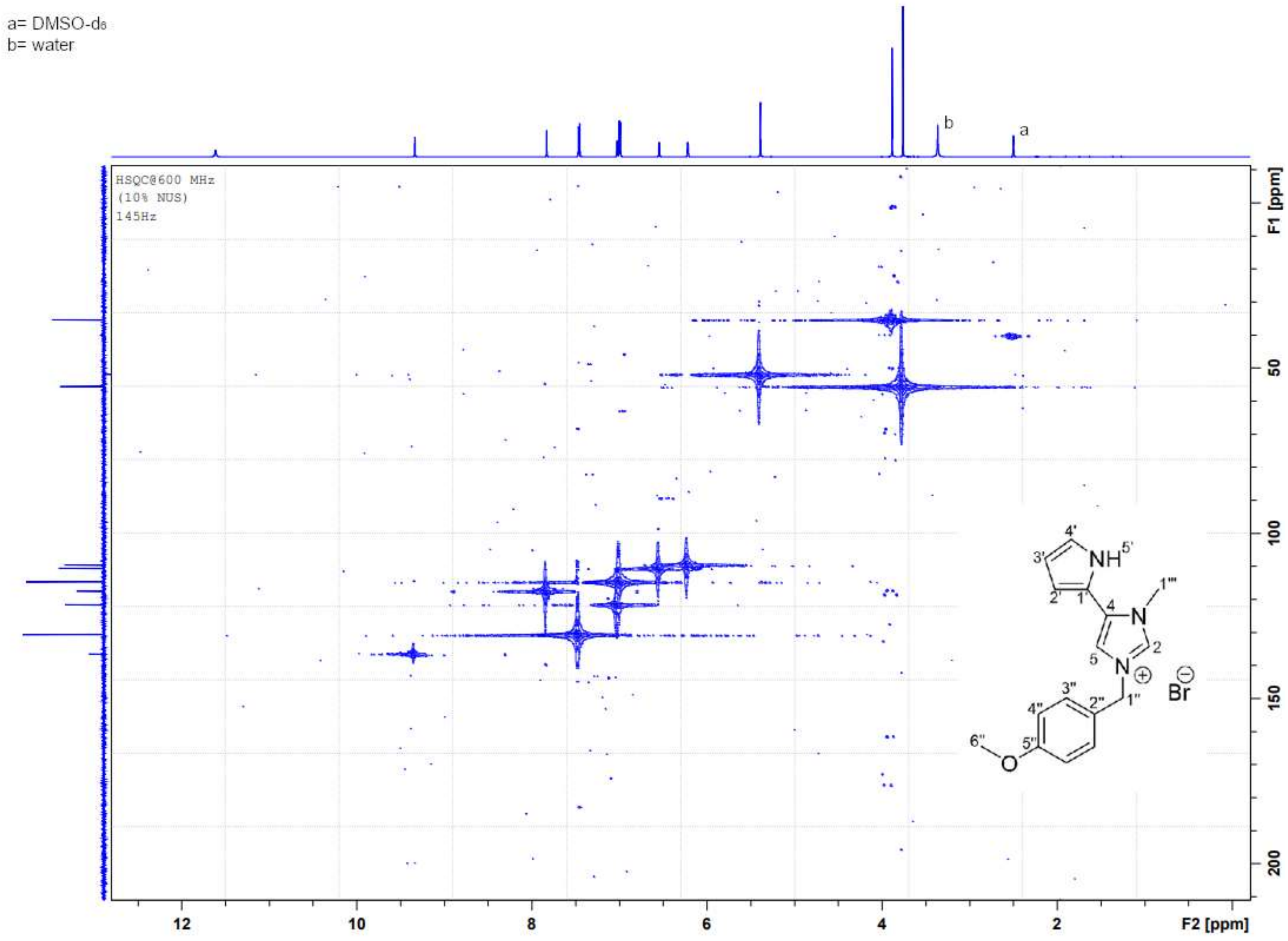

Figure S60. HSQC-NMR of compound 7g

HMBC-NMR 3-(4-Methoxybenzyl)-1-methyl-4-(1H-pyrrol-2-yl)-1H-imidazol-3-ium bromide (7g):

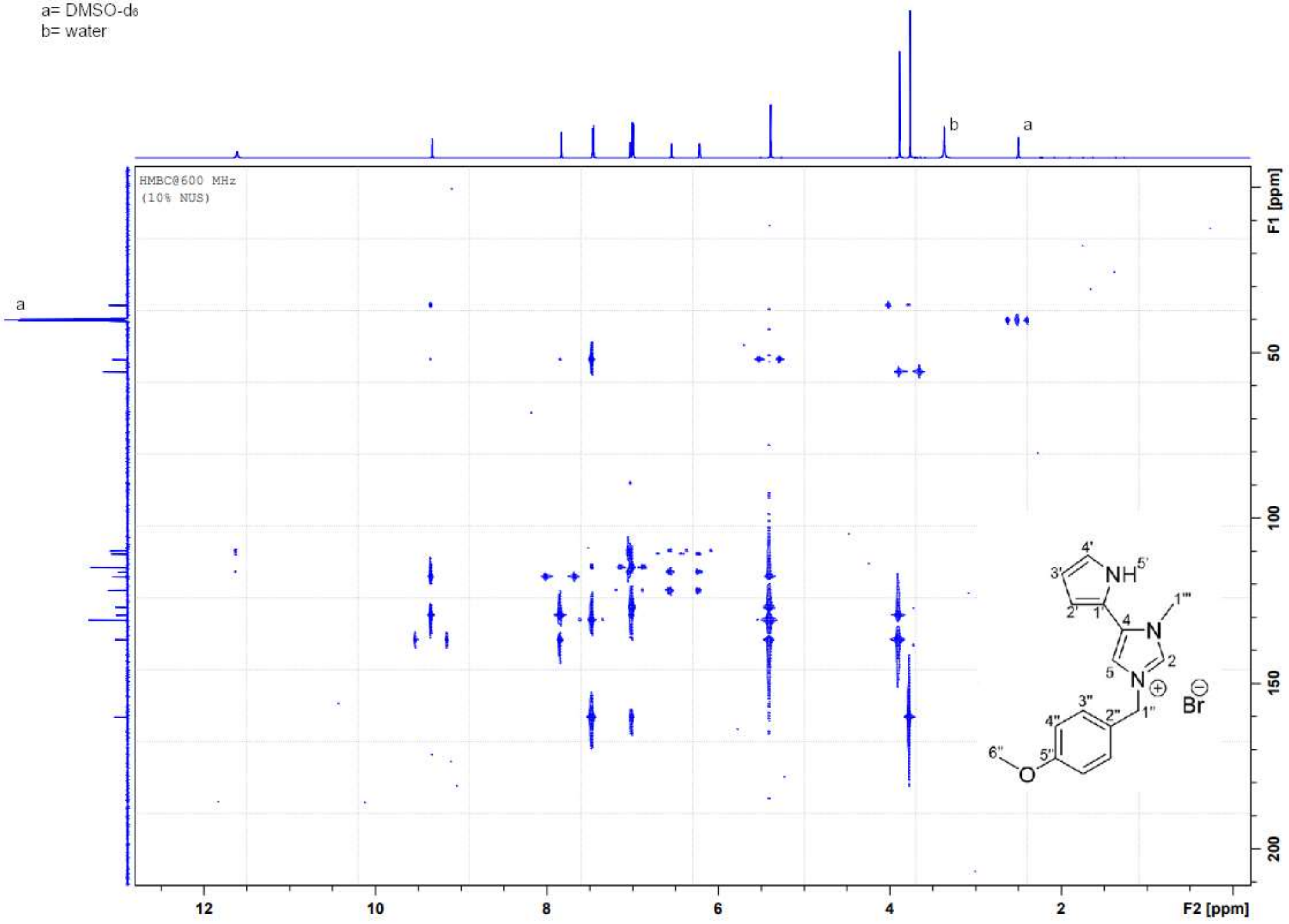

Figure S61. HMBC-NMR of compound 7g

^{1}H-NMR 1-Benzyl-3-(tert-butyl)-4-(1H-pyrrol-2-yl)-1H-imidazol-3-ium, bromide (7h):

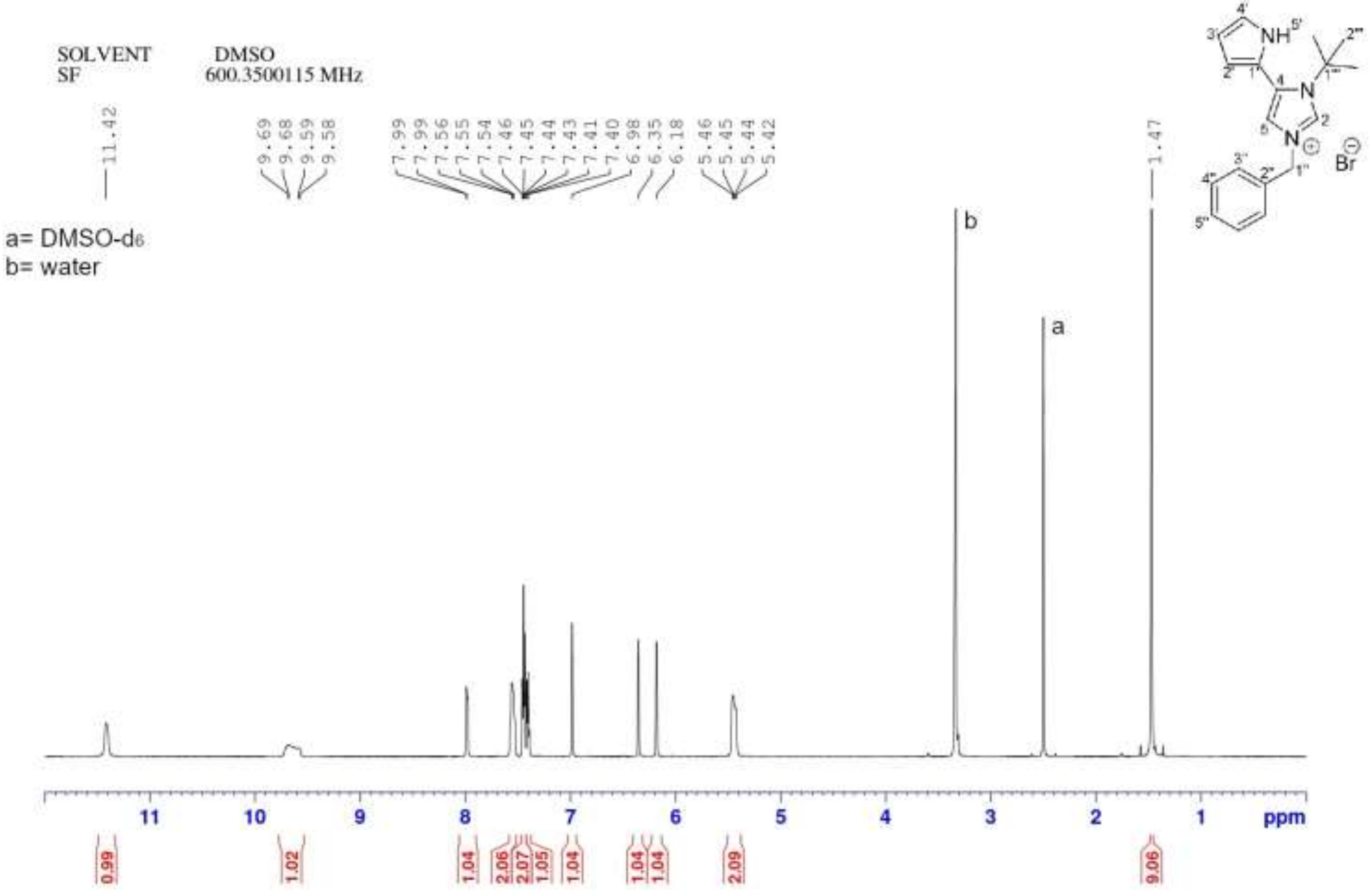

Figure S62. **^{1}H-NMR of compound 7h**

$^{13}C\{^1H\}$-NMR 1-Benzyl-3-(tert-butyl)-4-(1H-pyrrol-2-yl)-1H-imidazol-3-ium, bromide (7h):

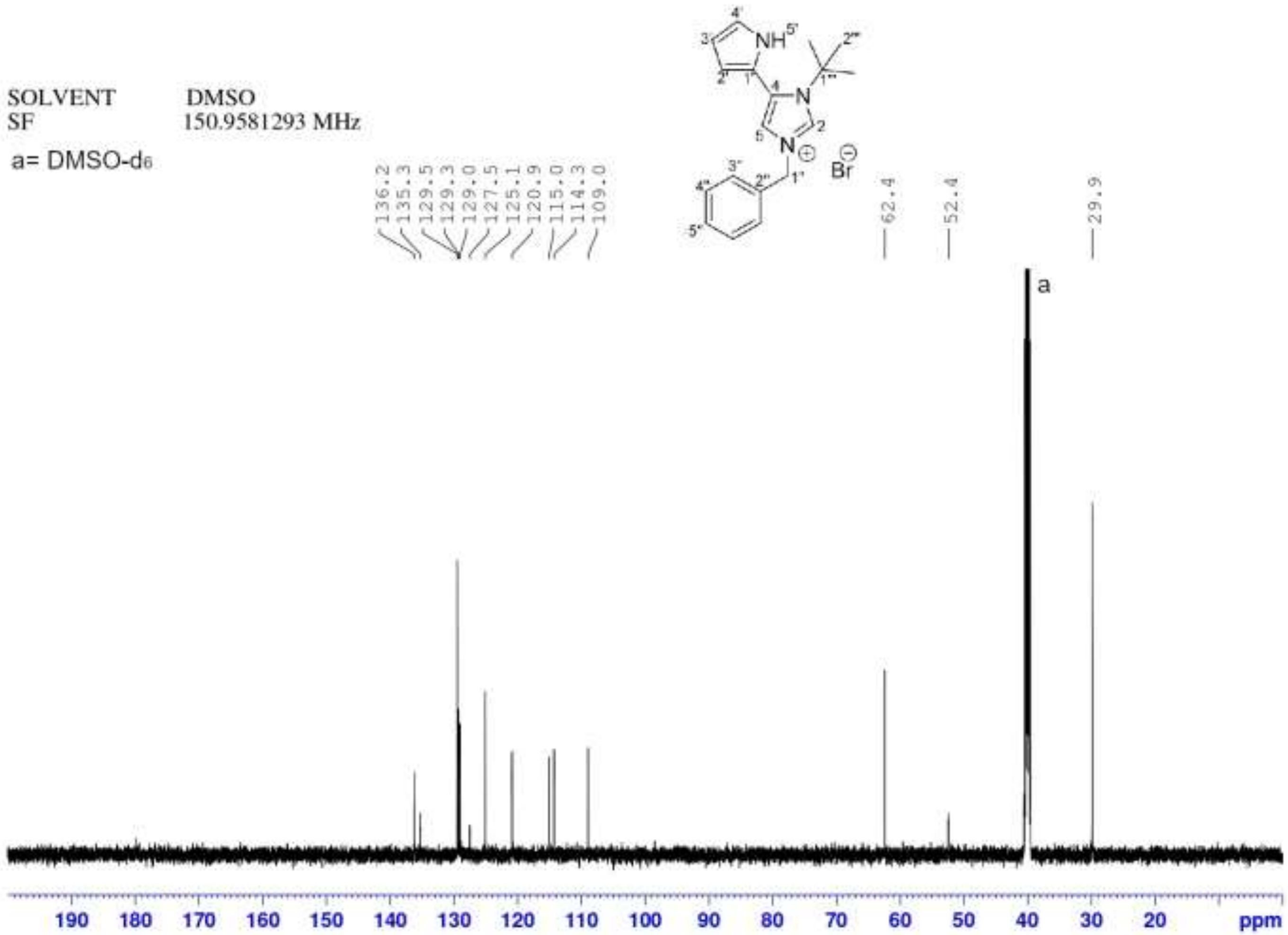

Figure S63. $^{13}C\{^1H\}$**-NMR of compound 7h**

^{13}C{^{1}H}-DEPT-NMR 1-Benzyl-3-(tert-butyl)-4-(1H-pyrrol-2-yl)-1H-imidazol-3-ium, bromide (7h):

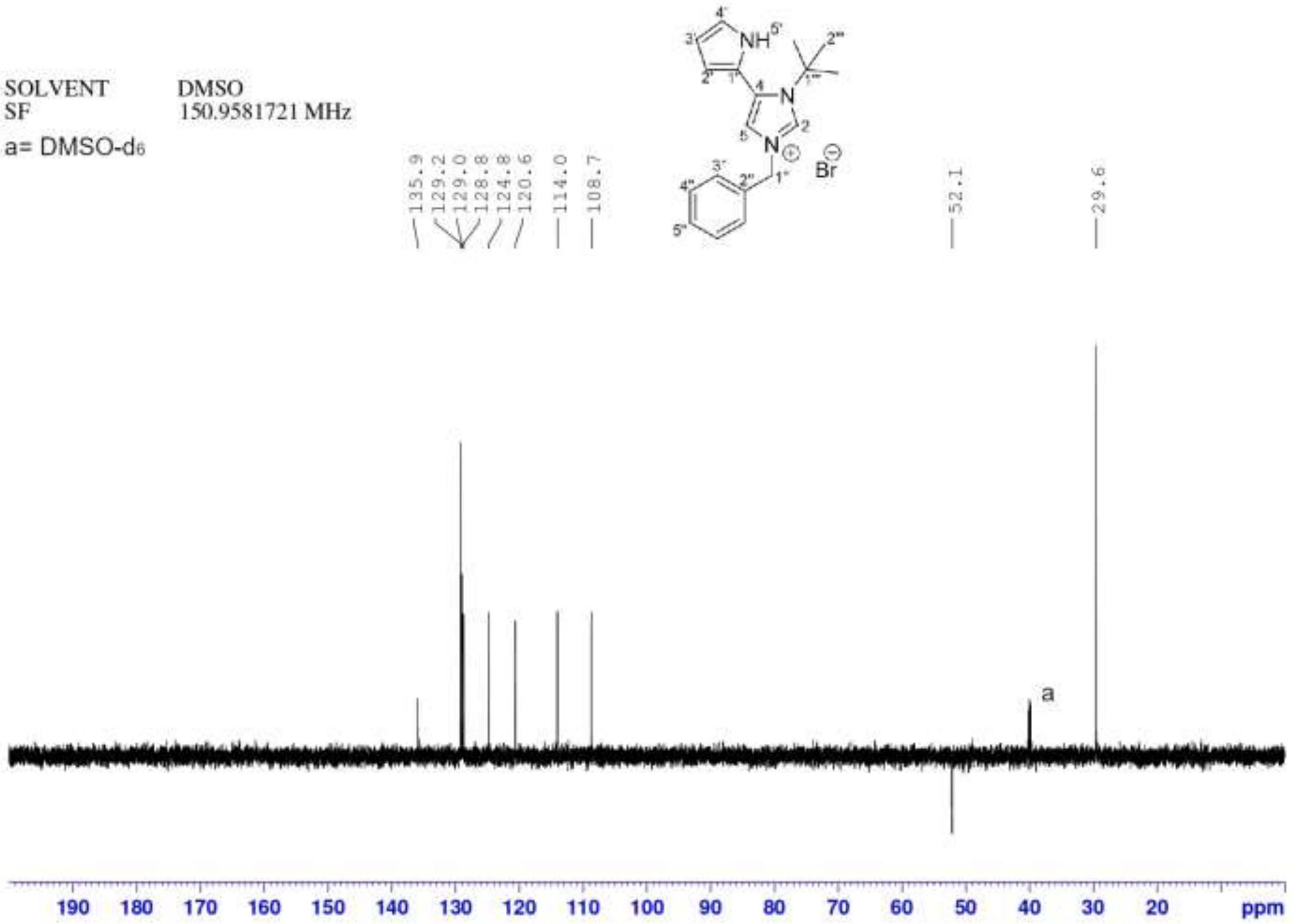

Figure S64. $^{13}C\{^1H\}$**-DEPT-NMR of compound 7h**

HSQC-NMR 1-Benzyl-3-(tert-butyl)-4-(1H-pyrrol-2-yl)-1H-imidazol-3-ium, bromide (7h):

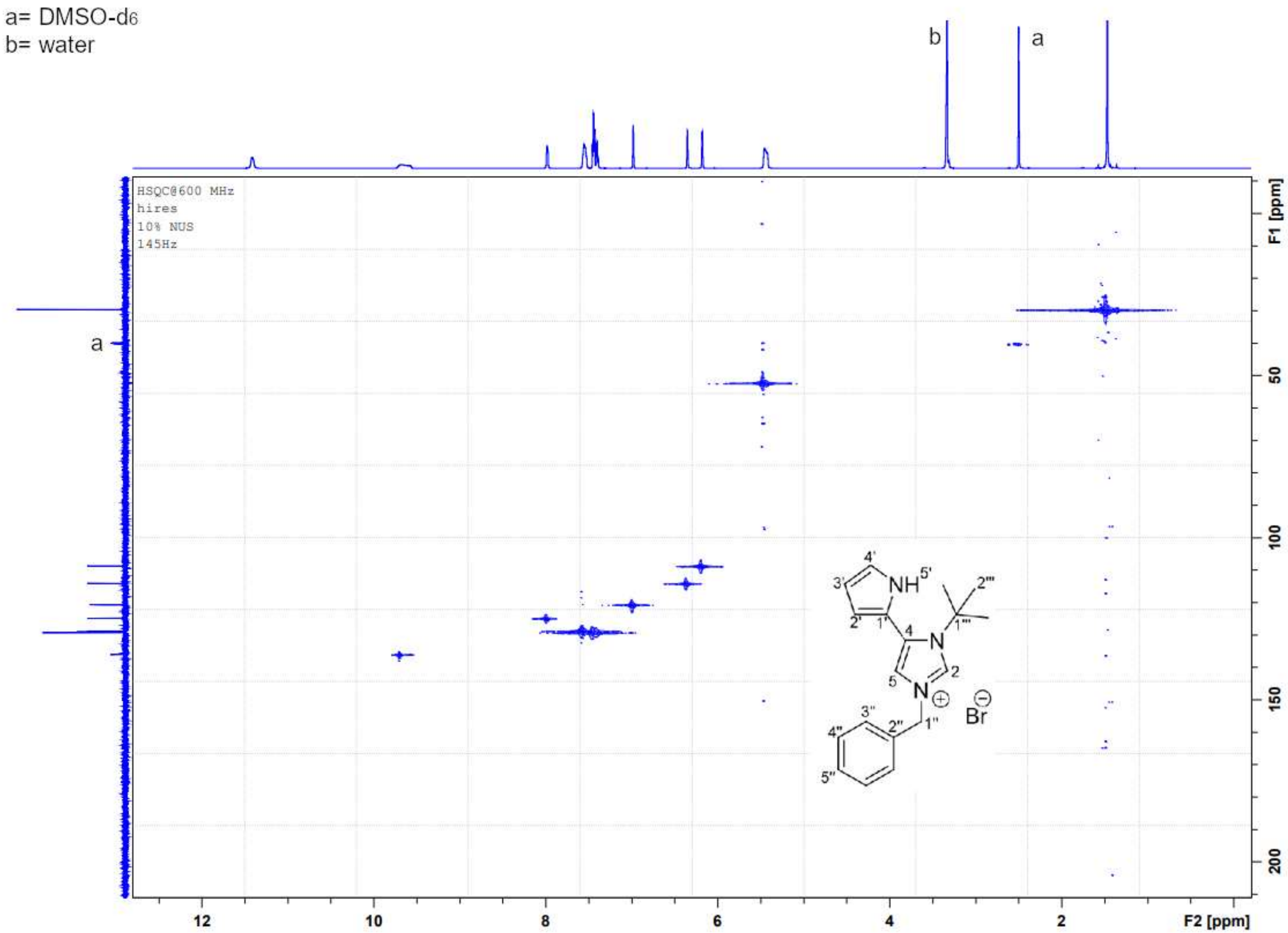

Figure S65. HSQC-NMR of compound 7h

HMBC-NMR 1-Benzyl-3-(tert-butyl)-4-(1H-pyrrol-2-yl)-1H-imidazol-3-ium, bromide (7h):

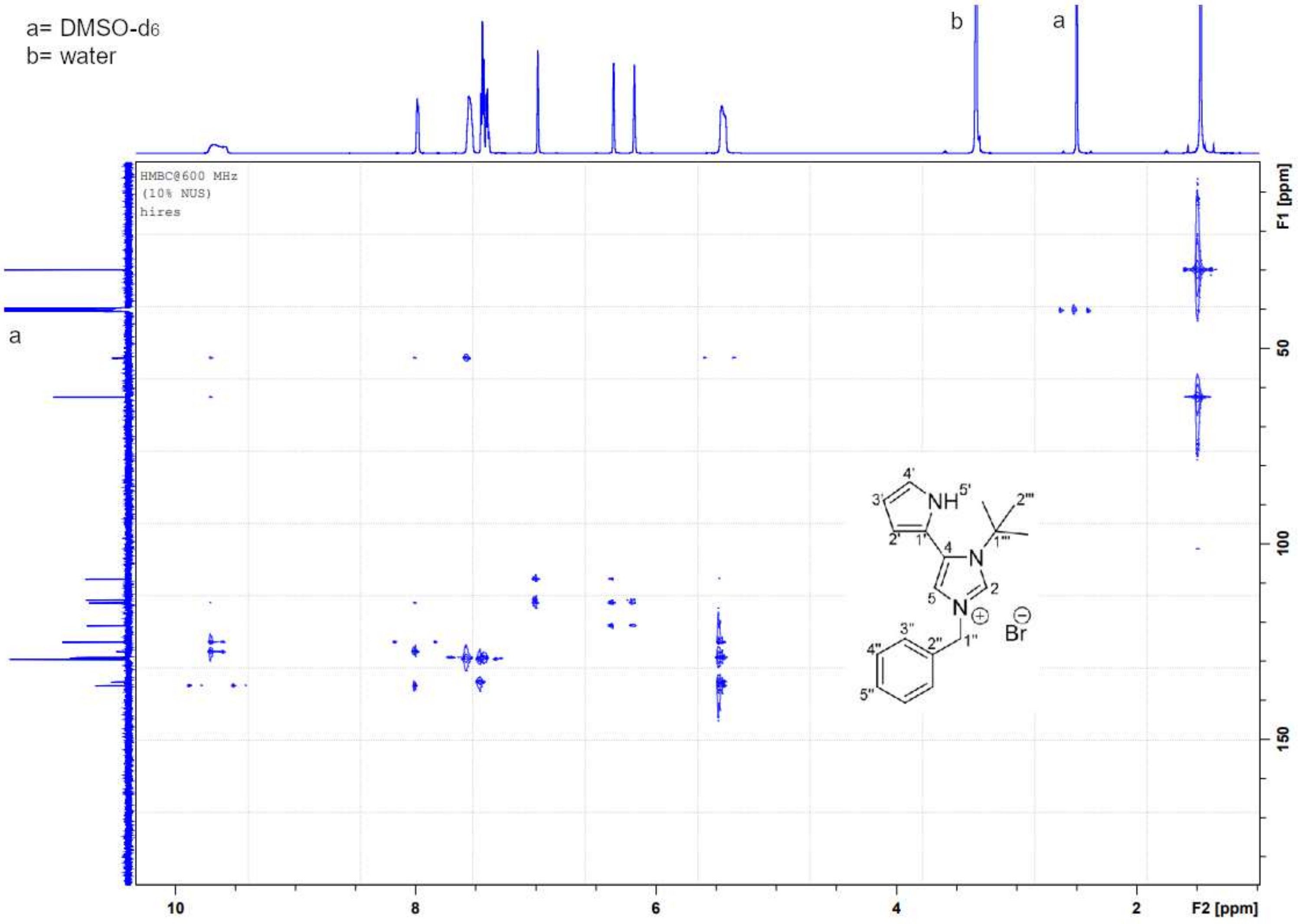

Figure S66. HMBC-NMR of compound 7h

^{1}H-NMR 3-(tert-Butyl)-1-(4-methylbenzyl)-4-(1H-pyrrol-2-yl)-1H-imidazol-3-ium bromide (7i):

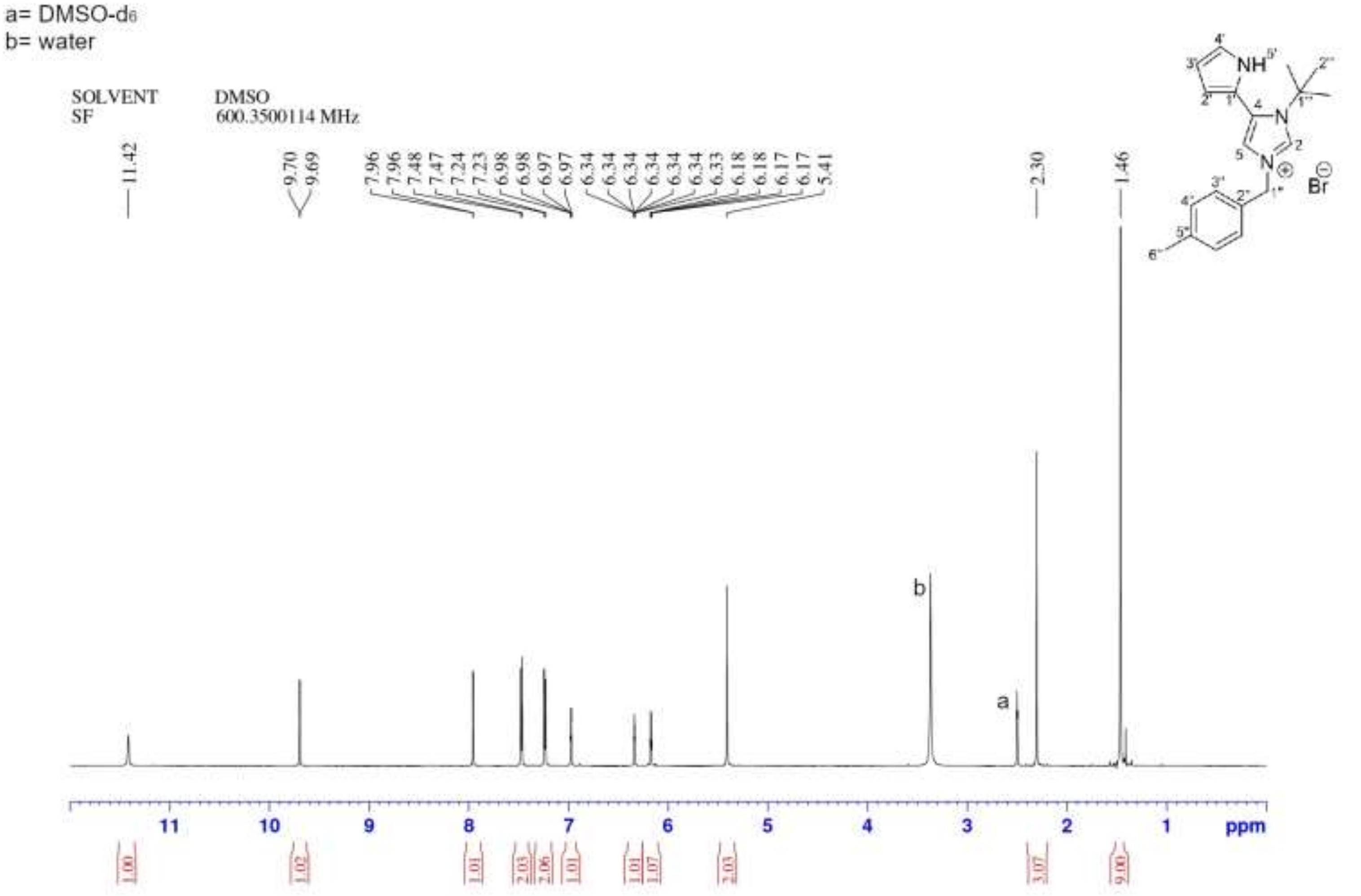

Figure S67. **^{1}H-NMR of compound 7i**

$^{13}C\{^1H\}$-NMR 3-(tert-Butyl)-1-(4-methylbenzyl)-4-(1H-pyrrol-2-yl)-1H-imidazol-3-ium bromide (7i):

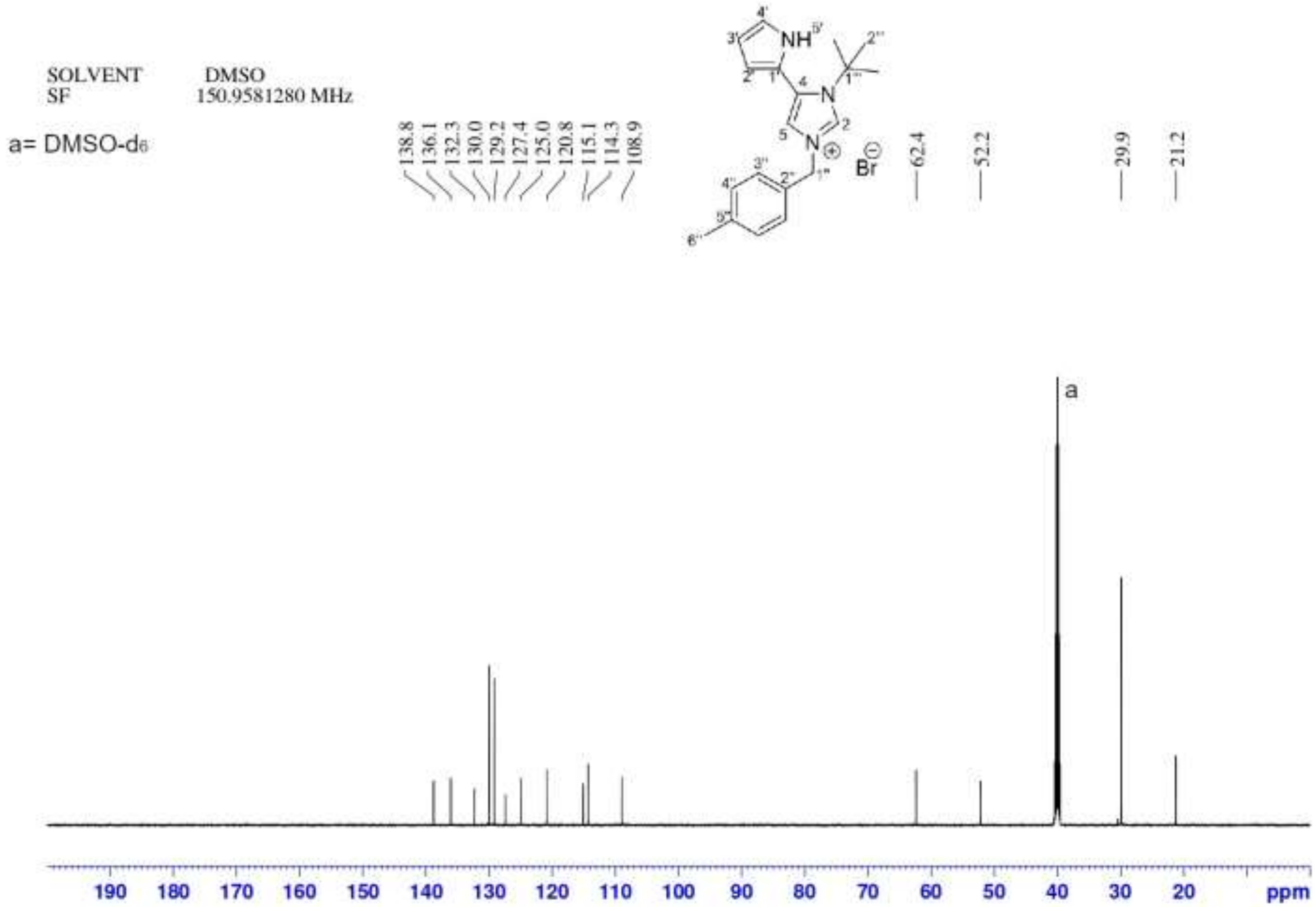

Figure S68. **$^{13}C\{^1H\}$-NMR of compound 7i**

$^{13}C\{^1H\}$-DEPT-NMR 3-(tert-Butyl)-1-(4-methylbenzyl)-4-(1H-pyrrol-2-yl)-1H-imidazol-3-ium bromide (7i):

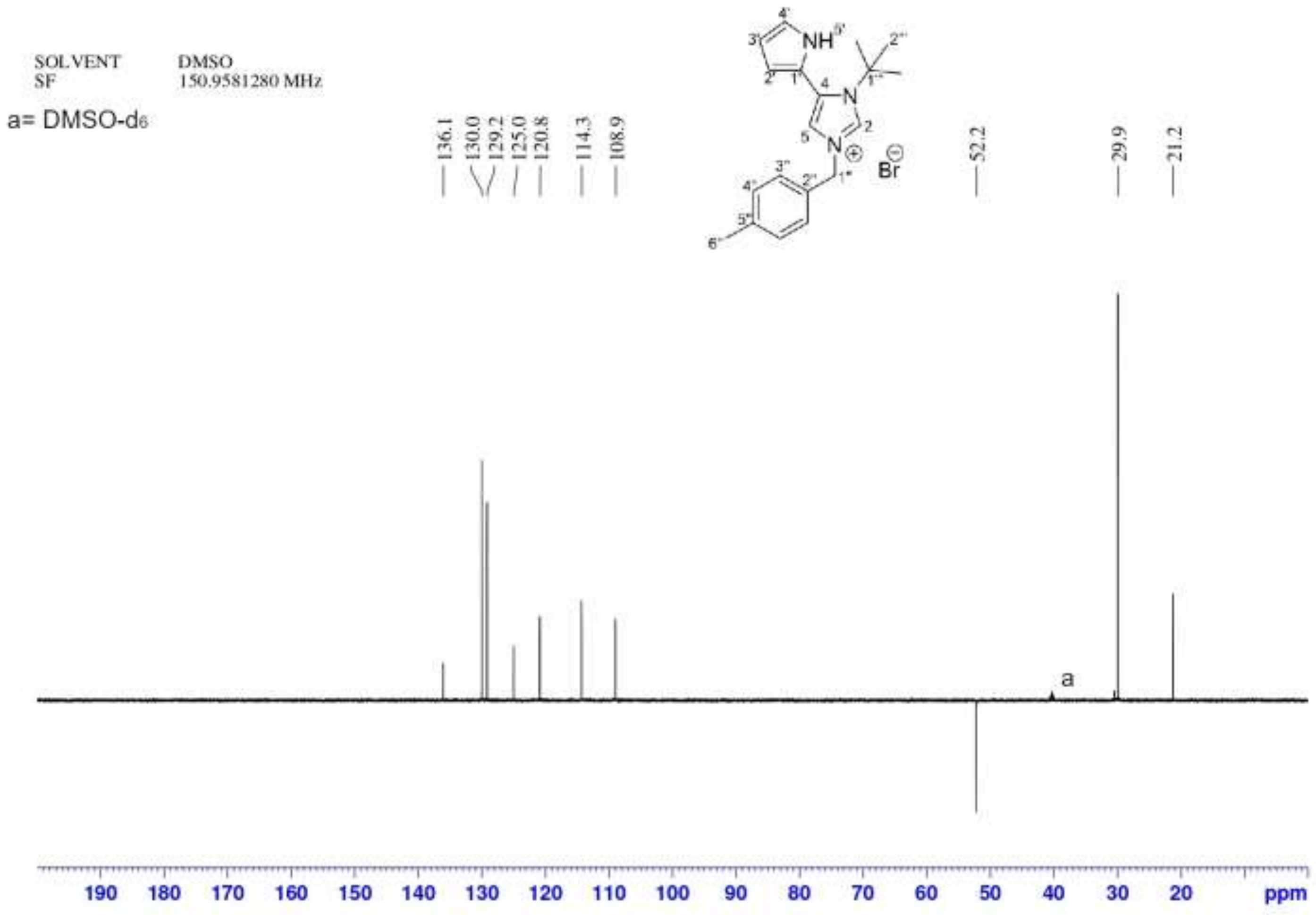

Figure S69. $^{13}C\{^1H\}$**-DEPT-NMR of compound 7i**

HSQC-NMR 3-(tert-Butyl)-1-(4-methylbenzyl)-4-(1H-pyrrol-2-yl)-1H-imidazol-3-ium bromide (7i):

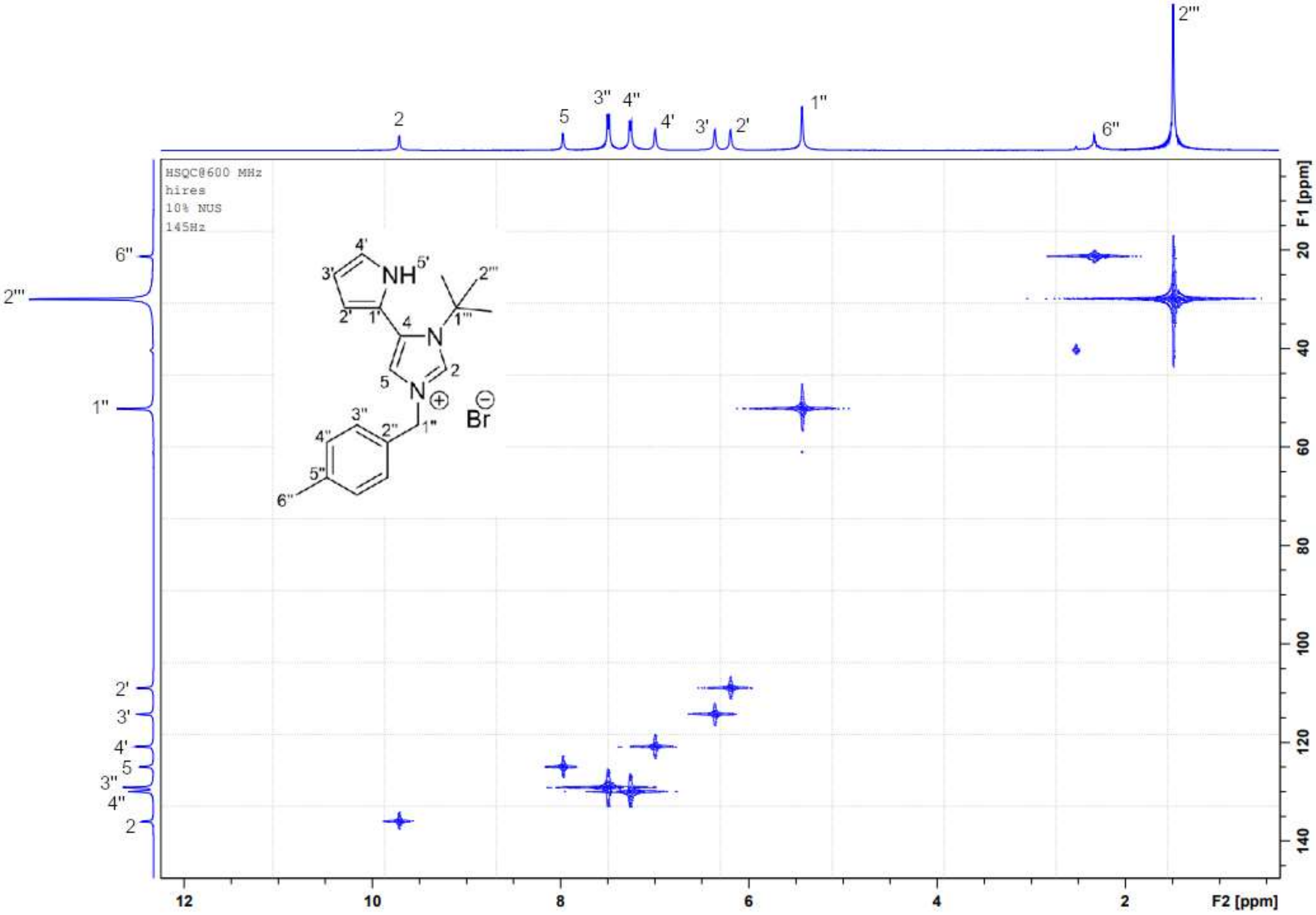

Figure S70. HSQC-NMR of compound 7i

HMBC-NMR 3-(tert-Butyl)-1-(4-methylbenzyl)-4-(1H-pyrrol-2-yl)-1H-imidazol-3-ium bromide (7i):

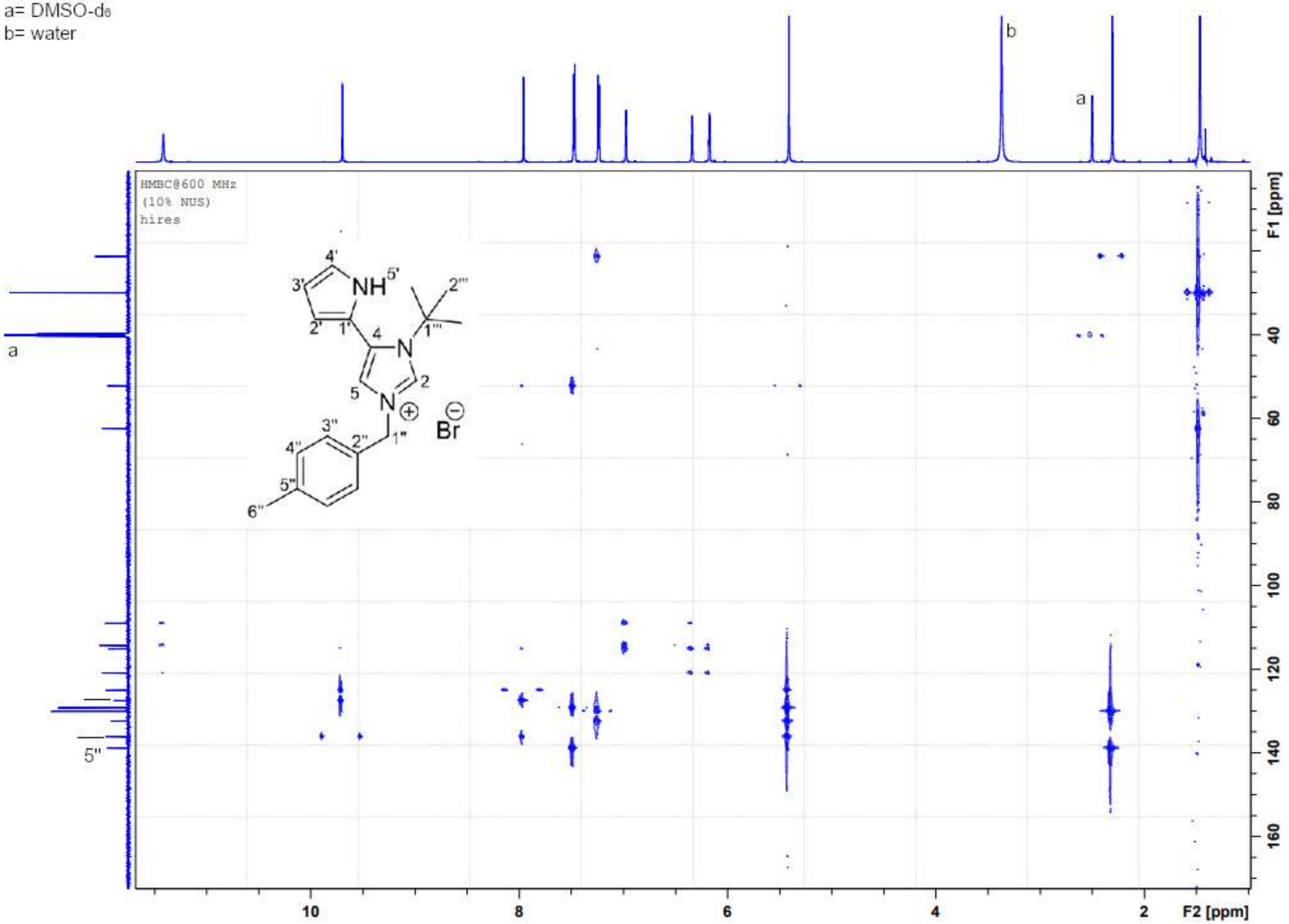

Figure S71. HMBC-NMR of compound 7i

^{1}H-NMR 2-(1,3-Dimethyl-1H-imidazol-3-ium-4-yl)pyrrol-1-ide (8a):

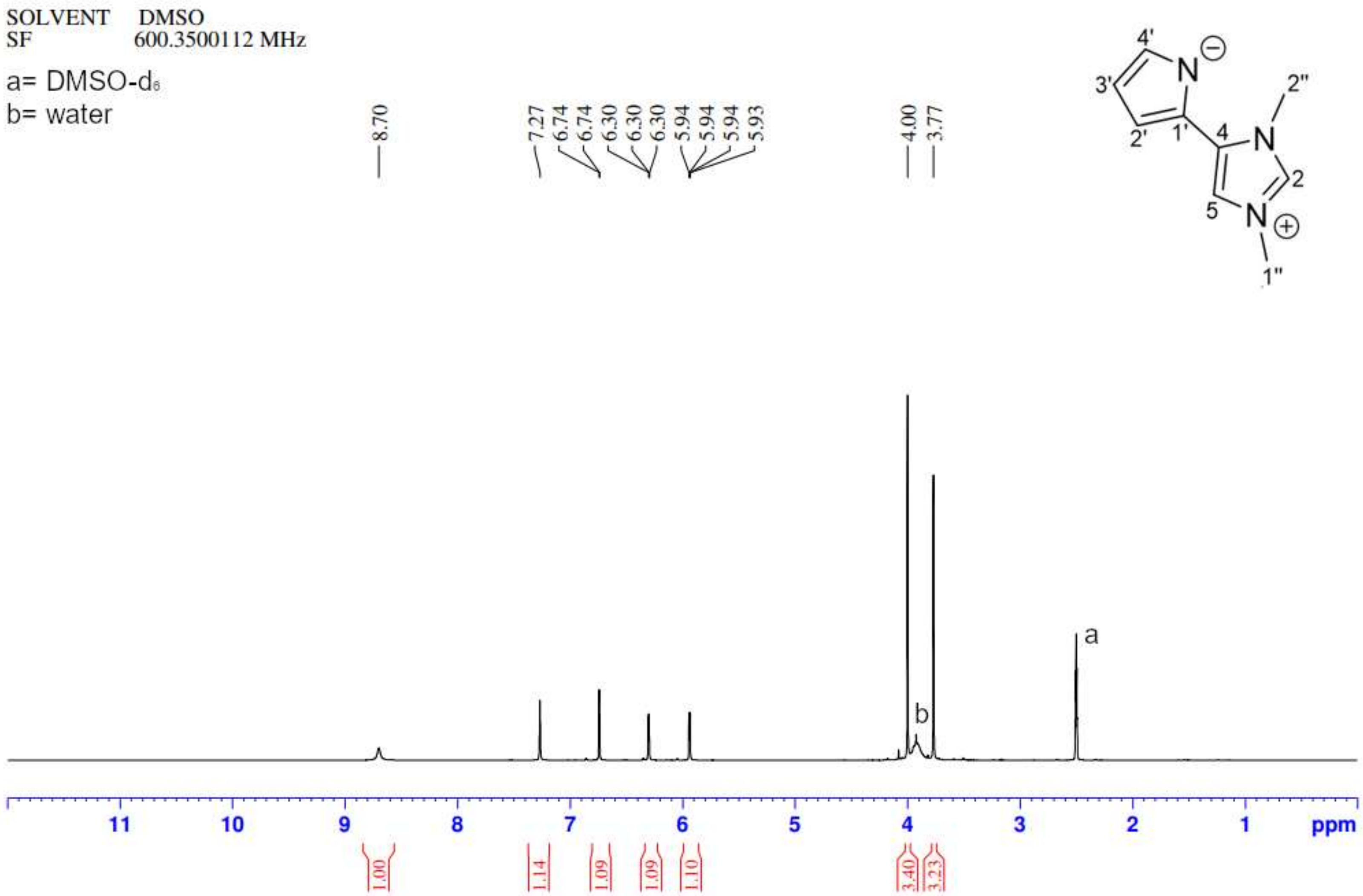

Figure S72. ^{1}H-NMR of compound 8a

$^{13}C\{^1H\}$-NMR 2-(1,3-Dimethyl-1H-imidazol-3-ium-4-yl)pyrrol-1-ide (8a):

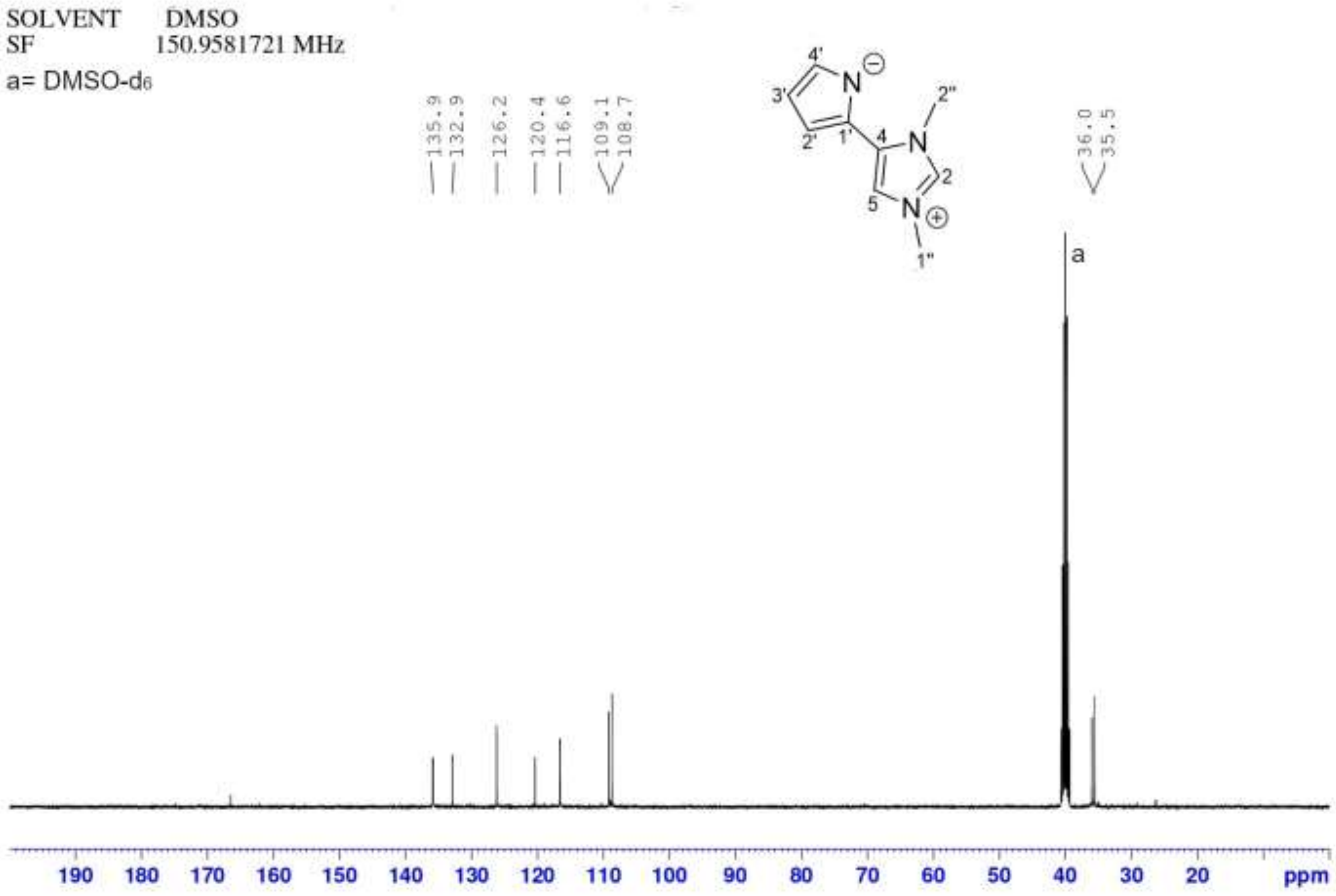

Figure S73. **$^{13}C\{^1H\}$-NMR of compound 8a**

$^{13}C\{^1H\}$-DEPT-NMR 2-(1,3-Dimethyl-1H-imidazol-3-ium-4-yl)pyrrol-1-ide (8a):

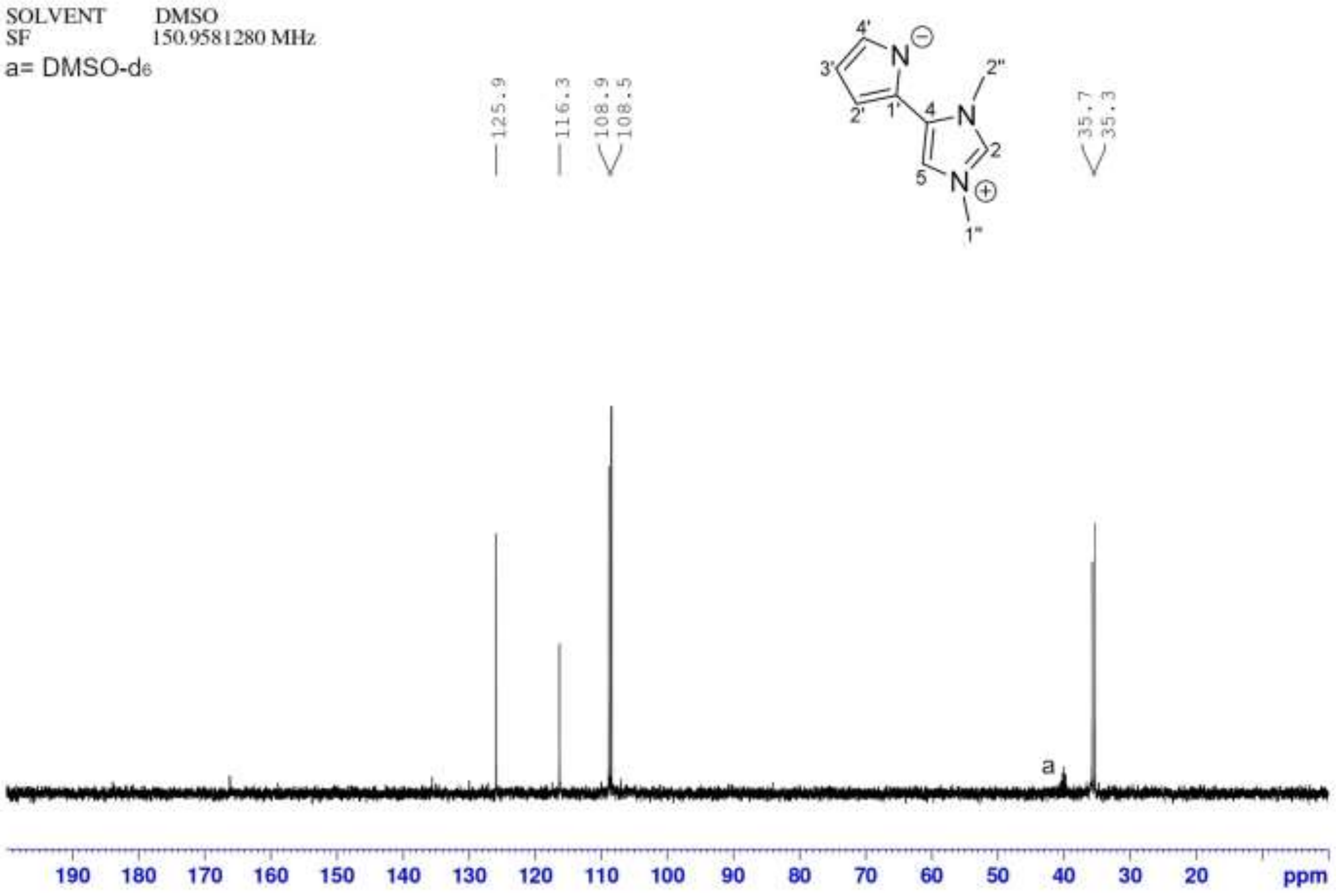

Figure S74. **$^{13}C\{^1H\}$-DEPT-NMR of compound 8a**

HSQC-NMR 2-(1,3-Dimethyl-1H-imidazol-3-ium-4-yl)pyrrol-1-ide (8a):

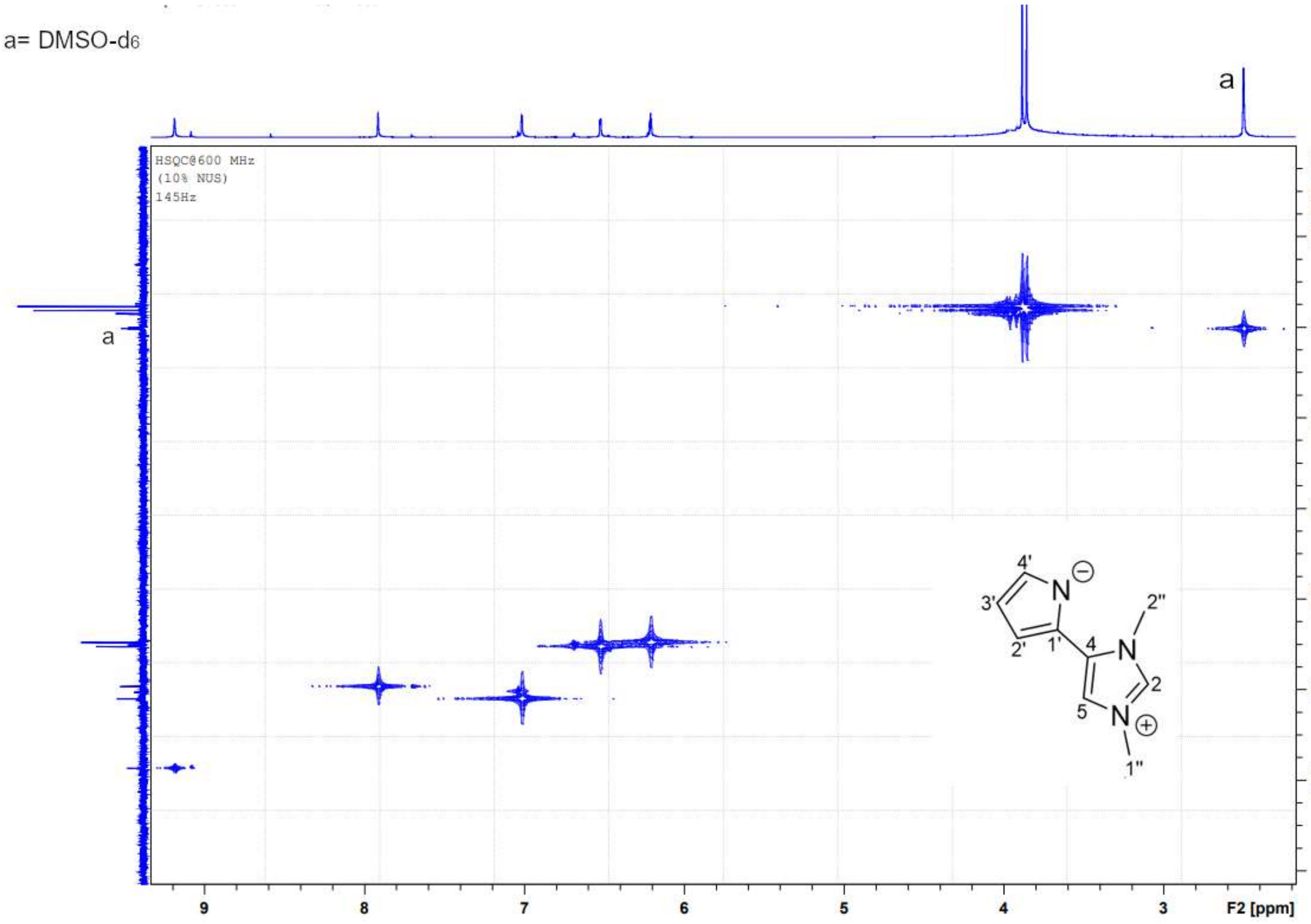

Figure S75. HSQC-NMR of compound 8a

HMBC-NMR 2-(1,3-Dimethyl-1H-imidazol-3-ium-4-yl)pyrrol-1-ide (8a):

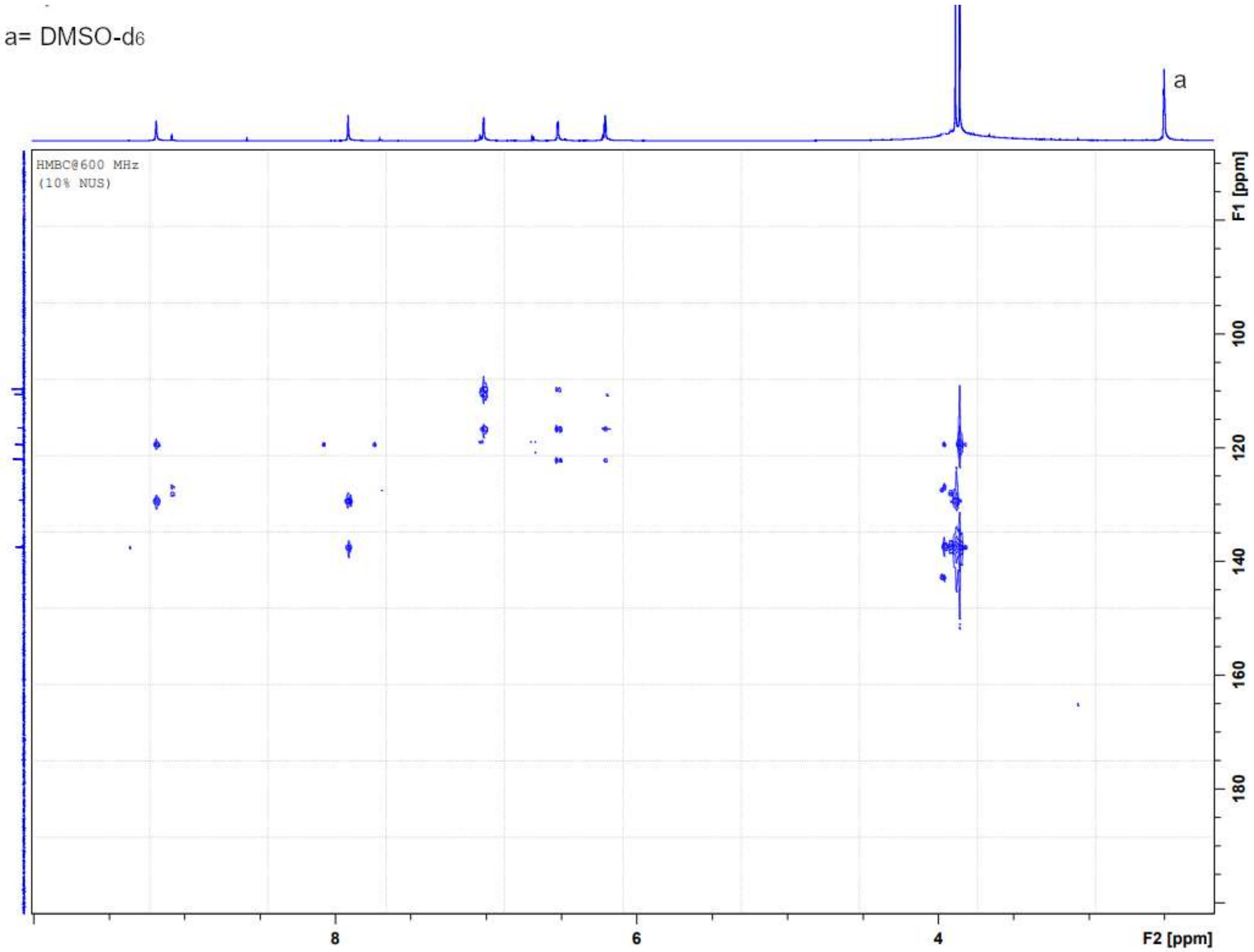

Figure S76. HMBC-NMR of compound 8a

^{1}H-NMR (2-(5-Benzyl-1,3-dimethyl-1H-imidazol-3-ium-4-yl)pyrrol-1-ide) (8b):

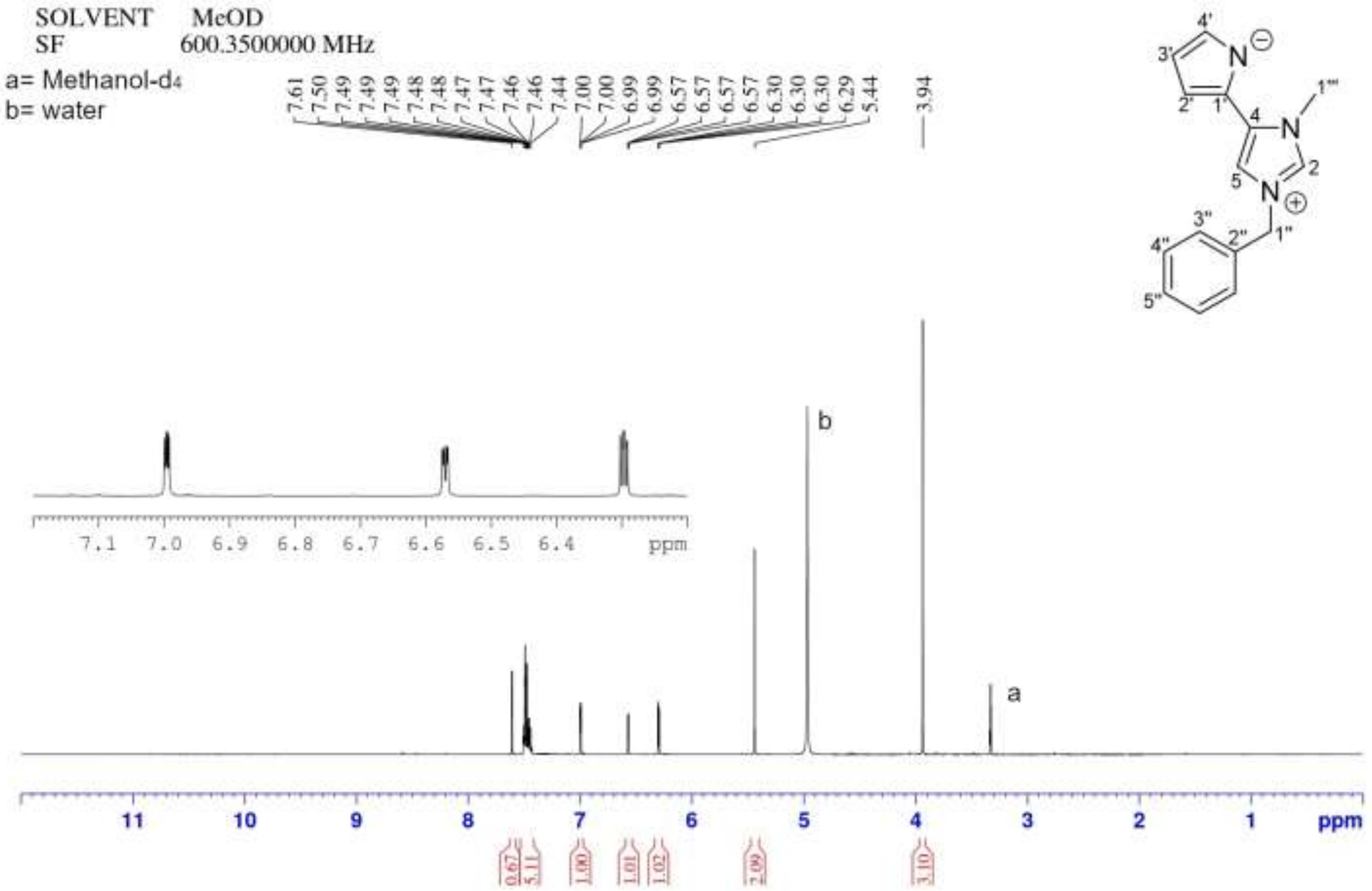

Figure S77. **^{1}H-NMR of compound 8b**

$^{13}C\{^1H\}$-NMR (2-(5-Benzyl-1,3-dimethyl-1H-imidazol-3-ium-4-yl)pyrrol-1-ide) (8b):

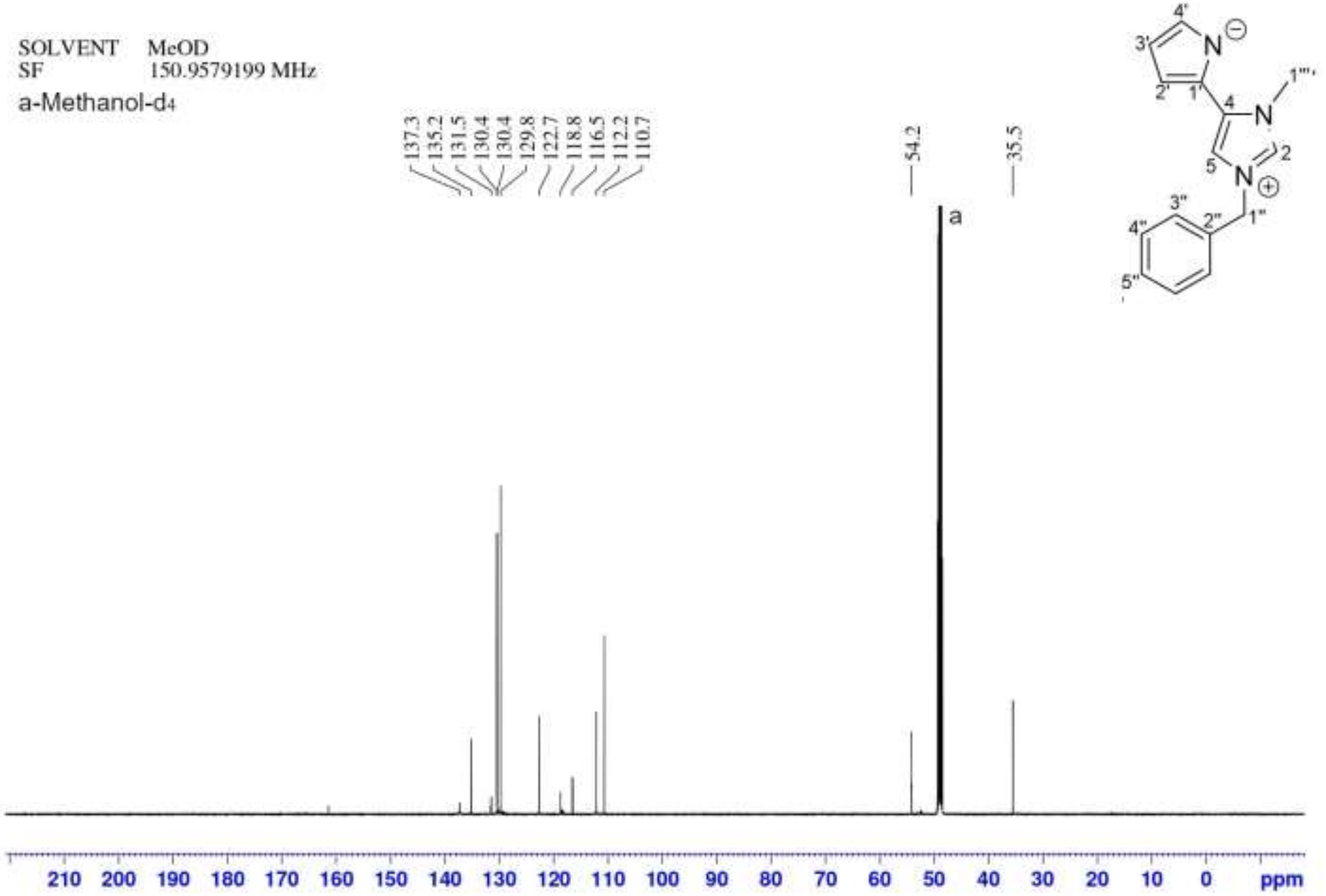

Figure S79. $^{13}C\{^1H\}$-NMR of compound 8b

^{13}C{^{1}H}-DEPT-NMR (2-(5-Benzyl-1,3-dimethyl-1H-imidazol-3-ium-4-yl)pyrrol-1-ide) (8b):

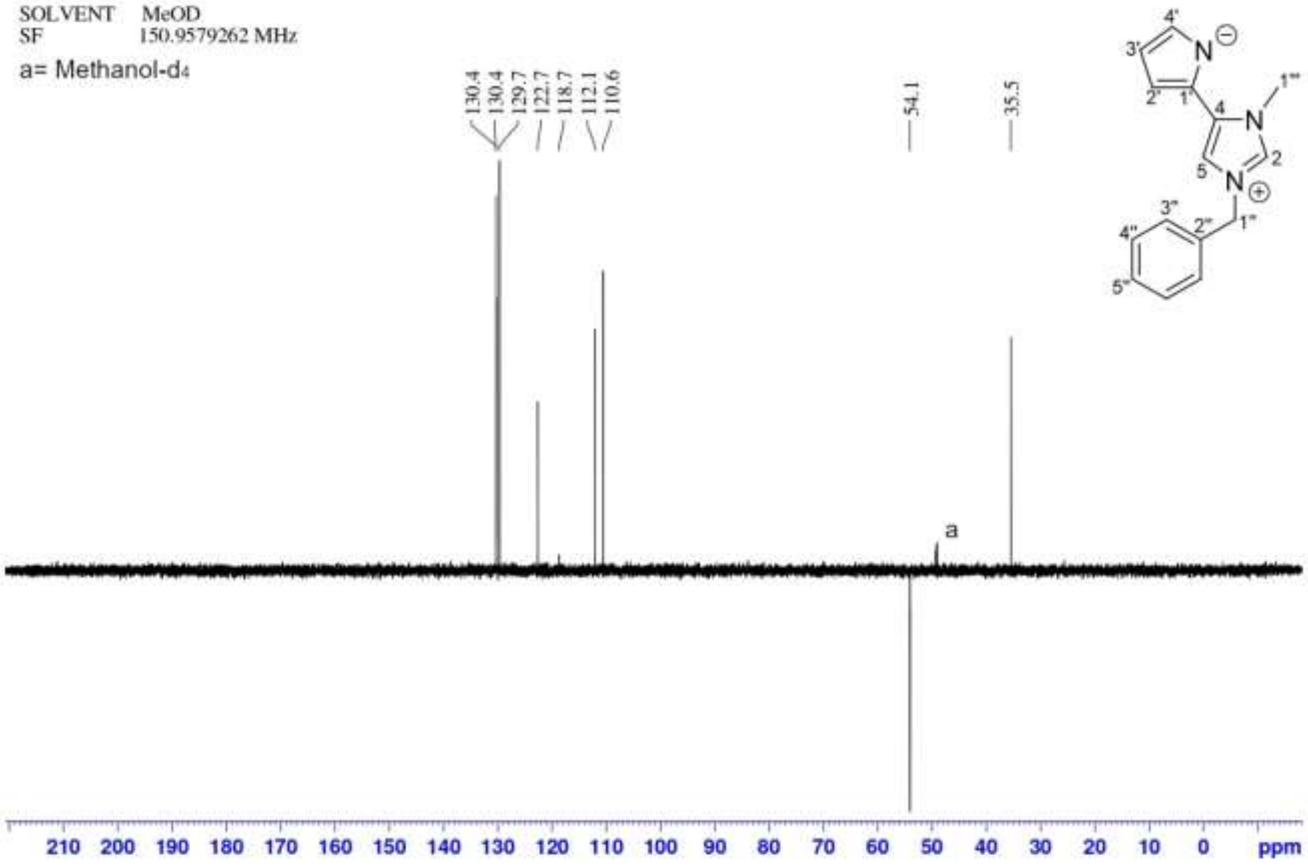

Figure S80. ^{13}C{^{1}H}-DEPT-NMR of compound 8b

HSQC-NMR (2-(5-Benzyl-1,3-dimethyl-1H-imidazol-3-ium-4-yl)pyrrol-1-ide) (8b):

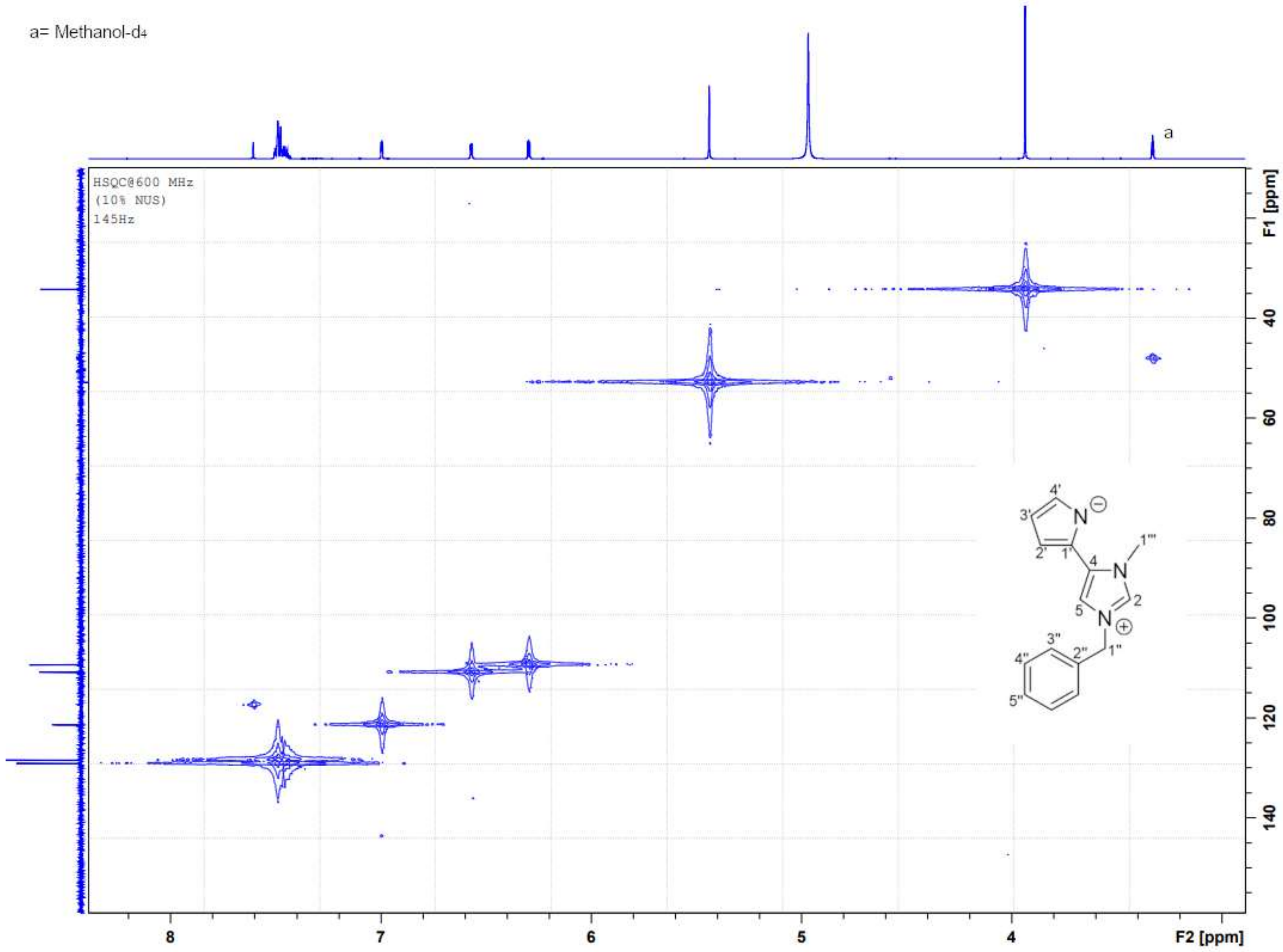

Figure S81. HSQC-NMR of compound 8b

HMBC-NMR (2-(5-Benzyl-1,3-dimethyl-1H-imidazol-3-ium-4-yl)pyrrol-1-ide) (8b):

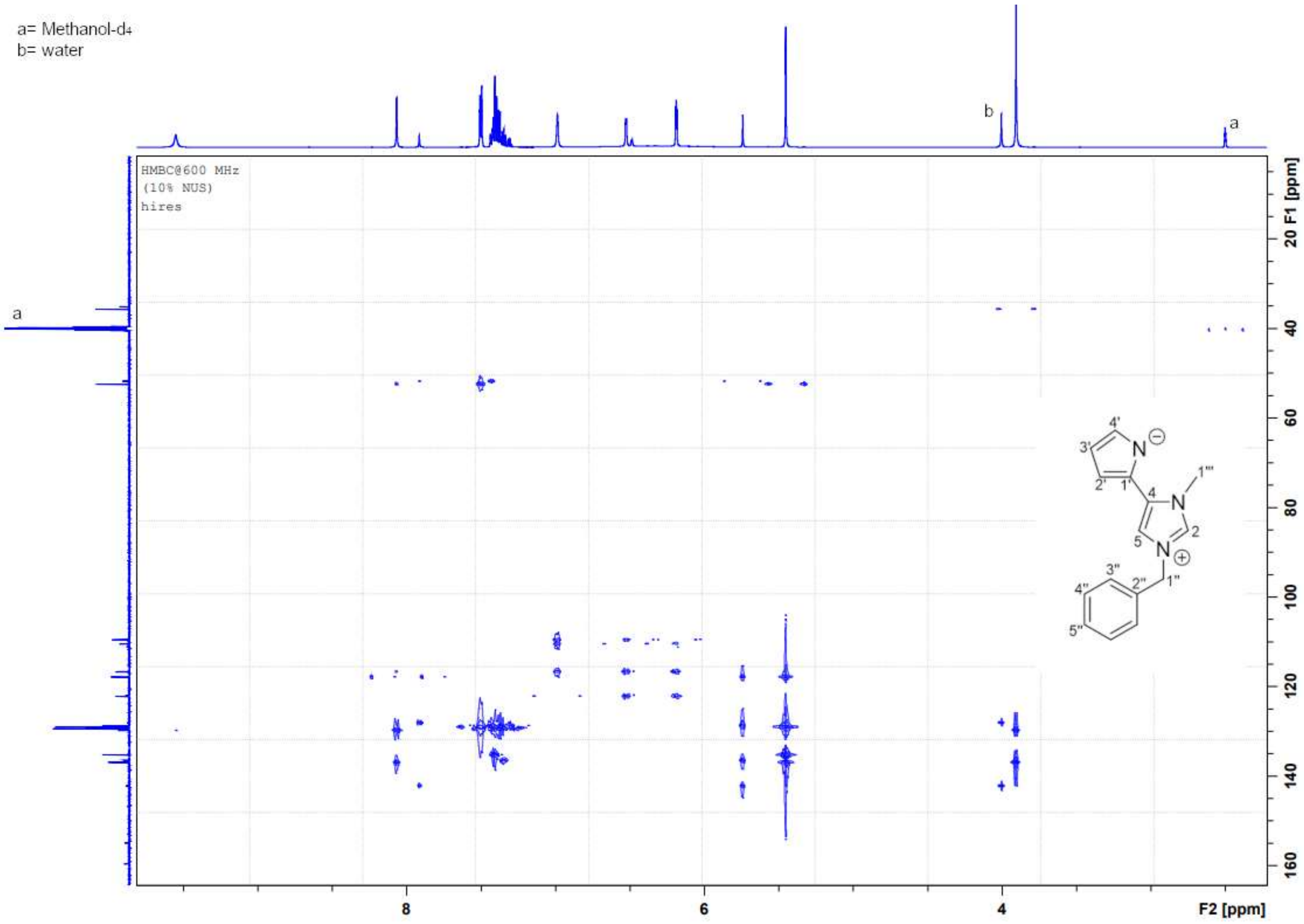

Figure S82. HMBC-NMR of compound 8b

^{1}H-NMR 2-(1,3-Dimethyl-5-(p-tolyl)-1H-imidazol-3-ium-4-yl)pyrrol-1-ide (8c):

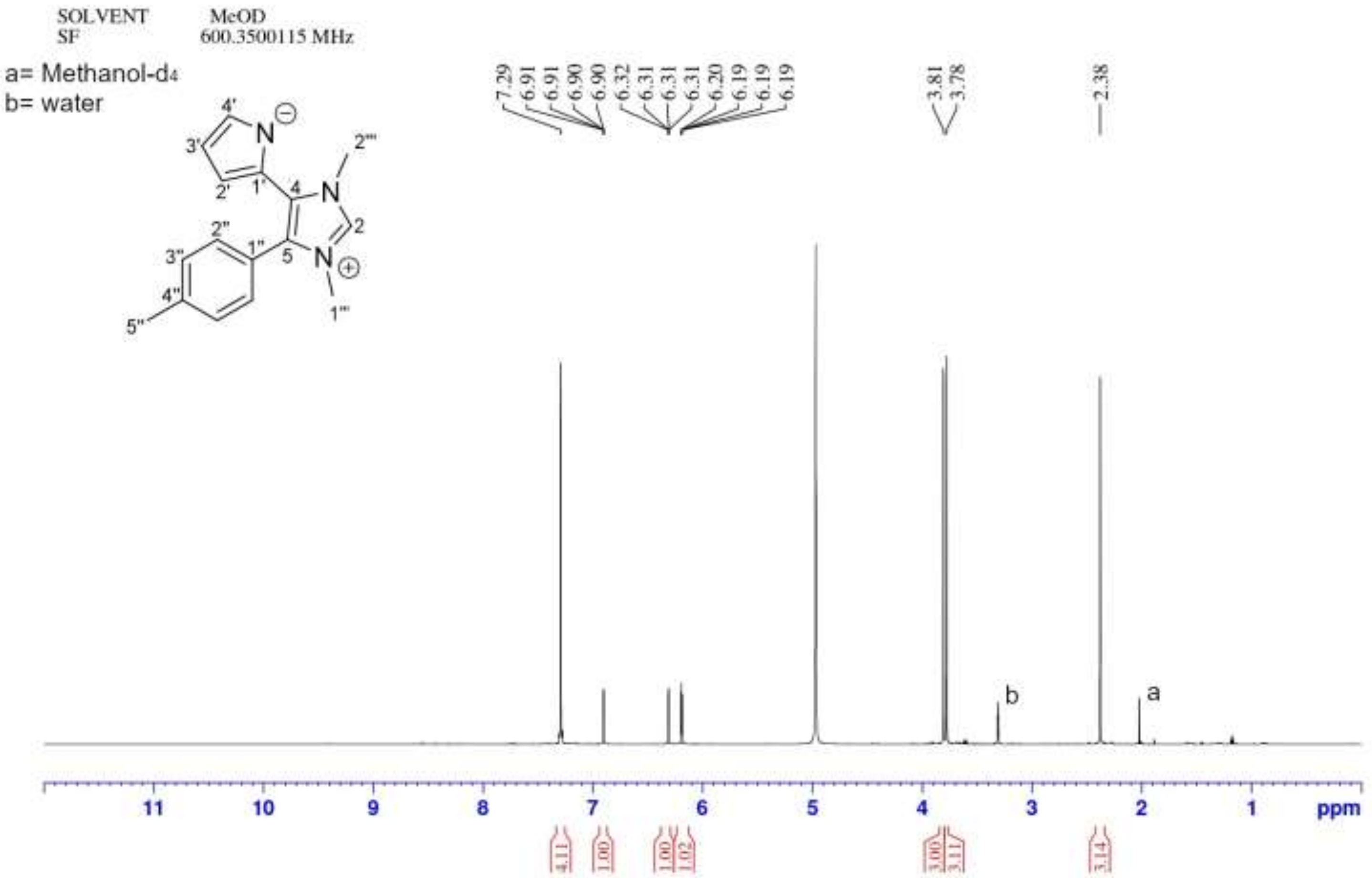

Figure S83. ^{1}H-NMR of compound 8c

²H-NMR 2-(1,3-Dimethyl-5-(p-tolyl)-1H-imidazol-3-ium-4-yl)pyrrol-1-ide (8c):

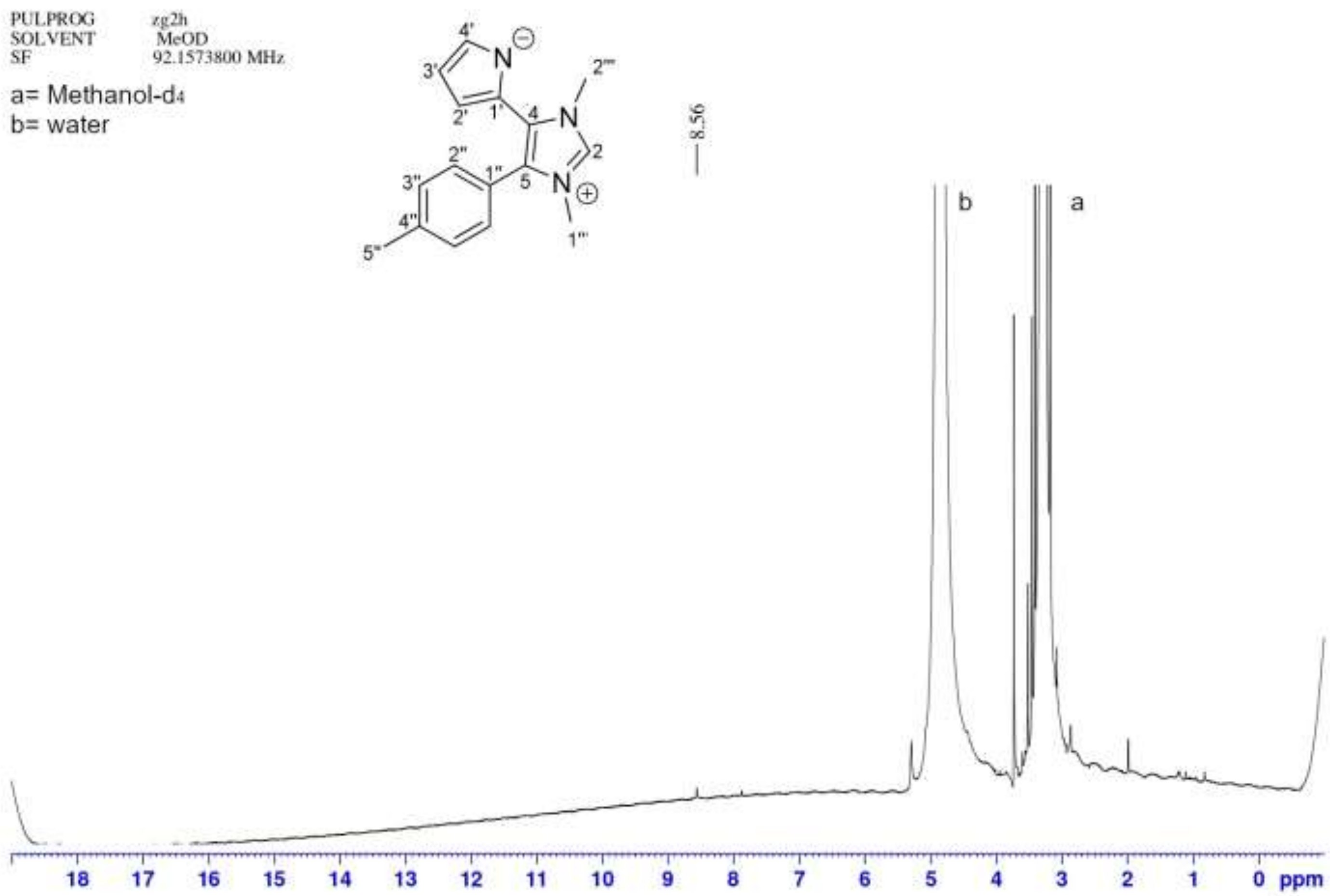

Figure S84. ²H-NMR of compound 8c

$^{13}C\{^1H\}$-NMR 2-(1,3-Dimethyl-5-(p-tolyl)-1H-imidazol-3-ium-4-yl)pyrrol-1-ide (8c):

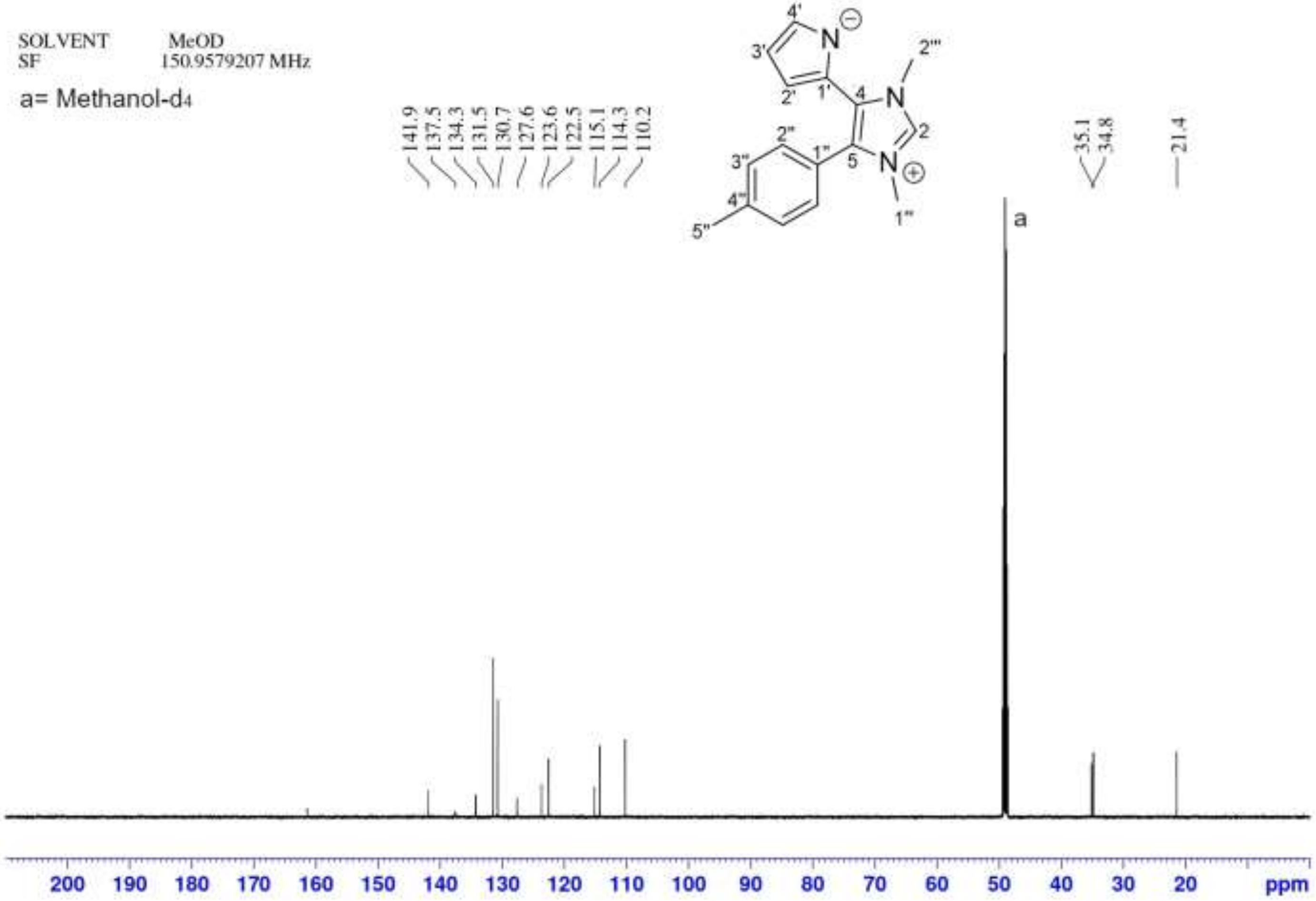

Figure S85. **$^{13}C\{^1H\}$-NMR of compound 8c**

$^{13}C\{^{1}H\}$-DEPT-NMR 2-(1,3-Dimethyl-5-(p-tolyl)-1H-imidazol-3-ium-4-yl)pyrrol-1-ide (8c):

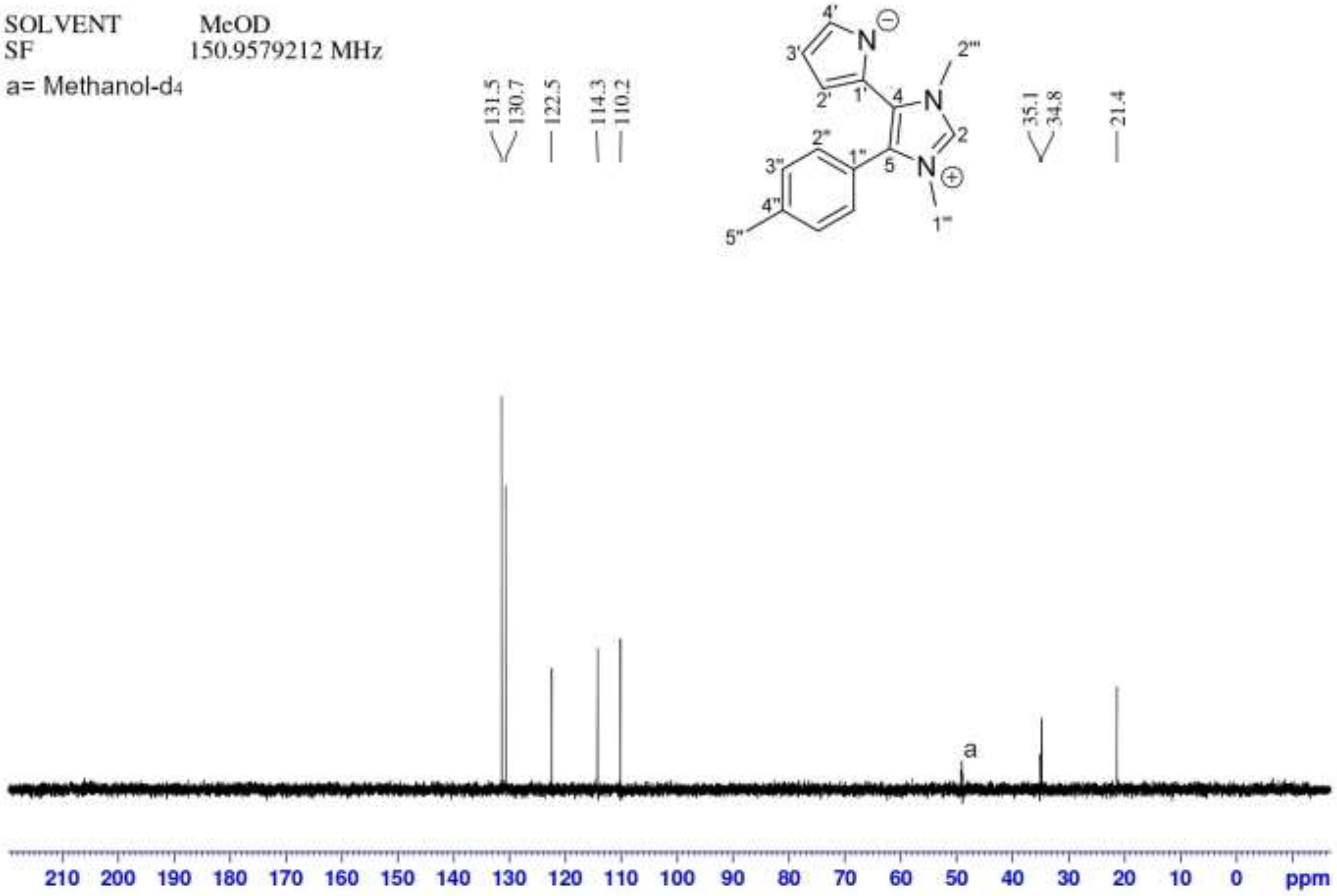

Figure S86. $^{13}C\{^{1}H\}$**-DEPT-NMR of compound 8c**

HSQC-NMR 2-(1,3-Dimethyl-5-(p-tolyl)-1H-imidazol-3-ium-4-yl)pyrrol-1-ide (8c):

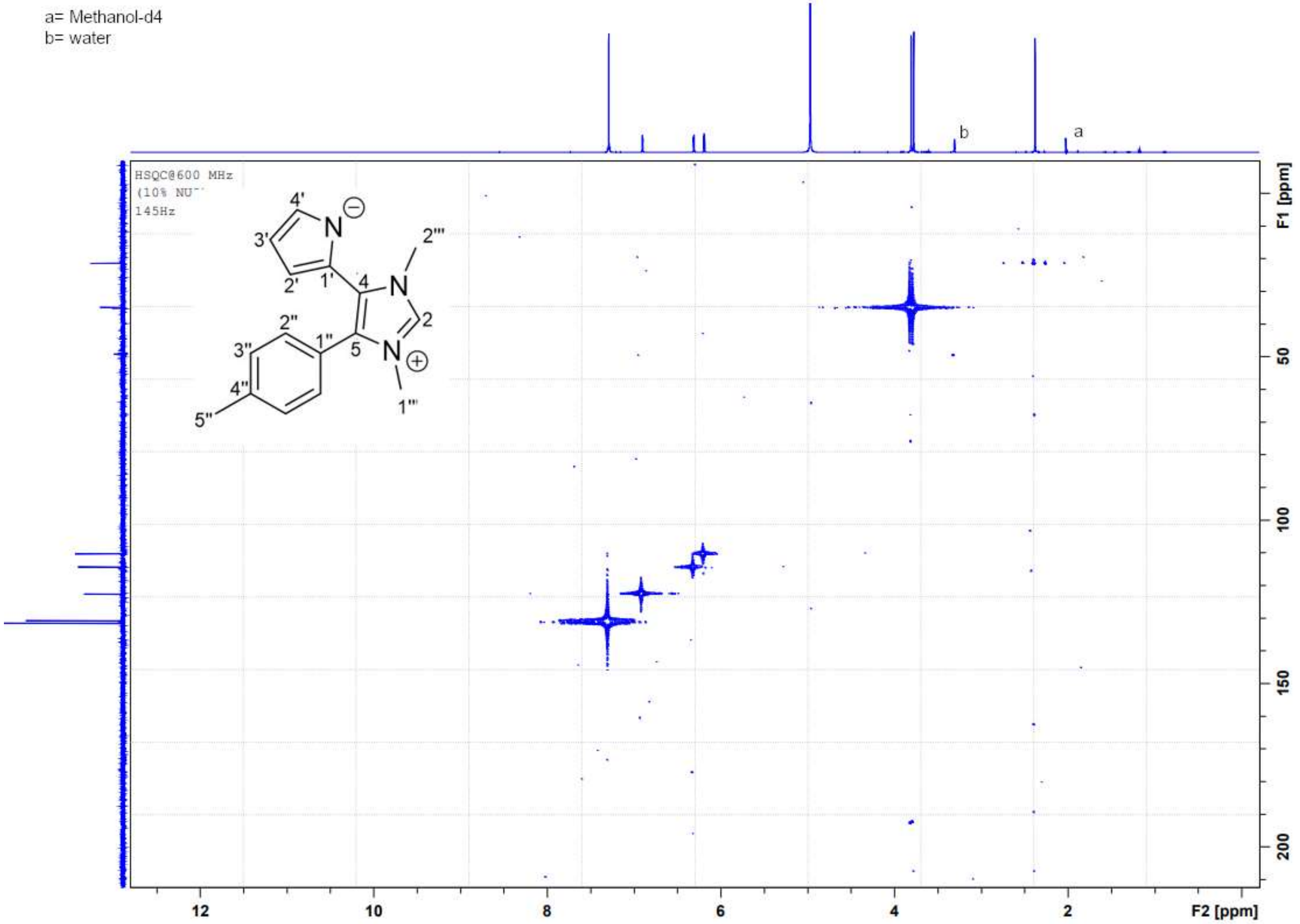

Figure S87. HSQC-NMR of compound 8c

HMBC-NMR 2-(1,3-Dimethyl-5-(p-tolyl)-1H-imidazol-3-ium-4-yl)pyrrol-1-ide (8c):

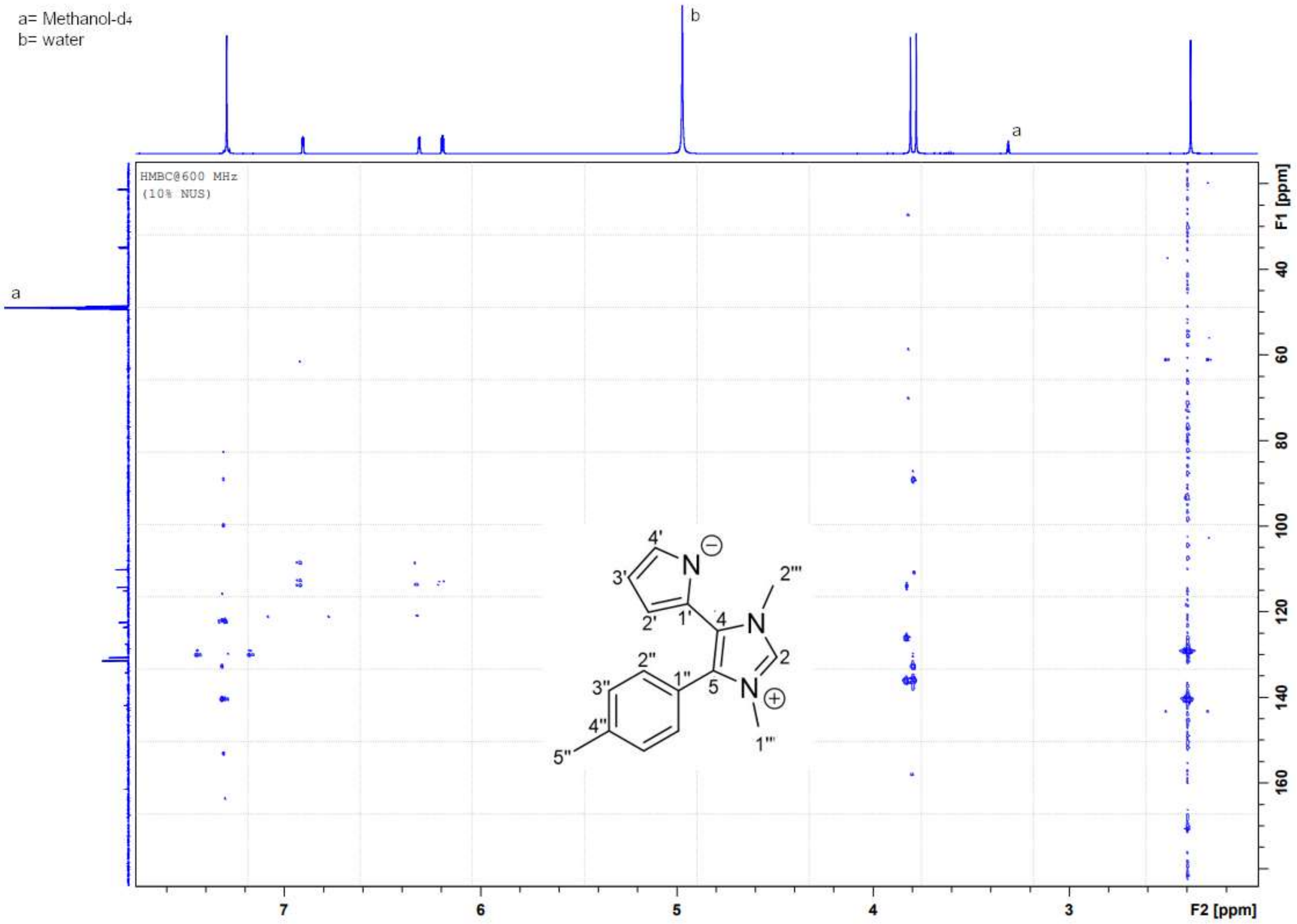

Figure S88. HMBC-NMR of compound 8c

^{1}H-NMR 2-(5-(4-Methoxyphenyl)-1,3-dimethyl-1H-imidazol-3-ium-4-yl)pyrrol-1-ide (8d):

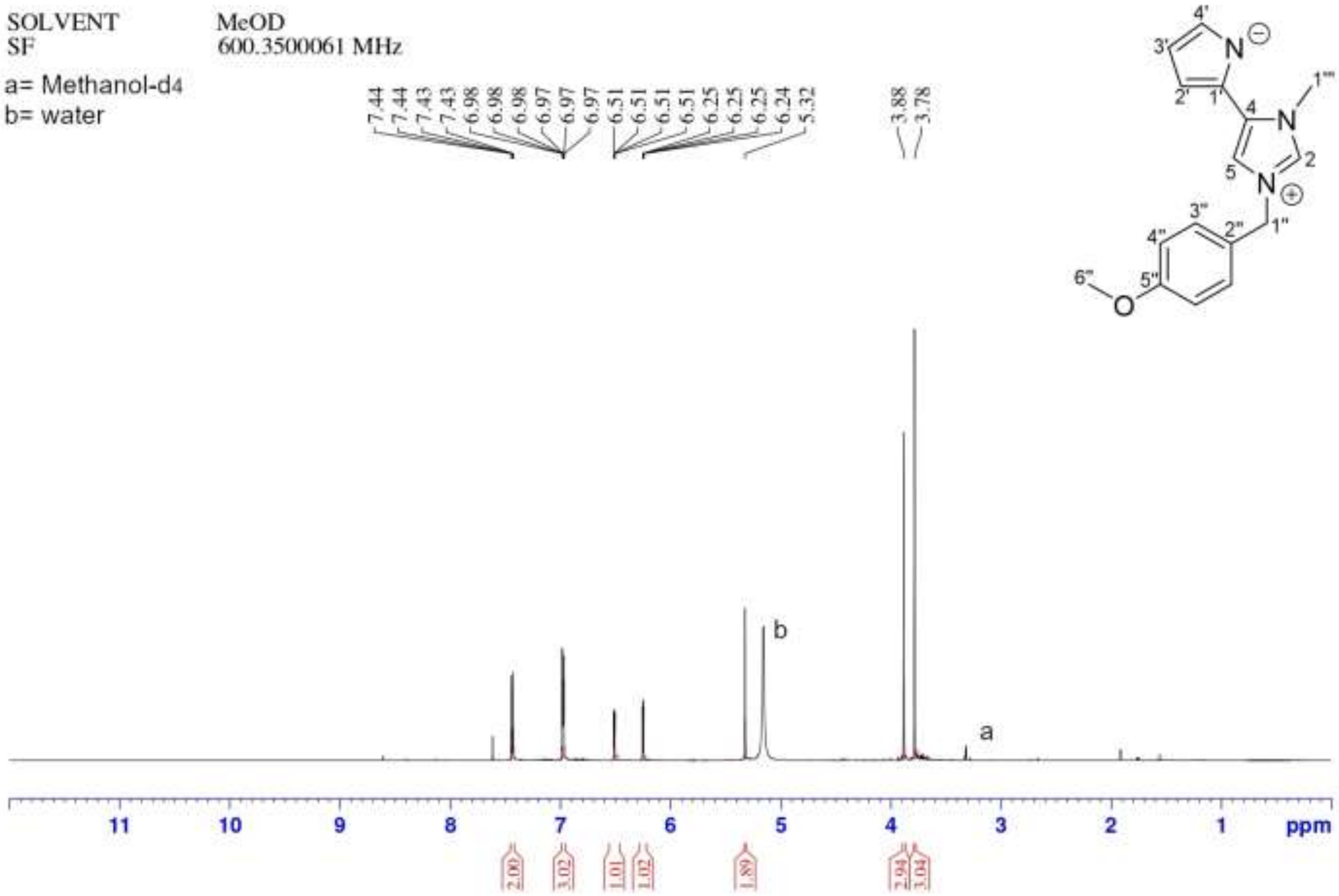

Figure S89. ^{1}H-NMR of compound 8d

^{2}H-NMR 2-(5-(4-Methoxyphenyl)-1,3-dimethyl-1H-imidazol-3-ium-4-yl)pyrrol-1-ide (8d):

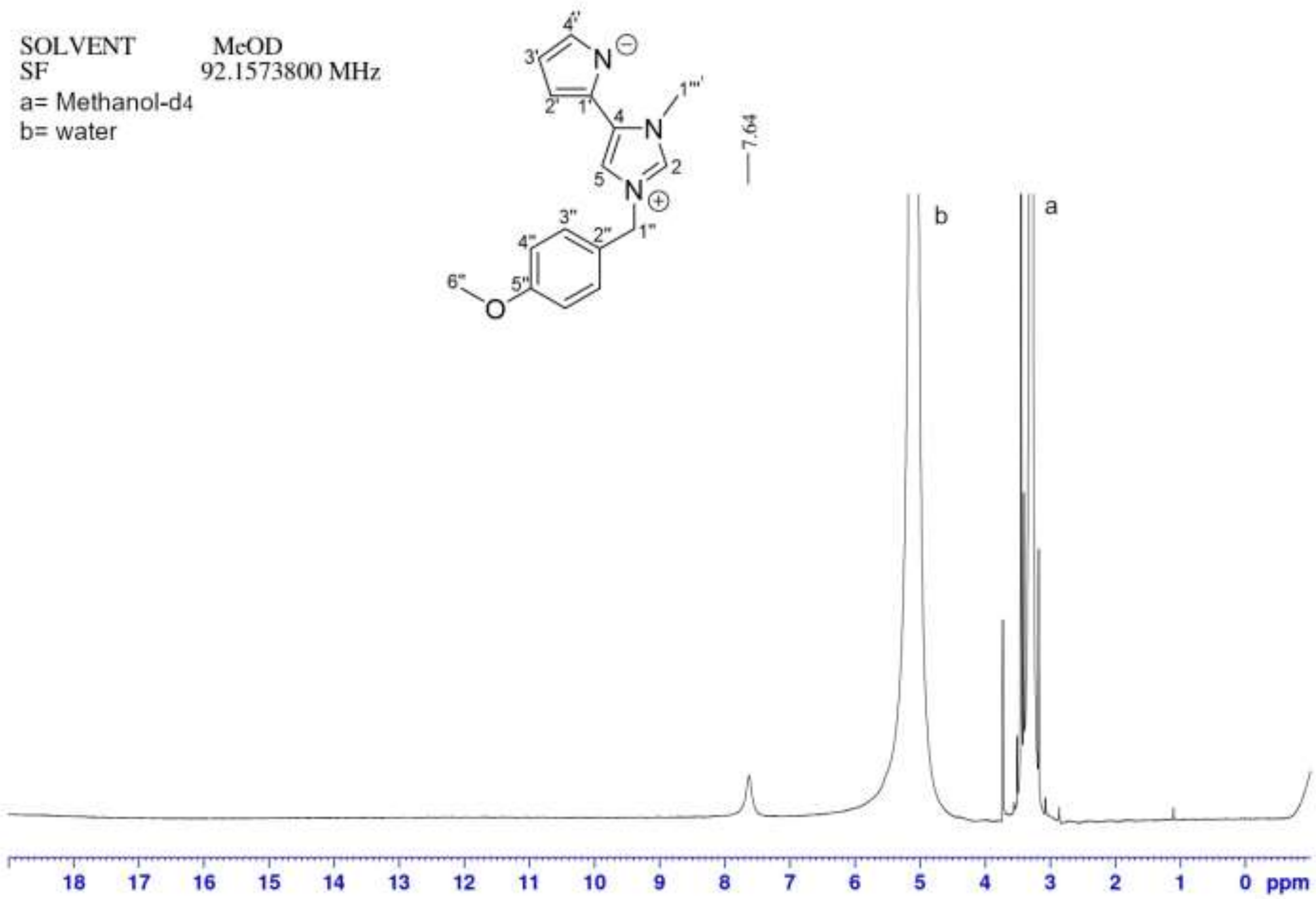

Figure S90. ^{2}**H-NMR of compound 8d**

$^{13}C\{^1H\}$-NMR 2-(5-(4-Methoxyphenyl)-1,3-dimethyl-1H-imidazol-3-ium-4-yl)pyrrol-1-ide (8d):

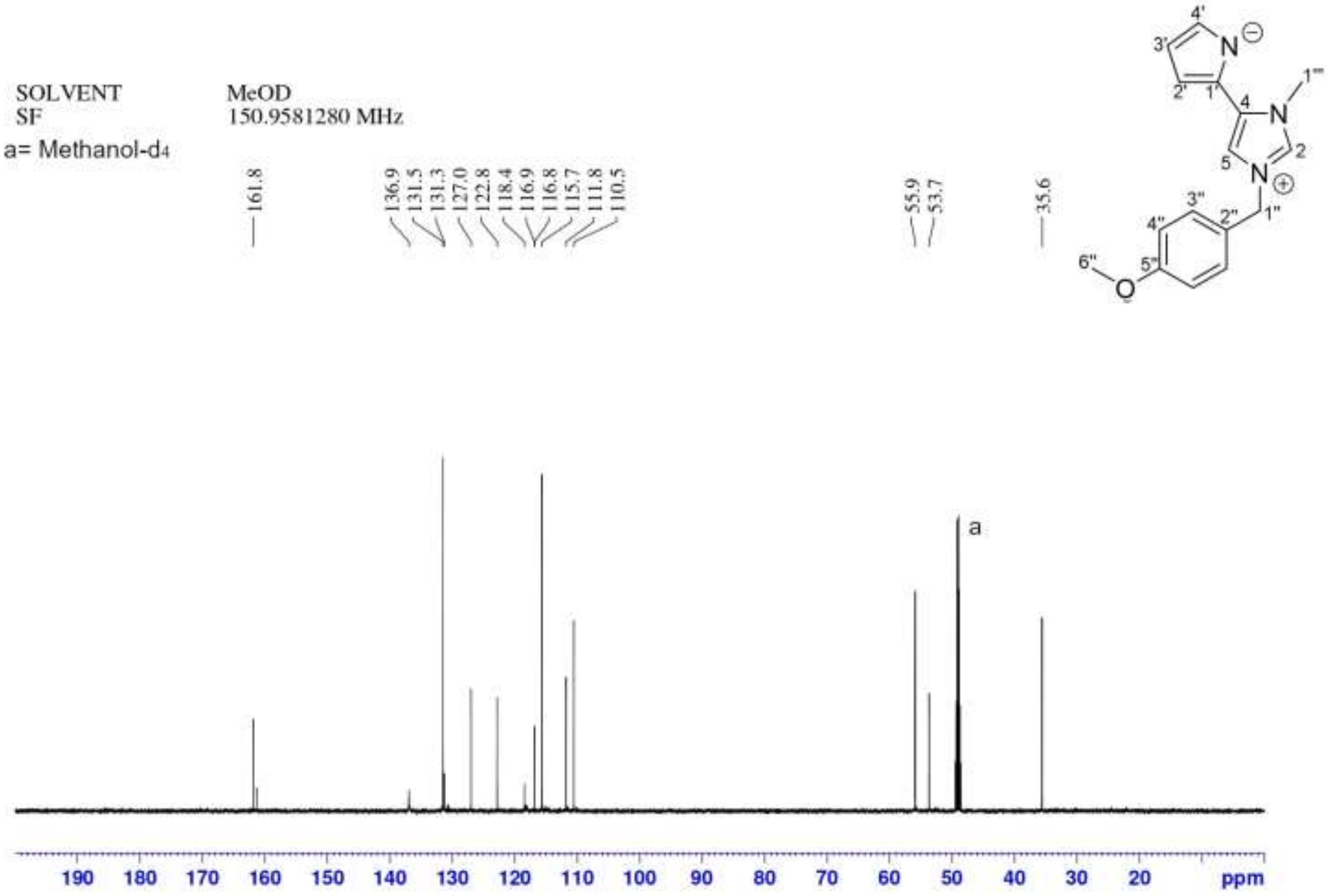

Figure S91. **$^{13}C\{^1H\}$-NMR of compound 8d**

$^{13}C\{^1H\}$-DEPT-NMR 2-(5-(4-Methoxyphenyl)-1,3-dimethyl-1H-imidazol-3-ium-4-yl)pyrrol-1-ide (8d):

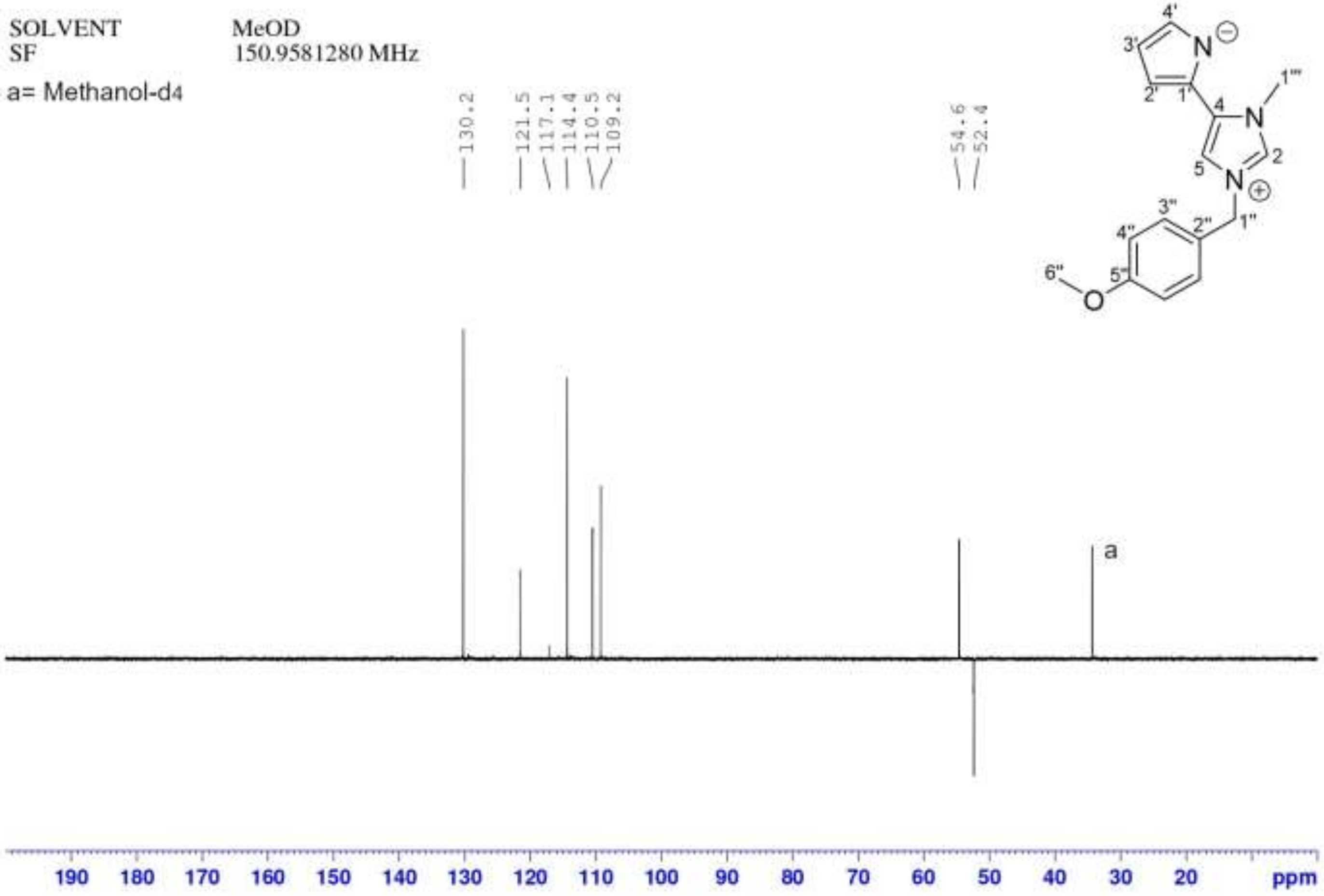

Figure S92. $^{13}C\{^1H\}$-DEPT-NMR of compound 8d

HSQC-NMR 2-(5-(4-Methoxyphenyl)-1,3-dimethyl-1H-imidazol-3-ium-4-yl)pyrrol-1-ide (8d):

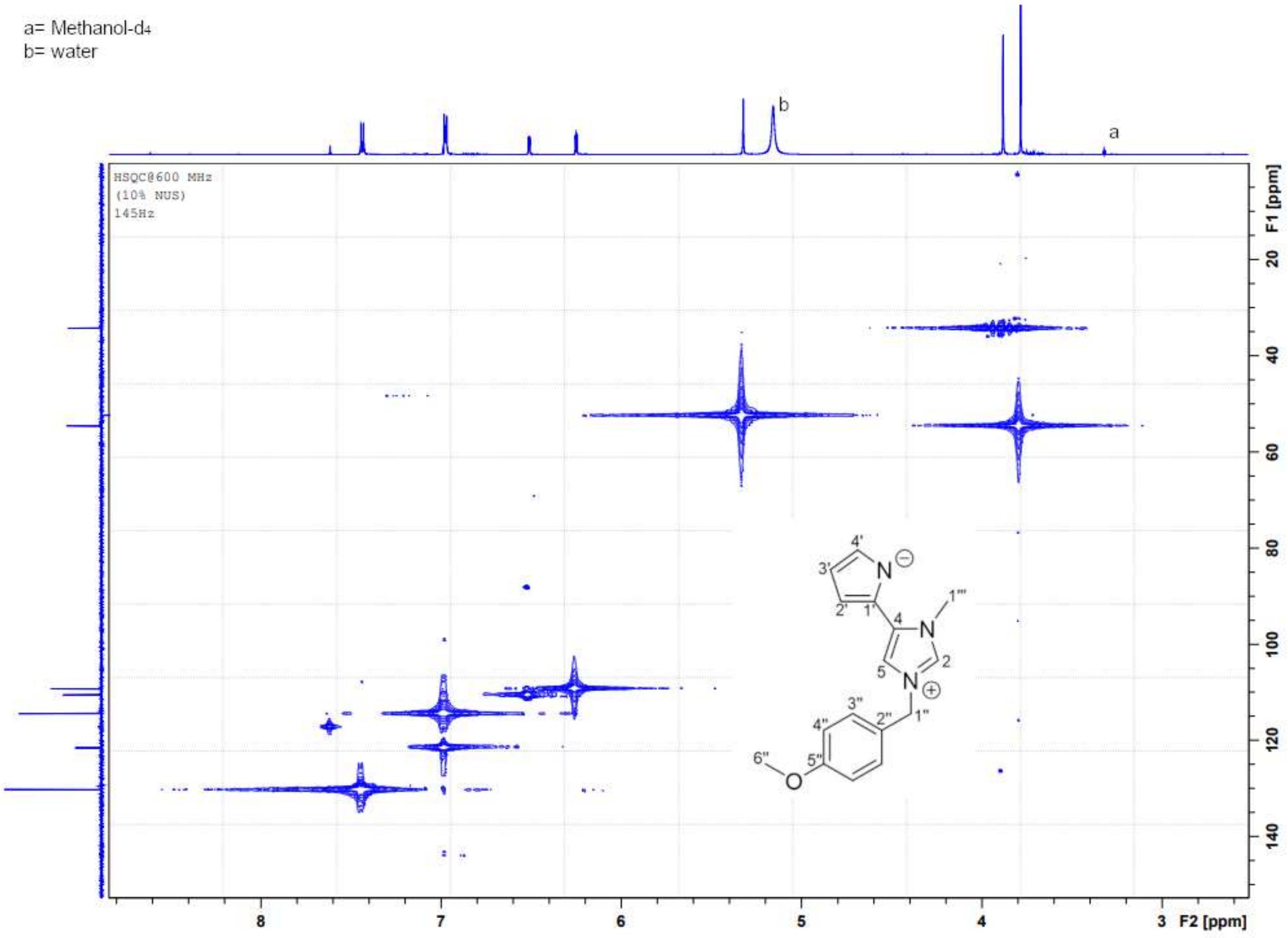

Figure S93. HSQC-NMR of compound 8d

HMBC-NMR 2-(5-(4-Methoxyphenyl)-1,3-dimethyl-1H-imidazol-3-ium-4-yl)pyrrol-1-ide (8d):

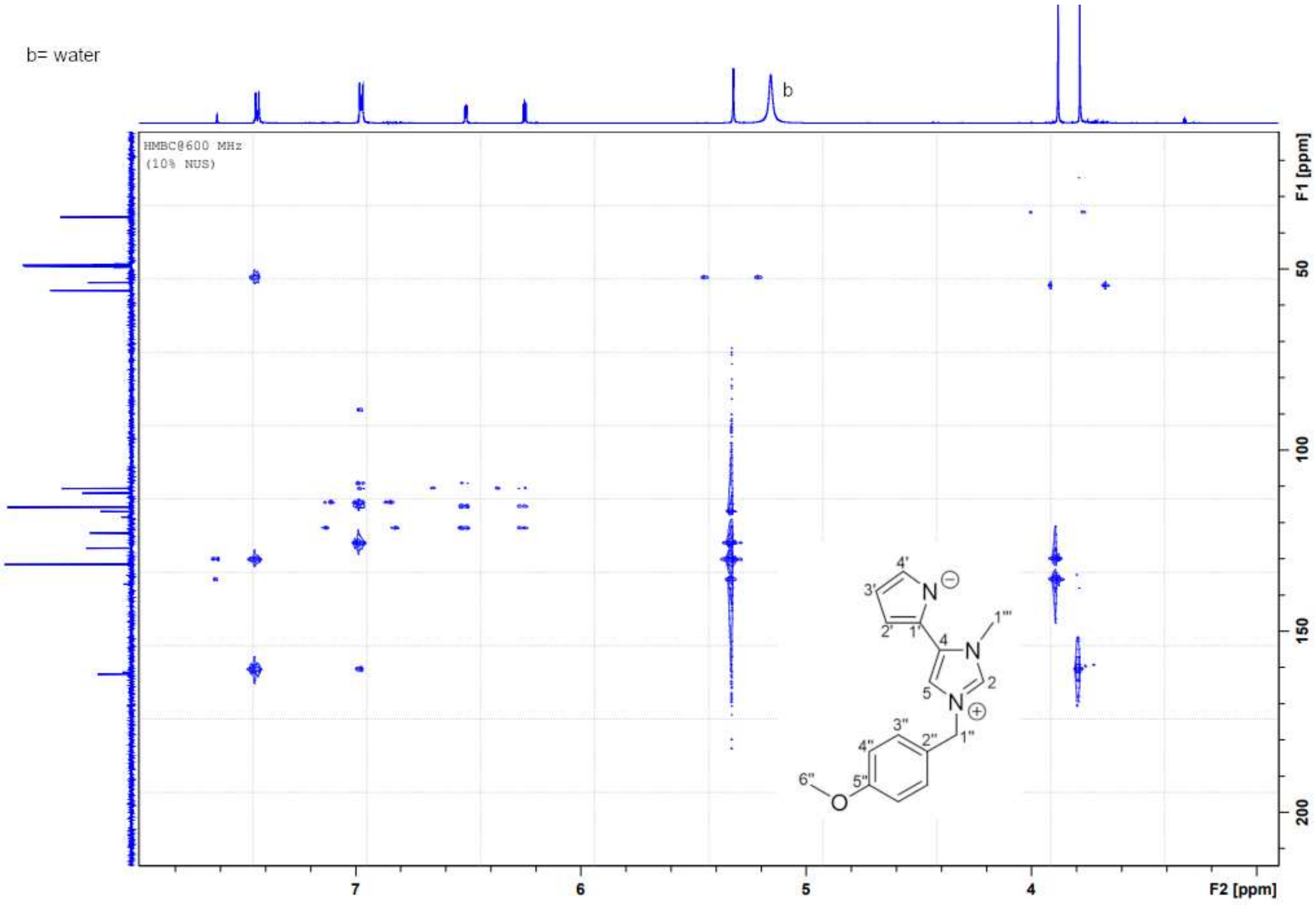

Figure S94. HMBC-NMR of compound 8d

^{1}H-NMR 2-(1-Benzyl-3-methyl-1H-imidazol-3-ium-4-yl)pyrrol-1-ide (8e):

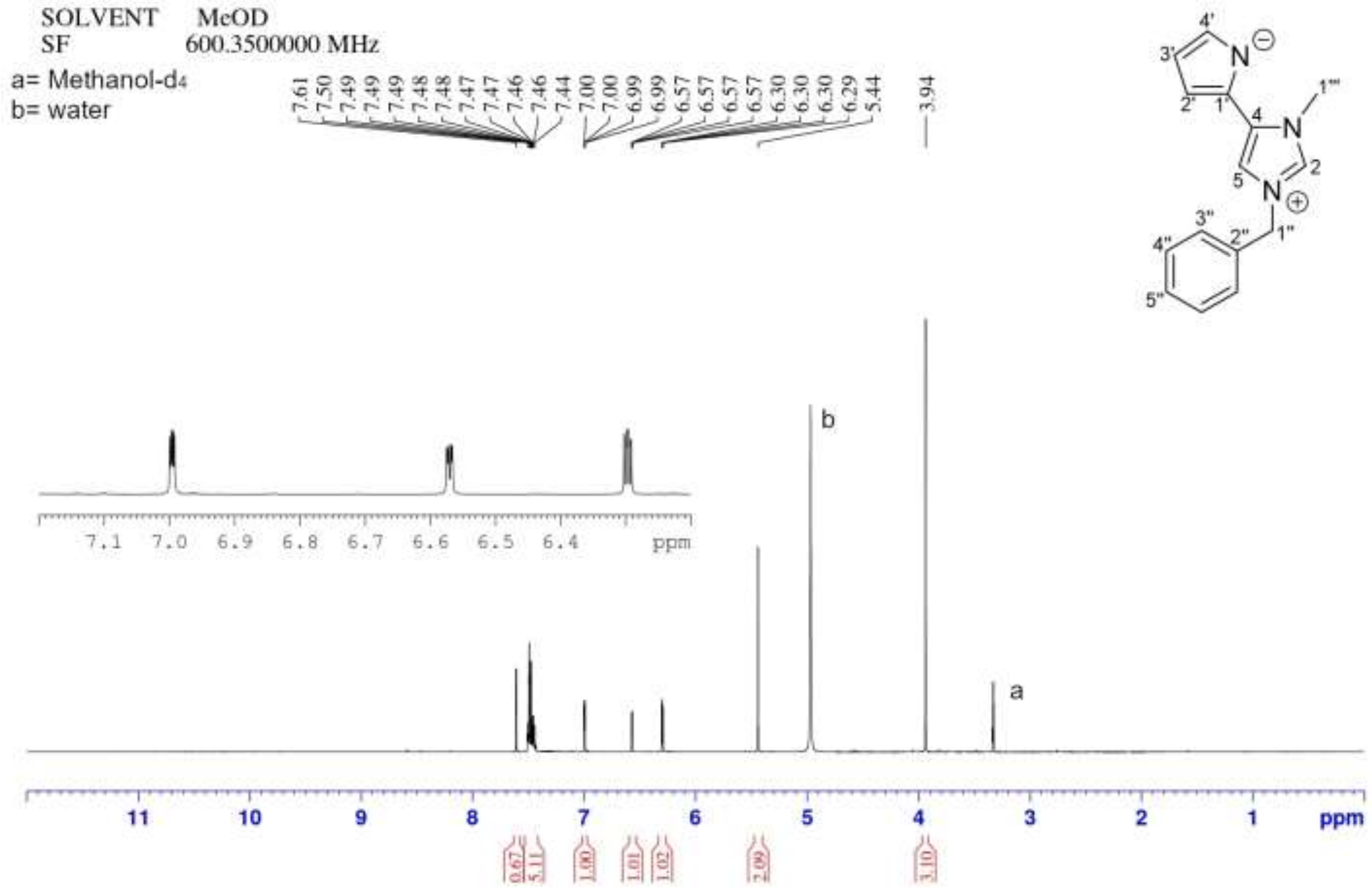

Figure S95. ^{1}H-NMR of compound 8e

$^{13}C\{^1H\}$-NMR 2-(1-Benzyl-3-methyl-1H-imidazol-3-ium-4-yl)pyrrol-1-ide (8e):

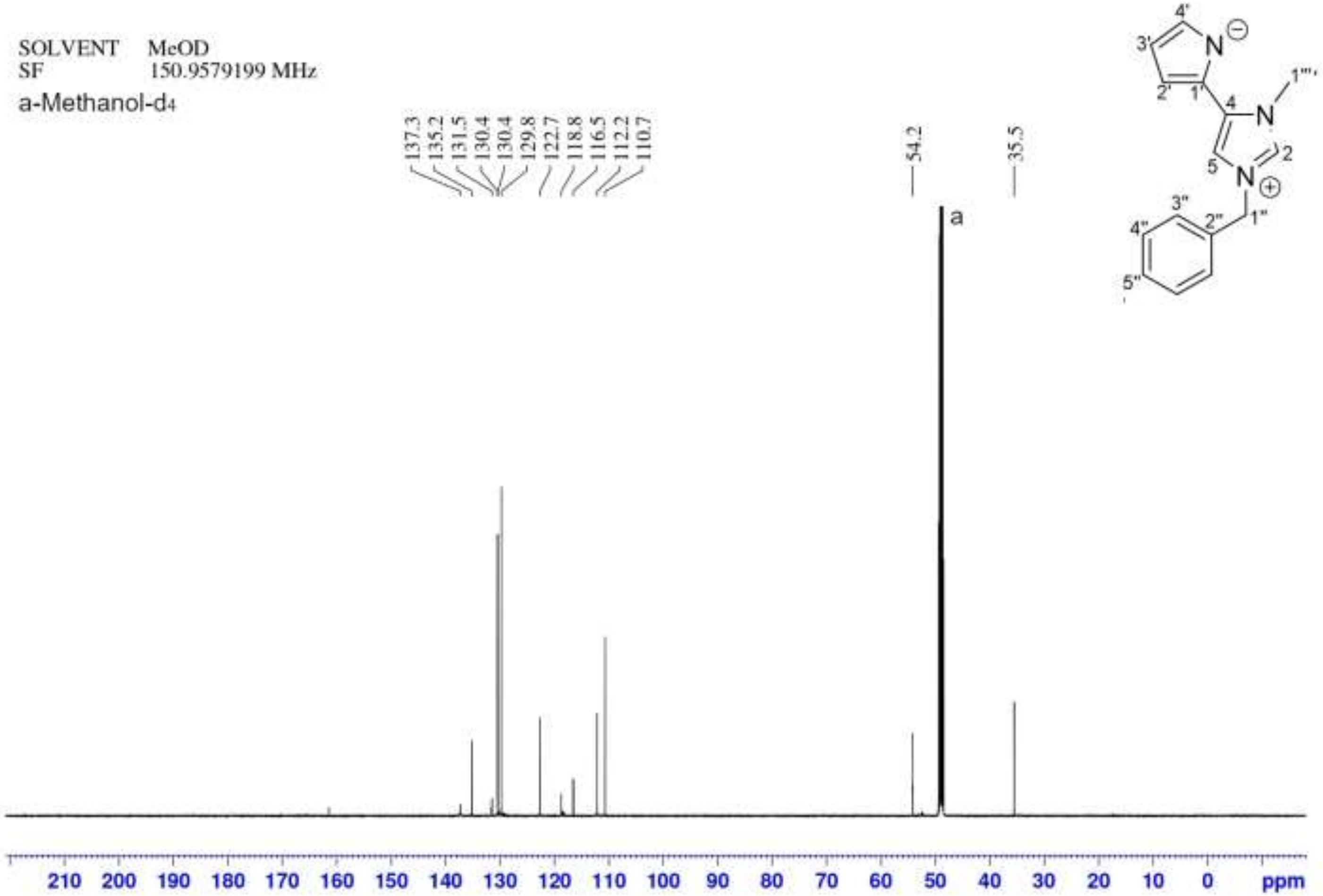

Figure S96. $^{13}C\{^1H\}$**-NMR of compound 8e**

$^{13}C\{^{1}H\}$-DEPT-NMR 2-(1-Benzyl-3-methyl-1H-imidazol-3-ium-4-yl)pyrrol-1-ide (8e):

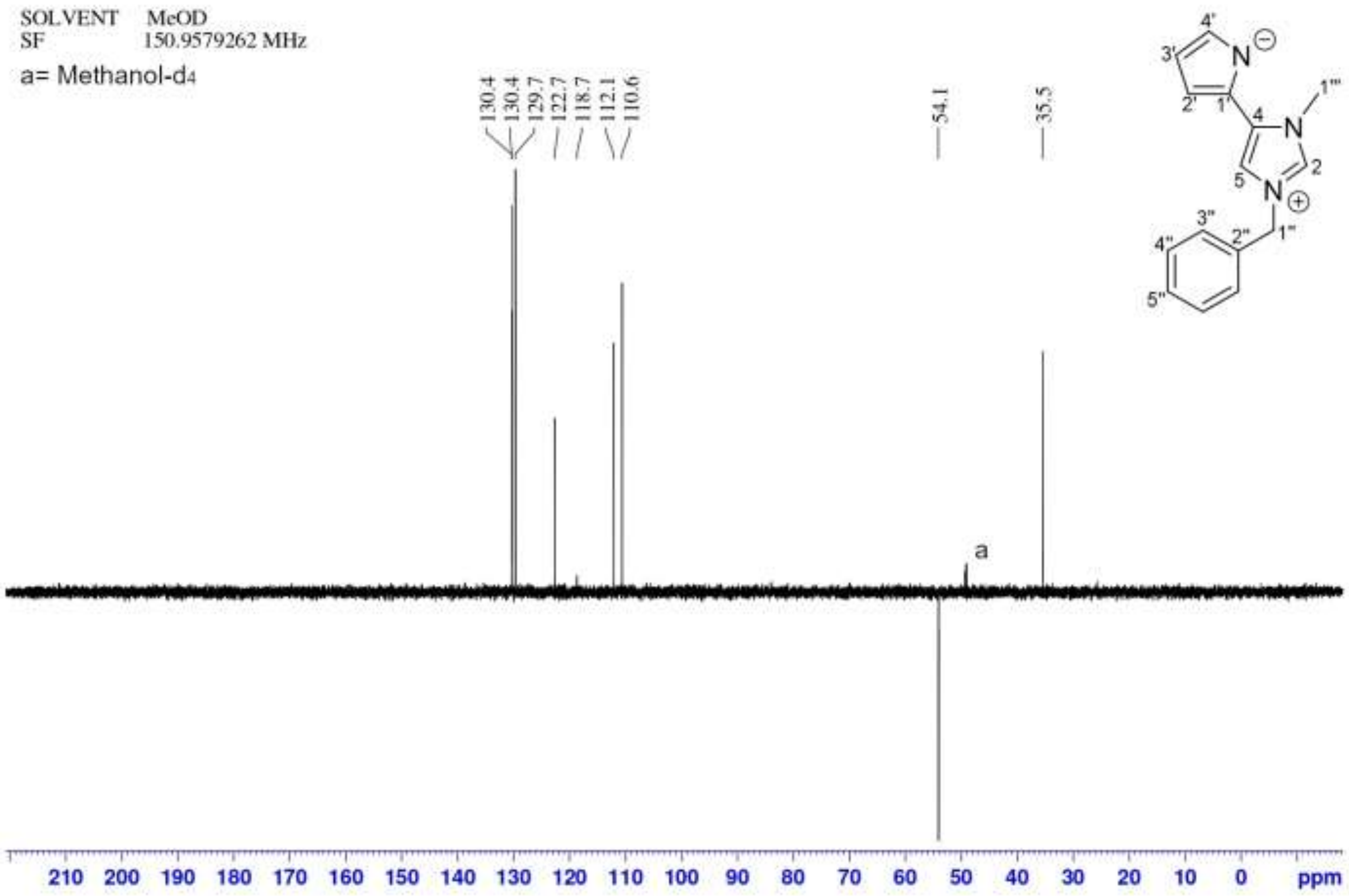

Figure S97. **$^{13}C\{^{1}H\}$-DEPT-NMR of compound 8e**

HSQC-NMR 2-(1-Benzyl-3-methyl-1H-imidazol-3-ium-4-yl)pyrrol-1-ide (8e):

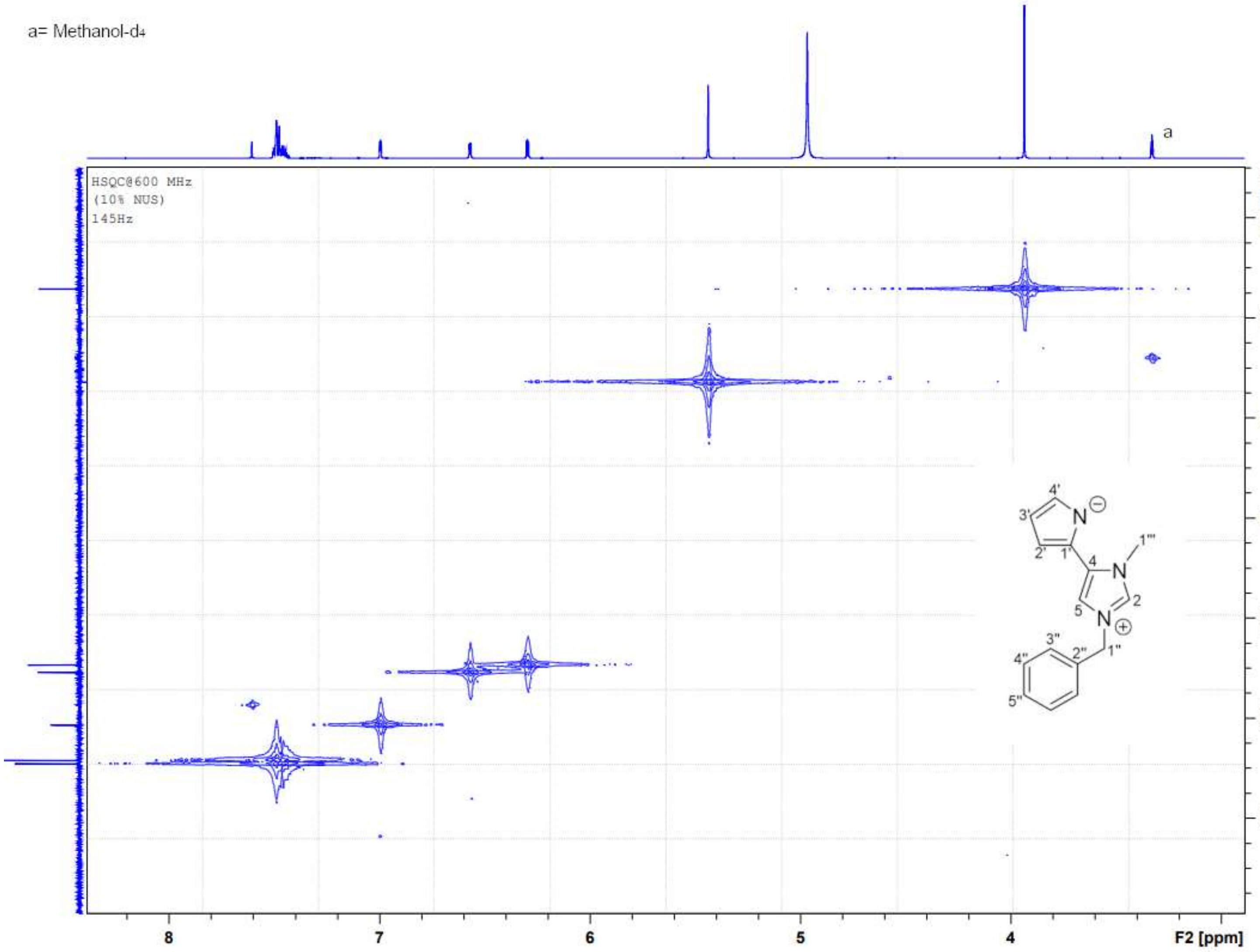

Figure S98. HSQC-NMR of compound 8e

HMBC-NMR 2-(1-Benzyl-3-methyl-1H-imidazol-3-ium-4-yl)pyrrol-1-ide (8e):

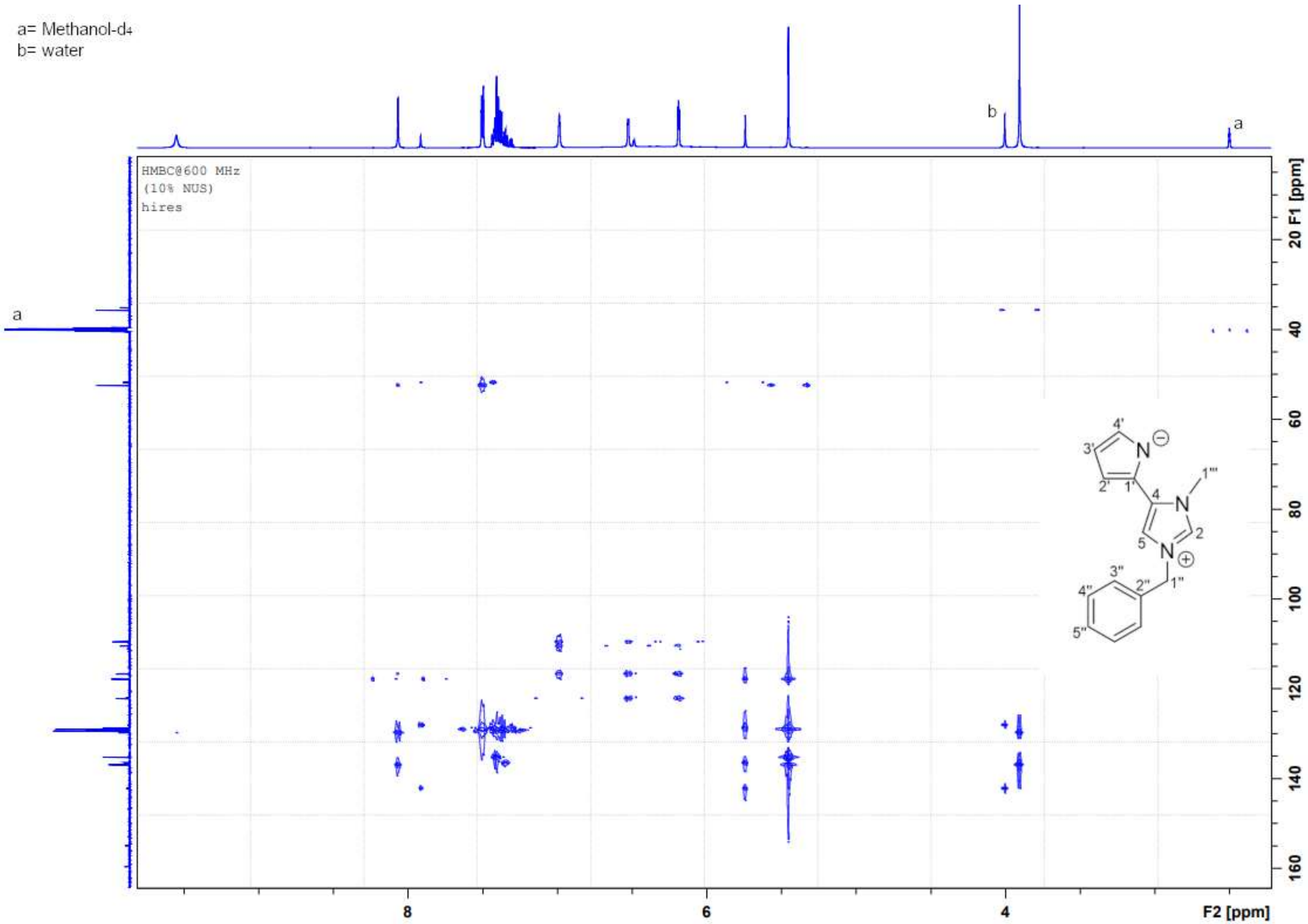

Figure S99. HMBC-NMR of compound 8e

^{1}H-NMR 2-(3-Methyl-1-(4-methylbenzyl)-1H-imidazol-3-ium-4-yl)pyrrol-1-ide (8f):

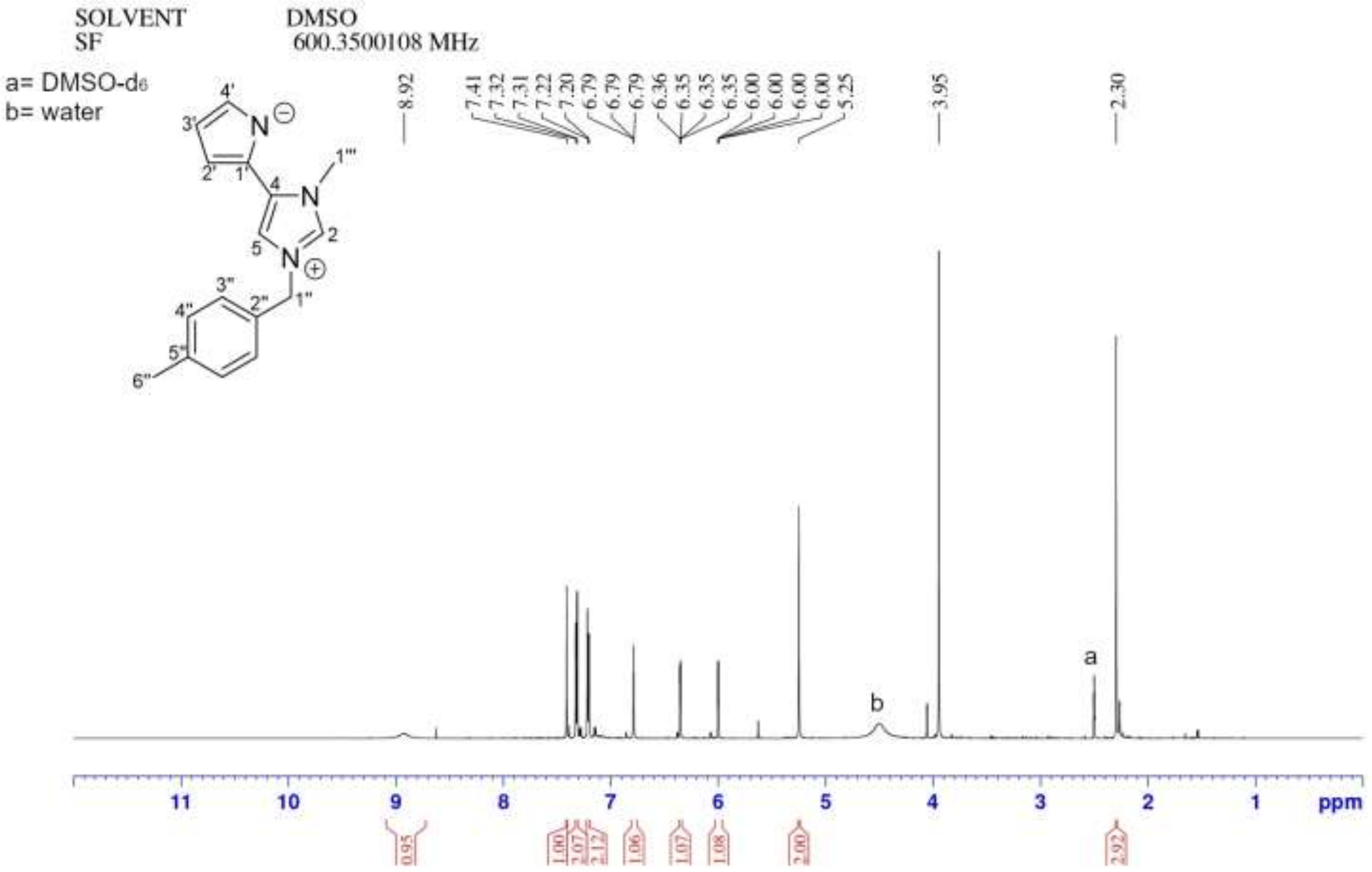

Figure S100. [1]**H-NMR of compound 8f**

$^{13}C\{^1H\}$-NMR 2-(3-Methyl-1-4-methylbenzyl)-1H-imidazol-3-ium-4-yl)pyrrol-1-ide (8f):

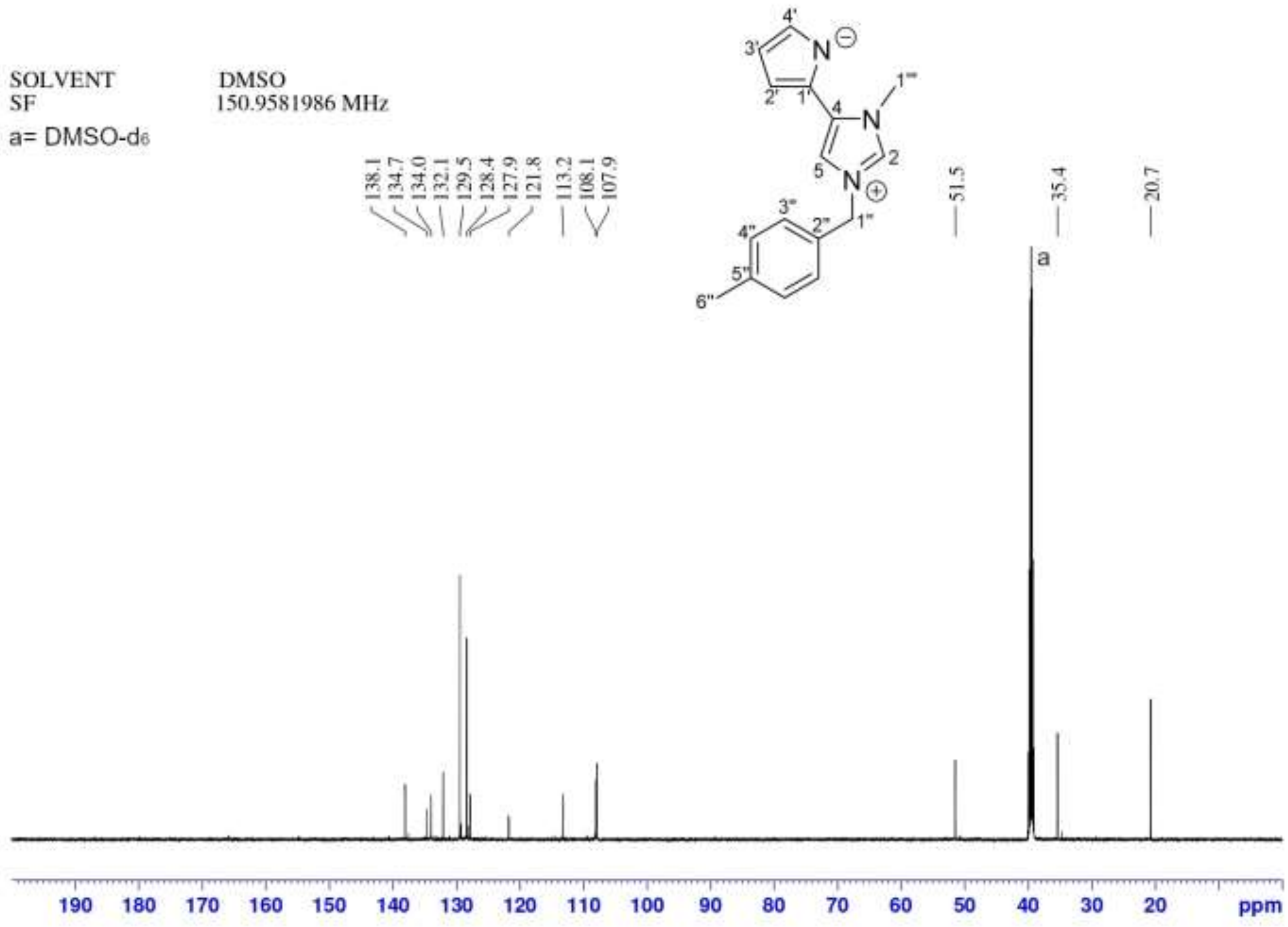

Figure S101. $^{13}C\{^1H\}$**-NMR of compound 8f**

$^{13}C\{^1H\}$-DEPT-NMR 2-(3-Methyl-1-(4-methylbenzyl)-1H-imidazol-3-ium-4-yl)pyrrol-1-ide (8f):

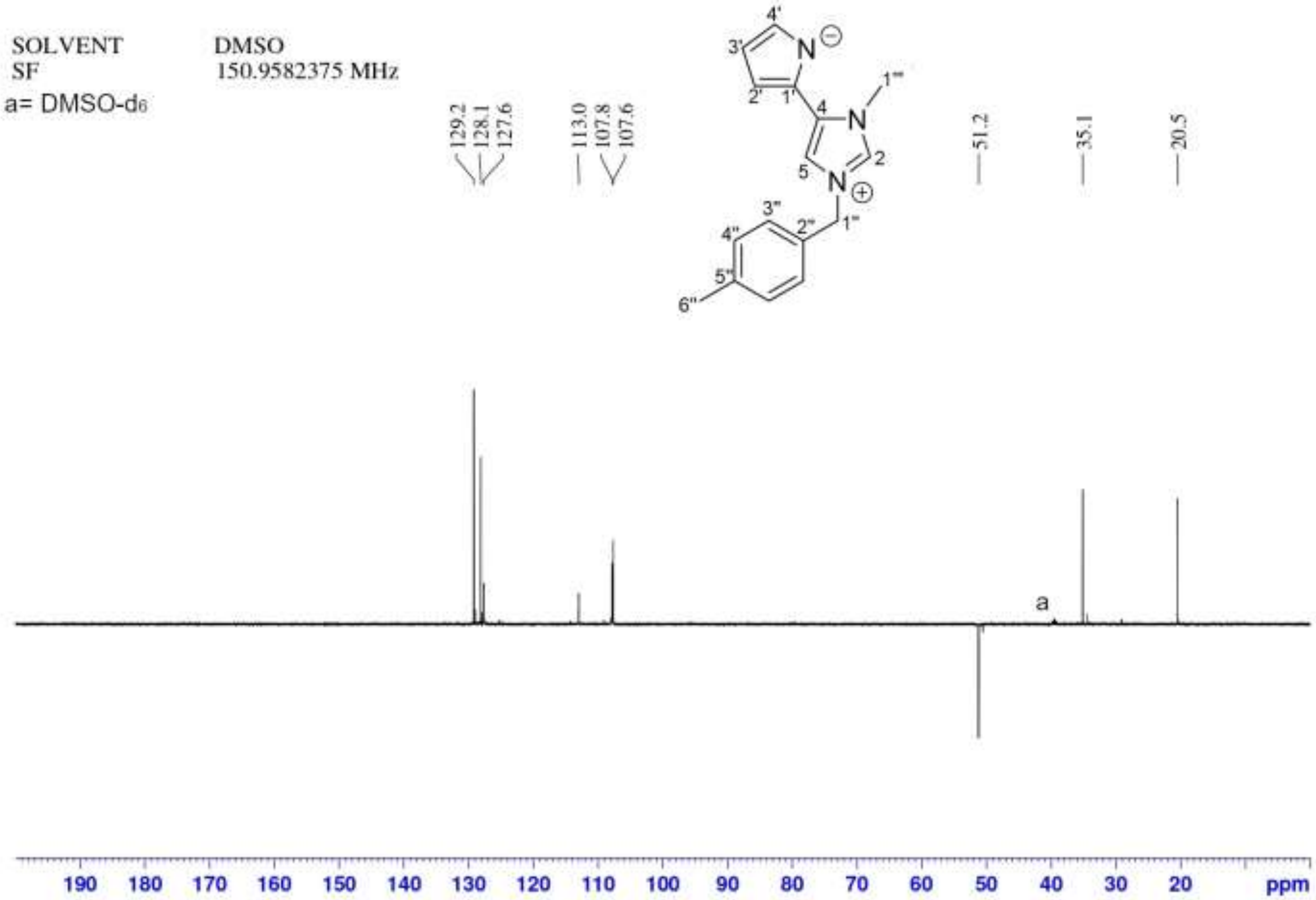

Figure S102. **$^{13}C\{^1H\}$-DEPT-NMR of compound 8f**

HSQC-NMR 2-(3-Methyl-1-(4-methylbenzyl)-1H-imidazol-3-ium-4-yl)pyrrol-1-ide (8f):

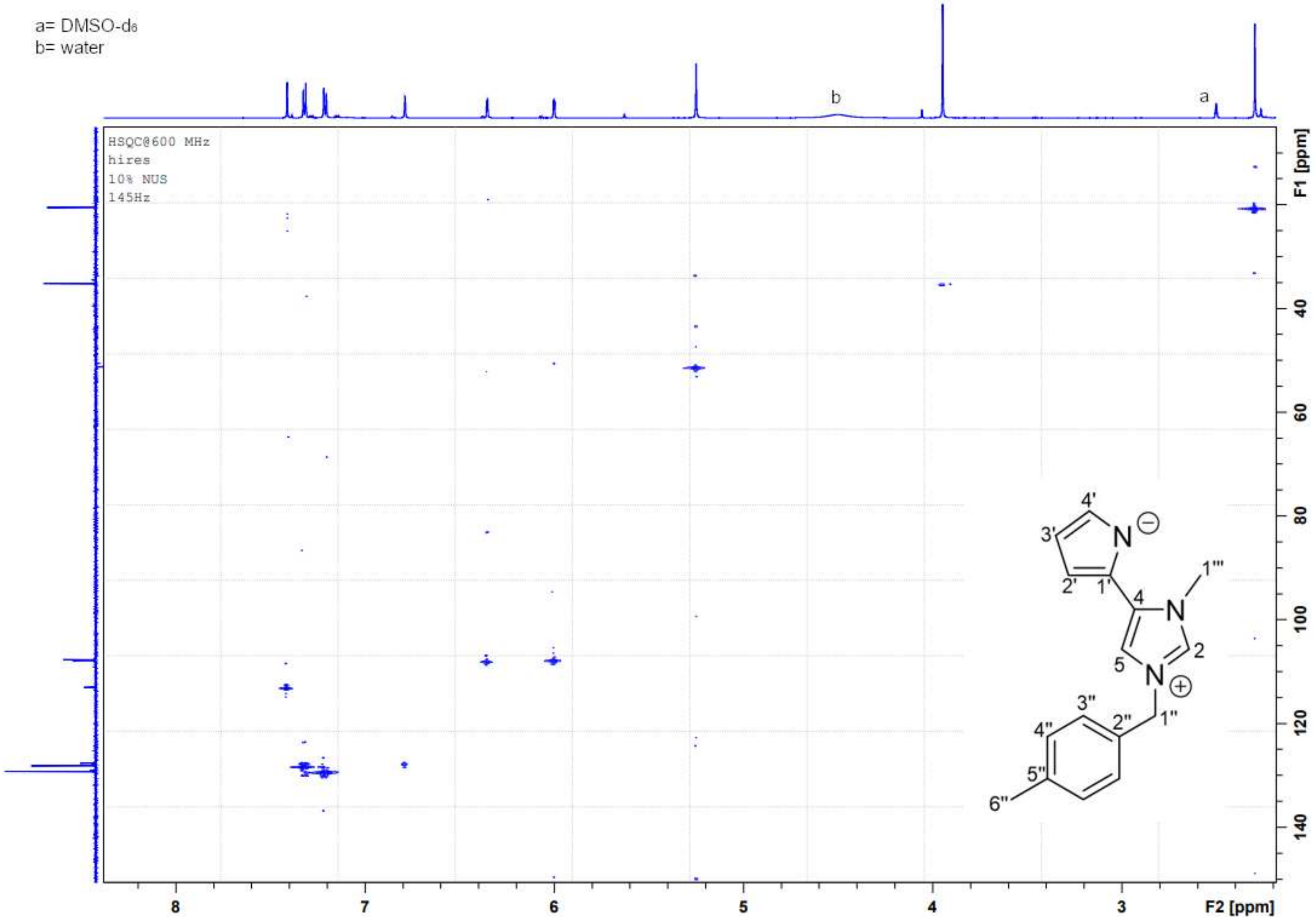

Figure S103. HSQC-NMR of compound 8f

HMBC-NMR 2-(3-Methyl-1-(4-methylbenzyl)-1H-imidazol-3-ium-4-yl)pyrrol-1-ide (8f):

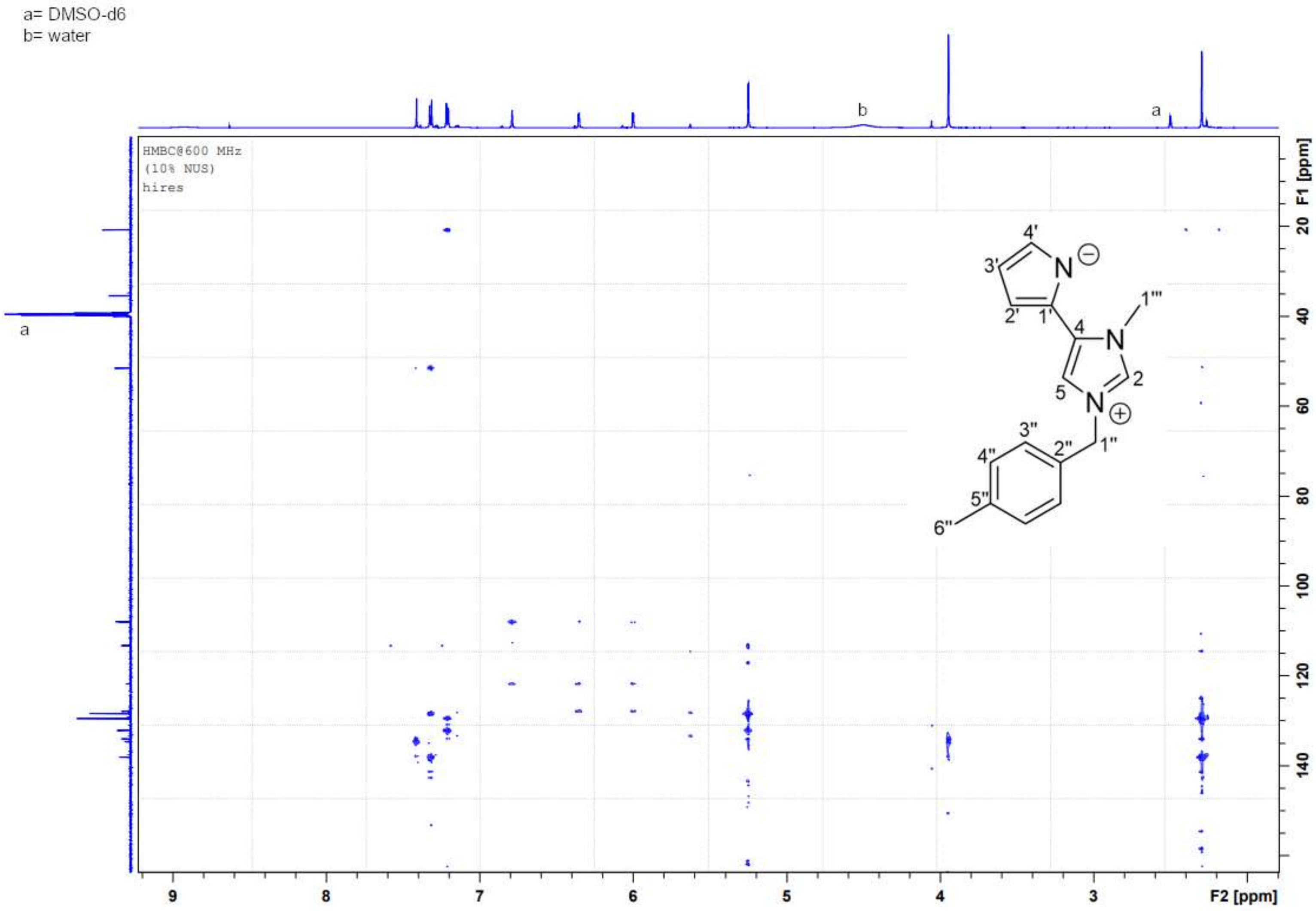

Figure S104. HMBC-NMR of compound 8f

^{1}H-NMR 2-(1-(4-Methoxybenzyl)-3-methyl-1H-imidazol-3-ium-4-yl)pyrrol-1-ide (8g):

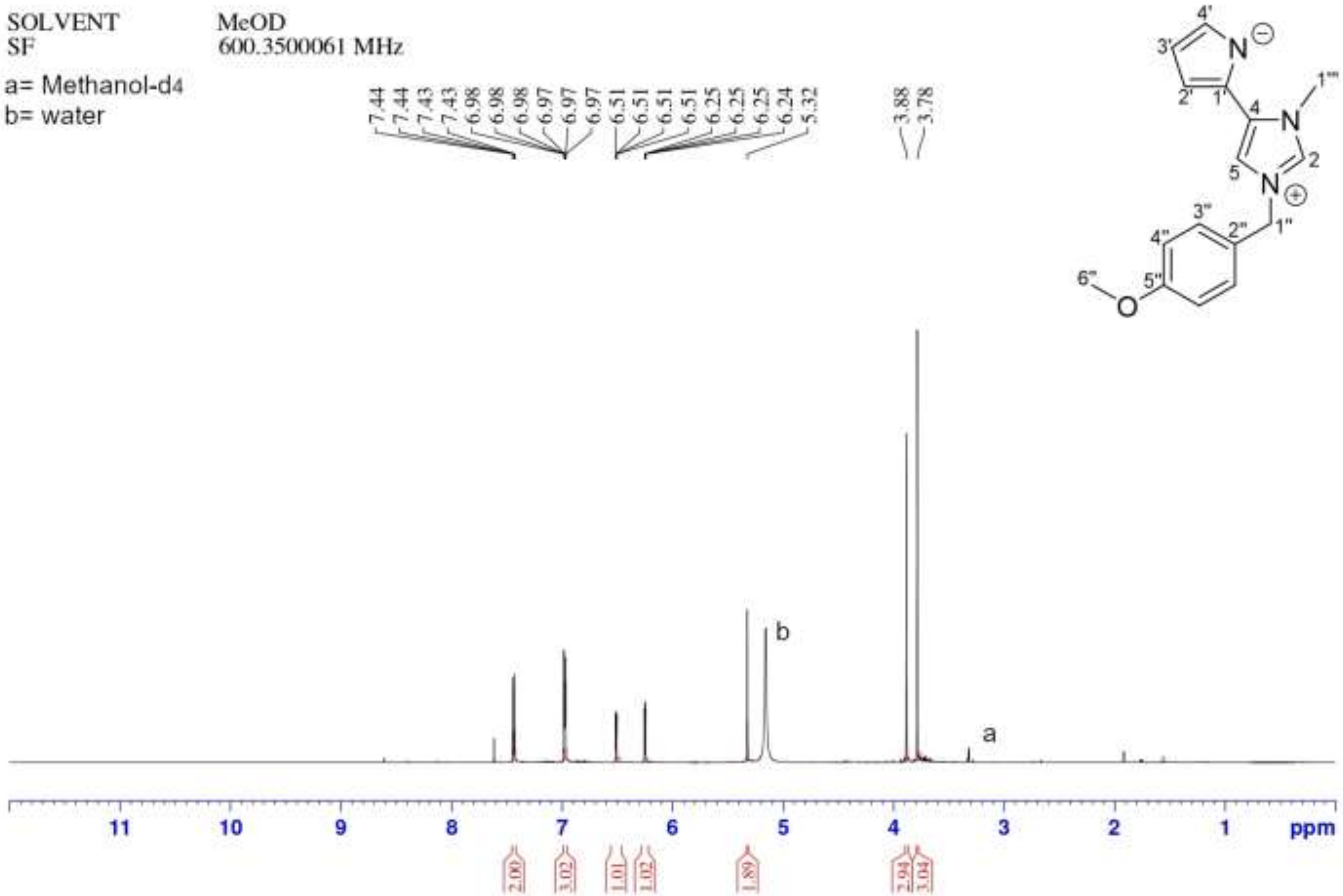

Figure S105. ^{1}H-NMR of compound 8g

^{2}**H-NMR 2-(1-(4-Methoxybenzyl)-3-methyl-1H-imidazol-3-ium-4-yl)pyrrol-1-ide (8g):**

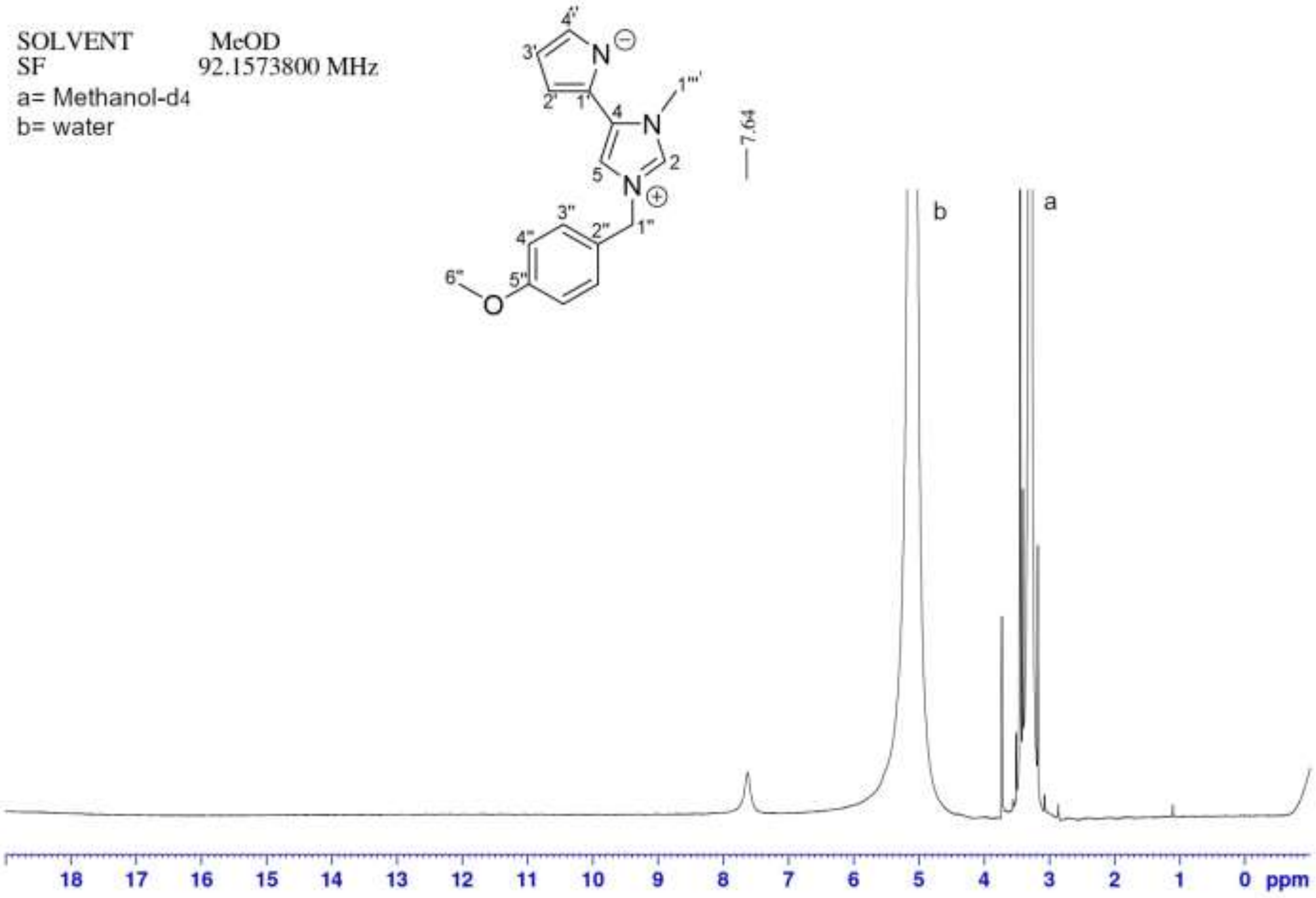

Figure S106. ^{2}**H-NMR of compound 8g**

$^{13}C\{^1H\}$-NMR 2-(1-(4-Methoxybenzyl)-3-methyl-1H-imidazol-3-ium-4-yl)pyrrol-1-ide (8g):

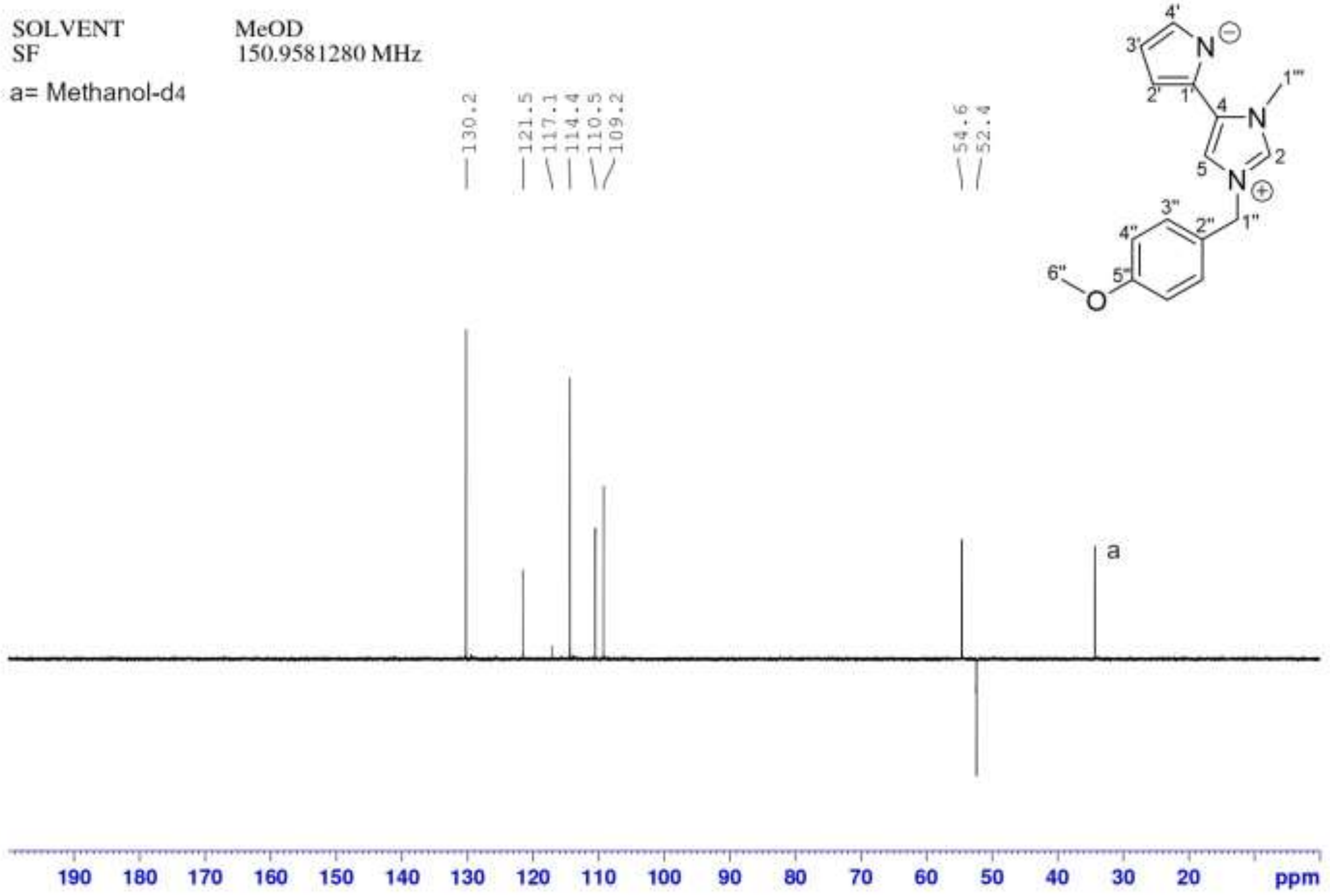

Figure S107. $^{13}C\{^1H\}$**-NMR of compound 8g**

$^{13}C\{^{1}H\}$-DEPT-NMR 2-(1-(4-Methoxybenzyl)-3-methyl-1H-imidazol-3-ium-4-yl)pyrrol-1-ide (8g):

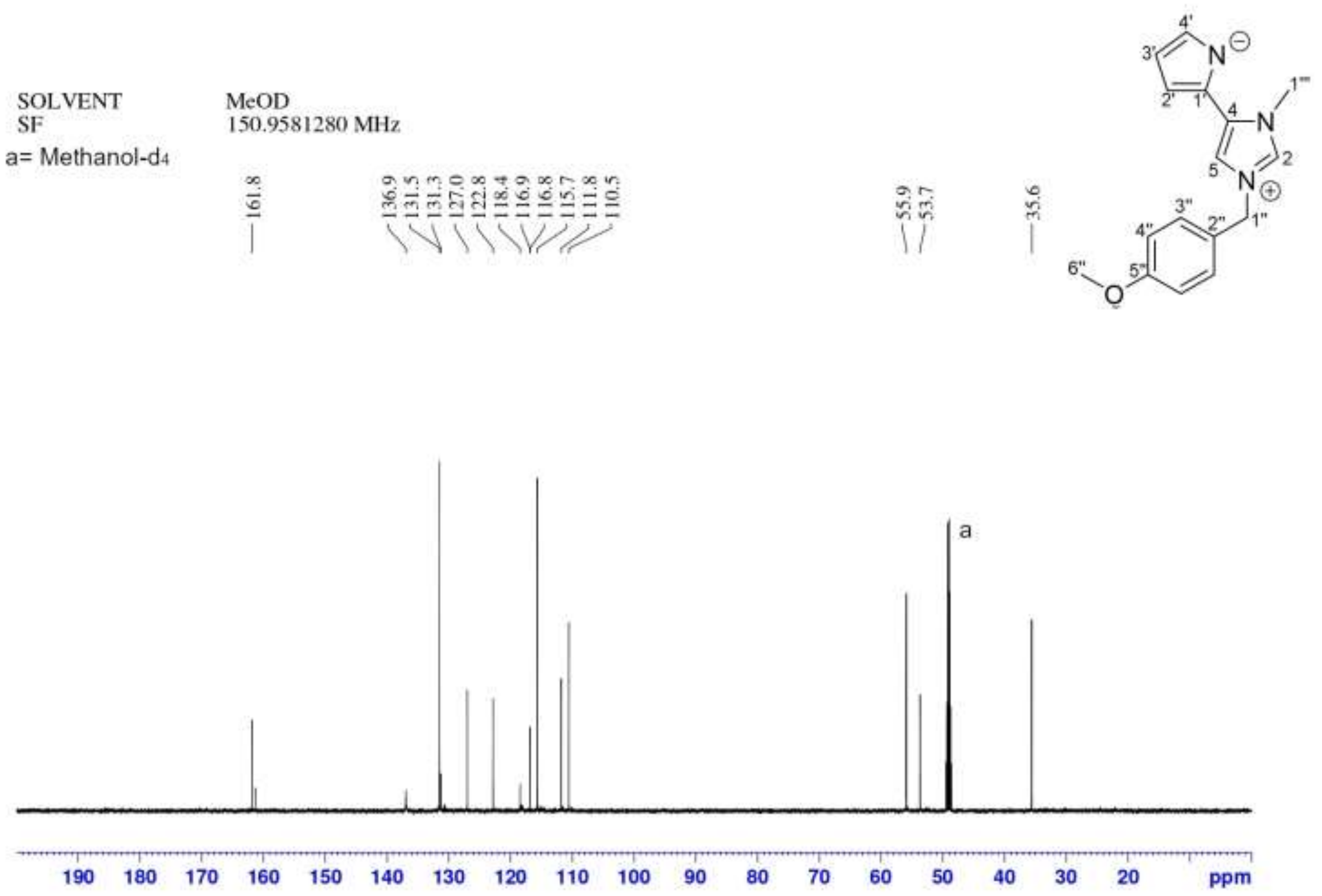

Figure S108. **$^{13}C\{^{1}H\}$-DEPT-NMR of compound 8g**

HSQC-NMR 2-(1-(4-Methoxybenzyl)-3-methyl-1H-imidazol-3-ium-4-yl)pyrrol-1-ide (8g):

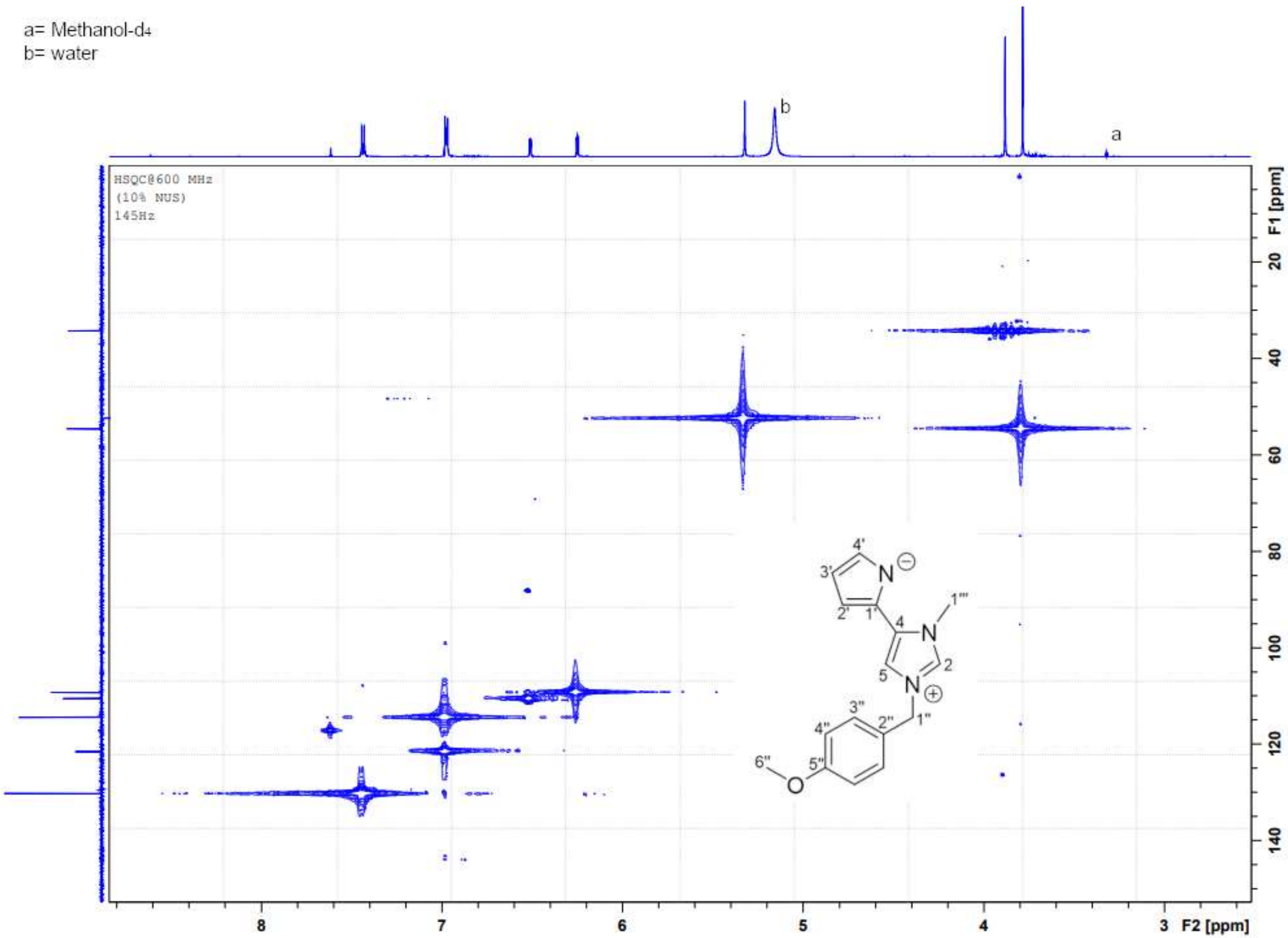

Figure S109. HSQC-NMR of compound 8g

HMBC-NMR 2-(1-(4-Methoxybenzyl)-3-methyl-1H-imidazol-3-ium-4-yl)pyrrol-1-ide (8g):

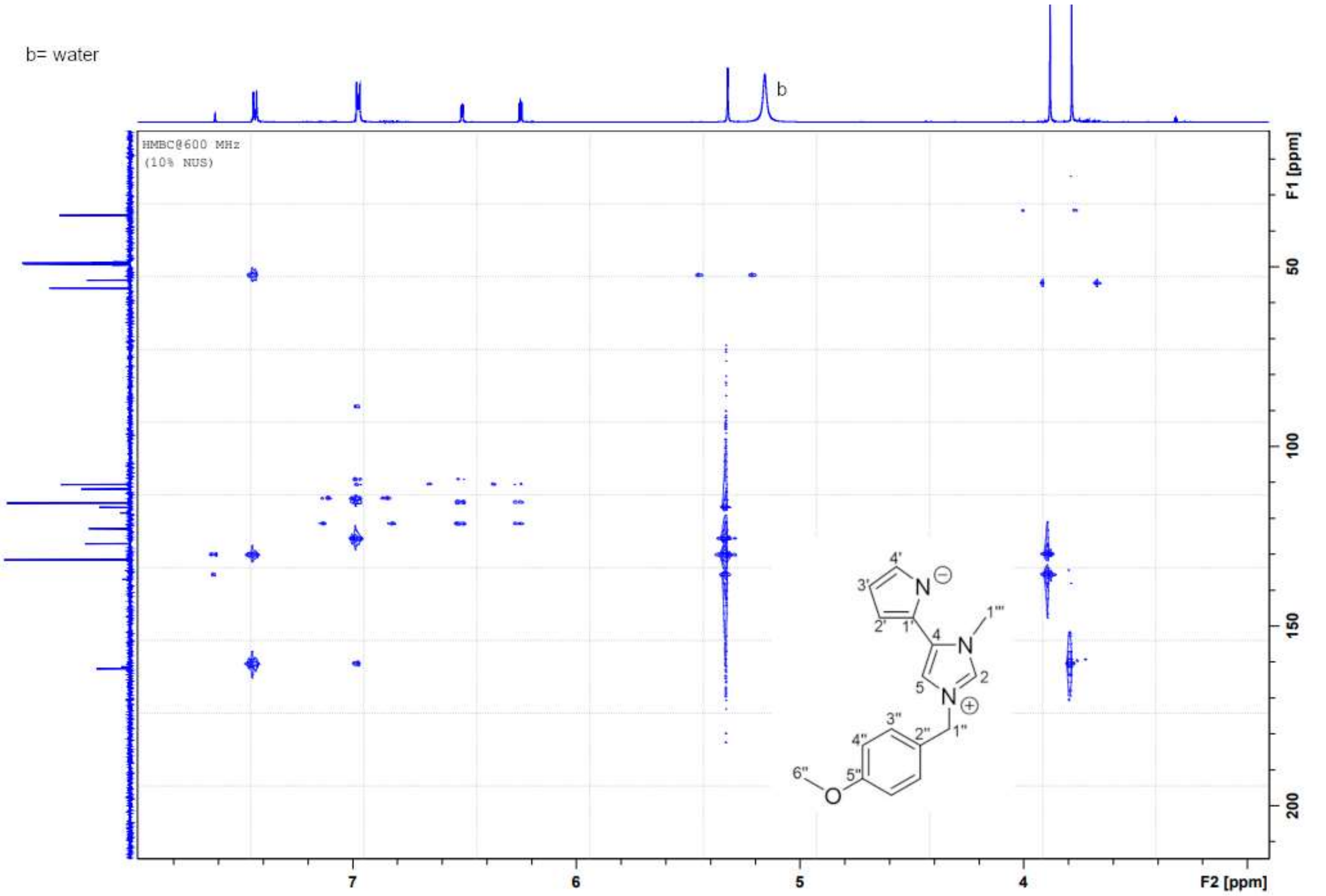

Figure S110. HMBC-NMR of compound 8g

¹H-NMR 2-(1-Benzyl-3-(tert-butyl)-1H-imidazol-3-ium-4-yl)pyrrol-1-ide (8h):

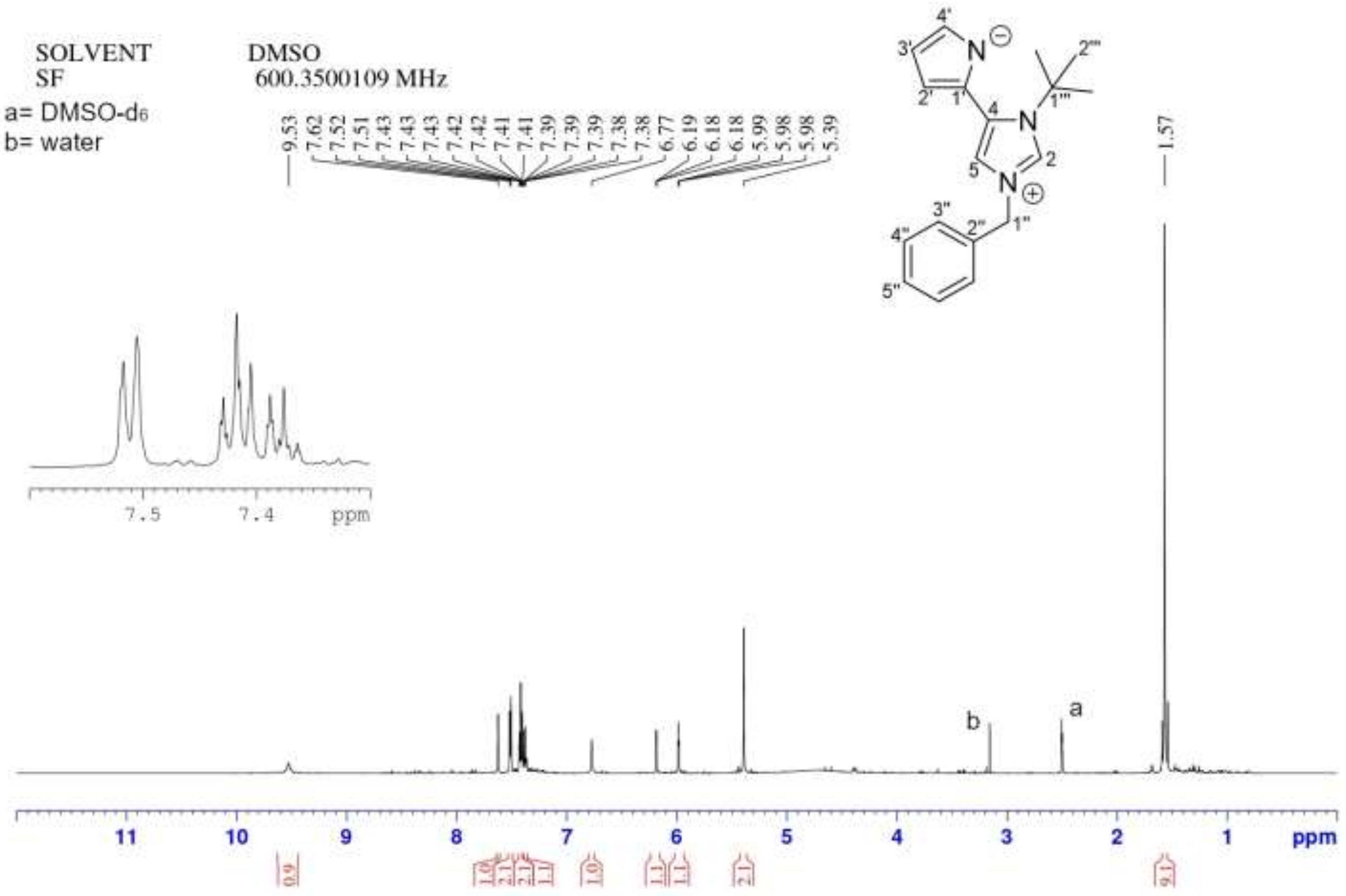

Figure S111. ¹H-NMR of compound 8h

^{13}C{^{1}H}-NMR 2-(1-Benzyl-3-(tert-butyl)-1H-imidazol-3-ium-4-yl)pyrrol-1-ide (8h):

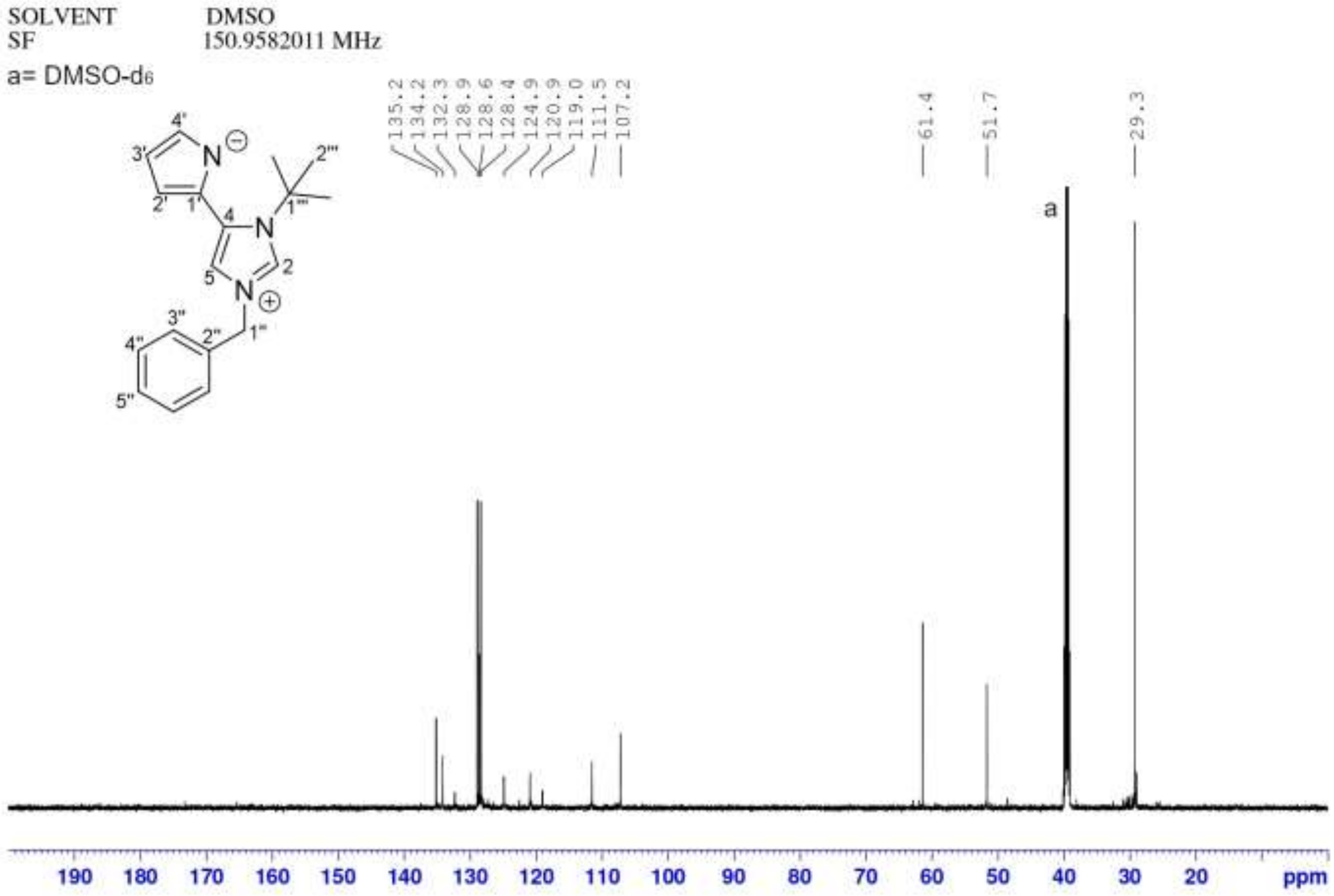

Figure S112. **^{13}C{^{1}H}-NMR of compound 8h**

$^{13}C\{^{1}H\}$**-DEPT-NMR 2-(1-Benzyl-3-(tert-butyl)-1H-imidazol-3-ium-4-yl)pyrrol-1-ide (8h):**

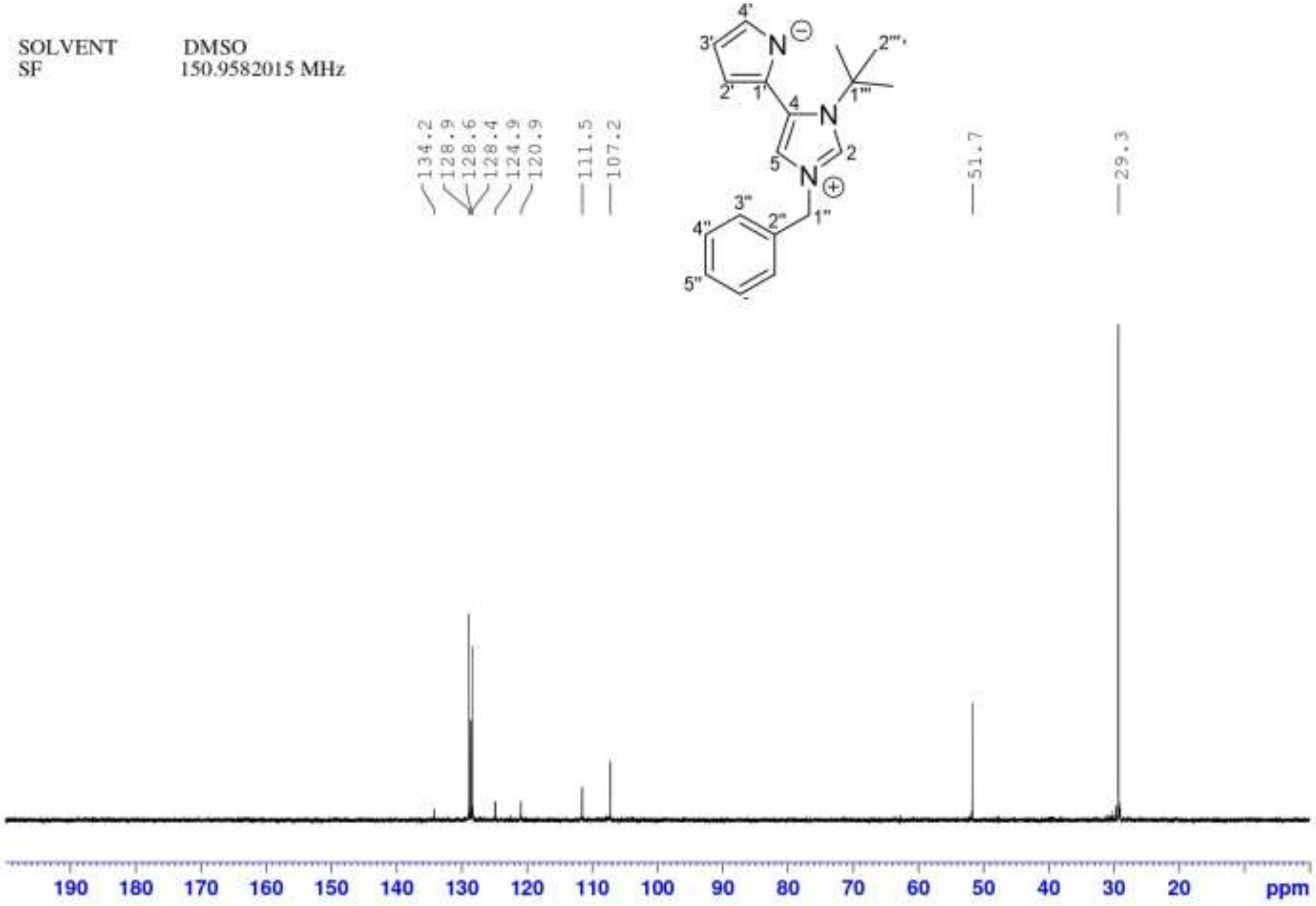

Figure S113. $^{13}C\{^{1}H\}$**-DEPT-NMR of compound 8h**

HSQC-NMR 2-(1-Benzyl-3-(tert-butyl)-1H-imidazol-3-ium-4-yl)pyrrol-1-ide (8h):

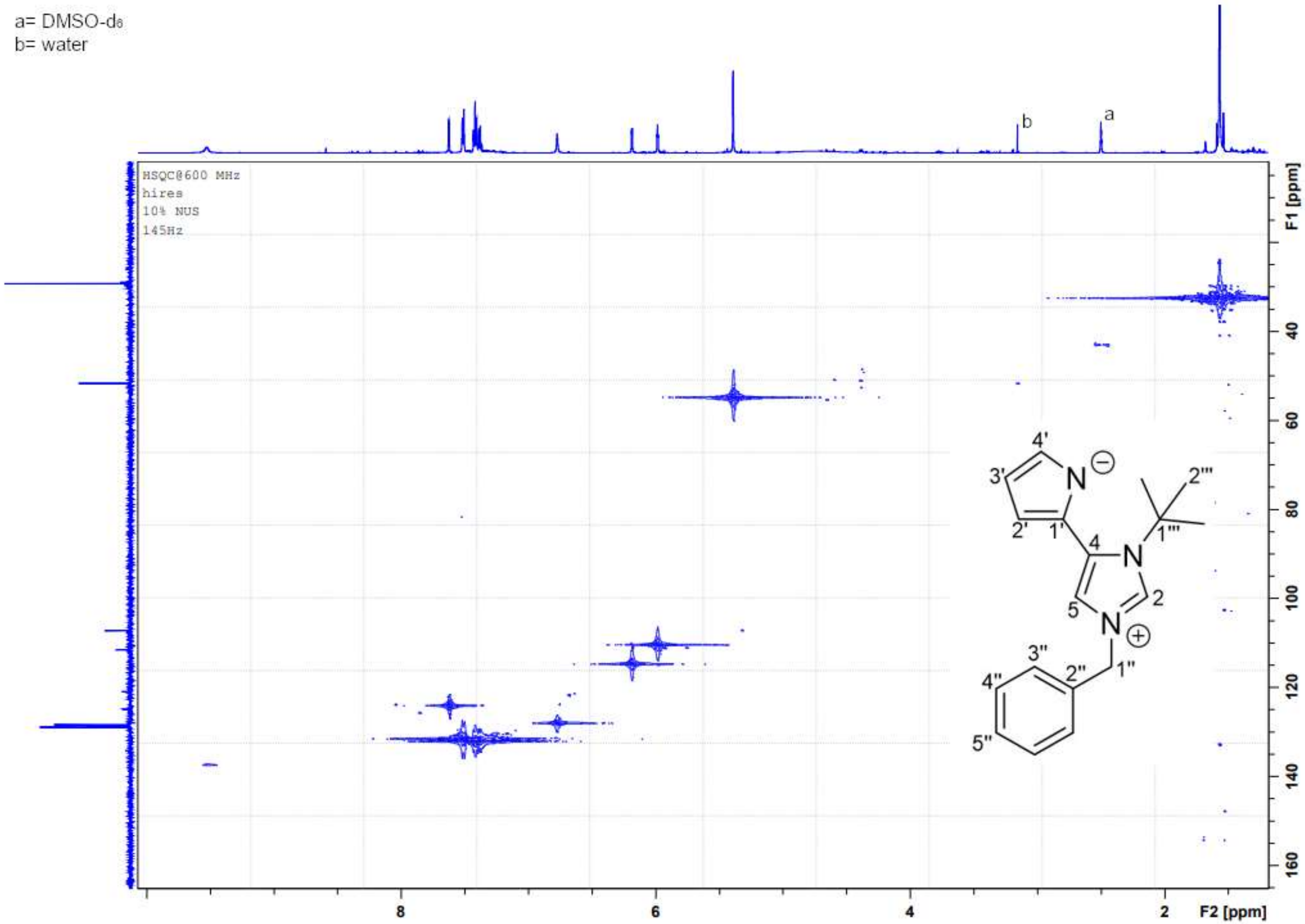

Figure S114. HSQC-NMR of compound 8h

HMBC-NMR 2-(1-Benzyl-3-(tert-butyl)-1H-imidazol-3-ium-4-yl)pyrrol-1-ide (8h):

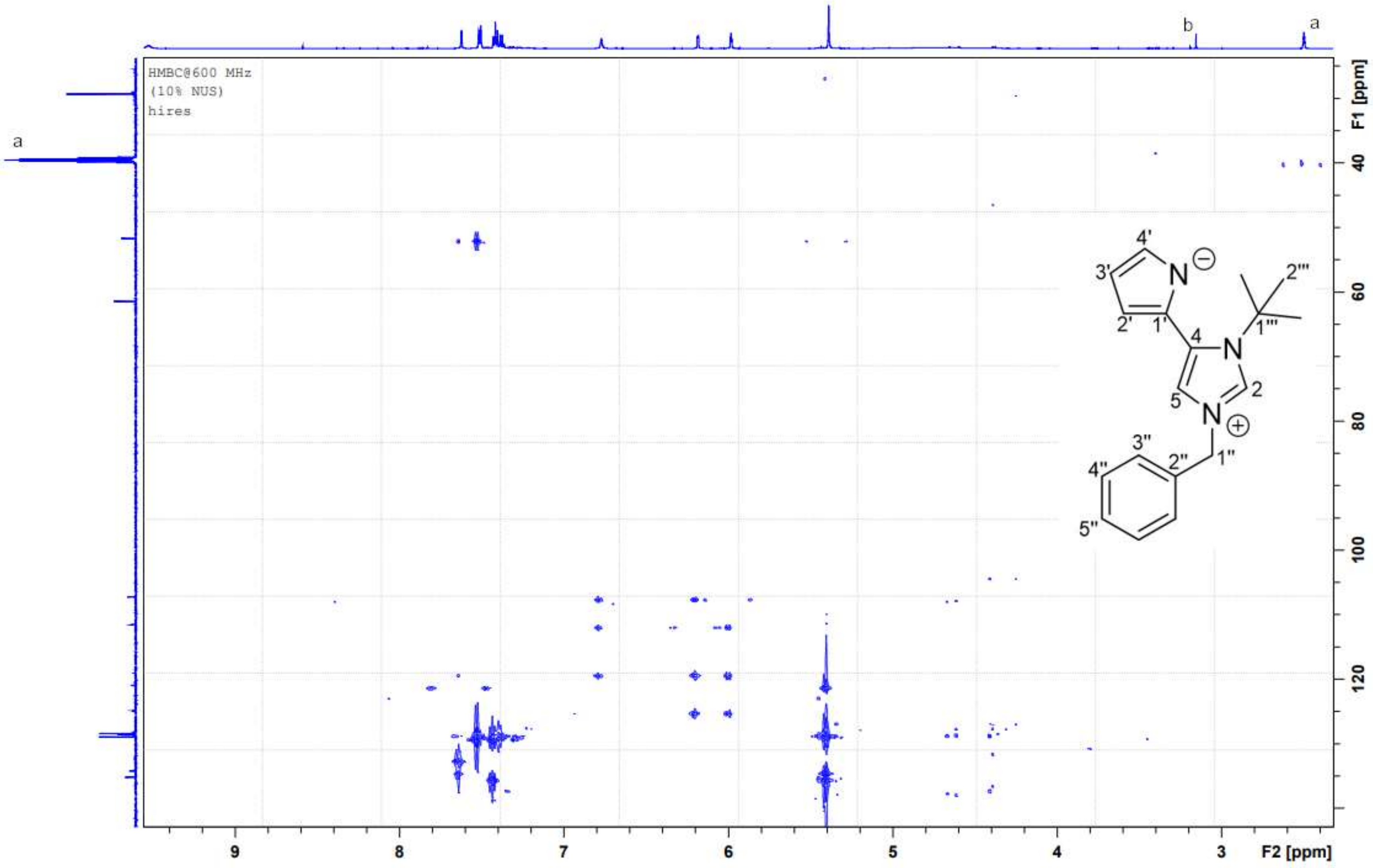

Figure S115. HMBC-NMR of compound 8h

^{1}H-NMR 2-(3-(tert-Butyl)-1-(4-methylbenzyl)-1H-imidazol-3-ium-4-yl)pyrrol-1-ide (8i):

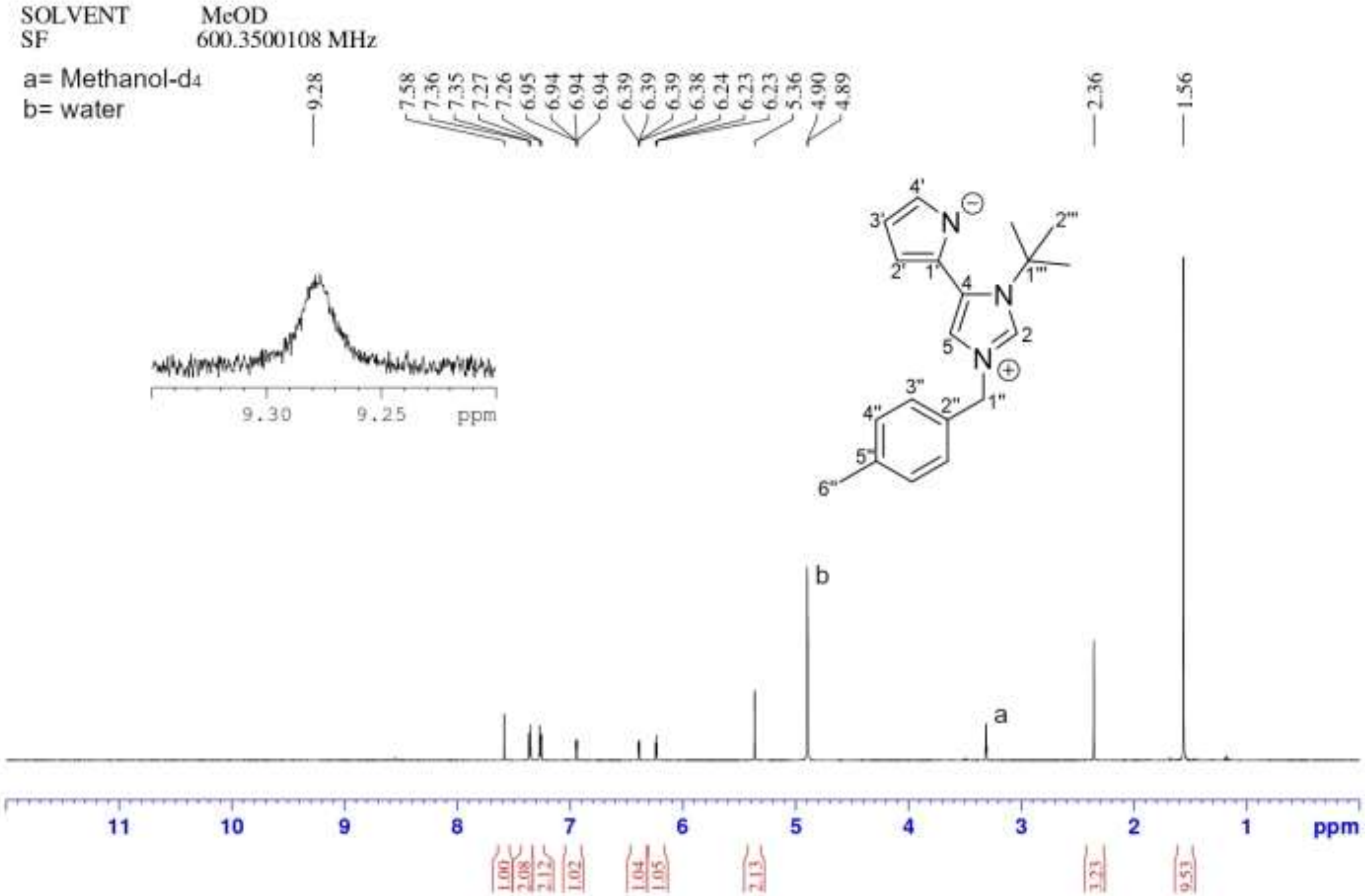

Figure S116. ^{1}H-NMR of compound 8i

^{2}H-NMR 2-(3-(tert-Butyl)-1-(4-methylbenzyl)-1H-imidazol-3-ium-5-yl)pyrrol-1-ide (8i):

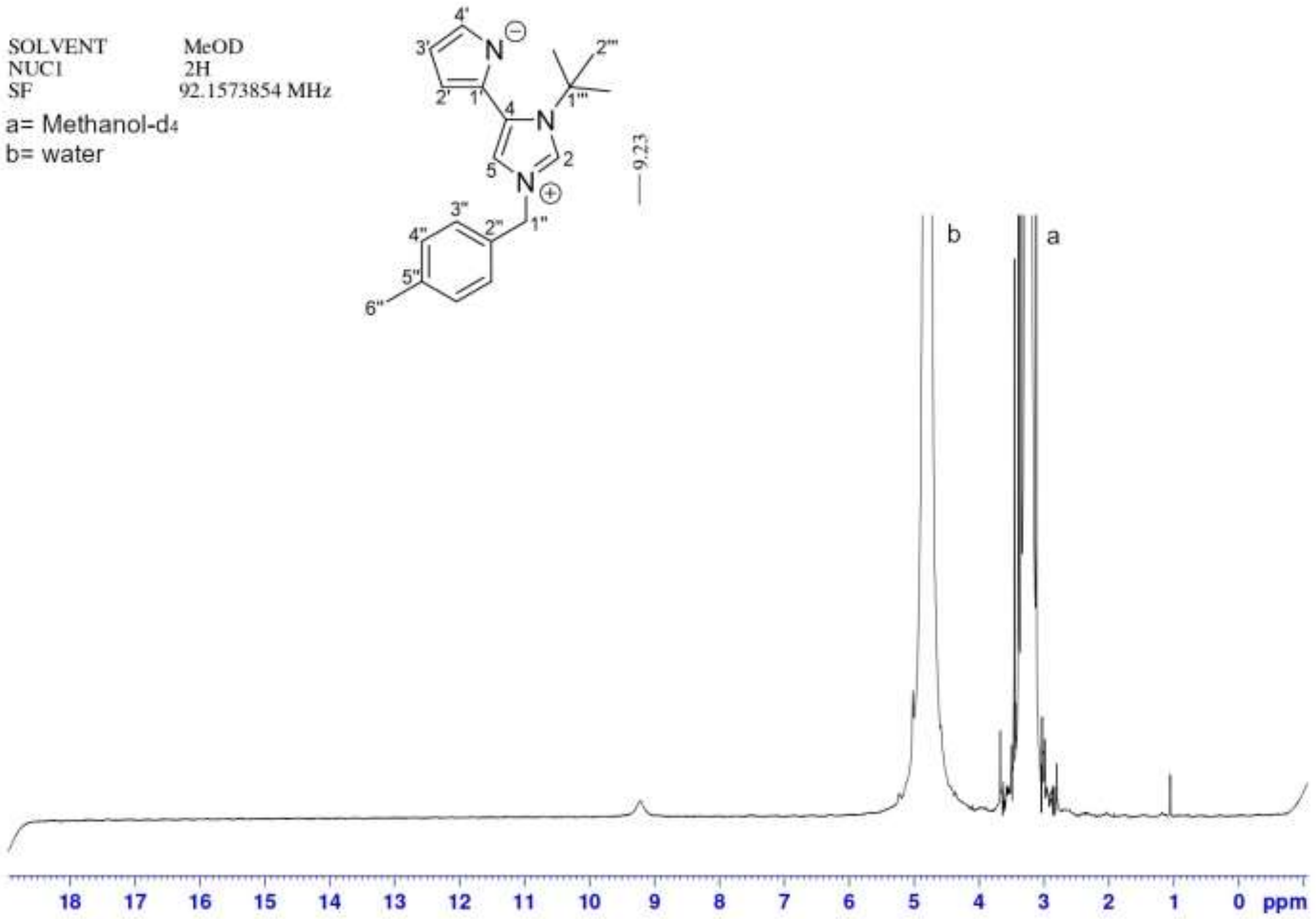

Figure S117. ^{2}H-NMR of compound 8i

$^{13}C\{^{1}H\}$-NMR 2-(3-(tert-Butyl)-1-(4-methylbenzyl)-1H-imidazol-3-ium-4-yl)pyrrol-1-ide (8i):

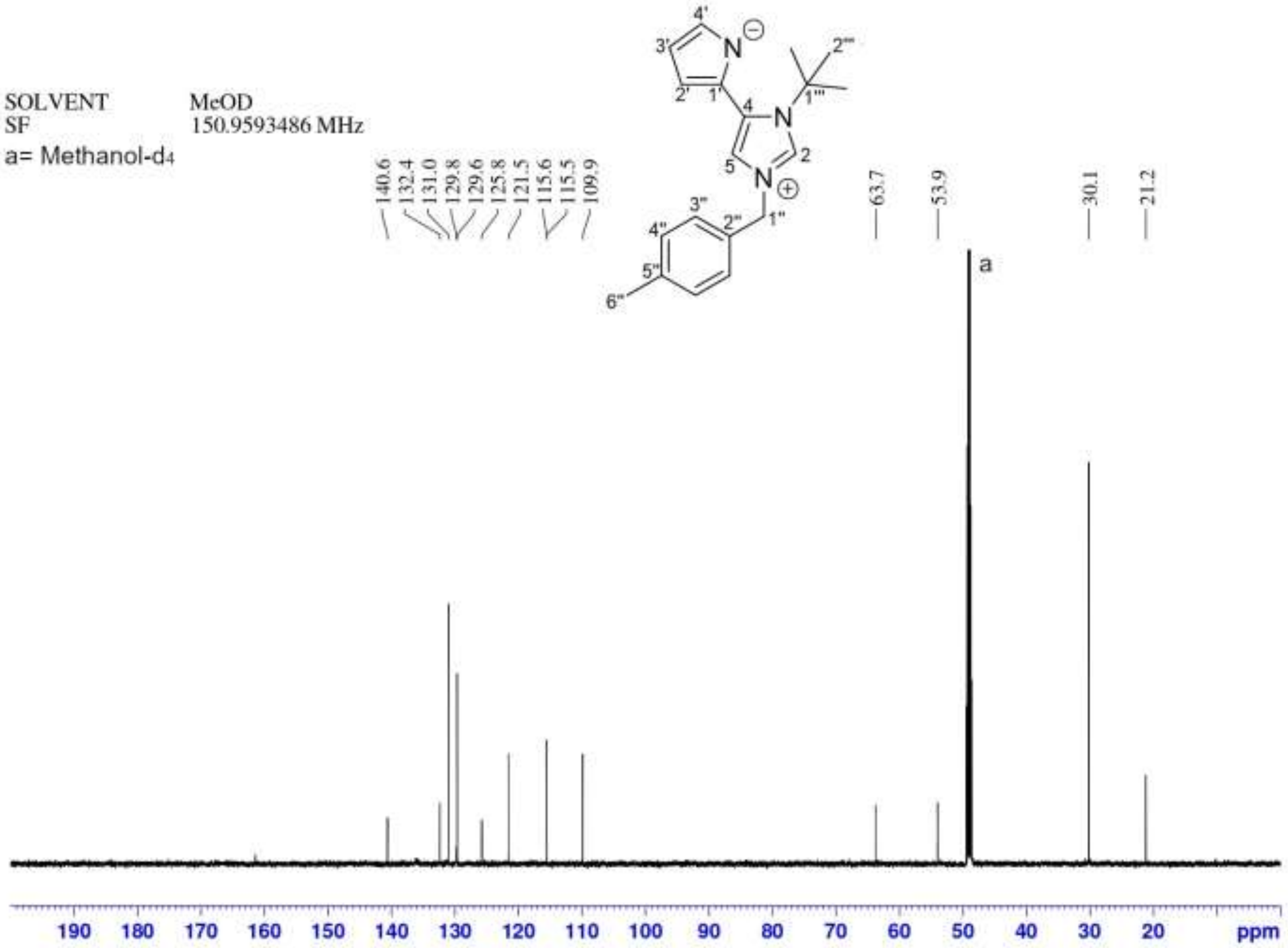

Figure S118. **$^{13}C\{^{1}H\}$-NMR of compound 8i**

$^{13}C\{^{1}H\}$-DEPT-NMR 2-(3-(tert-Butyl)-1-(4-methylbenzyl)-1H-imidazol-3-ium-4-yl)pyrrol-1-ide (8i):

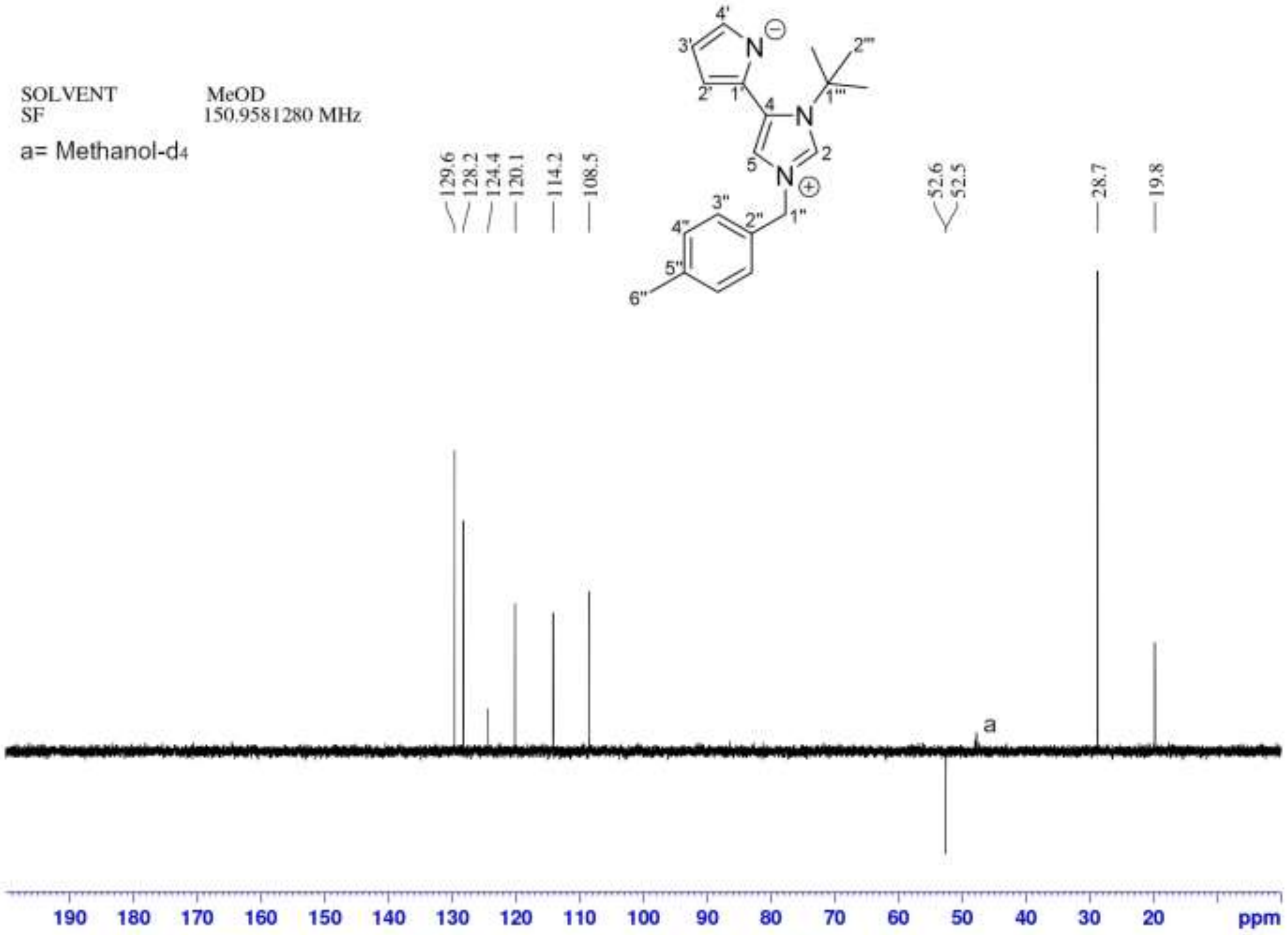

Figure S119. **$^{13}C\{^{1}H\}$-DEPT-NMR of compound 8i**

HSQC-NMR 2-(3-(tert-Butyl)-1-(4-methylbenzyl)-1H-imidazol-3-ium-4-yl)pyrrol-1-ide (8i):

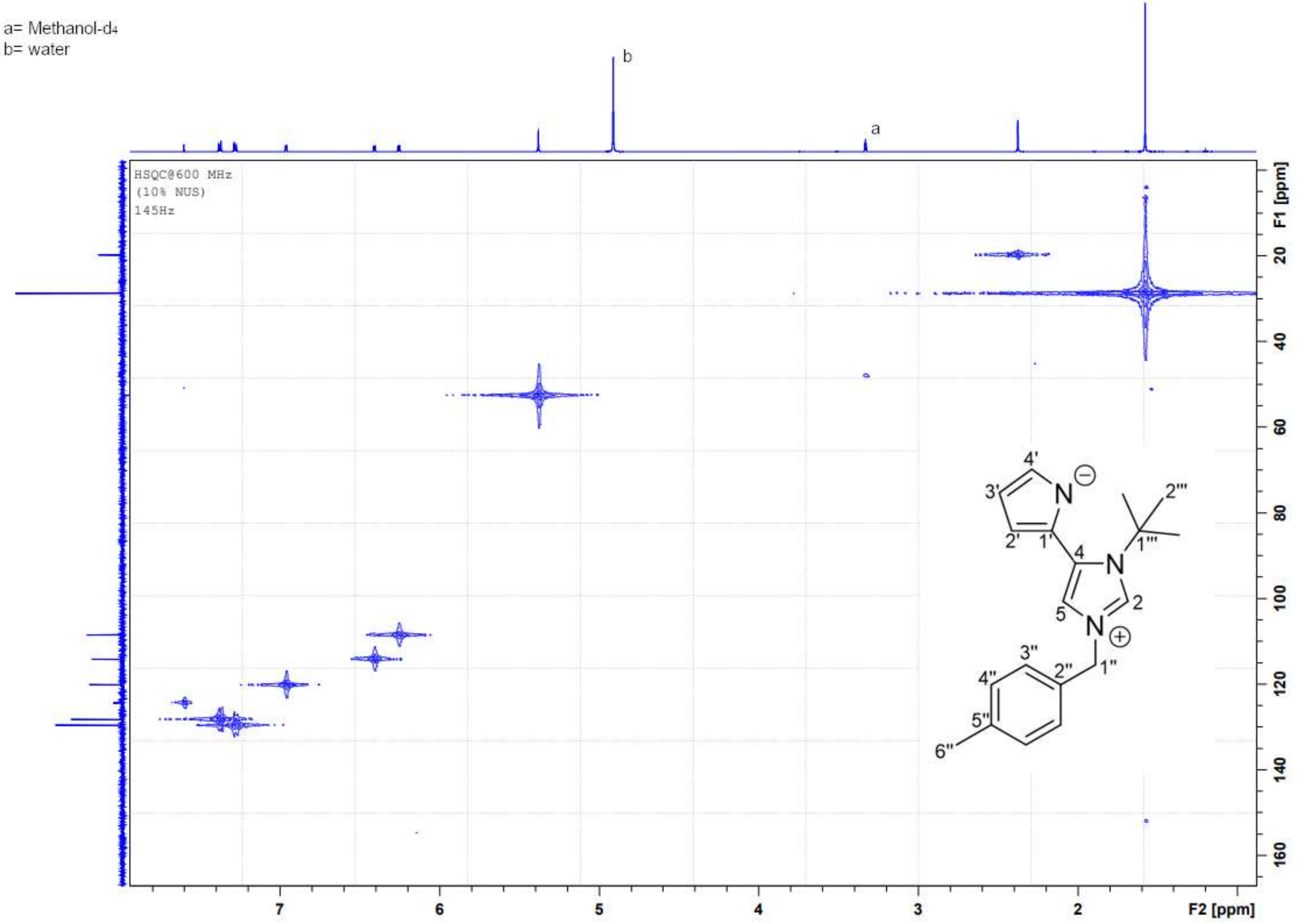

Figure S120. HSQC-NMR of compound 8i

HMBC-NMR 2-(3-(tert-Butyl)-1-(4-methylbenzyl)-1H-imidazol-3-ium-4-yl)pyrrol-1-ide (8i):

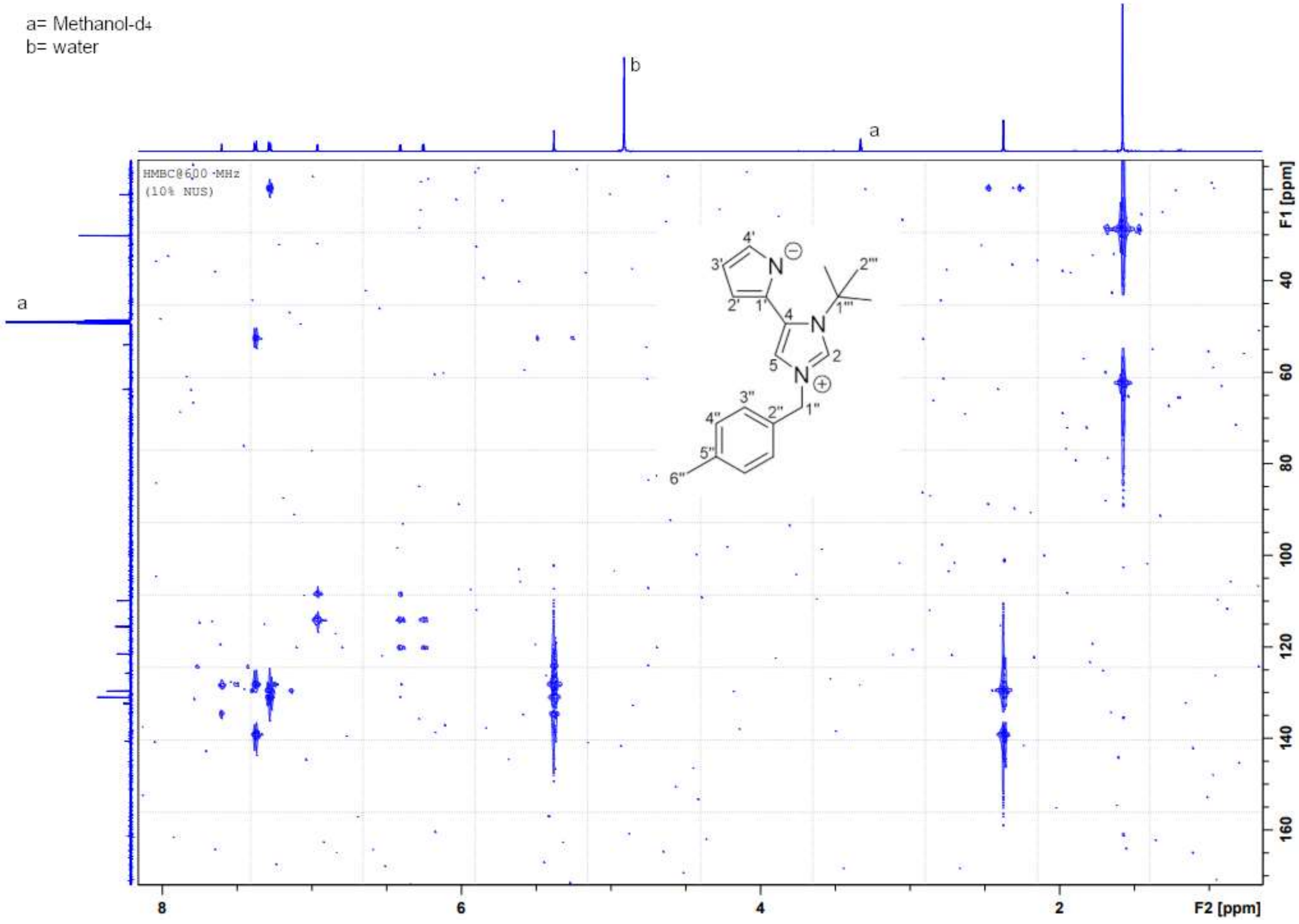

Figure S121. HMBC-NMR of compound 8i

¹H-NMR 1,3-Dimethyl-4-(1H-pyrrol-2-yl)-1,3-dihydro-2H-imidazole-2-selenone (9a):

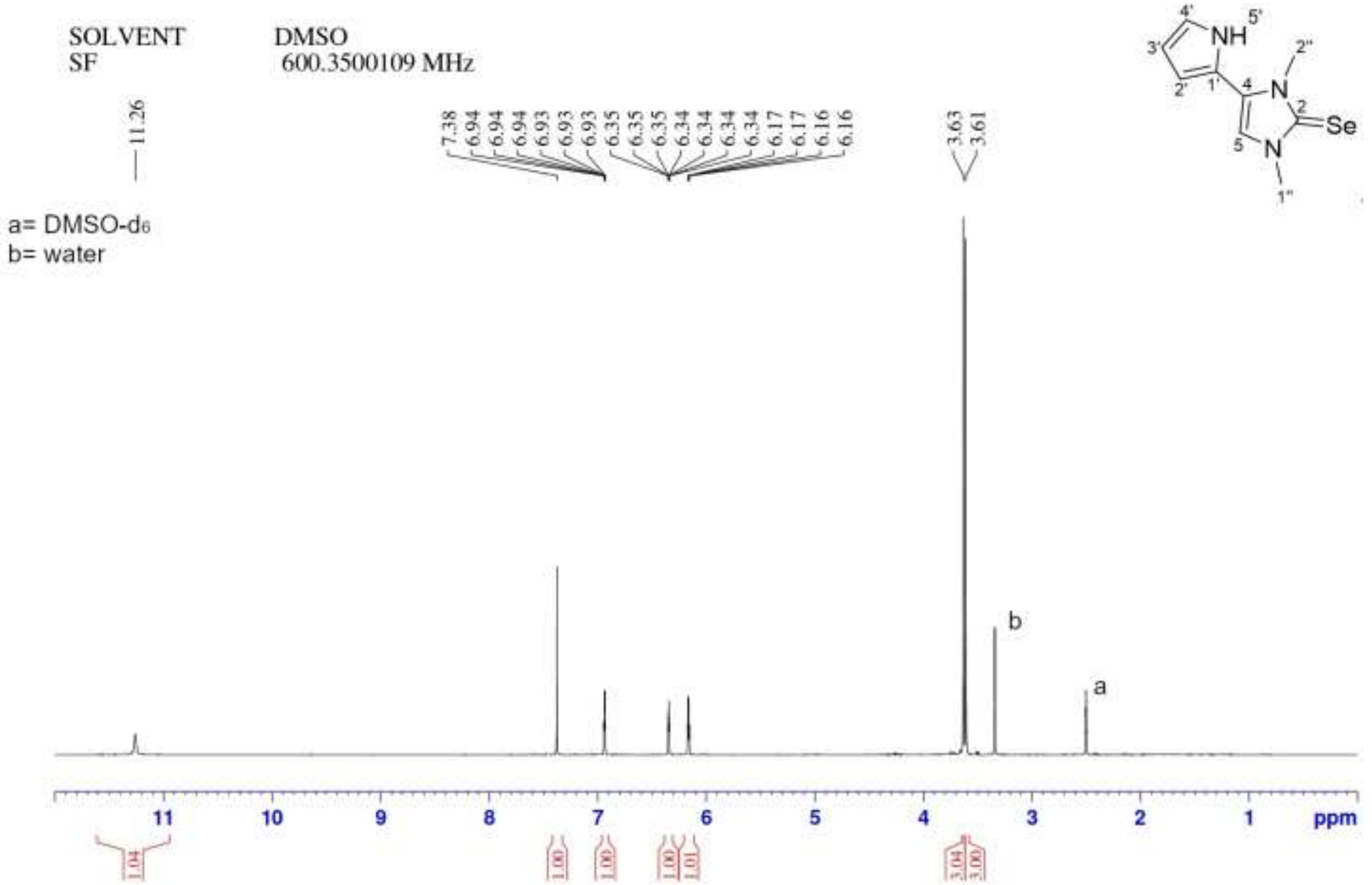

Figure S122. ¹H-NMR of compound 9a

$^{13}C\{^1H\}$-NMR 1,3-Dimethyl-4-(1H-pyrrol-2-yl)-1,3-dihydro-2H-imidazole-2-selenone (9a):

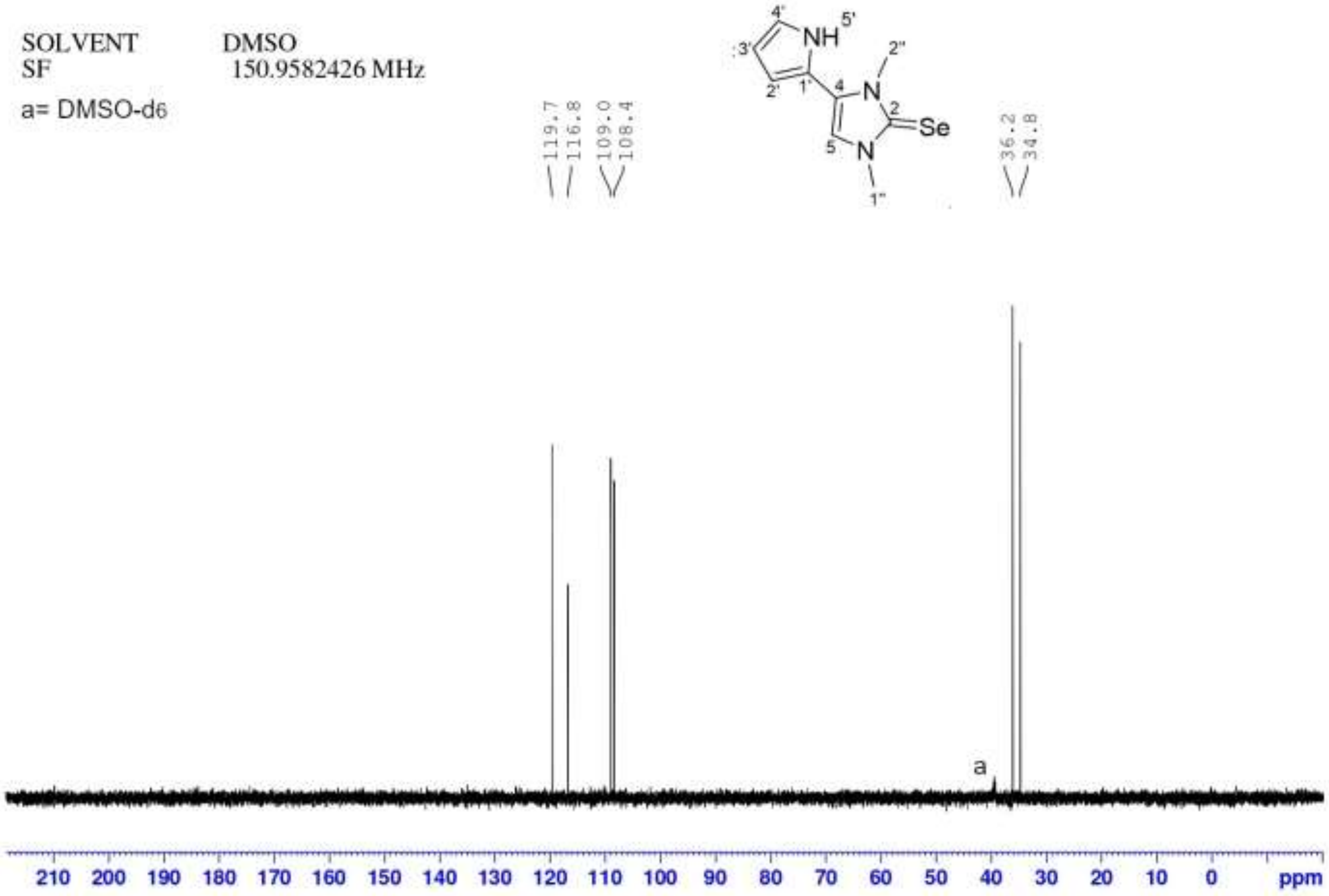

Figure S123. **$^{13}C\{^1H\}$-NMR of compound 9a**

$^{13}C\{^{1}H\}$-DEPT-NMR 1,3-Dimethyl-4-(1H-pyrrol-2-yl)-1,3-dihydro-2H-imidazole-2-selenone (9a):

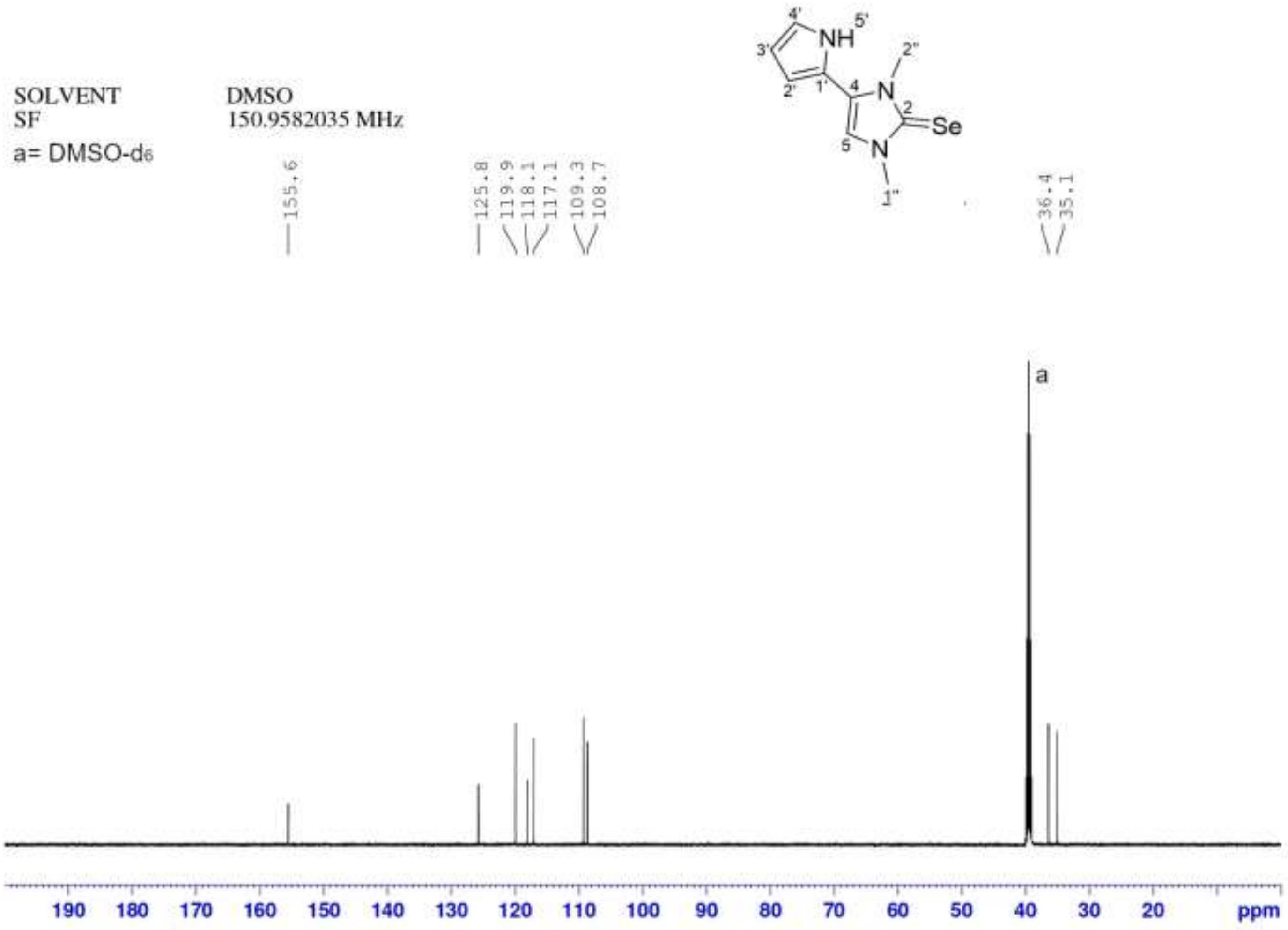

Figure S124. **$^{13}C\{^{1}H\}$-DEPT-NMR of compound 9a**

^{77}Se-NMR 1,3-Dimethyl-4-(1H-pyrrol-2-yl)-1,3-dihydro-2H-imidazole-2-selenone (9a):

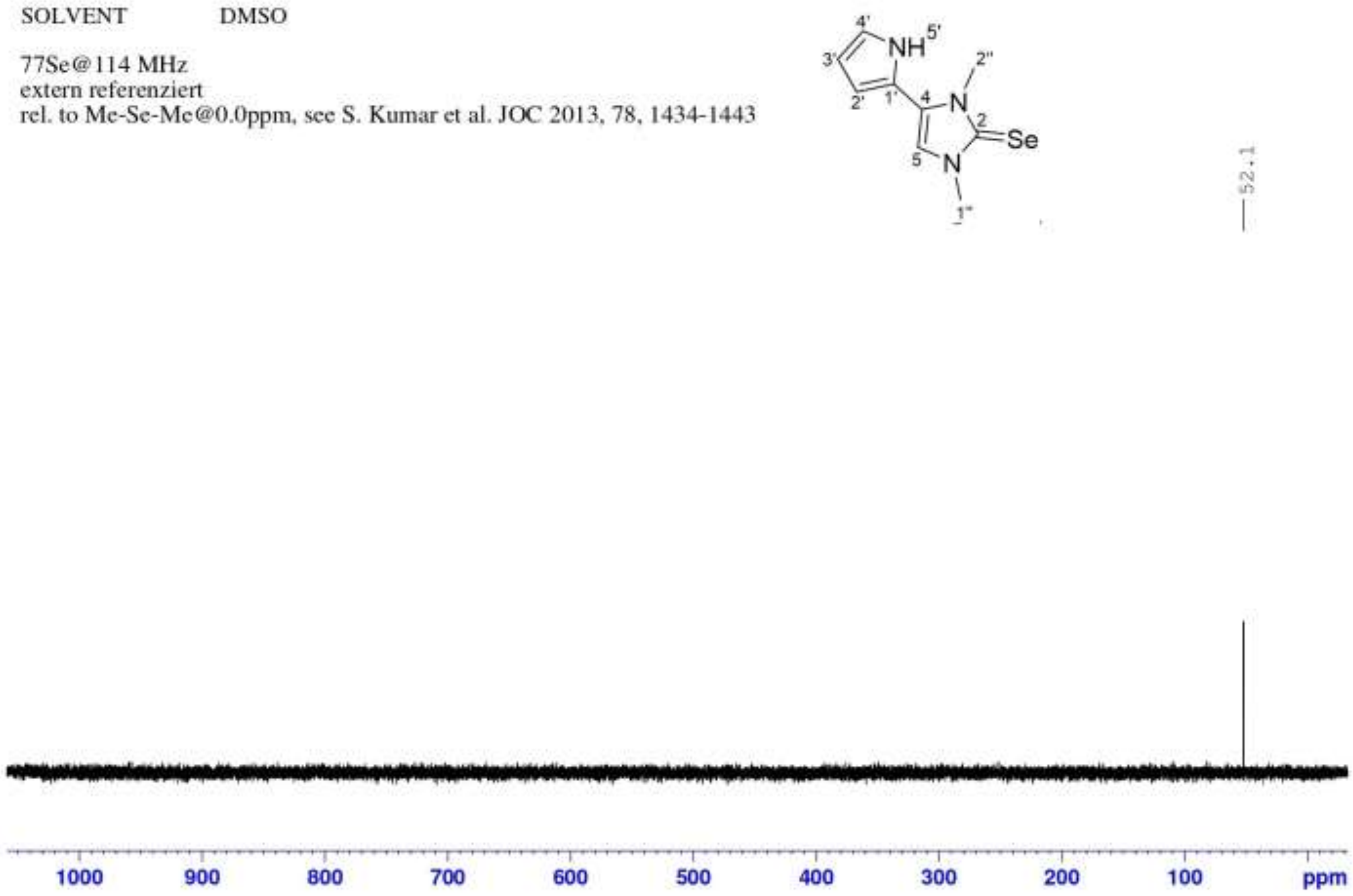

Figure S125. ^{77}Se-NMR of compound 9a

HSQC-NMR 1,3-Dimethyl-4-(1H-pyrrol-2-yl)-1,3-dihydro-2H-imidazole-2-selenone (9a):

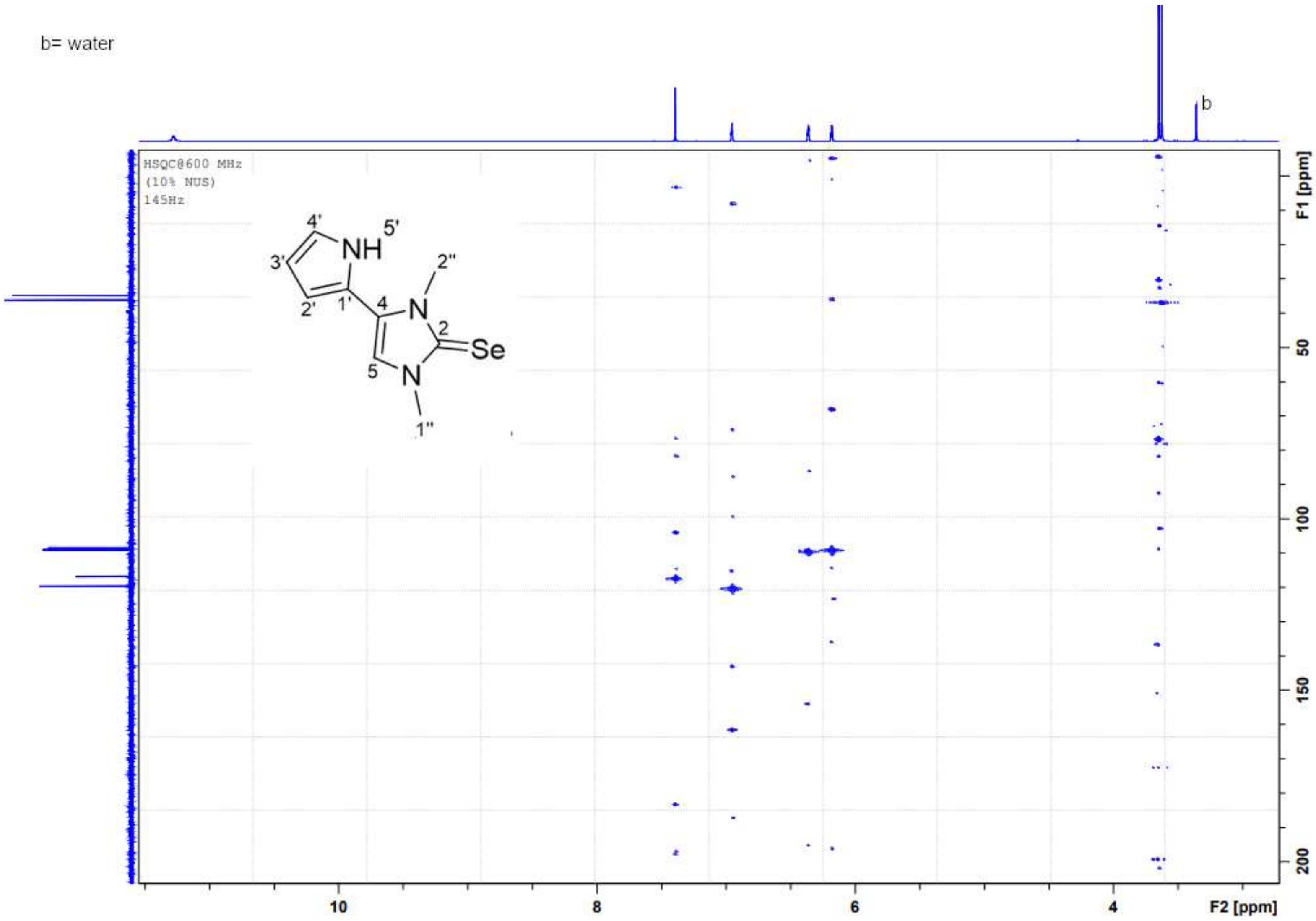

Figure S126. HSQC-NMR of compound 9a

HMBC-NMR 1,3-Dimethyl-4-(1H-pyrrol-2-yl)-1,3-dihydro-2H-imidazole-2-selenone (9a):

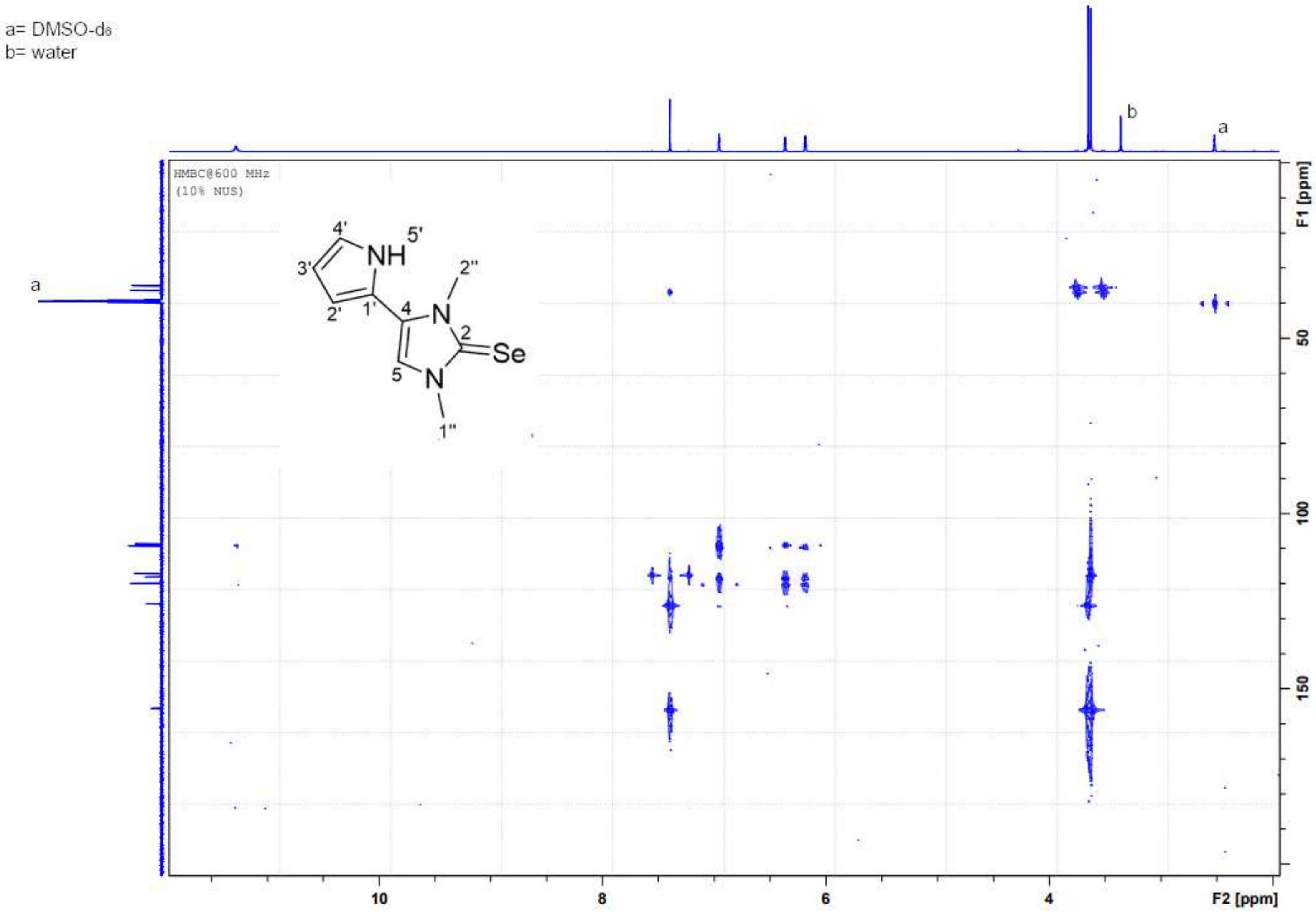

Figure S127. HMBC-NMR of compound 9a

^{1}H-NMR 1,3-Dimethyl-5-phenyl-4-(1H-pyrrol-2-yl)-1,3-dihydro-2H-imidazole-2-selenone (9b):

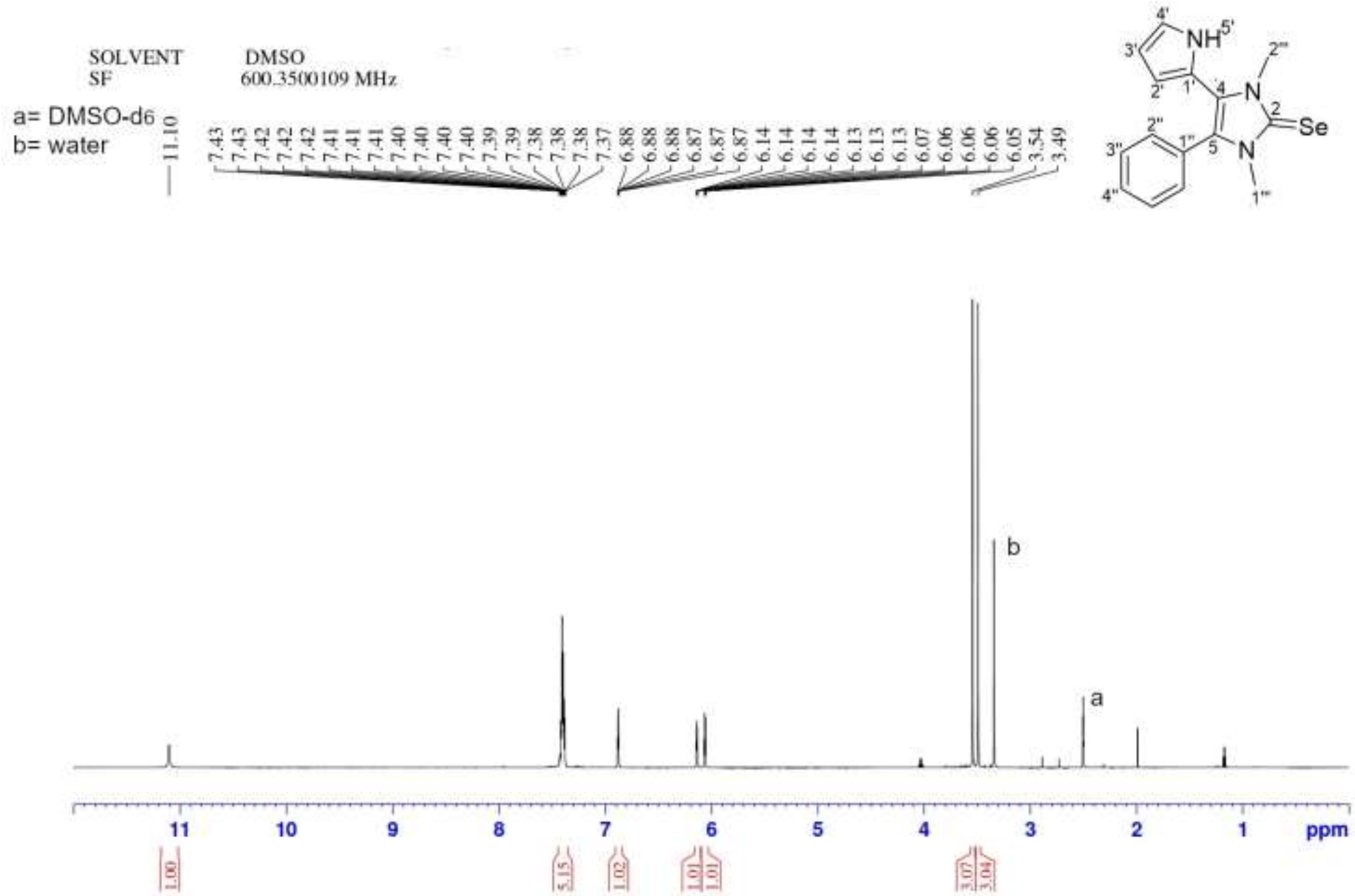

Figure S128. ^{1}H-NMR of compound 9b

$^{13}C\{^1H\}$-NMR 1,3-Dimethyl-5-phenyl-4-(1H-pyrrol-2-yl)-1,3-dihydro-2H-imidazole-2-selenone (9b):

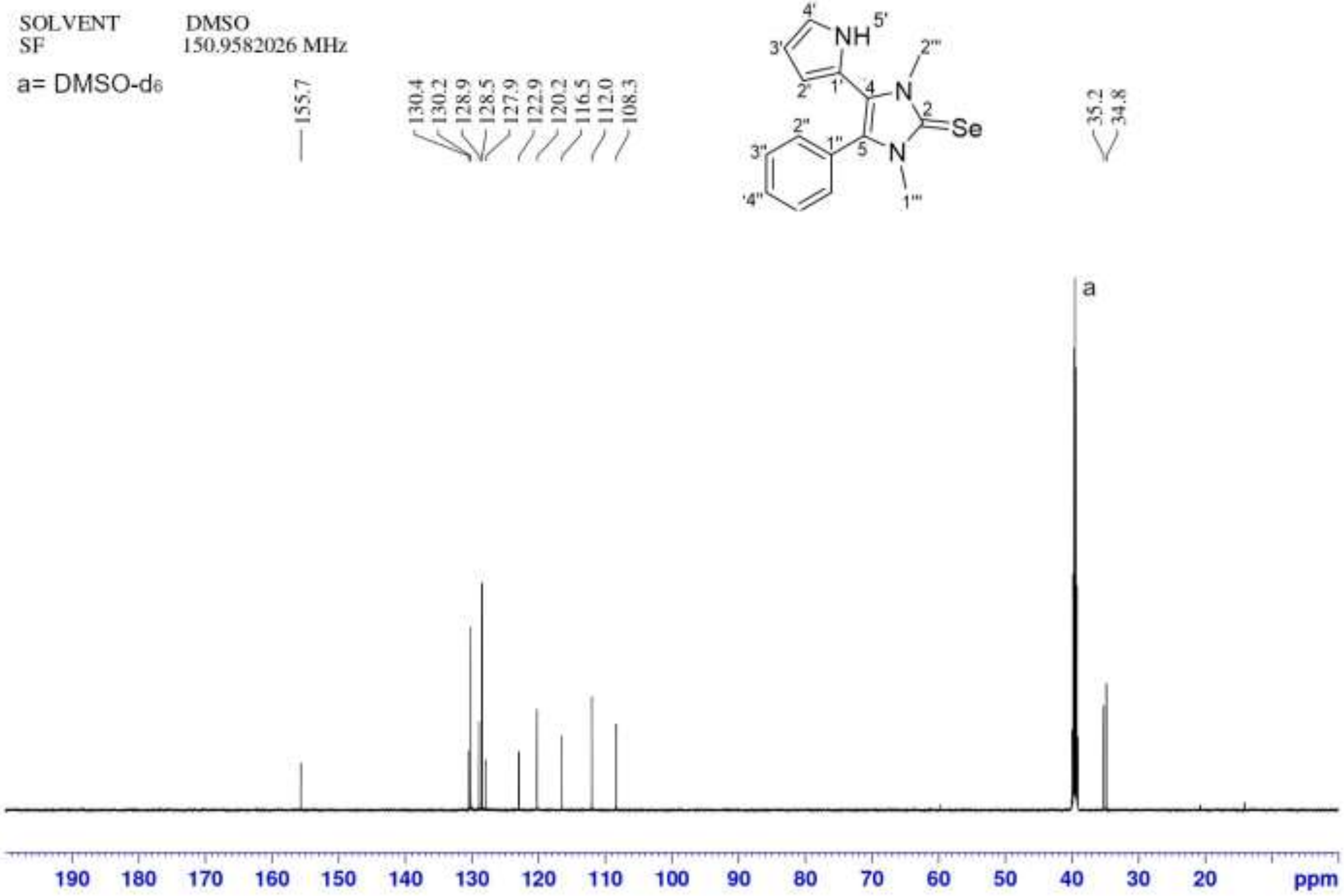

Figure S129. **$^{13}C\{^1H\}$-NMR of compound 9b**

$^{13}C\{^1H\}$-DEPT-NMR 1,3-Dimethyl-5-phenyl-4-(1H-pyrrol-2-yl)-1,3-dihydro-2H-imidazole-2-selenone (9b):

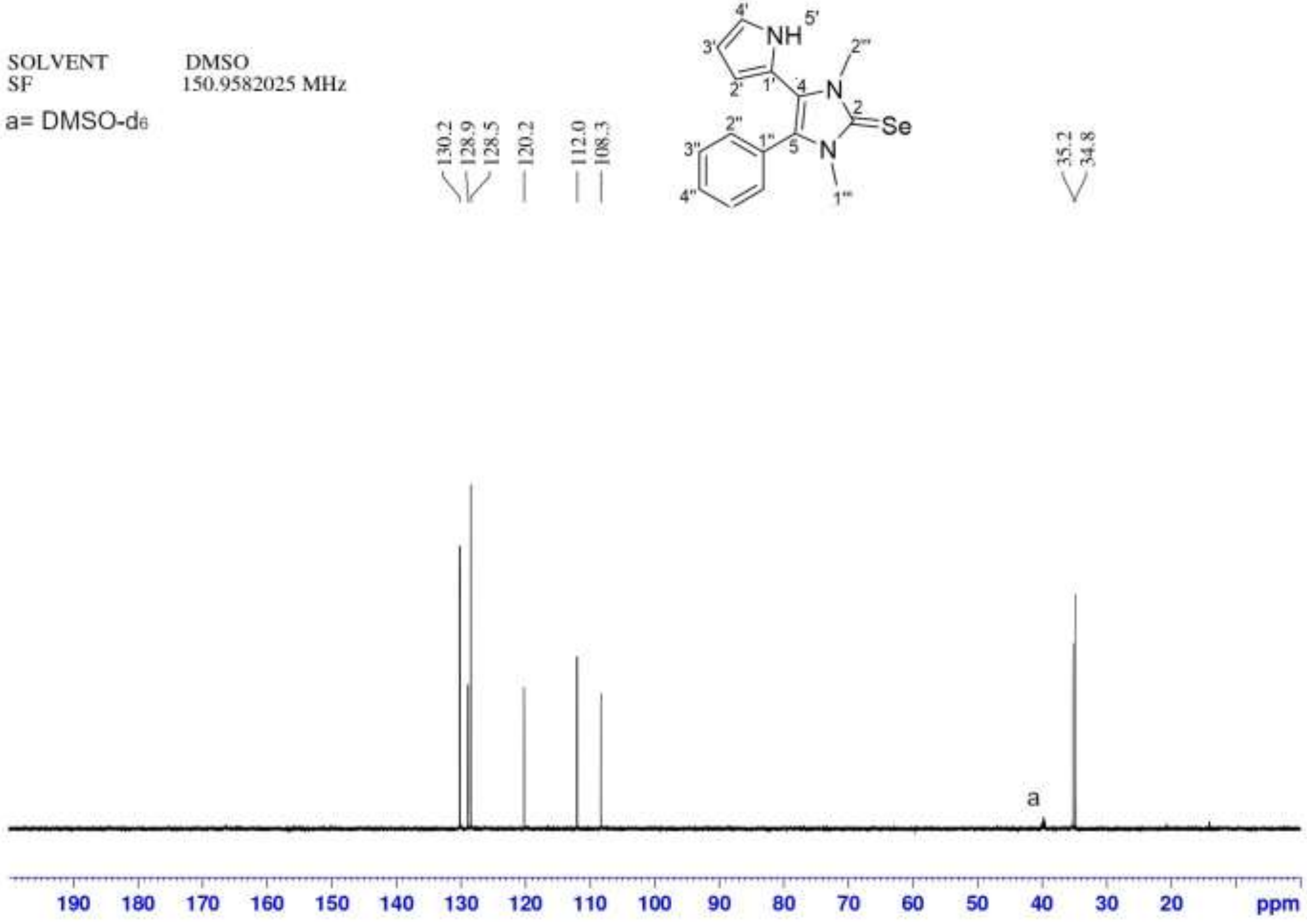

Figure S130. $^{13}C\{^1H\}$**-DEPT-NMR of compound 9b**

^{77}**Se-NMR 1,3-Dimethyl-5-phenyl-4-(1H-pyrrol-2-yl)-1,3-dihydro-2H-imidazole-2-selenone (9b):**

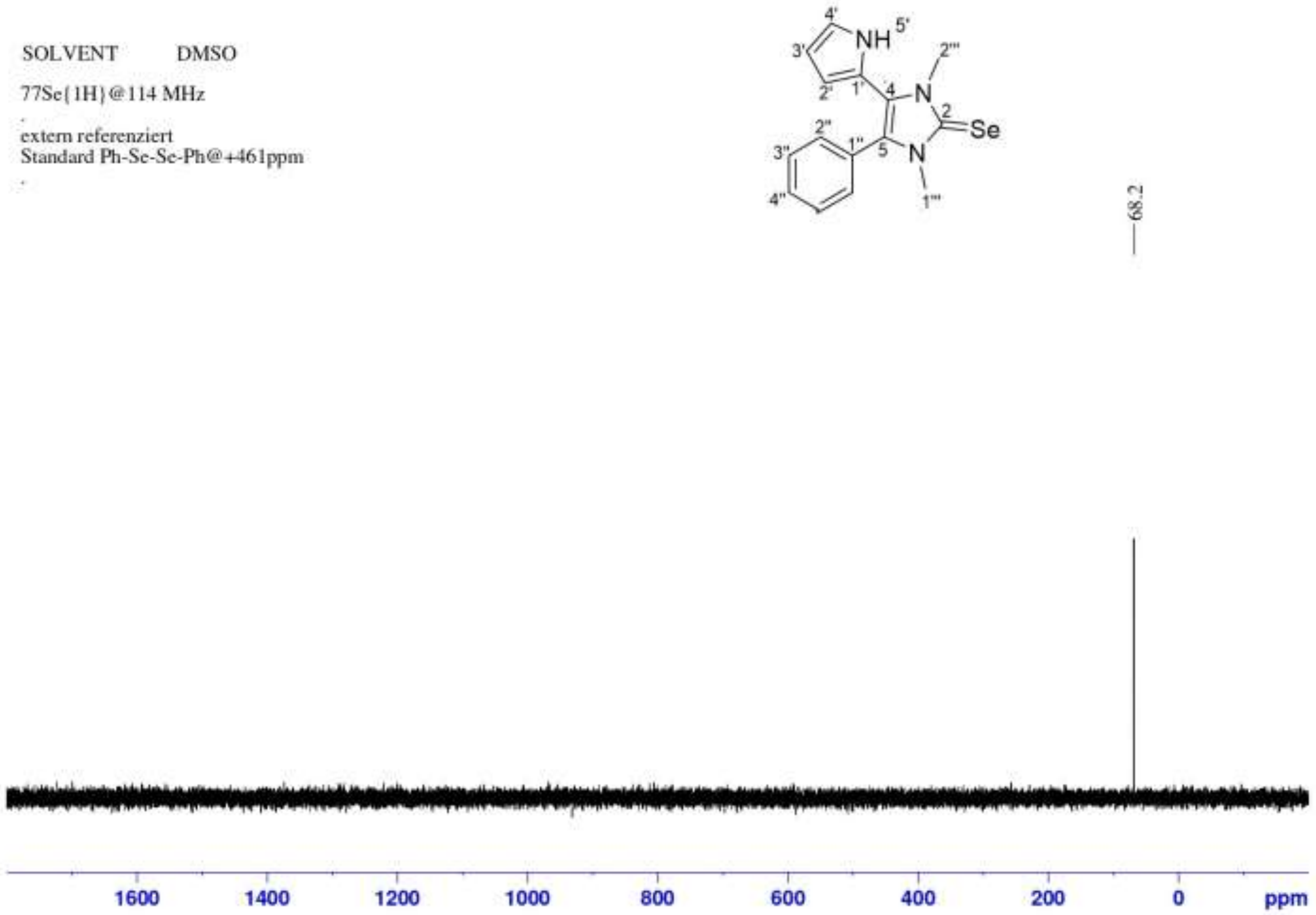

Figure S131. ^{77}**Se-NMR of compound 9b**

HSQC-NMR 1,3-Dimethyl-5-phenyl-4-(1H-pyrrol-2-yl)-1,3-dihydro-2H-imidazole-2-selenone (9b):

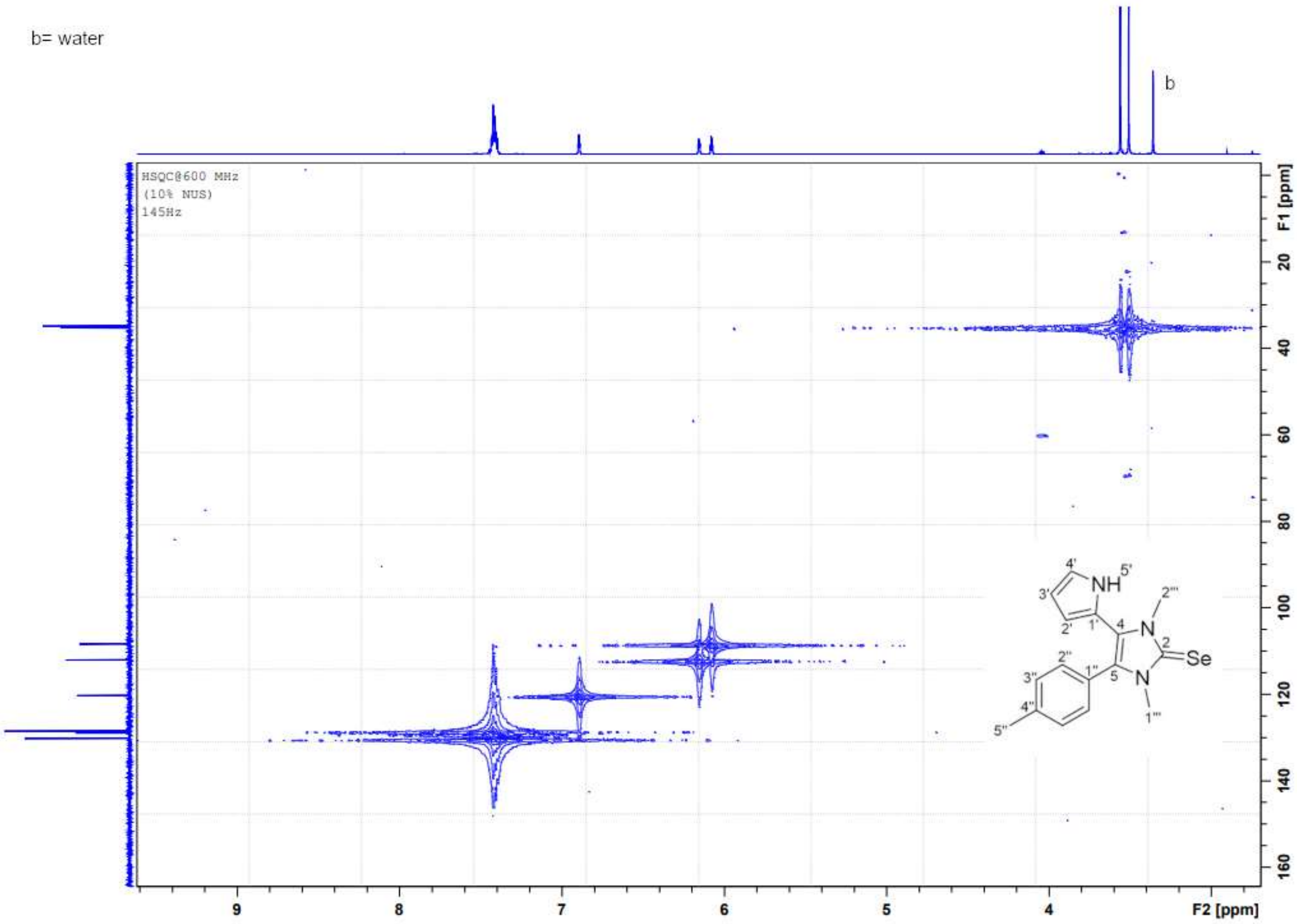

Figure S132. HSQC-NMR of compound 9b

HMBC-NMR 1,3-Dimethyl-5-phenyl-4-(1H-pyrrol-2-yl)-1,3-dihydro-2H-imidazole-2-selenone (9b):

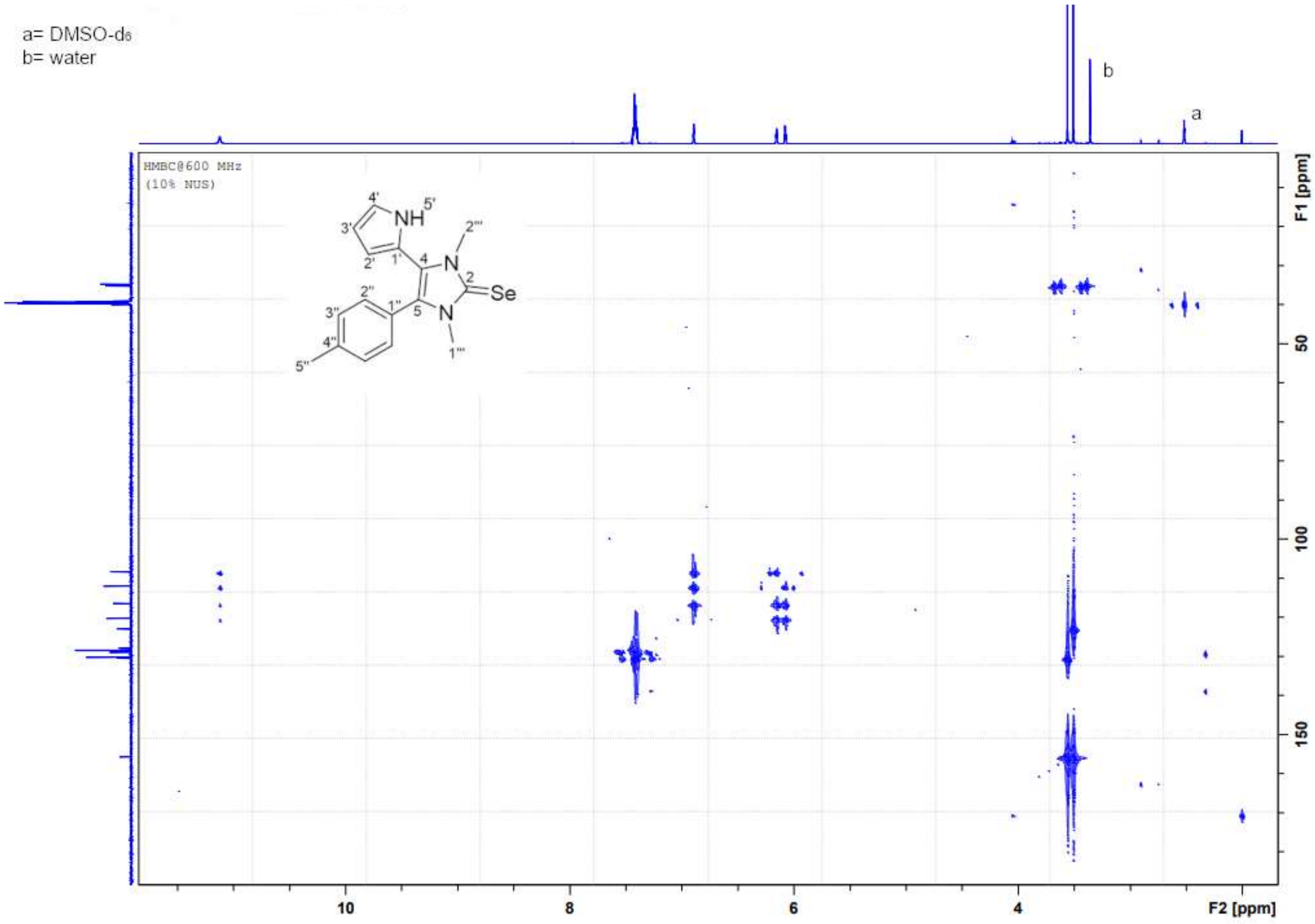

Figure S133. HMBC-NMR of compound 9b

^{1}H-NMR 1,3-Dimethyl-4-(1H-pyrrol-2-yl)-5-(p-tolyl)-1,3-dihydro-2H-imidazole-2-selenone (9c):

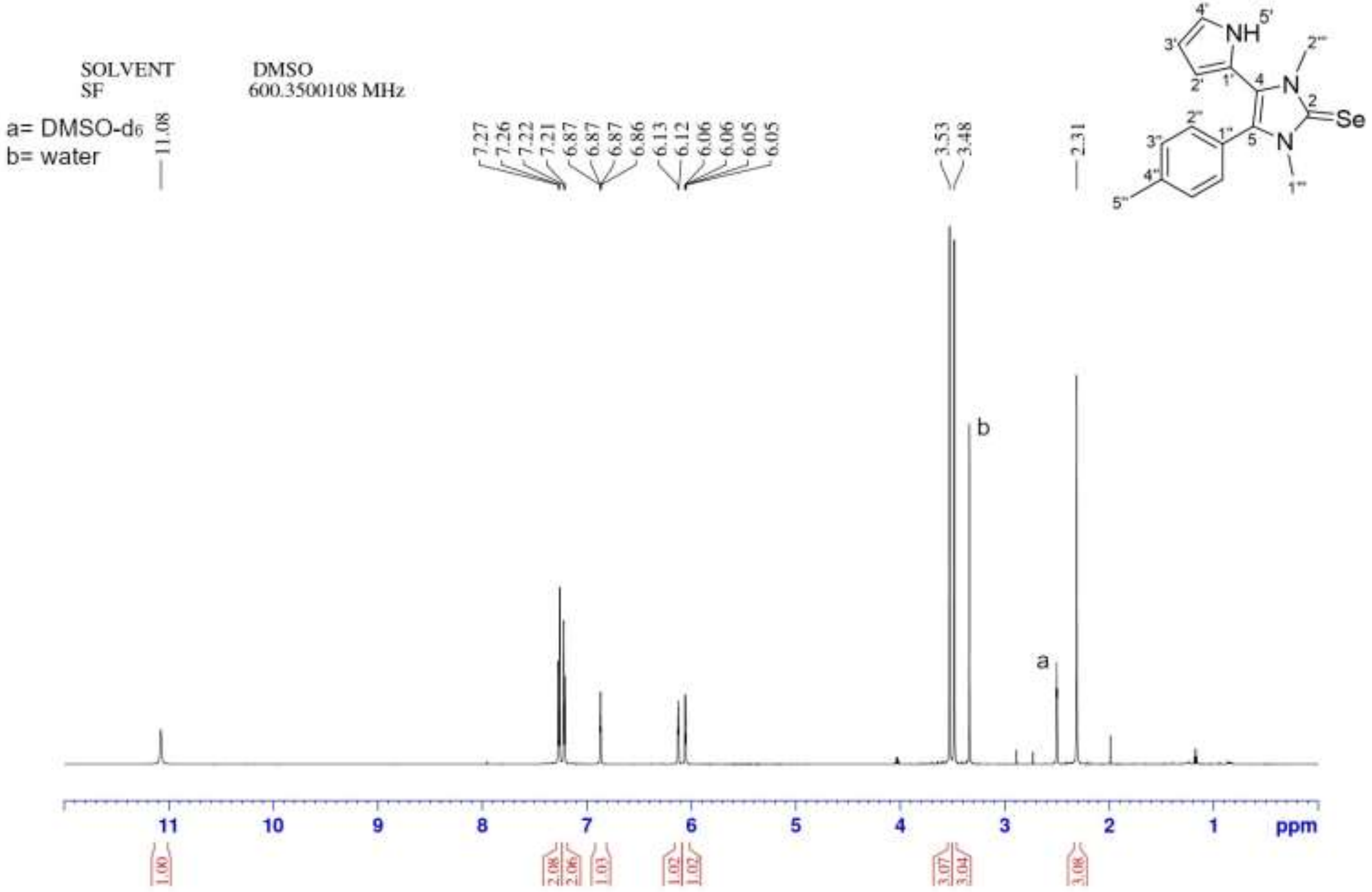

Figure S134. ^{1}H-NMR of compound 9c

$^{13}C\{^1H\}$-NMR 1,3-Dimethyl-4-(1H-pyrrol-2-yl)-5-(p-tolyl)-1,3-dihydro-2H-imidazole-2-selenone (9c):

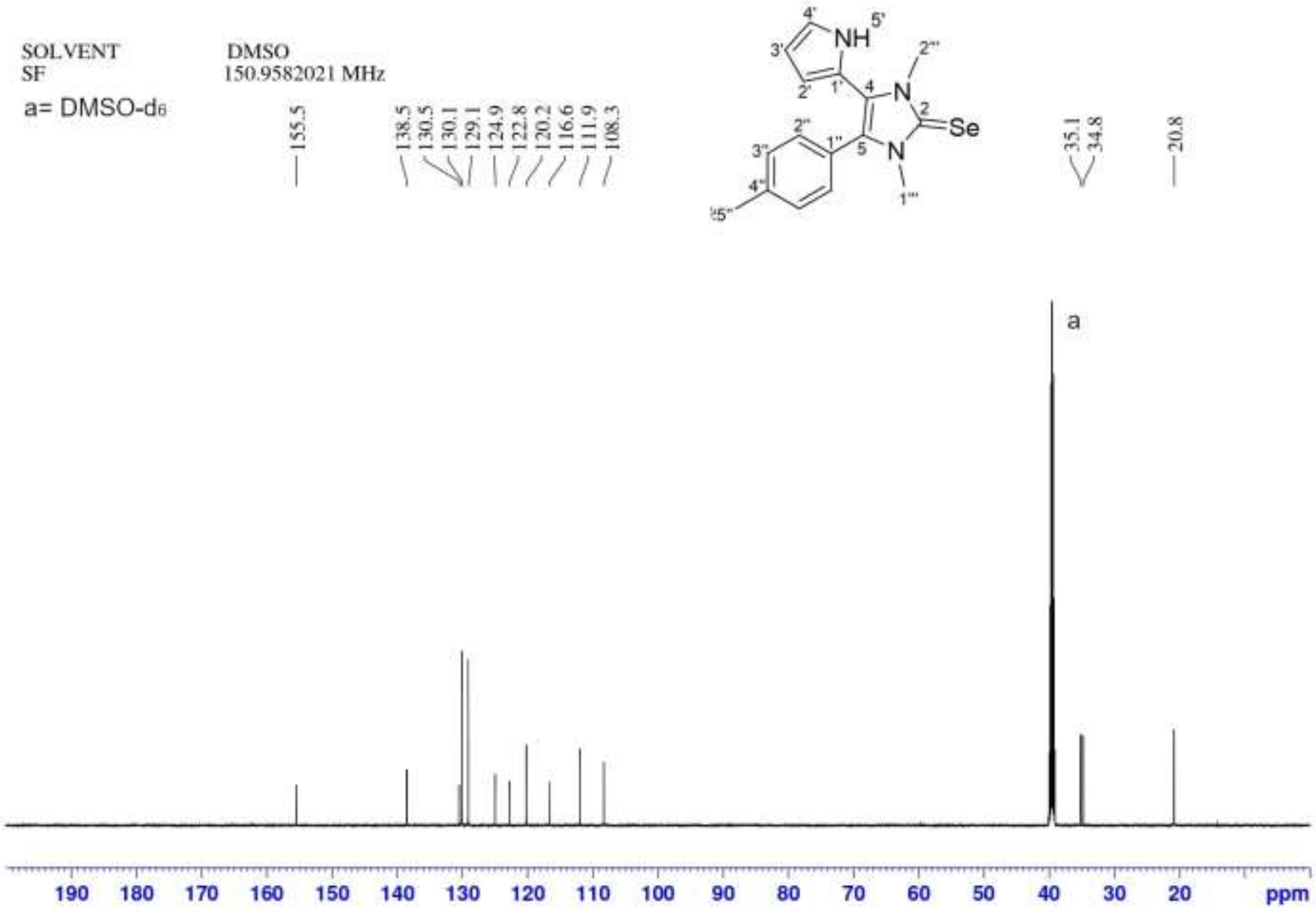

Figure S135. $^{13}C\{^1H\}$-NMR of compound 9c

$^{13}C\{^1H\}$-DEPT-NMR 1,3-Dimethyl-4-(1H-pyrrol-2-yl)-5-(p-tolyl)-1,3-dihydro-2H-imidazole-2-selenone (9c):

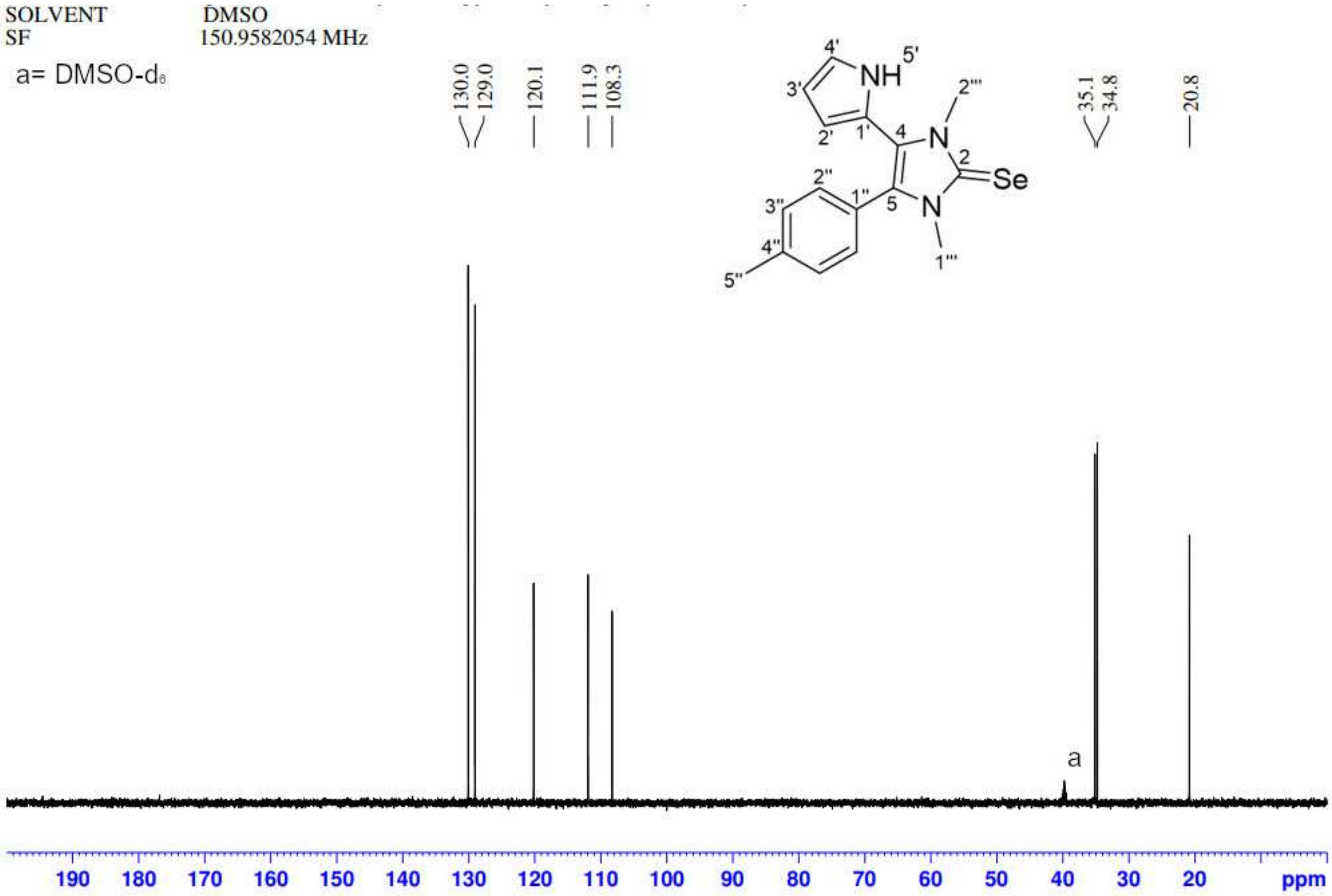

Figure S136. $^{13}C\{^1H\}$**-DEPT-NMR of compound 9c**

^{77}Se-NMR 1,3-Dimethyl-4-(1H-pyrrol-2-yl)-5-(p-tolyl)-1,3-dihydro-2H-imidazole-2-selenone (9c):

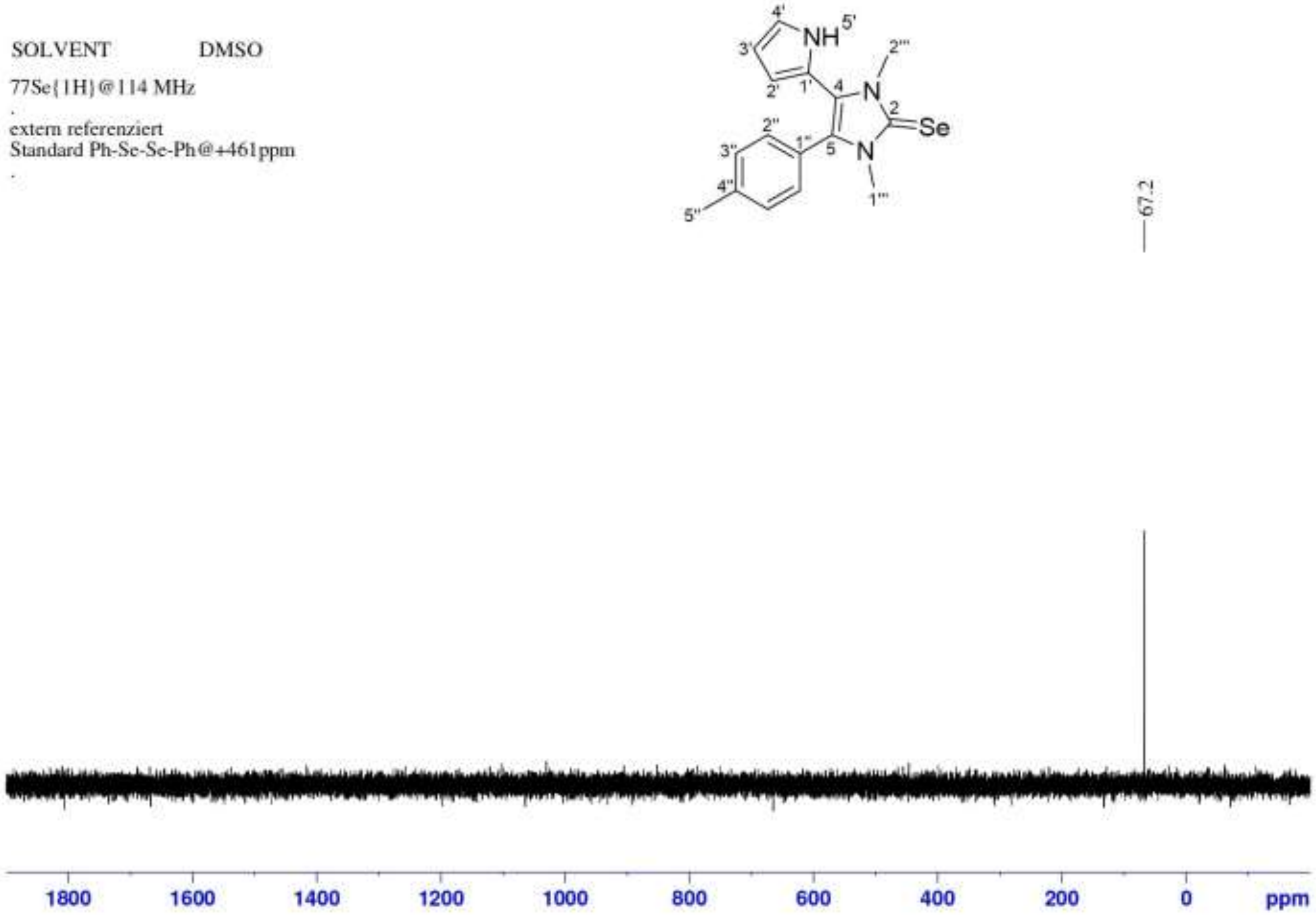

Figure S137. ^{77}Se-NMR of compound 9c

HSQC-NMR 1,3-Dimethyl-4-(1H-pyrrol-2-yl)-5-(p-tolyl)-1,3-dihydro-2H-imidazole-2-selenone (9c):

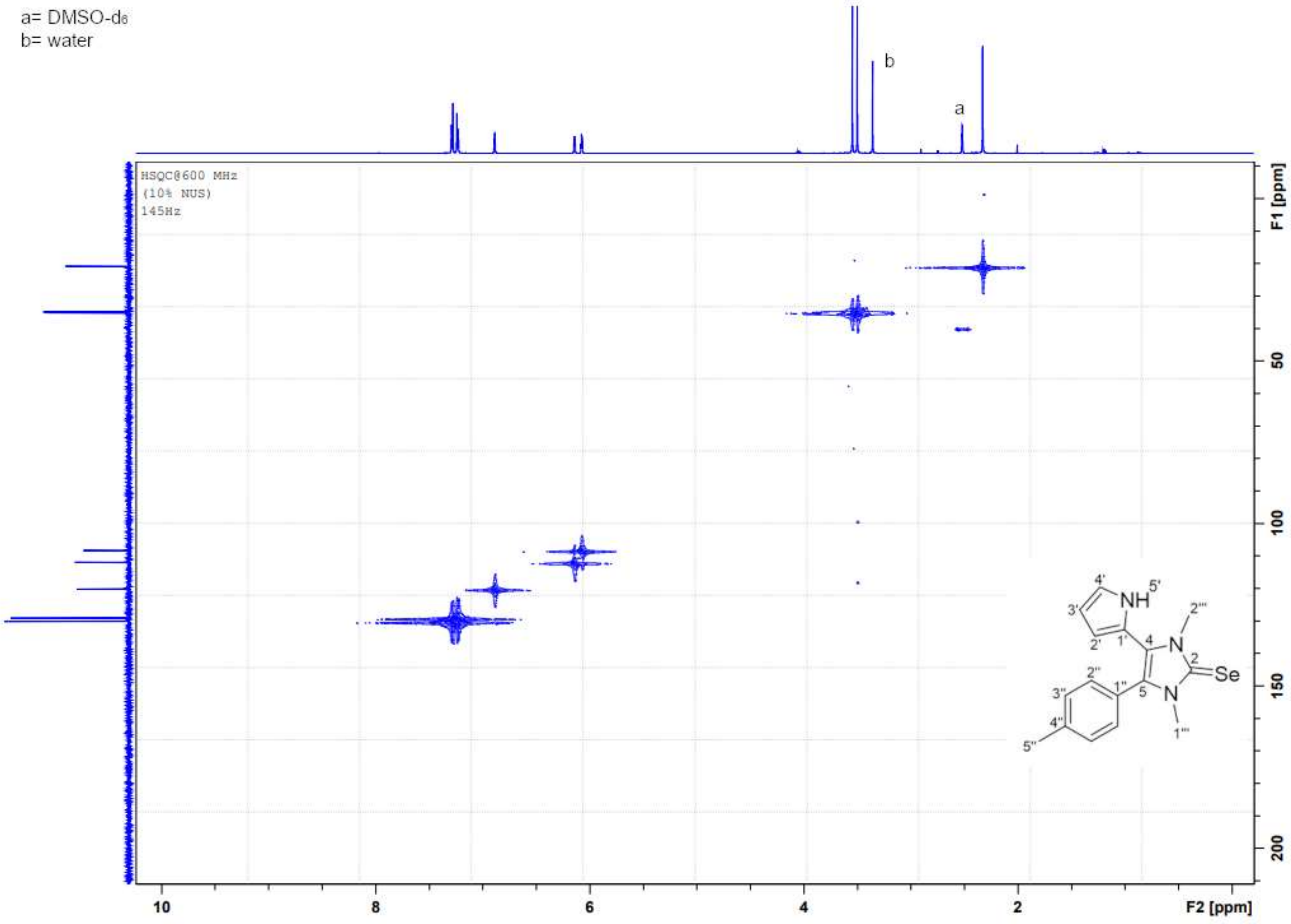

Figure S138. HSQC-NMR of compound 9c

HMBC-NMR 1,3-Dimethyl-4-(1H-pyrrol-2-yl)-5-(p-tolyl)-1,3-dihydro-2H-imidazole-2-selenone (9c):

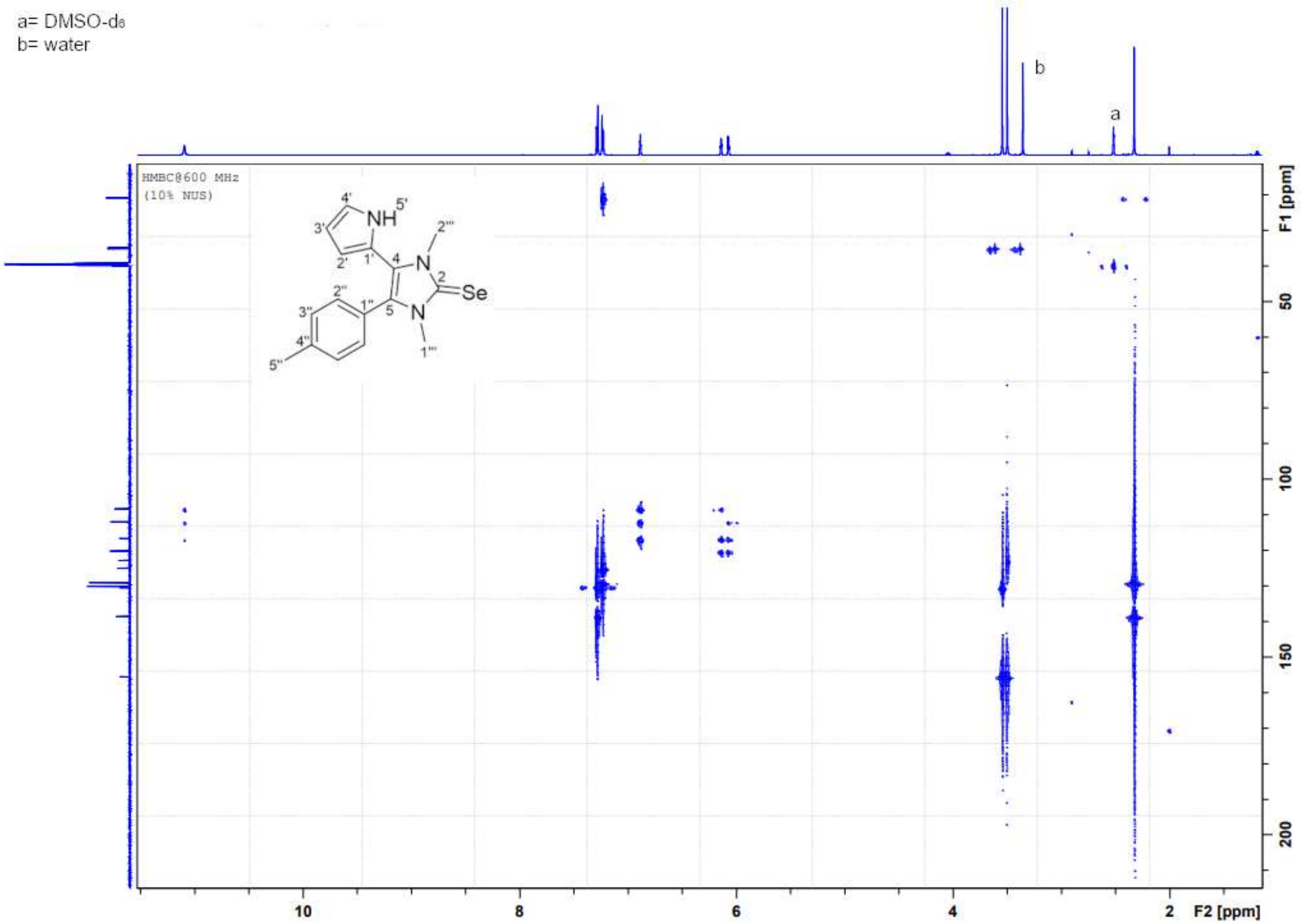

Figure S139. HMBC-NMR of compound 9c

^{1}H-NMR 5-(4-Methoxyphenyl)-1,3-dimethyl-4-(1H-pyrrol-2-yl)-1,3-dihydro-2H-imidazole-2-selenone (9d):

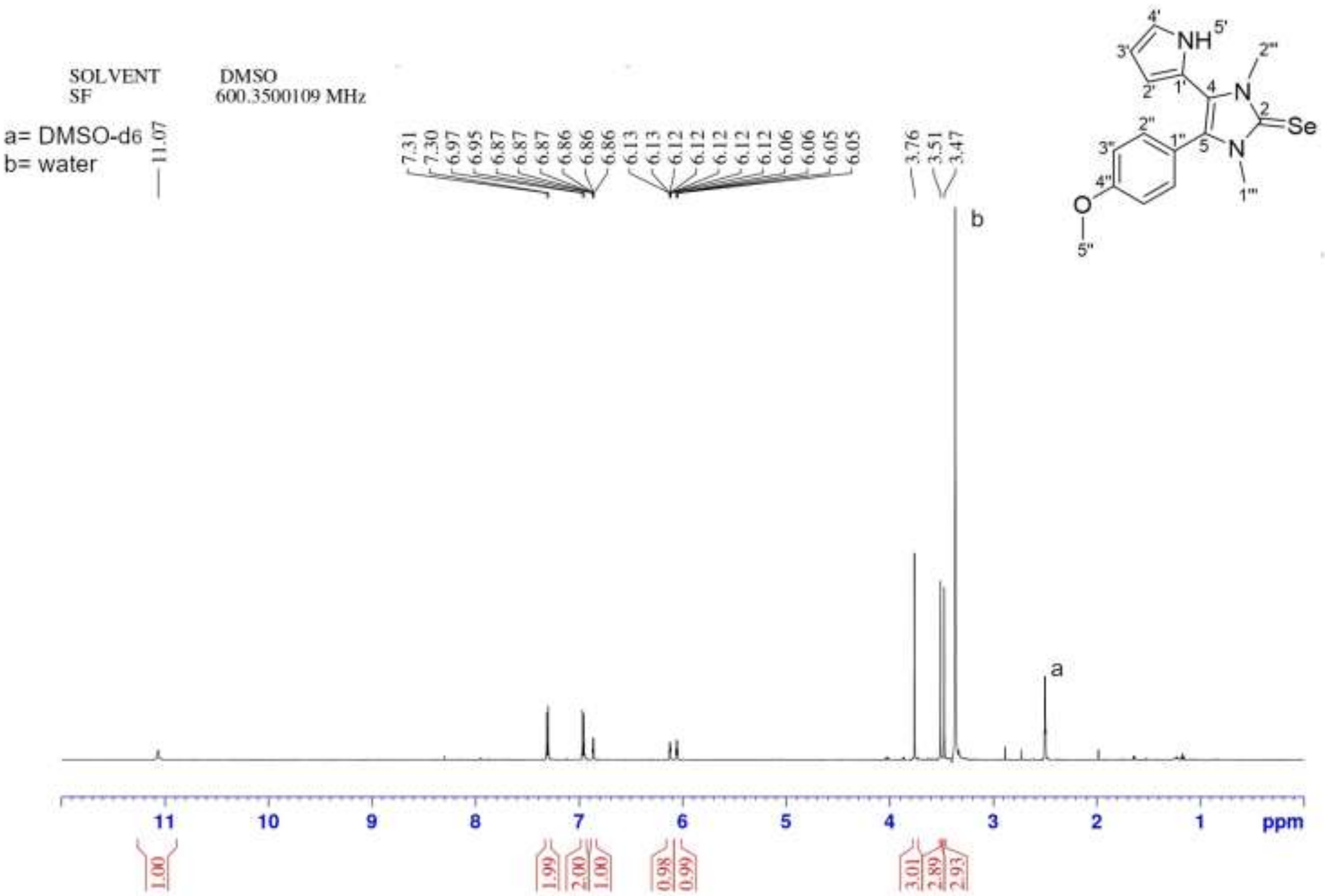

Figure S140. ^{1}H-NMR of compound 9d

$^{13}C\{^1H\}$-NMR 5-(4-Methoxyphenyl)-1,3-dimethyl-4-(1H-pyrrol-2-yl)-1,3-dihydro-2H-imidazole-2-selenone (9d):

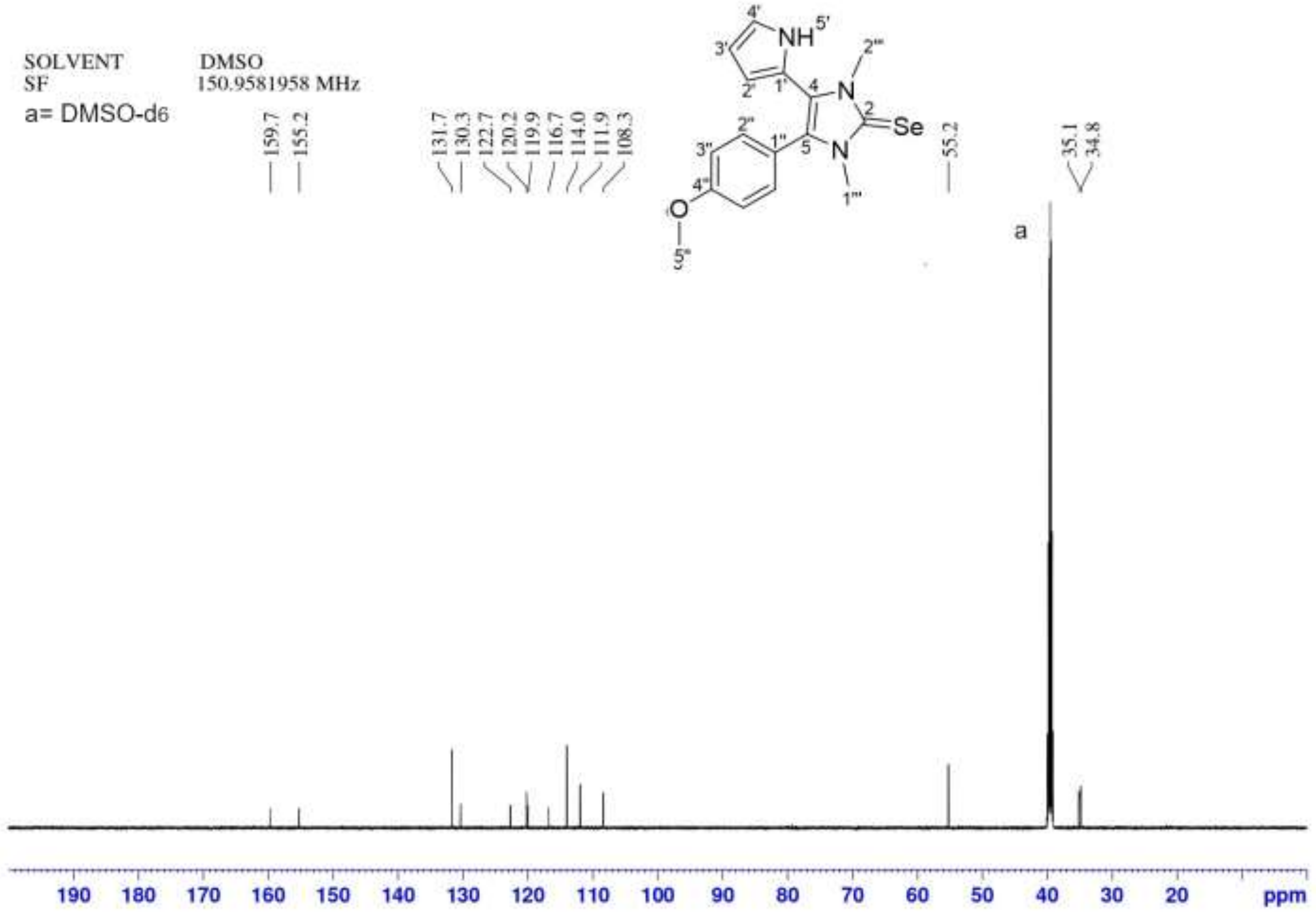

Figure S141. **$^{13}C\{^1H\}$-NMR of compound 9d**

$^{13}C\{^1H\}$-DEPT-NMR 5-(4-Methoxyphenyl)-1,3-dimethyl-4-(1H-pyrrol-2-yl)-1,3-dihydro-2H-imidazole-2-selenone (9d):

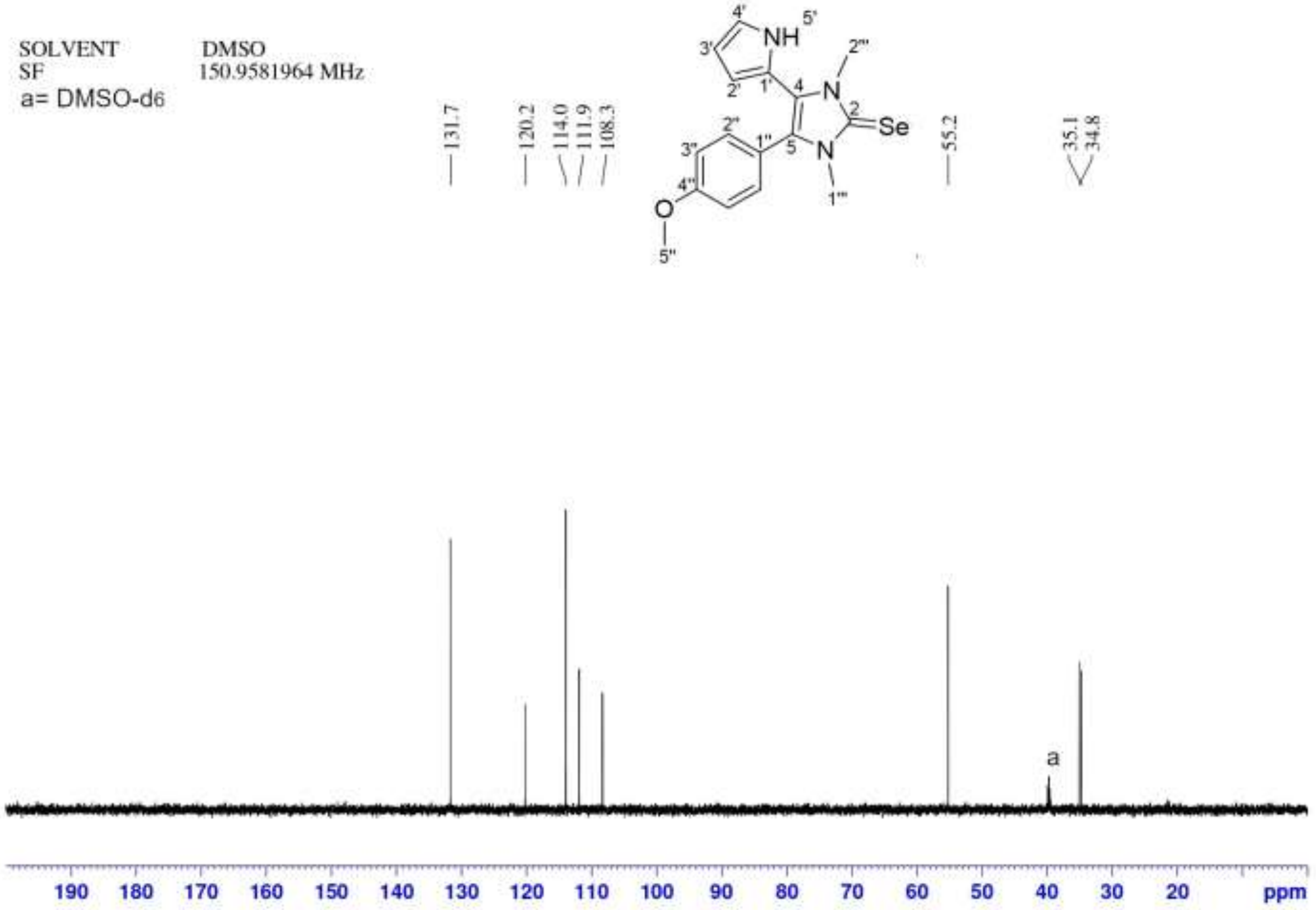

Figure S142. **$^{13}C\{^1H\}$-DEPT-NMR of compound 9d**

77Se-NMR 5-(4-Methoxyphenyl)-1,3-dimethyl-4-(1H-pyrrol-2-yl)-1,3-dihydro-2H-imidazole-2-selenone (9d):

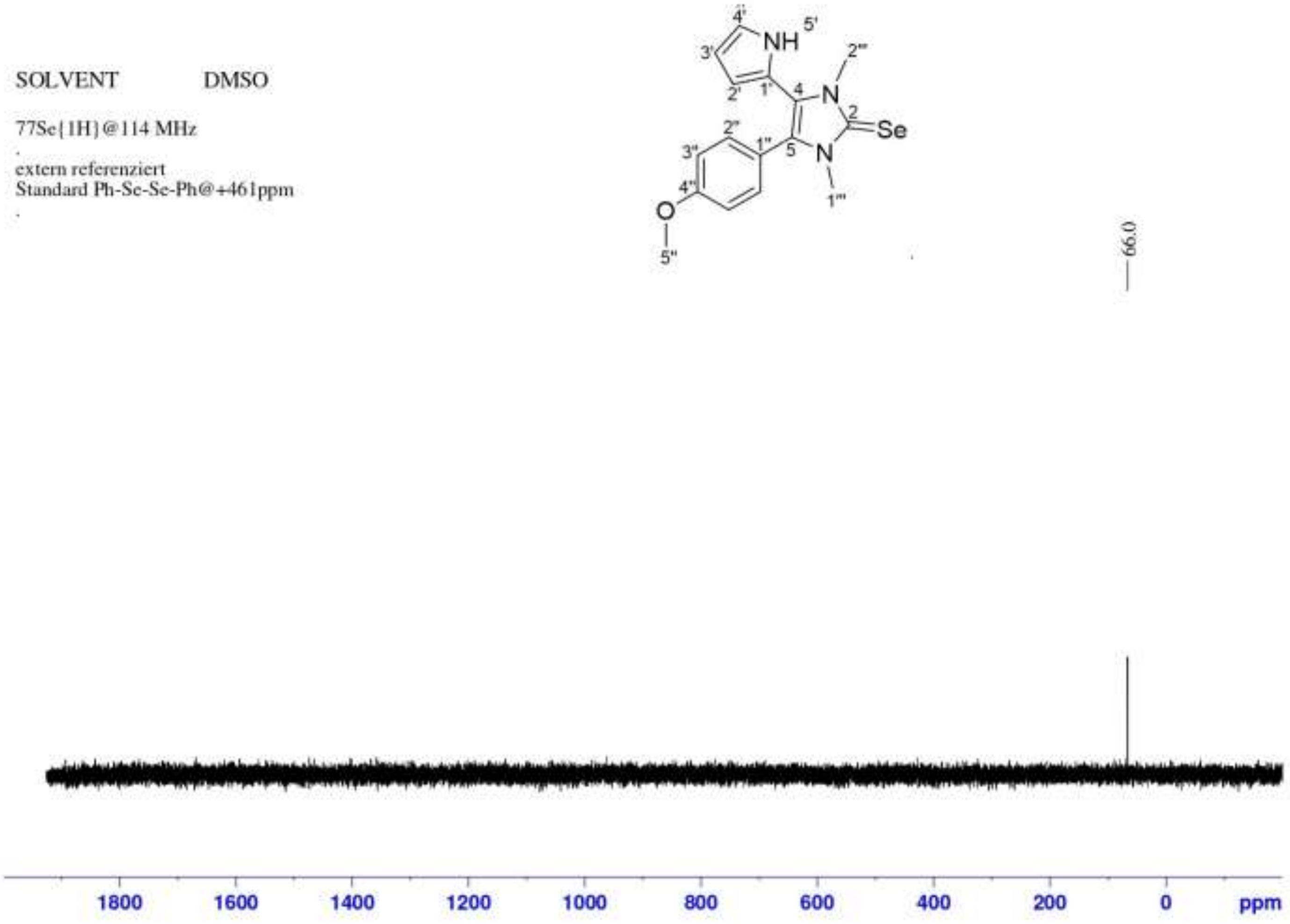

Figure S143. **77Se-NMR of compound 9d**

HSQC-NMR 5-(4-Methoxyphenyl)-1,3-dimethyl-4-(1H-pyrrol-2-yl)-1,3-dihydro-2H-imidazole-2-selenone (9d):

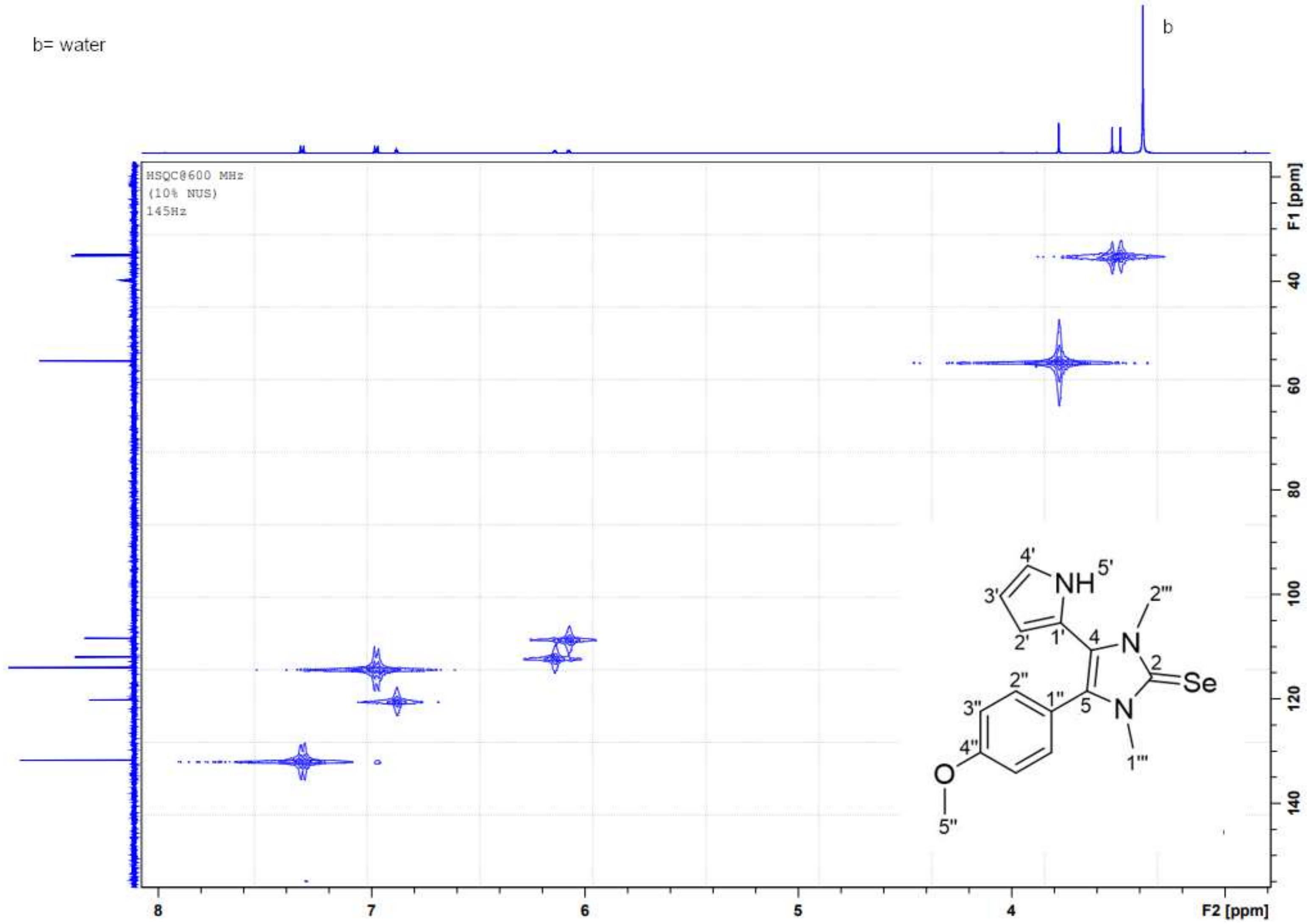

Figure S144. HSQC-NMR of compound 9d

HMBC-NMR 5-(4-Methoxyphenyl)-1,3-dimethyl-4-(1H-pyrrol-2-yl)-1,3-dihydro-2H-imidazole-2-selenone (9d):

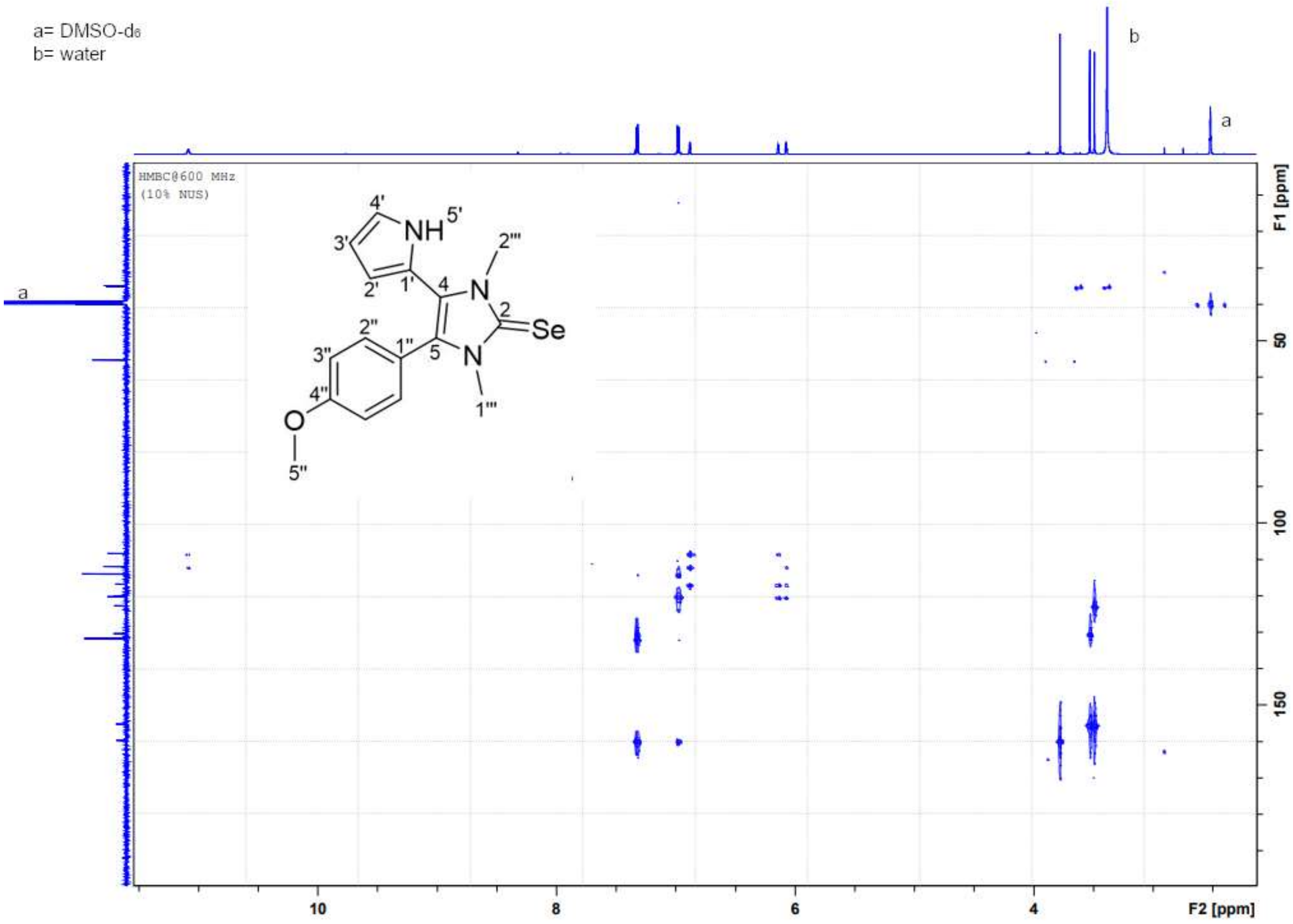

Figure S145. HMBC-NMR of compound 9d

^{1}H-NMR 1-Benzyl-3-methyl-4-(1H-pyrrol-2-yl)-1,3-dihydro-2H-imidazole-2-selenone (9e):

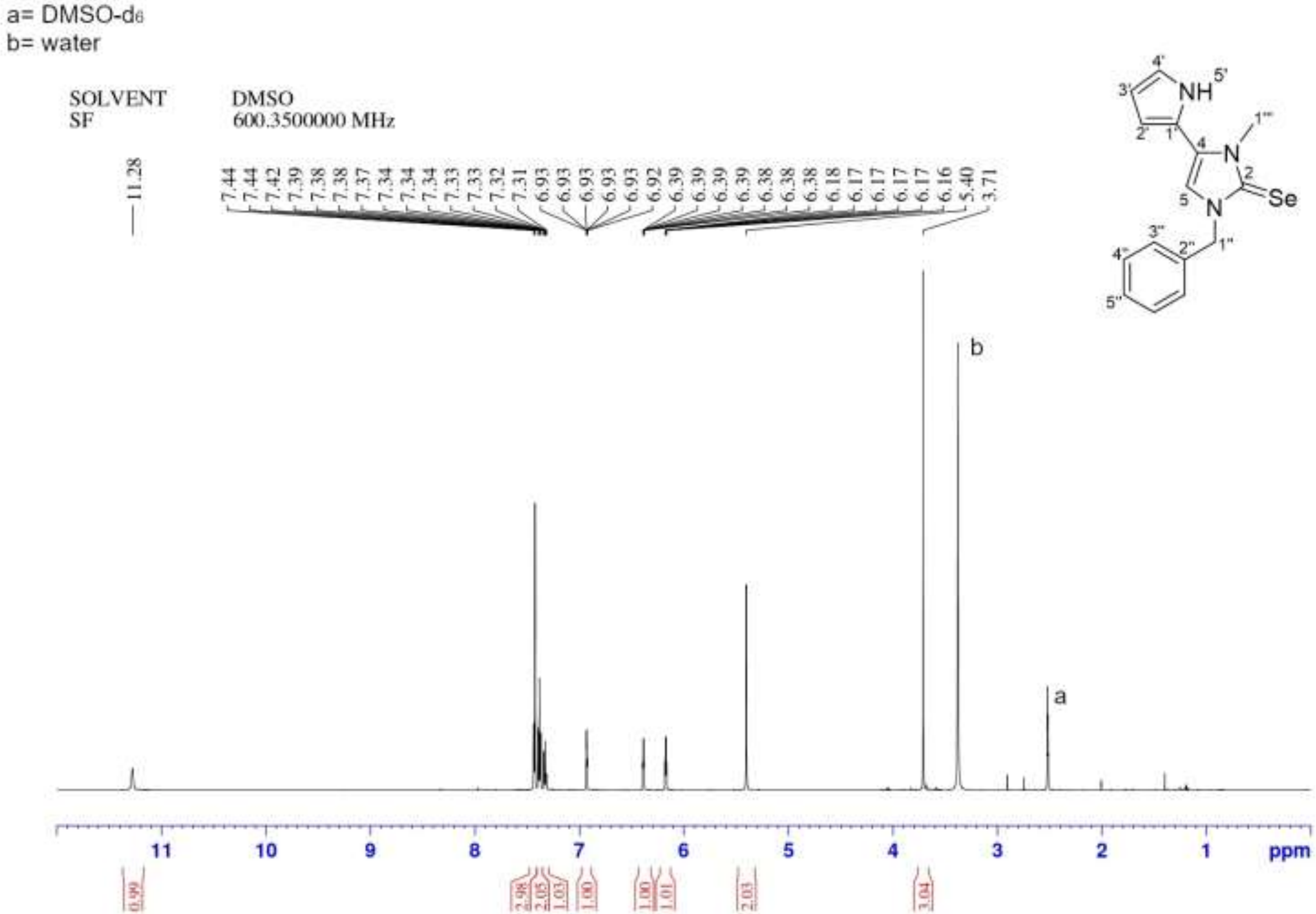

Figure S146. ^{1}H-NMR of compound 9e

$^{13}C\{^1H\}$-NMR 1-Benzyl-3-methyl-4-(1H-pyrrol-2-yl)-1,3-dihydro-2H-imidazole-2-selenone (9e):

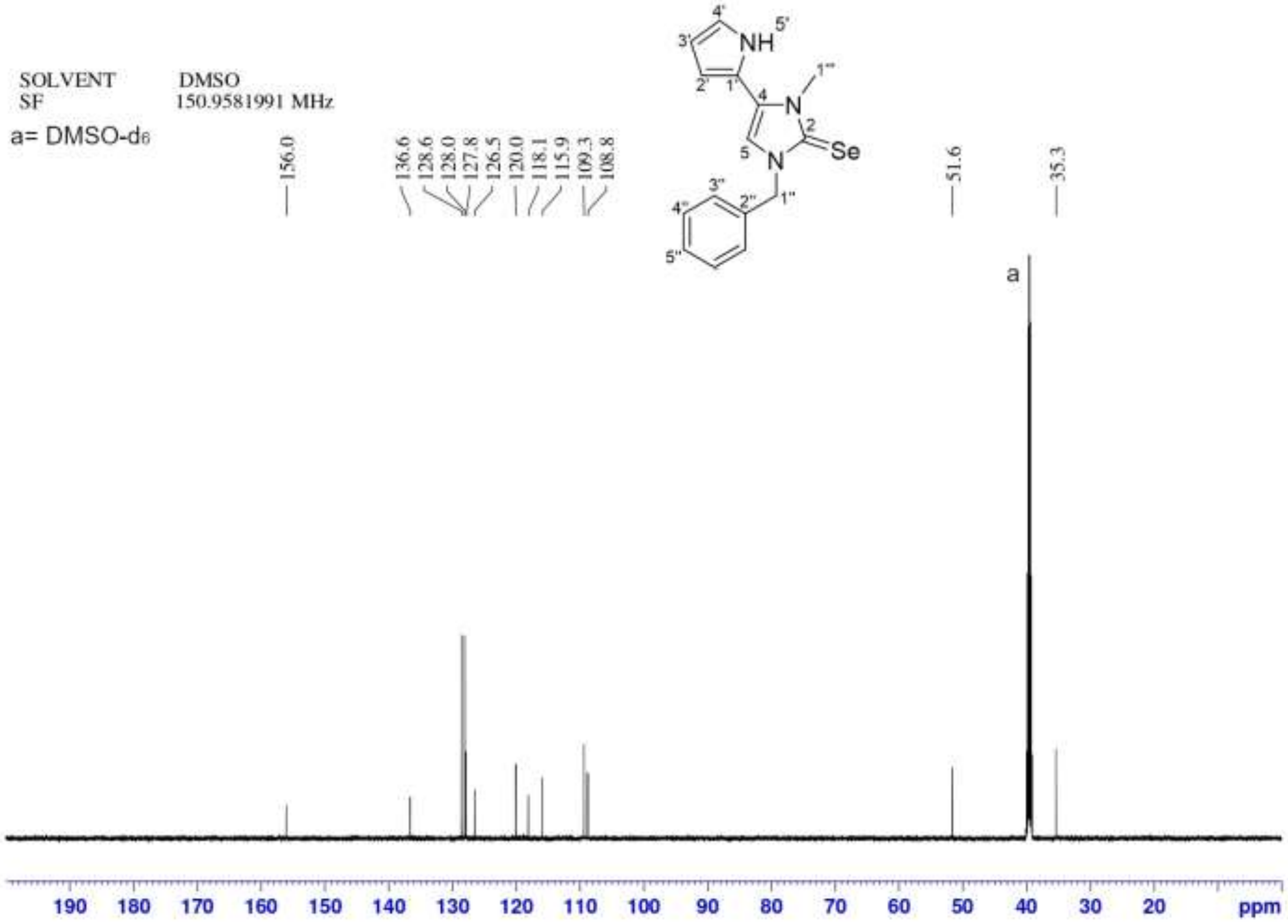

Figure S147. $^{13}C\{^1H\}$**-NMR of compound 9e**

$^{13}C\{^1H\}$-DEPT-NMR 1-Benzyl-3-methyl-4-(1H-pyrrol-2-yl)-1,3-dihydro-2H-imidazole-2-selenone (9e):

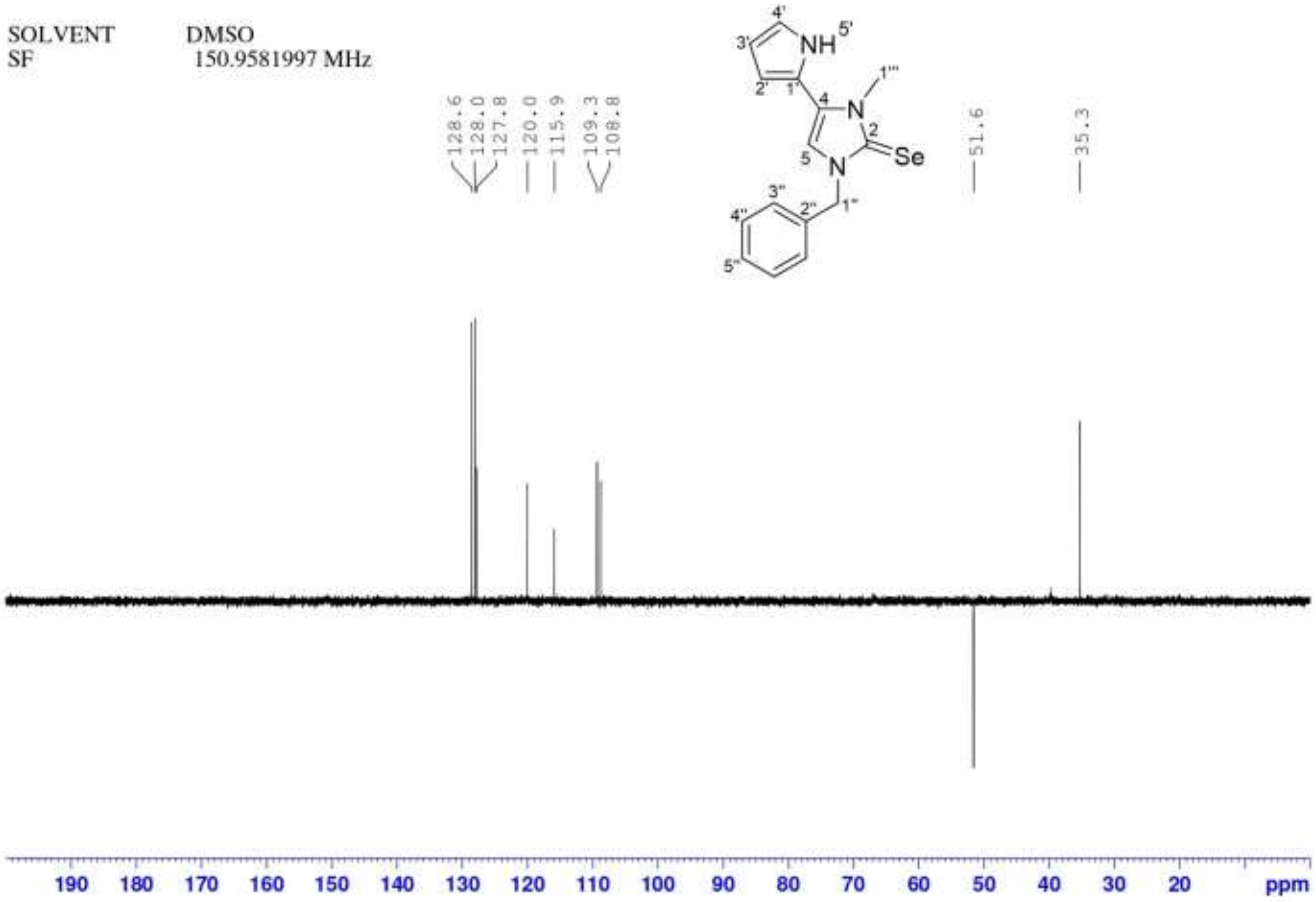

Figure S148. **$^{13}C\{^1H\}$-DEPT-NMR of compound 9e**

^{77}Se-NMR 1-Benzyl-3-methyl-4-(1H-pyrrol-2-yl)-1,3-dihydro-2H-imidazole-2-selenone (9e):

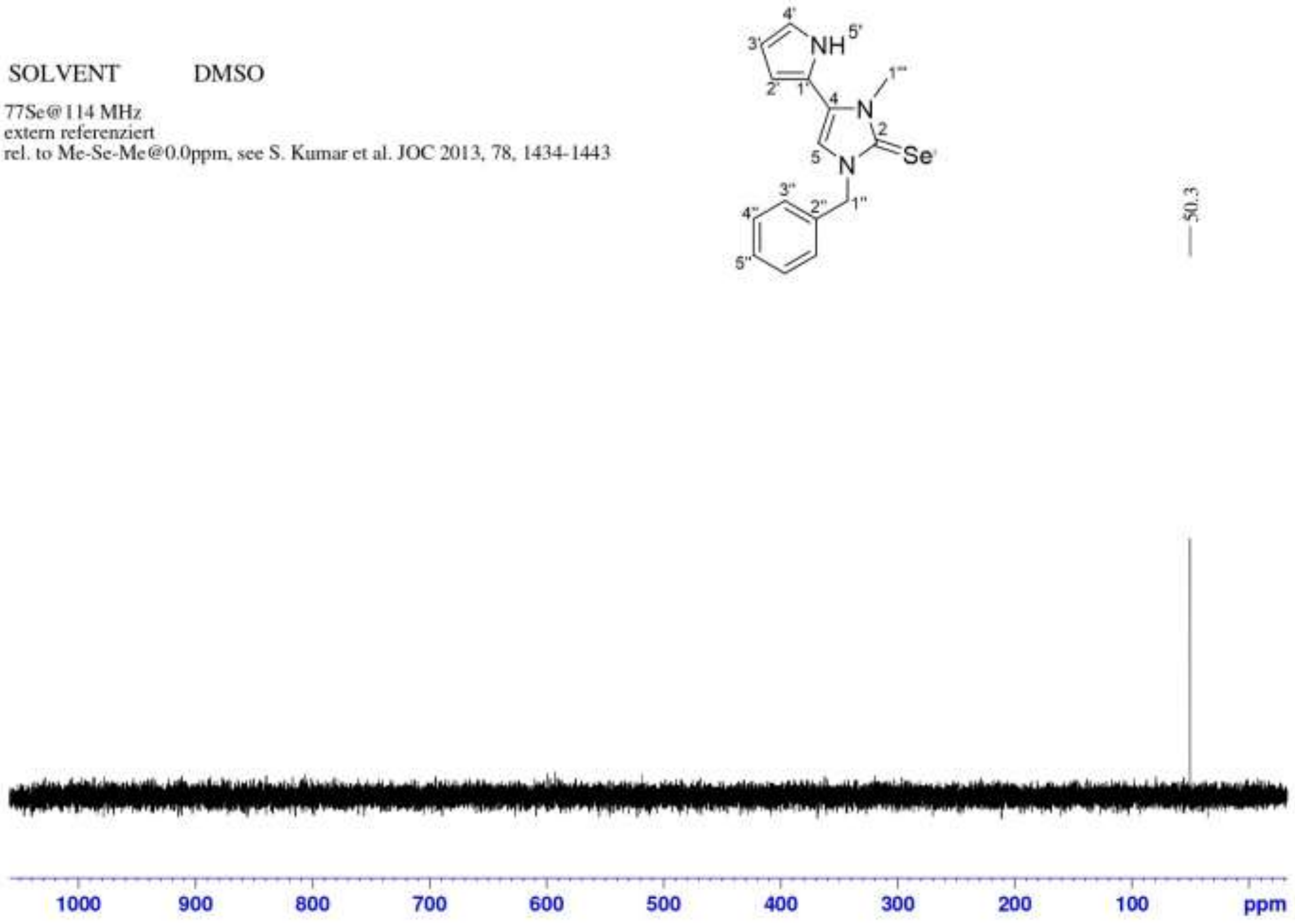

Figure S149. **^{77}Se-NMR of compound 9e**

HSQC-NMR 1-Benzyl-3-methyl-4-(1H-pyrrol-2-yl)-1,3-dihydro-2H-imidazole-2-selenone (9e):

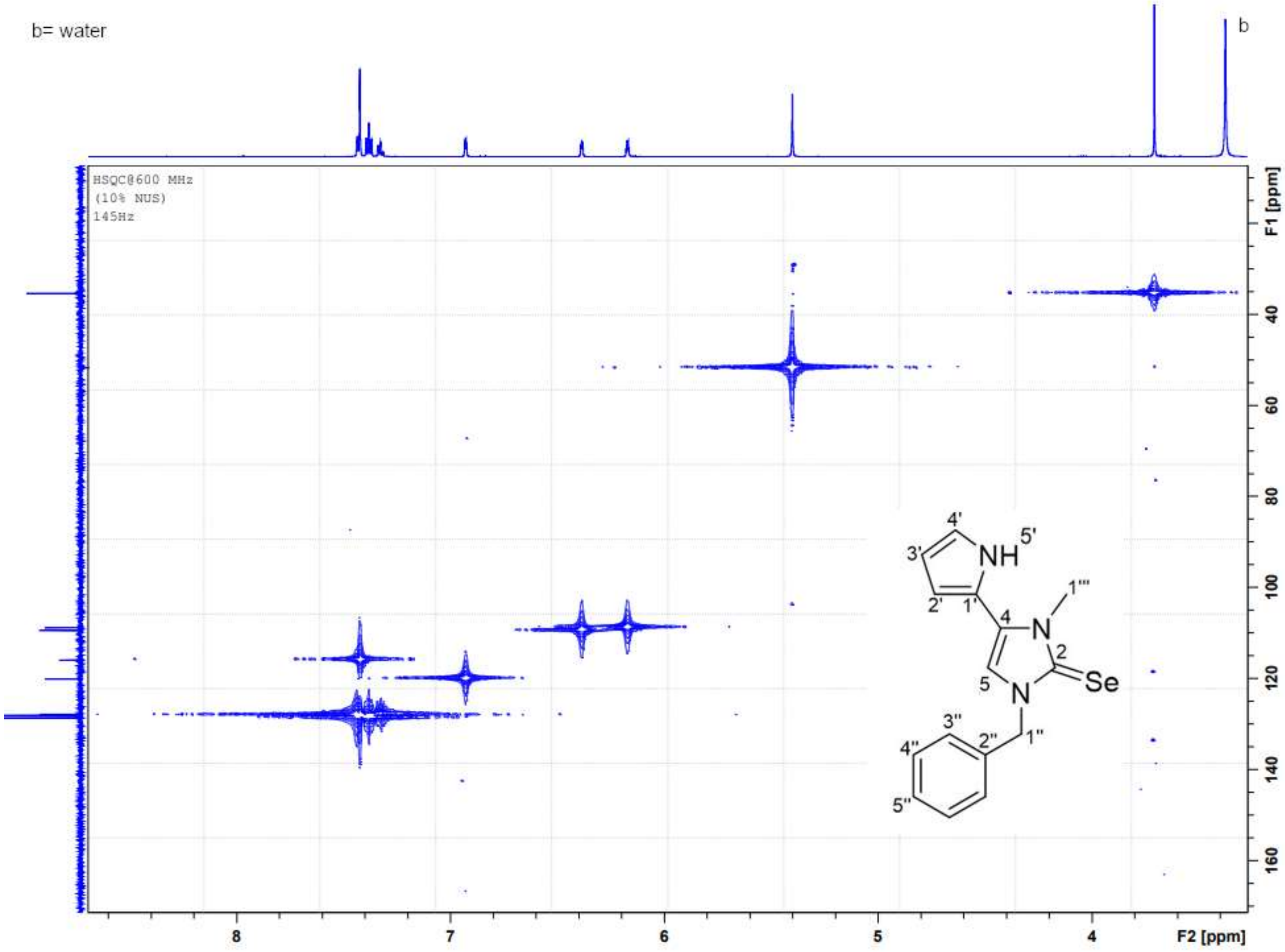

Figure S150. HSQC-NMR of compound 9e

HMBC-NMR 1-Benzyl-3-methyl-4-(1H-pyrrol-2-yl)-1,3-dihydro-2H-imidazole-2-selenone (9e):

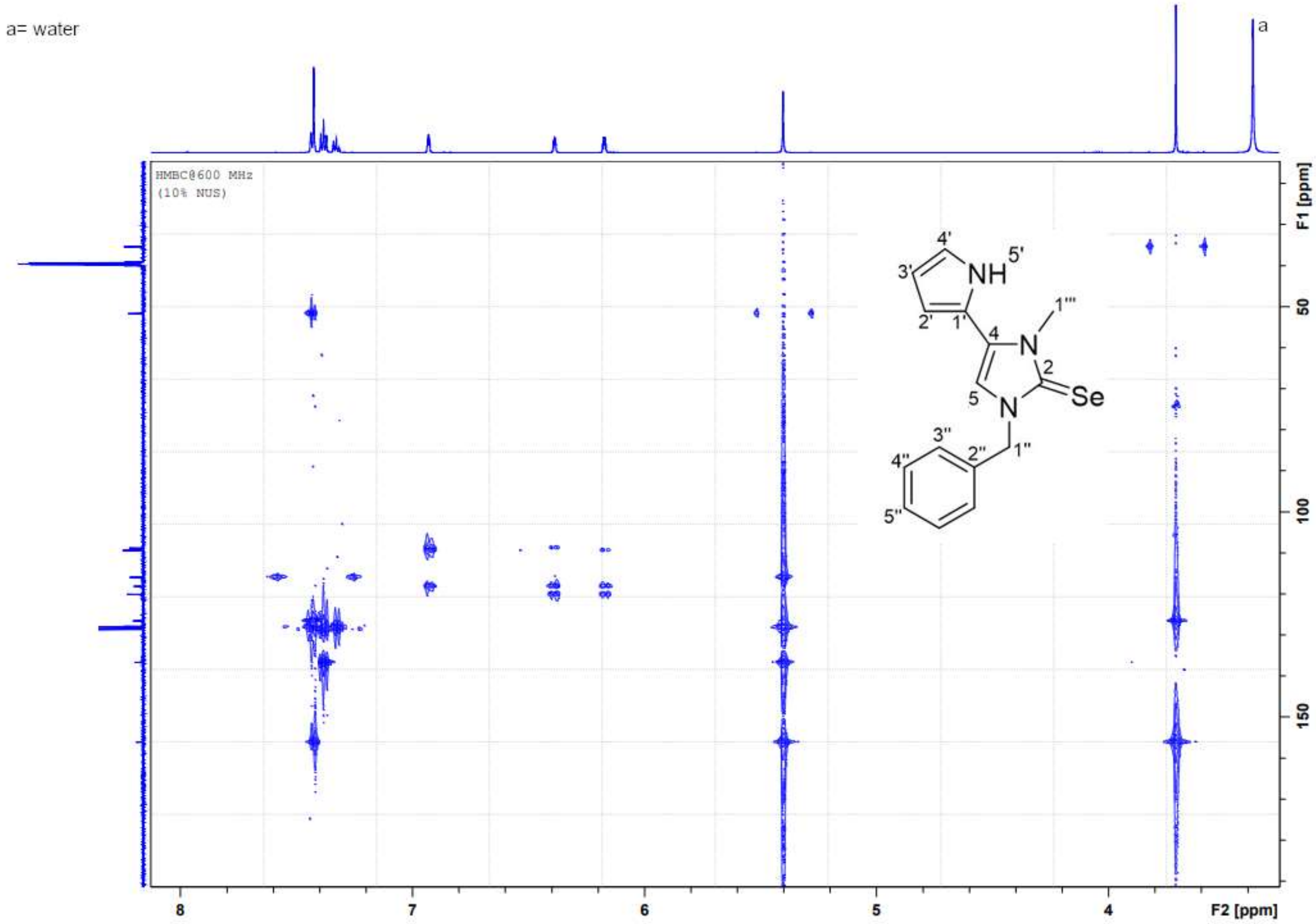

Figure S151. HMBC-NMR of compound 9e

^{1}H-NMR 3-Methyl-1-(4-methylbenzyl)-4-(1H-pyrrol-2-yl)-1,3-dihydro-2H-imidazole-2-selenone (9f):

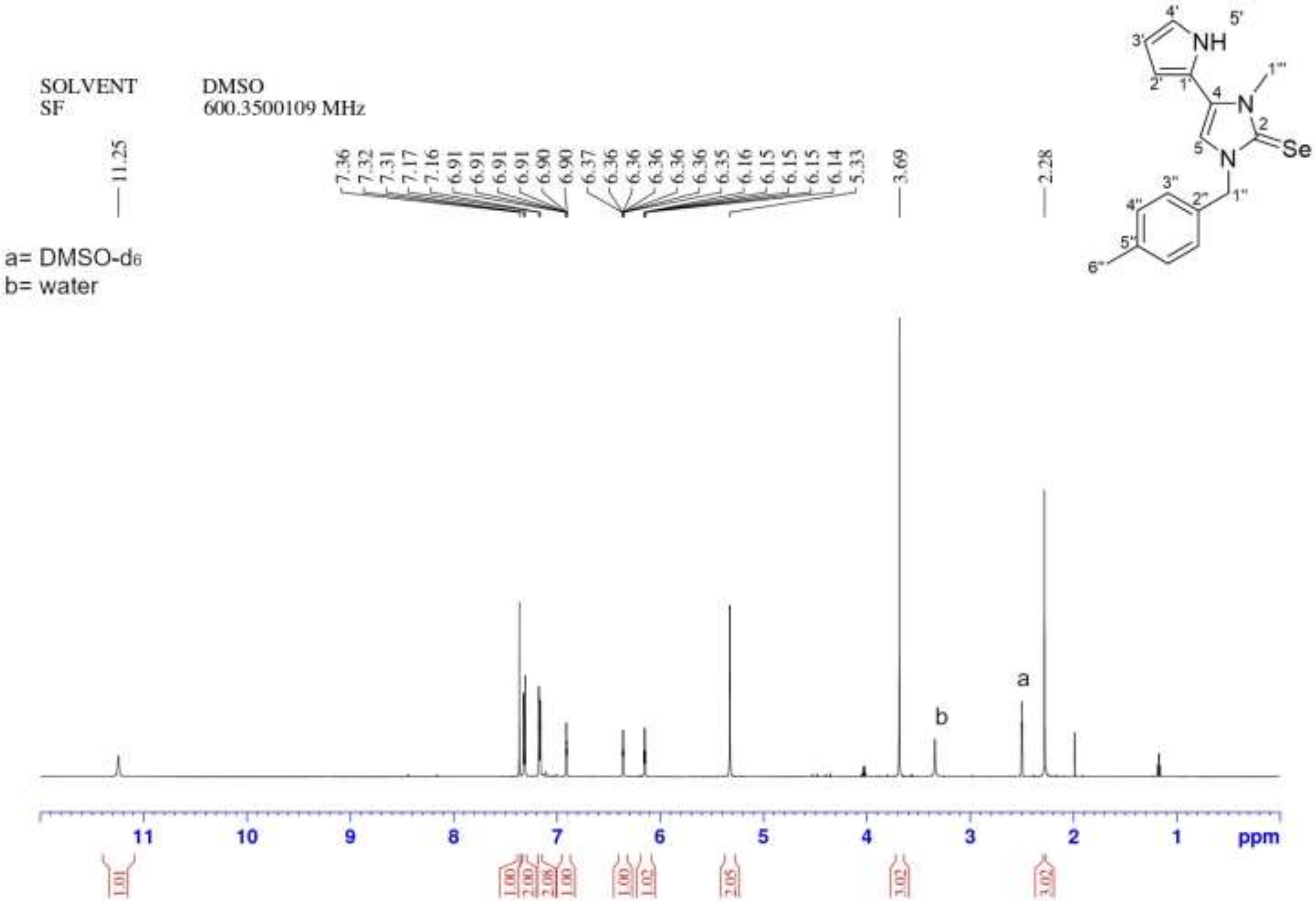

Figure S152. ^{1}H-NMR of compound 9f

$^{13}C\{^1H\}$-NMR 3-Methyl-1-(4-methylbenzyl)-4-(1H-pyrrol-2-yl)-1,3-dihydro-2H-imidazole-2-selenone (9f):

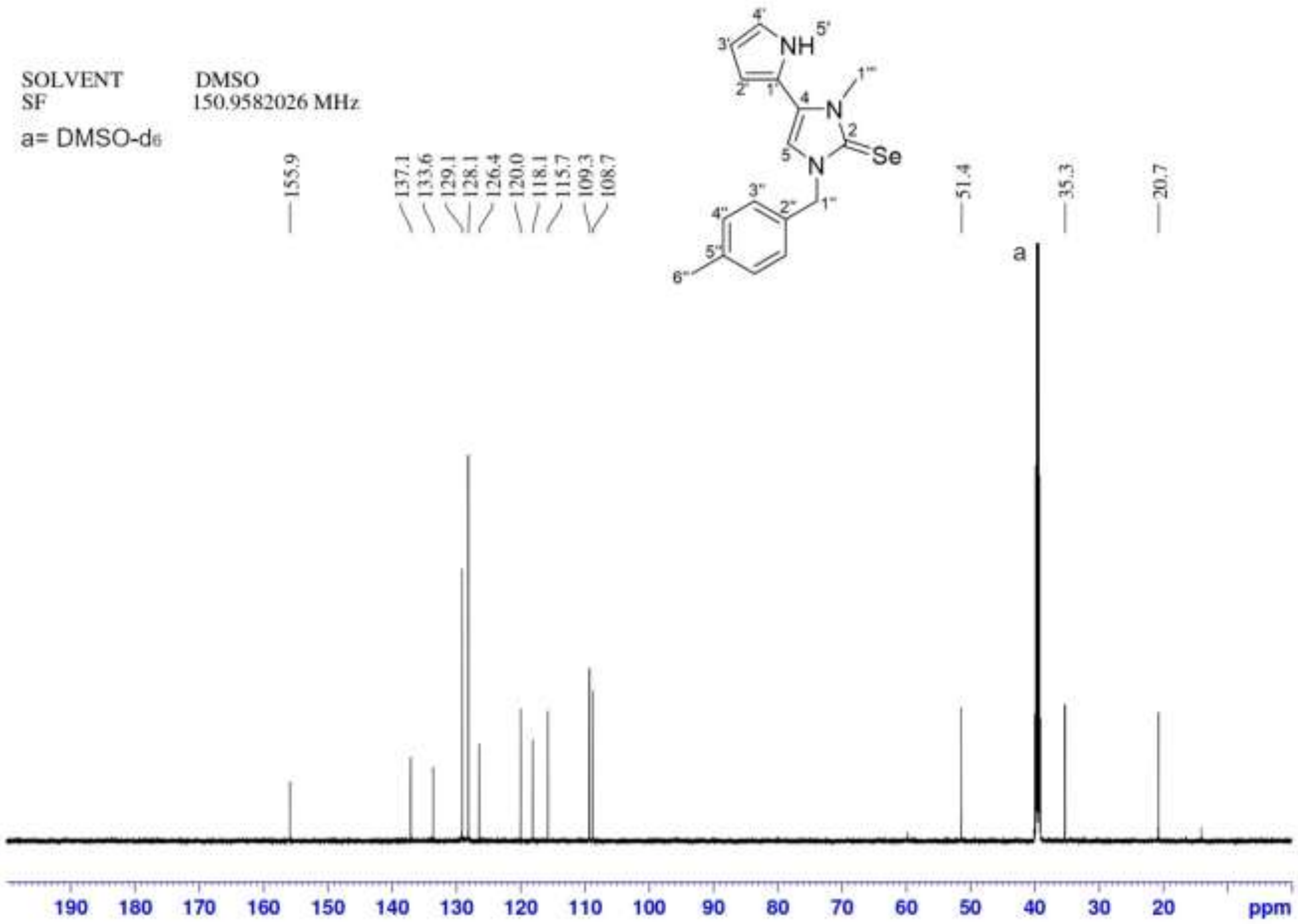

Figure S153. $^{13}C\{^1H\}$-NMR of compound 9f

$^{13}C\{^1H\}$**-DEPT-NMR 3-Methyl-1-(4-methylbenzyl)-4-(1H-pyrrol-2-yl)-1,3-dihydro-2H-imidazole-2-selenone (9f):**

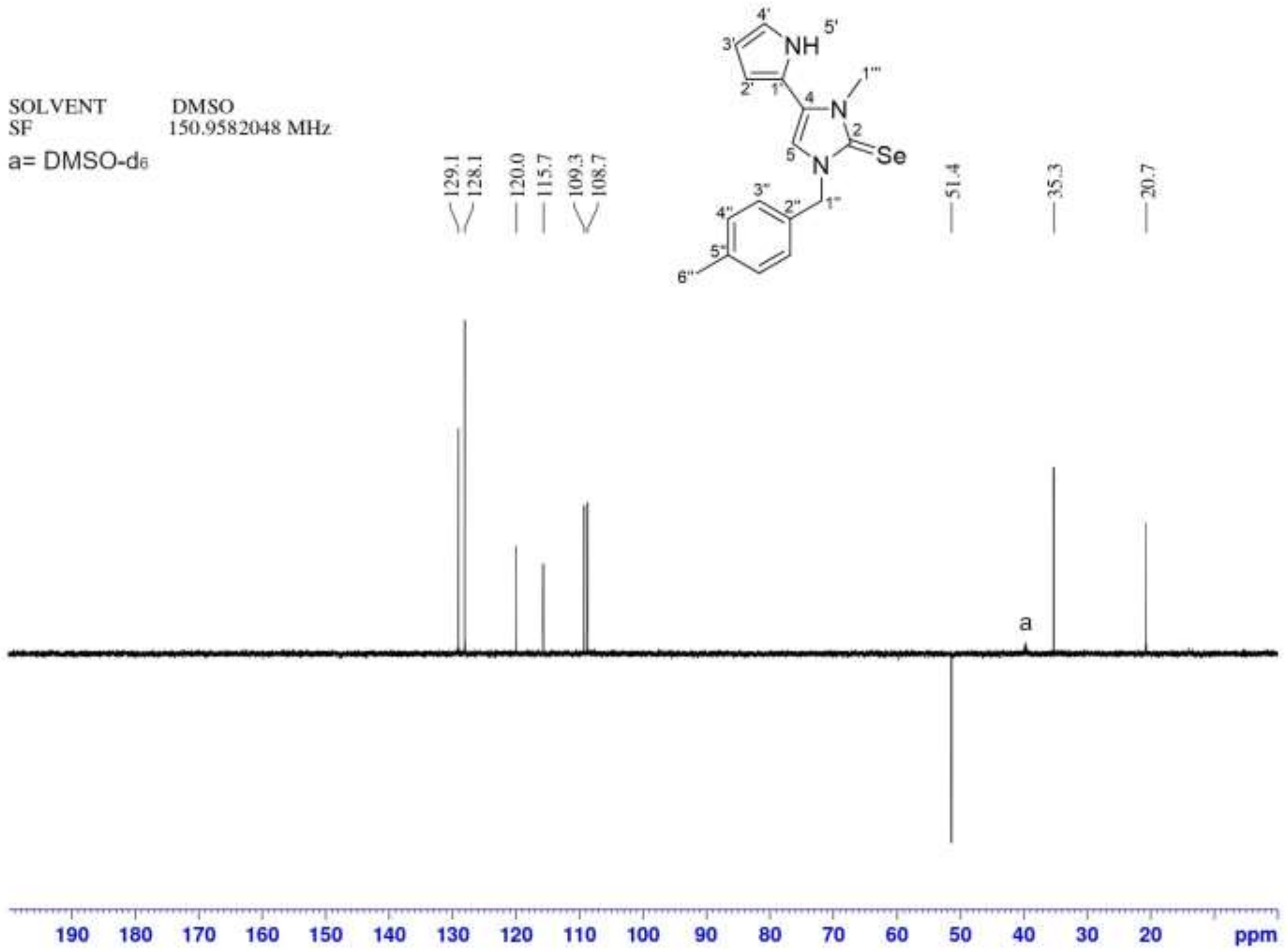

Figure S154. $^{13}C\{^1H\}$**-DEPT-NMR of compound 9f**

^{77}Se-NMR 3-Methyl-1-(4-methylbenzyl)-4-(1H-pyrrol-2-yl)-1,3-dihydro-2H-imidazole-2-selenone (9f):

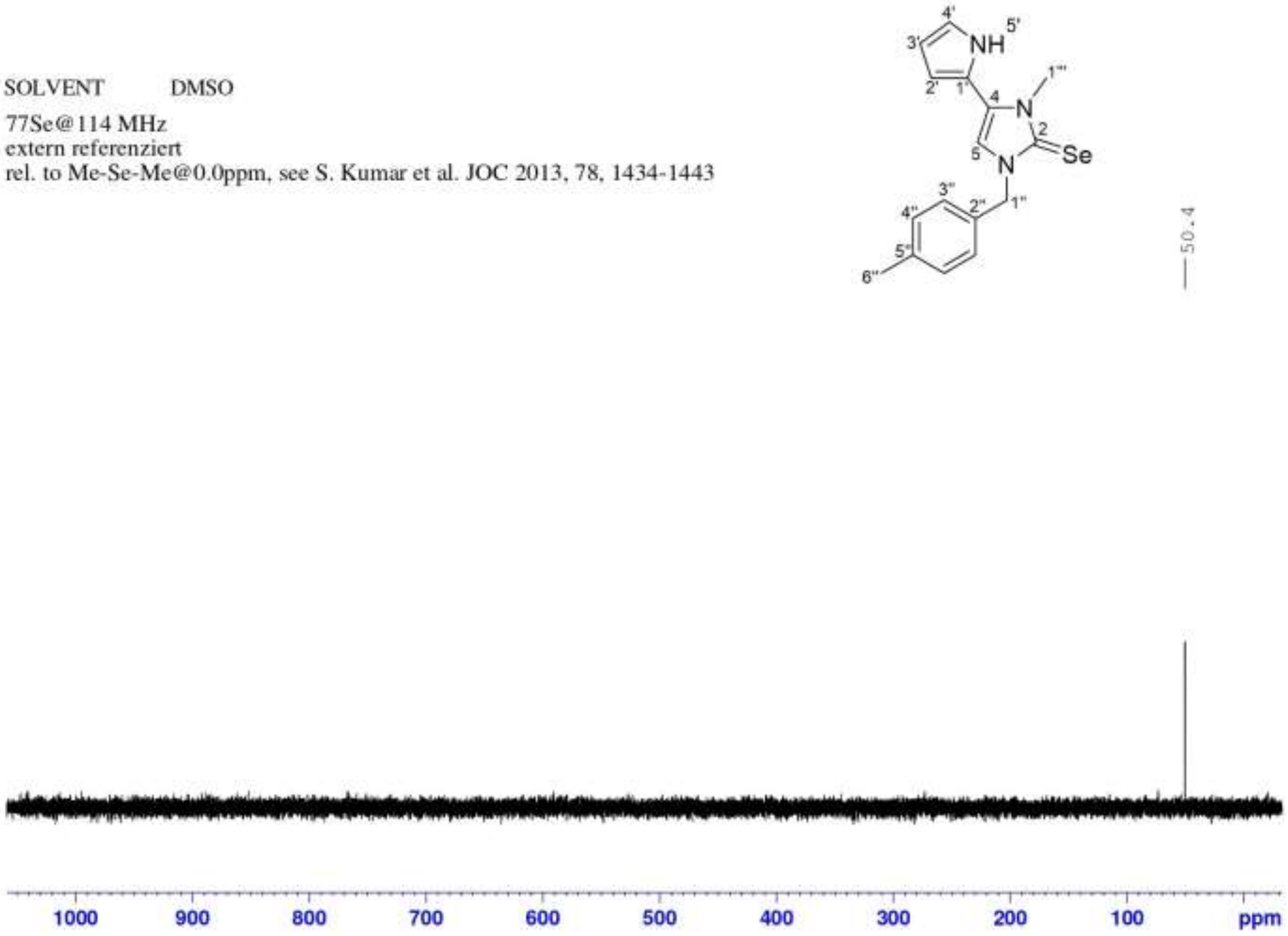

Figure S155. ^{77}Se-NMR of compound 9f

HSQC-NMR 3-Methyl-1-(4-methylbenzyl)-4-(1H-pyrrol-2-yl)-1,3-dihydro-2H-imidazole-2-selenone (9f):

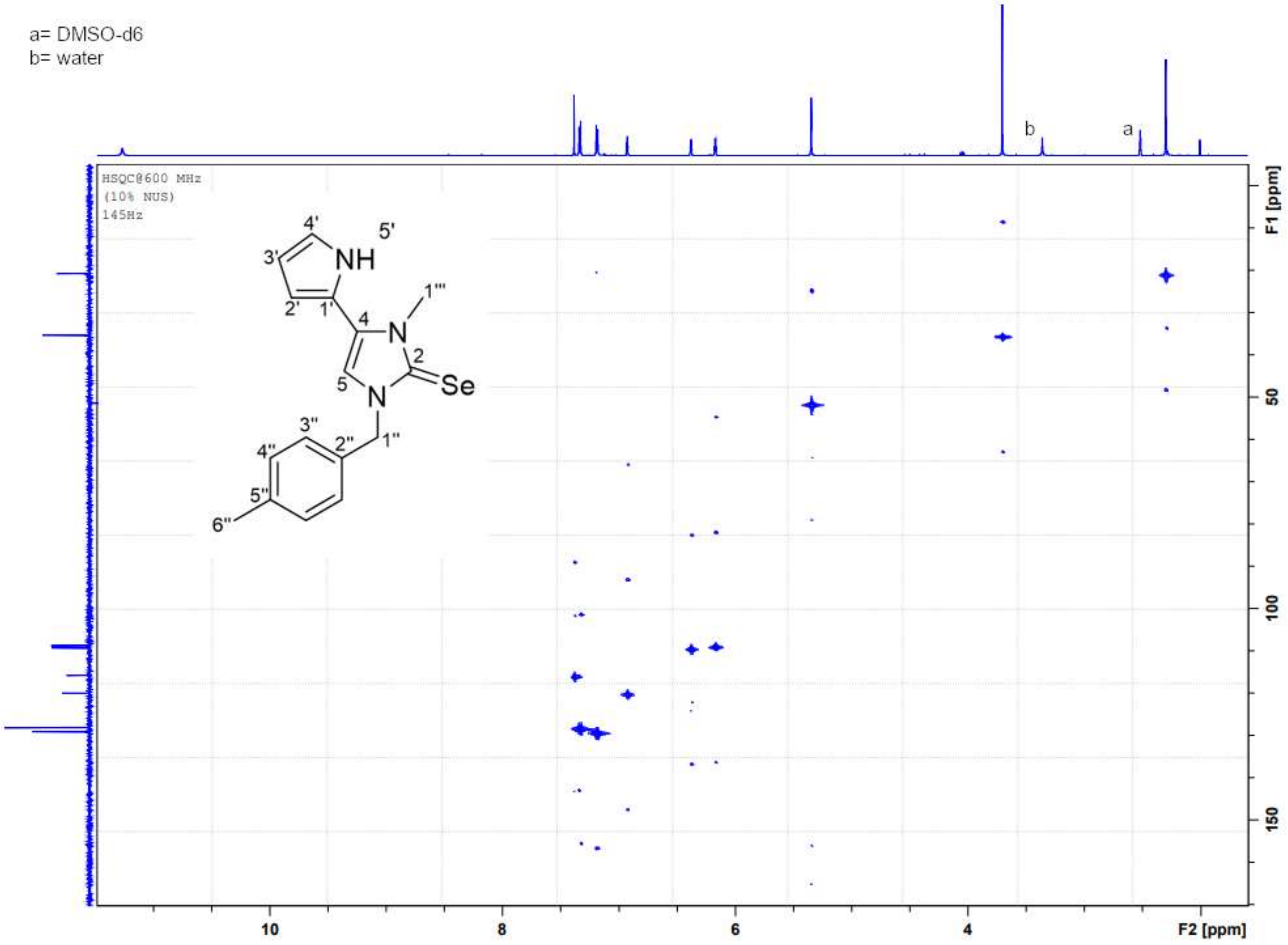

Figure 156 HSQC-NMR of compound 9f

HMBC-NMR 3-Methyl-1-(4-methylbenzyl)-4-(1H-pyrrol-2-yl)-1,3-dihydro-2H-imidazole-2-selenone (9f):

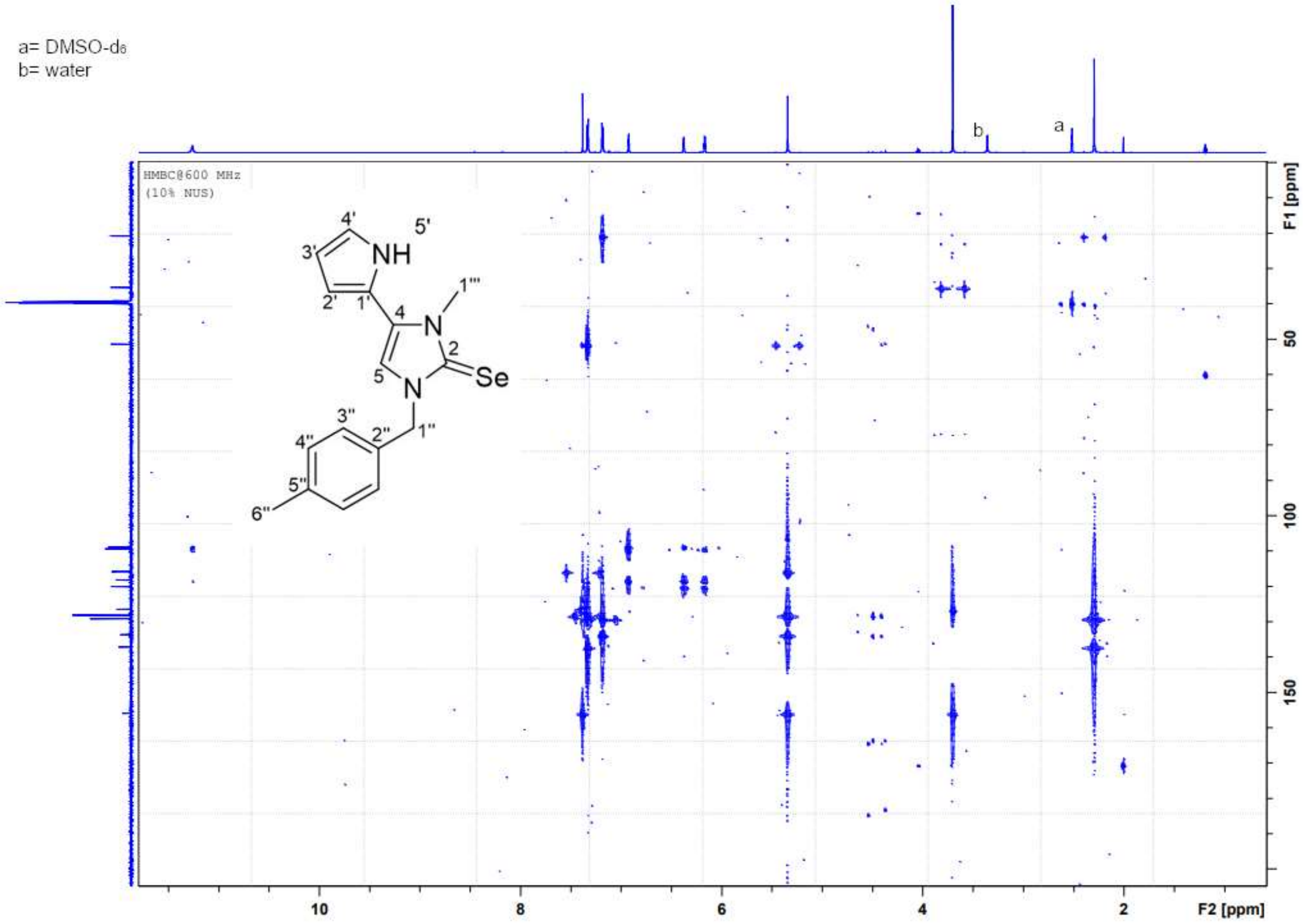

Figure S157. HMBC-NMR of compound 9f

^{1}H-NMR 1-(4-Methoxybenzyl)-3-methyl-4-(1H-pyrrol-2-yl)-1,3-dihydro-2H-imidazole-2-selenone (9g):

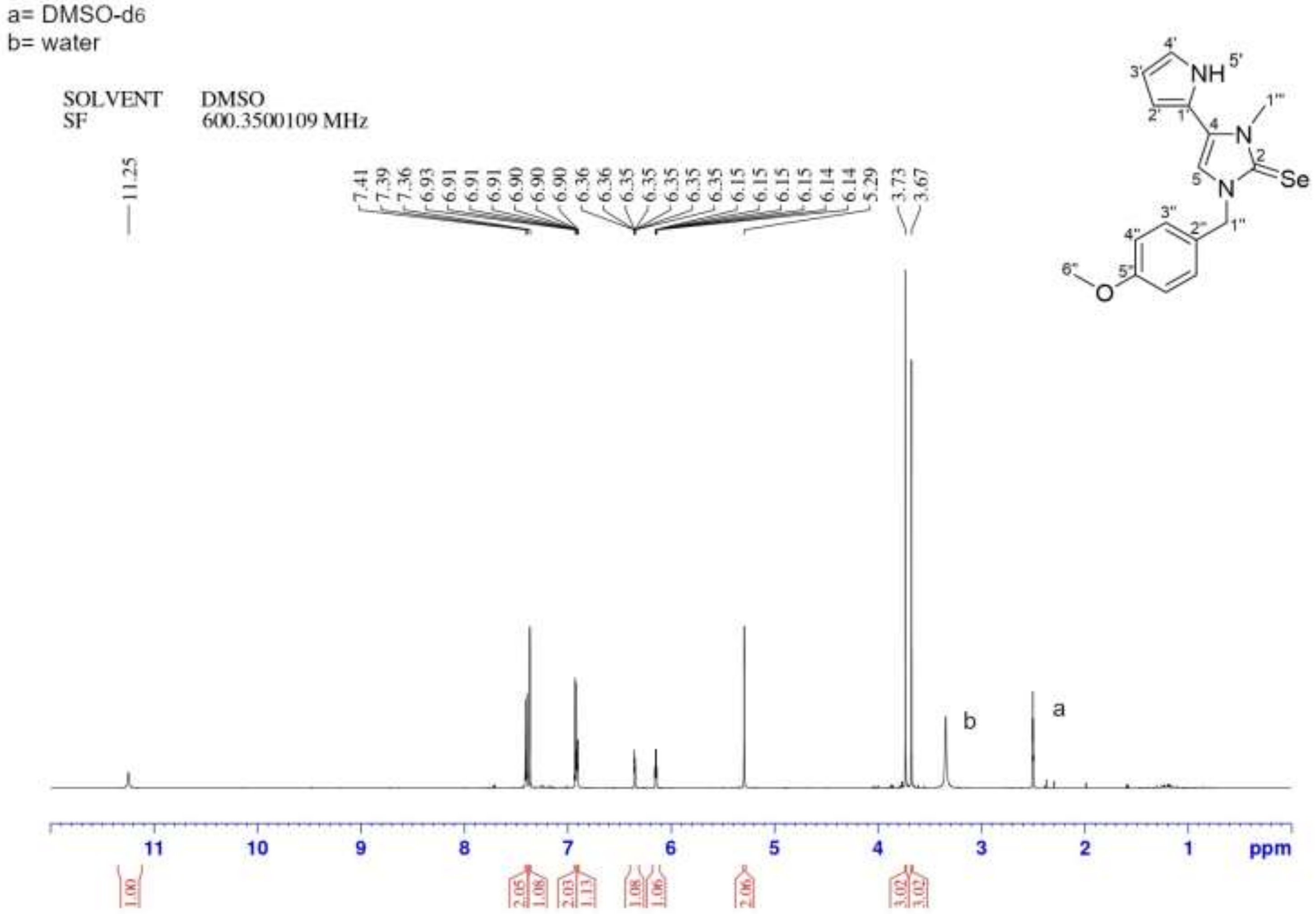

Figure S158. ^{1}H-NMR of compound 9g

$^{13}C\{^1H\}$-NMR 1-(4-Methoxybenzyl)-3-methyl-4-(1H-pyrrol-2-yl)-1,3-dihydro-2H-imidazole-2-selenone (9g):

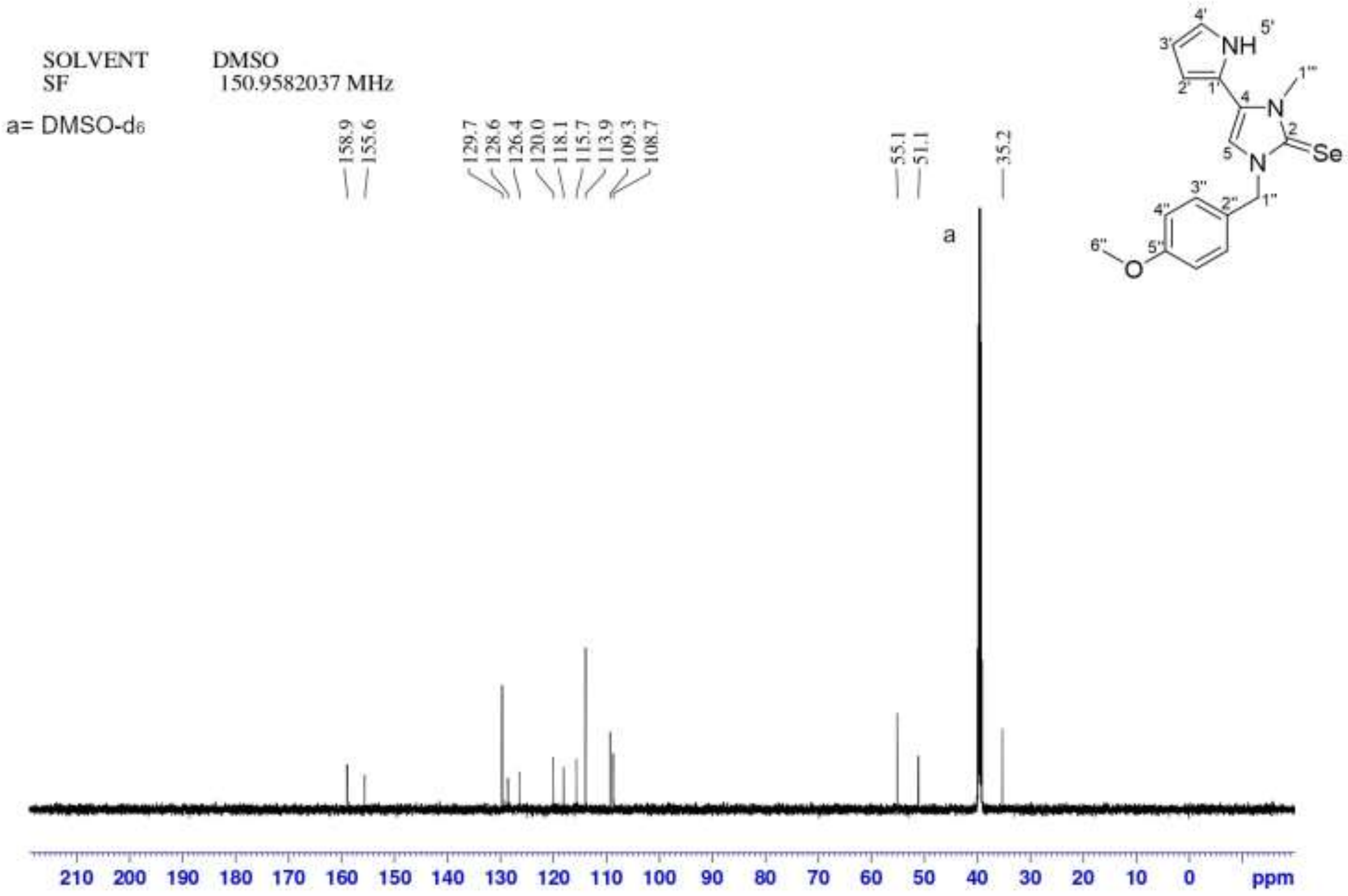

Figure S159. $^{13}C\{^1H\}$**-NMR of compound 9g**

$^{13}C\{^1H\}$-DEPT-NMR 1-(4-Methoxybenzyl)-3-methyl-4-(1H-pyrrol-2-yl)-1,3-dihydro-2H-imidazole-2-selenone (9g):

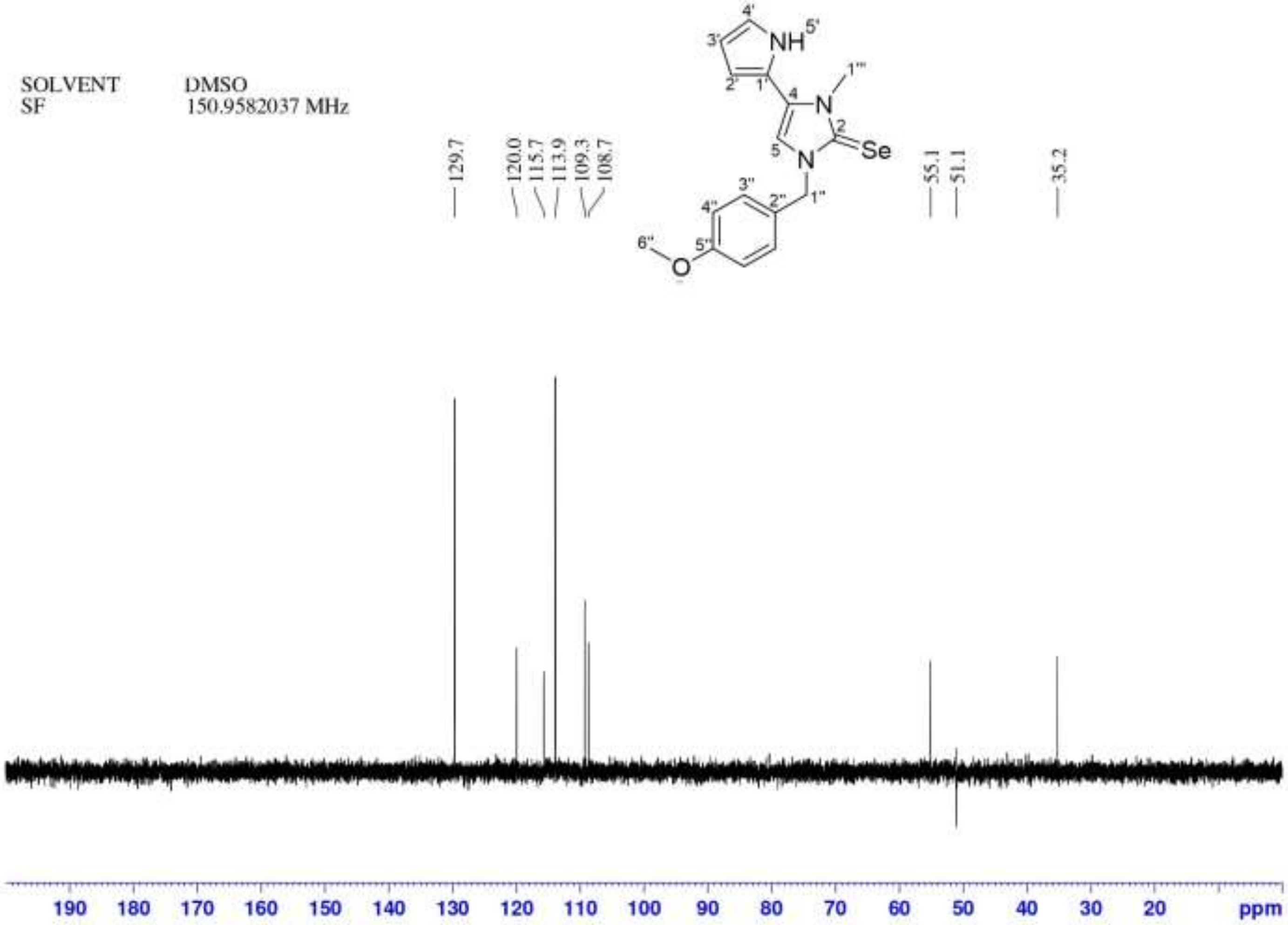

Figure S160. **$^{13}C\{^1H\}$-DEPT-NMR of compound 9g**

^{77}Se-NMR 1-(4-Methoxybenzyl)-3-methyl-4-(1H-pyrrol-2-yl)-1,3-dihydro-2H-imidazole-2-selenone (9g):

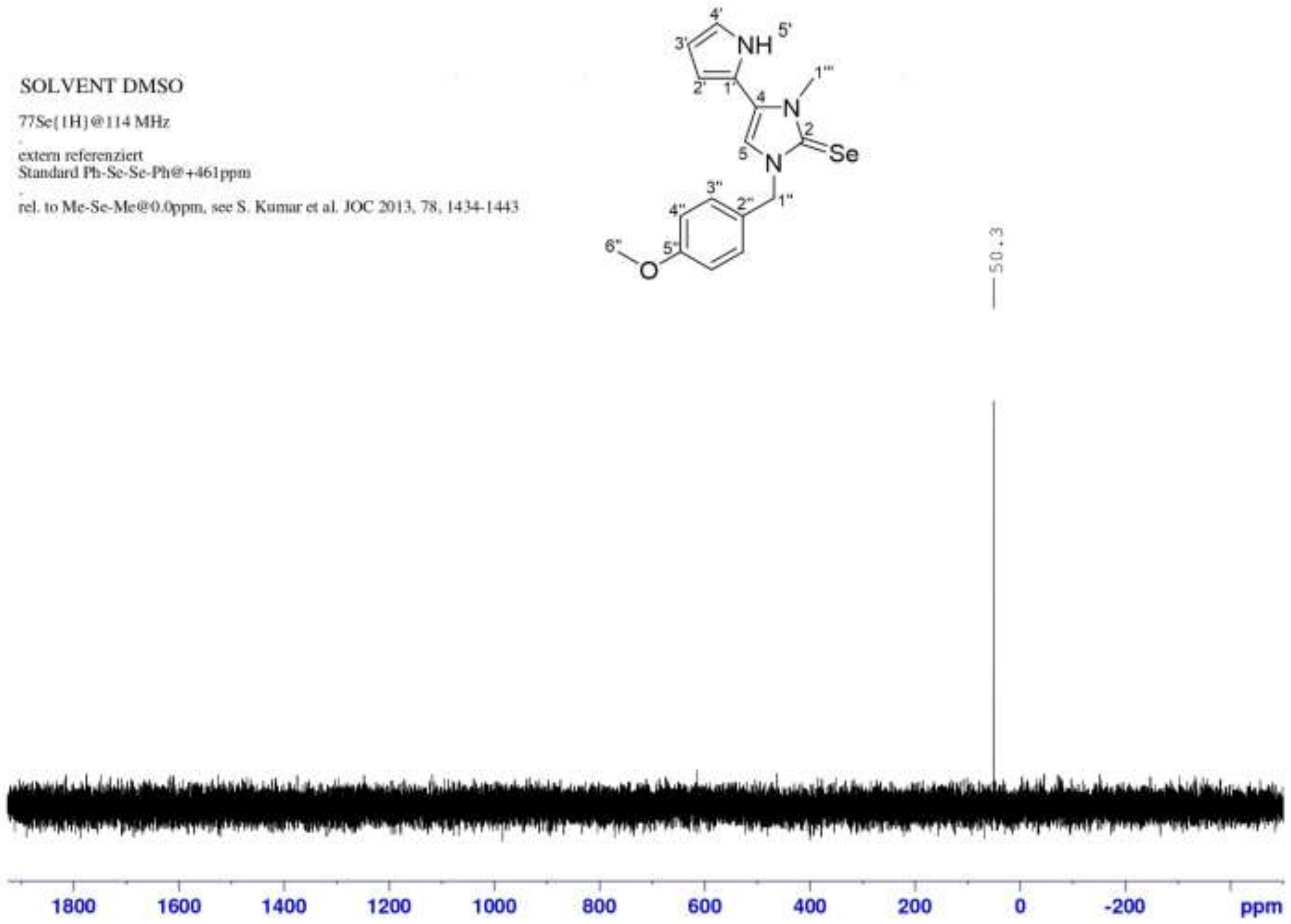

Figure S161. **^{77}Se-NMR of compound 9g**

HSQC-NMR 1-(4-Methoxybenzyl)-3-methyl-4-(1H-pyrrol-2-yl)-1,3-dihydro-2H-imidazole-2-selenone (9g):

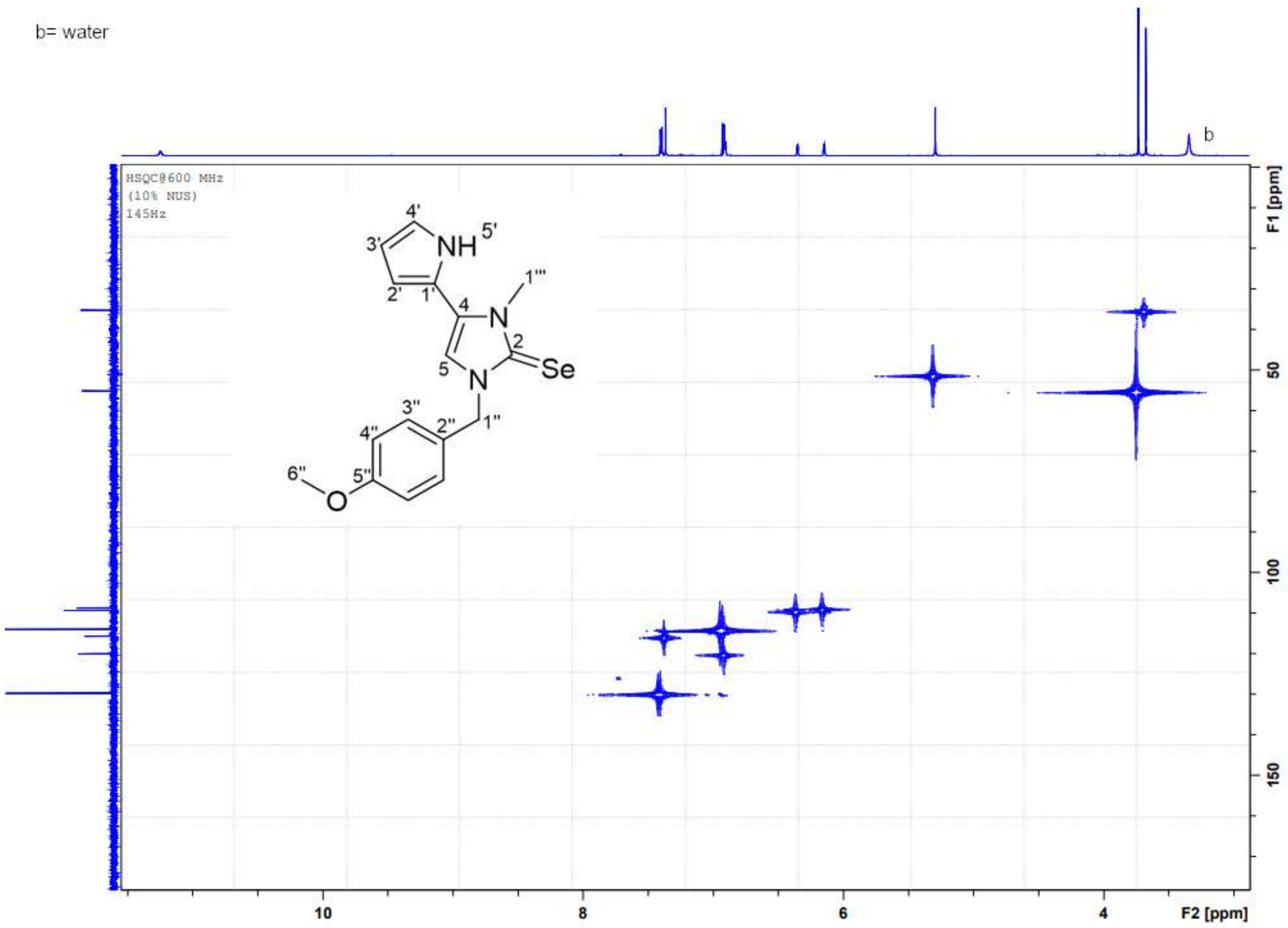

Figure S162. HSQC-NMR of compound 9g

HMBC-NMR 1-(4-Methoxybenzyl)-3-methyl-4-(1H-pyrrol-2-yl)-1,3-dihydro-2H-imidazole-2-selenone (9g):

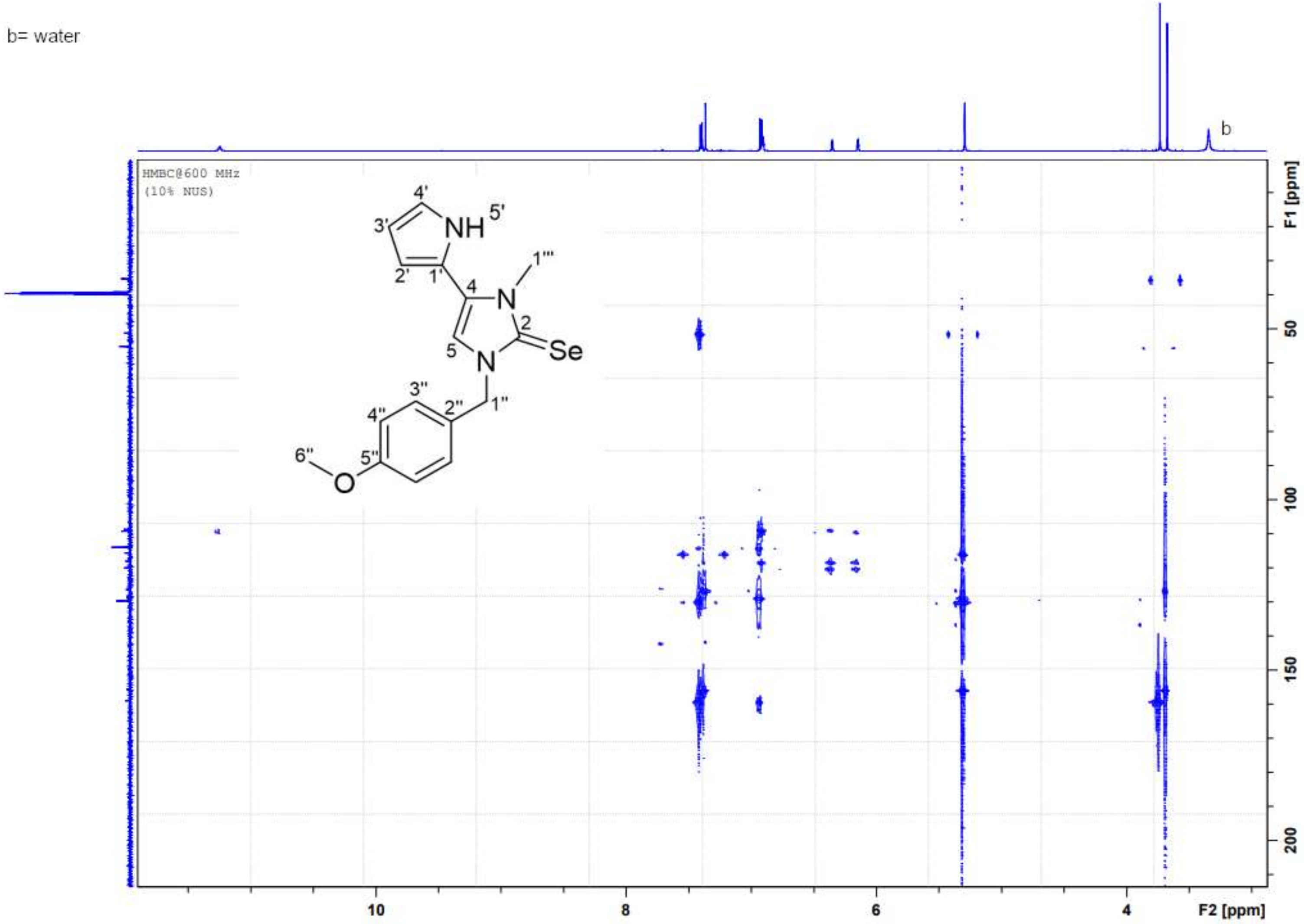

Figure S163. HMBC-NMR of compound 9g

^{1}H-NMR 1-Benzyl-3-(*tert*-butyl)-4-(1H-pyrrol-2-yl)-1,3-dihydro-2H-imidazole-2-selenone (9h):

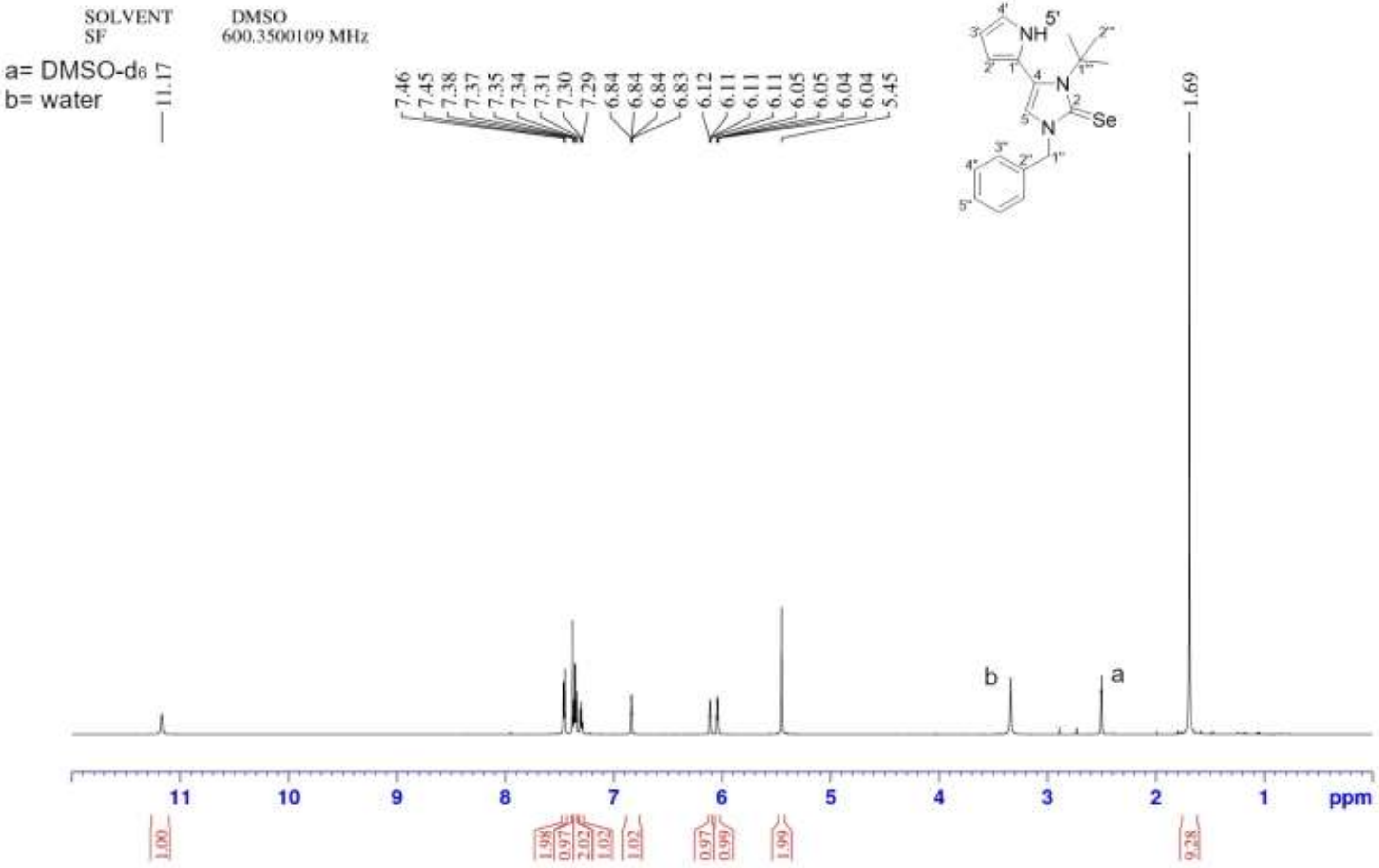

Figure S164. ^{1}H-NMR of compound 9h

$^{13}C\{^{1}H\}$-NMR 1-Benzyl-3-(*tert*-butyl)-4-(1H-pyrrol-2-yl)-1,3-dihydro-2H-imidazole-2-selenone (9h):

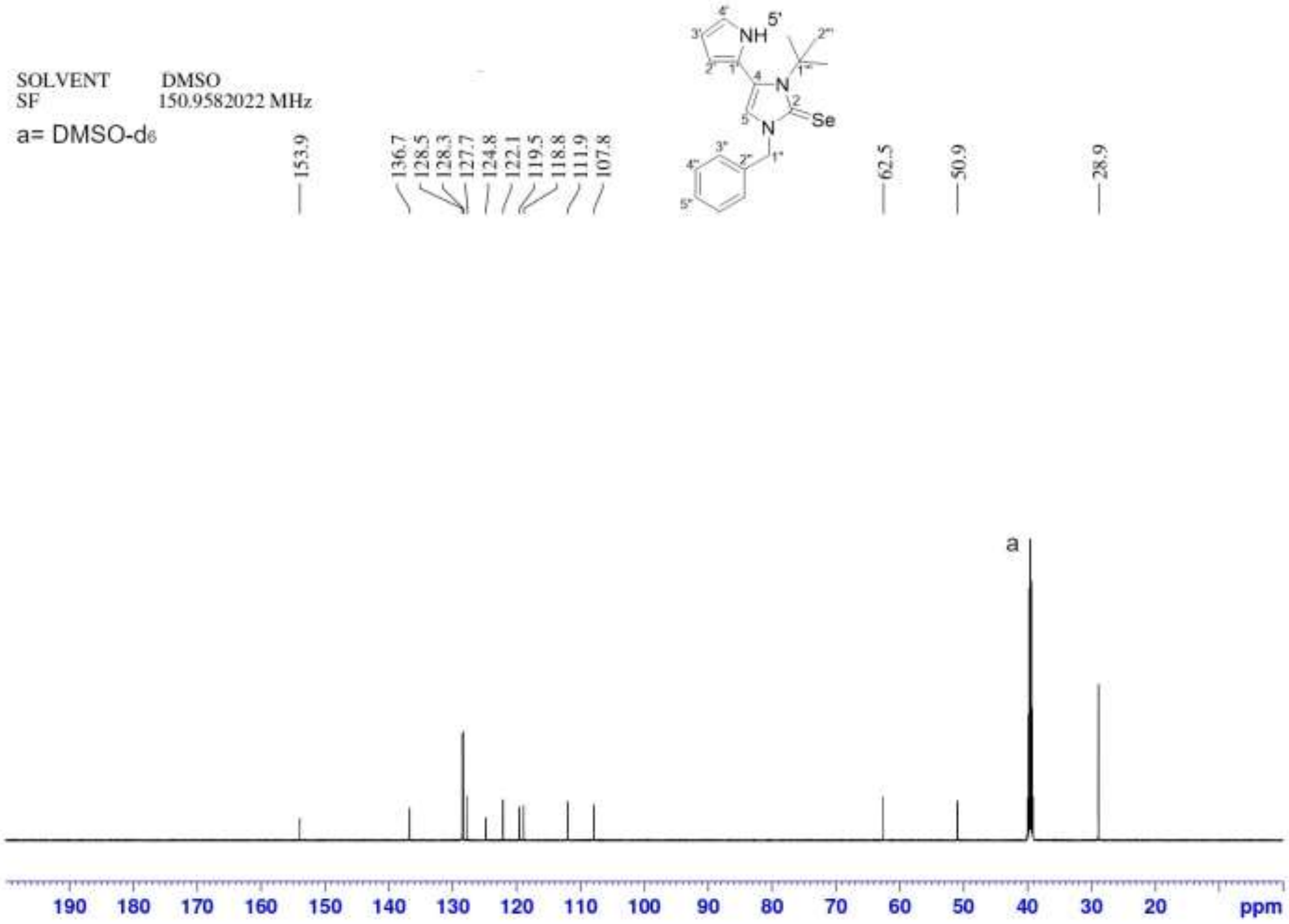

Figure S165. **$^{13}C\{^{1}H\}$-NMR of compound 9h**

$^{13}C\{^1H\}$-DEPT-NMR 1-Benzyl-3-(*tert*-butyl)-4-(1H-pyrrol-2-yl)-1,3-dihydro-2H-imidazole-2-selenone (9h):

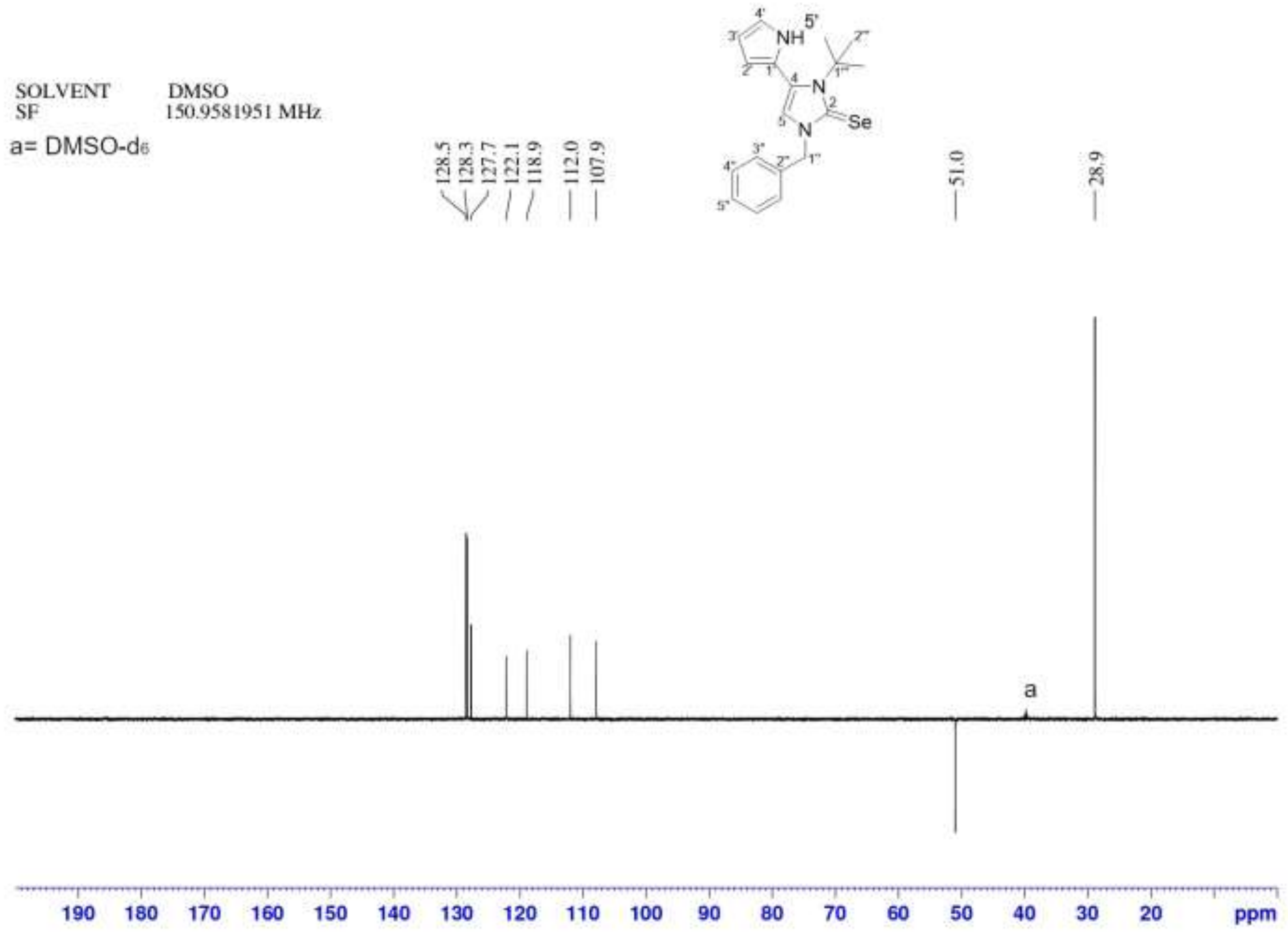

Figure S166. **$^{13}C\{^1H\}$-DEPT-NMR of compound 9h**

^{77}Se -NMR 1-Benzyl-3-(*tert*-butyl)-4-(1H-pyrrol-2-yl)-1,3-dihydro-2H-imidazole-2-selenone (9h):

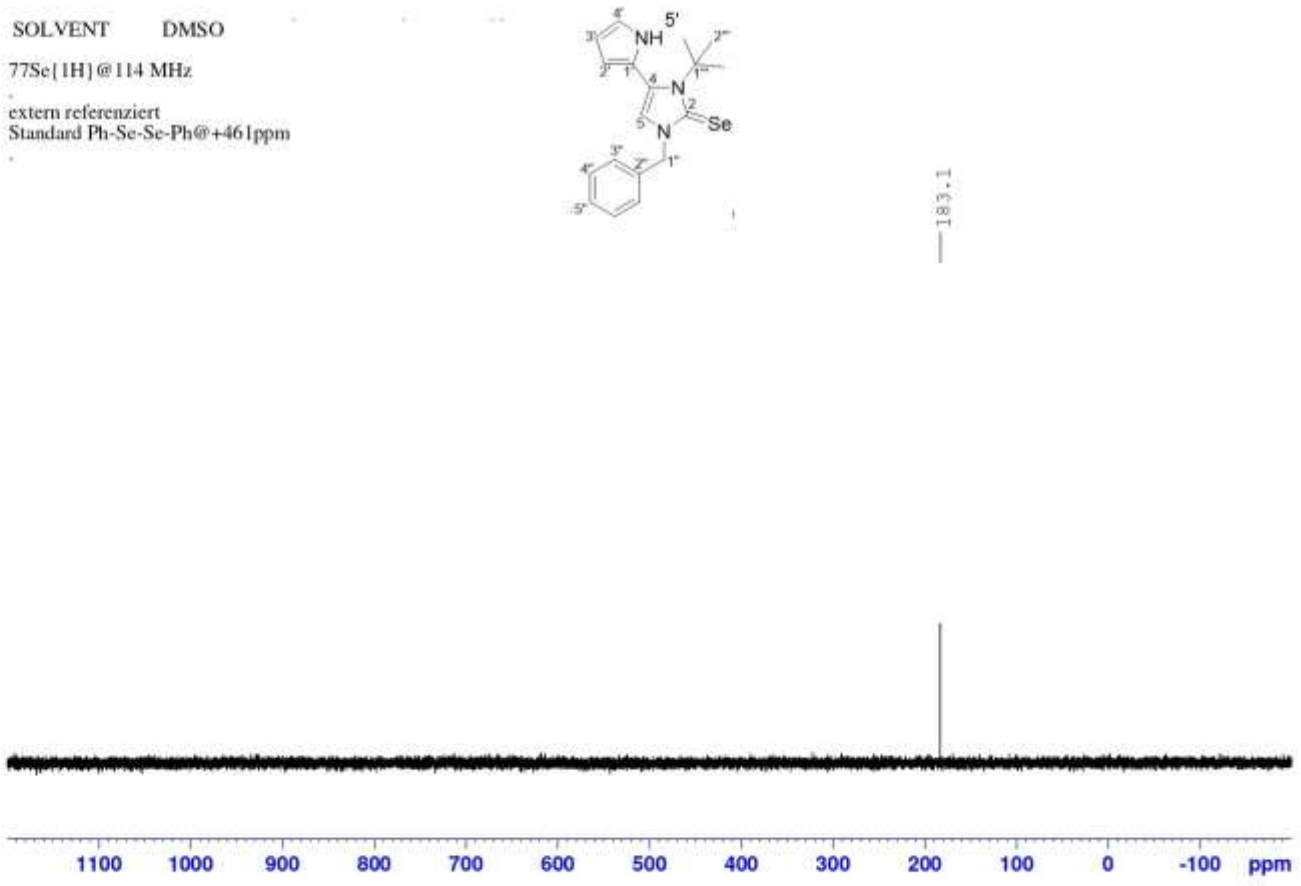

Figure S167. ^{77}**Se-NMR of compound 9h**

HSQC-NMR 1-Benzyl-3-(*tert*-butyl)-4-(1H-pyrrol-2-yl)-1,3-dihydro-2H-imidazole-2-selenone (9h):

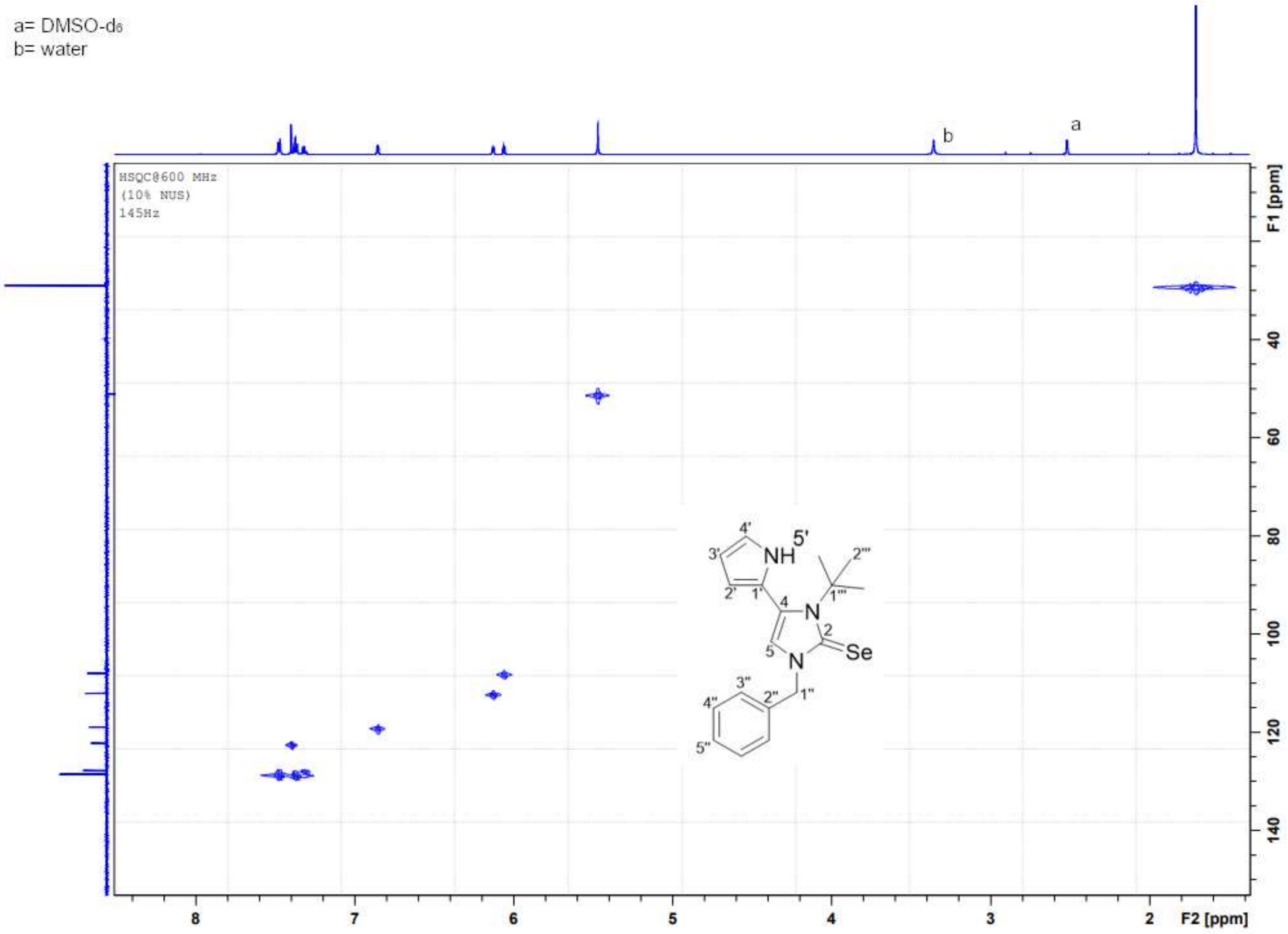

Figure S168. HSQC-NMR of compound 9h

HMBC-NMR 1-Benzyl-3-(*tert*-butyl)-4-(1H-pyrrol-2-yl)-1,3-dihydro-2H-imidazole-2-selenone (9h):

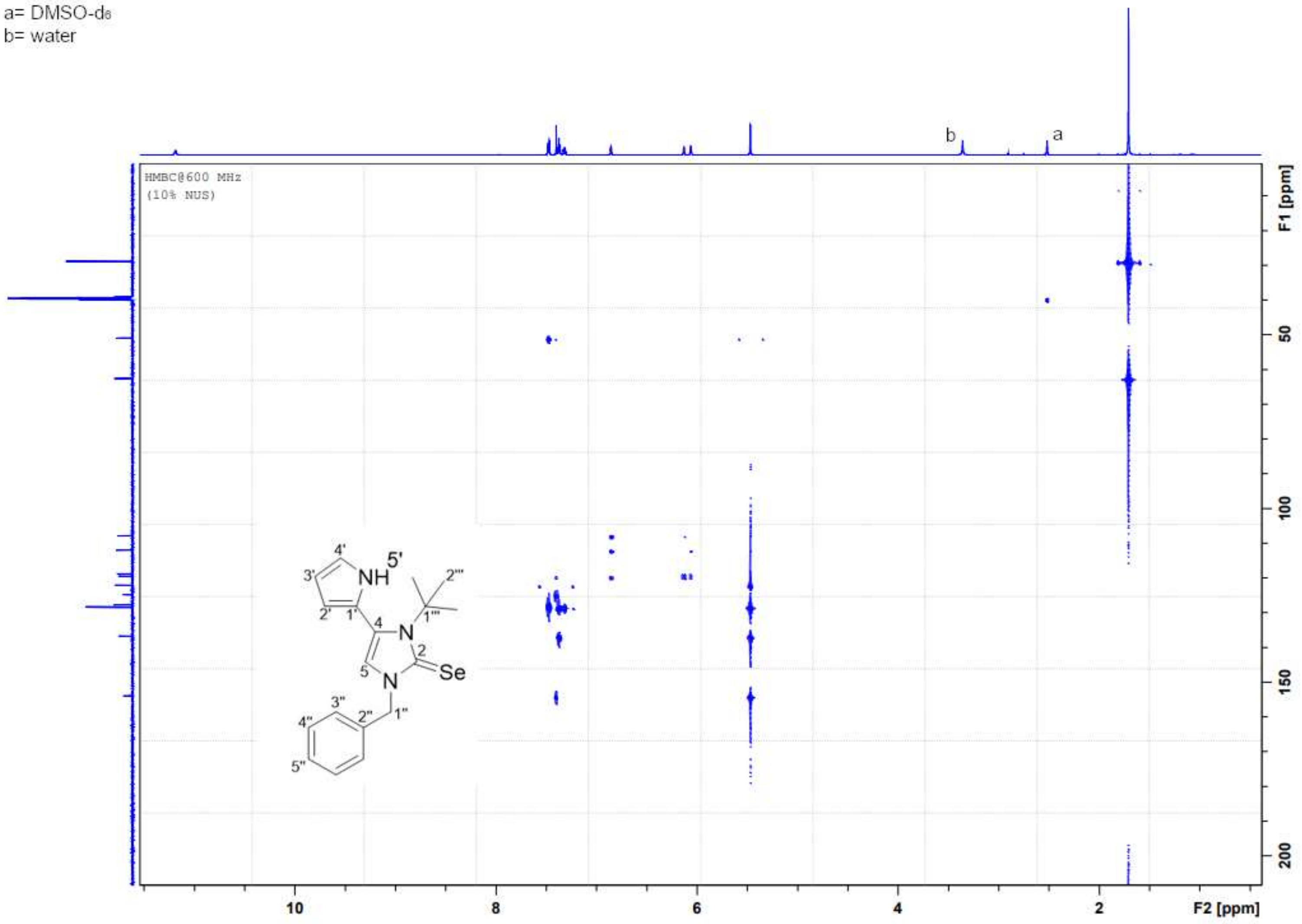

Figure S169. HMBC-NMR of compound 9h

^{1}H-NMR 3-(*tert*-Butyl)-1-(4-methylbenzyl)-4-(1H-pyrrol-2-yl)-1,3-dihydro-2H-imidazole-2-selenone (9i):

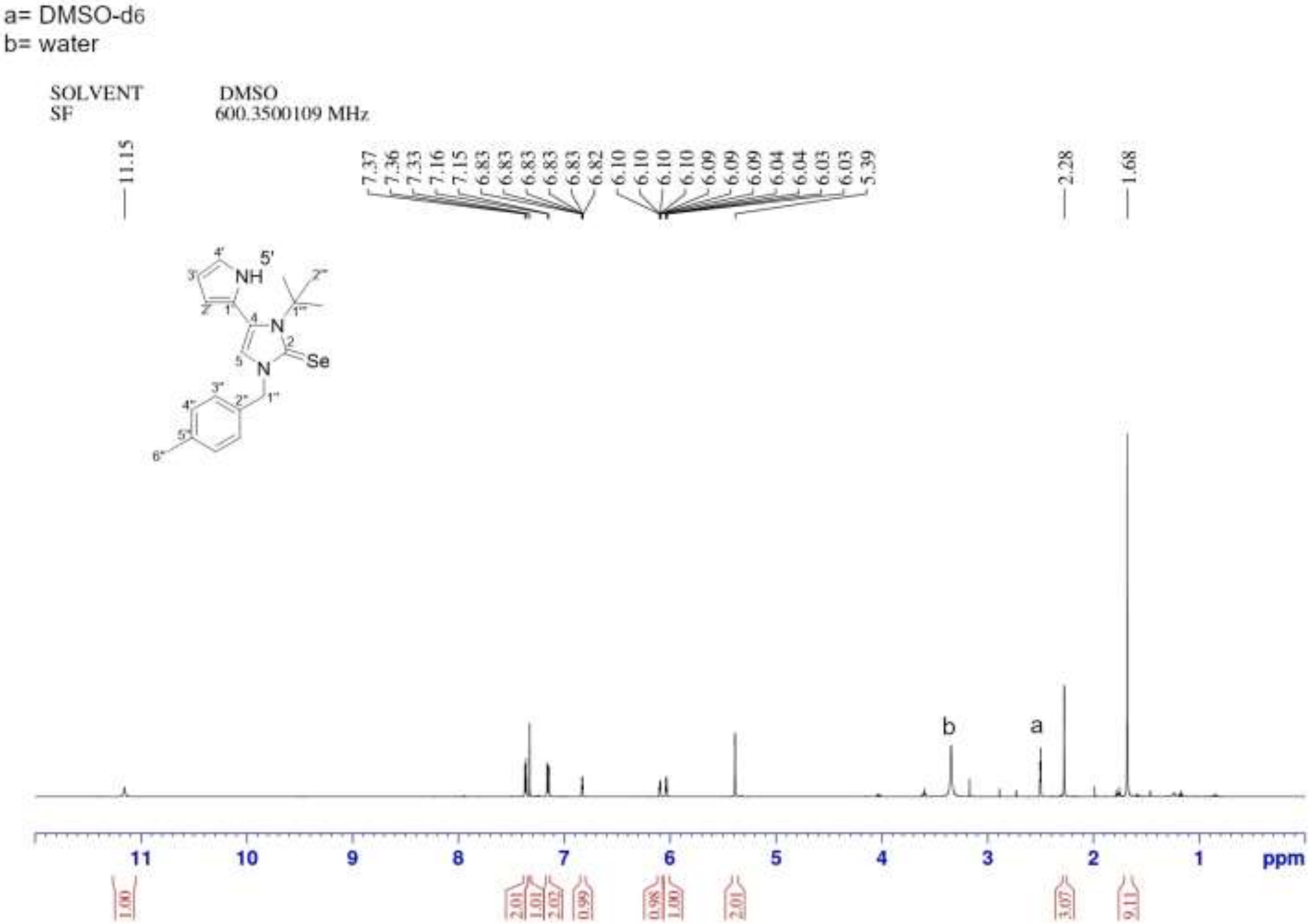

Figure S170. ^{1}H-NMR of compound 9i

$^{13}C\{^1H\}$-NMR 3-(*tert*-Butyl)-1-(4-methylbenzyl)-4-(1H-pyrrol-2-yl)-1,3-dihydro-2H-imidazole-2-selenone (9i):

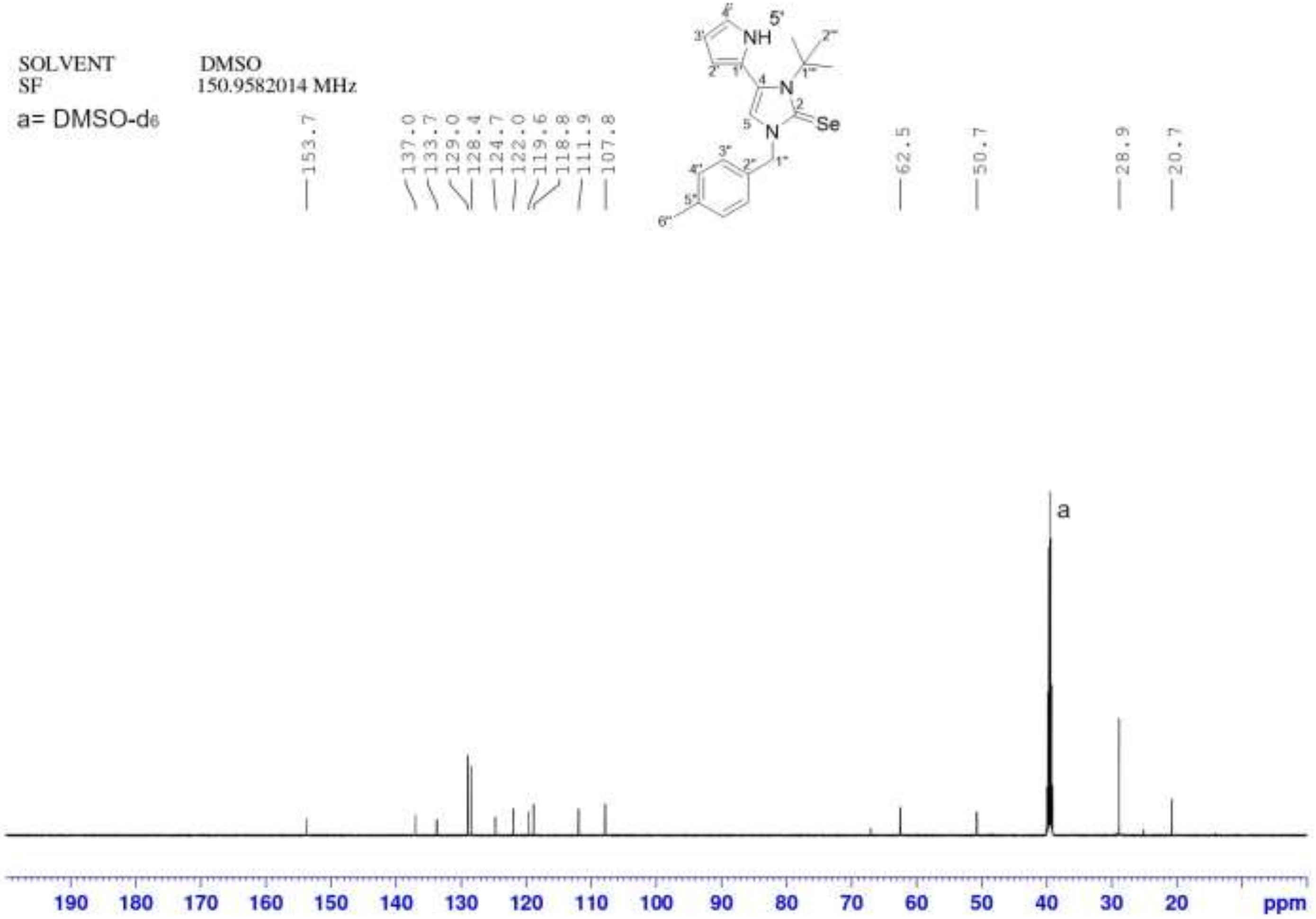

Figure S171. $^{13}C\{^1H\}$**-NMR of compound 9i**

$^{13}C\{^1H\}$-DEPT-NMR 3-(*tert*-Butyl)-1-(4-methylbenzyl)-4-(1H-pyrrol-2-yl)-1,3-dihydro-2H-imidazole-2-selenone (9i):

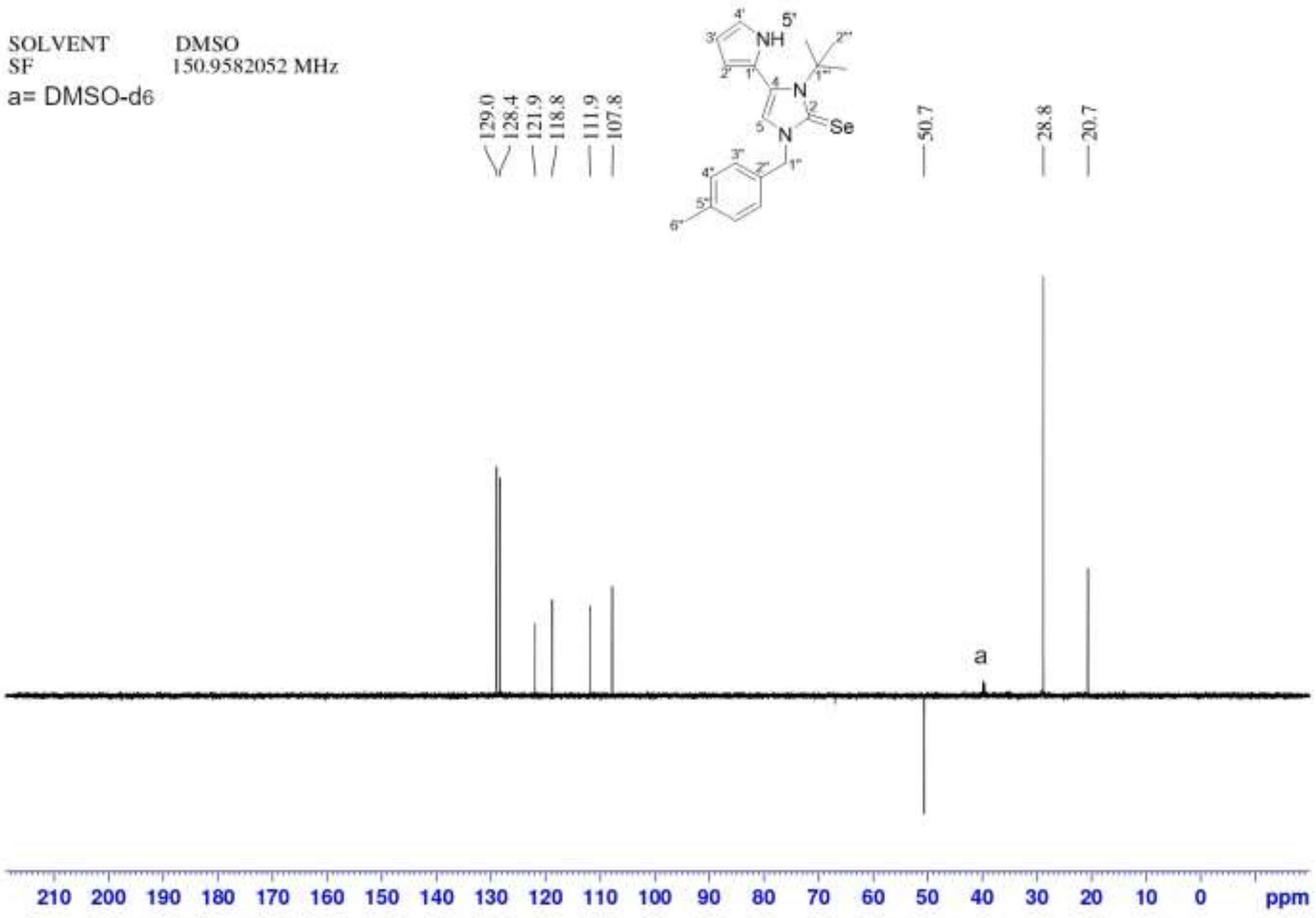

Figure S172. $^{13}C\{^1H\}$-DEPT-NMR of compound 9i

^{77}Se-NMR 3-(*tert*-Butyl)-1-(4-methylbenzyl)-4-(1H-pyrrol-2-yl)-1,3-dihydro-2H-imidazole-2-selenone (9i):

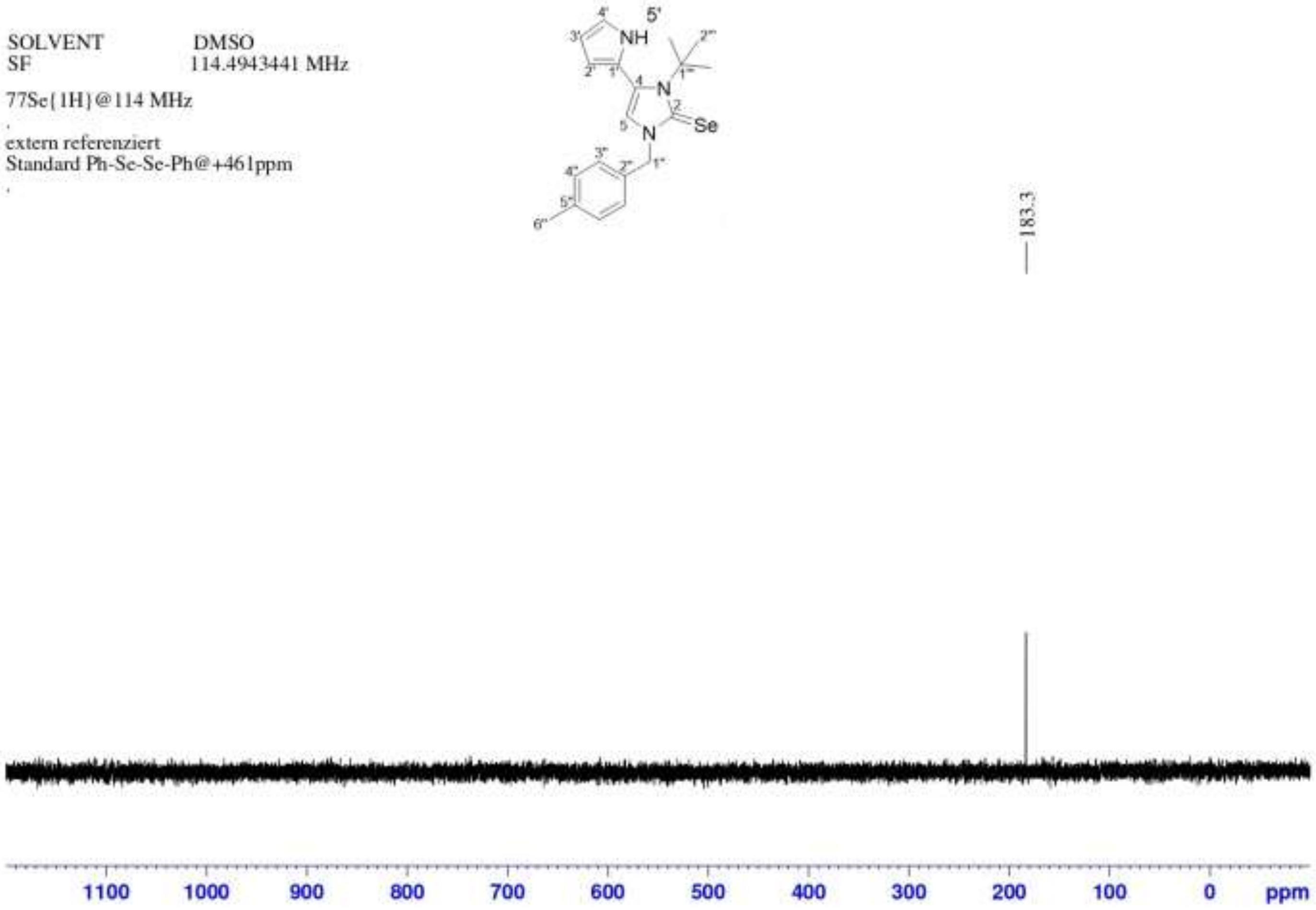

Figure S173. ^{77}**Se-NMR of compound 9i**

HSQC-NMR 3-(*tert*-Butyl)-1-(4-methylbenzyl)-4-(1H-pyrrol-2-yl)-1,3-dihydro-2H-imidazole-2-selenone (9i):

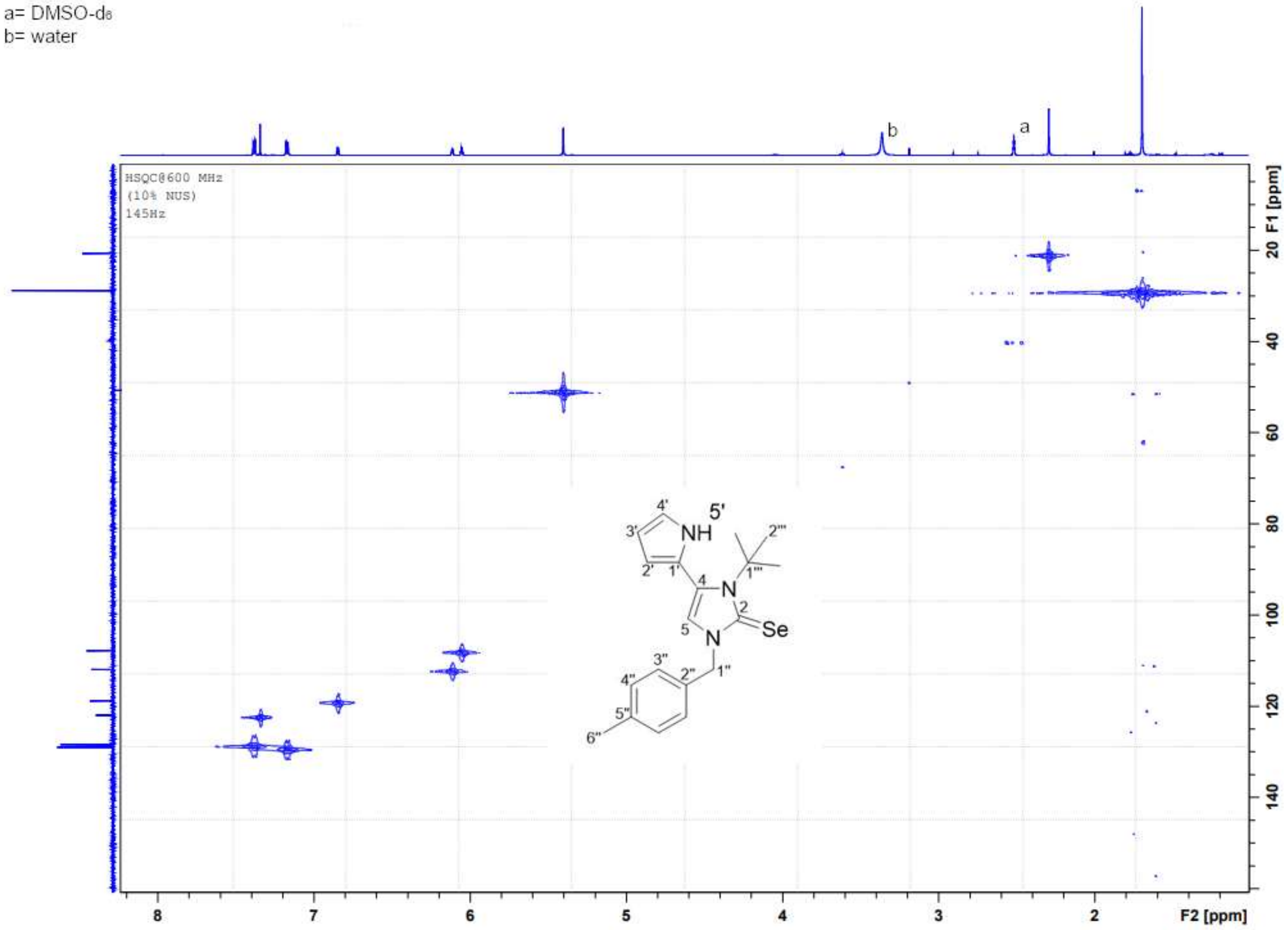

Figure S174. HSQC-NMR of compound 9i

HMBC-NMR 3-(*tert*-Butyl)-1-(4-methylbenzyl)-4-(1H-pyrrol-2-yl)-1,3-dihydro-2H-imidazole-2-selenone (9i):

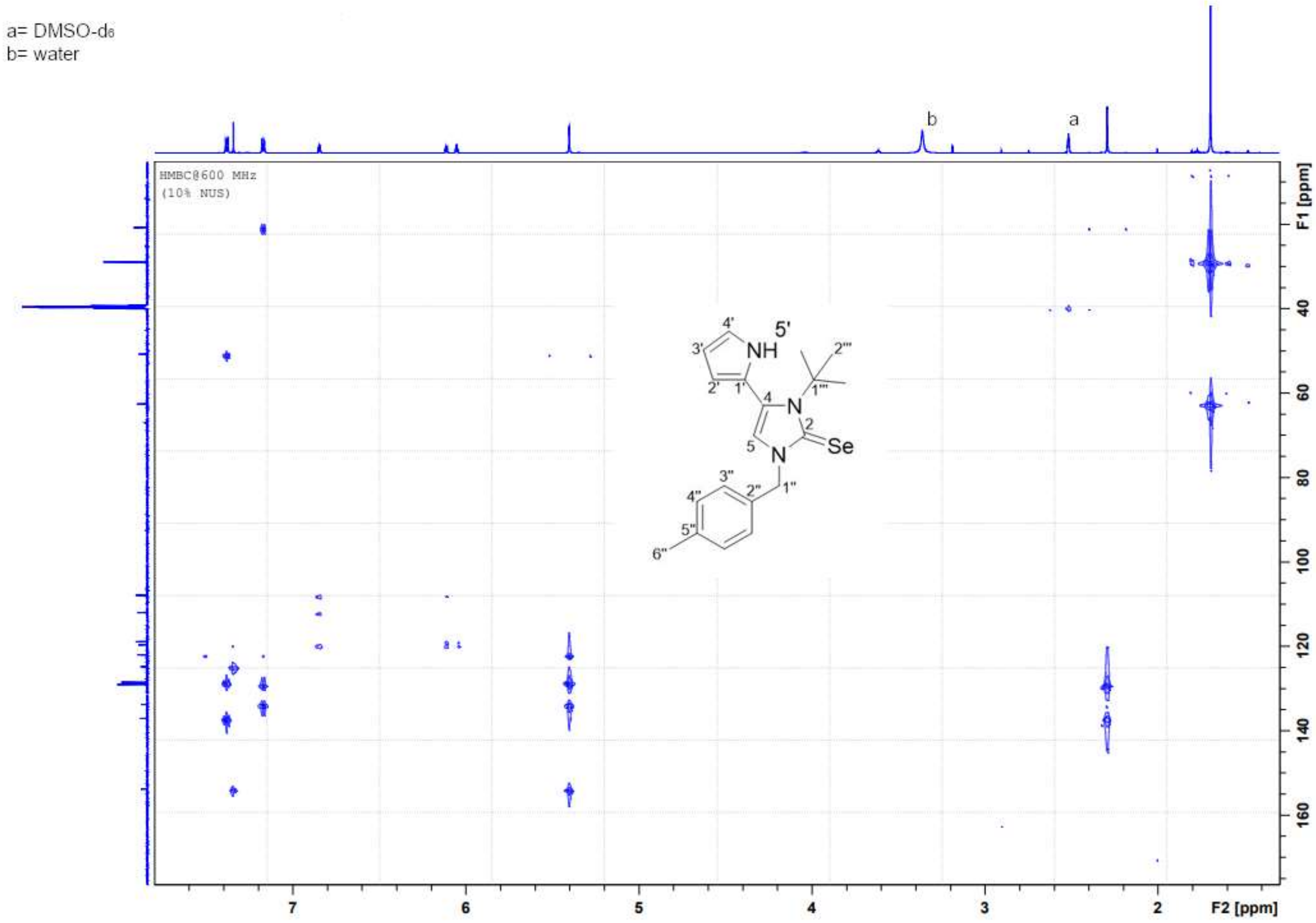

Figure S175. HMBC-NMR of compound 9i

^{1}H-NMR 2-(1,3-Dimethyl-2-selenoxo-2,3-dihydro-1H-imidazol-4-yl)pyrrol-1-ide (10a):

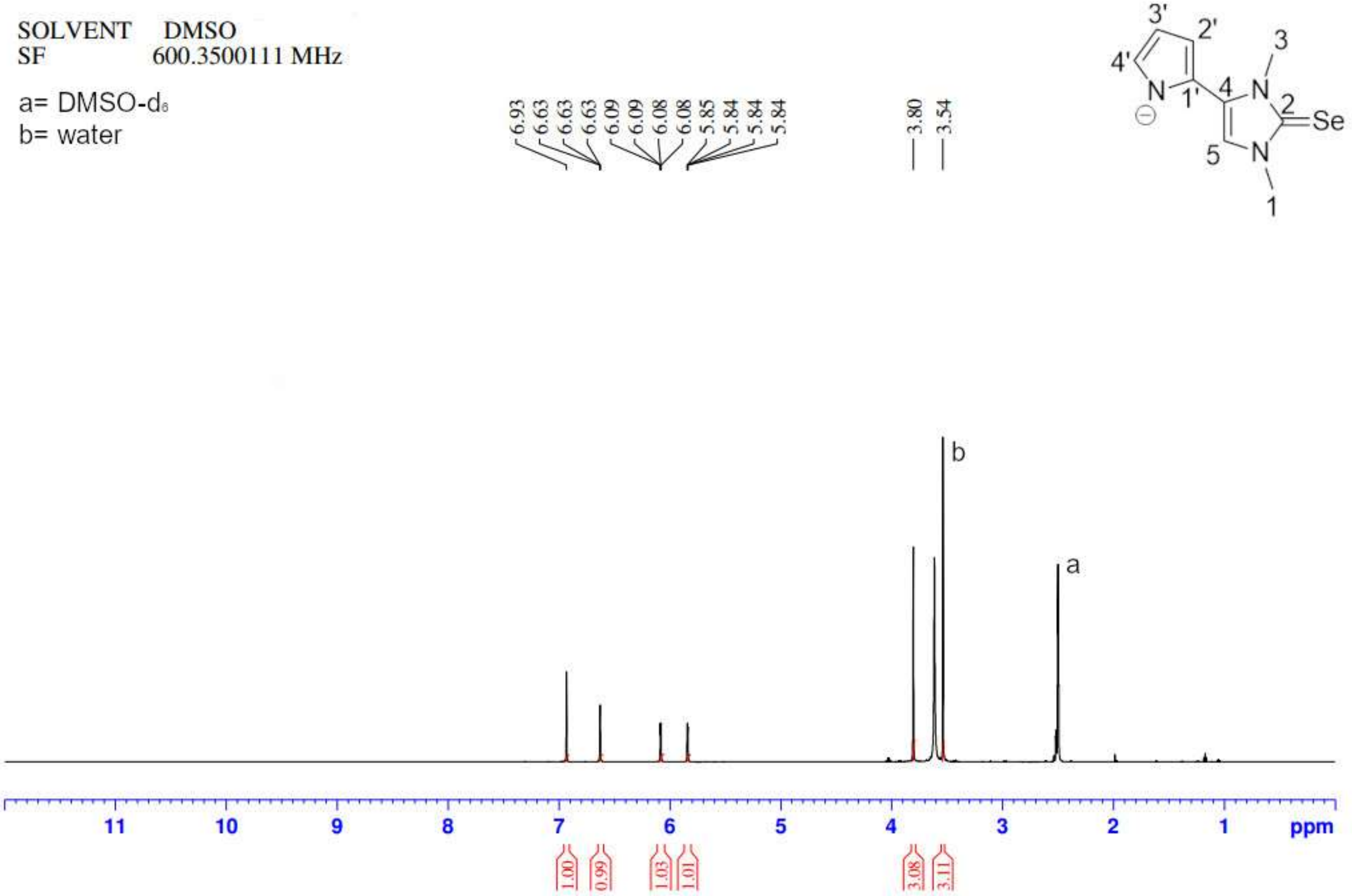

Figure S176. ^{1}H-NMR of compound 10a

$^{13}C\{^1H\}$-NMR 2-(1,3-Dimethyl-2-selenoxo-2,3-dihydro-1H-imidazol-4-yl)pyrrol-1-ide (10a):

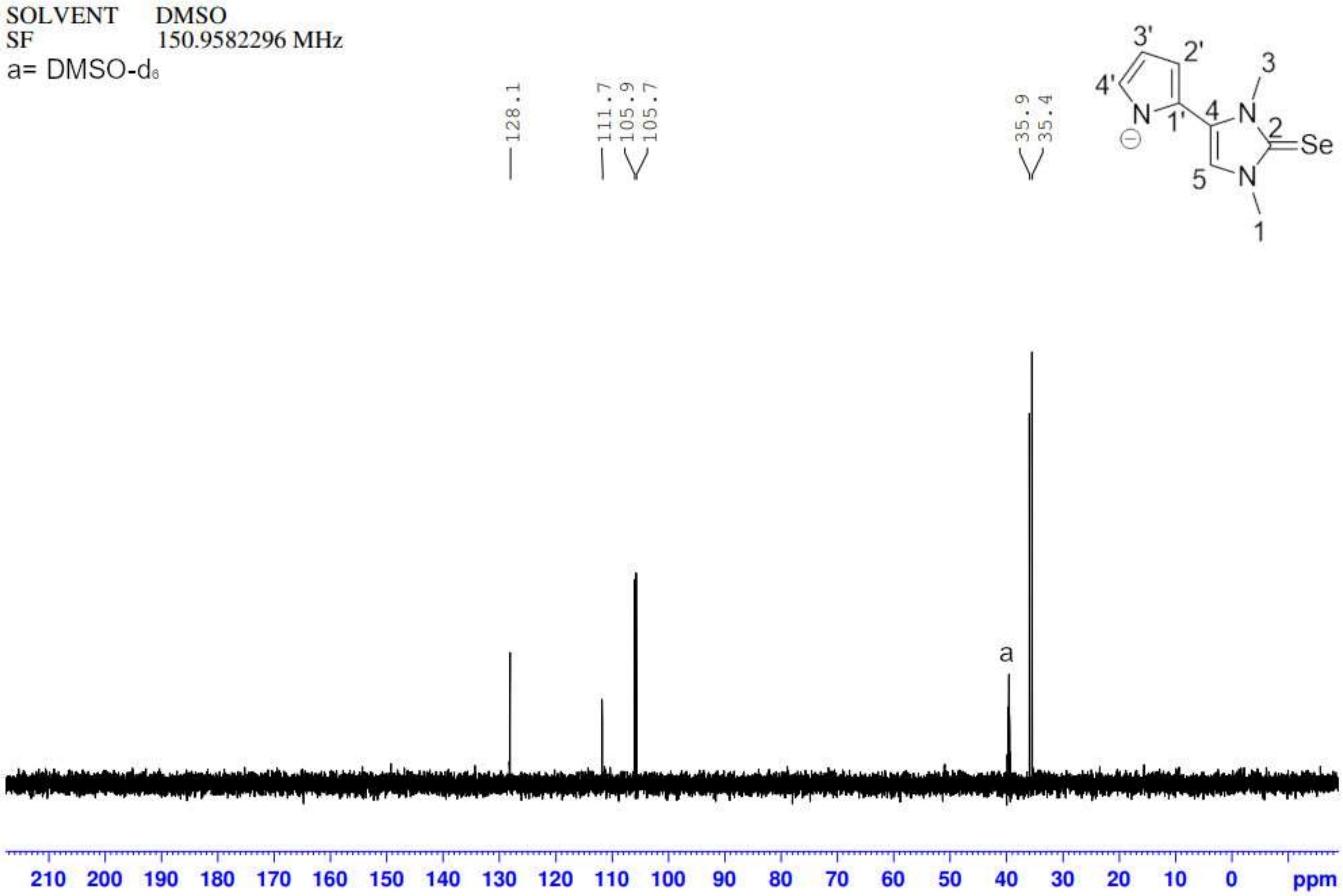

Figure S177. **$^{13}C\{^1H\}$-NMR of compound 10a**

$^{13}C\{^1H\}$-DEPT-NMR 2-(1,3-Dimethyl-2-selenoxo-2,3-dihydro-1H-imidazol-4-yl)pyrrol-1-ide (10a):

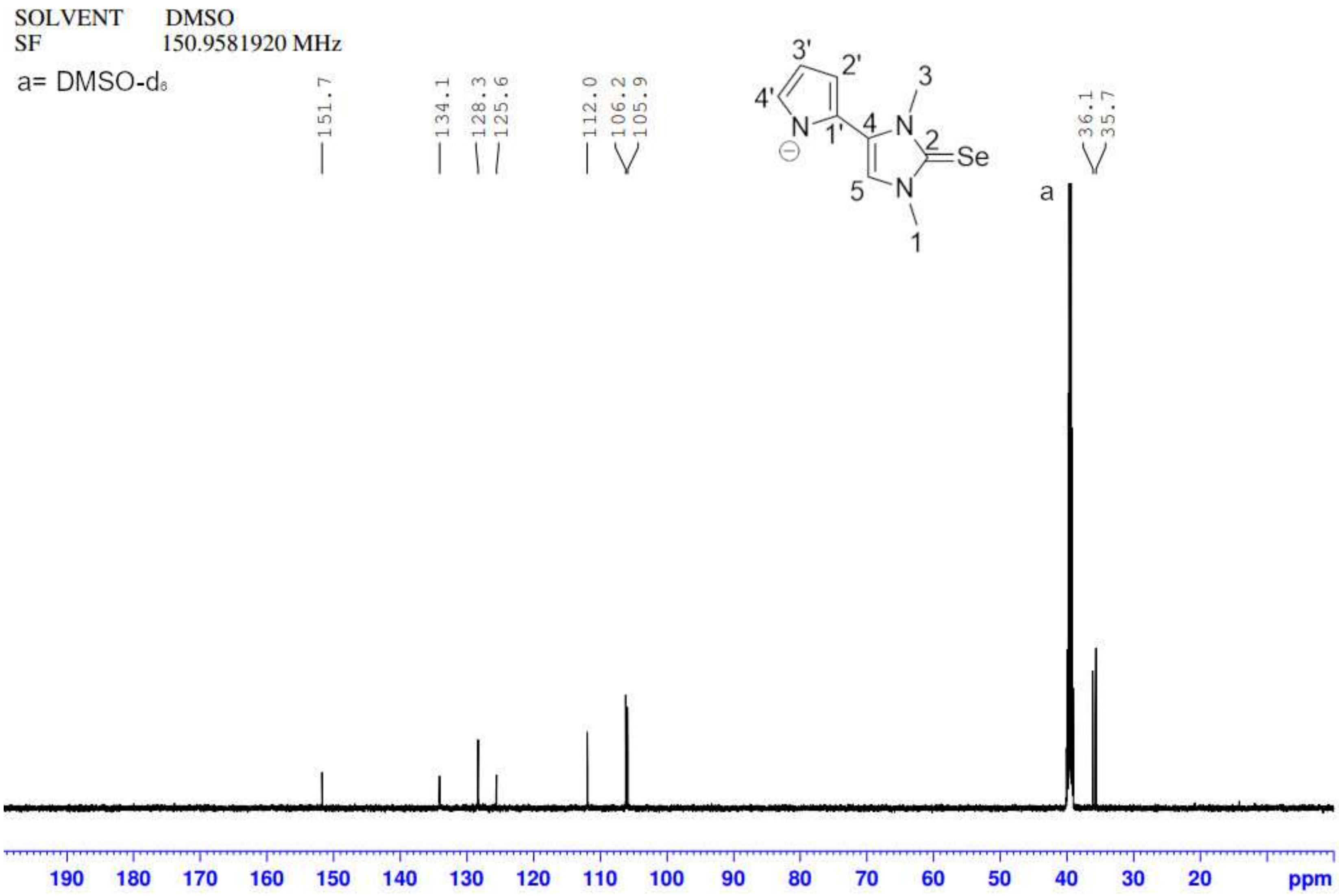

Figure S178. **$^{13}C\{^1H\}$-DEPT-NMR of compound 10a**

^{77}Se- 2-(1,3-Dimethyl-2-selenoxo-2,3-dihydro-1H-imidazol-4-yl)pyrrol-1-ide (10a):

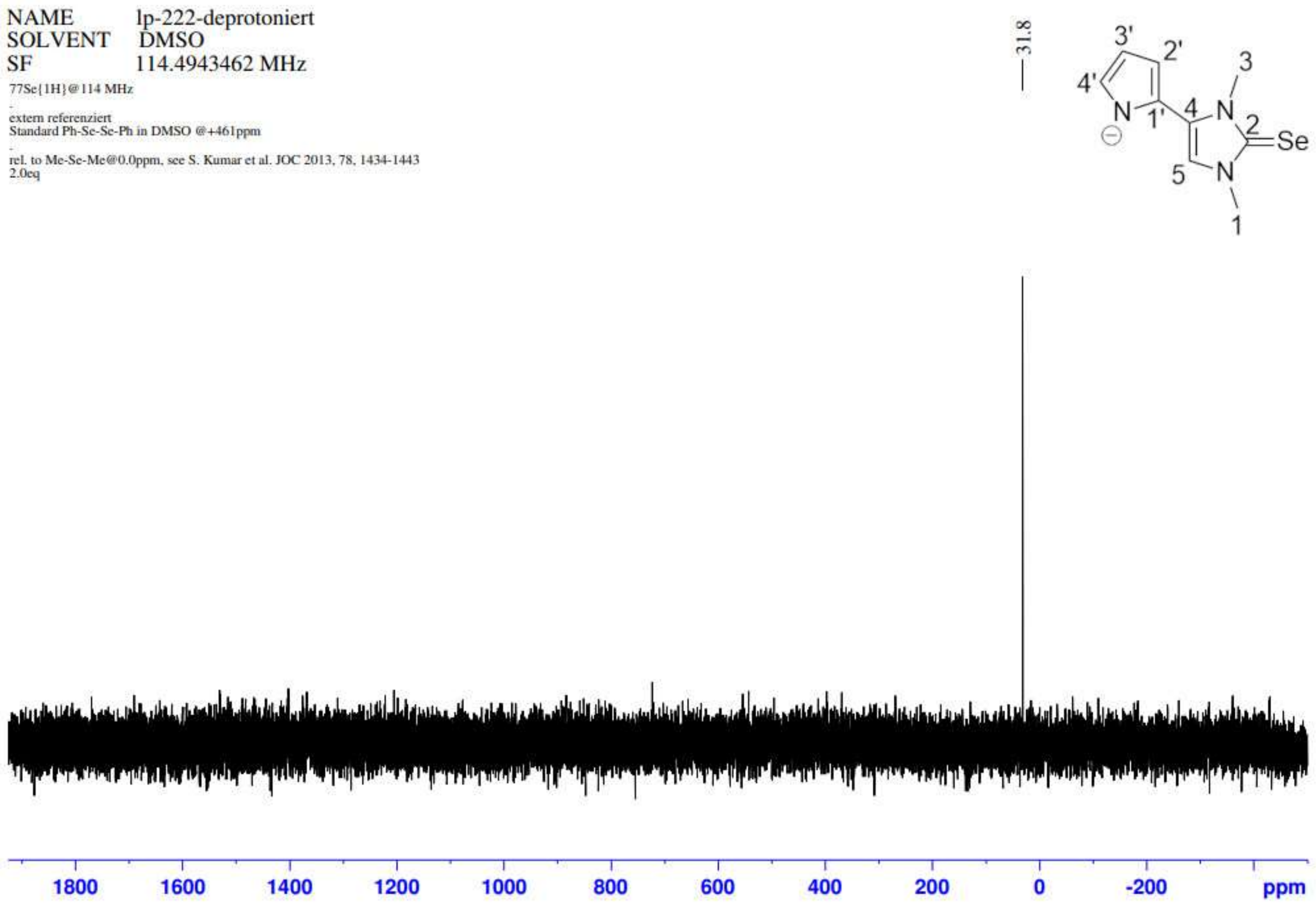

Figure S179. **^{77}Se-NMR of compound 10a**

HSQC-NMR 2-(1,3-Dimethyl-2-selenoxo-2,3-dihydro-1H-imidazol-4-yl)pyrrol-1-ide (10a):

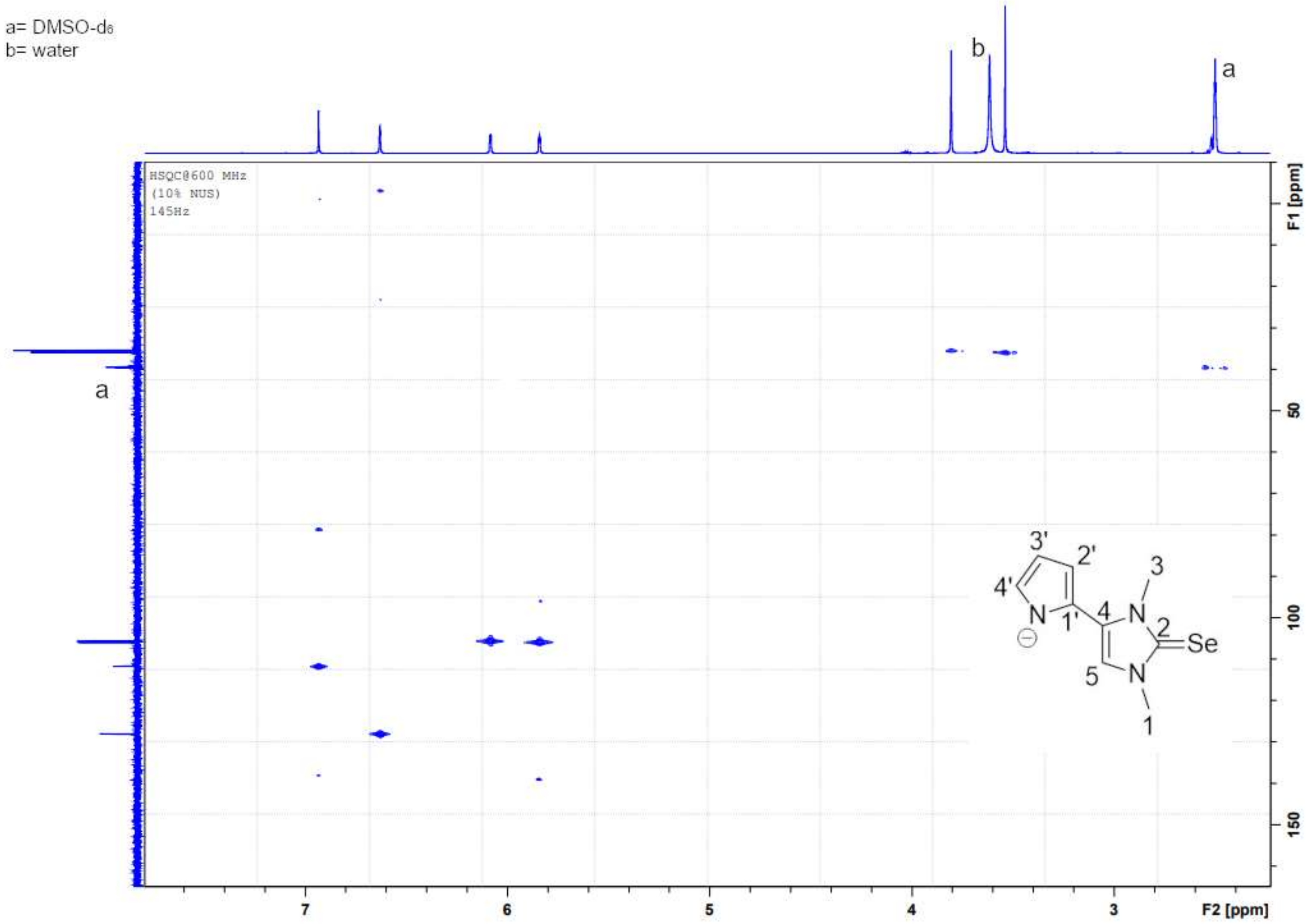

Figure S180. HSQC-NMR of compound 10a

HMBC-NMR 2-(1,3-Dimethyl-2-selenoxo-2,3-dihydro-1H-imidazol-4-yl)pyrrol-1-ide (10a):

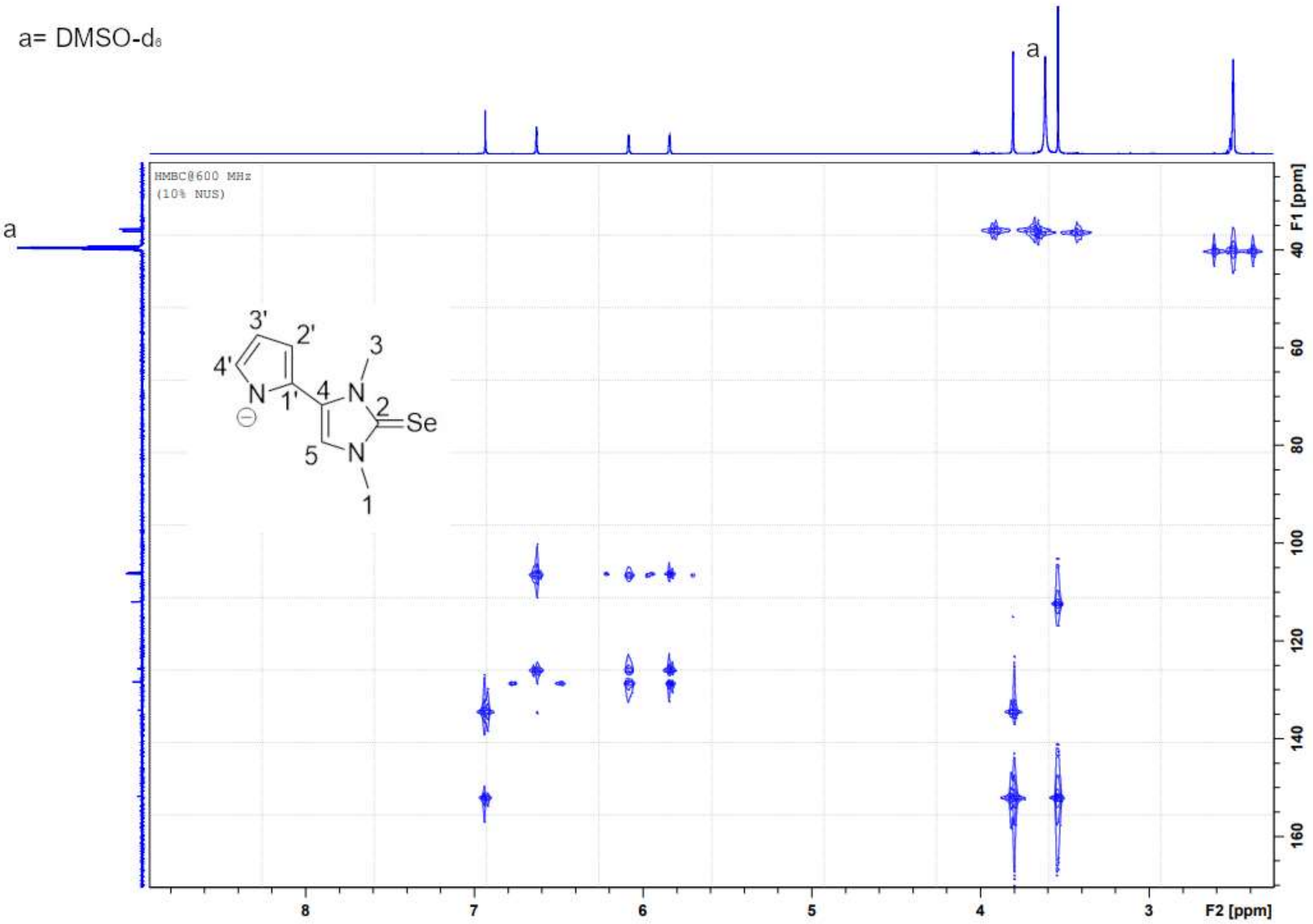

Figure S181. HMBC-NMR of compound 10a

^{1}H-NMR 2-(1,3-Dimethyl-2-selenoxo-2,3-dihydro-1H-imidazol-4-yl)pyrrol-1-ide (10a) NMR titration:

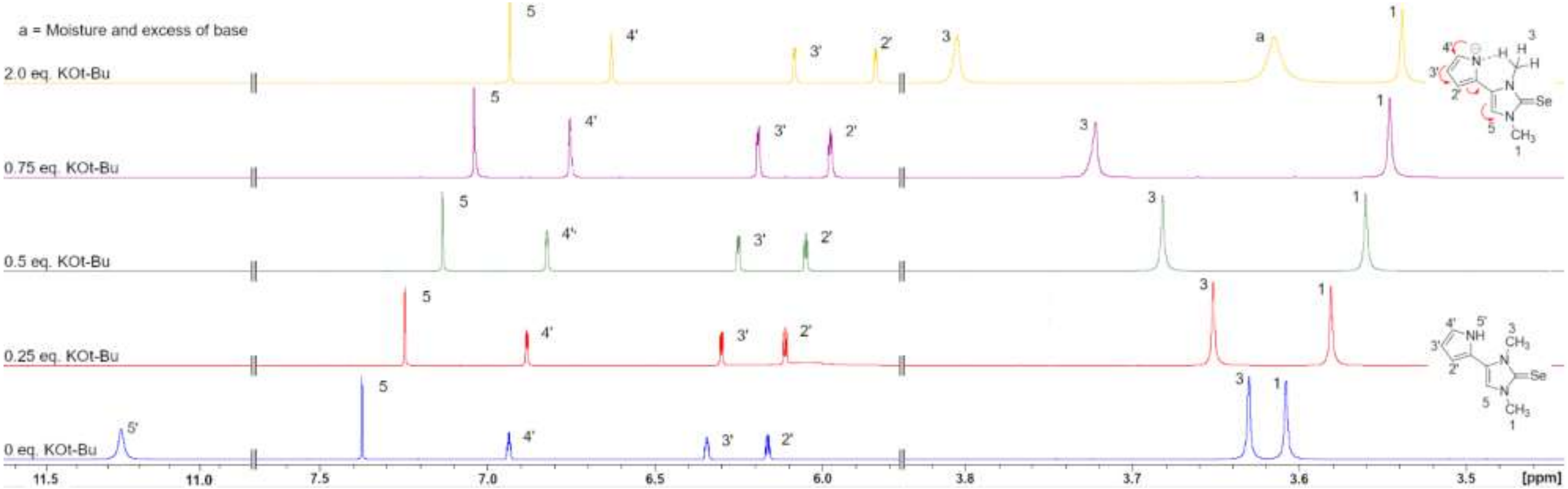

Figure S182. Comparison of the ^{1}H-NMR spectra of 10a after base addition.

^{77}Se-NMR 2-(1,3-Dimethyl-2-selenoxo-2,3-dihydro-1H-imidazol-4-yl)pyrrol-1-ide (10a) NMR titration:

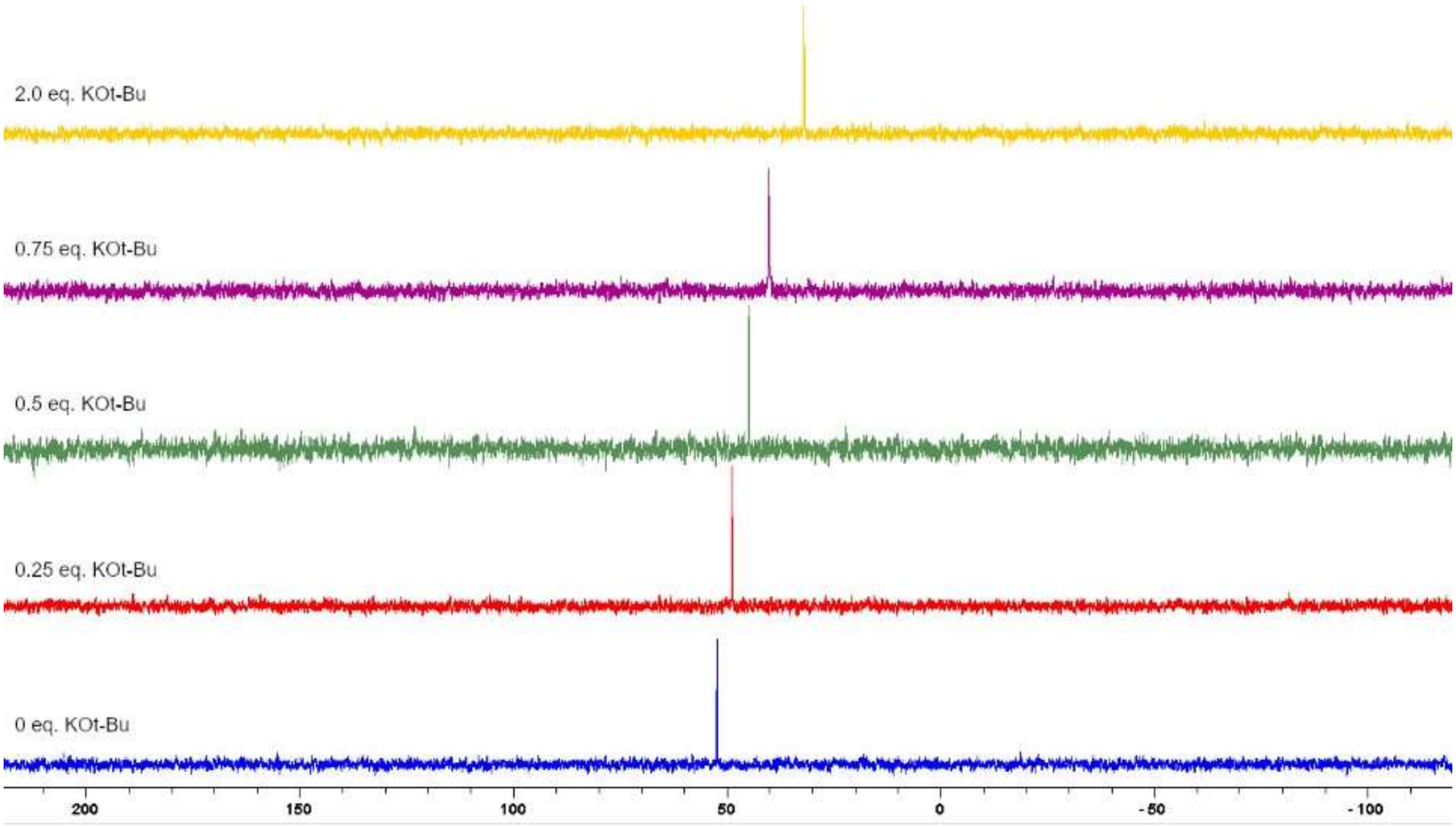

Figure S183. Comparison of the ^{77}Se-NMR spectra of 10a after base addition.

^{1}H-NMR 2-(1,3-Dimethyl-5-phenyl-2-selenoxo-2,3-dihydro-1H-imidazol-4-yl)pyrrol-1-ide (10b):

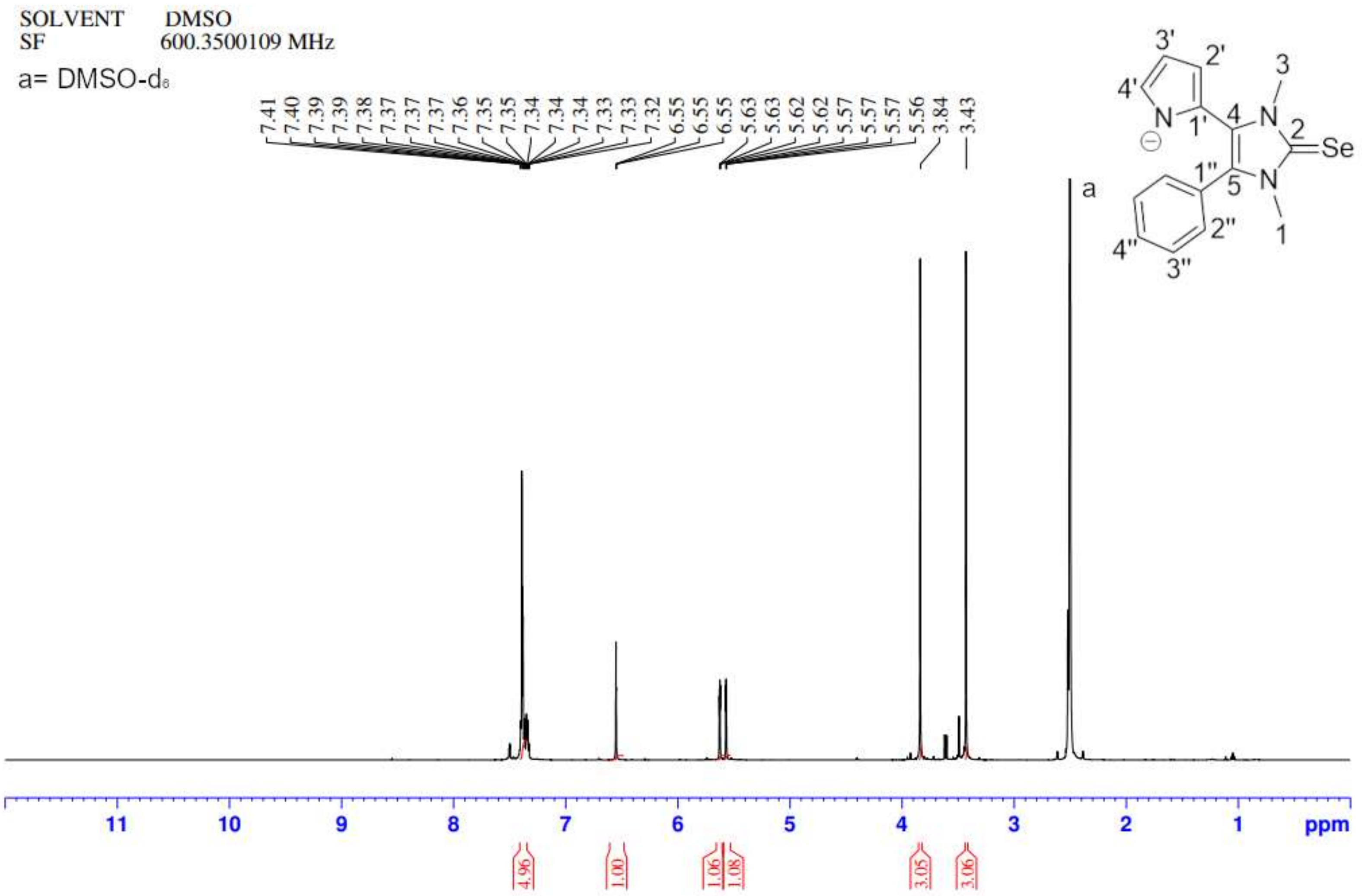

Figure S184. ^{1}H-NMR of compound 10b

$^{13}C\{^1H\}$-NMR 2-(1,3-Dimethyl-5-phenyl-2-selenoxo-2,3-dihydro-1H-imidazol-4-yl)pyrrol-1-ide (10b):

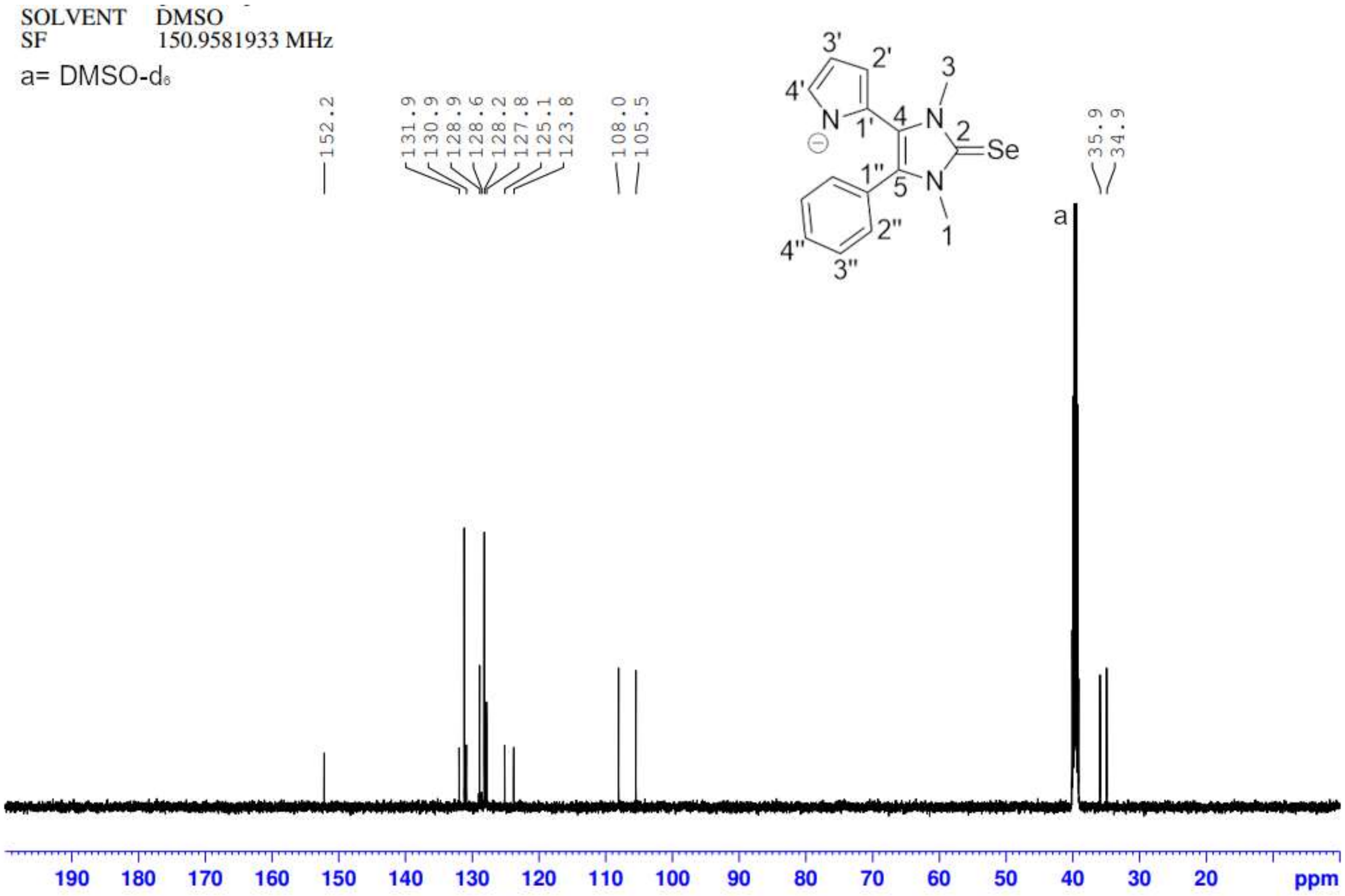

Figure S185. **$^{13}C\{^1H\}$-NMR of compound 10b**

^{13}C{^{1}H}-DEPT-NMR 2-(1,3-Dimethyl-5-phenyl-2-selenoxo-2,3-dihydro-1H-imidazol-4-yl)pyrrol-1-ide (10b):

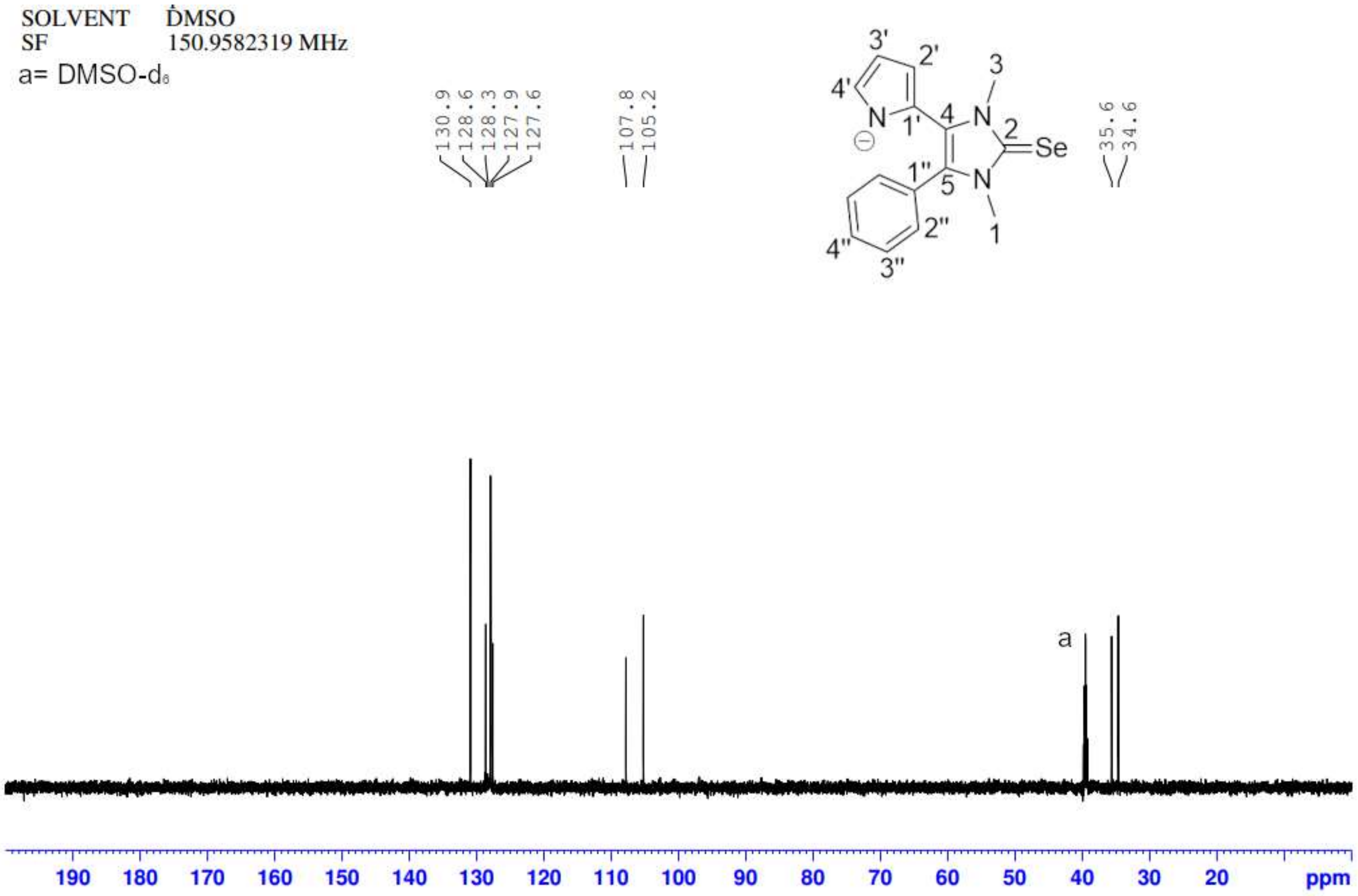

Figure S186. ^{13}C{^{1}H}-DEPT-NMR of compound 10b

^{77}Se-NMR 2-(1,3-Dimethyl-5-phenyl-2-selenoxo-2,3-dihydro-1H-imidazol-4-yl)pyrrol-1-ide (10b):

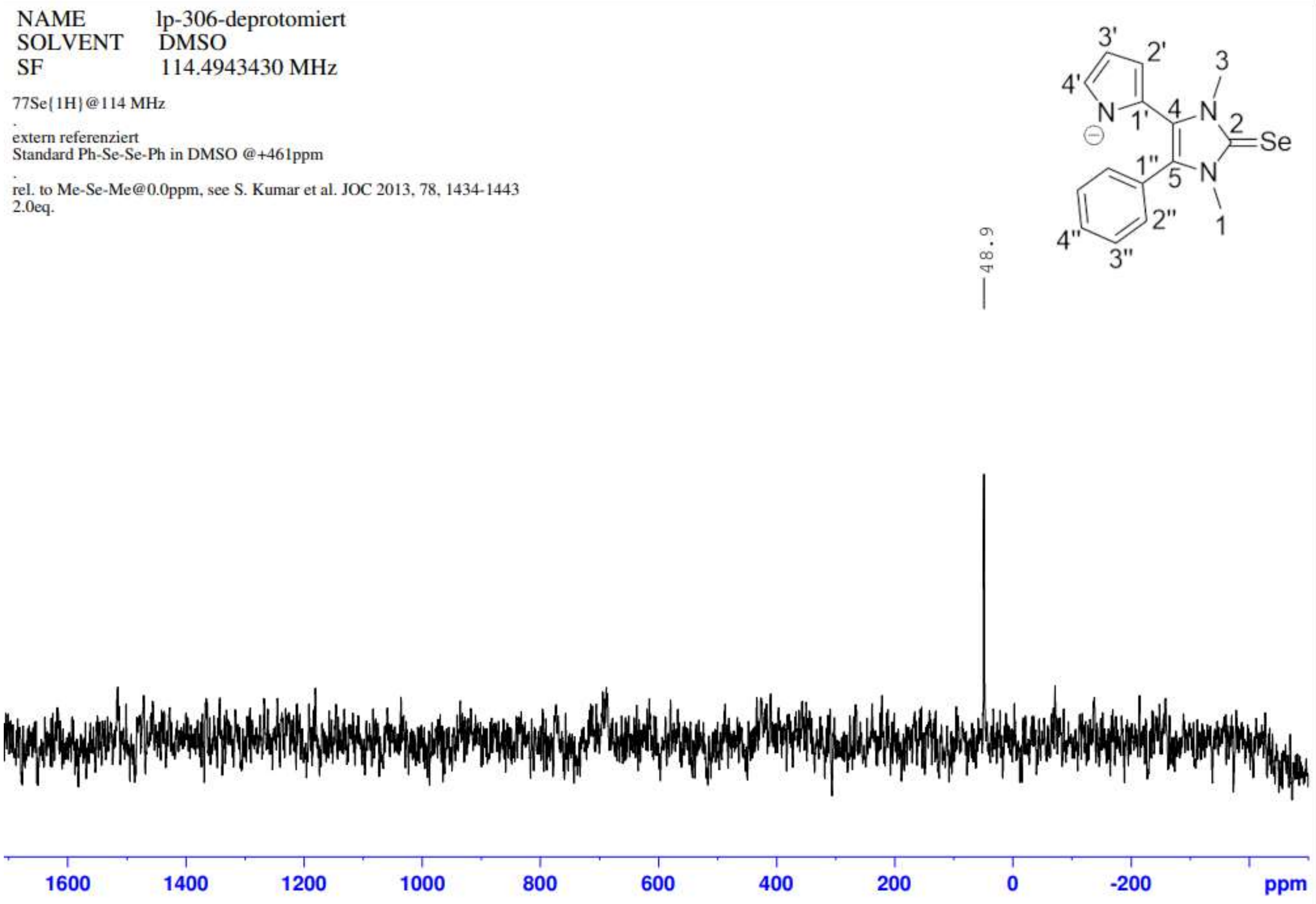

Figure S187. ^{77}Se-NMR of compound 10b

HSQC-NMR 2-(1,3-Dimethyl-5-phenyl-2-selenoxo-2,3-dihydro-1H-imidazol-4-yl)pyrrol-1-ide (10b):

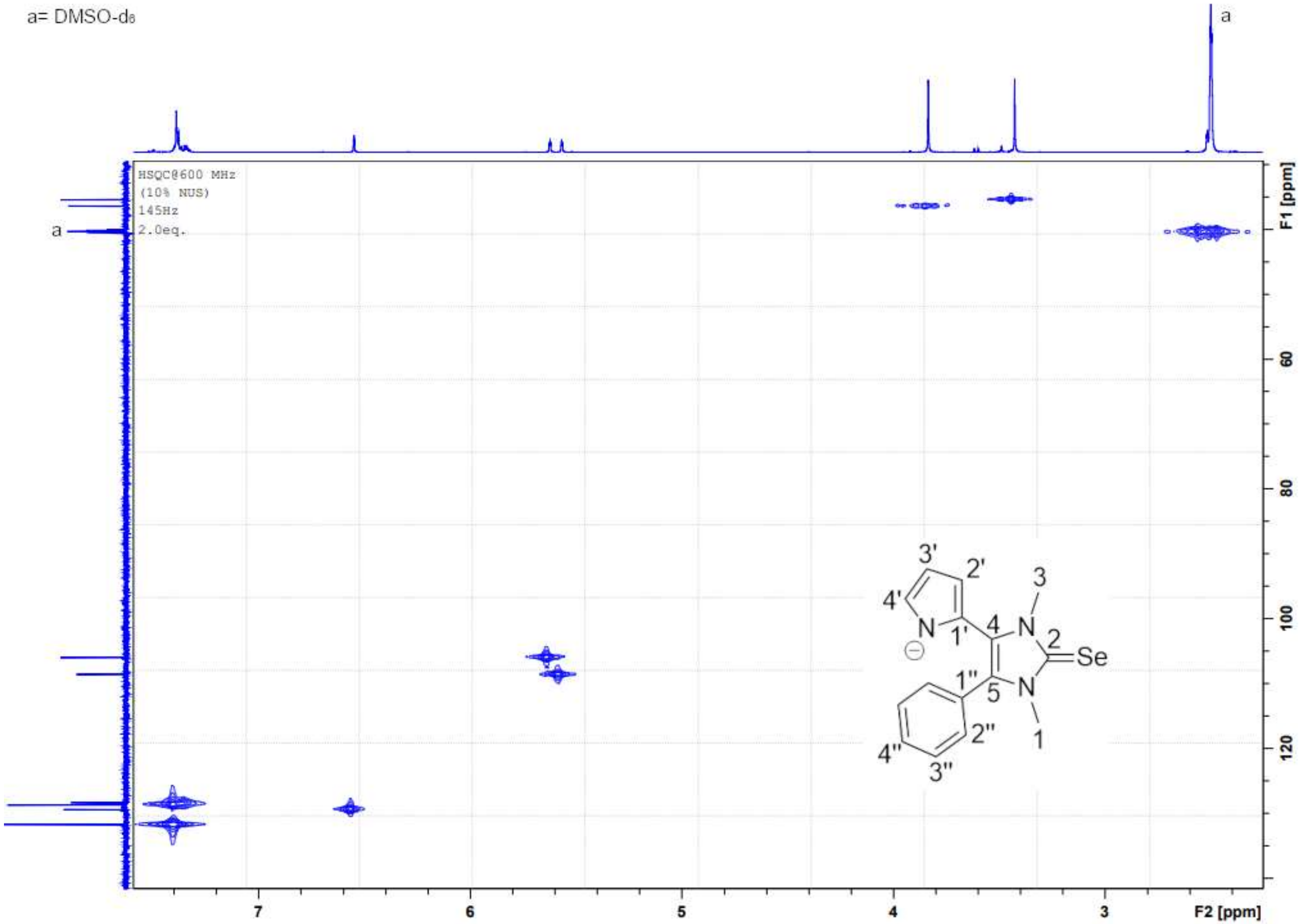

Figure S188. HSQC-NMR of compound 10b

HMBC-NMR 2-(1,3-Dimethyl-5-phenyl-2-selenoxo-2,3-dihydro-1H-imidazol-4-yl)pyrrol-1-ide (10b):

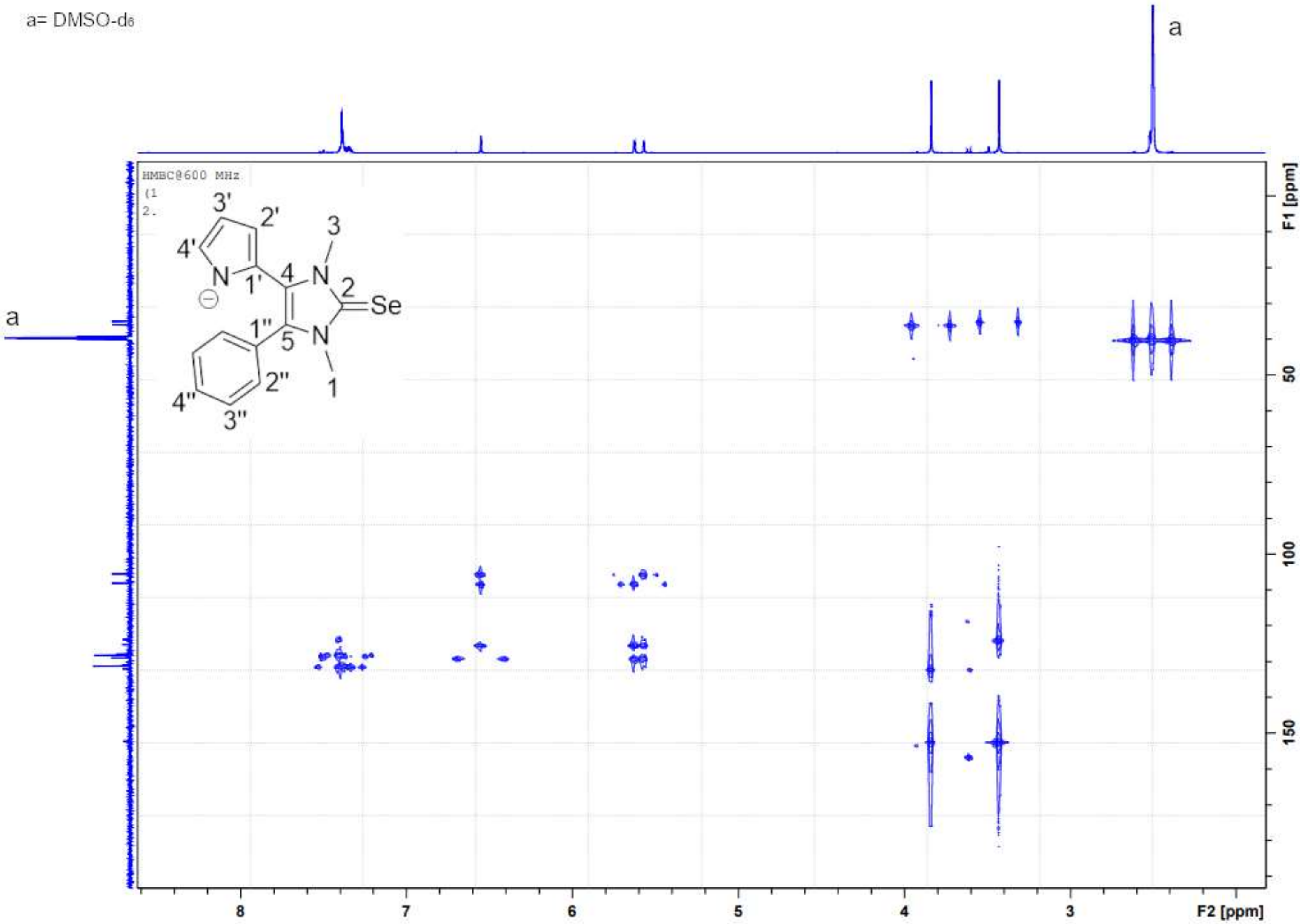

Figure S189. HMBC-NMR of compound 10b

^{1}H-NMR 2-(1,3-Dimethyl-5-phenyl-2-selenoxo-2,3-dihydro-1H-imidazol-4-yl)pyrrol-1-ide (10b) NMR titration:

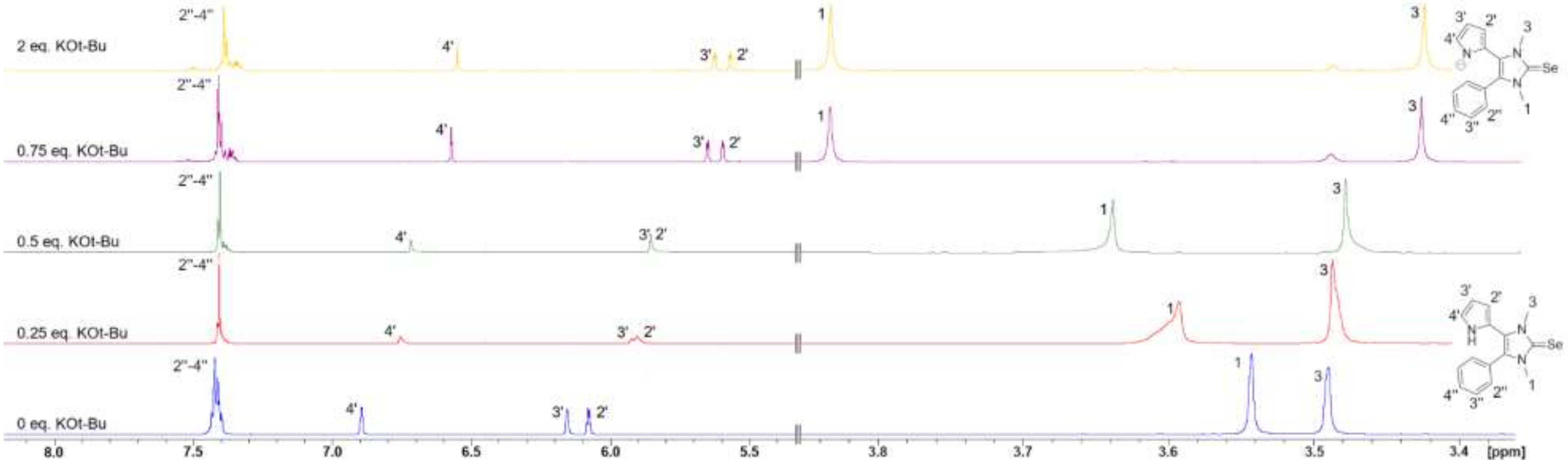

Figure S190. Comparison of the ^{1}H-NMR spectra of 10b after base addition.

^{77}Se-NMR 2-(1,3-Dimethyl-5-phenyl-2-selenoxo-2,3-dihydro-1H-imidazol-4-yl)pyrrol-1-ide (10b) NMR titration:

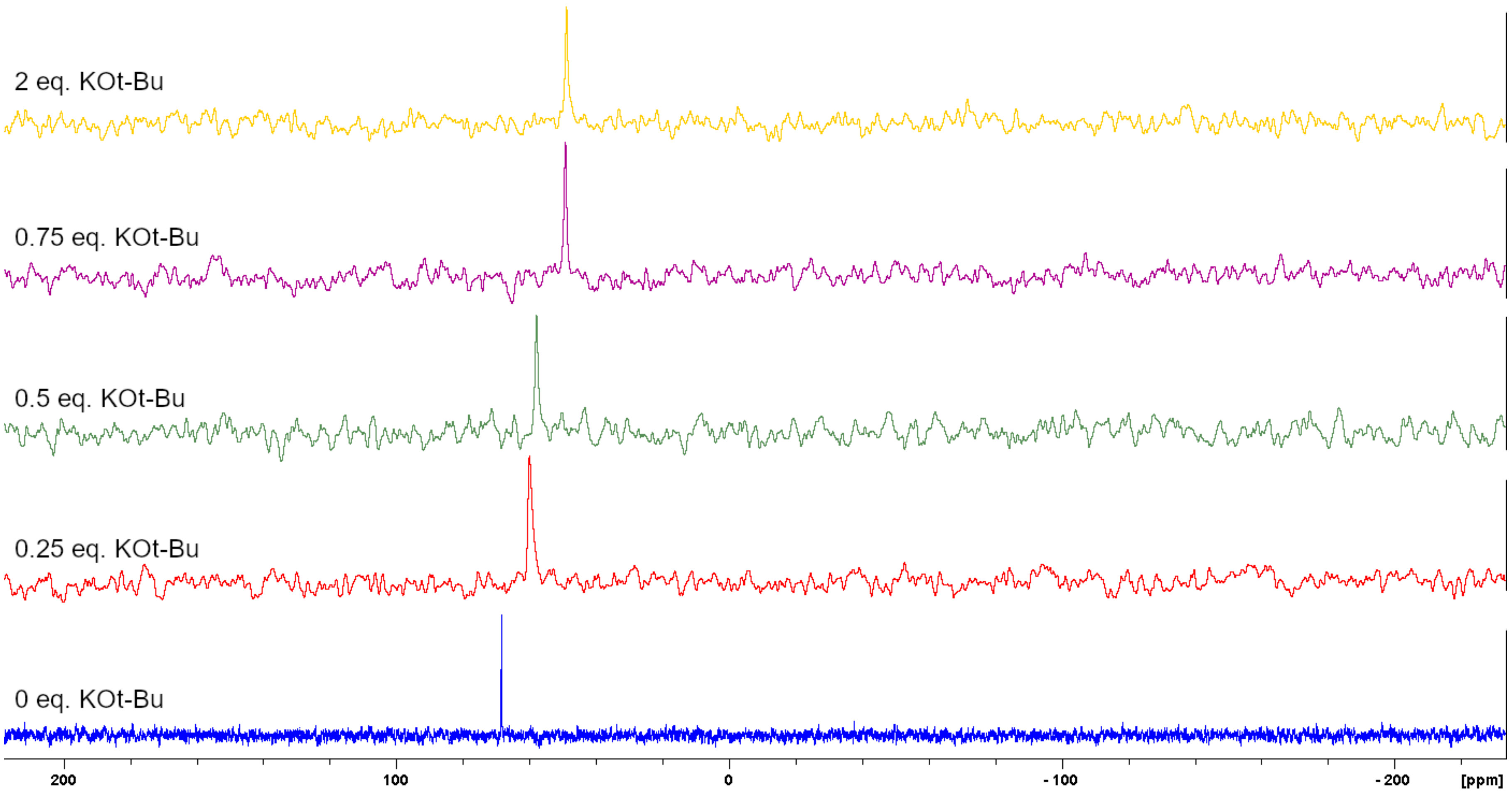

Figure S191. Comparison of the ^{77}Se-NMR spectra of 10b after base addition.

^{1}H-NMR 2-(1-Benzyl-3-methyl-2-selenoxo-2,3-dihydro-1H-imidazol-4-yl)pyrrol-1-ide (10c):

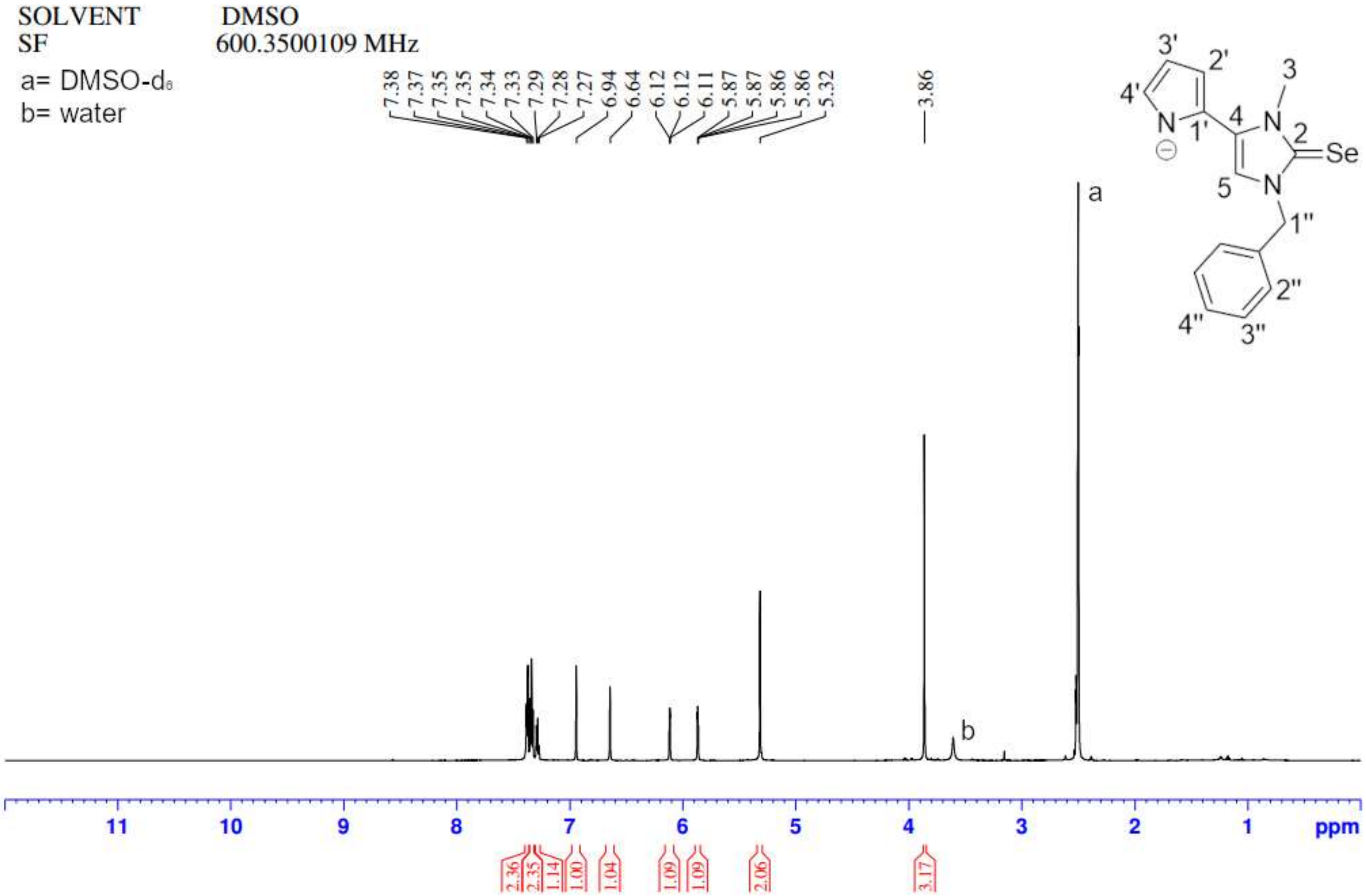

Figure S192. ^{1}H-NMR of compound 10c

$^{13}C\{^1H\}$-NMR 2-(1-Benzyl-3-methyl-2-selenoxo-2,3-dihydro-1H-imidazol-4-yl)pyrrol-1-ide (10c):

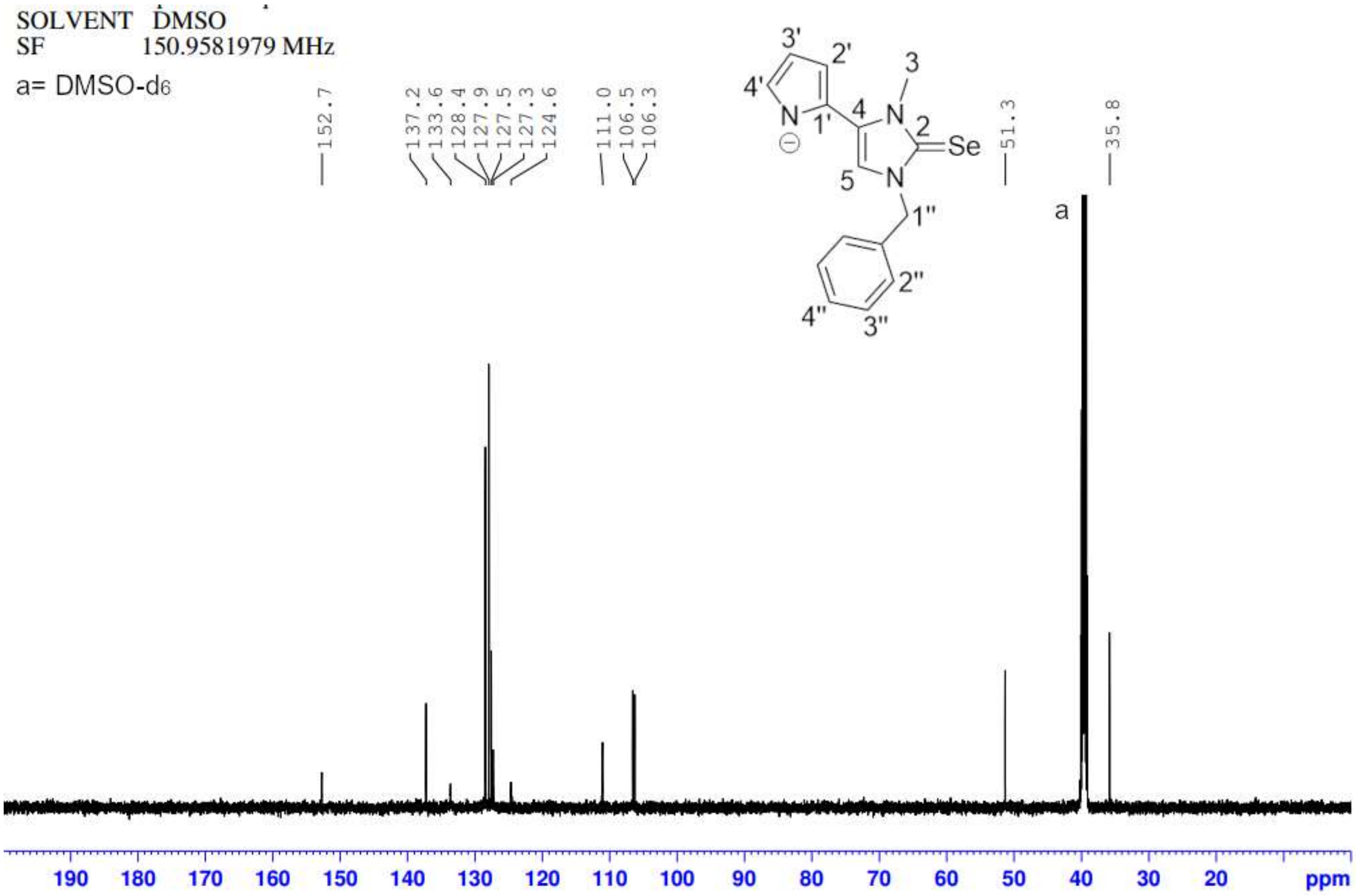

Figure S193. **$^{13}C\{^1H\}$-NMR of compound 10c**

$^{13}C\{^1H\}$-DEPT-NMR 2-(1-Benzyl-3-methyl-2-selenoxo-2,3-dihydro-1H-imidazol-4-yl)pyrrol-1-ide (10e):

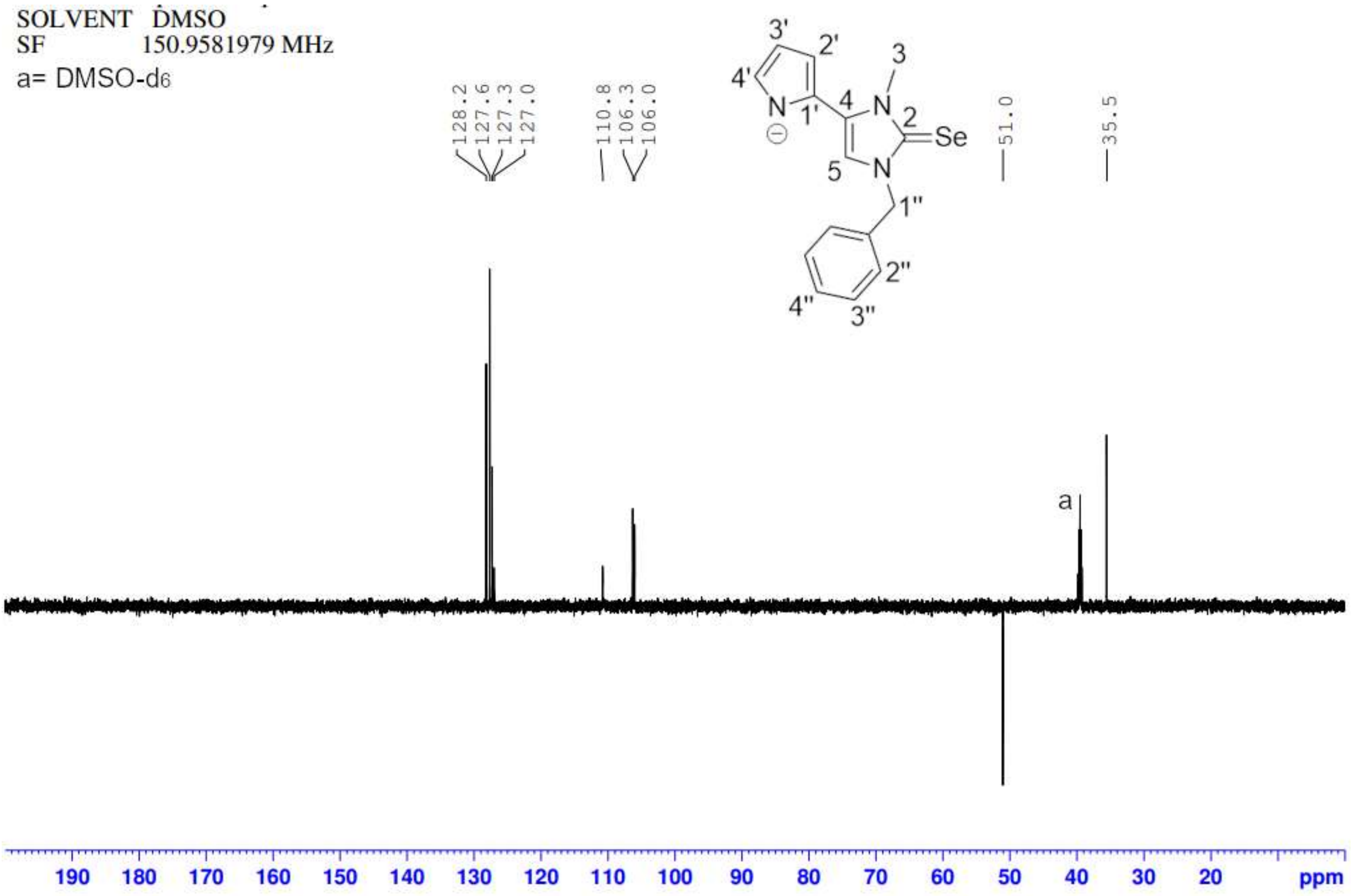

Figure S194. $^{13}C\{^1H\}$**-DEPT-NMR of compound 10c**

^{77}Se-NMR 2-(1-Benzyl-3-methyl-2-selenoxo-2,3-dihydro-1H-imidazol-4-yl)pyrrol-1-ide (10c):

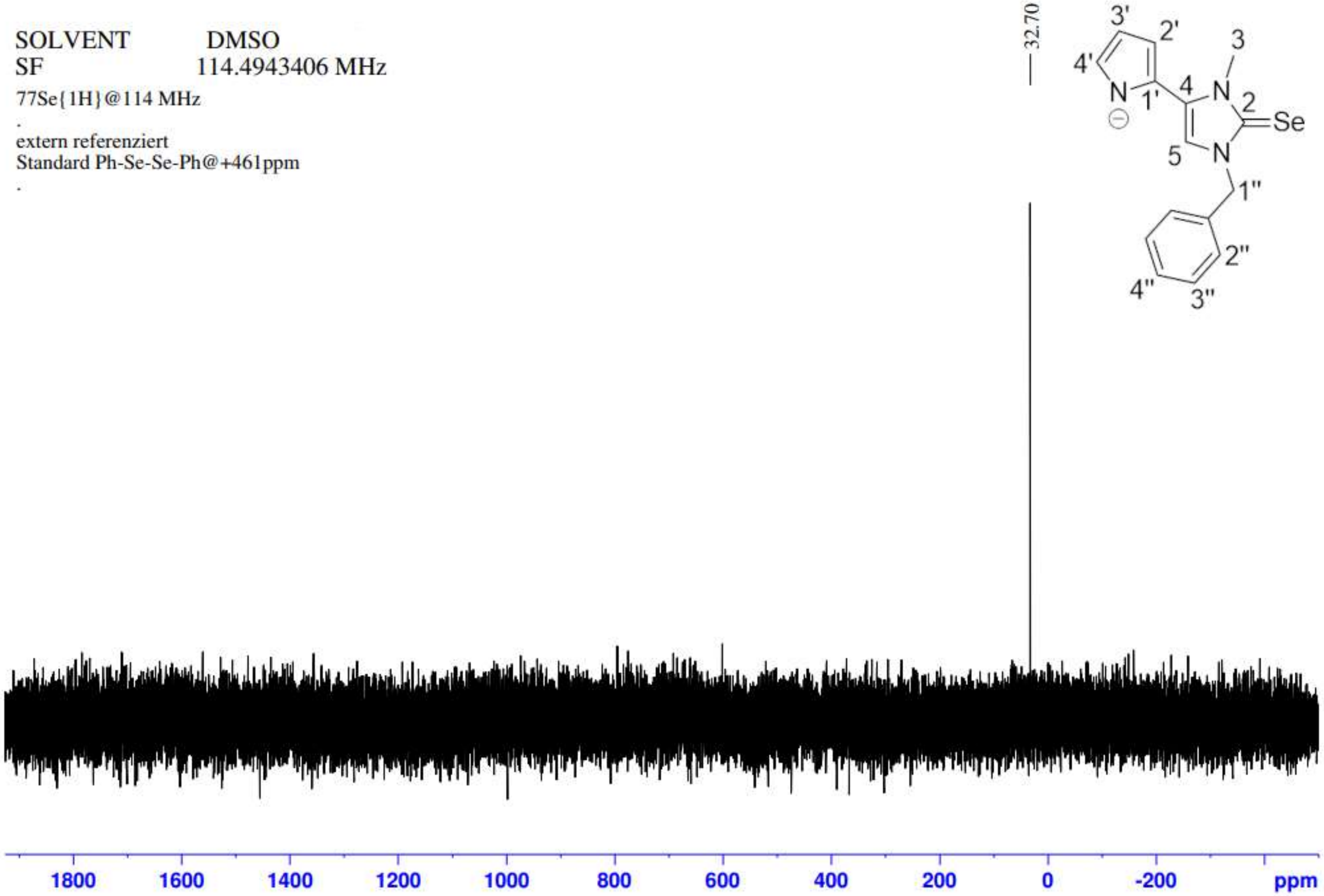

Figure S195. ^{77}Se-NMR of compound 10c

HSQC-NMR 2-(1-Benzyl-3-methyl-2-selenoxo-2,3-dihydro-1H-imidazol-4-yl)pyrrol-1-ide (10c):

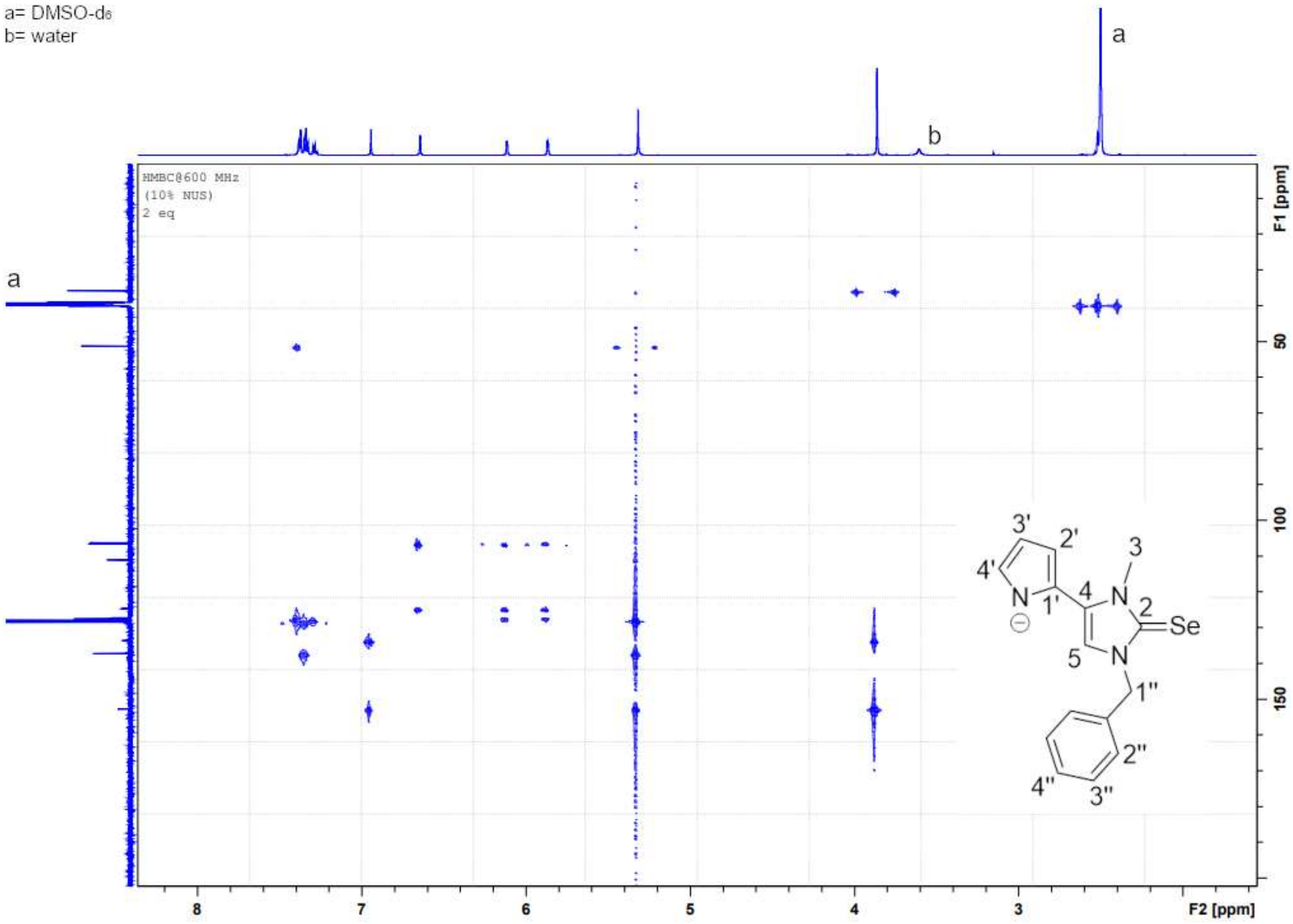

Figure S196. HSQC-NMR of compound 10c

HMBC-NMR 2-(1-Benzyl-3-methyl-2-selenoxo-2,3-dihydro-1H-imidazol-4-yl)pyrrol-1-ide (10c):

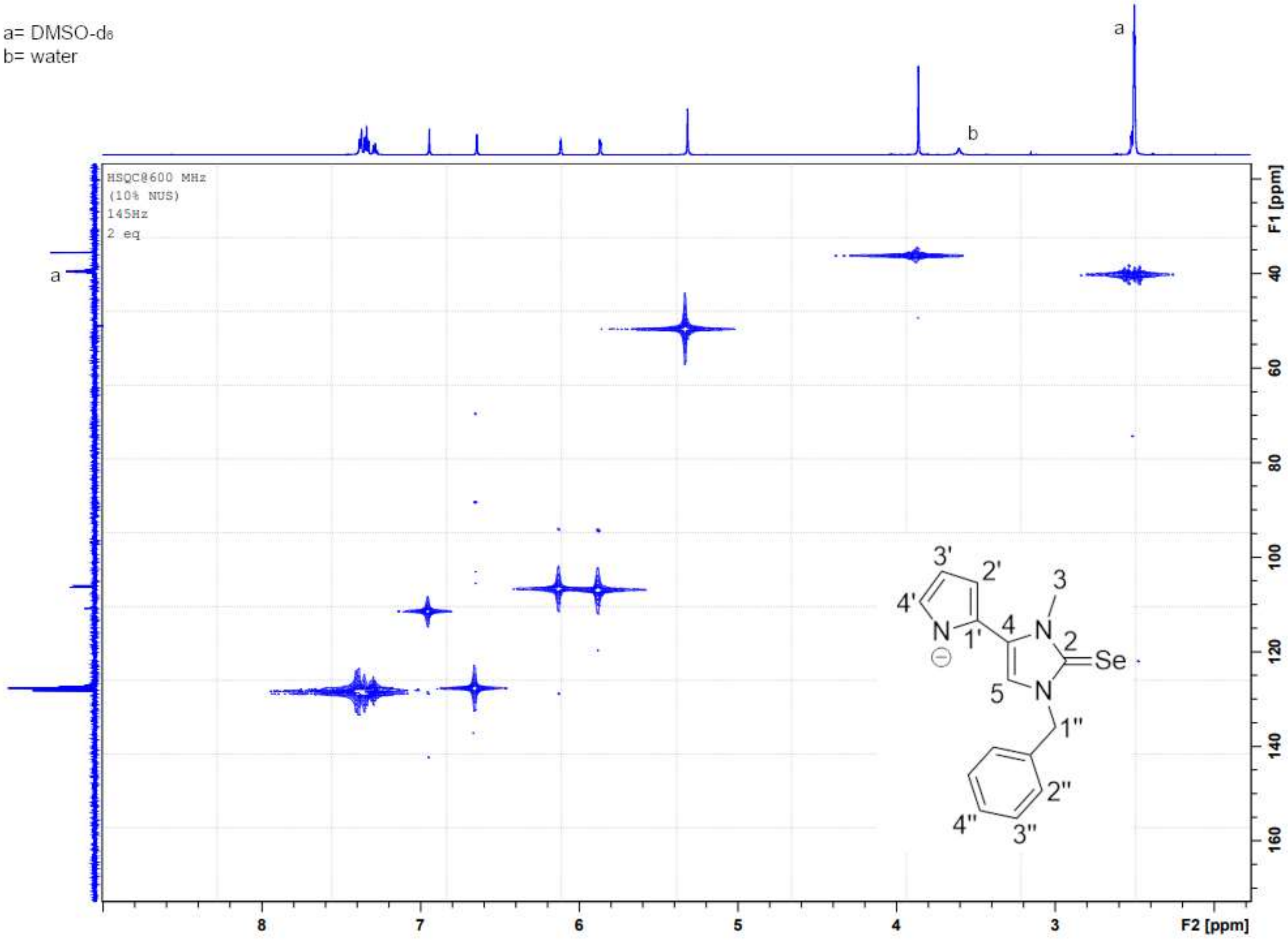

Figure S197. HMBC-NMR of compound 10c

^{1}H-NMR 2-(1-Benzyl-3-methyl-2-selenoxo-2,3-dihydro-1H-imidazol-4-yl)pyrrol-1-ide (10c) NMR titration:

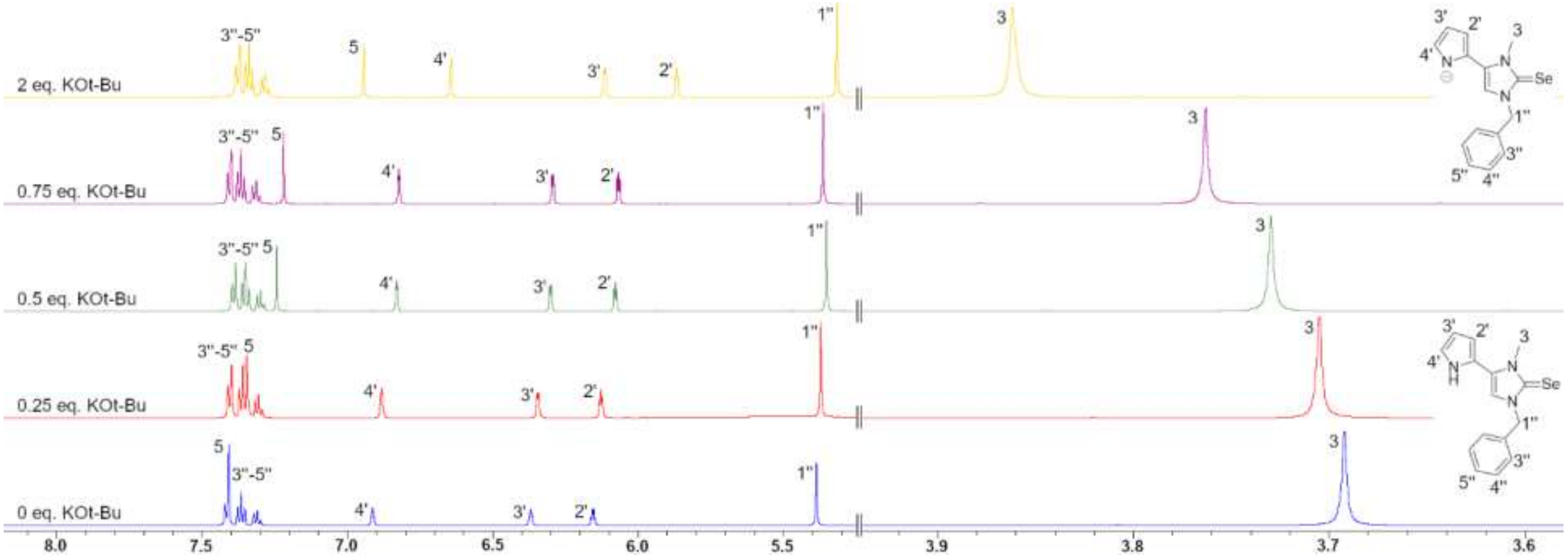

Figure S198. Comparison of the ^{1}H-NMR spectra of 10c after base addition.

^{77}Se-NMR 2-(1-Benzyl-3-methyl-2-selenoxo-2,3-dihydro-1H-imidazol-4-yl)pyrrol-1-ide (10c) NMR titration:

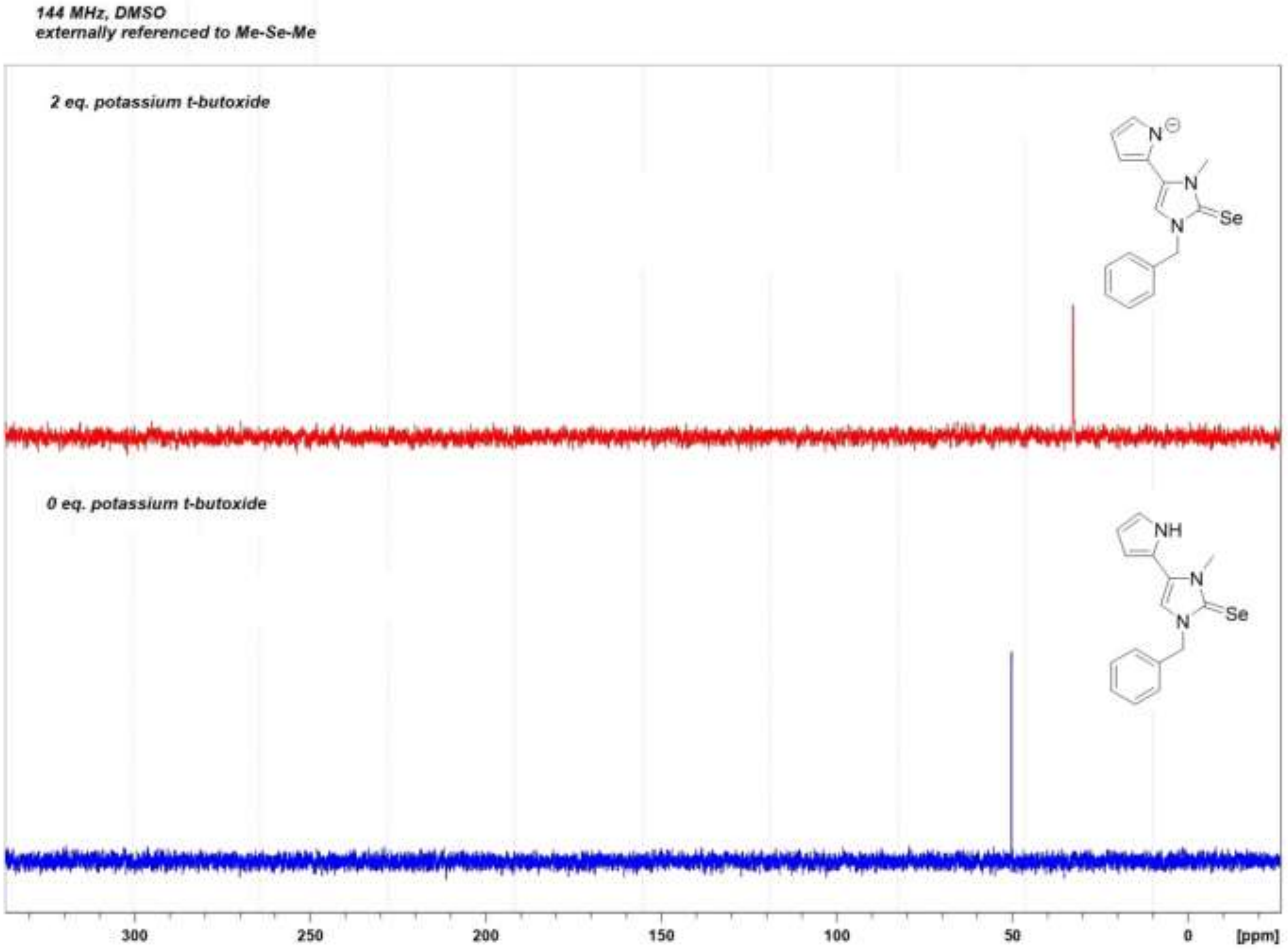

Figure S199. Comparison of the ^{77}Se-NMR spectra of 10c after base addition.

^{77}Se -NMR 1-Benzyl-3-(*tert*-butyl)-4-(1H-pyrrol-2-yl)-1,3-dihydro-2H-imidazole-2-selenone (9h) temperature measurement externally referenced:

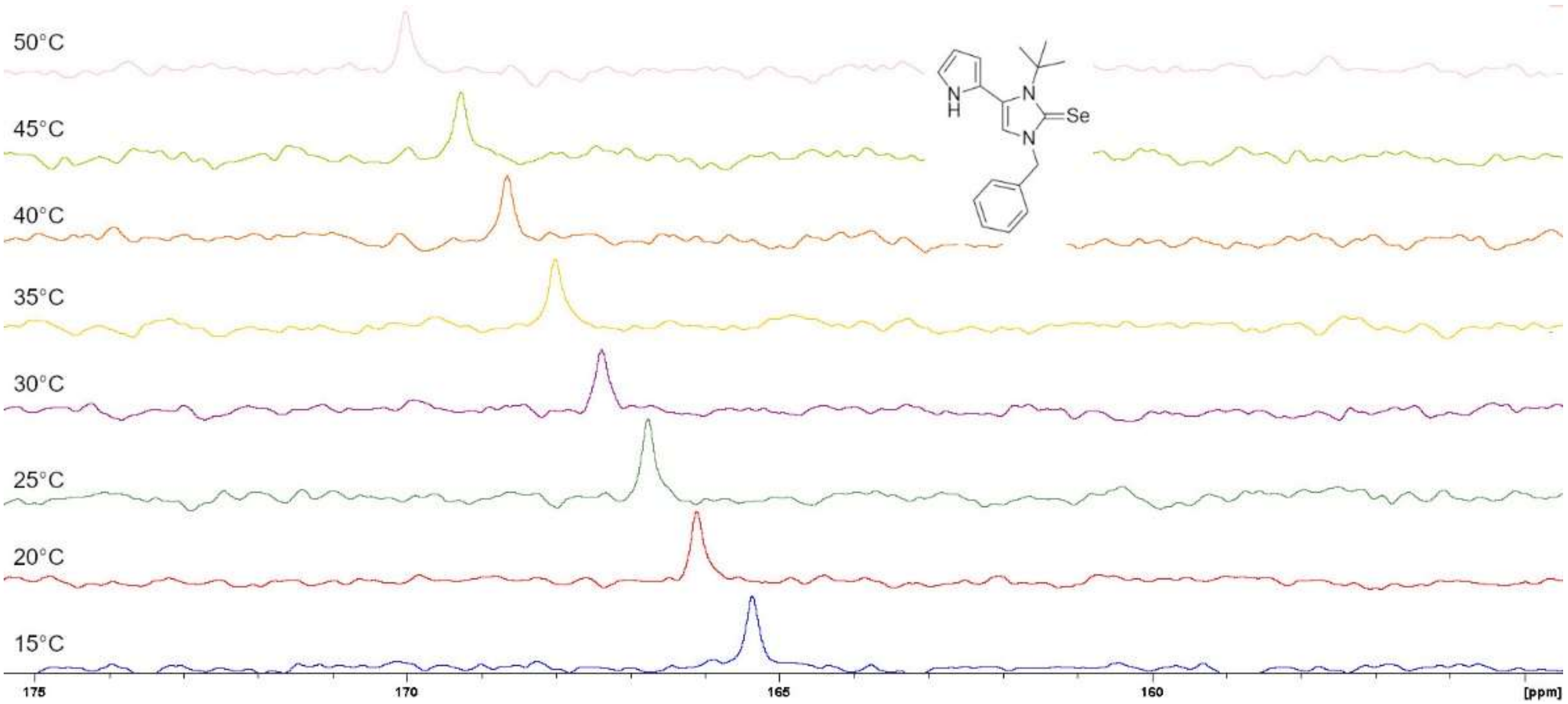

Figure S200. Comparison of the ^{77}Se-NMR spectra of 10h depending on the temperature between 15°C and 50°C. Referenced to PH-Se-Se-Ph at 461 ppm in CD_3CN against Me_2Se at 0.0 ppm

^{77}Se -NMR 1-Benzyl-3-(*tert*-butyl)-4-(1H-pyrrol-2-yl)-1,3-dihydro-2H-imidazole-2-selenone (9h) temperature measurement internally referenced:

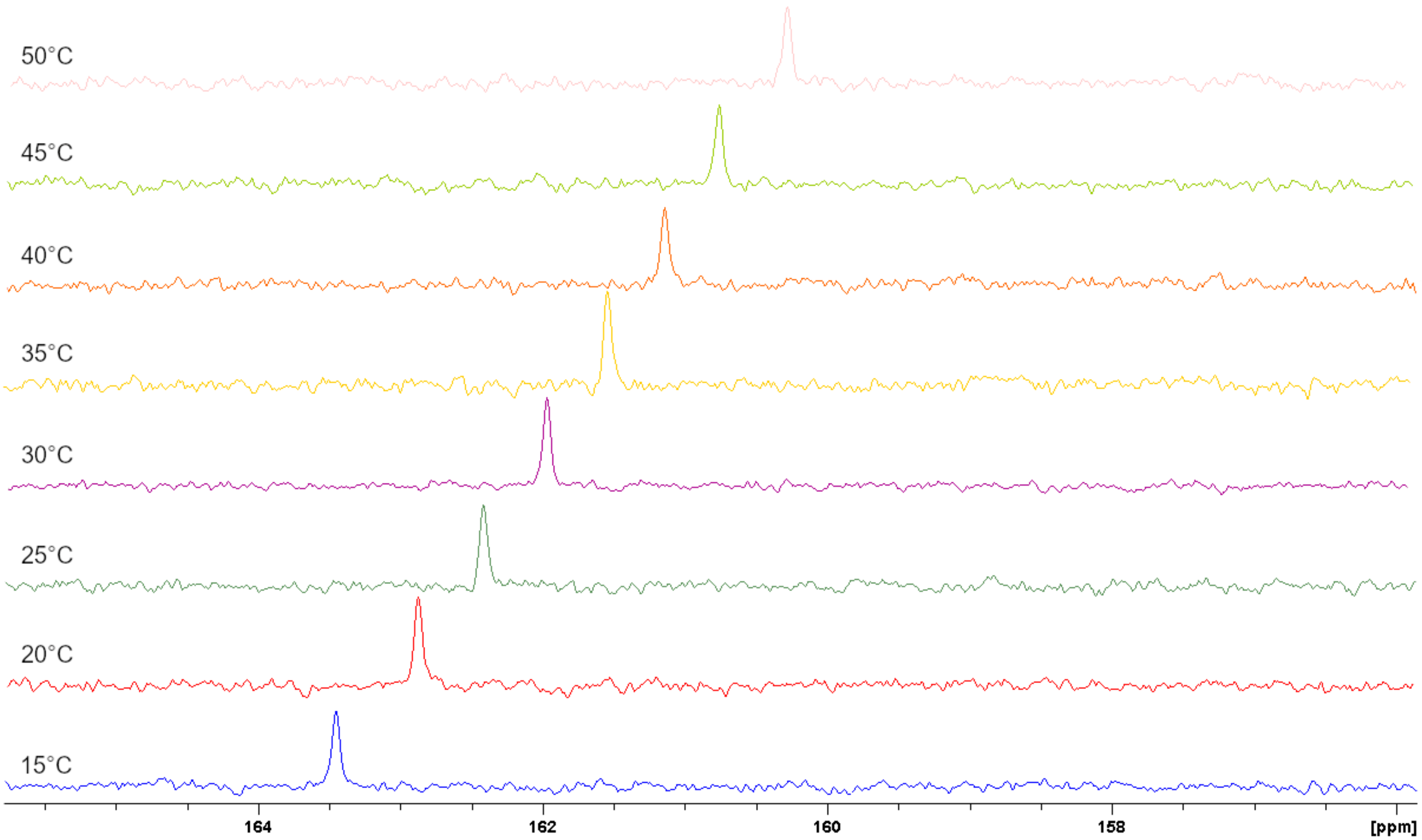

Figure S201. Comparison of the ^{77}Se-NMR spectra of 10h depending on the temperature between 15°C and 50°C. Referenced internally to PH-Se-Se-Ph at 461 ppm.

^{77}Se -NMR Diphenyl diselenide temperature measurement externally referenced:

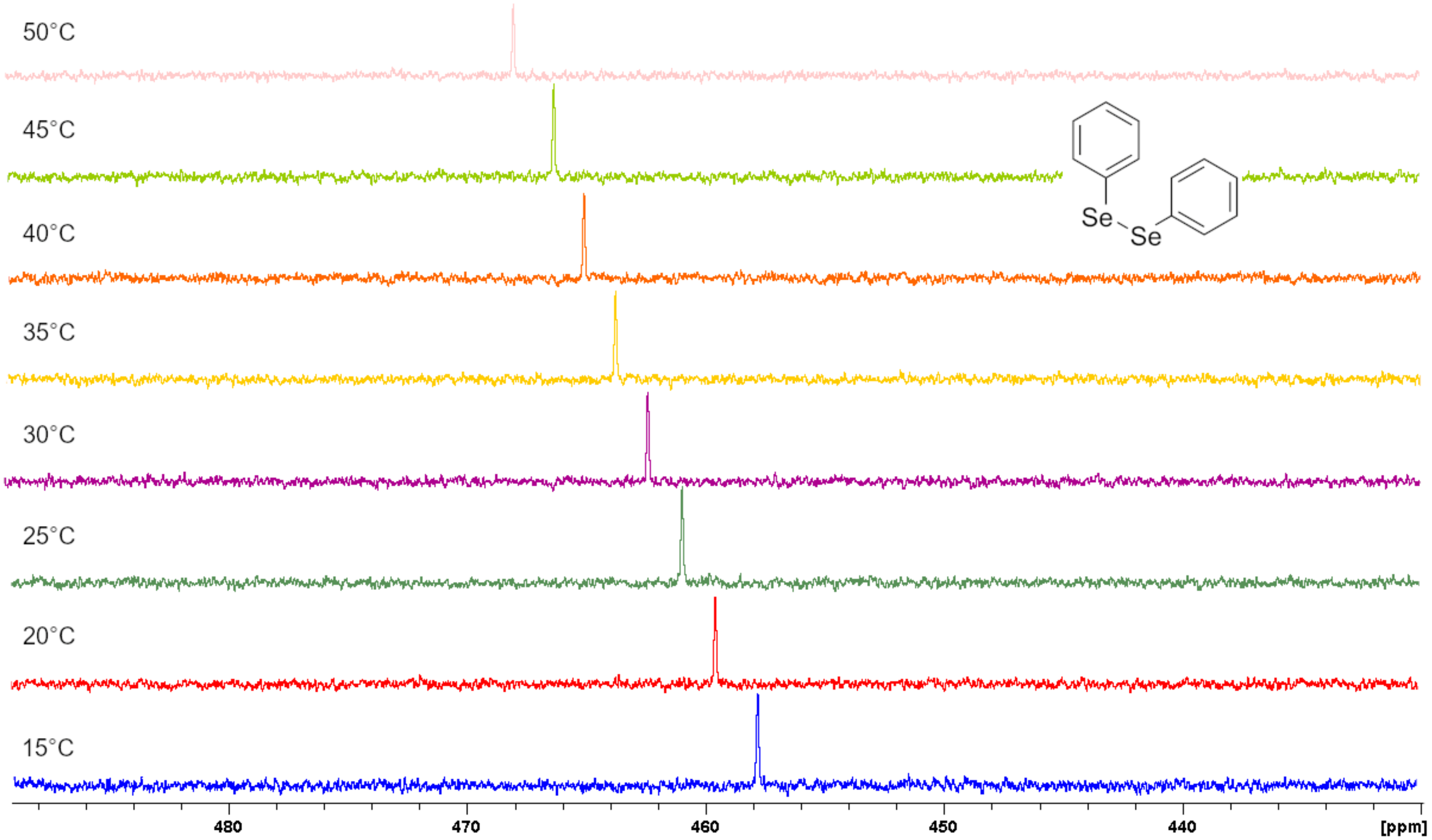

Figure S202. Comparison of the ^{77}Se-NMR spectra of Diphenyl diselenide depending on the temperature between 15°C and 50°C. Referenced to PH-Se-Se-Ph at 461 ppm in CD_3CN @25°C.

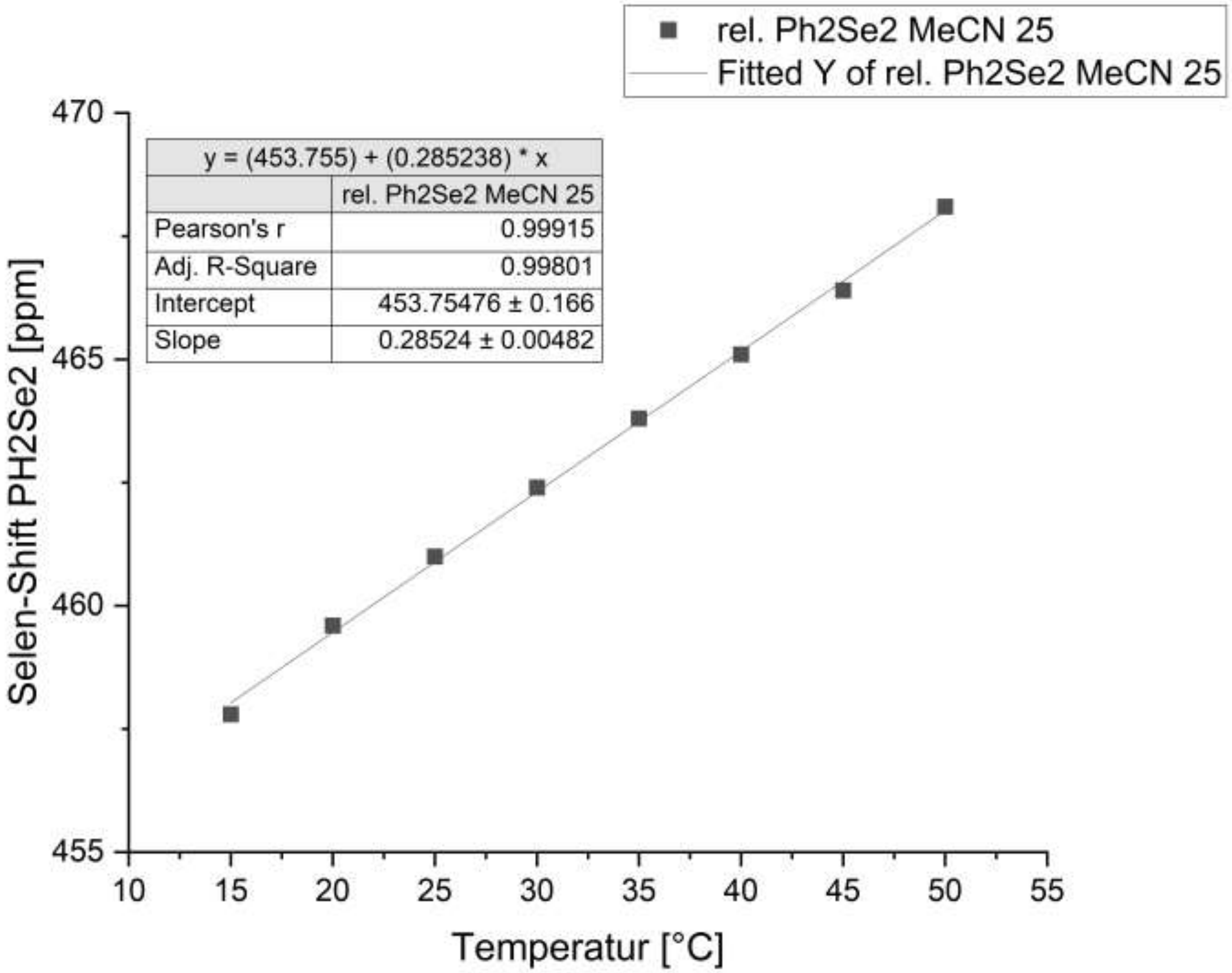

Figure S203. Temperature dependence of Ph_2Se_2 in CD_3CN externally referenced to Ph_2Se_2 in CD_3CN @ 25°C @461ppm

^{77}Se -NMR of 9h in CD_3CNinternally and externally referenced to Ph_2Se_2:

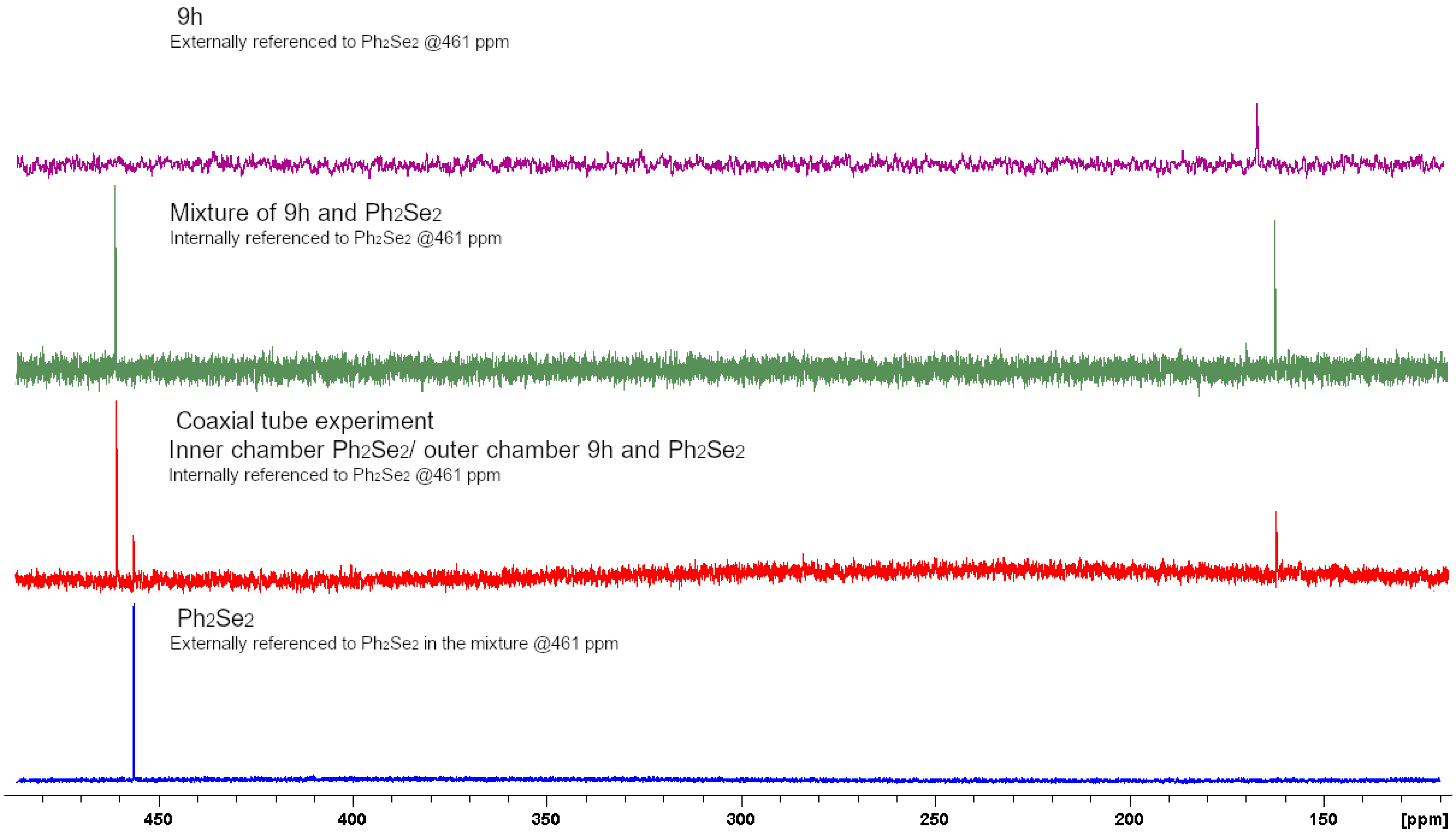

Figure S204. Referencing experiments for 9h in CD_3CN

^{77}Se -NMR of 9h and 9e in $CDCl_3$ internally and externally referenced to Ph_2Se_2:

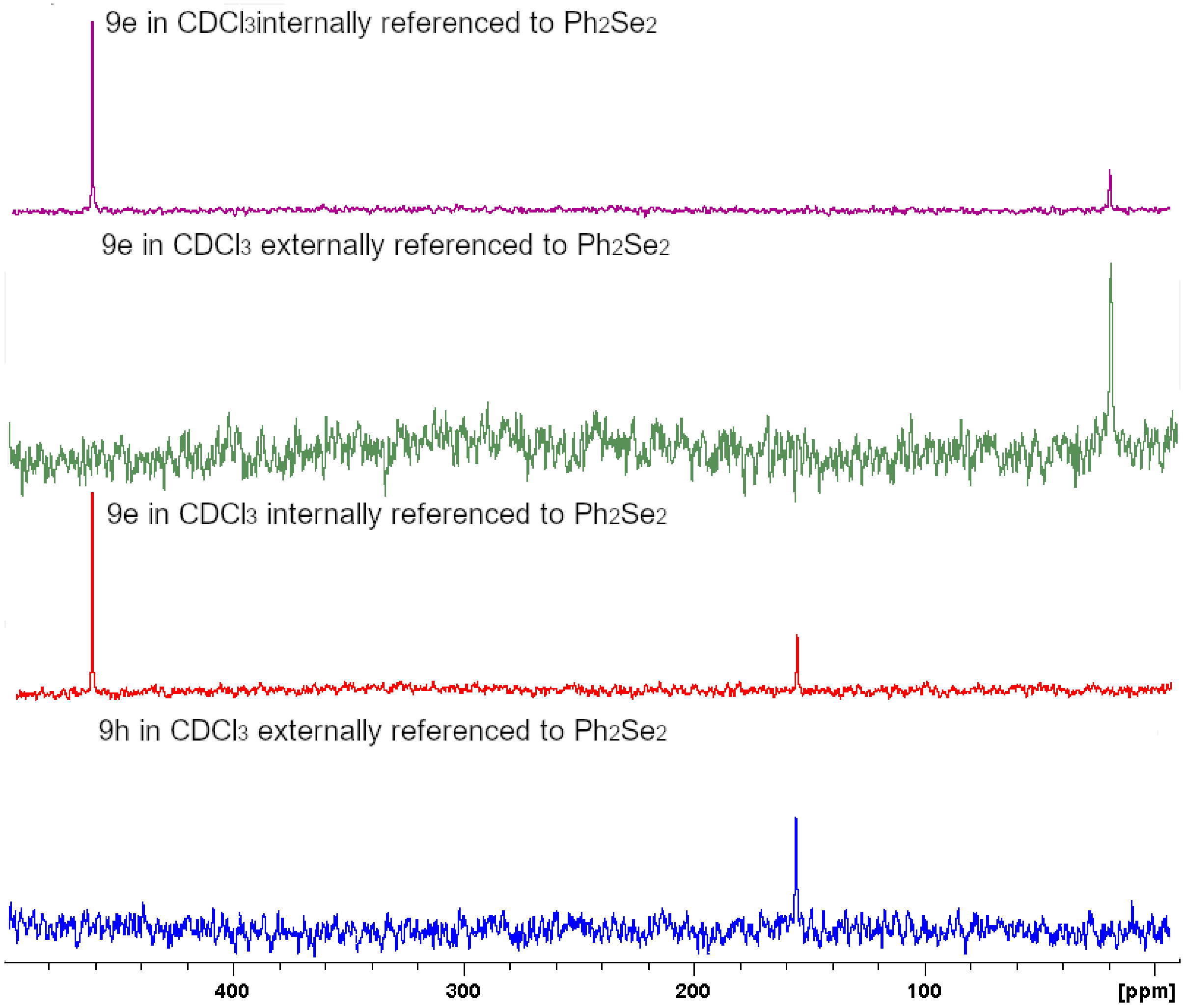

Figure S205. **^{77}Se -NMR of 9h and 9e in $CDCl_3$ internally and externally referenced to Ph_2Se_2**

DFT calculations (HOMO/LUMO coefficients and orbital energies, CREF value)

HOMO-LUMO DFT calculations were performed using the Spartan Software (*Spartan'20*, Wavefunction, Inc., Irvine, CA. Available from: http://www.wavefun.com) running on a MS Windows 10 Pro PC system with an AMD Ryzen Threadripper 3970X 32-Core and 128 GB RAM. MMFF optimized structures were used as starting geometries for the geometry optimizations with the B3LYP density functional and the 6-311++G(d,p) basis set carried out as vacuum calculations. Subsequent frequency calculations of all final structures evidenced the absence of imaginary frequencies and thus the presence of true minima on the potential energy surface. Based on the DFT calculations, the CREF values were calculated in accordance with the literature[6a-c].

2-(1,3-Dimethyl-1H-imidazol-3-ium-5-yl)pyrrol-1-ide (8a), precursor:

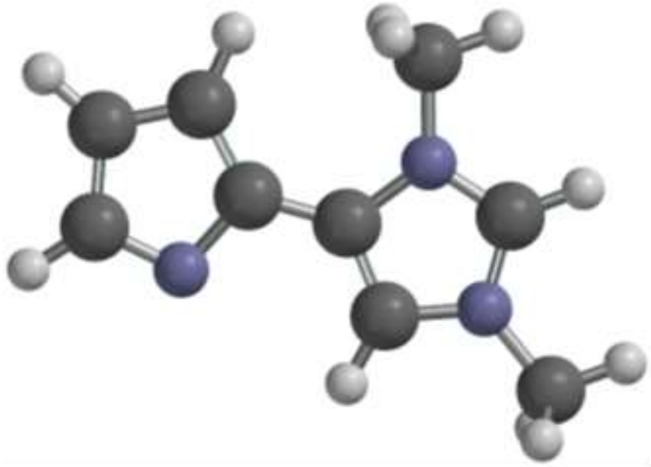

Charge: 0

Number of imaginary frequencies: 0

E: -513.9086695563 ha

Table S2. HOMO/LUMO profiles and energies of 8a (precursor)

LUMO 8a	HOMO 8a
LUMO+2 (-0.45 eV)	HOMO (-4.32 eV)
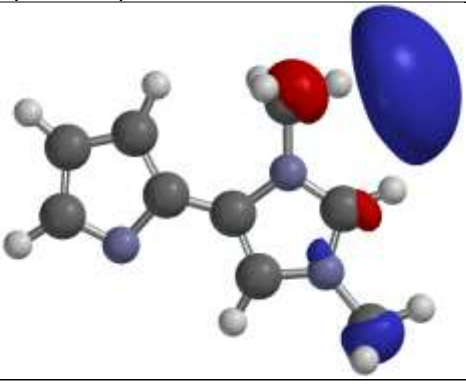	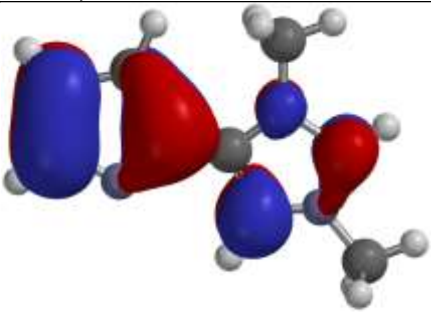
LUMO+1 (-0.90 eV)	HOMO-1 (-5.10 eV)

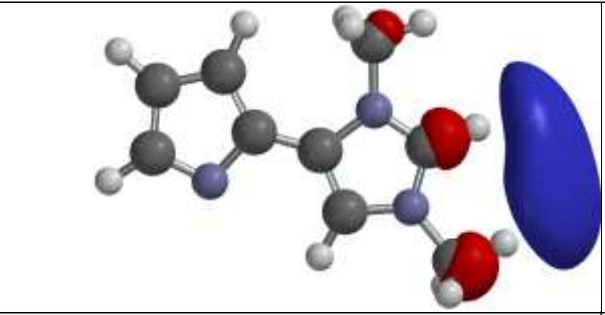	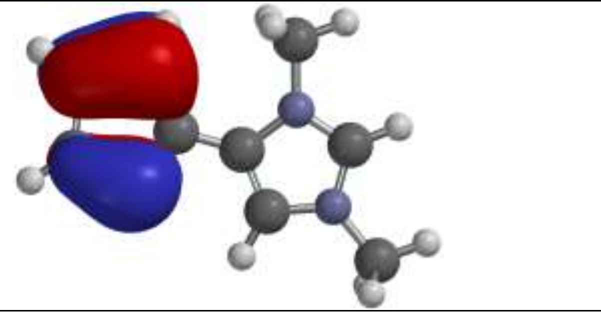
LUMO (-1.21 eV)	HOMO-2 (-5.96 eV)
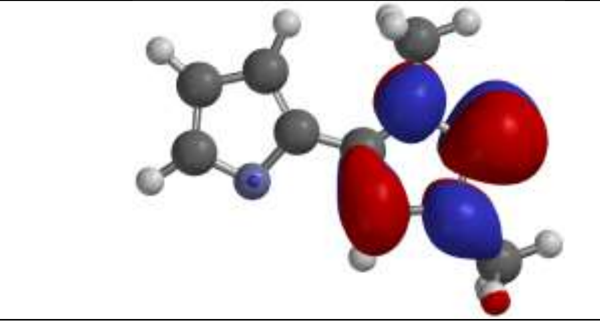	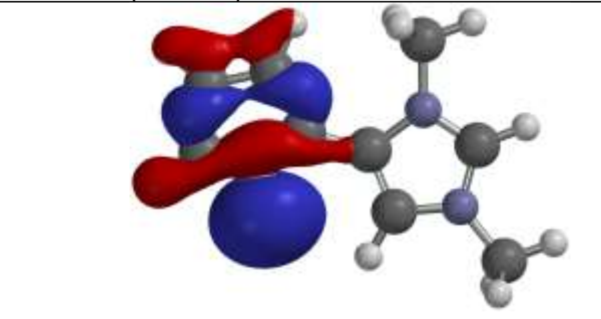
	HOMO-3 (-7.41 eV)
	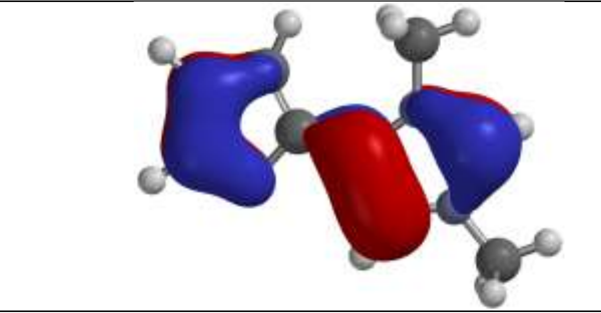
	HOMO-4 (-8.76 eV)
	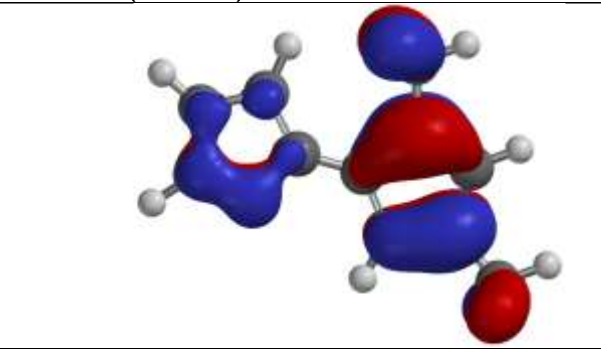

2-(1,3-Dimethyl-1H-imidazol-3-ium-5-yl)pyrrol-1-ide , anionic carbene from 8a:

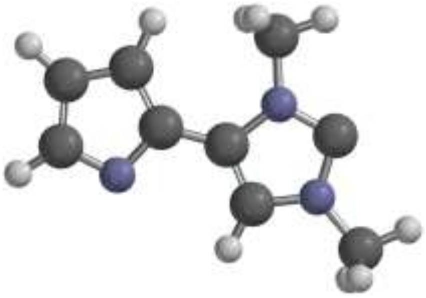

Charge: -1

Number of imaginary frequencies: 0

E: -513.3490343517 ha

Table S3. HOMO/LUMO profiles and energies of 8a (carbene)

**LUMO, anionic carbene form 8a**	**HOMO anionic carbene form 8a**
LUMO+2 (2.90 eV)	HOMO (-0.60 eV)
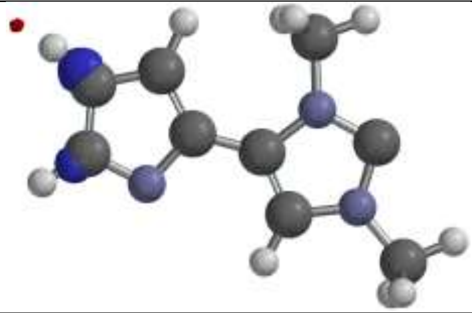	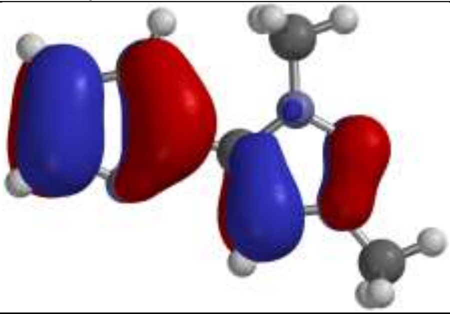
LUMO+1 (2.60 eV)	HOMO-1 (-1.59 eV)
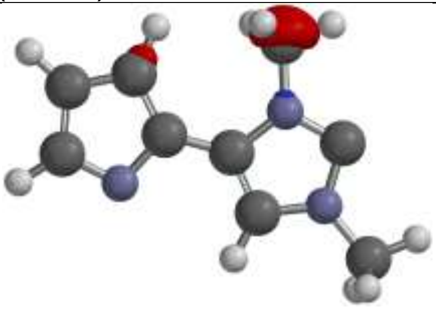	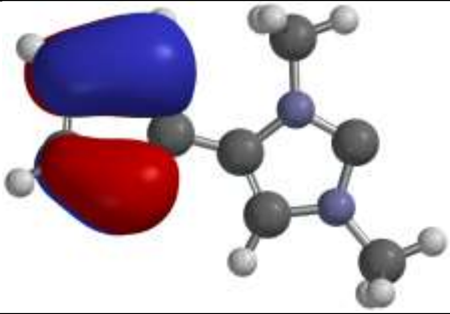
LUMO (2.21 eV)	HOMO-2 (-2.41 eV)
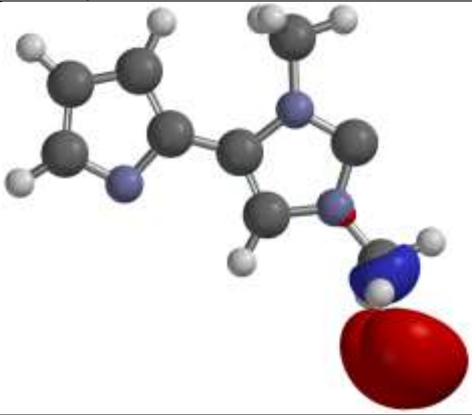	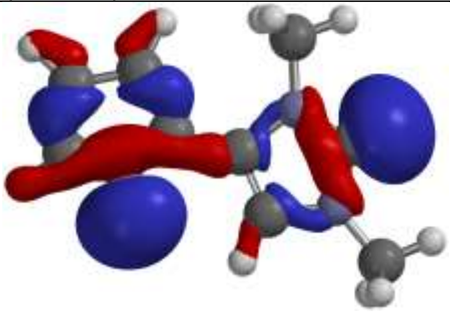

	HOMO-3 (-2.58 eV)
	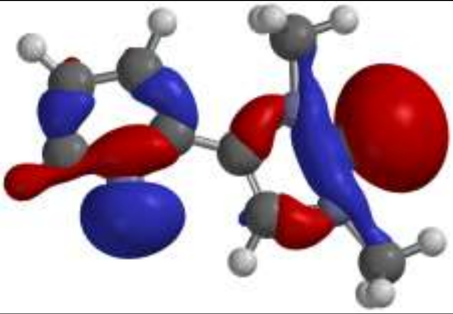
	HOMO-4 (-2.87 eV)
	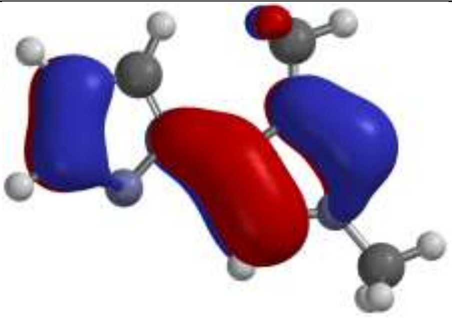

Calculated CREF value

Precursor **8a**:

Electronic energy:	-513.90866956 ha
Zero point energy:	0.18835732 ha
Sum of electronic and ZPE:	-513.72031224 ha

Corresponding carbine **7a**:

Electronic energy:	-513.34903435 ha
Zero point energy:	0.17487067 ha
Sum of electronic and ZPE:	-513.17416369 ha

Resulting CREF value: **0.546**

1,3-Dimethyl-5-phenyl-4-(1H-pyrrol-2-yl)-1H-imidazol-3-ium (8b), precursor:

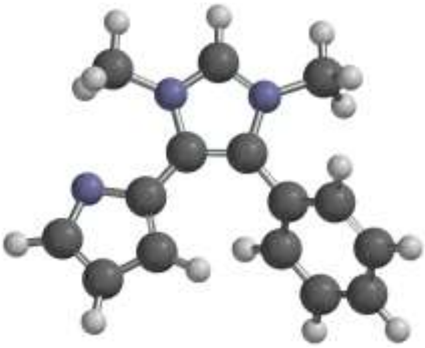

Charge: 0

Number of imaginary frequencies: 0

E: - 745.0215234475 hartrees

Table S4. HOMO/LUMO profiles and energies of 8b (precursor)

LUMO 8b	HOMO 8b
LUMO+2 (-0.81 eV)	HOMO (-4.27 eV)
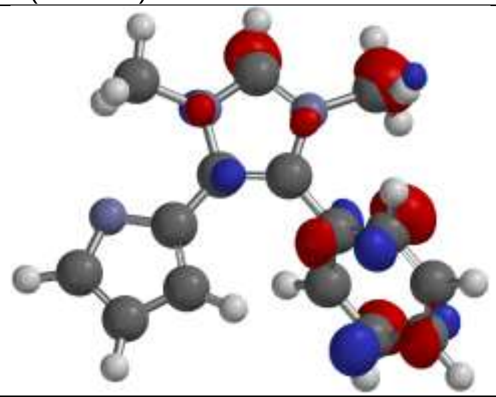	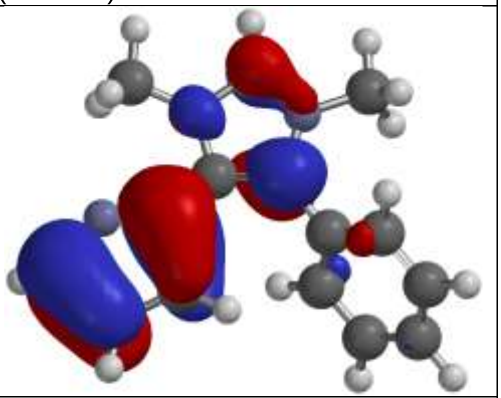
LUMO+1 (-0.88 eV)	HOMO-1 (-5.07 eV)
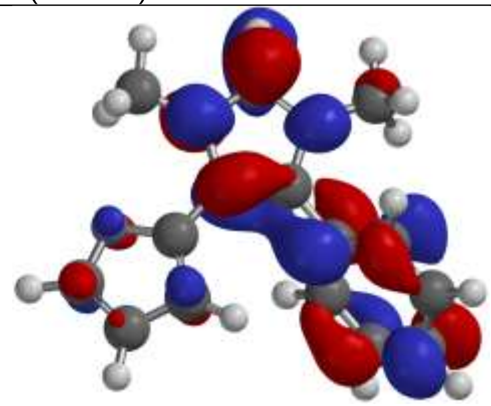	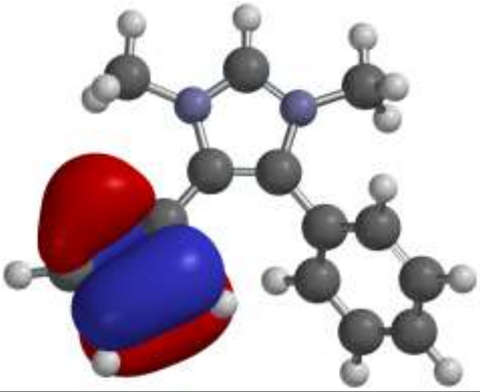
LUMO (-1.29 eV)	HOMO-2 (-6.00 eV)
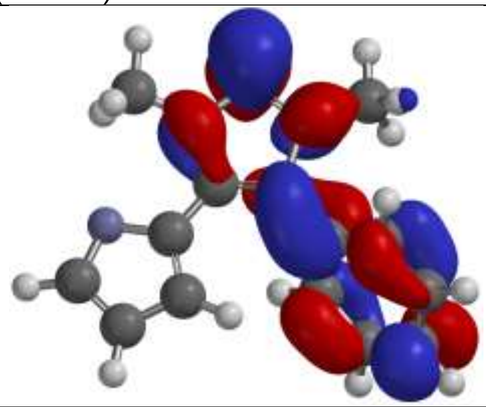	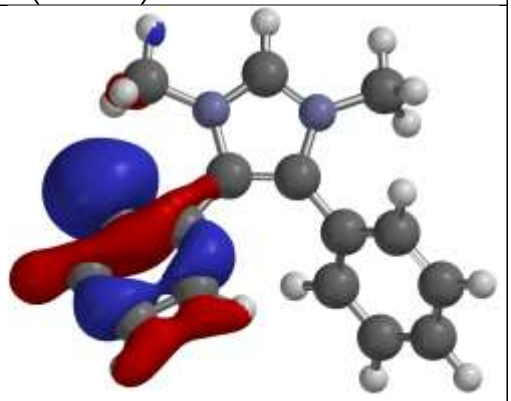

	HOMO-3 (-6.94 eV)
	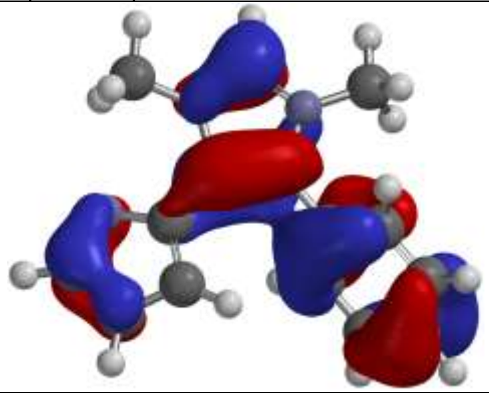
	HOMO-4 (-7.32 eV)
	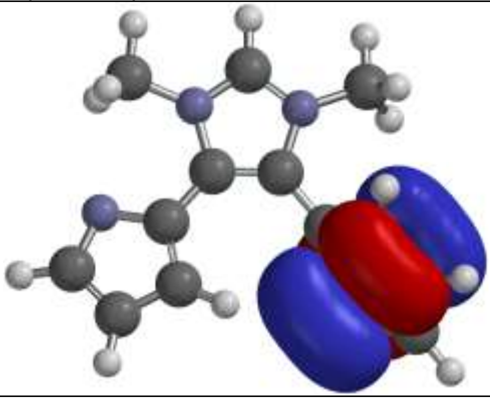

1,3-Dimethyl-5-phenyl-4-(1H-pyrrol-2-yl)-1H-imidazol-3-ium, anionic carbene from 8b:

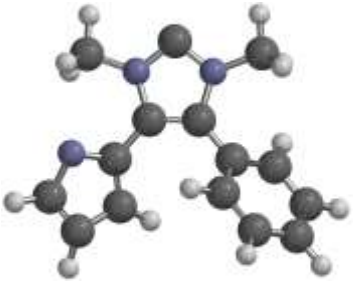

Charge: -1

Number of imaginary frequencies: 0

E: -744.4626852049 hartrees

Table S5. HOMO/LUMO profiles and energies of 8b (carbene)

LUMO, anionic carbene form 8b	HOMO anionic carbene form 8b
LUMO+2 (2.24 eV)	HOMO (-0.91 eV)
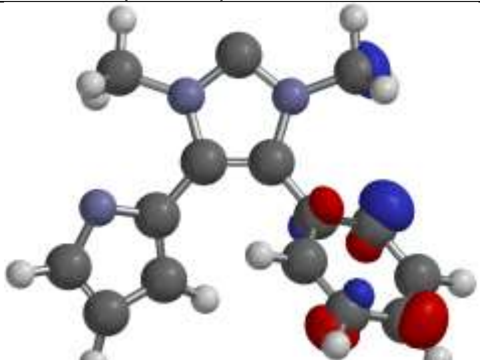	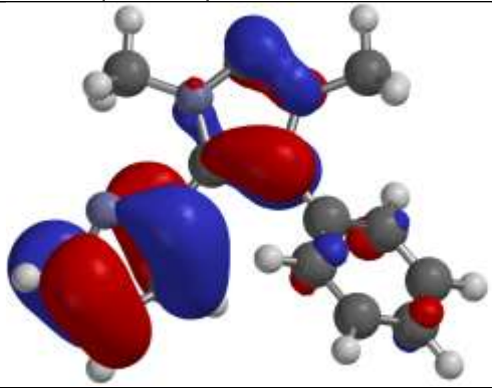
LUMO+1 (2.06 eV)	HOMO-1 (-1.82 eV)

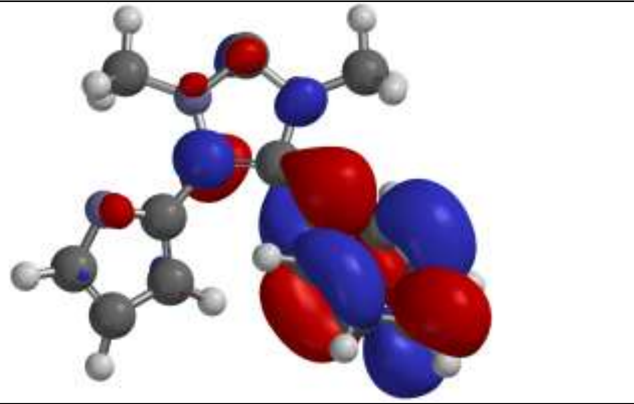	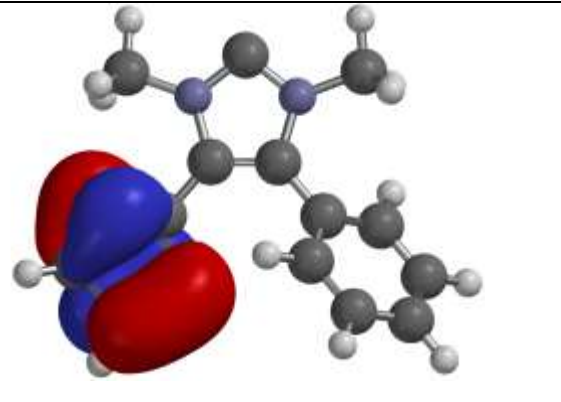
LUMO (185 eV)	HOMO-2 (-2.58 eV)
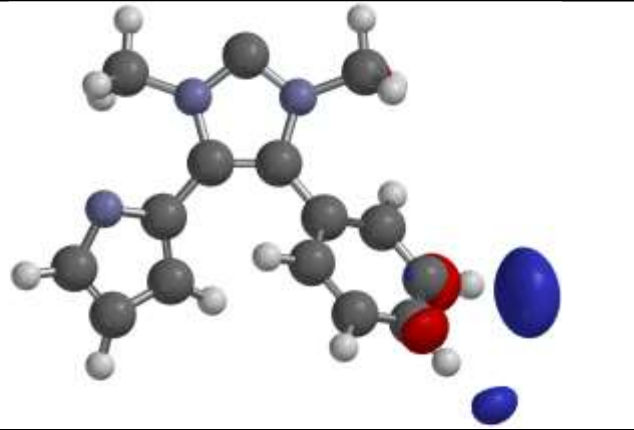	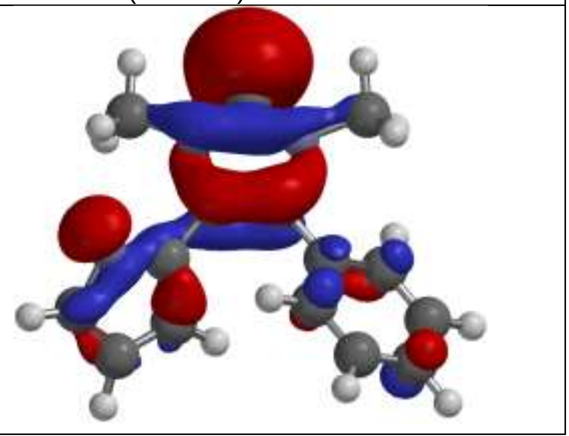

	HOMO-3 (-2.73 eV)
	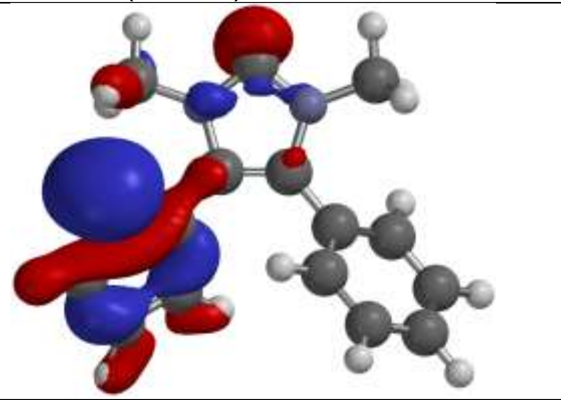
	HOMO-4 (-2.90 eV)

	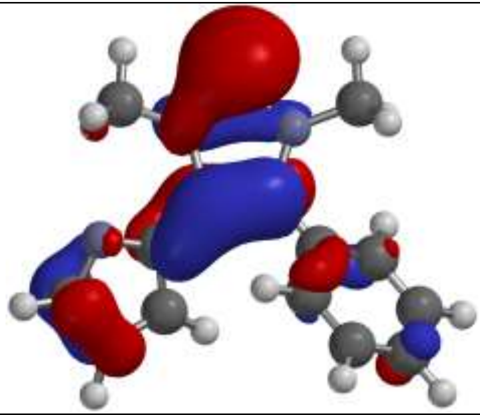

Calculated CREF value for compound 8b and its carbene

Neutral precursor:

Electronic energy: -745.02152345 hartrees

Zero point energy: 0.26927880 hartrees

Sum of electronic energy and ZPE: -744.75224464 hartrees

Corresponding carbene:

Electronic energy: -744.46268520 hartrees

Zero point energy: 0.25539694 hartrees

Sum of electronic energy and ZPE: -744.20728826 hartrees

Resulting CREF value: **0.545**

2-(1,3-Dimethyl-5-(p-tolyl)-1H-imidazol-3-ium-4-yl)pyrrol-1-ide (8c), precursor:

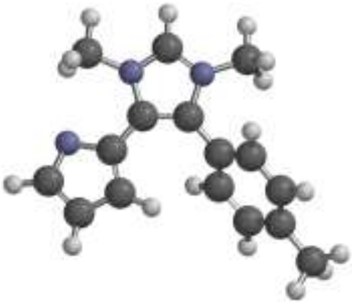

Charge: 0

Number of imaginary frequencies: 0

E: - 784.3495600875 hartrees

Table S6. HOMO/LUMO profiles and energies of 8c (precursor)

LUMO 8c	HOMO 8c
LUMO+2 (-0.77 eV)	HOMO (-4.20 eV)
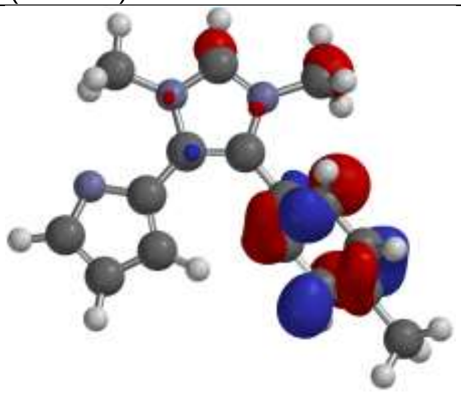	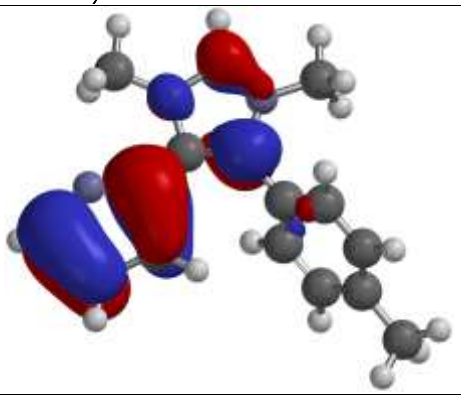
LUMO+1 (-0.85 eV)	HOMO-1 (-5.01 eV)
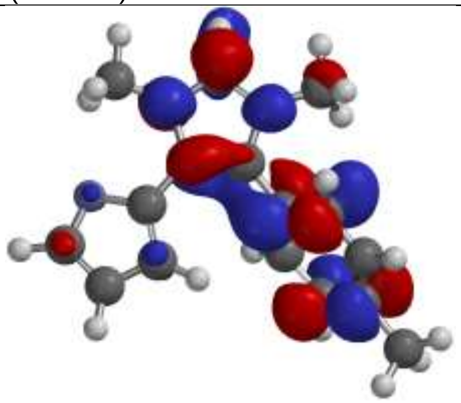	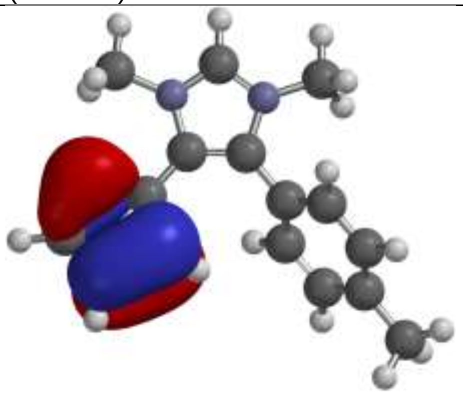
LUMO (-1.16 eV)	HOMO-2 (-5.94 eV)
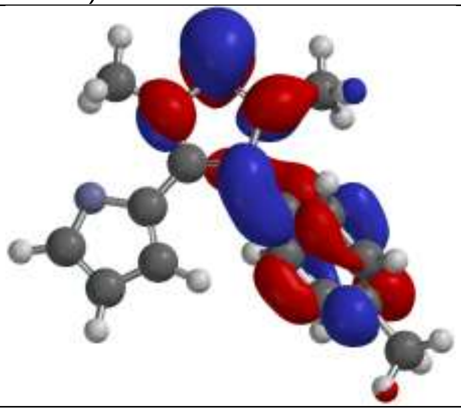	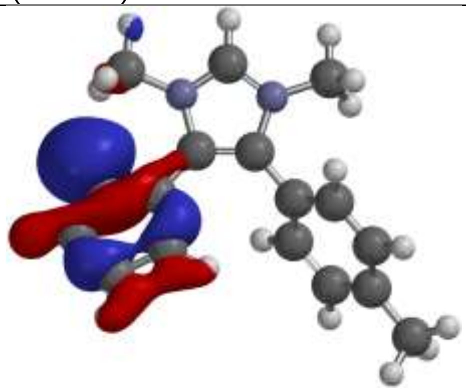

	HOMO-3 (-6.82 eV)
	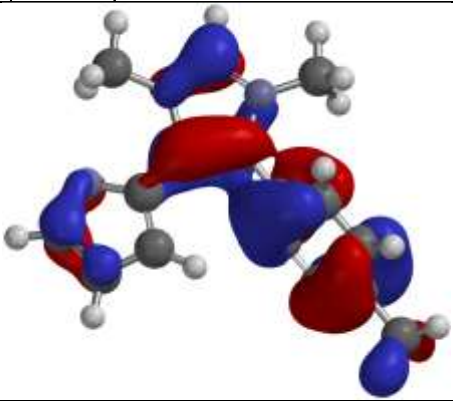
	HOMO-4 (-7.27 eV)
	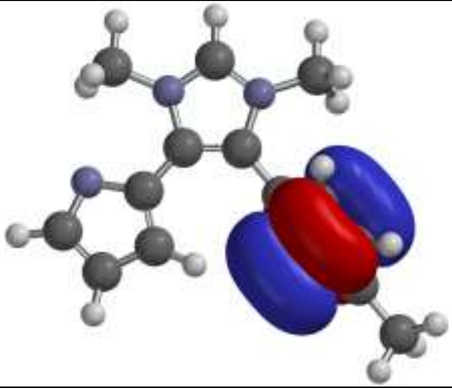

2-(1,3-Dimethyl-5-(p-tolyl)-1H-imidazol-3-ium-4-yl)pyrrol-1-ide , anionic carbene from 8c:

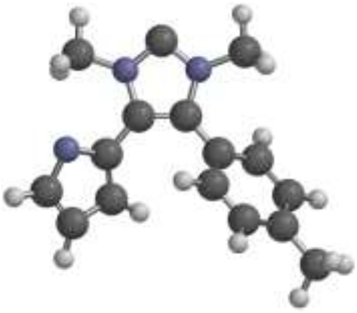

Charge: -1

Number of imaginary frequencies: 0

E: -783.7893353036 hartrees

Table S7. HOMO/LUMO profiles and energies of 8c (carbene)

LUMO, anionic carbene form 8c	HOMO anionic carbene form 8c
LUMO+2 (2.11 eV)	HOMO (-0.89 eV)
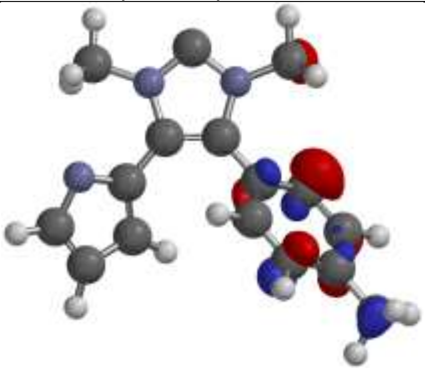	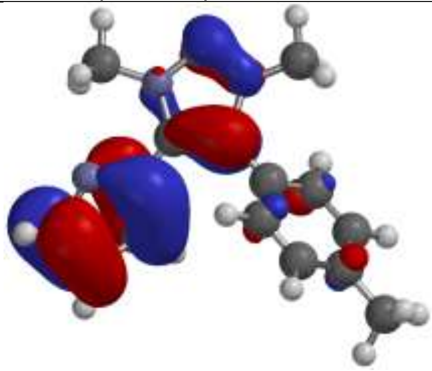

LUMO+1 (2.03 eV)	HOMO-1 (-1.80 eV)
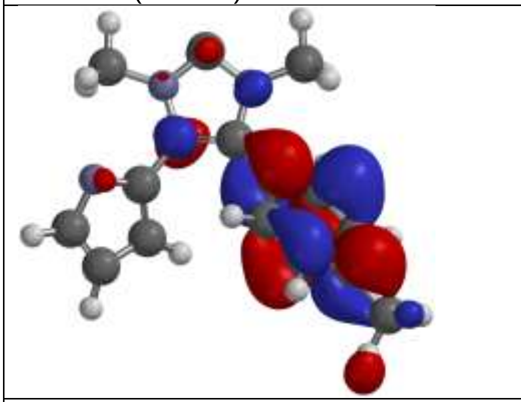	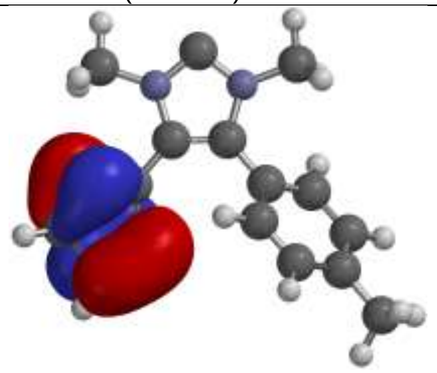
LUMO (1.71 eV)	HOMO-2 (-2.54 eV)
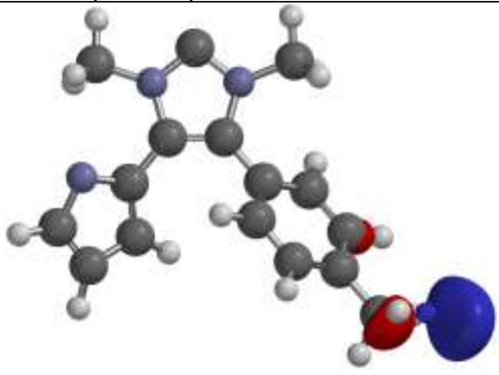	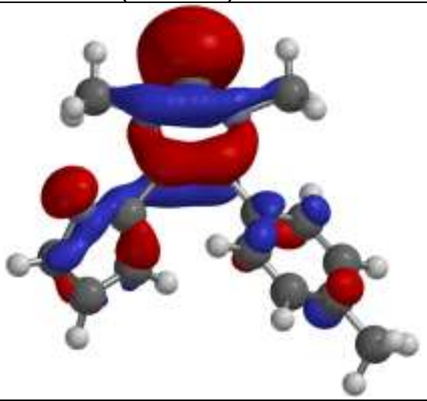
	HOMO-3 (-2.70 eV)
	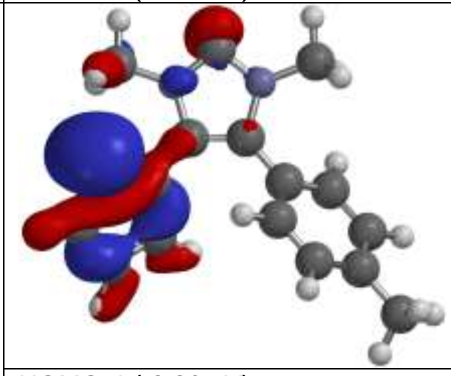
	HOMO-4 (-2.86 eV)
	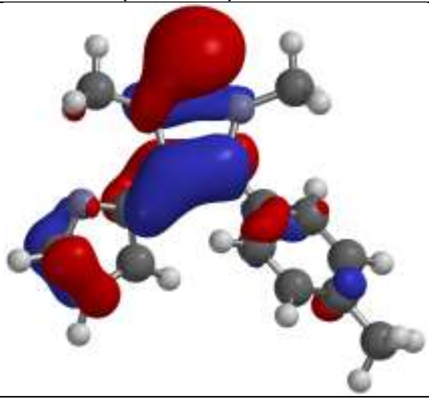

Calculated CREF value for compound 8c and its carbene

Neutral precursor:

Electronic energy: - 784.34956009 hartrees

Zero point energy: 0.29651505 hartrees

Sum of electronic energy and ZPE: -784.05304504 hartrees

Corresponding carbene:

Electronic energy: -783.78933530 hartrees

Zero point energy: 0.28271286 hartrees

Sum of electronic energy and ZPE: -783.50662244 hartrees

Resulting CREF value: **0.546**

2-(5-(4-Methoxyphenyl)-1,3-dimethyl-1H-imidazol-3-ium-4-yl)pyrrol-1-ide (8d), precursor:

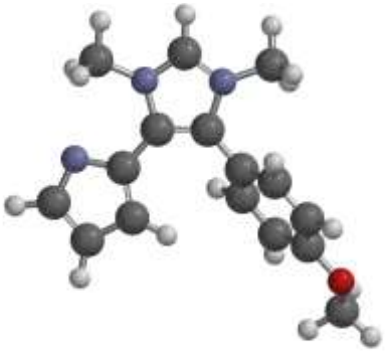

Charge: 0

Number of imaginary frequencies: 0

E: -859.5787526616 hartrees

Table S8. HOMO/LUMO profiles and energies of 8d (precursor)

LUMO 8d	HOMO 8d
LUMO+2 (-0.75 eV)	HOMO (-4.17 eV)
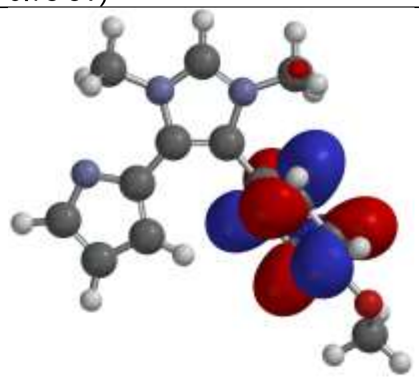	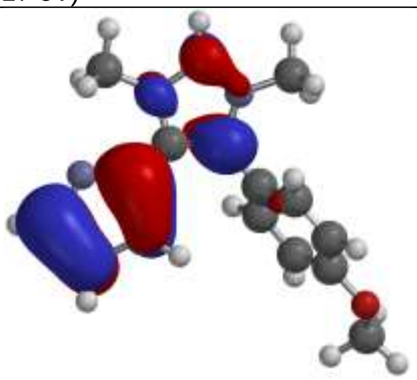

LUMO+1 (-1.07 eV)	HOMO-1 (-4.98 eV)
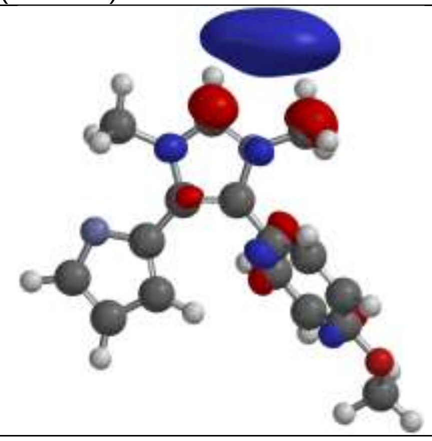	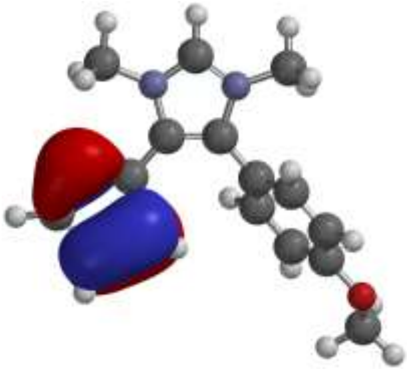
LUMO (-1.16 eV)	HOMO-2 (-5.91 eV)
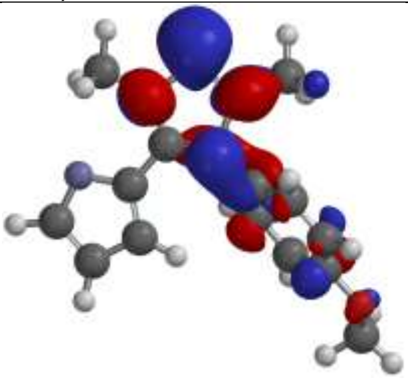	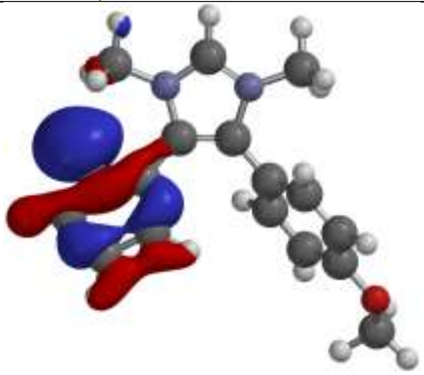
	HOMO-3 (-6.46 eV)
	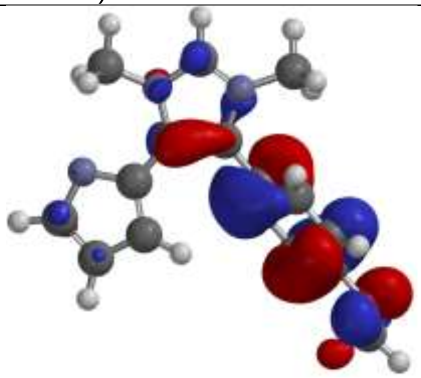
	HOMO-4 (-7.31 eV)
	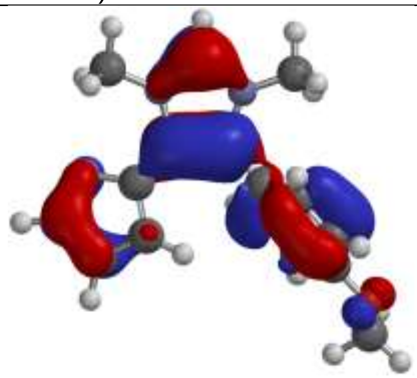

2-(5-(4-Methoxyphenyl)-1,3-dimethyl-1H-imidazol-3-ium-4-yl)pyrrol-1-ide , anionic carbene from 8d:

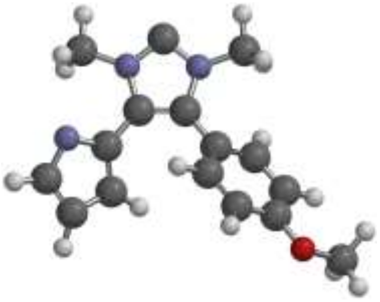

Charge: -1

Number of imaginary frequencies: 0

E: -859.0173217691 hartrees

Table S9. HOMO/LUMO profiles and energies of 8d (carbene)

LUMO, anionic carbene form 7d	HOMO anionic carbene form 7d
LUMO+2 (2.19 eV)	HOMO (-0.87 eV)
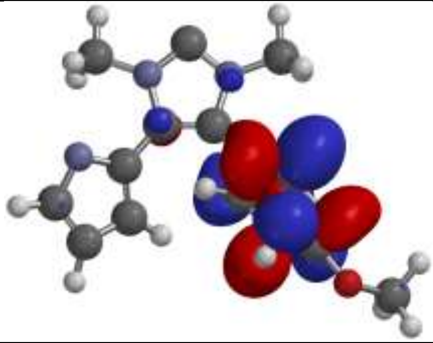	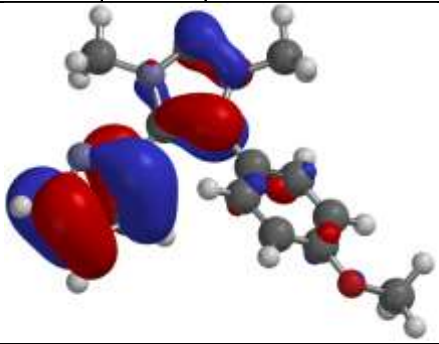
LUMO+1 (2.04 eV)	HOMO-1 (-1.77 eV)
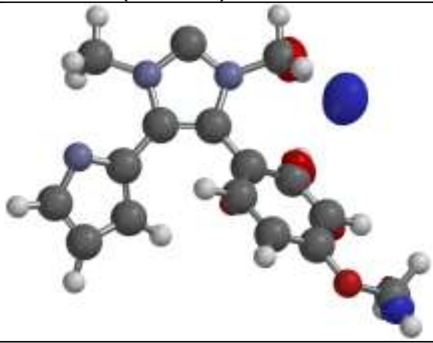	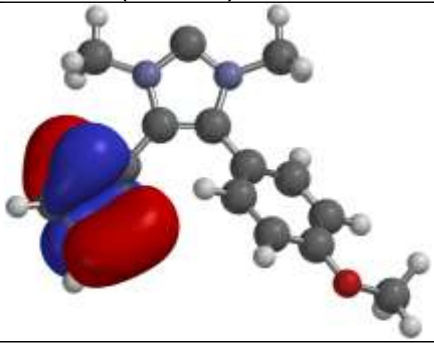
LUMO (1.45 eV)	HOMO-2 (-2.50 eV)
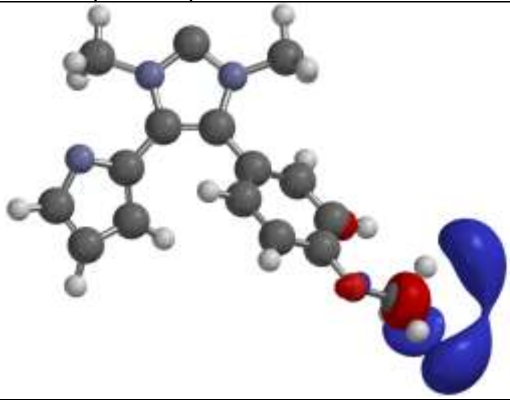	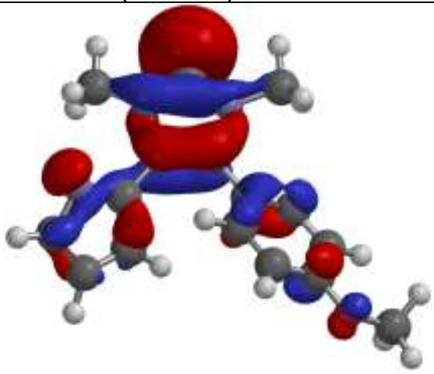

	HOMO-3 (-2.68 eV)
	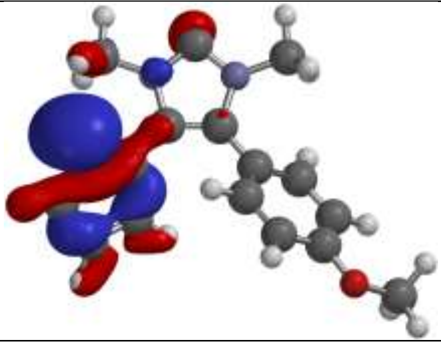
	HOMO-4 (-2.83 eV)
	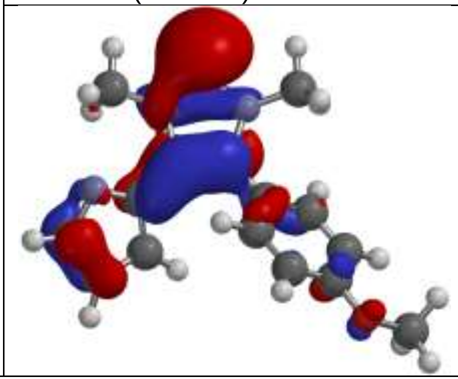

Calculated CREF value for compound 8d and its carbene

Neutral precursor:

Electronic energy:	-859.57875266 hartrees
Zero point energy:	0.30169744 hartrees
Sum of electronic energy and ZPE:	-859.27705522 hartrees

Corresponding carbene:

Electronic energy:	-859.01732177 hartrees
Zero point energy:	0.28752873 hartrees
Sum of electronic energy and ZPE:	-858.72979304 hartrees

Resulting CREF value: 0.547

2-(1-Benzyl-3-methyl-1H-imidazol-3-ium-4-yl)pyrrol-1-ide (8e), precursor:

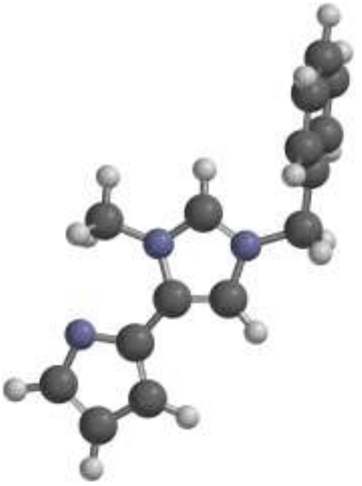

Charge: 0

Number of imaginary frequencies: 0

E: -745.0191754162 hartrees

Table S10. HOMO/LUMO profiles and energies of 8e (precursor)

LUMO 8e	HOMO 8e
LUMO+2 (-1.03 eV)	HOMO (-4.22 eV)
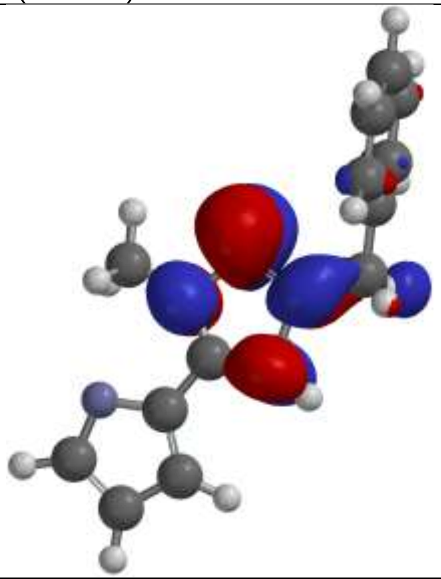	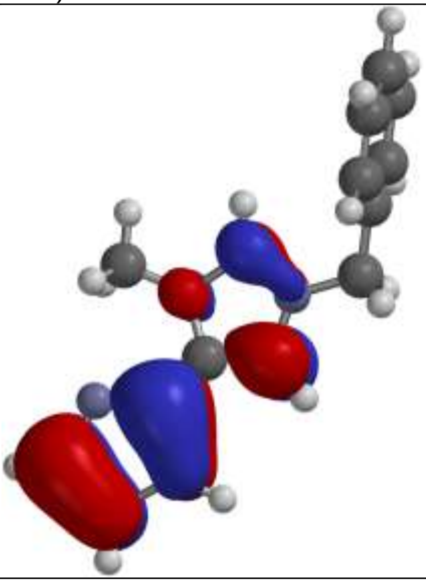
LUMO+1 (-1.40 eV)	HOMO-1 (-5.03 eV)
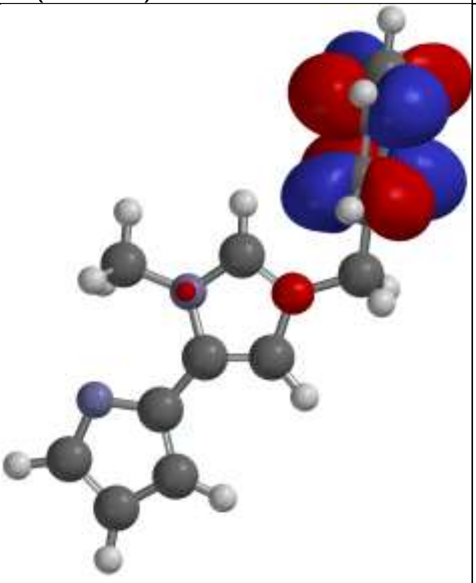	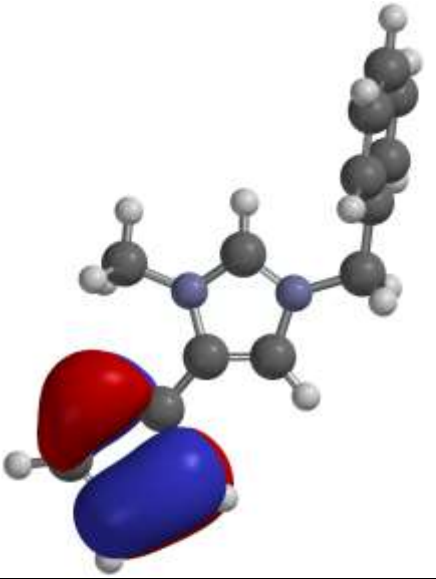

LUMO (-1.57 eV)	HOMO-2 (-5.96 eV)
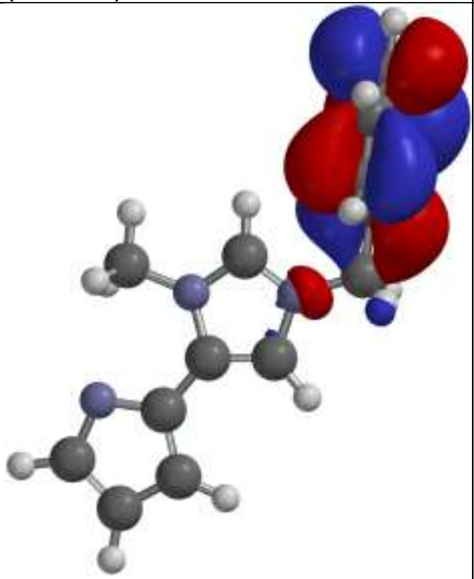	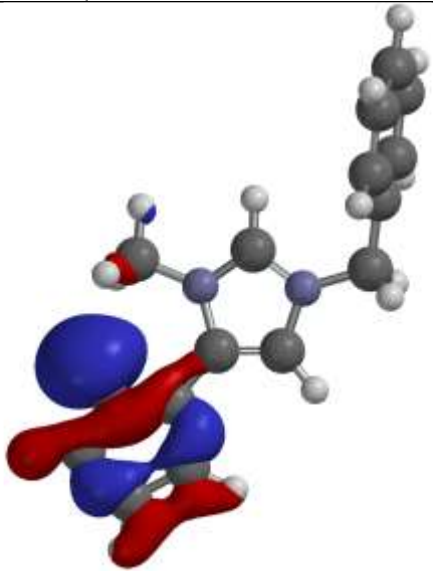
	HOMO-3 (-7.36 eV)
	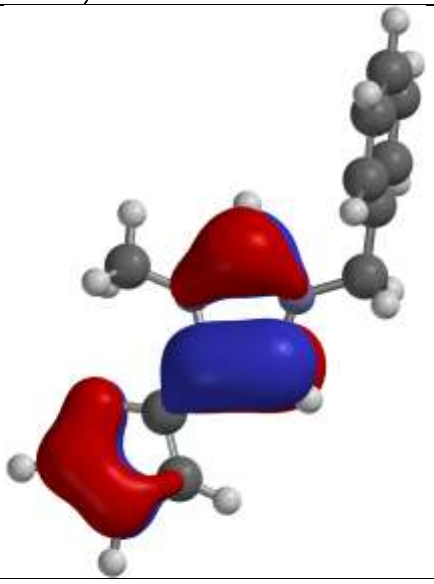
	HOMO-4 (-7.97 eV)

	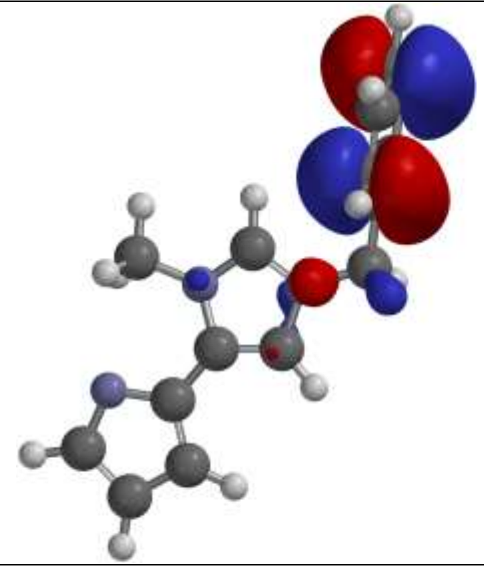

2-(1-Benzyl-3-methyl-1H-imidazol-3-ium-4-yl)pyrrol-1-ide, anionic carbene from 8e:

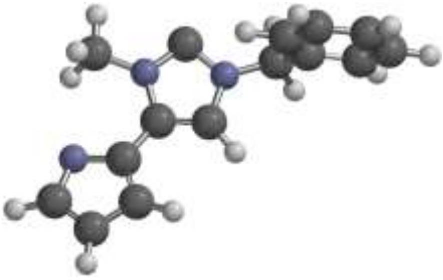

Charge: -1

Number of imaginary frequencies: 0

E: -744.4596786651 hartrees

Table S11. HOMO/LUMO profiles and energies of 8e (carbene)

LUMO, anionic carbene form 8e	HOMO anionic carbene form 8e
LUMO+2 (1.74 eV)	HOMO (-0.75 eV)
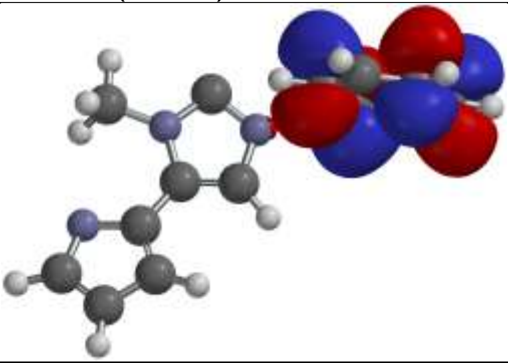	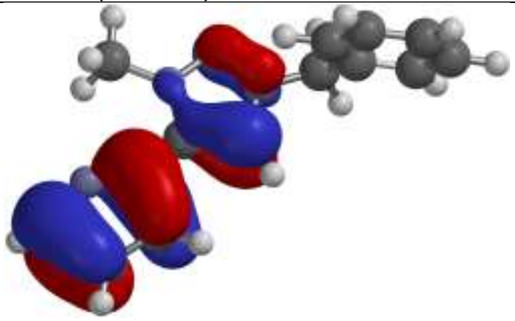
LUMO+1 (1.67 eV)	HOMO-1 (-1.73 eV)
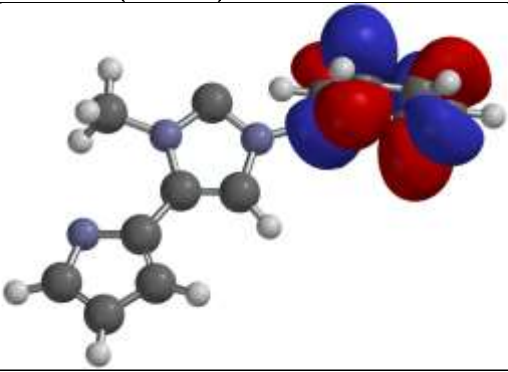	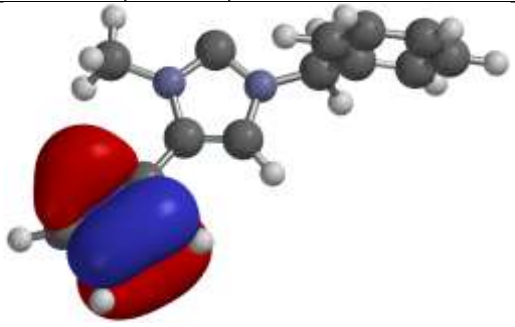
LUMO (1.56 eV)	HOMO-2 (-2.58 eV)

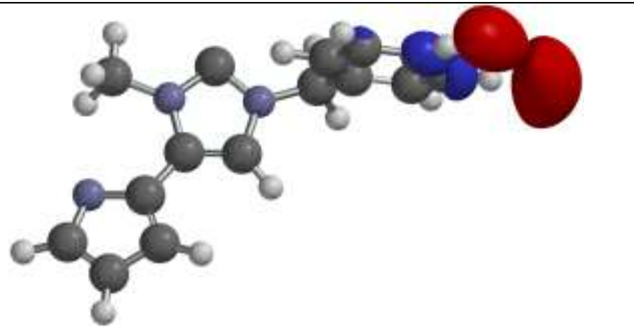	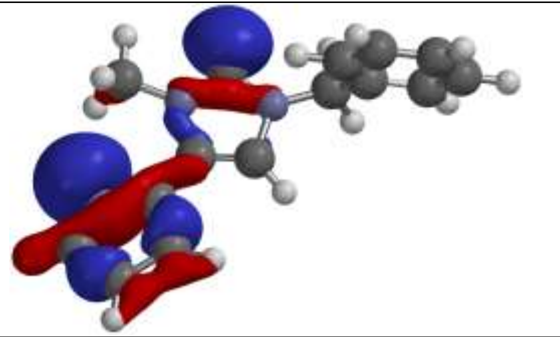

	HOMO-3 (-2.69 eV)
	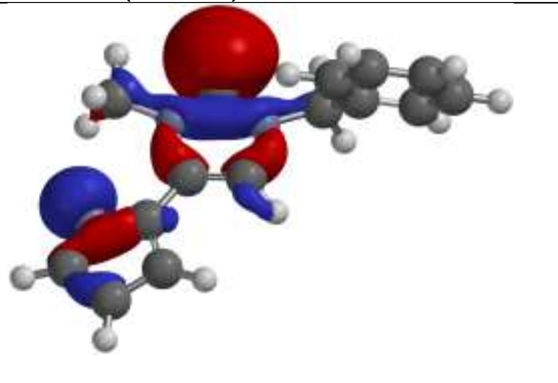
	HOMO-4 (-3.04 eV)
	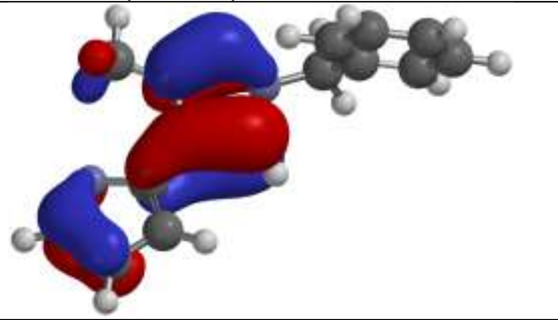

Calculated CREF value for compound 8e and its carbene

Neutral precursor:

Electronic energy:	-745.01917542 hartrees
Zero point energy:	0.26989553 hartrees
Sum of electronic energy and ZPE:	-744.74927989 hartrees

Corresponding carbene:

Electronic energy:	-744.45967867 hartrees
Zero point energy:	0.25581606 hartrees
Sum of electronic energy and ZPE:	-744.20386261 hartrees

Resulting CREF value: **0.545**

2-(3-Methyl-1-(4-methylbenzyl)-1H-imidazol-3-ium-4-yl)pyrrol-1-ide (8f), precursor:

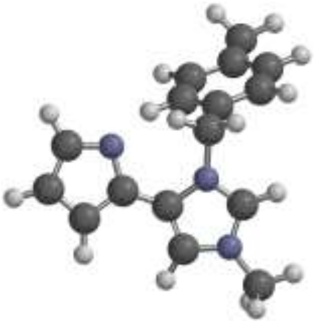

Charge: 0

Number of imaginary frequencies: 0

E: -784.3486257810 ha

Table S12. HOMO/LUMO profiles and energies of 8f (precursor)

LUMO 8f	HOMO 8f
LUMO+2 (-0.57 eV)	HOMO (-4.36 eV)
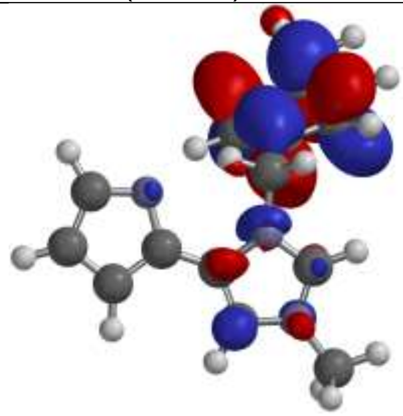	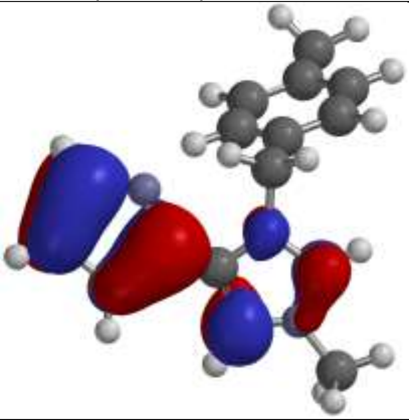
LUMO+1 (-0.85 eV)	HOMO-1 (-5.18 eV)
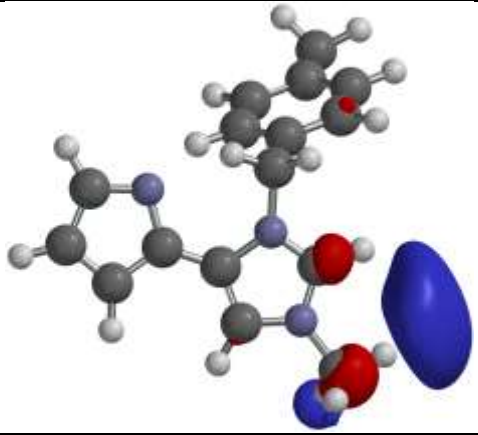	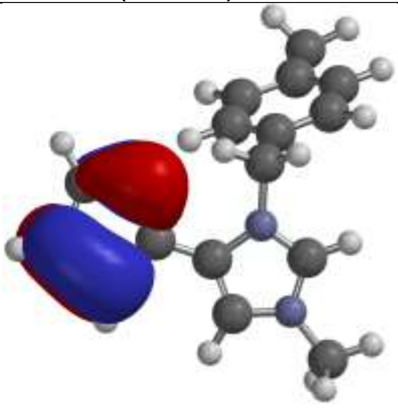
LUMO (-1.22 eV)	HOMO-2 (-6.12 eV)
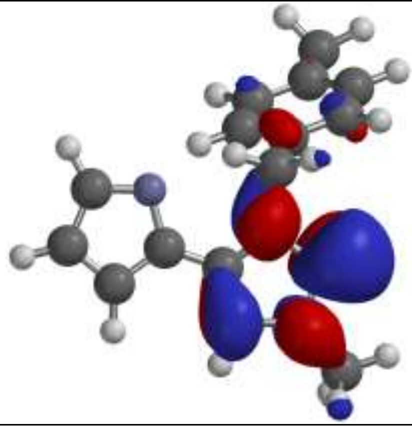	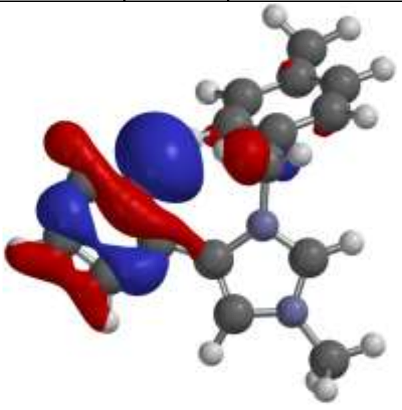

	HOMO-3 (-6.78 eV)
	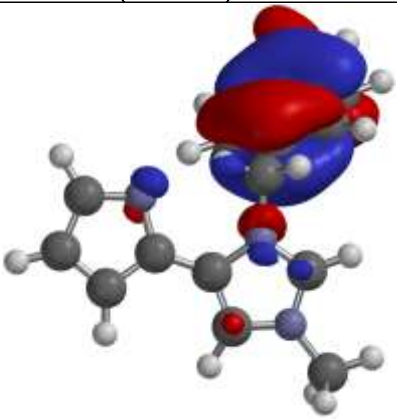
	HOMO-4 (-7.06 eV)
	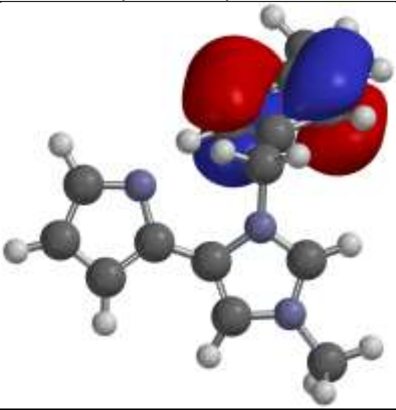

2-(3-Methyl-1-(4-methylbenzyl)-1H-imidazol-3-ium-4-yl)pyrrol-1-ide, anionic carbene from 8f:

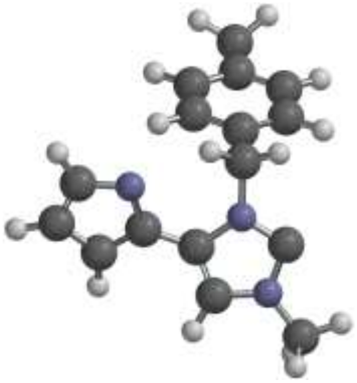

Charge: -1

Number of imaginary frequencies: 0

E: -783.7878483559 ha

Table S13. HOMO/LUMO profiles and energies of 8f (carbene)

LUMO, anionic carbene form 8f	HOMO anionic carbene form 8f
LUMO+2 (2.28 eV)	HOMO (-0.86 eV)
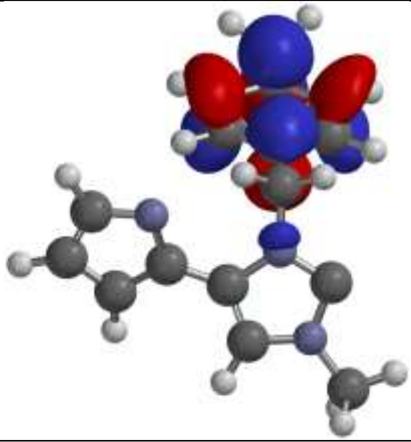	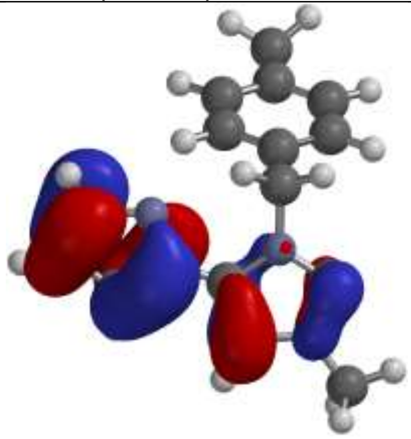
LUMO+1 (2.08 eV)	HOMO-1 (-1.83 eV)
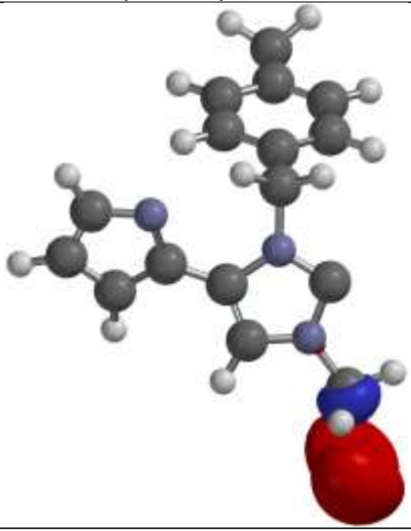	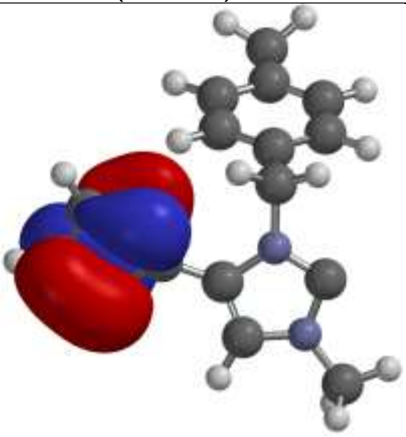

LUMO (1.79 eV)	HOMO-2 (-2.60 eV)
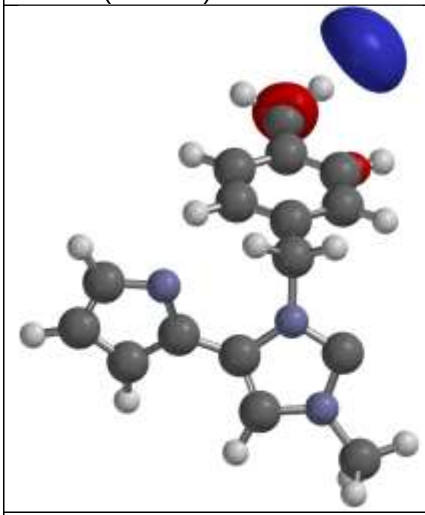	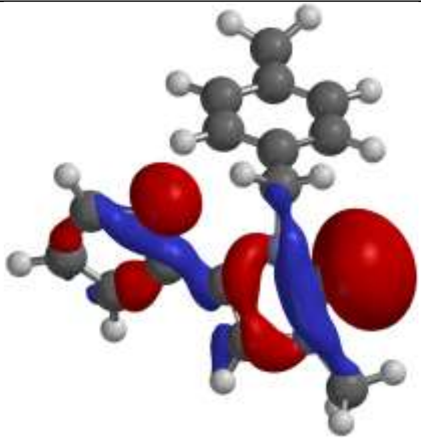
	HOMO-3 (-2.76 eV)
	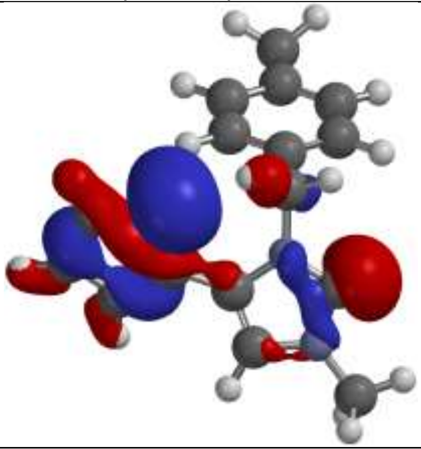
	HOMO-4 (-3.02 eV)
	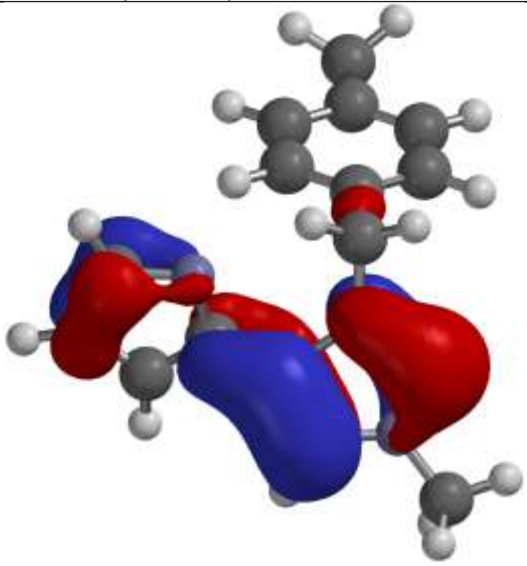

Calculated CREF value for compound 8f and its carbene

Precursor :

Electronic energy:	-784.34862578 ha
Zero point energy:	0.29691823 ha
Sum of electronic and ZPE:	-784.05170755 ha

Corresponding carbene:

Electronic energy:	-783.78784836 ha
Zero point energy:	0.28345867 ha
Sum of electronic and ZPE:	-783.50438969 ha

Resulting CREF value: **0.547**

2-(1-(4-Methoxybenzyl)-3-methyl-1H-imidazol-3-ium-4-yl)pyrrol-1-ide (8g), precursor:

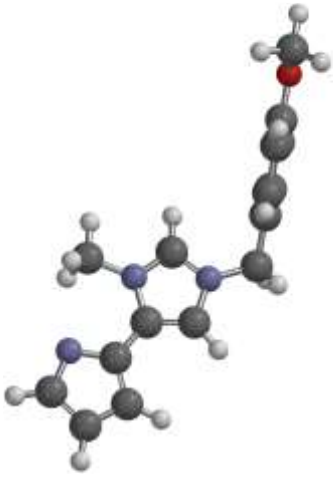

Charge: 0

Number of imaginary frequencies: 0

E: -859.5764916027 hartrees

Table S14. HOMO/LUMO profiles and energies of 8g (precursor)

LUMO 8g	HOMO 8g
LUMO+2 (-1.00 eV)	HOMO (-4.17 eV)

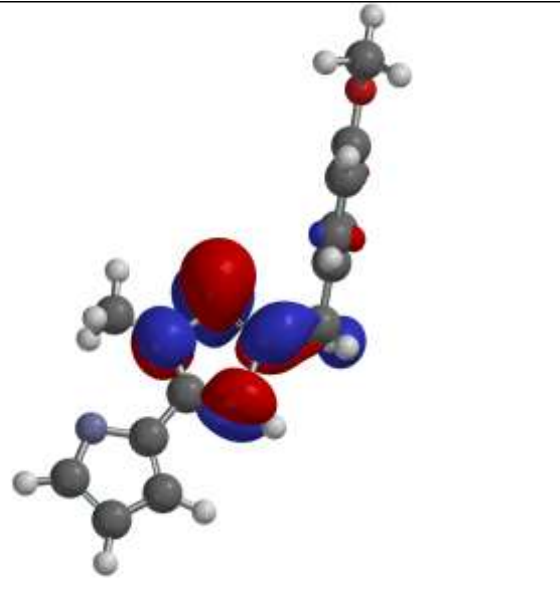

LUMO+1 (-1.11 eV)

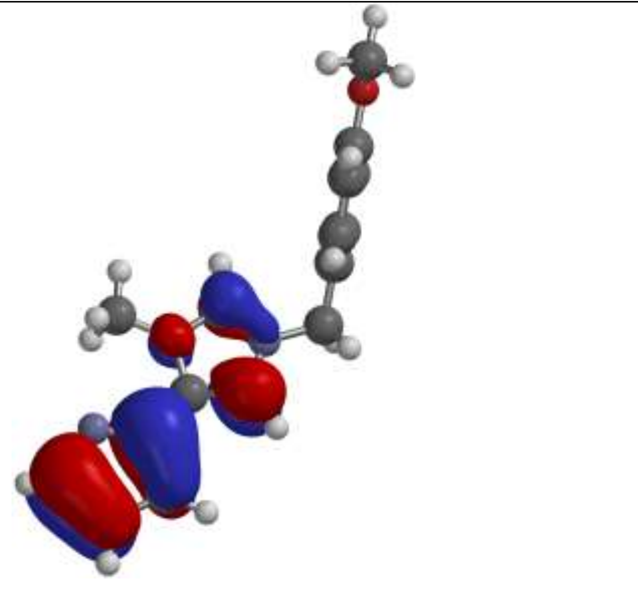

HOMO-1 (-5.00 eV)

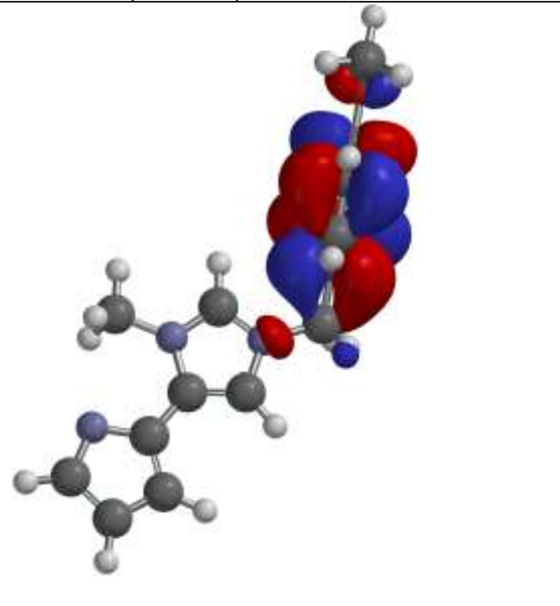

LUMO (-1.39 eV)

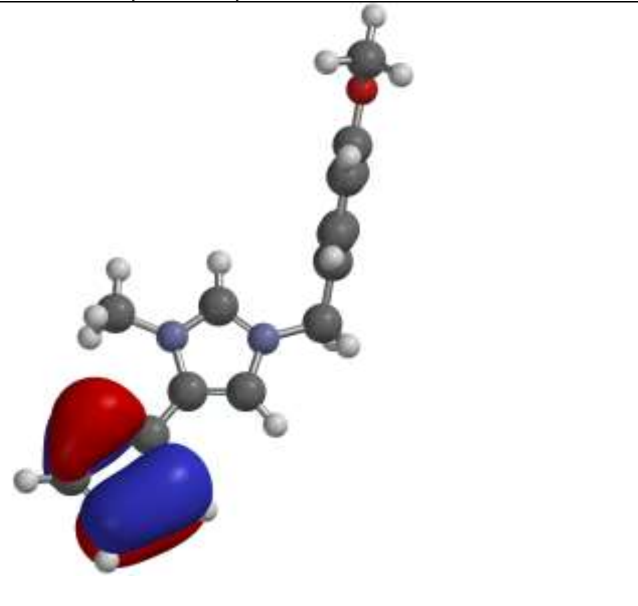

HOMO-2 (-5.92 eV)

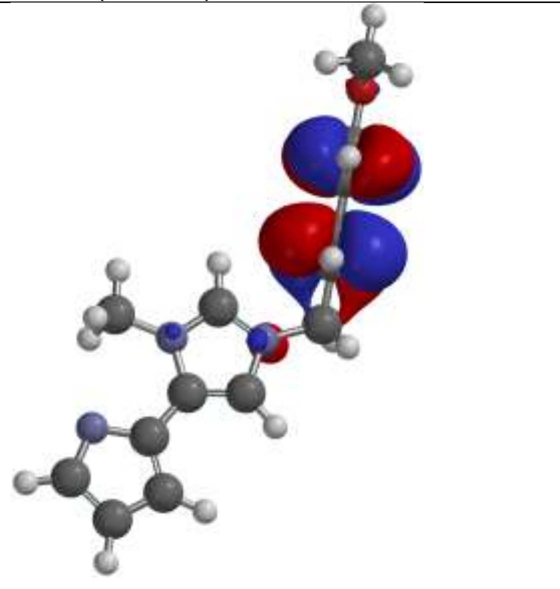

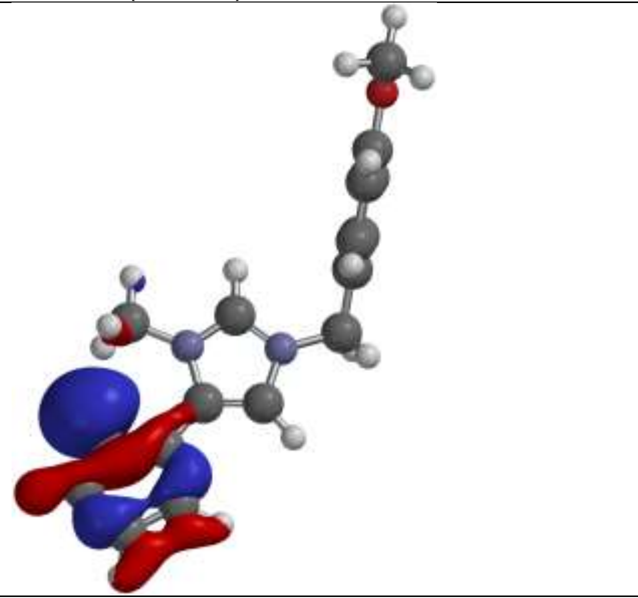

	HOMO-3 (-7.02 eV)
	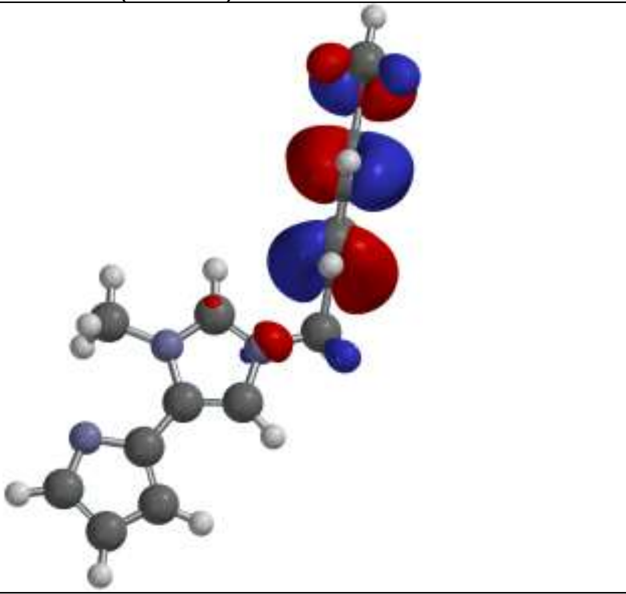
	HOMO-4 (-7.32 eV)
	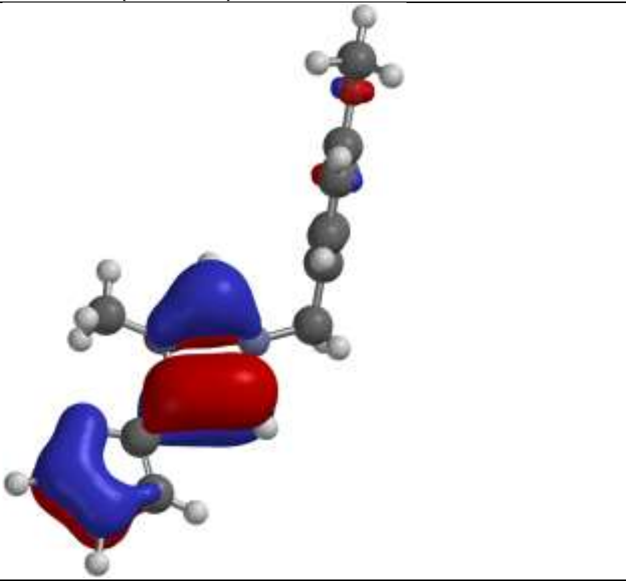

2-(1-(4-Methoxybenzyl)-3-methyl-1H-imidazol-3-ium-4-yl)pyrrol-1-ide, anionic carbene from 8g:

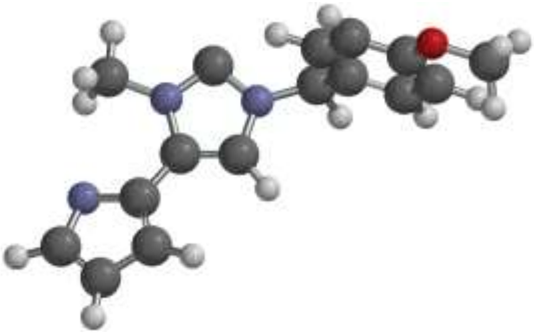

Charge: -1

Number of imaginary frequencies: 0

E: -859.0145163544 hartrees

Table S15. HOMO/LUMO profiles and energies of 8g (carbene)

LUMO, anionic carbene form 8g	HOMO anionic carbene form 8g
LUMO+2 (1.84 eV)	HOMO (-0.72 eV)

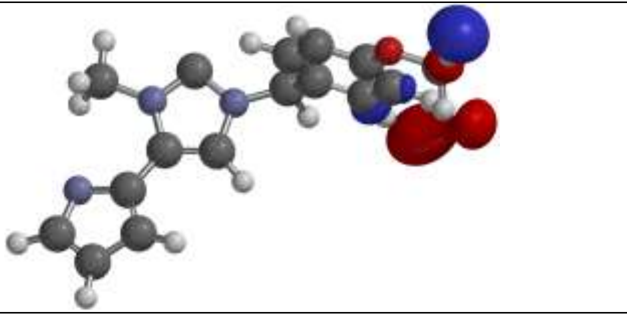	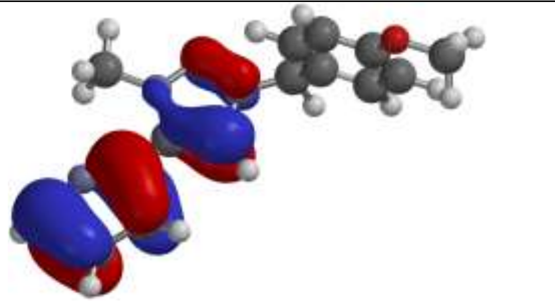
LUMO+1 (1.62 eV)	HOMO-1 (-1.68 eV)
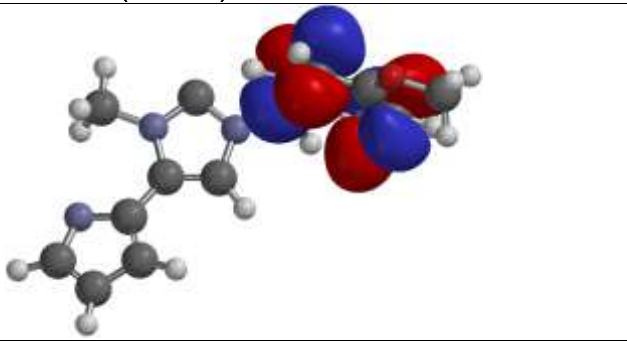	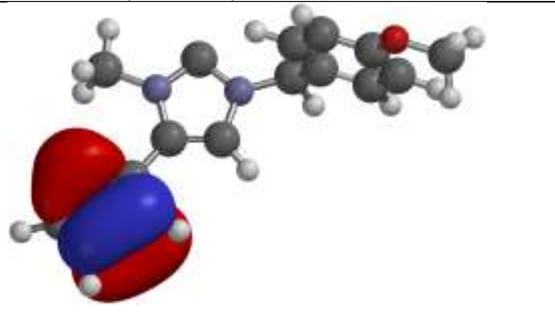

LUMO (1.21 eV)	HOMO-2 (-2.54 eV)
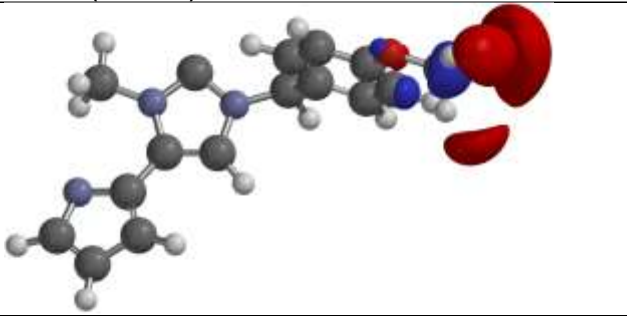	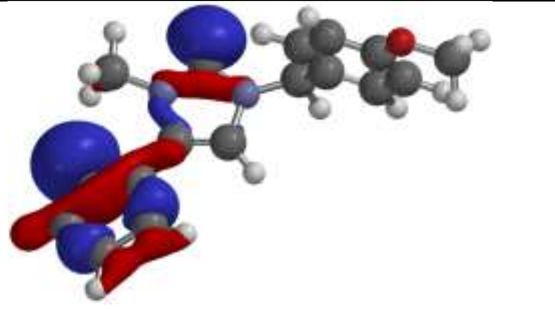
	HOMO-3 (-2.66 eV)
	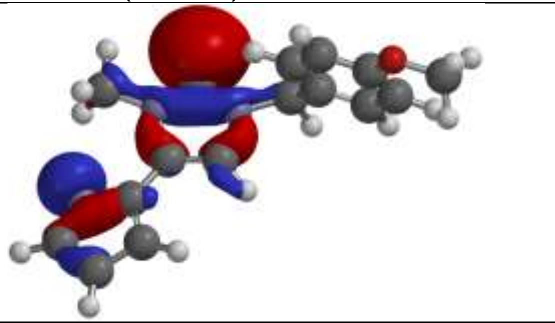
	HOMO-4 (-2.99 eV)
	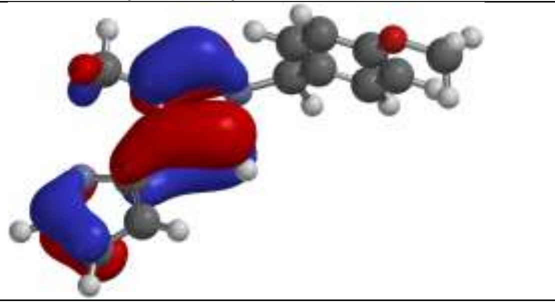

Calculated CREF value for compound 8g and its carbene

Neutral precursor:

Electronic energy: -859.57649160 hartrees

Zero point energy: 0.30200181 hartrees

Sum of electronic energy and ZPE: -859.27448979 hartrees

Corresponding carbene:

Electronic energy: -859.01451635 hartrees

Zero point energy: 0.28793510 hartrees

Sum of electronic energy and ZPE: -858.72658126 hartrees

Resulting CREF value: **0.548**

2-(1-Benzyl-3-(tert-butyl)-1H-imidazol-3-ium-4-yl)pyrrol-1-ide (8h), precursor:

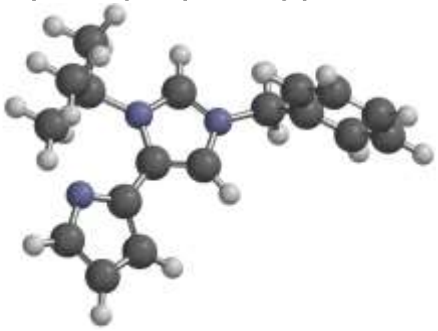

Charge: 0

Number of imaginary frequencies: 0

E: -862.9881239928 hartrees

Table S16. HOMO/LUMO profiles and energies of 8h (precursor)

LUMO 8h	HOMO 8h
LUMO+2 (-0.99 eV)	HOMO (-4.25 eV)
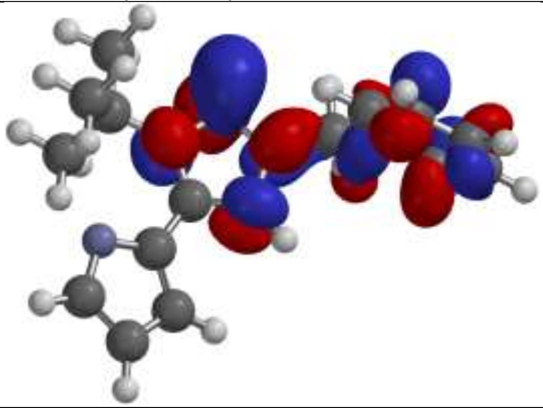	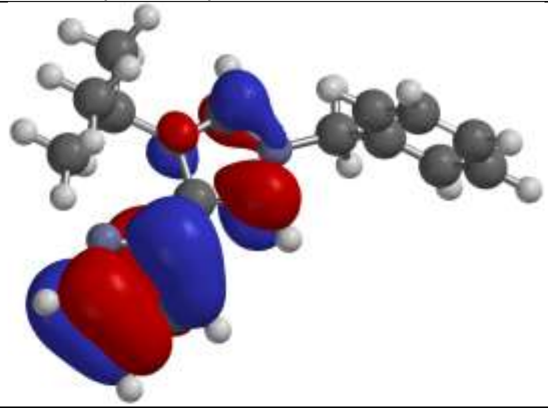
LUMO+1 (-1.17 eV)	HOMO-1 (-5.04 eV)

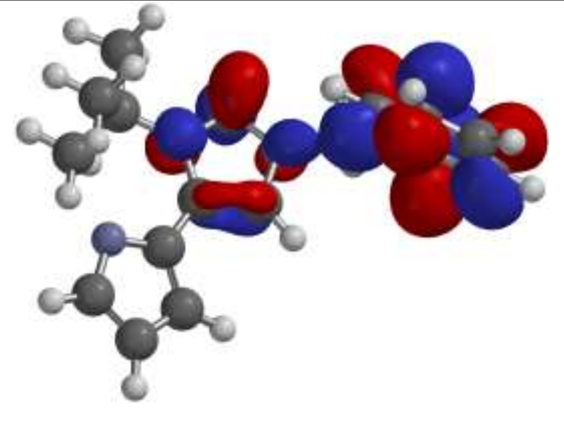	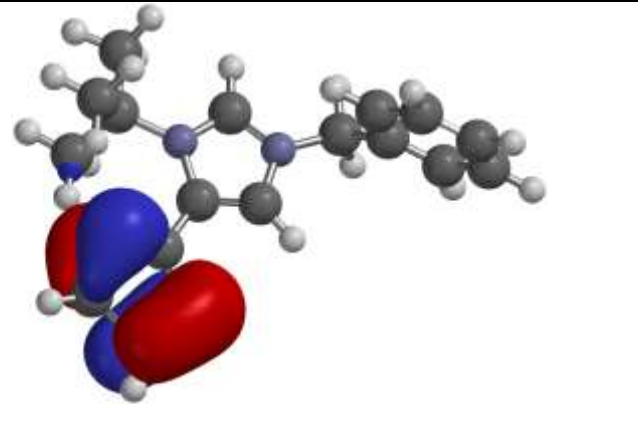
LUMO (-1.31 eV)	HOMO-2 (-5.94 eV)
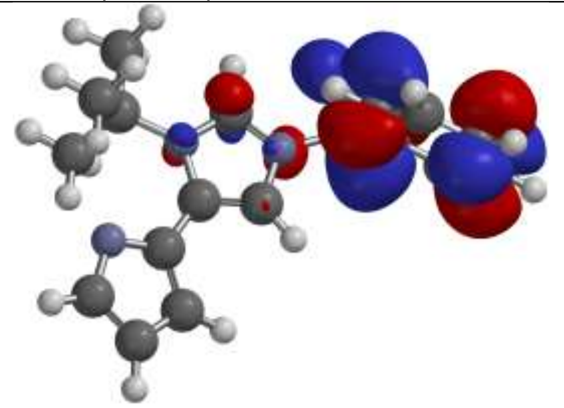	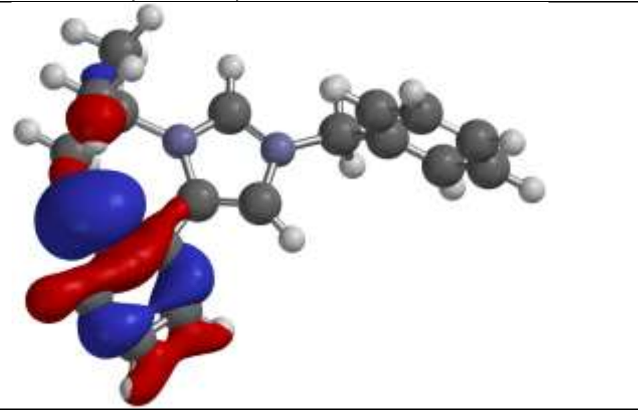

	HOMO-3 (-7.36 eV)
	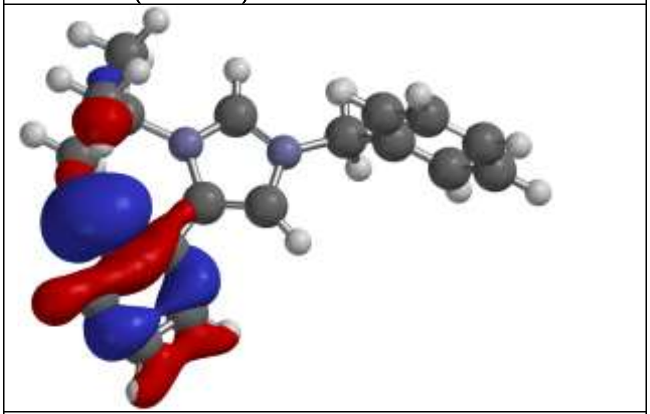
	HOMO-4 (-7.62 eV)
	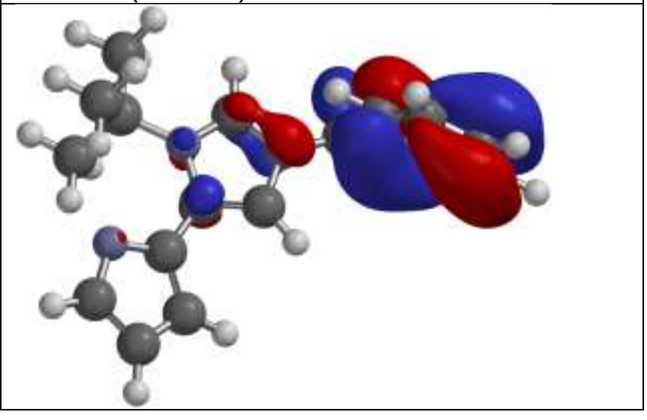

2-(1-Benzyl-3-(tert-butyl)-1H-imidazol-3-ium-4-yl)pyrrol-1-ide, anionic carbene from 8h:

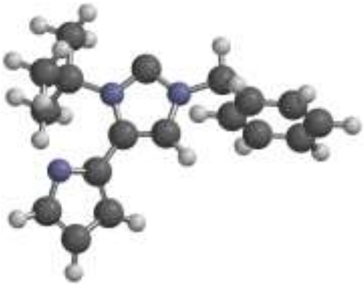

Charge: -1
Number of imaginary frequencies: 0
E: -862.4286005556 hartrees

Table S17. HOMO/LUMO profiles and energies of 8h (carbene)

LUMO, anionic carbene form 8h	HOMO anionic carbene form 8h
LUMO+2 (1.69 eV)	HOMO (-0.94 eV)
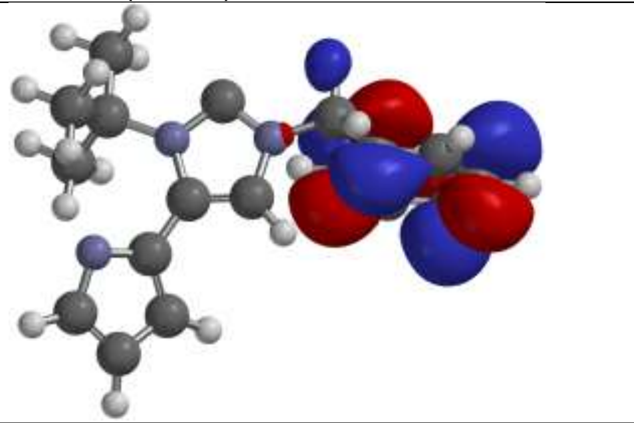	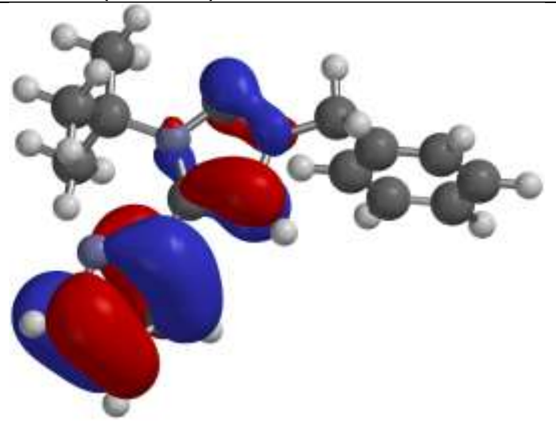

LUMO+1 (1.62 eV)	HOMO-1 (-1.76 eV)
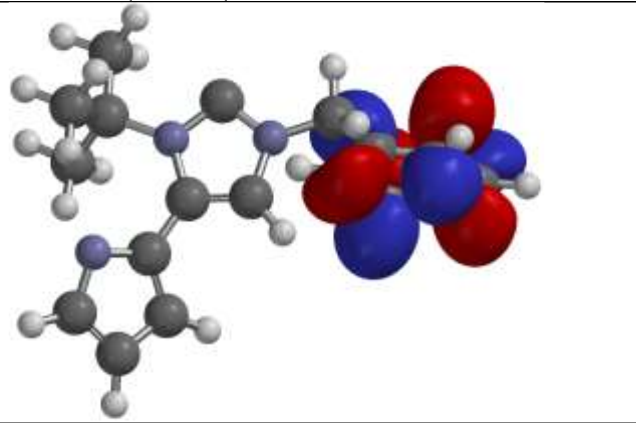	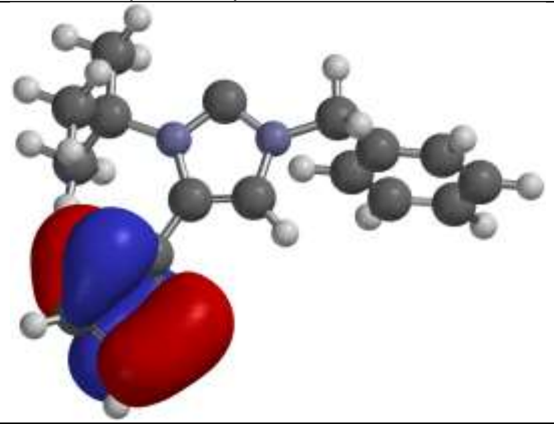
LUMO (1.54 eV)	HOMO-2 (-2.55 eV)

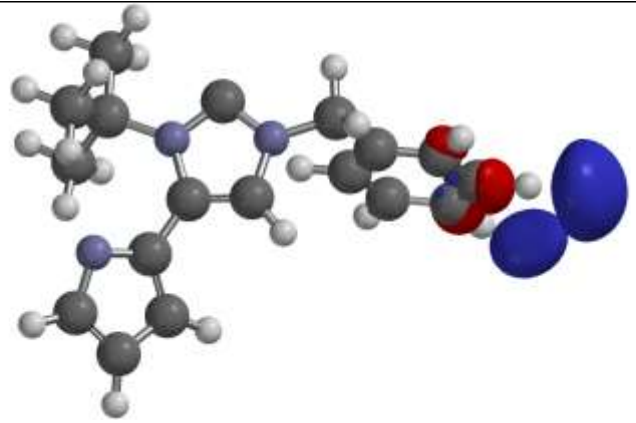	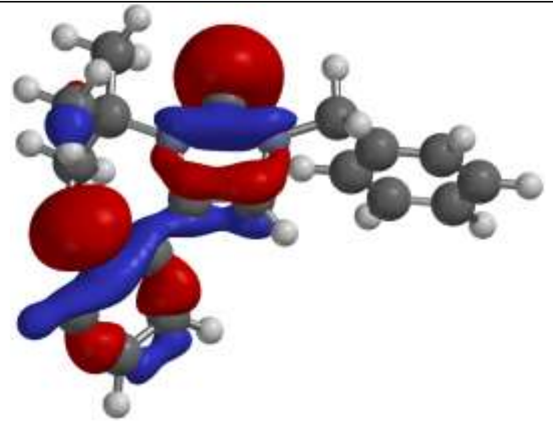
	HOMO-3 (-2.74 eV)
	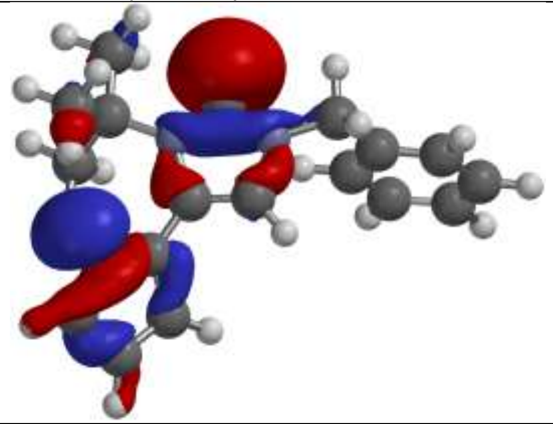
	HOMO-4 (-3.04 eV)
	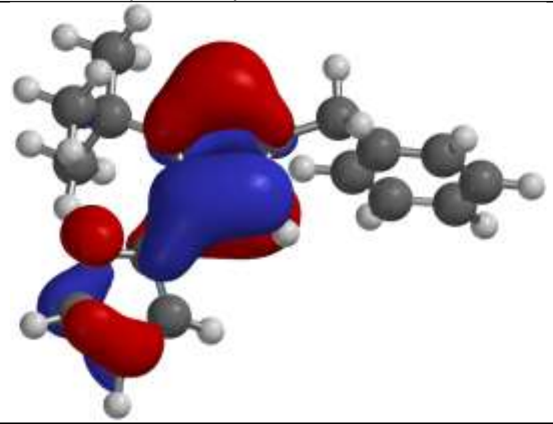

Calculated CREF value for compound 8h and its carbene

Neutral precursor:

Electronic energy:	-862.98812399 hartrees
Zero point energy:	0.35372534 hartrees
Sum of electronic energy and ZPE:	-862.63439866 hartrees

Corresponding carbene:

Electronic energy:	-862.42860056 hartrees
Zero point energy:	0.33948651 hartrees

Sum of electronic energy and ZPE: -862.08911405 hartrees

Resulting CREF value: **0.545**

2-(1-(tert-Butyl)-3-(4-methylbenzyl)-1H-imidazol-3-ium-4-yl)pyrrol-1-ide (8i), precursor:

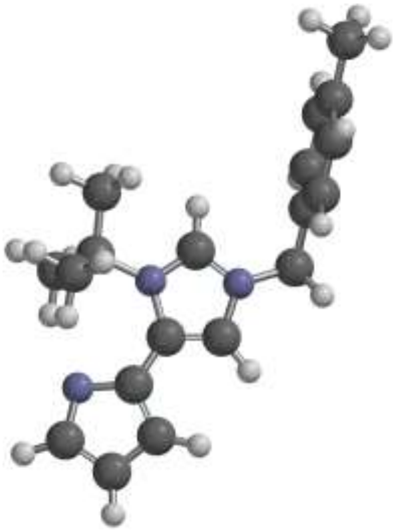

Charge: 0

Number of imaginary frequencies: 0

E: -902.3167802583 hartrees

Table S18. HOMO/LUMO profiles and energies of 8i (precursor)

LUMO 7i	HOMO 7i
LUMO+2 (-0.96 eV)	HOMO (-4.18 eV)
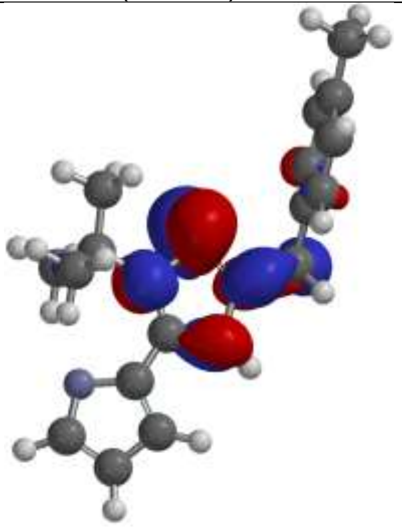	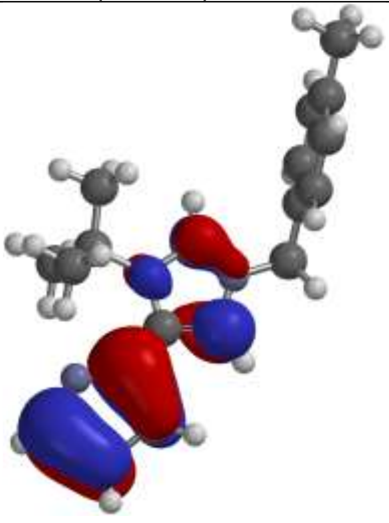
LUMO+1 (-1.31 eV)	HOMO-1 (-4.98 eV)

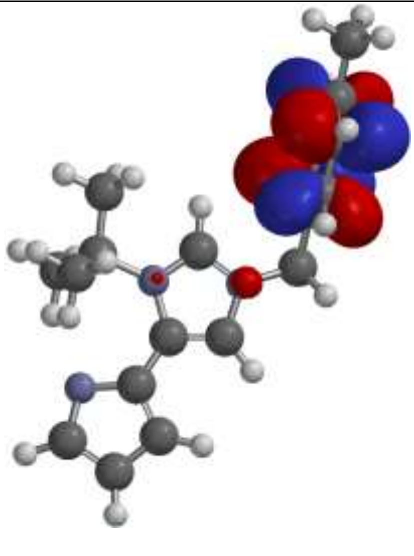	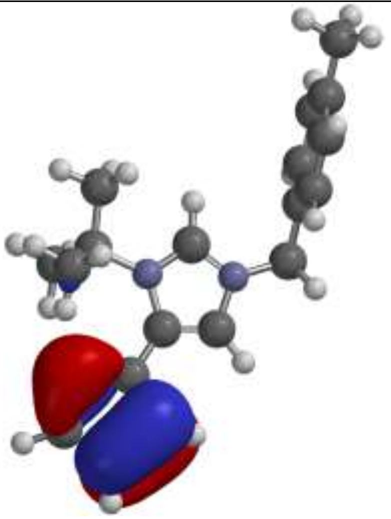
LUMO (-1.40 eV)	HOMO-2 (-5.87 eV)
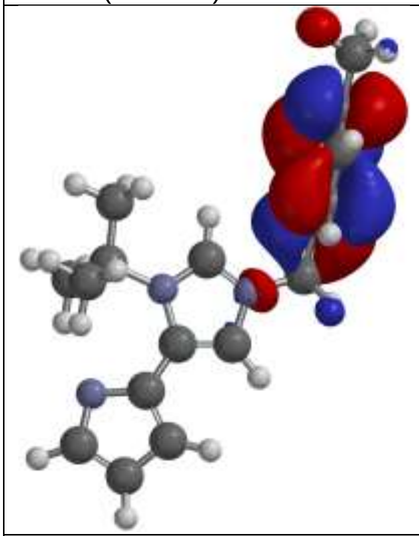	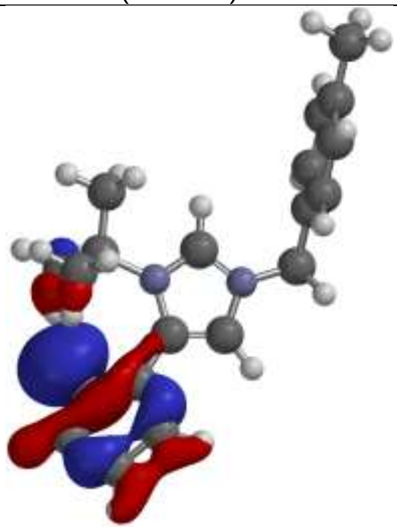
	HOMO-3 (-7.27 eV)
	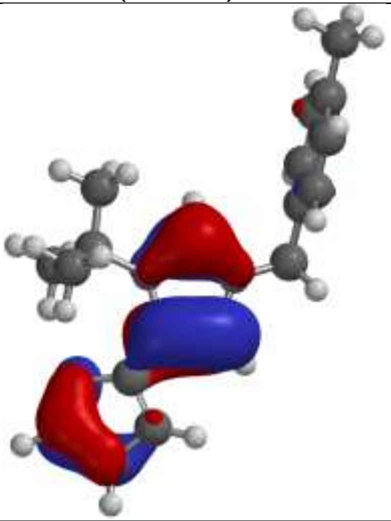
	HOMO-4 (-7.58 eV)

	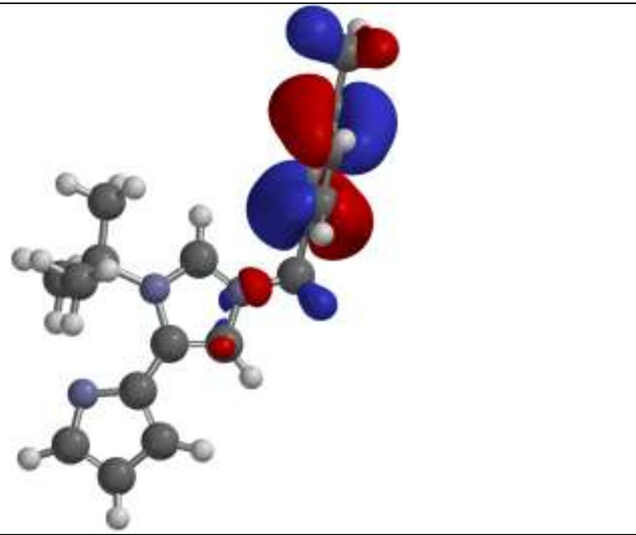

2-(3-(tert-Butyl)-1-(4-methylbenzyl)-1H-imidazol-3-ium-4-yl)pyrrol-1-ide, anionic carbene from 8i:

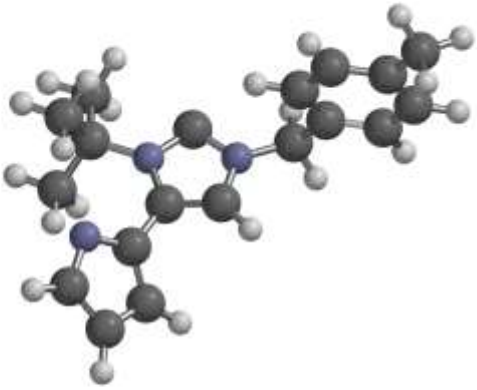

Charge: -1
Number of imaginary frequencies: 0
E: -901.7555879737 hartrees

Table S19. HOMO/LUMO profiles and energies of 8i (carbene)

LUMO, anionic carbene form 7i	HOMO anionic carbene form 7i
LUMO+2 (1.69 eV)	HOMO (-0.92 eV)
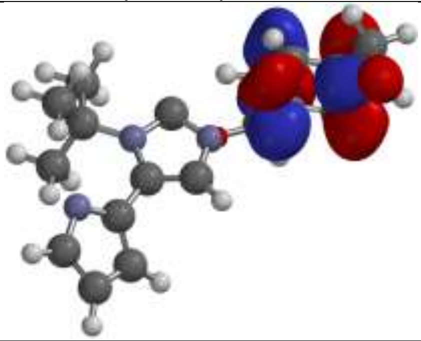	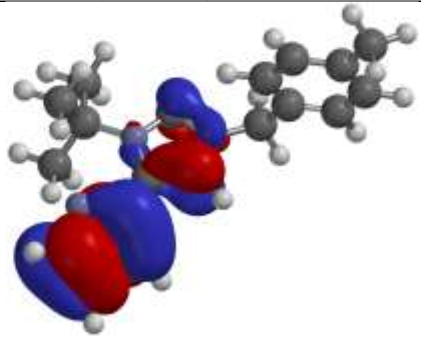
LUMO+1 (1.64 eV)	HOMO-1 (-1.75 eV)

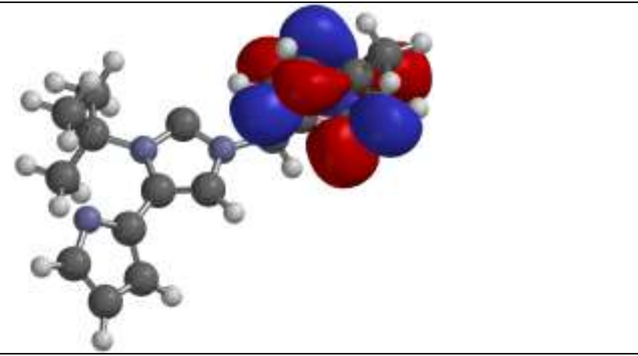	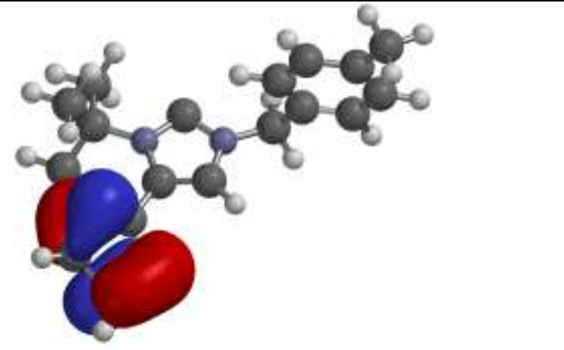

LUMO (1.42 eV)	HOMO-2 (-2.52 eV)
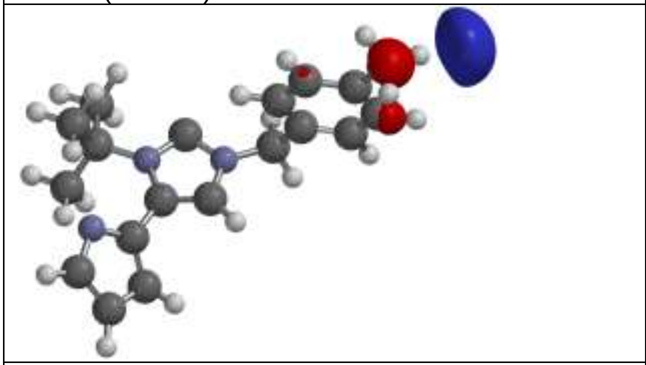	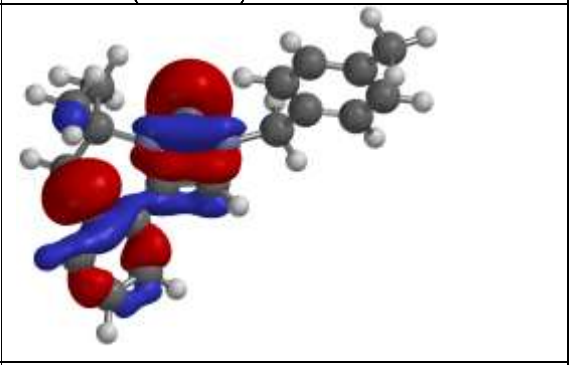
	HOMO-3 (-2.72 eV)
	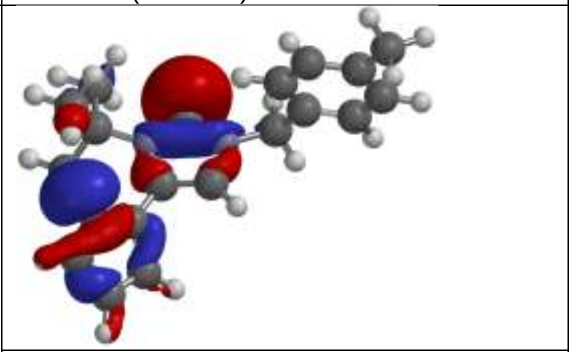
	HOMO-4 (-3.03 eV)
	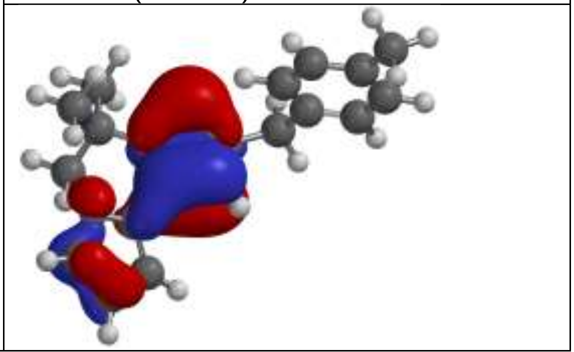

Calculated CREF value for compound 8i and its carbene

Neutral precursor:

Electronic energy: -902.31678026 hartrees

Zero point energy: 0.38080222 hartrees

Sum of electronic energy and ZPE:	-901.93597804 hartrees
Corresponding carbene:	
Electronic energy:	-901.75558797 hartrees
Zero point energy:	0.36656180 hartrees
Sum of electronic energy and ZPE:	-901.38902618 hartrees

Resulting CREF value: **0.547**

Geometrie optimizations calculations were performed using the Spartan Software (*Spartan'20*, Wavefunction, Inc., Irvine, CA. Available from: http://www.wavefun.com) running on a MS Windows 10 Pro PC system with an AMD Ryzen Threadripper 3970X 32-Core and 128 GB RAM. MMFF optimization was used with the B3LYP density functional and the 6-311++G(d,p) basis set carried out as vacuum calculations. Subsequent frequency calculations of all final structures evidenced the absence of imaginary frequencies and thus the presence of true minima on the potential energy surface.

Geometry optimization1,3-Dimethyl-4-(1H-pyrrol-2-yl)-1,3-dihydro-2H-imidazole-2-selenone (9a)

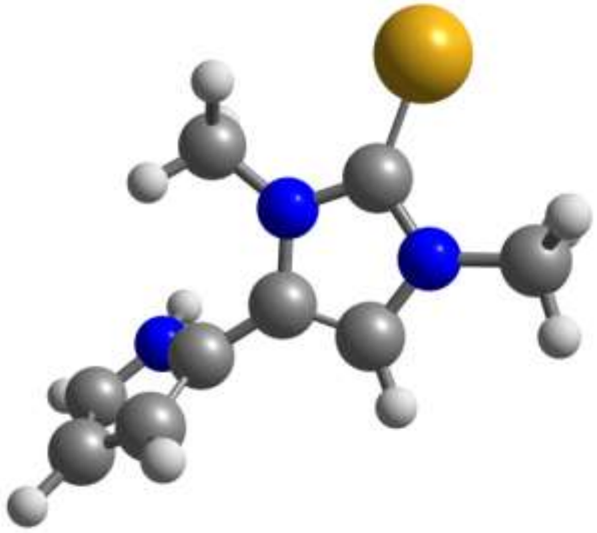

Charge: 0
E: -2915.52004784087 hartrees

	X	Y	Z
C	-0.692168	3.528671	-0.231527
C	0.278507	3.624250	0.740763
C	0.691937	2.302483	1.059398
C	-0.040408	1.428579	0.276299
N	-0.877995	2.198548	-0.511516
C	-0.036316	-0.026202	0.233177
C	-0.255759	-0.917539	1.236993

N	-0.120969	-2.191688	0.715639
C	0.169755	-2.126620	-0.620111
N	0.231601	-0.789746	-0.913770
Se	0.423376	-3.538222	-1.775973
C	0.601927	-0.277296	-2.225270
C	-0.280702	-3.435730	1.453395
H	-1.257428	4.290645	-0.743320
H	0.656955	4.540617	1.165594
H	1.455280	2.011026	1.763868
H	-1.573518	1.829905	-1.139830
H	-0.511648	-0.740260	2.267233
H	0.883889	0.769513	-2.125059
H	1.440369	-0.859053	-2.608422
H	-0.228290	-0.379874	-2.928708
H	-0.489653	-3.194096	2.495205
H	0.632345	-4.028499	1.383248
H	-1.101091	-4.019415	1.032694

Geometry optimization 1-Benzyl-3-(*tert*-butyl)-4-(1H-pyrrol-2-yl)-1,3-dihydro-2H-imidazole-2-selenone (9h)

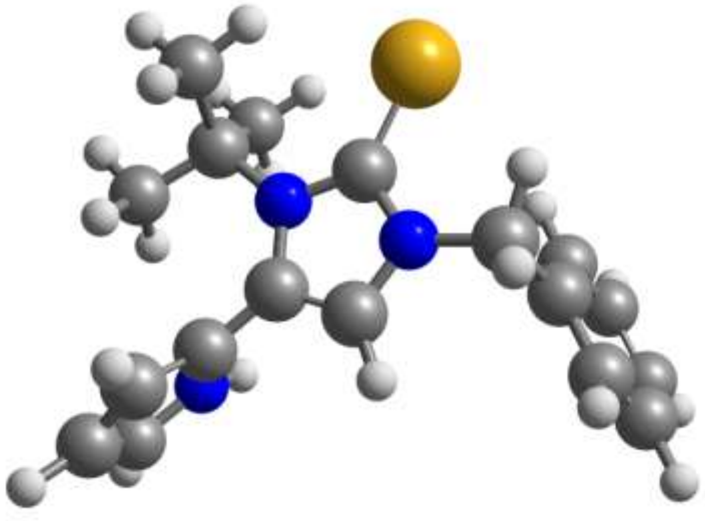

Charge: 0
E: - -3264.58403647608 hartrees

	X	Y	Z
C	-1.466806	-1.785256	-3.770585
C	-2.781835	-1.739564	-3.361481
C	-2.807980	-1.105666	-2.089621
C	-1.510634	-0.767853	-1.756635

N	-0.704234	-1.201341	-2.791647
C	-0.959617	-0.168157	-0.538056
C	-0.691746	-0.879503	0.582940
N	-0.183502	-0.028406	1.531423
C	-0.113286	1.247804	1.031485
N	-0.600249	1.179416	-0.261706
Se	0.548832	2.674314	2.032786
C	-0.730990	2.390780	-1.178941
C	0.213049	-0.454723	2.880265
C	1.437423	-1.343429	2.881866
C	0.672073	2.969117	-1.431683
C	-1.339348	2.030588	-2.543105
C	-1.672332	3.412157	-0.516037
C	2.693736	-0.816666	2.556461
C	3.821042	-1.633181	2.562069
C	3.710975	-2.984619	2.894110
C	2.466531	-3.514591	3.224160
C	1.336803	-2.695614	3.217745
H	-1.017455	-2.193477	-4.661367
H	-3.626932	-2.123703	-3.910975
H	-3.676748	-0.900745	-1.483192
H	0.300559	-1.129966	-2.797377
H	-0.818980	-1.933886	0.759153
H	-0.637476	-0.975839	3.326775
H	0.392394	0.465159	3.439580
H	1.303570	2.230193	-1.934019
H	1.155691	3.277834	-0.506877
H	0.584485	3.840327	-2.085830
H	-0.725409	1.336188	-3.115572
H	-2.347634	1.626941	-2.466064
H	-1.394968	2.961292	-3.112465
H	-1.780038	4.275696	-1.177371
H	-2.662198	2.971736	-0.368061
H	-1.290637	3.755753	0.442886
H	2.780211	0.236030	2.308385

H	4.789787	-1.213256	2.314527
H	4.591496	-3.617236	2.900497
H	2.372399	-4.562139	3.488094
H	0.369978	-3.112509	3.483459

Geometry optimization 1-Benzyl-3-methyl-4-(1H-pyrrol-2-yl)-1,3-dihydro-2H-imidazole-2-selenone (9e):

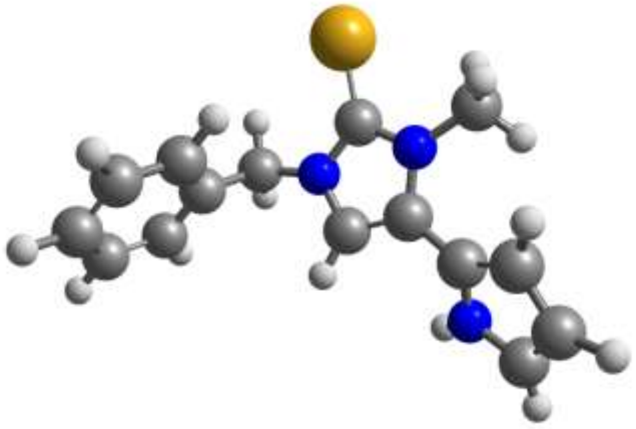

Charge: 0
E: - -3146.627646 hartrees

	X	Y	Z
C	-2.292020	-1.130680	-3.769749
C	-2.812616	0.091994	-4.132830
C	-2.367601	1.045482	-3.179341
C	-1.580709	0.380833	-2.251960
N	-1.552577	-0.948427	-2.629230
C	-0.911265	0.815495	-1.039750
C	-0.826979	0.181298	0.161959
N	-0.055814	0.963658	1.003822
C	0.345891	2.101756	0.354143
N	-0.177613	2.001777	-0.909133
Se	1.384226	3.472512	1.028286
C	0.049310	2.988202	-1.957184
C	0.247408	0.637070	2.398896
C	1.033898	-0.647630	2.543682
C	2.289901	-0.780806	1.939338
C	3.017956	-1.957970	2.083413

C	2.505027	-3.015573	2.837065
C	1.260046	-2.888579	3.446486
C	0.528543	-1.709414	3.296907
H	-2.377227	-2.101205	-4.230705
H	-3.447376	0.279766	-4.984460
H	-2.622086	2.093206	-3.150907
H	-0.989094	-1.651826	-2.179171
H	-1.274791	-0.740883	0.489268
H	1.037759	3.421555	-1.811834
H	-0.015612	2.495326	-2.925478
H	-0.691108	3.790499	-1.903634
H	-0.692429	0.574571	2.954191
H	0.810647	1.491774	2.781624
H	2.695252	0.043662	1.362092
H	3.991221	-2.049613	1.614021
H	3.075817	-3.930649	2.949092
H	0.855639	-3.703714	4.036132
H	-0.441626	-1.613469	3.774949

References

[1] Gorjian, H.; Khaligh, N.G. 4,4′-Trimethylenedipiperidine, a safe and greener alternative for piperidine, catalyzed the synthesis of *N*-methyl imines. *Res Chem Intermed* **2022,** *48*, 2035–2045. DOI: 10.1007/s11164-022-04680-2

[2] Chen, G.-H.; Leu, W.-J.; Guh,J.-H., Lin; C.-H., Huang,J.-H. Synthesis, characterization and cancer cell growth inhibition activity of ruthenium(II) complexes bearing bidentate pyrrole-imine ligands. *J. Organomet. Chem.* **2018**, *868*. 122-130. DOI: 10.1016/j.jorganchem.2018.05.012

[3] Sisko, J.; Mellinger, M.; Sheldrake, P. W.; Baine, N. H.; Dong, Y.; Wolff, S. α-TOSYLBENZYL ISOCYANIDE. *Org. Synth.* **2000**, *77*, 198. DOI: 10.15227/orgsyn.077.0198

[4] Gallati, C. M.; Goetzfried, S. K.; Ausserer, M.; Sagasser, J.; Plangger, M.; Wurst, K.; Hermann, M.; Baecker, D.; Kircher, B.; Gust, R. Synthesis, characterization and biological activity of bromido3-ethyl-4-aryl-5-(2-methoxypyridin-5-yl)-1-propyl-1,3-dihydro-2H-imidazol-2-ylidenegold(i) complexes. *Dalton Trans.*, **2020**,*49*, 5471-5481. DOI: 10.1039/C9DT04824C

[5] Prasad, C. D.; Balkrishna, S. J.; Kumar, A.; Bhakuni, B. S.; Shrimali, K.; Biswas, S.; Kumar, S. Transition-Metal-Free Synthesis of Unsymmetrical Diaryl Chalcogenides from Arenes and Diaryl Dichalcogenides. *J. Org. Chem.* **2013**, *78*, 1434 – 1443. DOI: 10.1021/jo302480j

[6] (a) Ramsden, C. A.; Oziminński, W. P. Quantitative Index of the Relative Ease of Formation and σ-Bonding Strength of N-Heterocyclic Carbenes. *J. Org. Chem.* **2016**, *81* (21), 10295–10301. DOI: 10.1021/acs.joc.6b01304 ,

(b) Ramsden, C. A.; Oziminński, W. P. Quantitative Index of the Relative Ease of Formation and σ-Bonding Strength of N-Heterocyclic Carbenes. *J. Org. Chem.* **2016**, *81* (21), 10295–10301. DOI: 10.1021/acs.joc.6b01304 .

(c) Oziminński, W. P.; Ramsden, C. A. The Influence of Substituent Field and Resonance Effects on the Ease of N-Heterocyclic Carbene Formation from Imidazolium Rings. *RSC Adv.* **2018**, *8* (27), 14833–14837. DOI: 10.1039/c8ra02526f.
